# Pseudo-resonance structures in chiral alcohols and amines and their possible aggregation states

**DOI:** 10.3389/fchem.2022.964615

**Published:** 2022-08-29

**Authors:** Huajie Zhu, Shengnan Li, Yunjing Jia, Juxing Jiang, Feiliu Hu, Longfei Li, Fei Cao, Xiaoke Wang, Shenhui Li, Guanghui Ouyang, Gengfang Tian, Ke Gong, Guangjin Hou, Wei He, Zheng Zhao, Charles U. Pittman, Feng Deng, Minghua Liu, Kai Sun, Ben Zhong Tang

**Affiliations:** ^1^ School of Chemical and Pharmaceutical Engineering, Hebei University of Science and Technology, Shijiazhuang, China; ^2^ Institute of Life Science and Green Development, Hebei University, Baoding, China; ^3^ Kunming Institute of Botany CAS, Kunming, Yunnan, China; ^4^ State Key Laboratory of Magnetic Resonance and Atomic and Molecular Physics, Wuhan Institute of Physics and Mathematics CAS, Wuhan, Hubei, China; ^5^ Institute of Chemistry, Chinese Academy of Sciences, Beijing, China; ^6^ Neutron Scattering Laboratory, Department of Nuclear Physics, China Institute of Atomic Energy, Beijing, China; ^7^ State Key Laboratory of Catalysis, Dalian Institute of Chemical Physics CAS, Dalian, China; ^8^ School of Science and Engineering, Shenzhen Institute of Aggregate Science and Technology, The Chinese University of Hong Kong, Shenzhen, China; ^9^ Department of Chemistry, Mississippi State University, Starkville, MS, United States

**Keywords:** x-ray, variable-temperature NMR, ^13^C CP-MAS NMR, crystal, pseudo-resonance structure, different bond lengths in structures in solid state or in solution for the same compound

## Abstract

We now report that some chiral compounds, like alcohols, which are not sterically hindered atropisomers nor epimer mixtures, exhibit two sets of simultaneous NMR spectra in CDCl_3_. Some other chiral alcohols also simultaneously exhibit two different NMR spectra in the solid state because two different conformers, **A** and **B** had different sizes because their corresponding bond lengths and angles are different. These structures were confirmed in the same solid state by X-ray. We designate these as pseudo-resonance for a compound exhibiting several different corresponding lengths that simultaneously coexist in the solid state or liquid state. Variable-temperature NMR, 2D NMR methods, X-ray, neutron diffraction, IR, photo-luminesce (PL) and other methods were explored to study whether new aggregation states caused these heretofore unknown pseudo-resonance structures. Finally, eleven chiral alcohols or diols were found to co-exist in pseudo-resonance structures by X-ray crystallography in a search of the CDS database.

## Introduction

**FIGURE 1:**
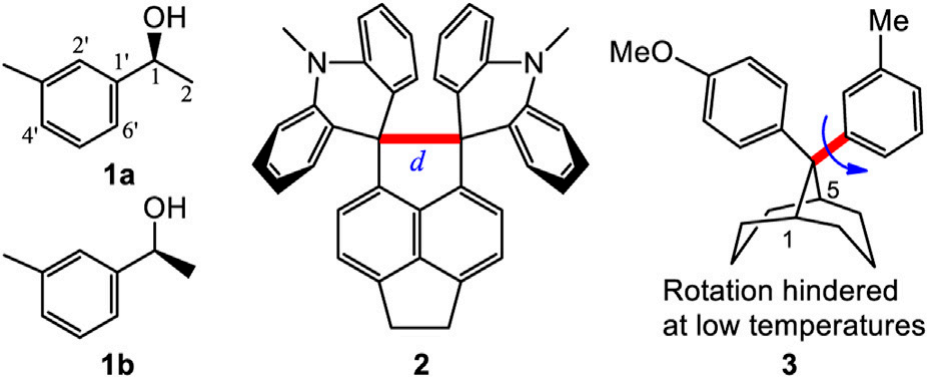
Compounds **1** to **3** and the two rotational conformers **1a** and **1b** of **1**.

The corresponding bonds, e.g. C1-C1′ or C1′=C2′, in the two different rotational conformations **1a** and **1b** of **1** are expected to have almost identical bond lengths (Frisch et al., 2003). For example, the calculated (B3LYP/6-311+G(d)) lengths of C1-C1′ were 1.5161 Å in **1a** and 1.5159 Å in **1b** (Figure 1). Both are very close to 1.516 Å. Similarly, the given internal phenyl ring’s C2′-C1′-C6′ angle, should have almost identical values in these two conformers (calculated 119.05° in **1a** vs. 119.06° in **1b**). This structural similarity concept is generally understood when identifying structures and explains their structural characteristics. However, examples are known where one or two bonds in a molecule may elongate or shorten in different conformations. For example, the C-C bond (red bond in **2**) exhibited lengths of 1.772 Å and 1.712 Å lengths in its two conformations (Kawai et al., 2005). In other cases, especially long bond lengths have been reported, such as the 1.72 Å C-C bond recorded in 1,1,2,2-tetraphenyl-3,8-dichloronaphthenocyclobutene (Toda et al., 1994). In some twisted amides, long N-CO and short C=O bond lengths were found (DusparaMatta, et al., 2001). One of the interesting dihydrogen bonded (DHB) complexes (Burg, 1964.; Brown and Heseltine, 1968; Brown, et al., 1970; Grabowski, 2000), H_2_OH^+^┉HBeH, had a very short calculated H┉H distance, 1.229 Å, between the formally non-bonded O-H and H-Be hydrogen atoms (Grabowski, et al., 2005). One recent report stated that an intramolecular H-bond (Zhao, et al., 2018) interaction led to some carbons appearing as doublets but only one set of ^1^H NMR spectra. These reports reflect the structural complexities encountered in various organic compounds. However, no molecule has been reported in solution, where two different conformations coexist and where several different lengths for the corresponding bonds were shown to exist in each, leading to different two NMR solution spectra occurring simultaneously.

*There are important consequences resulting from the corresponding bonds in two or more conformations of a molecule having almost identical bond lengths.* This results in a molecule having one set of NMR signals, which we call a one-to-one correspondence. This permits its structural identification using NMR, IR and other physical data, which are normally the averaged experimental values. Two different NMR spectra coexist only in the case of atropisomers. This is caused by sterically hindered single bond rotation between its two conformers (Gasparrini et al., 2000). An atropisomer has two NMR spectra which is designated here as a one-to-two correspondence. Pestalotiopsin C at room temperature is such an example (Yu, et al., 2015). However, several of the remaining corresponding bond lengths in such atropisomer pairs do not exhibit different lengths (e.g. alternating elongations or shortenings). Compound **3** produced two separate NMR spectra at low temperature, representing its two different rotational conformations since the single bond (red and bold line) rotational barrier was 7–12 kcal/mol (Casarini, et al., 2003). *Their remaining corresponding bond lengths were almost identical in these two conformers.* This one-to-one or one-to-two correspondence principle is universally used to identify, simulate or explain various molecular structures and their characteristics (Parr and Yang, 1989; Nafie 2011; Berova, et al., 2012; Stephens et al., 2012). To the best of our knowledge, no previous report has shown conformers in which most its bonds have different detectable lengths in different conformers.

If several corresponding bonds, such as C-C, C=C, C-O, C=O, C-N and/or C=N bonds, within two conformers of a molecule had different bond lengths, then these two conformers would differ in size. They would produce two sets of NMR peaks (e.g. two separate and distinct NMR spectra) that could be experimentally observed. We designate this class of conformers as pseudo-resonance structures. If such a case is demonstrated to exist, its two pseudo-resonance structures (alternatively may be called bond length conformer, BLC) would produce not only two NMR spectra but other physical data like two IR spectra as well. This will be called a two-to-two correspondence. In this study, we demonstrate the first existence of such two-to-two correspondences and call these pseudo-resonance structures. They form in new aggregation structures in the solid state, which are characterized here by X-ray, ^13^C cross-polarized magic-angle spinning (CP-MAS) NMR, and also in the liquid state, verified by variable-temperature NMR, 2-D NMR methods, IR spectra, quantum computations and other methods.

## Materials and methods

### General experimental procedures for compounds

The reaction of phthalaldehyde with primary amines afforded **4**, **6–14** in dichloromethane or other solvents with yields of 40–60%. 2-Substituted isoindolin-1-ones were obtained by Dess–Martin oxidations (Dess and Martin, 1991). The recovered yields of the isolated **4** (91% yield), **6**–**14** were up to 90% using HPLC.

Synthesis of **15**–**26** involved the esterification of L*-abrine* and the Pictet-Spengler reactions of L-abrine methyl ester with 2-oxopropanal to generate the intermediate (1*S*,3*S*)-methyl 1-acetyl-2-methyl-2,3,4,9-tetrahydro-1H-pyrido[3,4-b]-indole-3-carboxylate (Dai, et al., 1996). Its reductions using sodium borohydride provided chiral compounds **15**–**16**. Using 2-oxo-2-phenylacetaldehyde in the Pictet-Spengler reaction finally gave compound **17**. Addition of the intermediate with MeMgCl afforded **18**. The additions of MeMgCl to **15** and **16** afforded **19** and **20**, respectively. The amination of **15** and **16** with ammonia produced **22** and **23**, respectively. When the ethyl or isopropyl esters of L-abrine were used in the Pictet-Spengler reactions, compounds **24**–**25** were obtained. Compound **26** was synthesized by sodium borohydride reduction using similar reaction conditions that were applied for synthesis of **15**.

All compounds **4** to **26** were purified by column chromatography using silica gel by solvents of various polarities or their mixtures, after separations based on solvent their polarities were predomonstrated by using TLC.

Synthesis of **38** was performed by two steps. 5-MeO-trypamine reacted with biacetyl to afford the corresponding Pictet-Spengler reaction product, which was used to react with tryptamine in chloroform catalyzed by TFA to give **38** at 60°C.

**DFT computational Methods**. Conformational searches for the corresponding compounds were performed using the MMFF94S force field (Halgren, 1999). These geometries were then used for optimizations at the B3LYP/6-311++G(2d,p) level in the gas phase or in liquid using the PCM model (Mennucci, et al., 2002). Rotation barriers were computed at the B3LYP/6-311++G(2d,p) level in the gas phase and in liquid (CHCl_3_) using the PCM model. The potential energy scans (PES) were performed by rotating the single C3-C1′ bond in incremental steps of 5°. Gaussian software was used. The lowest energy geometry was used as the initial structure. The B3LYP/6-311++G(2d,p) was used in PES calculations in the gas phase. A total of 71 steps with 355° (71×5=355) were calculated.

## Results and discussion

### Discovery of bond length conformers

**FIGURE 2:**
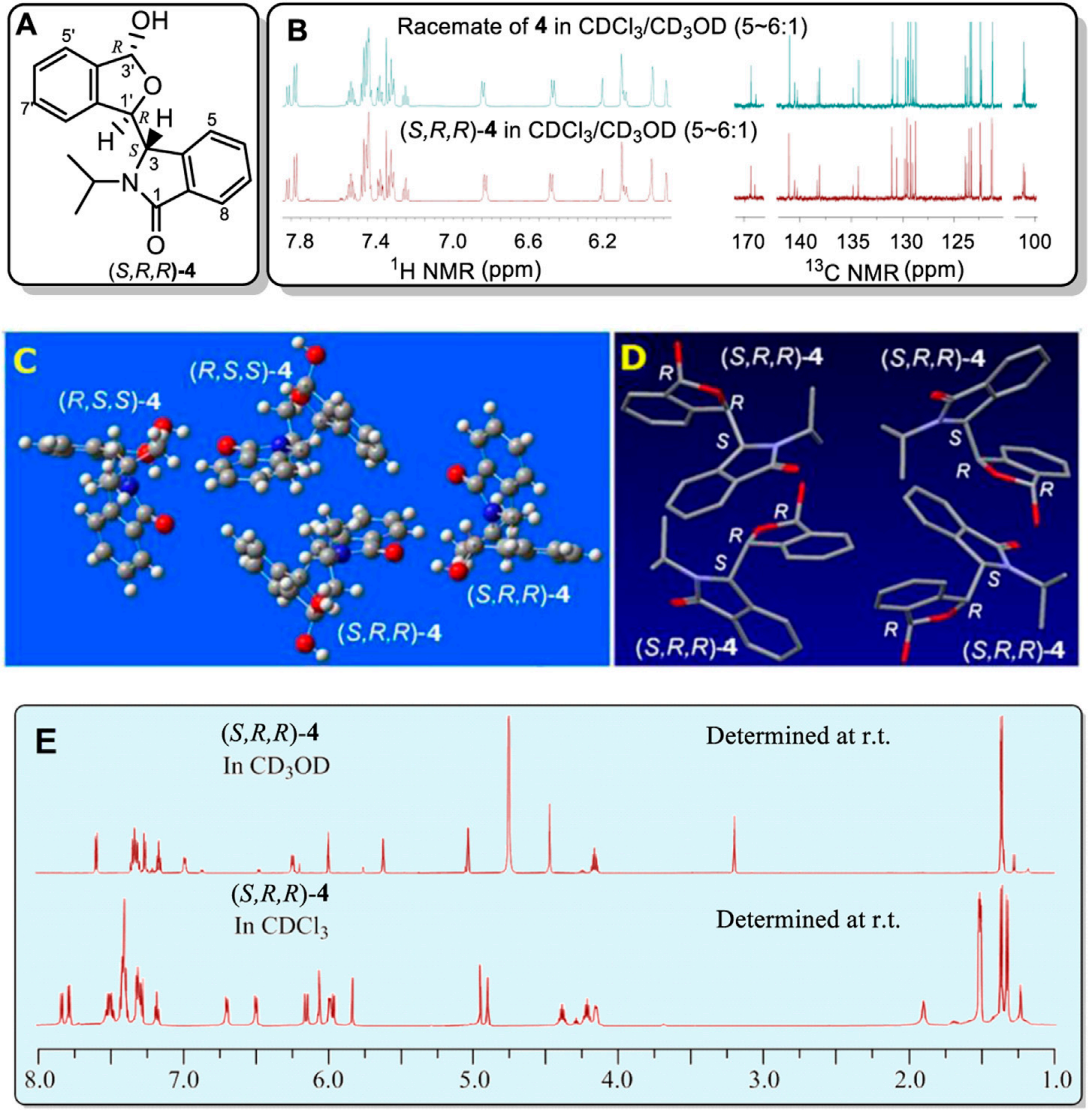
**(A)** Structure of (*S,R,R*)-**4**. **(B)** Partial sections of the ^1^H and ^13^C NMR for the racemate of **4** in CDCl_3_/CD_3_OD mixture solvent (v/v, 5–6:1) (top) and the single enantiomer, (*S,R,R*)-**4**, (bottom) in a CDCl_3_/CD_3_OD mixture solvent (v/v, 5–6:1). **(C)** X-ray structure of racemic **4**. Two (*R,S*,*S*)-**4** and two (*S,R*,*R*)-**4** molecules occupy one unit cell. The corresponding bonds of these two configurations had the same bond lengths. **(D)** Four conformers of (*S,R,R*)-**4** were included in the unit cell. These four which have identical bond lengths (H atoms are hidden for clarity) in the solid structure. **(E)** Comparison of the ^1^H NMR spectrum at room temperature of single enantiomer (*S,R,R*)-**4** in CD_3_OD with that in CDCl_3_. The signals at 3.21 and 4.75 ppm are due to CH_3_OH in the CD_3_OD.

While investigating enantioselective reactions of phthalaldehyde and amines (DoMinh, et al., 1977; Bai, et al., 2012), we obtained pure 3-(3-hydroxy-1,3-dihydroisobenzofuran-1-yl)-2-isopropylisoindolin-1-one (**4**) as the major product (Eq. 1) (Figure 2A, also *see* Supplementary Figures S1–S8). Its racemate was confirmed by X-ray crystallography to consist of two enantiomers (3*S,*1′*R,*3′*R*)-**4** and (3*R,*1′*S,*3′*S*)-**4** (Figure 2C, also *see* Supplementary Figure S6 and Supplementary Table S1. These designations were condensed to (*S,R,R*)-**4** and (*R,S,S*)-**4** for reading and writing ease). Interestingly, the racemate has two different sets of solution NMR peaks (two distinct NMR spectra) in both its experimental ^1^H and ^13^C NMR spectra (Supplementary Figures S1, S2). These were present in a 1:2 a mole ratio in CDCl_3_/CD_3_OD (v/v, 5–6:1). In contrast, racemic **4** had only one set of NMR signals in CD_3_OD (Figure 2E, also *see* Supplementary Figures S7, S8). 

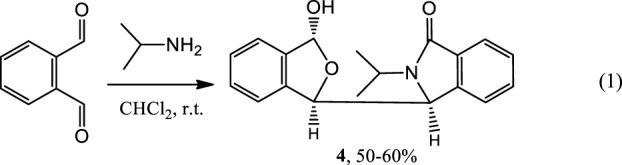

(1)



Next, the single (*S,R,R*)-**4** enantiomer was isolated from the racemate by chiral HPLC in order to determine if it would exhibit this same phenomenon (Supplementary Figures S9–S11). Its (*S,R,R*)-**4** configuration was confirmed by X-ray (Figure 2D, also *see* Supplementary Figure S11 and Supplementary Table S2). Remarkably, (*S,R,R*)-**4** also exhibited two simultaneous different ^1^H and two ^13^C NMR spectra, each in a 1:2 mol ratio, in CDCl_3_/CD_3_OD (v/v, 5–6:1, Supplementary Figure 2B) but almost 1:1 in pure CDCl_3_ (Supplementary Figures S9, S10). Like racemic **4**, a single set of ^1^H NMR peaks appeared for (*S,R,R*)-**4** in CD_3_OD (Supplementary Figures S9A, S10A) and this spectrum matched that of racemic **4** in CD_3_OD (Supplemental Figures S7, S8). Very weak signals (near 5% based on integrations) were recorded in CD_3_OD that have been excluded as any impure compound or its epimor later using various methods. The ^1^H NMR differences at room temperature between (*S,R,R*)-**4** in neat CD_3_OD and (*S,R,R*)-**4** in neat CDCl_3_ are illustrated in Figure 2E. To our best knowledge, this is the first reported case where a racemate (e.g. **4**) or one of its enantiomers had a single NMR spectra in one solvent (CD_3_OD) but two separate NMR spectra in another (CDCl_3_).

### The excluded possibilities

**SCHEME 1:**
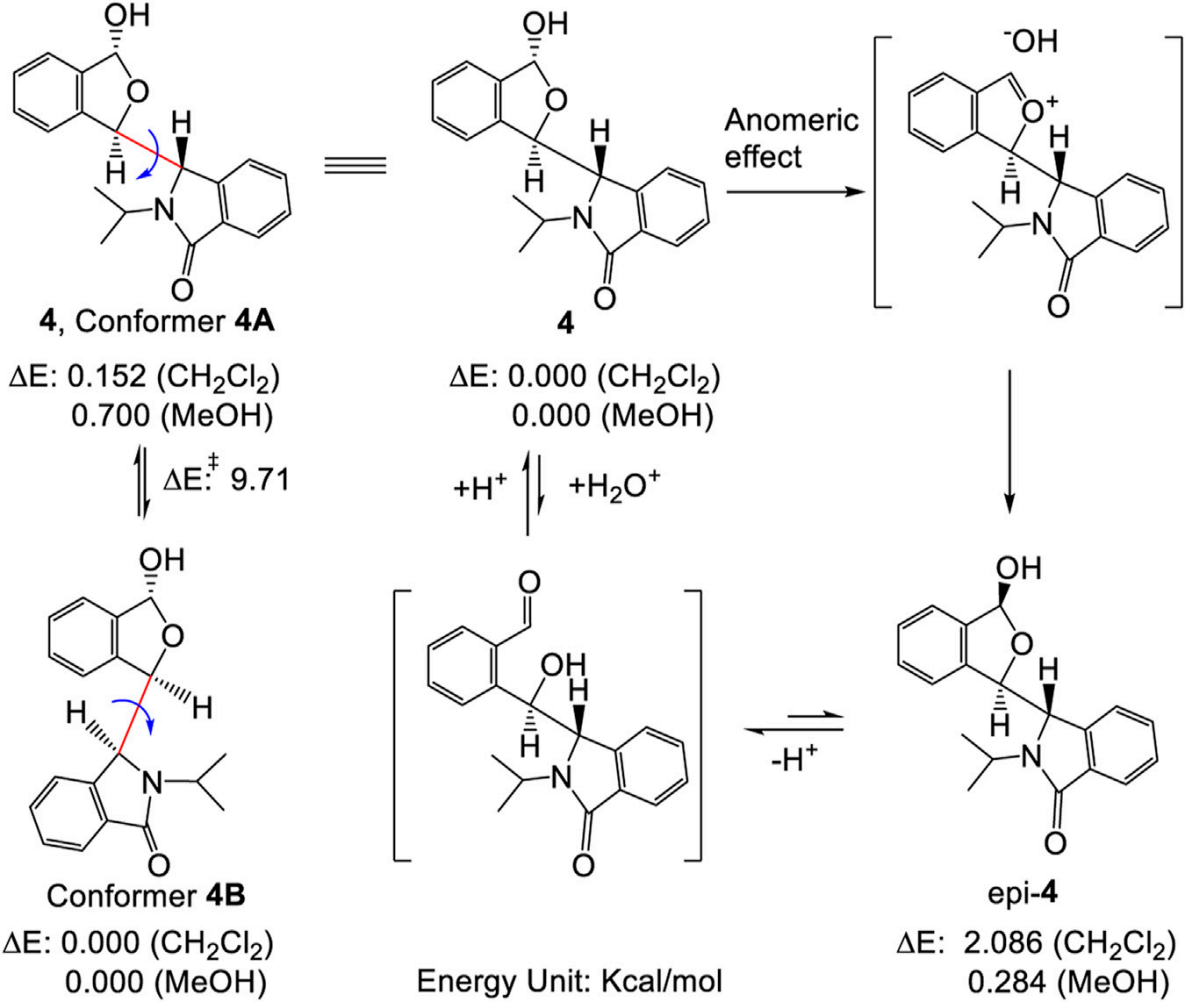
Inter-conversion between two stable conformers **4A** and **4B** (left), also illustrating the plausible formation of epi-**4** *via* either hydrolysis or an anomeric effect (right, *see* refs. 21 and 22 for mechanistic details). The energy differences shown here were from computations performed at the B3LYP/6-311++G(2d,p) level using PCM model. *See* Supplementary Material for details.

**FIGURE 3:**
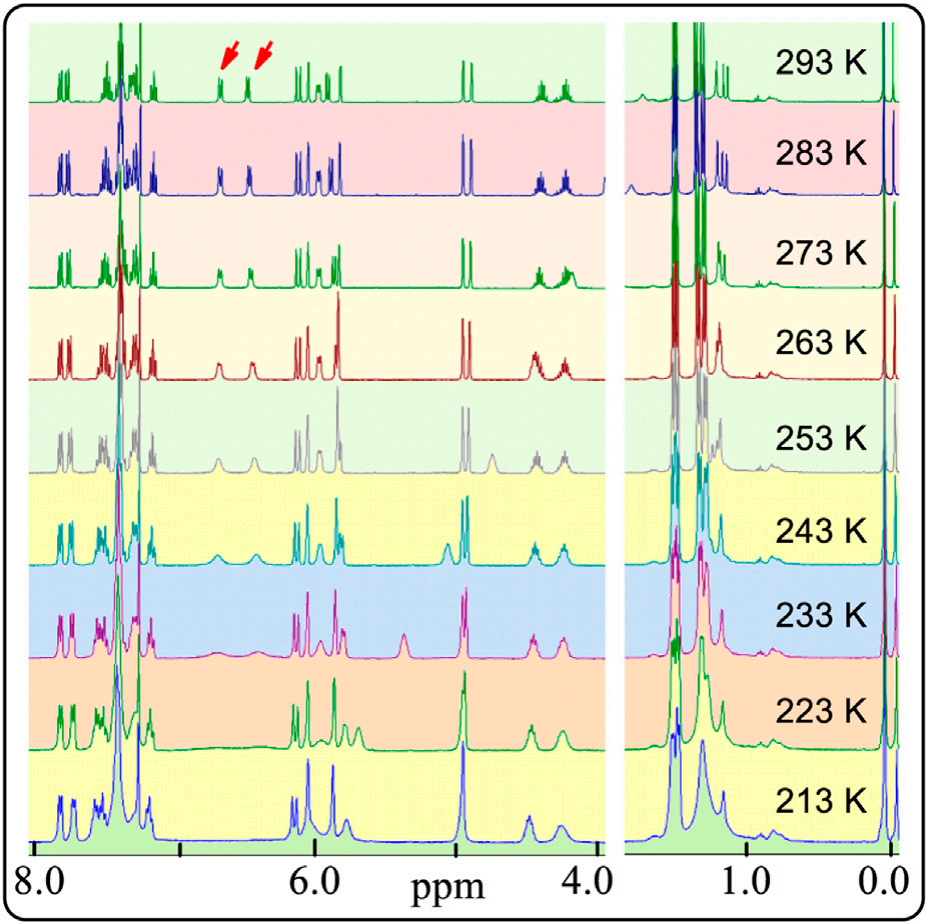
Partial ^1^H NMR spectra of (*S,R,R*)-**4** in CDCl_3_ from 293 to 213 K.

As mentioned above, sterically hindered atropisomers have two sets of NMR peaks (one-to-two correspondence) at room temperature. The rotation barrier of single bond C3-C1′ of **4** might be large enough to block its rotation similar to that of pestalotiopsin C (19.6 kcal/mol), earlier investigated using quantum method (Yu, et al., 2015)^11^ However, **4** had two set of simultaneous ^1^H NMR at room temperature and the rotational barrier between **4A** to **4B** was low, ranging from 9.2 to 9.9 kcal/mol (Scheme 1, also *see* Supplementary Figure S12 and Supplementary Tables S3, S4) using quantum calculations (Foresman and Frisch, 1996; Zhu, 2015). This barrier was close to that of compound **3** (7–12 Kcal/mol) which exhibited two sets of NMR spectra at a very low temperature instead of room temperature (Casarini, et al., 2012). Furthermore, if the two sets of NMR signals in either racemic **4** or (*S,R,R*)-**4** were really produced by the single bond rotation restriction (atropismer) at room temperature, it would indicate that the exchange rate of the nuclei was very slow based on NMR time scale. When the temperature decreased, the exchange rate must be slower than that at room temperature. Thus, the observed ^1^H NMR signals must become much sharper and clear or a singlet may split into two signals at low temperature. However, at −60°C in variable-temperature NMR experiments, (*S,R,R*)-**4** NMR signals neither split into two separate sets of peaks nor became a sharper signal as expected (Figure 3, also *see* Supplementary Figure S13). Instead, most of the doublets or multiple signals changed into single and wide signals (Figure 3 also *see* Supplementary Figures S13, S14).

This is opposite of the well-known behaviour of atropisomer in variable-temperature NMR experiments. Amazingly, the two signals at 6.4 and 6.8 ppm consisting of two peaks each became wider and wider. Finally, both signals appeared as a line after the measured temperature decreased to 223 K. Indeed, these integrations of the two protons are close to an area of two (Supplementary Figure S14, p44 to p50 in Supplementary Material). The reason for this could be that the resonance frequencies range of the two protons become wider and wider when the temperature decreased. These observations conflict with the usual common sense interpretations of the ^1^H NMR behaviour of atropisomers from room temperature to low temperatures.

Other evidence also confirmed that these two sets of NMR peaks were not produced by the mixtures of **4** and its epimer (epi-**4**) (Scheme 1) (Lemieux, et al., 1969; Juaristi and Cuevas, 1992). First, the single optically pure enantiomer, (*S,R,R*)-**4**, was obtained by chiral HPLC in ethanol/hexane at room temperature; it gave a single chromatographic peak at room temperature under these chiral HPLC analysis conditions (Supplementary Figure S3). Therefore, this enantiomer was stable throughout the complete separation procedure. Second, if **4** and epi-**4** coexisted in solution caused by an anomeric effect as reported (Lemieux, et al., 1969), the 1 to 1 ratio of **4** to epi-**4** would have to exist in a polar solvent like methanol instead of a non-polar solvent. Indeed, the ∼1:1 ratio of peaks in the NMR was recorded in CDCl_3_, a lower polarity and non-protonic solvent in our study. Thus, it is not an anomeric effect.

Compound **4** might have self-assembled in CDCl_3_ solution to form an aggregated species with two different NMR spectra of **4**. The H-bonded self-assembly of dipeptidyl urea was observed in CD_2_Cl_2_ solvent at about 1 × 10^−2^ M concentration (Moriuchi, et al., 2002). It formed two sets of NMR signals under low temperatures at this concentration as expected. Self-assembly equilibria may not be observed at very low concentrations (Mekhalif, et al., 2003; Wu and Isaacs, 2003). In contrast, racemic **4** and its single (*S,R,R*)-**4** enantiomer both exhibited two completely independent NMR sets of signals in CDCl_3_ at a concentration of only 7.4 × 10^−6^ M at room temperature (Supplementary Figure S15). Importantly, the ^1^H NMR spectral changes (Figure 3) reversed the expected results as mentioned above in the variable-temperature NMR study of (*S,R,R*)-**4**. Therefore, the observed two sets of NMR peaks for (*S,R,R*)-**4** could not be caused by what is normally seen and reported for self-assembled structures that are in equilibrium.

### Scope of the phenomenon

**FIGURE 4:**
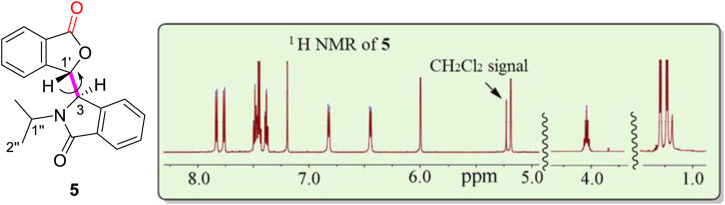
One ^1^H NMR spectrum at room temperature for racemic **5** (signal-free regions of the spectrum are hidden for clarity). There are a total of eight aromatic proton signals from 6.4 to 7.9 ppm, two isolated protons (H3, 5.19 ppm, H1′, 6.00 ppm), two methyl groups (H2″, 1.1 ppm) and one proton (H1″, 4.0 ppm), respectively. Residual CH_2_Cl_2_ remained from the isolation of **5** and was recorded at 5.25 ppm in the ^1^H NMR spectrum.

The –OH function in **4** may play an important role in the formation of the two sets of NMR signals. To determine the effect of this –OH, racemic lactone **5** was synthesized by oxidization of **4** using PCC. Compound **5** had just a single NMR spectrum in CDCl_3_ at room temperature (Figure 4, also *see* Supplementary Figures S16, S17) instead of two coexisting sets of NMR spectra. The calculated rotation barrier around C3-C1' (bold pink line) was approximately 10–11 kcal/mol using different computational methods (Supplementary Figure S18 and Supplementary Table S5) (Zhu, 2015). This range of calculated barriers was slightly higher (by 1 kcal/mol) than the maximum calculated C3-C1′ rotational barrier between conformer **4A** to **4B** (Scheme 1). This confirms that the two coexisting NMR spectra for **4** at room temperature in CDCl_3_ could not be caused by sterically hindered rotation about C3-C1'. Thus, the –OH group in **4** had to play a major role in the process leading to the two NMR spectra of **4** observed in CDCl_3_.

Nine other related hemiacetal racemates, **6** to **14**, were synthesized (Eq. 2) to further investigate this phenomenon. All nine of these racemates exhibited two simultaneous NMR spectra at room temperature in CDCl_3_ versus just a single NMR spectra in CD_3_OD (Supplementary Figures S19–S54)! When the size of the substituent on *N* is small, the signal strength of one of the two ^1^H NMR spectra was small indicating a smaller amount of that fraction. Compare **6** and **7**, which had an -Et and -*n*Pr group, respectively, on the *N* atom. Once the substituent on the *N* atom increased to -*n*Bu, the signal strengths of the second NMR spectrum increased quickly (near 50%). The same phenomenon is causing this behaviour in **4** and in **6–14**, including the changes in the minor NMR signals recorded in CD_3_OD. A bulky groups, such as the *N*-substituted *t*-butyl group in **9**, may increase the rotation barrier around C3-C1′. However, the calculated C3-C1′ rotational barrier of **9** was still only about 14.07 kcal/mol (Supplementary Table S6). This barrier is obviously not sufficient to block C3-C1′ rotation at the NMR time scale at room temperature. This observation of two different coexisting NMR solution spectra observed for **4** and **6**–**14** is a new phenomenon. It is not isolated to **4**. For clarity in this discussion, we simply described that only the one ^1^H NMR spectrum in CD_3_OD and two coexisting NMR spectra in CDCl_3_. 

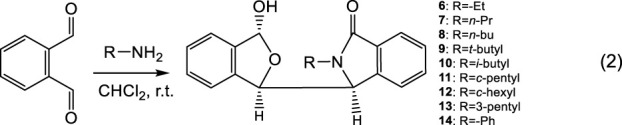

(1)



These experimental results for **6**–**14** further confirm that the two simultaneous and different NMR spectra of **4** in CDCl_3_ were not caused by either a sterically hindered rotation or by mixtures of **4** and epimer (epi-**4**) formed by hydrolysis or an anomeric effect. The recorded NMR data are the averaged values of all the atoms in all their corresponding positions. Therefore, the two NMR spectra recorded per each compound in these experiments, in the absence of any alternative that we have thought of, seems to be produced by an aggregation state in which two non-equivalent molecules of the structure **4** exist. In this aggregation state, one molecule of **4** produces one room temperature NMR spectrum, while another similar, but different structure of **4** produces the second similar NMR spectrum, in which some peaks overlapped. This general explanation also applies to **6**–**14**.

The same aggregation state formed when racemic **4** or its single enantiomer, (*S,R,R*)-**4**, is dissolved in CDCl_3_. What might this aggregation state look like? An answer is prompted by the structures of three tetrahydro-β-carbolines, **15** to **17**, (Figure 5, also *see* Supplementary Figures S55–S68 and Supplementary Tables S7–S9) prepared almost 15 years ago during chiral catalyst syntheses using L-*abrine* (Zhu, et al., 2005)*.*

**FIGURE 5:**
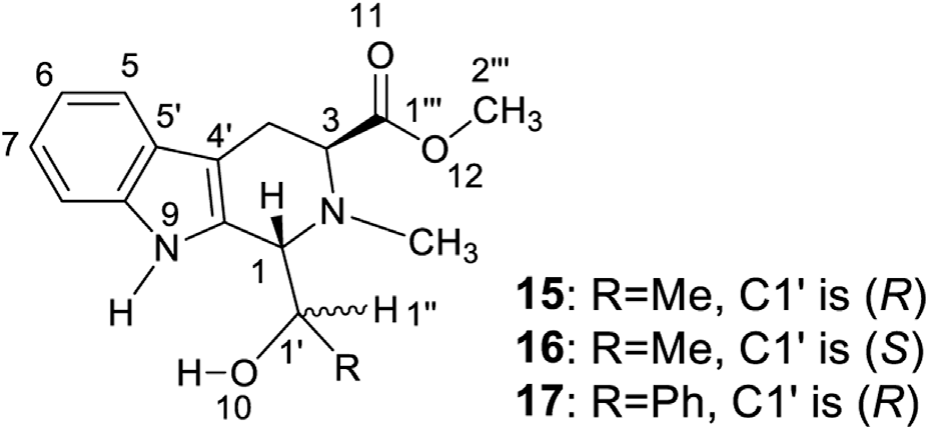
Synthesized tetrahydro-β-carbolines **15** to **17**.

**FIGURE 6:**
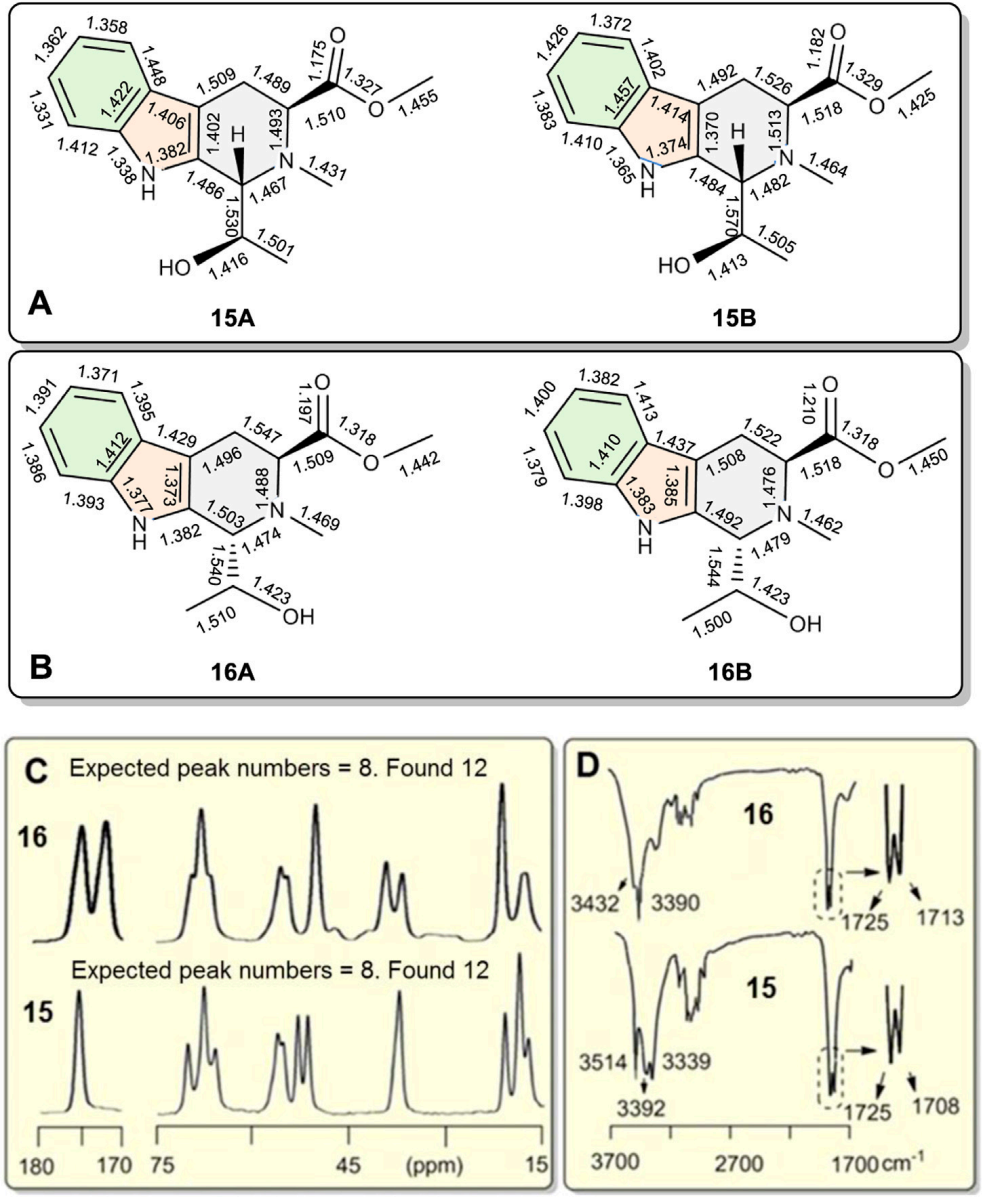
**(A**,**B)** The bond-length differences from single crystal X-ray diffractions of coexisting conformations **15A** vs. **15B**, and **16A** vs. **16B**, respectively. Conformations **15A** and **16A** are slightly smaller than **15B** and **16B**, respectively. **(C)** Partial ^13^C CP-MAS NMR spectra of **15** and **16** in the solid state. A total of 12 singlet carbon signals were recorded for both **15** and **16** indicating separate and distinct solid state NMR spectra exist for **15A**/**15B** as well as **16A**/**16B.** Each had just eight carbon resonances in the 15–75 ppm and 170–180 ppm ranges. **(D)** Partial IR spectra of **15** and **16**. Two stretching absorptions appeared in the corresponding C=O bonds in crystals of both **15** and **16**.

An X-ray study of **15** confirmed two similar solid state conformers of **15** co-existed in the solid state, each of which had different bond lengths and angles in their crystal structure (Figure 6A and Table 1 also *see* Supplementary Table S7). *Each of the corresponding bonds of these conformers* **15A** and **15B**, *including C=C, C-C, C=N C-N, C=O and C-O, had substantially different lengths* rather than almost the same bond lengths (Table 1). For example, C6=C7 bond had 1.362 Å length in **15A** while the corresponding C6=C7 length was 1.426 Å in **15B**. This C6=C7 bond length difference was remarkably large at 0.064 Å between the **15A** and **15B** geometries (Table 1, entry 8). Interestingly, many bonds in **15A** are a shorter than the corresponding bonds in **15B** (Supplementary Tables S10–S12). Thus, the geometry **15A** is a little smaller than **15B**. Similarly, two **16A** and **16B** solid state geometries co-existed (Figure 6B and Table 1, also *see* Supplementary Tables S8, S10–S12). Since recorded NMR data are averaged values for each nucleus, the peak locations depend on all of the molecules’ atomic positions in space. Based on the X-ray crystal structure data, it was expected that the two geometries **16A** and **16B** in the solid state could exhibit two different solid state NMR spectra for each. As expected, two distinct sets of ^13^C cross-polarized magic-angle spinning (CP-MAS) NMR spectra were observed for both **15** and **16** (Supplementary Figures S57, S60). Some signals for these different “bond length conformations” overlapped in **15** and in **16**, but others differed. For example, the ^13^C ester C=O resonances of **15A** and **15B** had one overlapped signal at 175.3 ppm. However, in **16A** and **16B**, two separated ^13^C ester C=O peaks appeared at 175.0 and 172.3 ppm (Figure 6C), respectively. Similarly, the different bond lengths of the ester C=O in **15A** (1.176 Å) versus **15B** (1.182 Å) suggested two IR carbonyl stretching frequencies would be detected. Indeed, the recorded IR (crystal **15** powder in a KBr pellet) displayed two carbonyl signals at 1708 and 1725 cm^−1^, respectively (Figures 6C,D) (Supplementary Figures S64, S65), a difference of 17 cm^−1^. The IR signals of some corresponding functional groups overlapped and others did not. Similar crystallographic, solid state NMR and IR results were found for **16** (Figure 6 and Table 1) and **15**.

**TABLE 1:** The bond lengths of compounds **15** to **16** and bond length differences of the corresponding bonds in two geometries **A** and **B**.

Entry	Bond^a^	Bond length (r) (Å)				Δ r (Å)^b^	
		r15A	r15B	r16A	r16B	r15A - r15B	r16A - r16B
1	C1-N2	1.467 (11)	1.483 (11)	1.474 (4)	1.479 (4)	−0.015	−0.005
2	N2-C3	1.493 (10)	1.513 (11)	1.488 (3)	1.476 (3)	−0.020	0.012
3	C3-C4	1.489 (14)	1.527 (12)	1.548 (4)	1.522 (4)	−0.038	0.026
4	C4-C4′	1.509 (12)	1.493 (13)	1.507 (4)	1.495 (4)	0.016	0.012
5	C4′-C5′	1.406 (13)	1.413 (11)	1.429 (4)	1.437 (4)	−0.007	−0.008
6	C5′-C5	1.448 (14)	1.403 (14)	1.395 (4)	1.413 (4)	0.045	−0.018
7	C5-C6	1.358 (16)	1.373 (16)	1.371 (5)	1.381 (5)	−0.015	−0.010
8	C6-C7	1.362 (17)	1.426 (17)	1.391 (5)	1.400 (5)	−0.064	−0.009
9	C7-C8	1.331 (16)	1.363 (14)	1.386 (5)	1.379 (5)	−0.032	0.007
10	C8-C8′	1.412 (13)	1.410 (13)	1.392 (4)	1.398 (4)	0.002	−0.006
11	C8′-N9	1.338 (11)	1.365 (12)	1.377 (4)	1.383 (4)	−0.027	−0.006
12	N9-C9′	1.382 (11)	1.375 (12)	1.382 (3)	1.392 (4)	0.007	−0.010
13	C9′-C1	1.486 (12)	1.484 (11)	1.503 (4)	1.492 (4)	0.002	0.011
14	C1-C1′	1.530 (12)	1.569 (11)	1.540 (4)	1.518 (4)	−0.039	0.022
15	C1′-C2′	1.501 (16)	1.505 (13)	1.509 (5)	1.499 (5)	−0.004^c^	0.010^c^
16	C1′-O10	1.416 (11)	1.413 (11)	1.423 (4)	1.423 (4)	0.003	0
17	N2-C1″	1.432 (11)	1.464 (10)	1.469 (4)	1.462 (4)	−0.032	0.007
18	C3-C1‴	1.510 (12)	1.518 (12)	1.509 (4)	1.518 (4)	−0.008	−0.009
19	C1‴-O11	1.175 (12)	1.182 (11)	1.197 (3)	1.210 (4)	−0.007	−0.013
20	C1‴-O12	1.327 (13)	1.329 (11)	1.316 (3)	1.317 (3)	−0.001	−0.001
21	O12-C2‴	1.454 (12)	1.424 (12)	1.442 (4)	1.449 (4)	0.030	−0.007
22	C4′-C9′	1.406 (12)	1.370 (12)	1.413 (4)	1.410 (4)	0.036	0.003
23	C5′-C8′	1.422 (13)	1.457 (13)	1.373 (4)	1.364 (4)	−0.035	0.009
The averaged difference of the bond length changes						0.019	0.008

a The bond here just means there is a formal bond between the two atoms. It does not mean the bond type. For example, C5-C6 does not mean single bond.

b (r_**15A**_ - r_**15B**_) The designated bond length in **15A** was subtracted from the corresponding bond length in **15B**. (r_**16A**_ - r_**16B**_ **)** had the same meaning as (r_**15A**_ - r_**15B**_).

c The data in parentheses are the estimated standard deviation (esd). The two esd data for each corresponding bond in two conformers are almost the same. However, the esd in different crystal unit cell may be different. For example, the C5′-C8′ had esd values of 13 in the two geometries of **15A** and **15B** (entry 23). But the esd values in the other crystal structure **16A** and **16B** were 4 (entry 23) due to different epimers or crystal form.

The corresponding angles within **15A** and **15B** also had different sizes as seen in their in X-ray structures. One example is the C1-N2-C3 angle, which is 109.5° in **15A** vs. 110.5° in **15B**. All the angle data are summarized in Table 2 (Supplementary Table S11). The largest difference between the corresponding angles of **15A** and **15B** was 3.5° for ∠C5-C6-C7 (entry 14). Thirteen of the seventeen corresponding **15A** and **15B** angles had differences of over 0.50°. Similar cases were recorded for **16A** and **16B**. The maximum angle size difference was 1.32° greater for the C4′-C9′-C1 angle in **16B** versus that in **16A** (entry 5). The analogue **17** (Figure 5) formed a crystalline solid that had just a single solid state NMR spectrum (Supplementary Figure S63). Thus, it was used as a possible reference to see the differences of bond lengths and angles in **15** and **16**.

**TABLE 2:** Partial angles of compounds **15** to **16** and the angle differences of the identical angle in two geometries using the X-ray structures (angle unit in degree).

Entry	Angle	in 15A; in 15B (^o^)	∠_in15A_-∠_in15B_ (°)	in 16A; in 16B (°)	∠_in16A_-∠_in16B_ (°)
1	∠C1-N2-C3	109.5 (6);^a^ 110.5 (6)	−1.0	110.0(2); 110.9(2)	−0.9
2	∠N2-C3-C4	113.2(7); 114.1(7)	−0.9	113.2 (2)	−1.5
				114.7 (2)	
3	∠C3-C4-C4′	109.2(7); 110.0(7)	−0.8	108.6(2); 107.7(2)	0.9
4	∠C4-C4′-C9′	120.6(8); 122.6(8)	−2.0	122.8(2); 122.4(3)	0.4
5	∠C4′-C9′-C1	124.0(8); 124.9(8)	−0.9	124.1(3); 125.4(3)	−1.3
6	∠C9′-C1-N2	110.65(7); 109.8(8)	0.8	110.0(2); 109.5(2)	0.5
7	∠C9′-C4′-C5′	106.4(7); 109.1(8)	−2.7	106.8(2); 107.4(2)	−0.6
8	∠C4′-C5′-C8′	107.9(7); 105.5(7)	2.4	107.2(2); 107.3(2)	−0.1
9	∠C5′-C8′-N9	107.0(8); 106.6(8)	0.4	107.4(2); 107.1(3)	0.3
10	∠C8′-N9-C9′	111.0(7); 110.6(8)	0.3	109.0(2); 109.3(2)	−0.3
11	∠N9-C9′-C4′	107.6(8); 108.2(8)	−0.6	109.5 (2);109.0 (3)	0.5
12	∠C8′-C5′-C5	117.7(9); 117.8(8)	−0.1	119.1(3); 118.7(3)	0.40
13	∠C5′-C5-C6	117.9(9); 121.2(11)	3.3	118.9(3); 118.5(3)	0.4
14	∠C5-C6-C7	122.7(10); 119.2(11)	3.5	121.4(3); 121.4(3)	0.0
15	∠C6-C7-C8	122.4(11); 122.7(8)	−0.3	121.60(3) 121.8(3)	0.2
16	∠C7-C8-C8′	118.9(8); 118.1(9)	0.8	116.9(3); 116.8(3)	0.1
17	∠C8-C8′-C5′	120.2(9); 120.8(8)	−0.6	122.1(3); 122.8(3)	−0.7

a The data in parentheses are the estimated standard deviation (esd). The two esd data for each same angel in two conformers are almost the same. For example, the ∠C8-C8′-C5′ had esd values of 9 and 8 in the two geometries (entry 17). They were very close.

The individual geometries in solid state crystal structures of **15A**, **15B**, **16A**, and **16B**, determined from their X-ray coordinates, were used directly for ^13^C NMR calculations performed *via* quantum methods in the gas phase (Foresman and Fri8sch, 1996). The predicted chemical shift difference between the carbonyl carbons of **15A** and **15B** was 0.5 ppm in the gas phase (Supplementary Table S11). This predicted gas phase carbonyl chemical shift difference between **16A** and **16B** was 3.6 ppm. The experimental carbonyl carbon chemical shift differences for solid state ^13^C were 0 ppm in **15A** vs. **15B** and 2.7 ppm for **16A** vs. **16B** (Supplementary Figure S67 and Supplementary Table S11). These predicted ^13^C NMR chemical shift differences for the corresponding carbonyl carbons vs. those found in experiments (0.5 vs. 0 ppm in **15A** and **15B**, 3.6 vs. 2.7 ppm in **16A** and **16B**) were similar. In contrast, the analogue **17** just had a single molecular geometry in the X-ray structure and it only had one set of CP-MAS ^13^C NMR peaks and a single carbonyl stretching band near 1700 cm^−1^ in its IR spectrum (Supplementary Figure S66). Packing forces play a role in shaping the exact structures of the molecules in the crystal, but it is hard to explain how and why these forces would cause the structurally similar corresponding bonds in **15** and **16**, but not **17**, to form different alternating bond lengths during crystallization from the same solvent.

**FIGURE 7:**
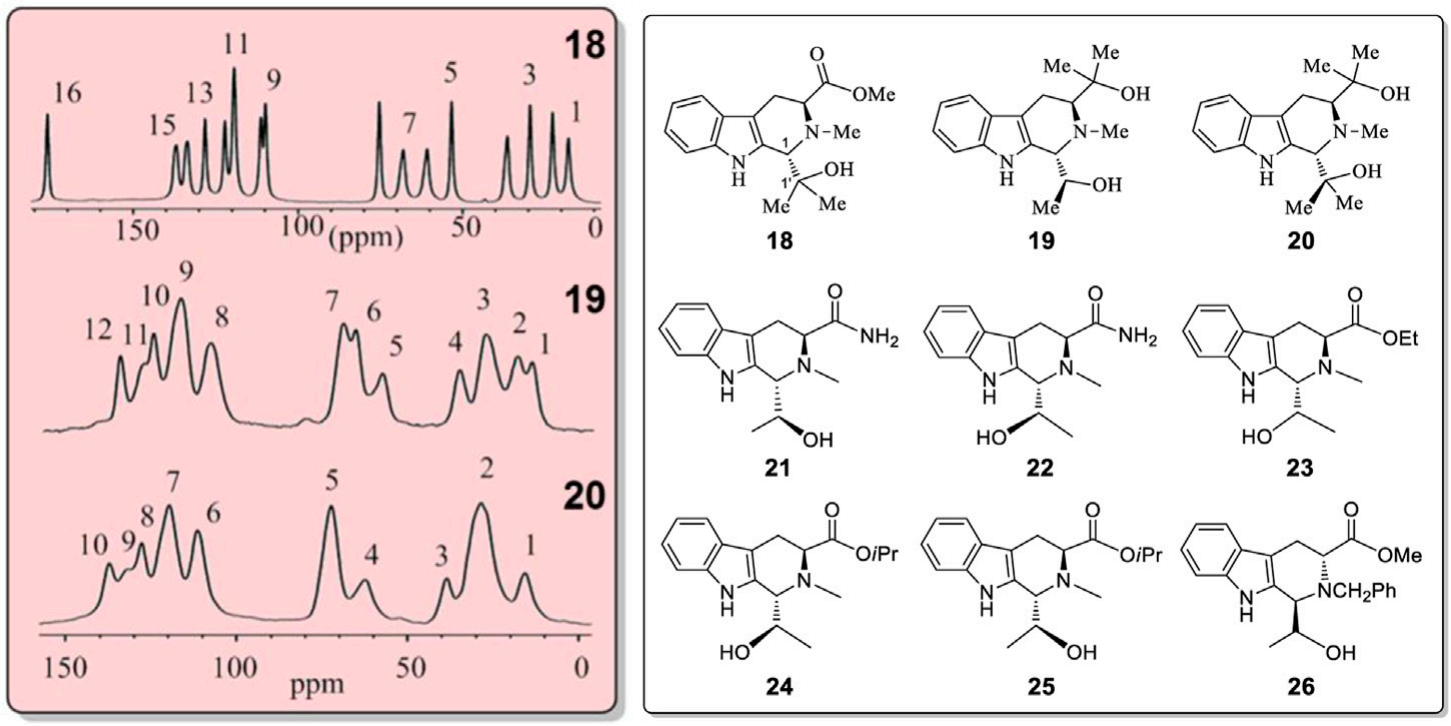
^13^C CP-MAS NMR spectra for compounds **18, 19** and **20** in the solid state. Structures of **18**–**26.**

Compounds **18** to **26** (Figure 7) were synthesized to further investigate this phenomenon (Supplementary Figures S69–S99). Compound **18** had one set of ^13^C CP-MAS NMR peaks in solid state (Figure 7, top; Supplementary Figure S71) and **a single sharp carbonyl stretching frequency** at 1709 cm^−1^ in its solid IR spectrum (Supplementary Figure S79). Thus, no solid state pseudo-resonance structures exist in **18**. However, diols **19** and **20** did have pseudo-resonance structures (or BLCs) in their solid states. The ^13^C CP-MAS NMR spectrum of **19** exhibited 12 broad signals instead of 17 sharp signals (its molecular formula had 17 carbons) (Figure 7, middle NMR, also *see* Supplementary Figure S74). The ^13^C CP-MAS NMR spectrum of **20** had only 10 signals (Figure 7, bottom, also *see* Supplementary Figure S78). These ^13^C sites experienced different but similar magnetic shielding effects, resulting in some signal overlap; they formed broad peaks in **19** and **20**. Furthermore, the X-ray structures of **19** confirmed that there different pseudo-resonance structures (**19A**, **19B**, **19C**) existed in the solid state (also *see* Supplementary Figure S75 and Supplementary Table S13) rather than just two pseudo-resonance structures. Their corresponding bond lengths had longer and shorter bond length differences existing among three different pseudo-resonance structures. Most of the ^13^C CP-MAS NMR signals of **19** were seriously overlapped. This is a more complex spectrum than that in the crystal of **15**. Compound **20** also exhibited overlapped resonances from different coexisting distinct pseudo-resonance structures ^13^C CP MAS NMR spectra. It also has two and more pseudo-resonance structures in its solid state. All six analogues, **21**–**26** (Figure 7 and Supplementary Figures S82–S93), which have *only a single* –OH function, exhibited only a sharp single signal of C=O at near 1700 cm^−1^ in the IR of solid samples (Supplementary Figures S94–S99) instead of two carbonyl stretching bands as found **15** or **16** (Supplementary Figures S64, S65). Furthermore, **21**–**26** have no observable alternating bond length changes.

Compounds **15**, **18**–**20** have quite similar structures. Nevertheless, **15**, **19** and **20** exhibited the existence of different solid state pseudo-resonance structures while **18**, which contains a single -OH group, did not. It is difficult to predict which specific molecular structures will have the several corresponding bonds with different lengths in this series of analogues. To attribute this new solid state structural phenomena exhibited by **4**, **6**–**14**, **15**, **16**, **19** and **20** to packing forces, generally, seems too simple to be reasonable. This is especially true since **4** and **6**-**14** also show this behavior in solution.

**FIGURE 8:**
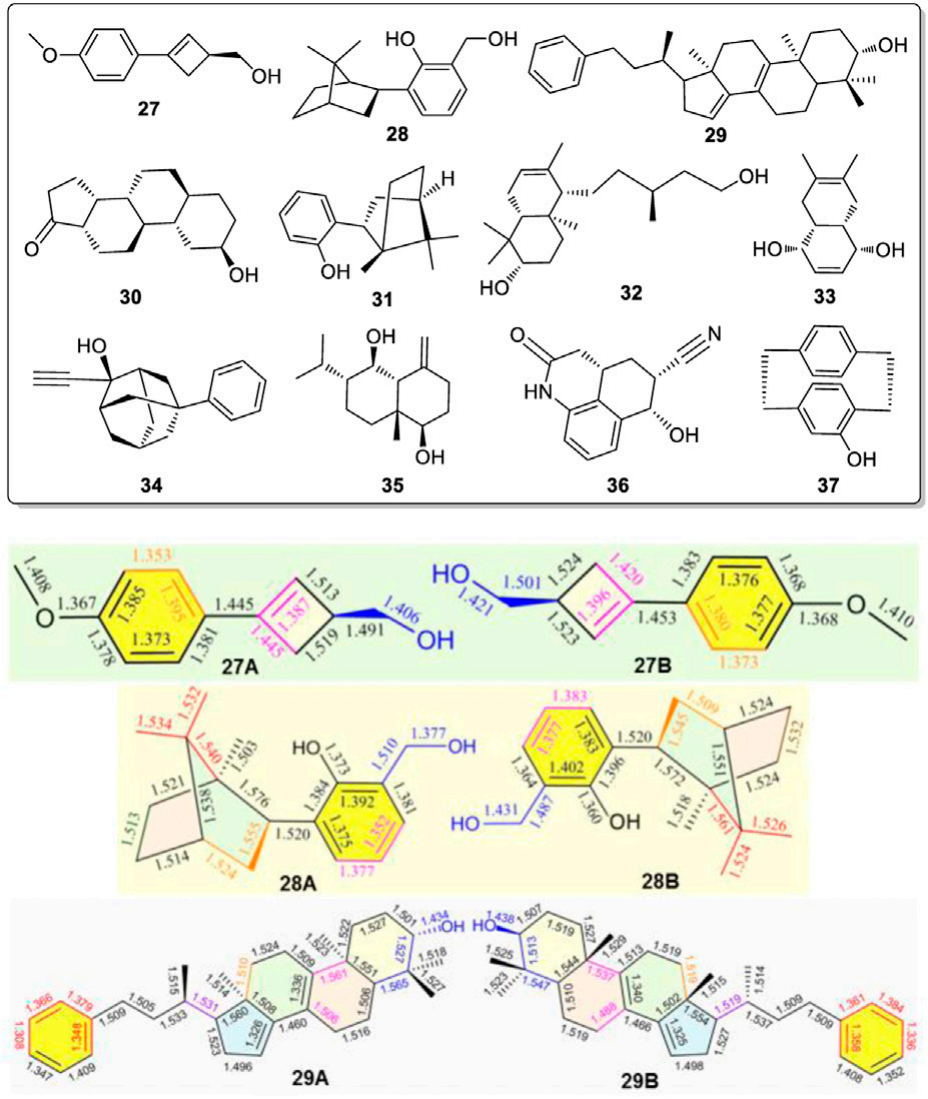
Compounds **27**-**37** all exist pseudo-resonance structures in the solid state. Typical geometric pairs with different solid state bond lengths for the corresponding bonds shown here for compounds **27**, **28** and **29** found co-existing in their single crystal structures.

It is possible that chiral alcohol structures may exist widely, having alternating bond length changes in their corresponding bonds of two or more conformers coexisting in the solid state. To test this possibility, a search for other examples of such conformers was undertaken. The X-ray structures of the CSD database were searched with assistance from X. F. He in the Laboratory of Computer Chemistry of CSS. A total of eleven chiral compounds (**27** to **37**, Figure 8) (Okaya, et al., 1979; Secco and Trotter, 1983; Urones, et al., 1995; Boer, et al., 2001; Chukicheva, et al., 2003; Grosch, et al., 2003; Sun et al., 2004; Chukicheva, et al., 2006; Vorontsova, et al., 2007; Wang, et al., 2007; Tian, et al., 2008) of various structural classes were found, all of which contain one or two -OH groups, where two uniquely different molecular geometries co-existed in their X-ray structures (Figure 8, also *see* Supplementary Figure S100). In each case, the corresponding bonds in these two geometries exhibited the phenomenon of having several alternating and different lengths.

Three of the eleven compounds giving these pseudo-resonance structural pairs, **27A** vs. **27B, 28A** vs. **28B,** and **29A** vs. **29B**, are illustrated in Figure 8 with their corresponding bond lengths given. The sizes of geometries **27A, 28A** and **29A** are each slightly smaller than the corresponding **27B, 28B** and **29B** structures, respectively. Similar results also existed in examples **30** to **37** (Supplementary Figure S100). Obviously, this phenomena widely exists in different kinds of chiral alcohols. Not only did the corresponding bonds in each pair of the molecules from **27** to **37** have significantly different corresponding bond lengths in their two solid state geometries, their bond angles in these rigid moieties also exhibited big differences. Thus, these compounds must also give two sets of ^13^C CP-MAS NMR peaks in solid state. The key and decisive role for forming these pseudo-resonance structures is postulated to result from their aggregation state during and after crystal formation.

Recall that two discrete of NMR spectra simultaneously existed in the liquid phase (CDCl_3_) for **4** and **6** through **14**. If each of these compounds had two different independent molecular structures existing in solution, analogous to **15A** and **15B** existing in the solid state, that would explain the two sets of ^1^H and ^13^C NMR observed in solution for these compounds. The question then becomes how do two such bond length conformations form and continue to co-exist within solution. We have suggested aggregation state structures which might exist within solution to help conceptualize this phenomenon.

## Discussion

**FIGURE 9:**
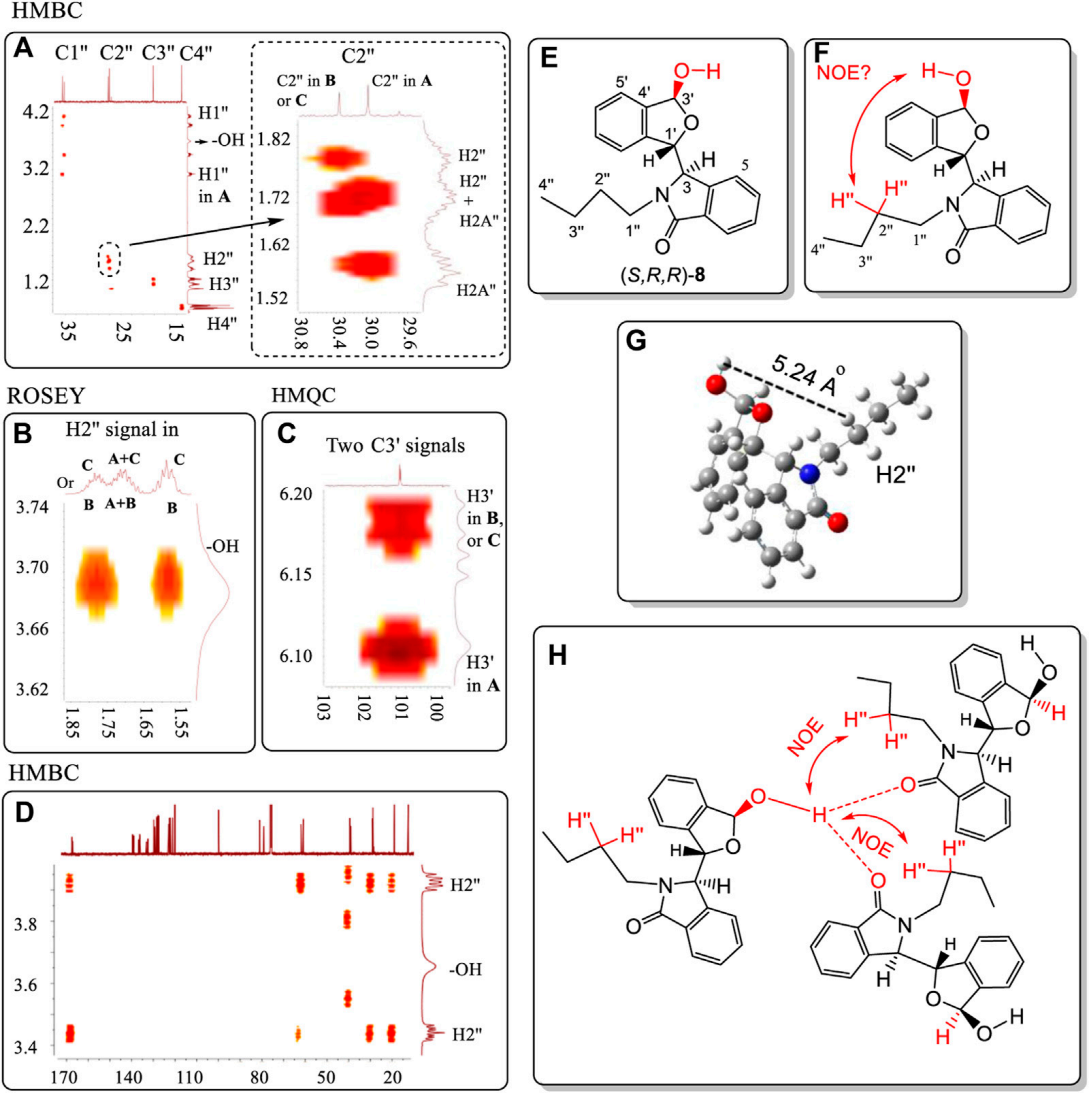
**(A)** HMBC correlations of C1″ to C4″ on the *N* substituent containing the corresponding H1″ to H4″ atoms in two geometries of **8**. **(B)** ROESY correlation of the hydroxyl proton with the H2″ atoms of the *N*-*n*Bu group of **8**. **(C)** HMQC correlations of C3′ with H3′ in the two distinct geometries of **8**. **(D)** HMBC correlations of the –OH proton with all carbons of **8**. **(E)** Numbering of structure **8**. **(F)** No observed intramolecular NOE interaction can occur between the H3′ and H2″ of *n*Bu and the –OH within the same molecule of **8**. **(G)** The distance between the –OH and H2″ is over 5.2 Å. **(H)** The strong intermolecular NOE between the –OH proton and *n*Bu H2″ atoms in two different molecules of **8** was observed (Figure 9B). However, this aggregate structure will lead to the ratio among the three molecules of **8** that is not equal to 1:1. All units in ppm. NMR solvent is CDCl_3_. In summary, the trimer structure like 9H is not likely favored.

Racemic **8** was one of the ten compounds **4**, **6**–**14**, which exhibited two different NMR spectra in CDCl_3_ but only a single spectrum in CD_3_OD. The two sets of NMR peaks had 1:1 area ratio and no peaks from either set overlapped. This was a distinct advantage for studying the proton-proton correlations in ROESY spectra, and proton-carbon correlations in the HMBC and HMQC spectra. The HMBC, HMQC and ROESY spectra of **8** were investigated (Figure 9, also *see* Supplementary Figures S101–S103). Importantly, very strong NOE correlations of –OH proton(s) of **8** existed with the H2″ atoms (on C2″) of the *n*-Bu groups of *other molecules of* ***8*** in the NOESY spectrum (Figure 9B). The –OH proton cannot form an NOE interaction with the intramolecular H2″ proton(s) (e.g. on same molecule’s -*n*Bu) (Figure 9F) because the distance between these two protons is over 5.6 Å in its lowest energy conformation obtained at the B3LYP/6-311+G(d) level in the gas phase (Figure 9G). This makes the assumption that the gas phase minimum energy conformation is appropriate to invoke in the liquid phase (CDCl_3_) and in the 3-molecule aggregate (Figure 9H) or some other existing aggregate.

**FIGURE 10:**
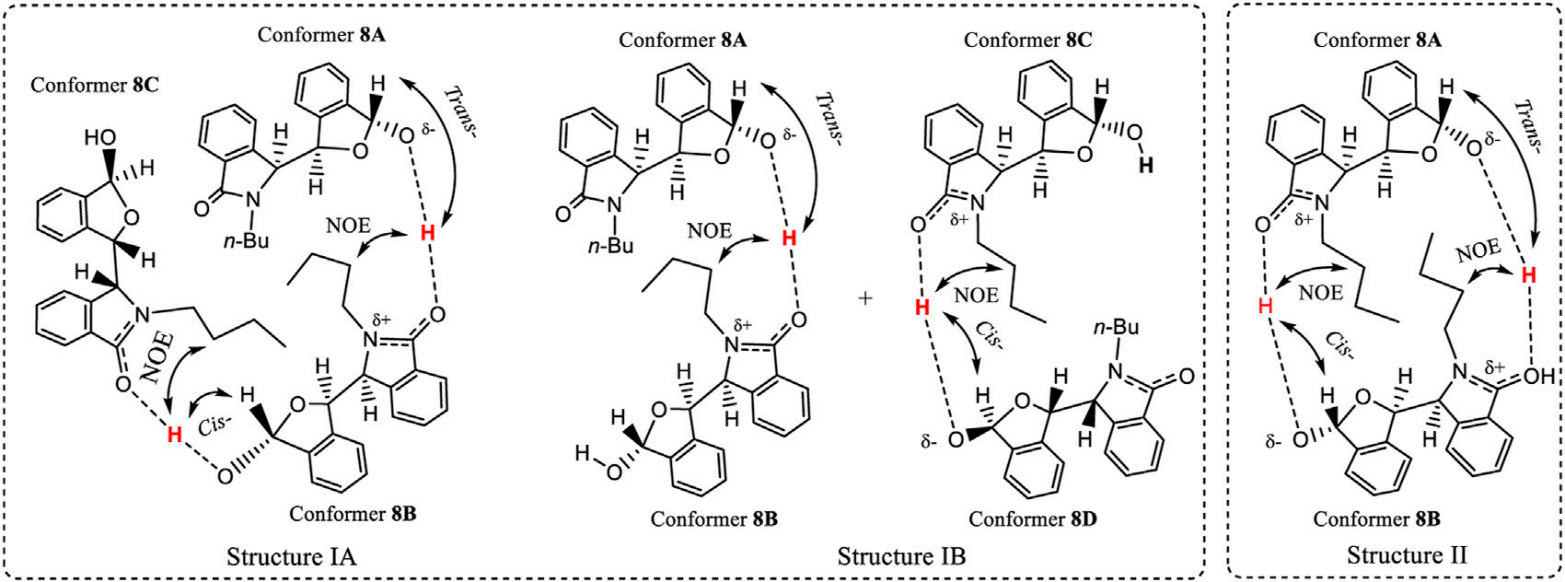
Two hypothetical aggregation state structures of **8** in CDCl_3_ solution, where the O-H may elongate and the proton approaches close to another molecule’s >C=O within the aggregate. After comprehensive consideration, the favourite dimeric structure is IB where conformers **8A** and **8B** are included.

This means that at least there are two additional molecules, for a total of at least three, involved in the same aggregation state as is depicted in Figure 9H. In this case, the ^1^H NMR ratio would be 1:2 instead of 1:1. Thus, this aggregation state in Figure 9H may not exist for **8** in solution. Another aggregation state structure is needed for CDCl_3_ solutions that has low energy. This is visualized next in Figure 10. Two separate signals for carbon C3′ appeared at 101.27 and 101.30 ppm for **8** in CDCl_3_. Each signal must belong to the C3′ atoms in two separate pseudo-resonance structures of **8**. The corresponding H3′ signals of these conformers were observed at 6.18 ppm and at 6.11 ppm (*see* HMQC spectrum in Figure 9C), respectively. The signal at 6.18 ppm was a doublet with a coupling constant of 10.8 Hz. This suggests that H3′ is located *trans-*to the –OH (3.68 ppm) in one enantiomer of **8**. The single 6.11 ppm signal (coupling constant is zero) showed that this H3′ must either be oriented *cis-* or it rotates freely around the O atom in another **8** enantiomer. Finally, only one –OH signal was found at 3.68 ppm in its ^1^H NMR spectrum. This is consistent with all the -O┉H┉O=C< existing connections having the same chemical shift for this –OH proton. On the other hand, the –OH proton was not correlated with any C atom(s) in the HMBC spectrum. For example, the C3′, C4′ or C5′ carbons had no HMBC spectral correlations with the –OH proton (Figure 9D).

A tentative explanation for these results is that this O-H bond may be elongated towards the carbonyl oxygens of other molecules of **8** (Figure 10) within an aggregated structure. This type of H-bond could be strong, assisting other intermolecular interactions to promote a sufficiently favourable aggregation in solution that results in two structurally different conformers. Such an aggregation must be stable enough to produce two separate solution NMR coexisting spectra for **8**. To meet the requirements of these NMR experiments, two hypothetical structures I (A and B) and II are proposed (Figure 10). Aggregation states I and II are illustrated in Figure 10 based on the key NOE observation that –OH proton(s) had the correlations with the other two molecules’ H2″ of -*n*Bu. State I may contain two sub-aggregation states IA and IB. In IA, a linear trimer structure may form where two –OH groups formed a *trans*- and *cis*- orientations to H3′, respectively. In IB, one pair of dimers formed, where one dimer’s –OH formed a *trans*-orientation to H3′, and another formed *cis*-orientation. The structures in 1A or 1B both may produce this NOE. State II involves a cyclic structure, where the two monomers of (*S,R,R*)-**8** are connected *via* a *trans*-orientation and a *cis*-orientation, respectively. Since monomer (*S,R,R*)-**8** had the same two distinct coexisting ^1^H NMR solution spectra as the racemic **8** had in CDCl_3_, and just one single NMR spectrum in CD_3_OD, the experimental results require that the two monomer dimer of (*S,R,R*)-**8** must be the same as the dimer of racemic **8**. Namely, racemic **8** must form the dimeric aggregate of two molecules of (*S,R,R*)-**8** or two molecules of (*R,S,S*)-**8**.

Both **4** and **8** had each produced two separate NMR spectra simultaneous in CDCl_3_. The number of possible conformers of **4** is far less than for **8**. Thus, the number of conformers in a dimer composed of two associated (*S,R,R*)-**4** molecules in structure II (and also for the dimer of (*S,R,R*)-**4** with (*R,S,S*)-**4**) will be less than the number of conformers of any dimer of **8.** Thus, the dimers of **4** were computationally investigated using quantum methods instead of dimer of **8** in structure II. The computations found that dimeric structure II, containing only (*S,R,R*)-**4,** had a 1.32 kcal/mol higher gas phase energy (Supplementary Table S14) than the dimer of (*S,R,R*)-**4** with (*R,S,S*)-**4**. Therefore, the aggregation structure II is ruled out, leaving the aggregation state I as a possibility. The observed mole ratio of the two coexisting NMR spectra of **8** was 1:1 in CDCl_3_, and the *trans* orientation of H3′ to the -OH (3.68 ppm) in one enantiomer of **8**, favoured IB, where conformers **8A** and **8B** formed the dimeric structure.

**FIGURE 11:**
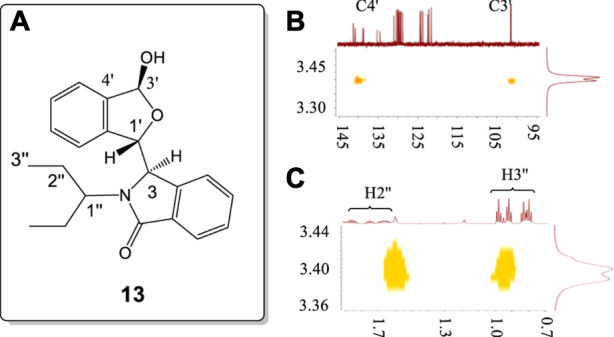
**(A)** Numbering of racemic **13**. **(B)** HMBC correlations of –OH proton with the C3' (102.3 ppm) and C4' (140.4 ppm) signals of **13**. **(C)** NOESY correlations of the −OH proton with the H2″, and H3″ protons of only a single ethyl of the 3-pentyl group in **13**. All units in ppm.

The racemate of **13**, containing a bulky flexible 3-pentyl substituent on the *N* atom, was investigated by 2-D NMR using HMBC, and NOESY (Figure 11, also *see* Supplementary Figures S104–S106). The coupling constant of H3' (6.12 ppm) to the –OH proton was 11.9 Hz. The –OH proton signal at 3.56 ppm had weak correlations with C3′ and C4′ carbons in HMBC (Figure 11A). This confirmed that the 3.56 ppm signal was the –OH proton resonance. This further confirmed that the 3.68 ppm resonance in **8** is also the –OH proton signal (Figure 10). The NOESY study of this –O-H proton only exhibited strong correlations with one ethyl group of 3-pentyl substituent (Figure 11B) rather than both of the ethyl groups. This demonstrated that rotation about the C−N bond of the *N*-CHEt_2_ function is seriously restricted in solution.

**FIGURE 12:**
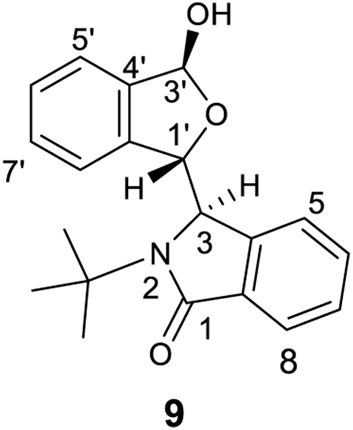
The structure of **9**.

Finally, HMQC, HMBC and ROESY experiments were performed on racemic **9** (Figure 12, also *see* Supplementary Figures S107–S109), which has a bulky and rigid *N*-*t-*butyl substituent. The doublet resonance for H3′ (located at C3 containing the –OH group) at 6.03 ppm had a coupling constant of 12.5 Hz. Its hydroxyl proton (3.02 ppm) had no correlations with carbons C3′, C4′ or others. This O-H proton’s ROESY signal had correlations with one Me of the -*t-*Bu group. Thus, racemic **9** in CDCl_3_ formed an aggregated structure similar to **8** in the BLC pair of **9**.

Indeed, the hydroxyl proton ^1^H NMR signals in CDCl_3_ of **4**, **6–14** were all readily observed. Their chemical shifts are: **4** (4.13 ppm, Supplementary Figure S9), **6** (4.57 ppm, Supplementary Figure S21), **7** (2.38 ppm, Supplementary Figure S25), **8** (3.68 ppm, Supplementary Figure S29), **9** (3.02 ppm, Supplementary Figure S33), **10** (3.50 ppm, Supplementary Figure S37), **11** (3.89 ppm, Supplementary Figure S41, broad peak), **12** (3.70 ppm, Supplementary Figure S45), **13** (3.56 ppm, Supplementary Figure S49) and **14** (3.37 ppm, Supplementary Figure S53). Therefore, **4**, **6**–**14** all had similar aggregation structures in CDCl_3_ casuing pseudo-resonance structures.

**FIGURE 13:**
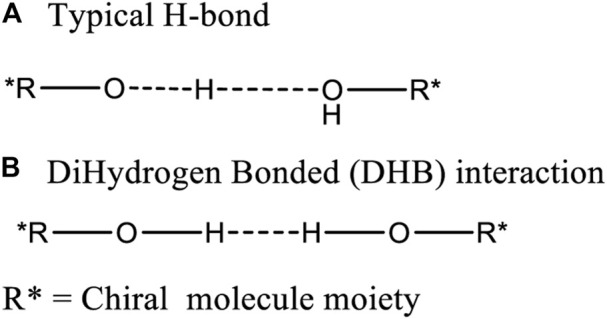
Two possible intermolecular interaction modes (**A**: typical H-bond and **B**:dihydrogen bond) between the hydroxyl groups on two chiral alcohols.

Chiral alcohols **15** to **26**, **19** and **20** each have two –OH function groups but no >C=O group. Neither **19** nor **20** gave two different coexisting NMR spectra in CDCl_3_. However, both **19** and **20** selectively formed alternating bond length changes with different length for corresponding bonds in the solid state. Thus, another interaction may occur between the molecules to form an aggregation which generates solid state pseudo-resonance structures. One O-H bond might elongate towards another hydroxyl oxygen to produce a strong intermolecular H-bonded interaction (Figure 13A). Another postulate with literature precedent are DHB structures, which contain a strong two proton interaction (Damodharana and Pattabhi, 2004; Grabowski, et al., 2005) (Figure 13B).

Neutron diffraction was explored as a means to determine the location of the two hydroxyl protons in a crystal of **15** (Supplementary Table S7) with the hope of determining the distance between them as evidence for the existence of a structure like (–O^δ−^┉H┉HO^δ+^–) or (–OH┉HO–). Such interactions might have played a role in causing the observed bond length differences. Unfortunately, the weak neutron diffraction detected failed to measure this distance (Supplementary Table S15). Furthermore, the single point energies (SPE) of the **15A** and **15B** molecular pair interactions in the gas phase, [E_(**15A**+**15B**)_] was compared to the sum of the SPEs of the individual monomers **15A** [E_**15A**_] and **15B** [E_**15B**_]. These were computed at the B3LYP/6-311++G(2d,p) level (Note, SPE) using their corresponding crystal coordinates. The sum of the individual SPE (E_**15A**_ + E_**15B**_) of a single **15A** and single **15B** was 12.1 kcal/mol higher than the energy of both **15A** and **15B** (E_(**15A**+**15B**)_)**.** This SPE difference is within the predicted energy range (11–16) kcal/mol found for a dihydrogen bonded (DHB) structures with an approximate 1.23 Å H┉H distance (Wolstenholme and Cameron, 2006). Unfortunately, a trial to optimize both **15A** and **15B** together in the gas phase failed to simulate the pseudo-resonance structures using the crystal coordinates as the initial input coordinates using the B3LYP/6-311++G(2d,p) level of the theory in optimizations.

**FIGURE 14:**
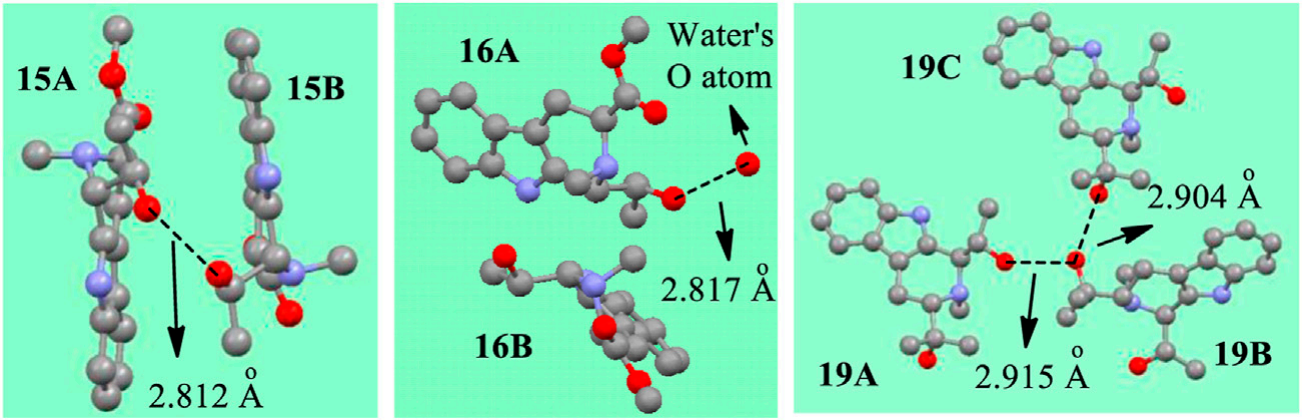
The distances between the O atoms in X-ray structures of **15**, **16** and **19** (The red balls represent O atoms, the grey represents C, the light blue means *N*. H atoms are omitted for clarity).

Additional evidence concerning forming either a (–O^δ−^H_2_O^δ+^–) or a (-OH┉HO-) interaction structures in the solid state is illustrated by examining the X-ray crystal structures in Figure 14. The distance between the two hydroxyl O atoms of **15A** and **15B** was 2.812 Å in the crystal structure (Supplementary Figure S68). This is shorter by 0.005 Å than 2.817 Å distance from the hydroxyl O atom in **16A** to a water’s O atom. This short oxygen/oxygen distance in **15** may mean that the H┉H distance is short enough to a big elongation of O-H bond as theoretically predicted previously (Grabowski, et al., 2005). This bond may produce a bigger elastic force when its bond length was compressed. This hypothetical larger elastic force might lead to larger bond length elongations or shortenings in **15A** and **15B**. The recorded bond length elongations or shortenings observed between **15A** and **15B** are in fact larger than those in **16** (Tables 2 and 3). In addition, compound **19** formed a three-molecule solid state aggregate where three molecules of **19** and one molecule of water were involved. In the crystal structure of this aggregate of **19**, three protons interacting together (Figure 14, also *see* Supplementary Figure S75).

**FIGURE 15:**
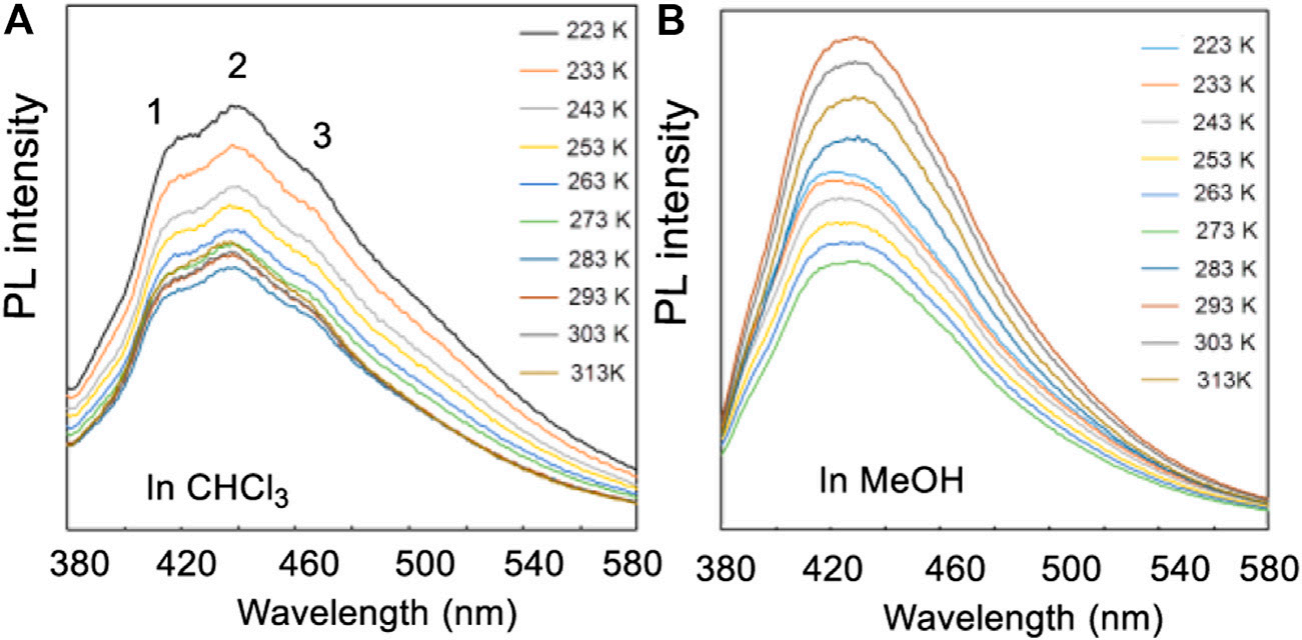
**(A)** The PL spectra of compound **4** in the mixture of CHCl_3_/MeOH (v/v, 5:1), **(B)** PL spectra in methanol. Temperatures varied from 223 to 313 K.

Scanning electron microscope (SEM) studies of **4** and **15** did not exhibit any interesting SEM structures initially (Supplementary Figure S110). However, photo-luminesce (PL) experiments exhibited three obvious signals (1–3) in a CHCl_3_ solution of **4** (containing nearby 20% of MeOH to increase its solubility) but only one in pure MeOH (Figure 15B) at temperatures from −50 to 40°C (223–313 K) (Supplementary Tables S16, S17). If two different pseudo-resonance structures of **4** existed in the chloroform but one in methanol as was proposed above, it is easy to understand that three signals were recorded in chloroform but only one in pure methanol. These properties may be considered for further uses of new functional materials.

**FIGURE 16:**
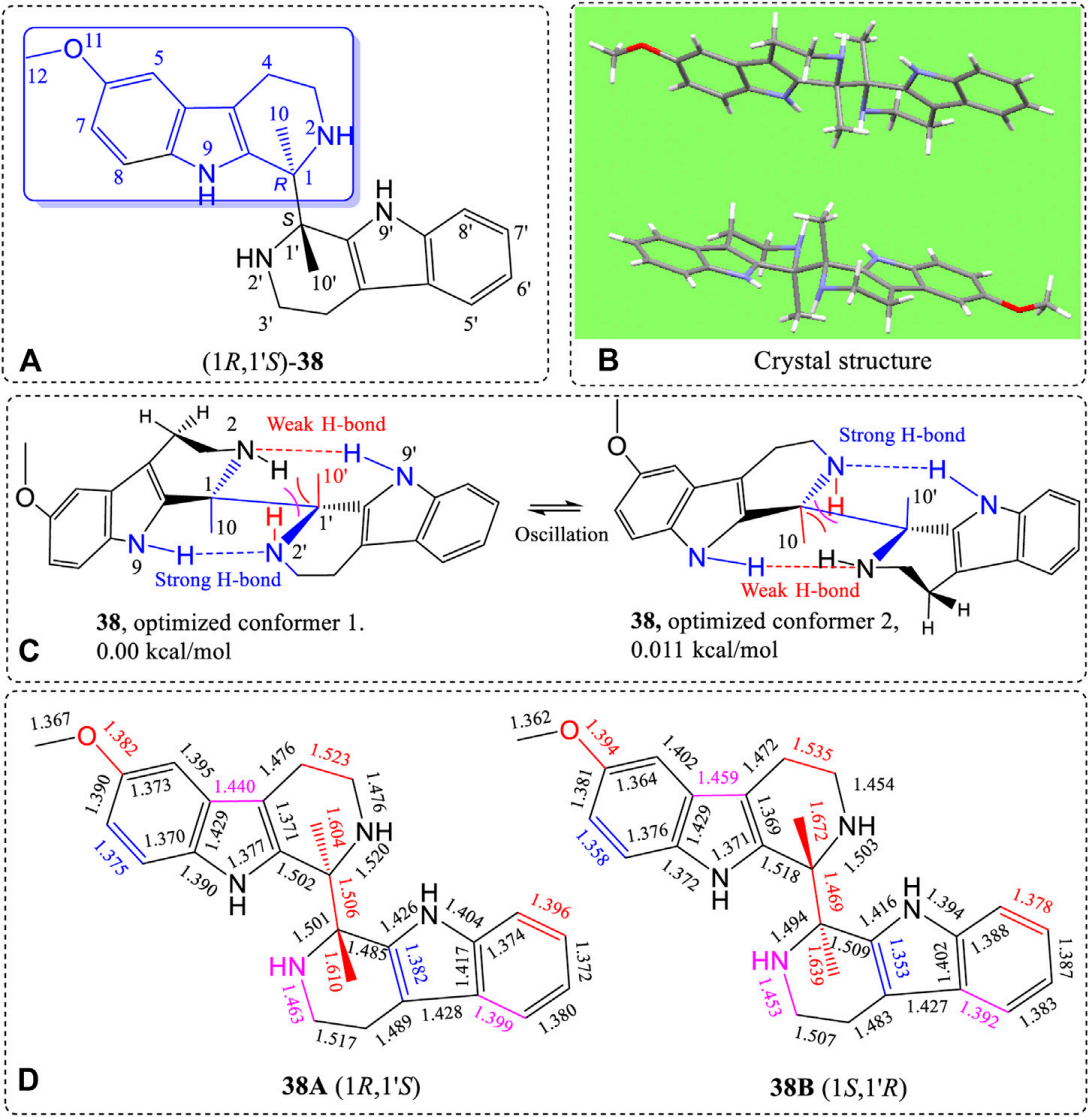
**(A)** Structure of **38**, **(B)** X-ray structure, **(C)** B3LYP/6-311+G(d)-optimized geometries with the lowest and second lowest energy structures shown, and **(D)** differences of bond lengths of the corresponding bonds of the (1*R*,1′*S*)-**38A** and (1*S*,1′*R*)-**38B** enantiomer from the X-ray structure of the crystalline racemate.

Finally, racemic **38** (Figure 16A, also *see* Supplementary Figures S111–S114) was synthesized by our Pictet-Spengler method (Cox and Cook, 1995), and its structure was determined by X-ray (Figure 16B, also *see* Supplementary Tables S18, S19). Compound **38** does not contain hydroxyl or carbonyl groups. However, **38** has four >NH groups including two similar secondary cyclic piperidine amines (N2 and N2′) and two pyrole nitrogens (N9 and N9′, with nitrogen lone pairs conjugated with the ring’s carbon-carbon double bonds). The lone pair electrons of N2 can closely approach H9′ to form a H-bond. Similarly, N2′ also forms a H-bond to H9. The lone pair delocalization of N9 and N9′ favor H-bond formation between N2 vs. H9′ and N2′ vs. H9. These two H-bonds formed simultaneously, as indicated by conformational searches. The two most stable conformations, 1 and 2 (Figure 16C), were determined from B3LYP/6-311+G(d)-optimized geometries. The dihedral angle around C10-C1-C1′-C10′ was 171.6° in the optimized structure. During the conformational equilibrium between 1 and 2 caused by N2 and N2′ geometry oscillations predicted by calculations (Figure 16C), the H-bond lengths (N2-H9′ and N2′-H9) must change, causing other periodic bond length changes at room temperature. The specific solid state bond lengths determined by X-ray are illustrated in Figure 16D. The driving force for the bond length differences in these pseudo-resonance structures originated from the C1-C1′ rotational angle. This angle influences the C1-C1′ length, the existence of the H-bonded moieties and the strong repulsions caused by the very short distance between H2 and C10 in space (Figure 16C). Therefore, the maximum bond length changes occurred in this area of the molecule for the pseudo-resonance structures of **38**. For example, the C1-C10 bond had the largest lengths (1.672 Å) in (1*S*,1′*R*)-**38B** (Figure 16D). This same bond in (1*R*,1′*S*)-**38A** had a length of only 1.604 Å. This bond length difference of 0.068 Å is very high.

**FIGURE 17:**
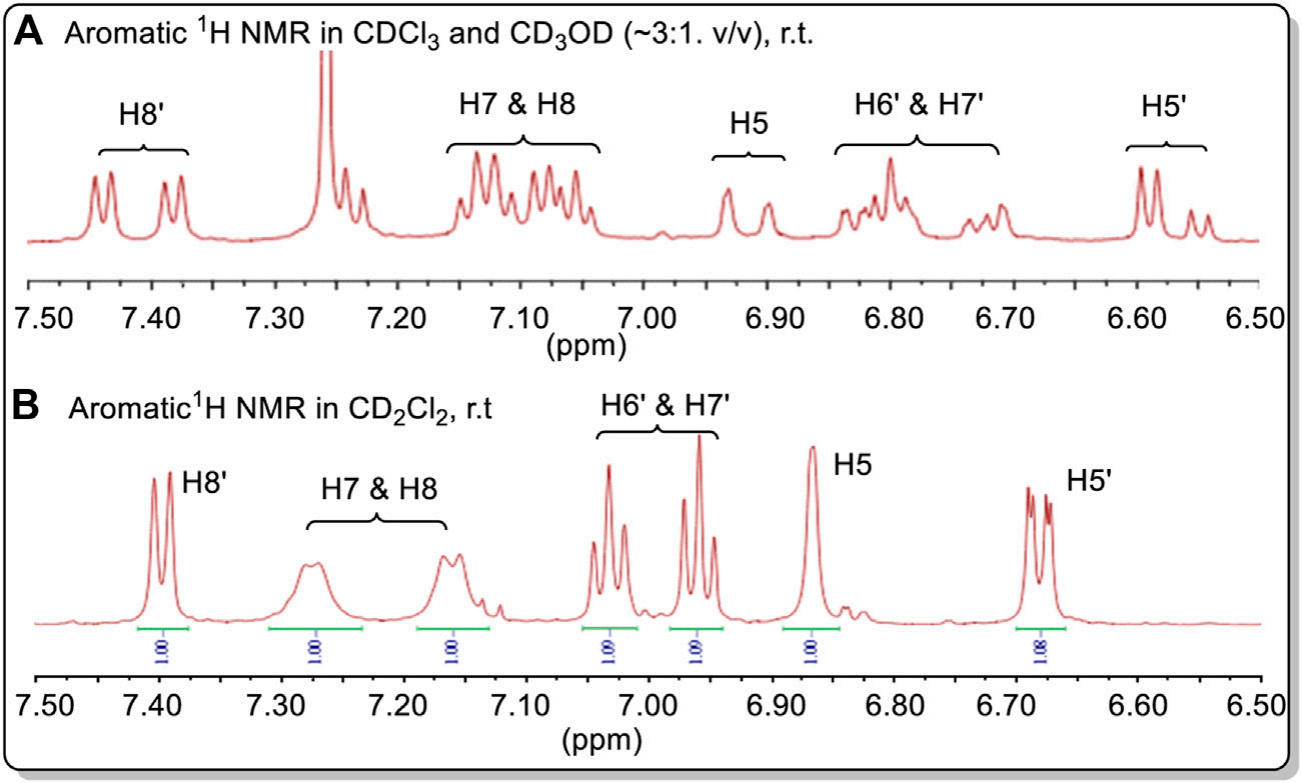
**(A)** Aromatic ^1^H NMR spectra of **38** in CDCl_3_ + CD_3_OD. **(B)** The aromatic ^1^H NMR spectra of **38** in CD_2_Cl_2_. Compound **38** had a relatively low solubility in CDCl_3_. Minor amounts CD_3_OD can significantly increase its solubility.

The ^1^H NMR spectrum of **38** determined in a CDCl_3_ and CD_3_OD (∼3:1, v/v) solution at 298 K gave two distinctly different ^1^H NMR spectra, simultaneously, in a nearly 1:1 ratio proving the existence of pseudo-resonance structures in this solution (Figure 17A, also *see* Supplementary Figures S112–S114). On changing solvent to CD_2_Cl_2_, the spectrum initially appeared to have a single structure, with one set of ^1^H NMR signals at 298 K. However, the two doublets appearing at 6.67 ppm could indicate that two different and distinct NMR spectra still existed in CD_2_Cl_2_. This possibility has not yet been proven. The broad singlet at 6.87 ppm (H5), doublets at 7.17 and 7.27 ppm (H7 and H8) in CD_2_Cl_2_ may be the corresponding overlapped signals. These signals belong to the aromatic protons located on the methoxy-containing carboline moiety (in blue line square in Figure 16).

Gussian03 was used in an attempt to simulate the two conformers **15A** and **15B** using the keyword “opt” at the level of B3LYP/6-311++G(2d,p) level or other basis sets. However, all trials failed. The same bond length and angle in the two B3LYP/6-311++G(2d,p)-optimized conformers were almost the same. Other levels of theory like MP2 and different basis set failed to simulate the two different geometries **15A** and **15B** under optimization study. However, the different bond lengths of structure **2** can be predicted in different geometries. We did not try other software in the simulations. More computational methods may be required to simulate the pseudo-resonance structures in the future.

## Conclusion

Twenty-three chiral alcohols were synthesized. Ten of them (**4**, **6**–**14**) had CDCl_3_ solutions where two distinct NMR spectra of the compound coexisted. Additionally, four compounds (**15**, **16**, **19** and **20**) exhibited two distinct ^13^C CP-MAS NMR spectra coexisted for those compounds in the solid state, where alternating bond length changes were verified by X-ray structures and differences in these corresponding bond lengths between the two pseudo-resonance structures existed. These coexisting conformers with alternating and different bond lengths for corresponding conformer bonds suggest they aggregated into dimer or trimer structures of pseudo-resonance structures in solution. During incorporation into the crystalline solid such conformers may have been preserved, modified or formed as a result of solid state assembly. Eleven additional chiral alcohols (**27**–**37**) were discovered in the literature which had two distinct solid state coexisting conformers with alternating corresponding bond lengths. They are expected to also exhibit different ^13^C CP-MAS NMR spectra. A new racemate with four *N*-H functions but no hydroxyl group, **38**, was synthesized and found to form pseudo-resonance (or BLC) structures in both in liquid (CH_2_Cl_2_) and solid state. Compound **38** exhibited two different coexisting of ^1^H NMR spectra in coexisting both liquid (CH_2_Cl_2_) and solid states.

The existence of different solution NMR spectra coexisting in the same solution (**4**, **6**–**14**, **38**) and the 2D-NMR spectra described here, argue for the existence of such “pseudo-resonance structures (or BLC)” in solution. We have proposed these results from aggregation effects that have been rationalized the 1-D and 2-D NMR solution experiments and IR observations. This rational also draws upon the X-ray crystal structure data, illustrating the different and alternating bond length and bond angle data and IR adsorptions. These current explanations using hypothetical aggregates are admittedly quite speculative. Obviously, more study is required in the future. However, these unique observations of the same compound exhibiting different NMR spectra at the same time in both solution and solid state are established facts. The concept coexisting of “pseudo-resonance structures (or BLCs)” is novel. These should draw extensive further study and discussion.

## Data Availability

The raw data supporting the conclusions of this article will be made available by the authors, without undue reservation.
