# Supplementary material for: Pseudo-resonance structures in chiral alcohols and amines and their possible aggregation states
Source: Front Chem. 2022 Aug 29;10:964615. doi: 10.3389/fchem.2022.964615 (PMC9465258; doi:10.3389/fchem.2022.964615)
Supplement: Supplementary file 1 [file DataSheet1.PDF]

Supplementary Material for

Chiral Alcohol Bond Length Conformers and Possible Aggregation States

Huajie Zhu, [1,9†] Shengnan Li,[1,8] Yunjing Jia, [8] Juxing Jiang, [5] Feiliu Hu, [8] Longfei Li, [8] Xiaoke Wang,[8] Shenhui Li,[2] Guanghui Ouyang, [3] Gengfang Tian, [4] Ke Gong, [6] Guangjin Hou,[6] Wei He,[7] Zheng Zhao,[7] Charles U. Pittman, Jr.[8] Feng Deng,[2†] Minghua Liu, [3†] Kai Sun, [5†] Ben zhong Tang [7†]

Content

Entry		Pages
1	CCSD ID of crystals (**15**, **16**, **17**, **19, 38**) X-ray	S2
2	Identification of raceme of compound **4** (Figures S1-S15，NMR, X-ray, HPLC, calculations etc.)	S3
3	NMR spectra for the oxidized product **5** (Figures S16-S18)	S67
4	NMR data of racemates **6-14** in CD_3OD and $CDCl_3$ (Figures S19-S54)	S70
5	NMR and other data of compounds **15, 16** and **17** in liquid and solid (Figures S55-S67)	S106
6	Analysis of crystal structures of **15, 16** and **19** (Figures S68-S69)	S120
7	Physical data for compounds **18** to **20** (Figures S70-S81)	S122
8	Physical data for compounds **21-26** (Figures S82-S99)	S135
9	Bong length changes for compounds **30** to **37** (Figure S100)	S155
10	2D NMR for compounds **8, 13** and **9** (Figures S101-S109, HMBC, HSQC and NOSEY)	S157
11	SEM pictures for **4** and **15** (Figure S110)	S205
12	X-ray structure and ^1H and ^{13}C NMR for compound **38** (Figures S111-S114)	S210
13	Crystal data for raceme **4** (Tables S1-S2)	S214
14	Transition state calculations for **4A** and **4B**, and energy computation for epi-**4** (Tables S3-S4, energy barriers and coordinates)	S218
15	Computed rotation barriers around C3-C1’ bond for **5** (Table S5)	S230
16	Computed rotation barriers around C3-C1’ bond for **8** (Table S6)	S231
17	Crystal data for crystal**s 15-17** and **19** and computed bond length for **15-17** (Tables S7-S13)	S232
18	Dimers energy of **4** (Table S14) and their coordinates	S245
19	Neutron diffraction experimental data (Table S15)	S250
20	The PL spectra data of compound **4** in solvents (Tables S16-S17)	S437
21	The crystal data for compound **38** (Tables S18-S19)	S449

ID of crystals (**15**,**16**,**17**,**19**) X-ray CCSD.

The CCSD numbers deposited early at the Cambridge Crystallographic Data Centre with the deposition numbers are listed here.

Crystal **15**, CCDC 687115
Crystal **16**. CCDC 687116
Crystal **17**, CCDC 687117
Crystal **19**, CCDC 696914.
Crystal **38**, CCDC 2119349

Identification of raceme of compound **4**

HRMS, calcd for $C_{19}H_{20}NO_3$ [M+H$^+$]: 310.1443, found [M+H$^+$]: 310.1450. Due to its poor solubility in $CDCl_3$, minor CD_3OD was added, the ratio of $CDCl_3$:CD_3OD was about 5~6 : 1 (v/v). ^{1}H NMR (600 MHz, $CDCl_3$) δ 7.82 (d, *J* = 7.3 Hz, 0.5 H), 7.79 (d, *J*= 7.2 Hz, 1H), 7.50 (m, 1H), 7.43 (m, 3H), 7.36 – 7.31 (m, 1H), 7.28 – 7.22 (m, 1.5 H), 7.20 (m, 0.5 H), 6.79 (d, *J* = 6.8 Hz, 1H), 6.46 (d, *J* = 7.0 Hz, 1H), 6.19 (s, 0.45 H), 6.09 (s, 1.5 H), 5.94 (s, 1H), 5.85 (s, 0.5 H), 4.99 (s, 1H), 4.94 (s, 0.5 H) 4.40 (m, 0.5 H), 4.26 (m, 1H), 2.61 (s, 3H), 1.58 (m, 3H), 1.37 (d, *J* = 6.7 Hz, 3 H) 1.32(d, *J*= 6.8 Hz, 3 H).

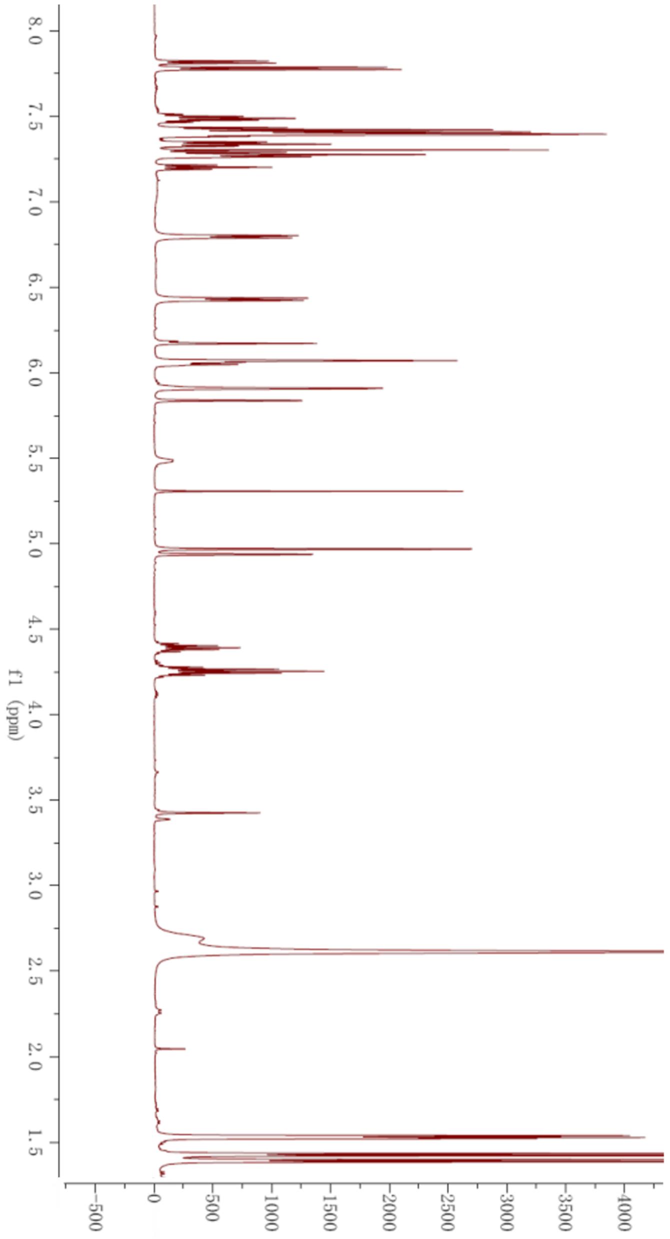

Figure S1. The ^{1}H NMR for the raceme **4** in $CDCl_3$ + CD_3OD (5~6:1,v/v). The signal strength ratio is almost 1.0:0.50 between the two sets of ^{1}H NMR.

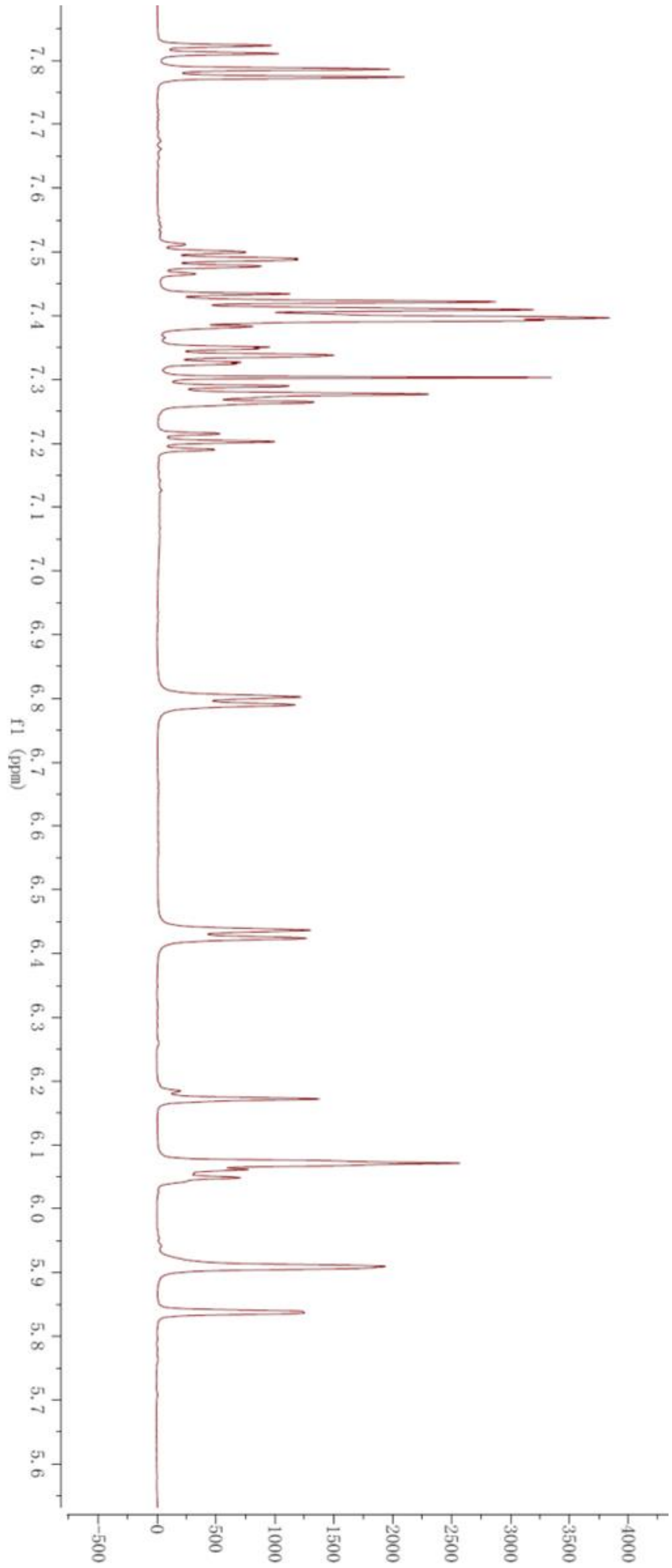

Figure S1. Continued. Partial amplified ^{1}H NMR in Figure 1.

^{13}C NMR (151 MHz, $CDCl_3$ +CD_3OD(minor)) δ 169.26, 168.83, 141.09, 140.61, 140.31, 138.29, 138.11, 134.82, 134.32, 130.93, 130.47, 129.70, 129.45, 129.14, 128.96, 128.66, 123.83, 123.67, 123.41, 123.24, 122.36, 122.28, 121.25, 121.22, 101.11, 100.96, 82.33, 81.38, 77.37, 77.16, 76.95, 64.24, 63.95, 46.55, 46.03, 29.71, 20.87, 20.51, 20.10, 19.93.

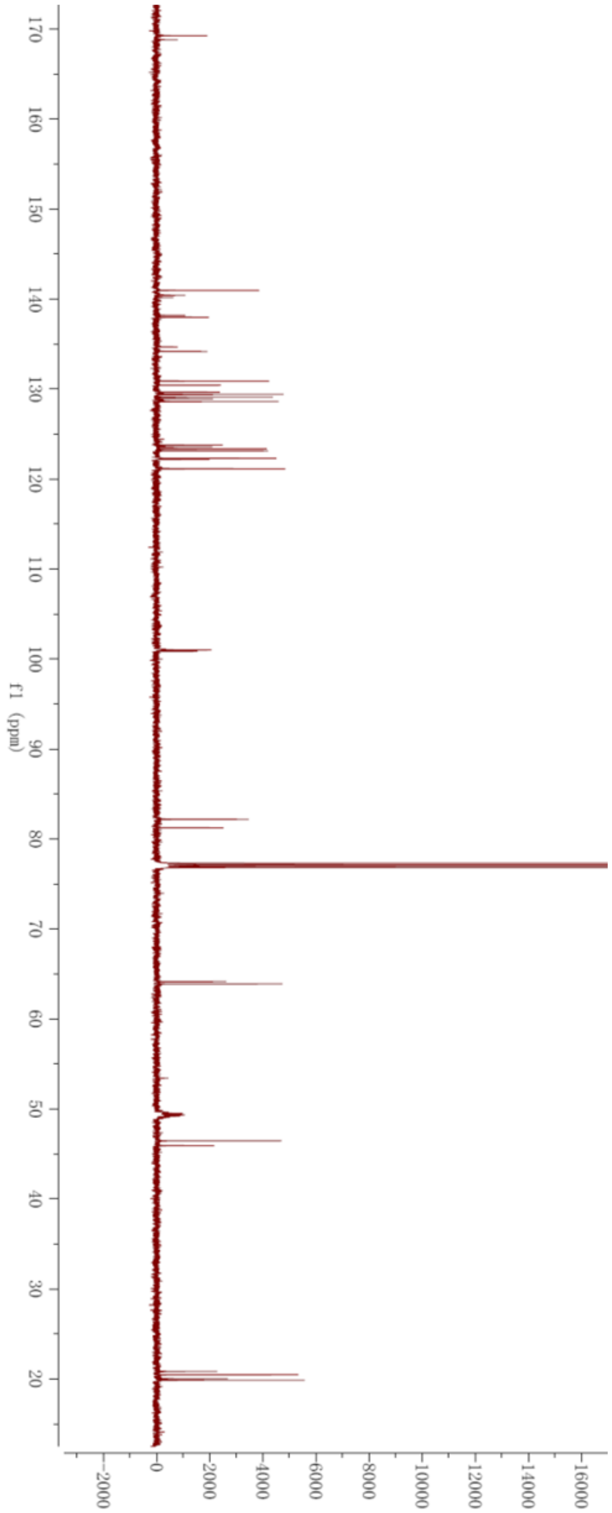

Figure S2. ^{13}C NMR for pure raceme **4** in $CDCl_3$ + CD_3OD (5~6:1,v/v).

HPLC plot using Chiracel ID column using the mixture of petroleum ether **:** ethanol = 9:1. Peaks 1 and 4 have almost the same areas percentages (47.1% vs 47.5%). Signals 2 and 3 may be another pair of enantiomers that were not obtained due to very low yields. The high quality peak of 4 in next HPLC analysis (below) was recorded. No any other signals like 2 and 3 were found in the HPLC plot. This may hint it is pure one compound. It is relatively stable during the experimental period. It looks not like mixture of epimers as shown below:

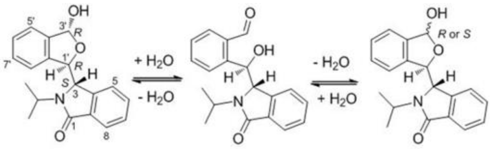

1 intermediate **1** and its epimer

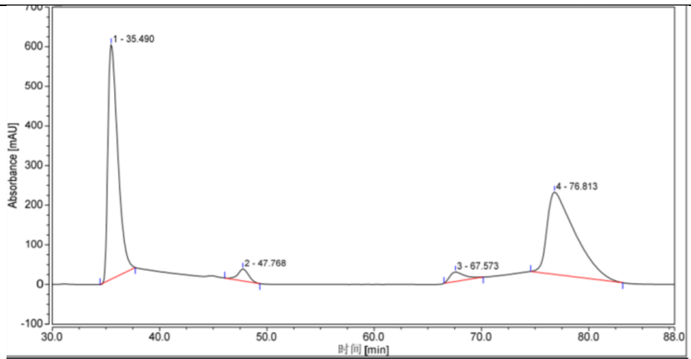

积分结果

序号	峰名称	保留时间 min	峰面积 mAU*min	峰高 mAU	相对峰面积 %	相对峰高 %	样品量 n.a.
1		35.490	630.683	591.808	47.11	69.40	n.a.
2		47.768	35.308	29.937	2.64	3.51	n.a.
3		67.573	37.398	23.735	2.79	2.78	n.a.
4		76.813	635.439	207.325	47.46	24.31	n.a.
**总和:**			**1338.828**	**852.805**	**100.00**	**100.00**	

The optical rotation values ([α]$_D$) for peak (signal) 1: -68.8 (*c*, 5.55), and peak 4: +70.3 (*c*, 7.4) in CH_2Cl_2.
It hints that signals 1 and 4 may be enantiomers.

Each epimer (peak 2 or 3) was about 3% among the four epimers..

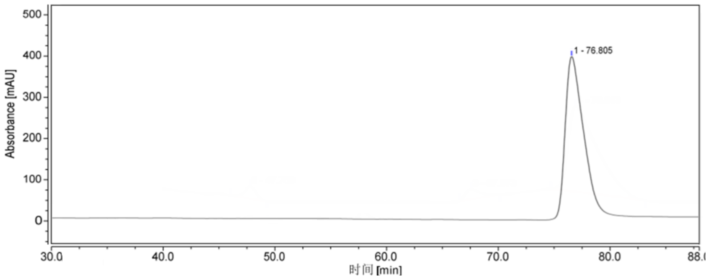

The obtained separated enantiomer from peak 4 (see above HPLC plot) was analyzed after it dissolved in methanol. Only one major HPLC signal was obtained.
This indicates no isomerization happened during its dissolved in methanol.
The same results was obtained when it dissolved in $CHCl_3$ and re-analyzed.

Figure S3. HPLC analysis plot for raceme compound **4**.

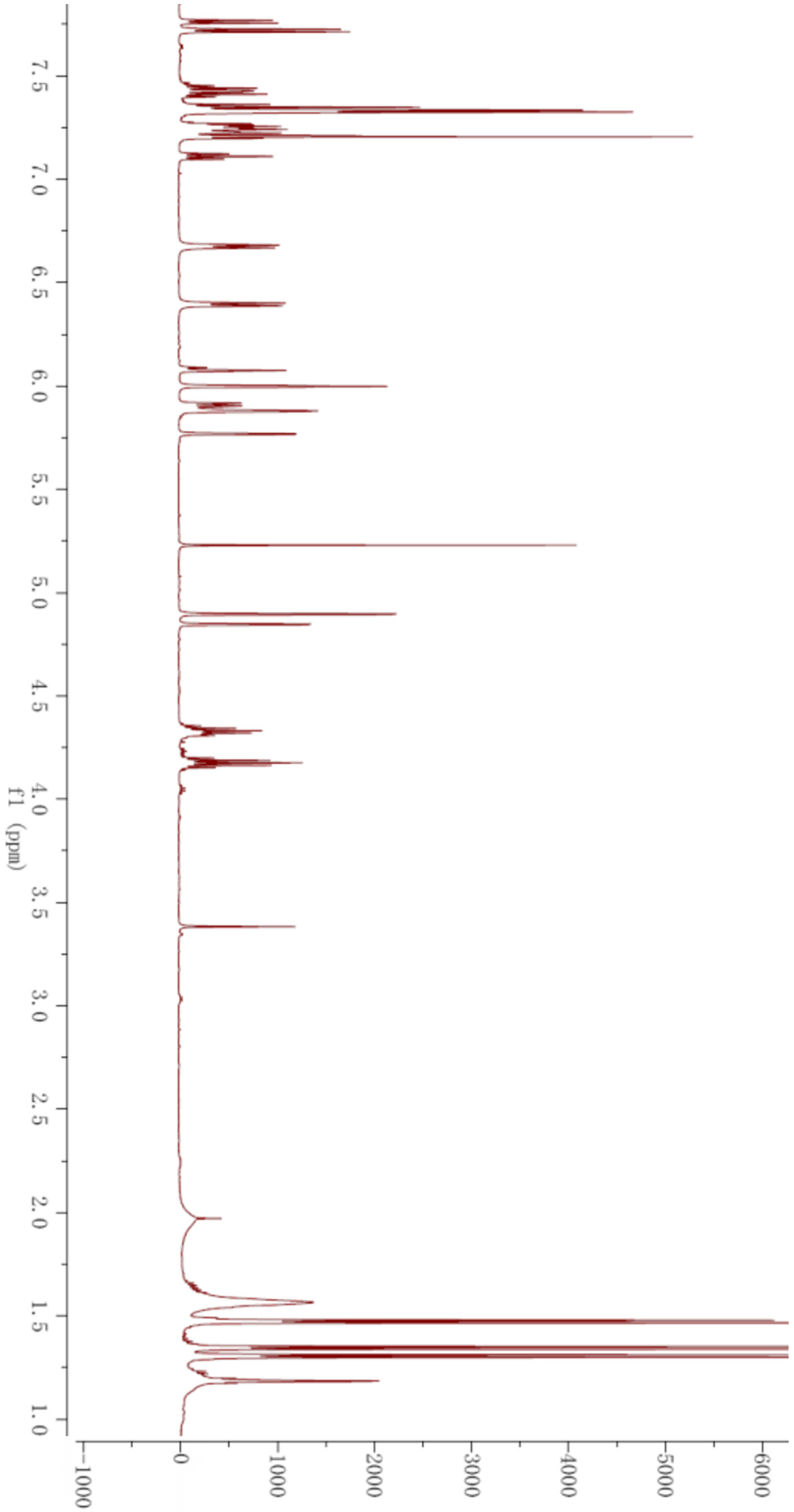

Figure S4. The ^{1}H NMR for single enantiomer (*R,S,S*)-**4** (or (*S,R,R*)-**4**) in $CDCl_3$ + CD_3OD (5~6:1,v/v)

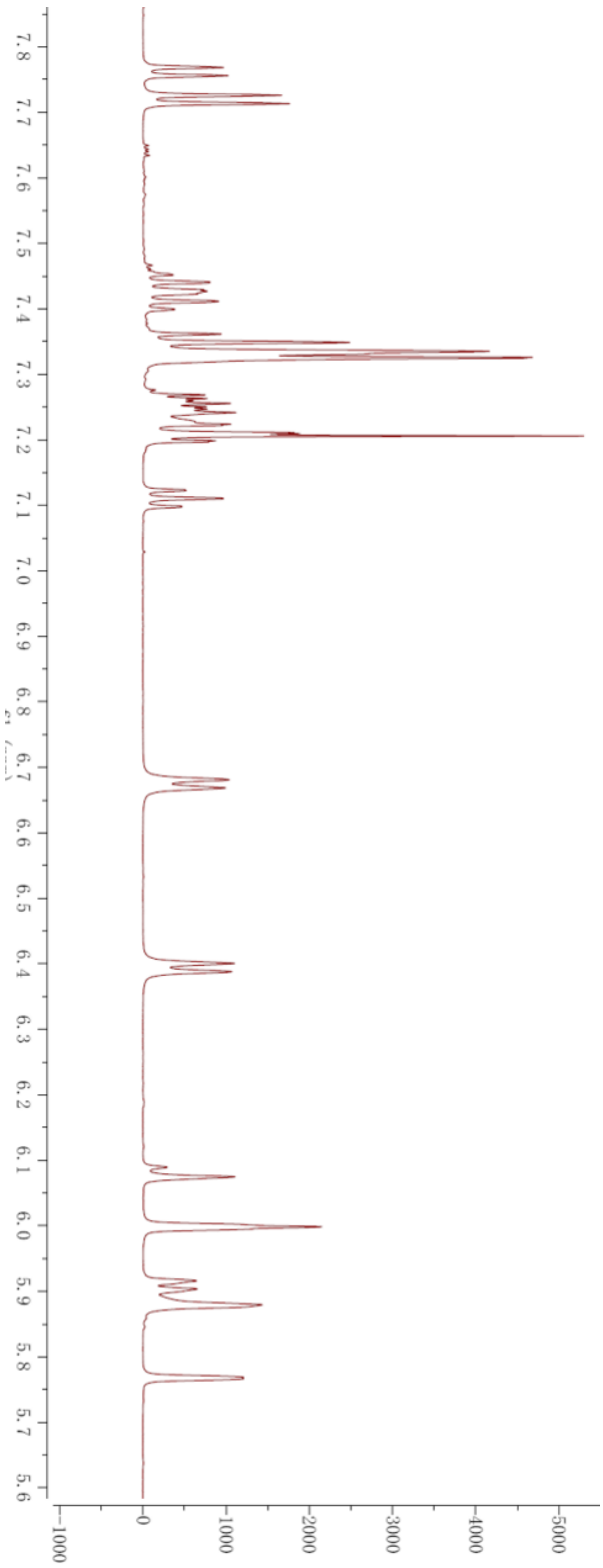

Figure S4. Continued. Partial amplified ^{1}H NMR for **4**.

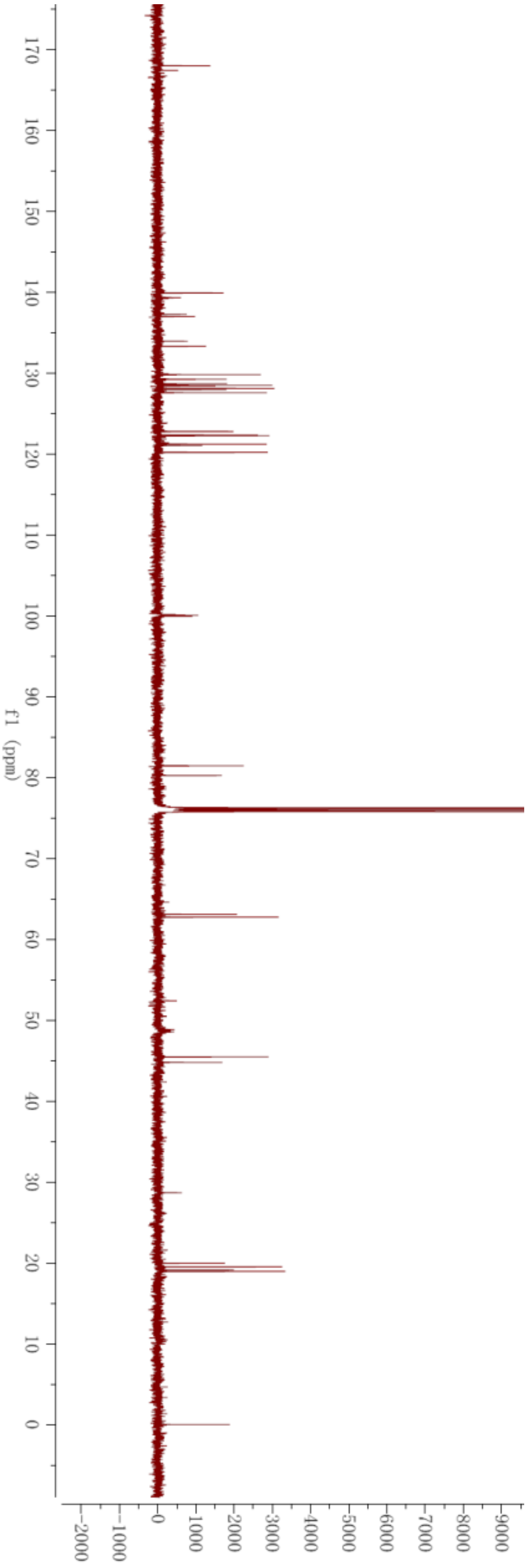

Figure S5. ^{13}C NMR for single enantiomer of (*R,S,S*)-**4** (or (*S,R,R*)-**4**) in $CDCl_3$ + CD_3OD (5~6:1, v/v).

X-ray study

The mixture could form crystal. The X-ray experiments exhibited there are four geometries included in a cell. See the following Figure ure.

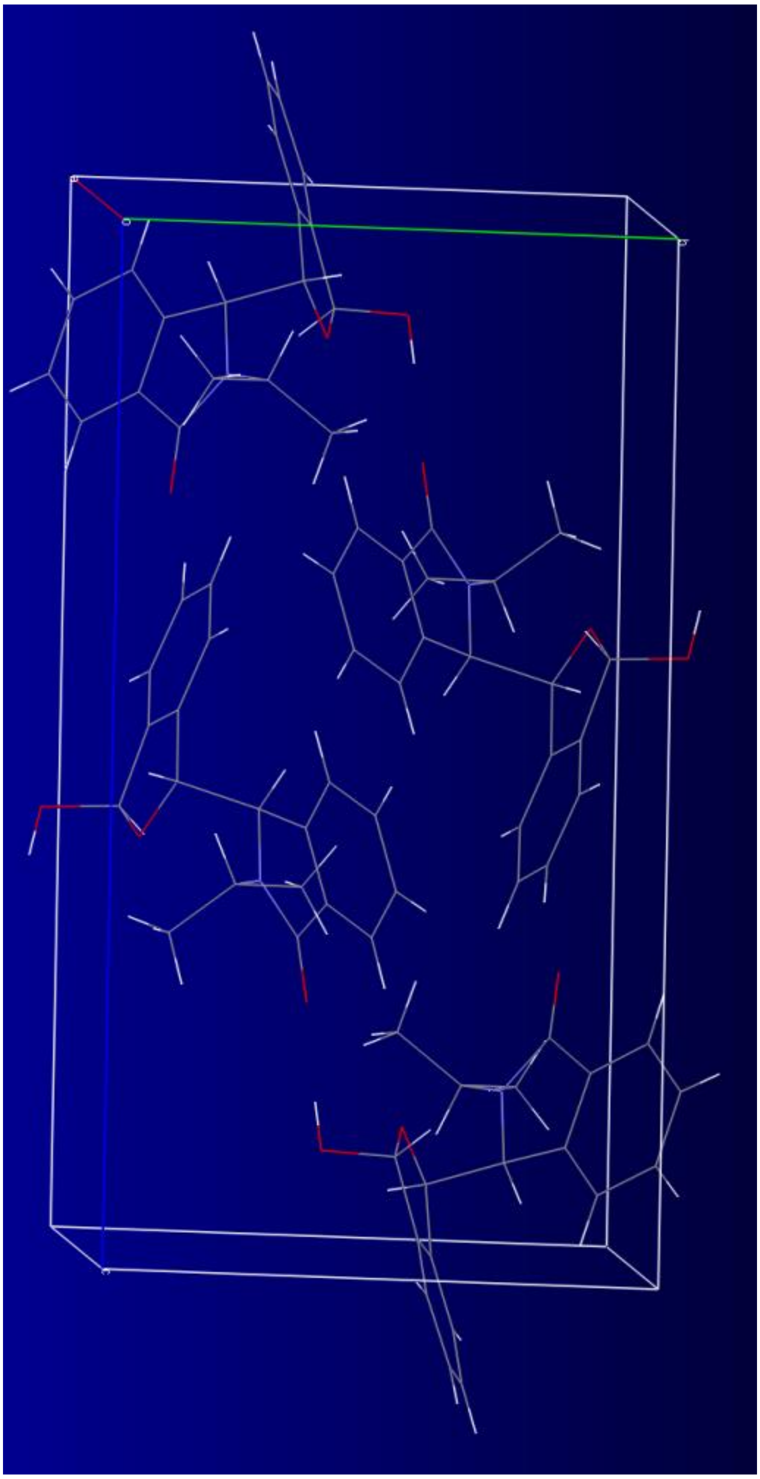

Figure S6. The X-ray experimental results. Every two (*R,S,S*)-**4** and two (*S,R,R*)-**4** molecules neighbored via H-bonds. The two pairs of **4** are closed each other.

^{1}H NMR of the raceme 4 determined in CD_3OD

Only one set of NMR data. Pure crystal was dissolved in CD_3OD for ^{1}H NMR determination. There are some weak signals were recorded. It was near 5% as mentioned before. ^{1}H NMR (600 MHz, $CDCl_3$) δ 7.72 (d, J = 7.4 Hz, 1H), 7.46-7.34 (m, 4H), 7.28 (t, J = 7.5 Hz, 1H), 6.96 (d, J = 6.8 Hz, 1H), 6.28 (d, J = 7.3 Hz, 1H), 6.06 (s, 1H), 5.78 (s, 1H), 5.10 (s, 1H), 4.27 (m, 1H), 3.34 (s, 1H), 1.46 (t, J = 6.7 Hz, 6H).

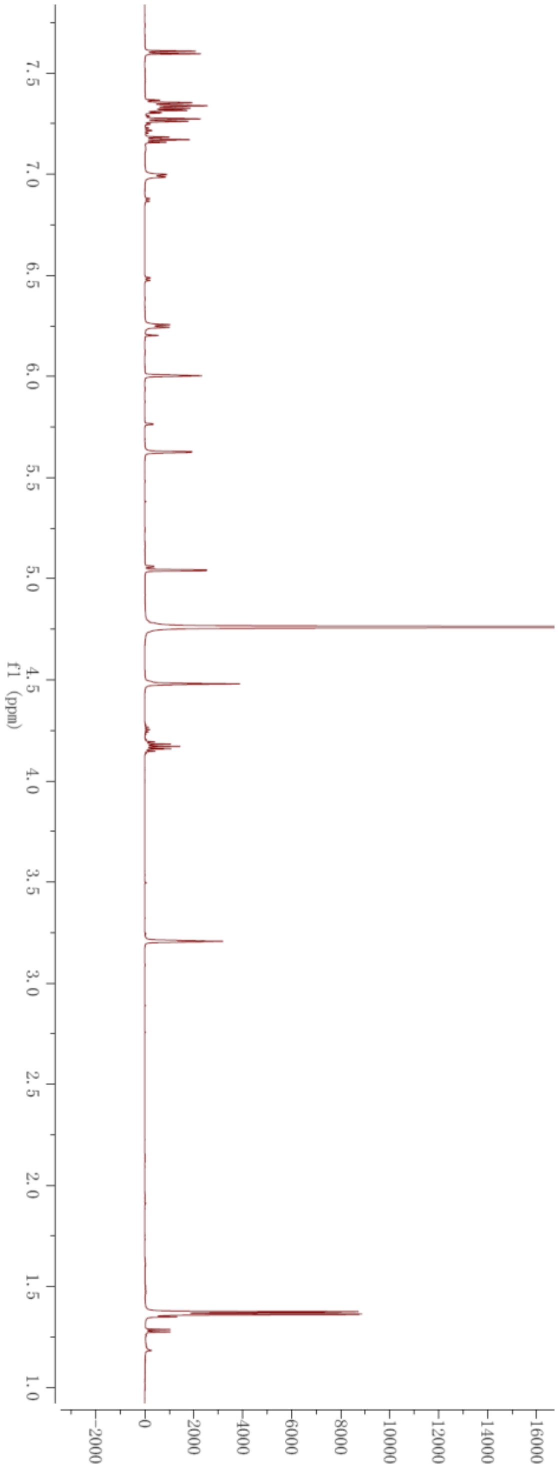

Figure S7. ^{1}H NMR of the racemic **4** recorded in CD_3OD.

^{13}C NMR (151 MHz, CD_3OD) δ 170.70, 142.49, 141.82, 139.35, 135.05, 131.96, 131.88, 130.16, 129.90,129.42, 124.12, 123.54, 122.33, 102.02, 82.72, 79.02, 78.80, 78.58, 65.41, 47.71, 30.46, 20.66, 20.19.

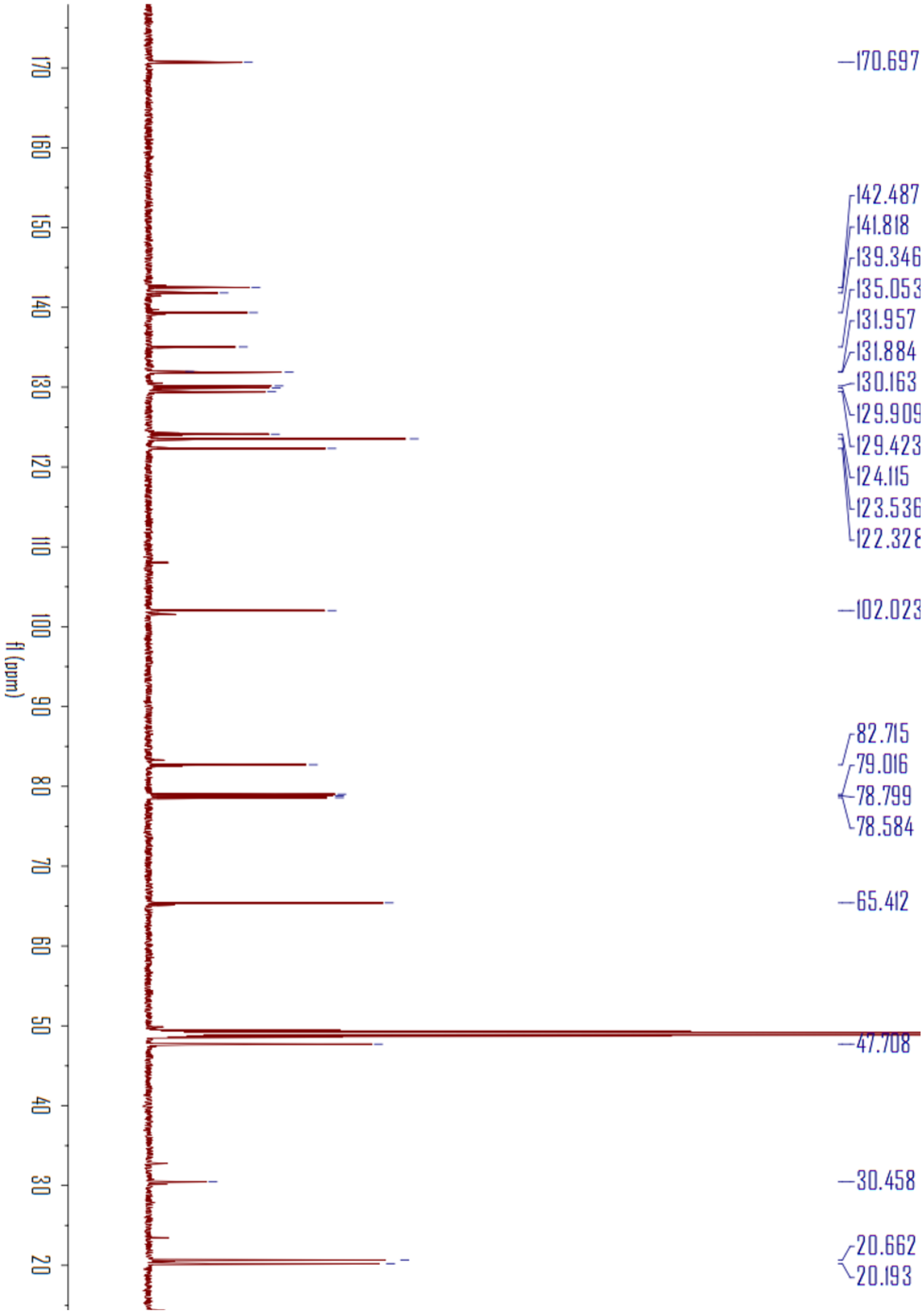

Figure S8. ^{13}C NMR for racemic **4** in CD_3OD.

The ^{1}H NMR for the single enantiomer of (*R,S,S*)-4 in pure $CDCl_3$.

The expected one set of NMR spectrum did not appear. Instead, two sets of ^{1}H NMR spectrum were recorded.

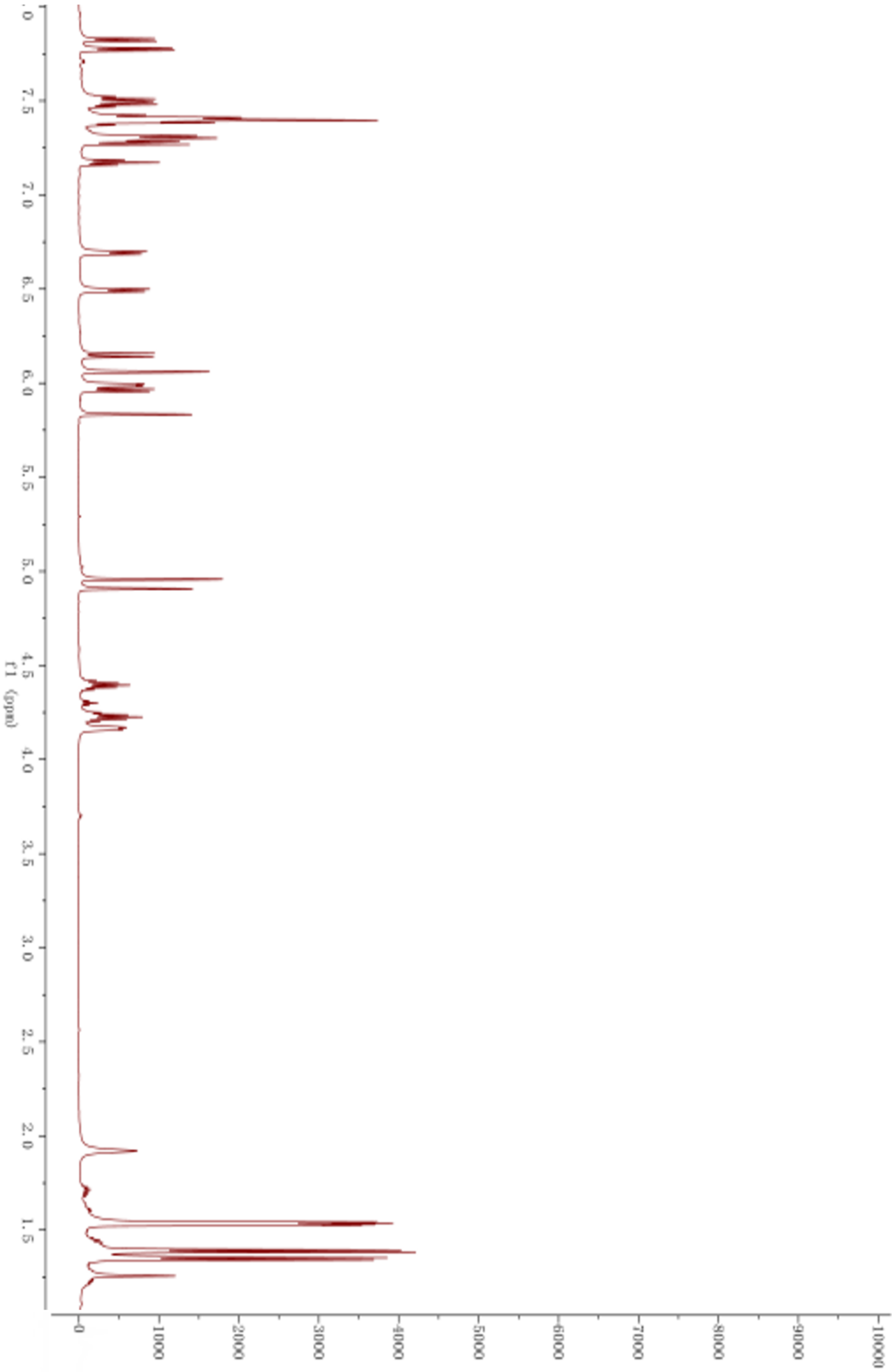

Figure S9. The ^{1}H NMR spectrum for single enantiomer of (*R,S,S*)-**4** in pure $CDCl_3$. The solubility of enantiomer **4** in $CDCl_3$ is higher than the racemic **4**.

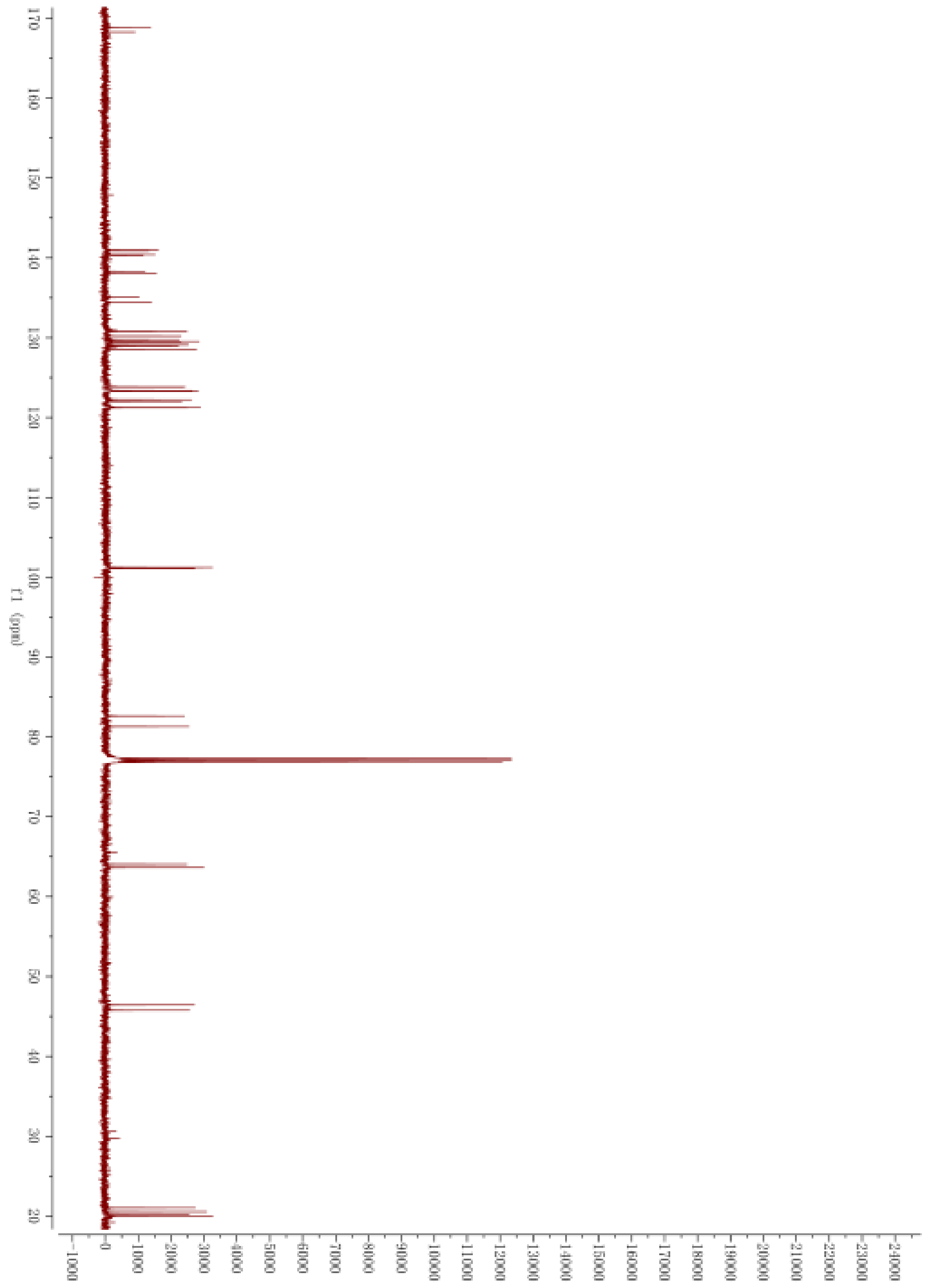

Figure S10. The ^{13}C NMR spectrum for single enantiomer of (*R,S,S*)-**4** in $CDCl_3$.

NOTICE: The ^{1}H and ^{13}C NMR spectra of the single enantiomer of (*R,S,S*)-**4** in CD_3OD were the same as those of racemic **4**. Two copies are pasted here using Figure 9A and Figure 10A, respectively, below.

^{1}H NMR (600 MHz, CD_3OD) δ 7.71 (d, *J* = 7.4 Hz, 1H), 7.44-7.36 (m, 4H), 7.28 (t, *J* = 7.5 Hz, 1H), 7.02 (d, *J*= 7.2Hz, 1H), 6.38 (d, *J* = 7.3 Hz, 1H), 6.11 (s, 1H), 5.78 (s, 1H), 5.10 (s, 1H), 4.27 (m, 1H), 3.32 (s, 1H), 1.46 (t, *J* = 6.7 Hz, 6H).

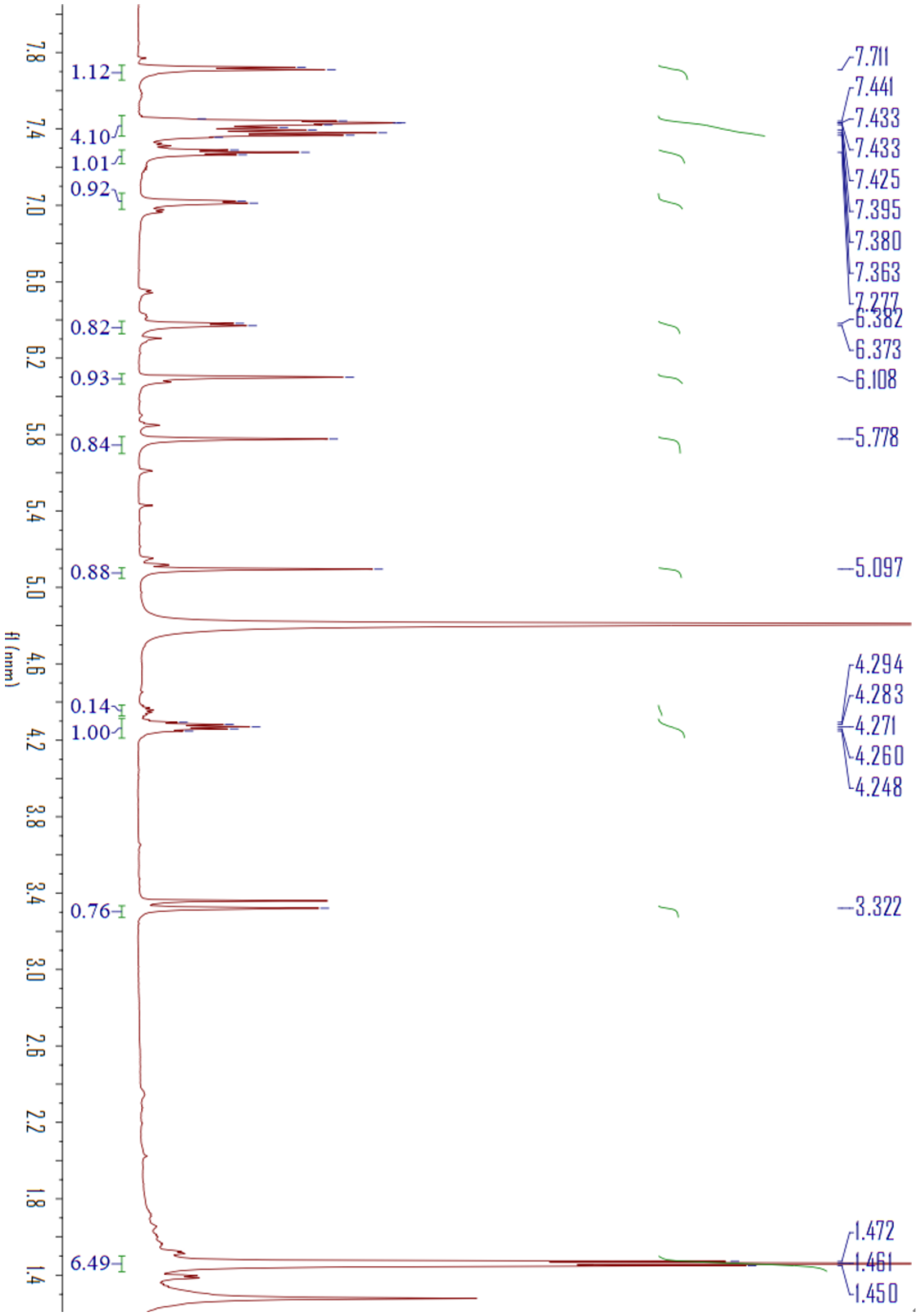

Figure S9A. ^{1}H NMR for single enantiomer of **4** in CD_3OD. There is one set of signals with about 12% of content were observed. For clarity in discussion, only major product's 1H NMR signals were selected here.

^{13}C NMR (151 MHz, CD_3OD) δ 170.71, 142.50, 141.83, 139.36, 135.06, 131.89, 130.17, 129.92, 129.43, 124.12, 123.55, 122.34, 102.04, 82.73, 79.03, 78.81, 78.59, 65.42, 47.72, 30.47, 20.67, 20.21.

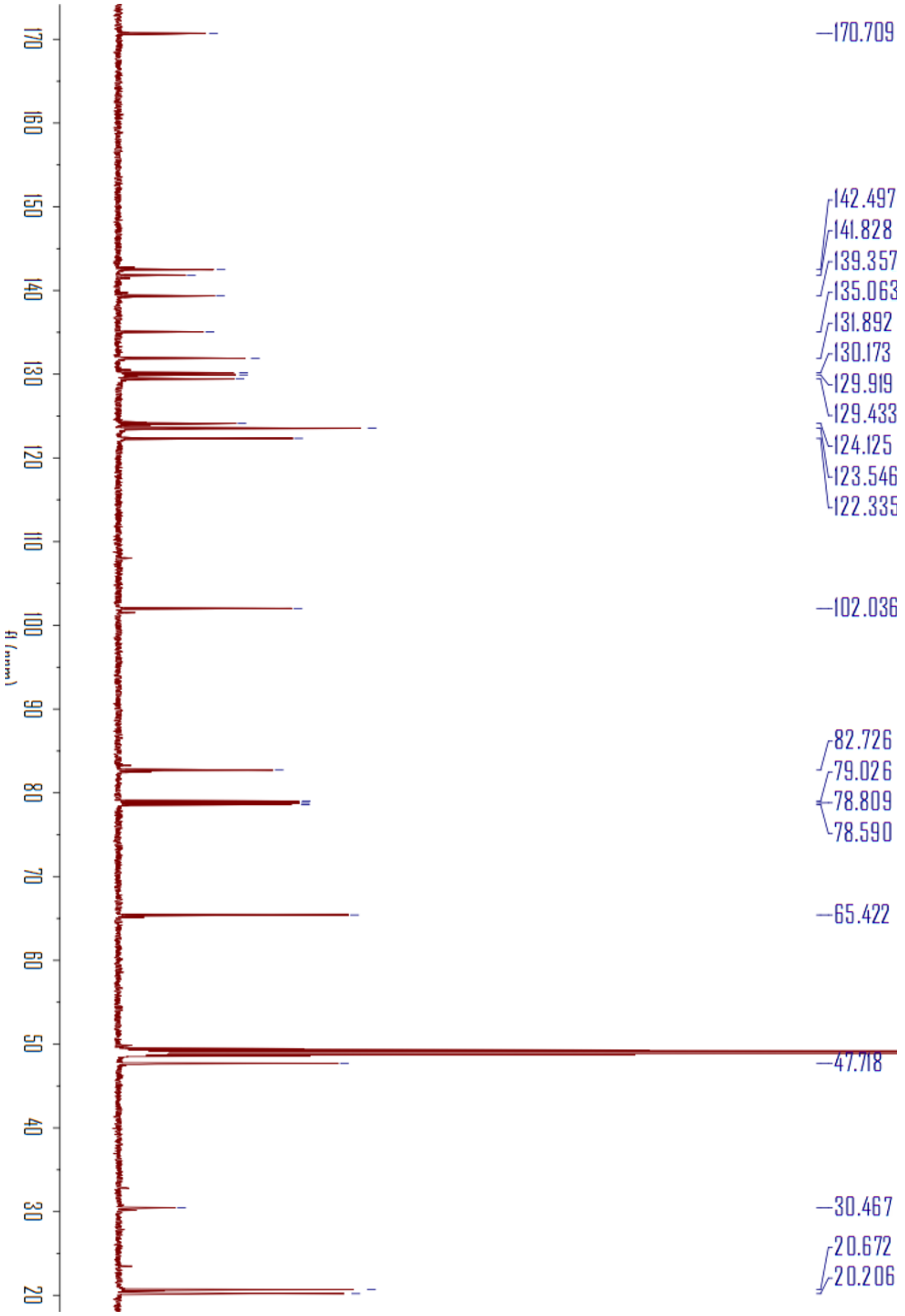

Figure S10A. ^{13}C NMR for single enantiomer **4** in CD_3OD.

Relative configuration of compound 4 and its structure

(1) Crystal quantificational formation test. Total pure enantiomer (signal d in HPLC plot, Figure 2) 50.0 mg were dissolved in methanol. It was placed in the 4-8 °C environment for about one week. The crystal was then filtered. The solution was then placed at room temperature to evaporate the methanol until the crystal formed again. This was put into the 4-8 °C environment for about one week again. After the crystal was filtered, the left solution was only 0.5 mL. Again, very minor crystal could be obtained via the same procedure. Finally, total 49.6 mg crystal was recovered. It exhibited that almost the obtained compound can form crystal. This pure crystal was used in NMR measurement in different solvents.

(2) X-ray study.

The crystal formed via the quantificational test was used in X-ray experiments. The result confirmed there are four same geometries of (*S*,*R*,*R*)-**4** (relative configuration) were included in one cell. The structures are listed below.

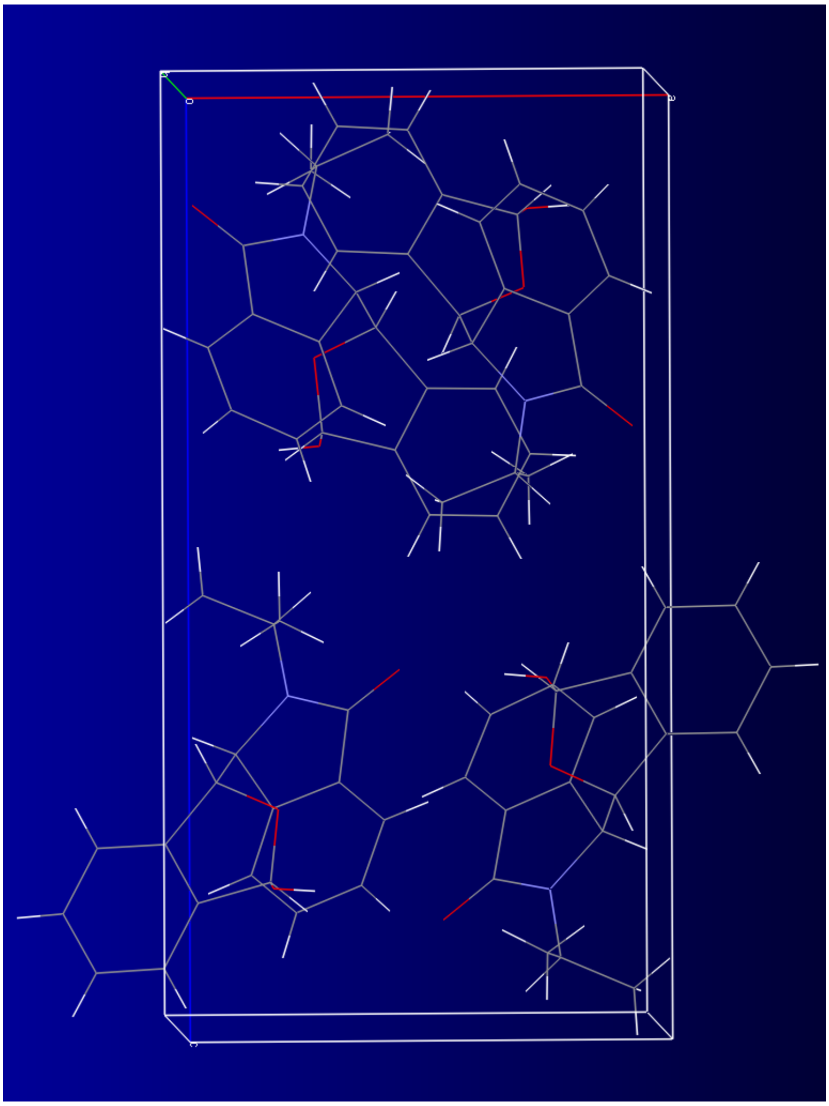

Figure S11. The X-ray structure for the single enantiomer of **4**.

Potential Energy Scan (PES) study

To exclude the possible atropisomer or epimers, the calculations of potential PES were performed. They were performed at the B3LYP/6-311++G(2d,p) level in the gas phase. Its PES plot is illustrated below. Total 71 steps were performed by every 5 degrees by rotating the single bond C3-C1'. Thus, the single bond C3-C1' rotated 355 degrees in the PES study. In the PES plot, the highest barrier was 9.71 kcal/mol when the single bond C3-C1' rotated about 75 degree from the initial 168.9 degree of the dihedral angle of O(2')-C(1')-C(3)-C(4) (bonds in red lines). Only this barrier is highest. The other two barriers were only 6.3 kcal/mol and 4.6 kcal/mol, respectively. They are much small. Thus, the single bond C3-C1' can rotate freely at room temperature. Monomer A is the lowest geometry, monomer B is the second most stable geometry among the three stable geometries in the gas phase. Indeed, there is only one high barrier of 9.7 kcal/mol. Thus, monomer A can change to B more easily via overcoming the other two smaller barriers of 6.3 and 4.6 kcal/mol. The conversion rate should be faster than that via the route by overcoming the highest barrier of 9.7 kcal/mol.

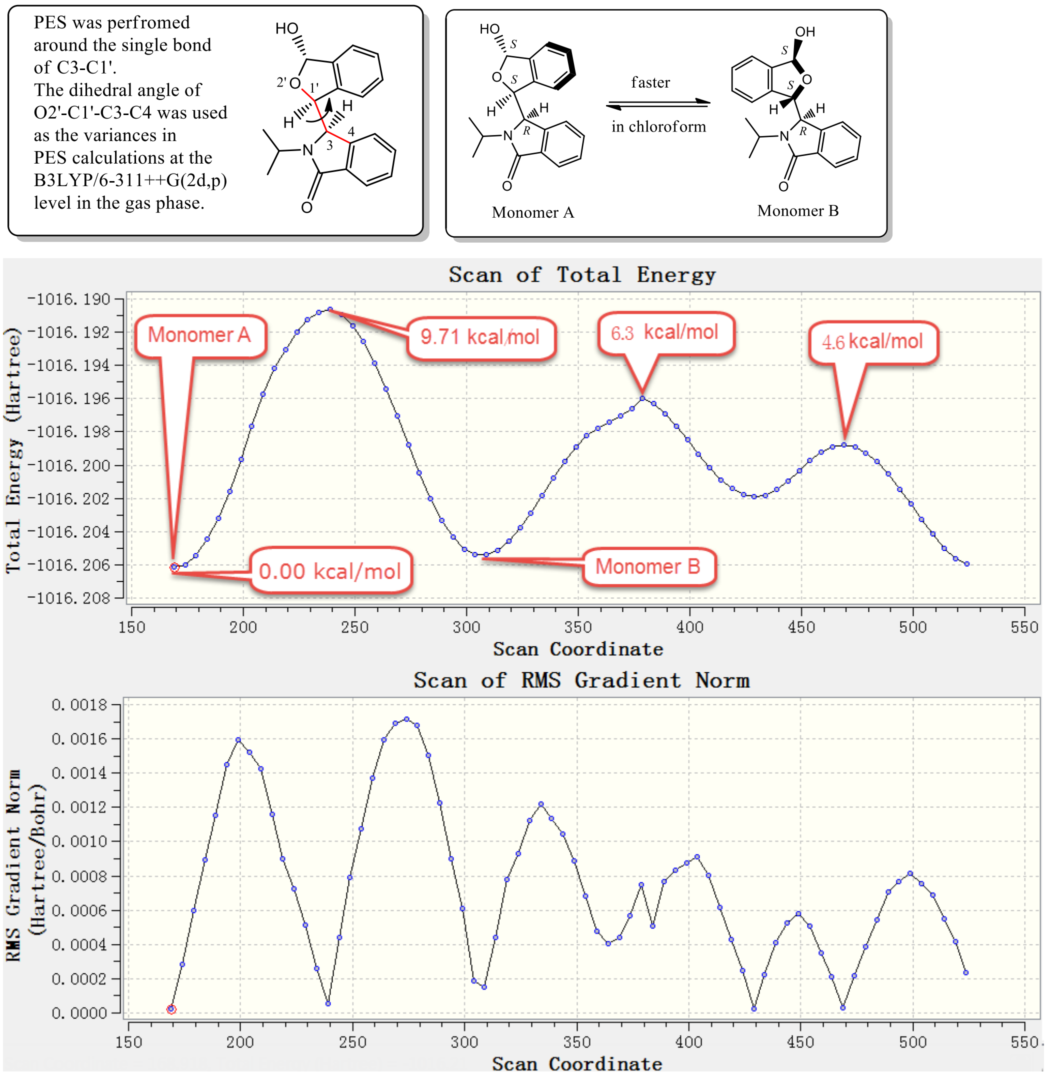

Figure S12. PES calculations for the two geometries A and B. The largest rotation barrier around the single bond C3-C1' is very close to the transition state barrier computation results under different conditions. See the specific transition state barriers data in Table 3.

The ^{1}HNMR measured from 293 K to 213 K in $CDCl_3$ and 298 to 198 K in CD_2Cl_2 .

(1). Measured in $CDCl_3$ from 298 K to 213 K.

Summarized together:

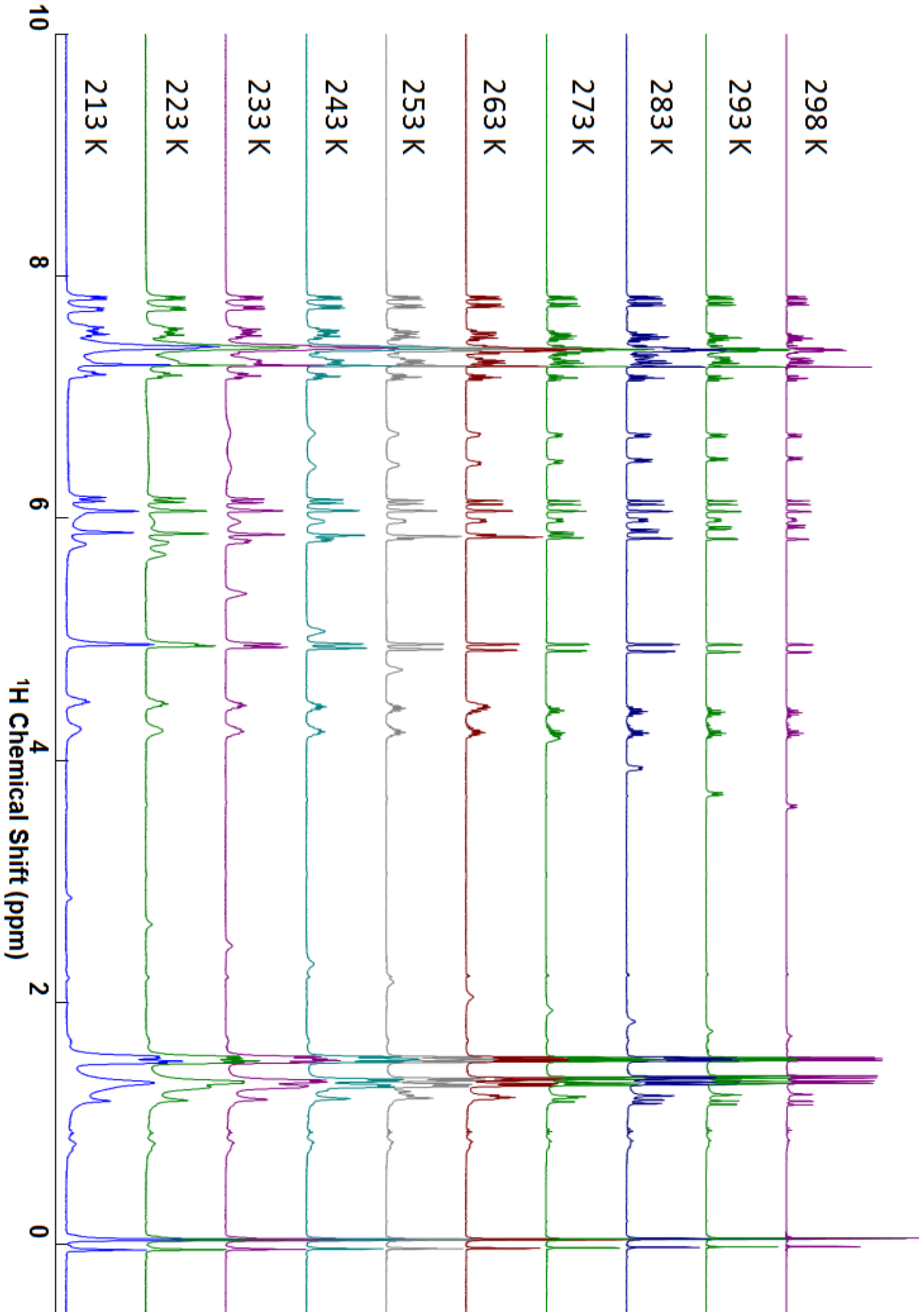

Figure S13. The variable-temperature NMR ^{1}H NMR for enantiomer **4**.

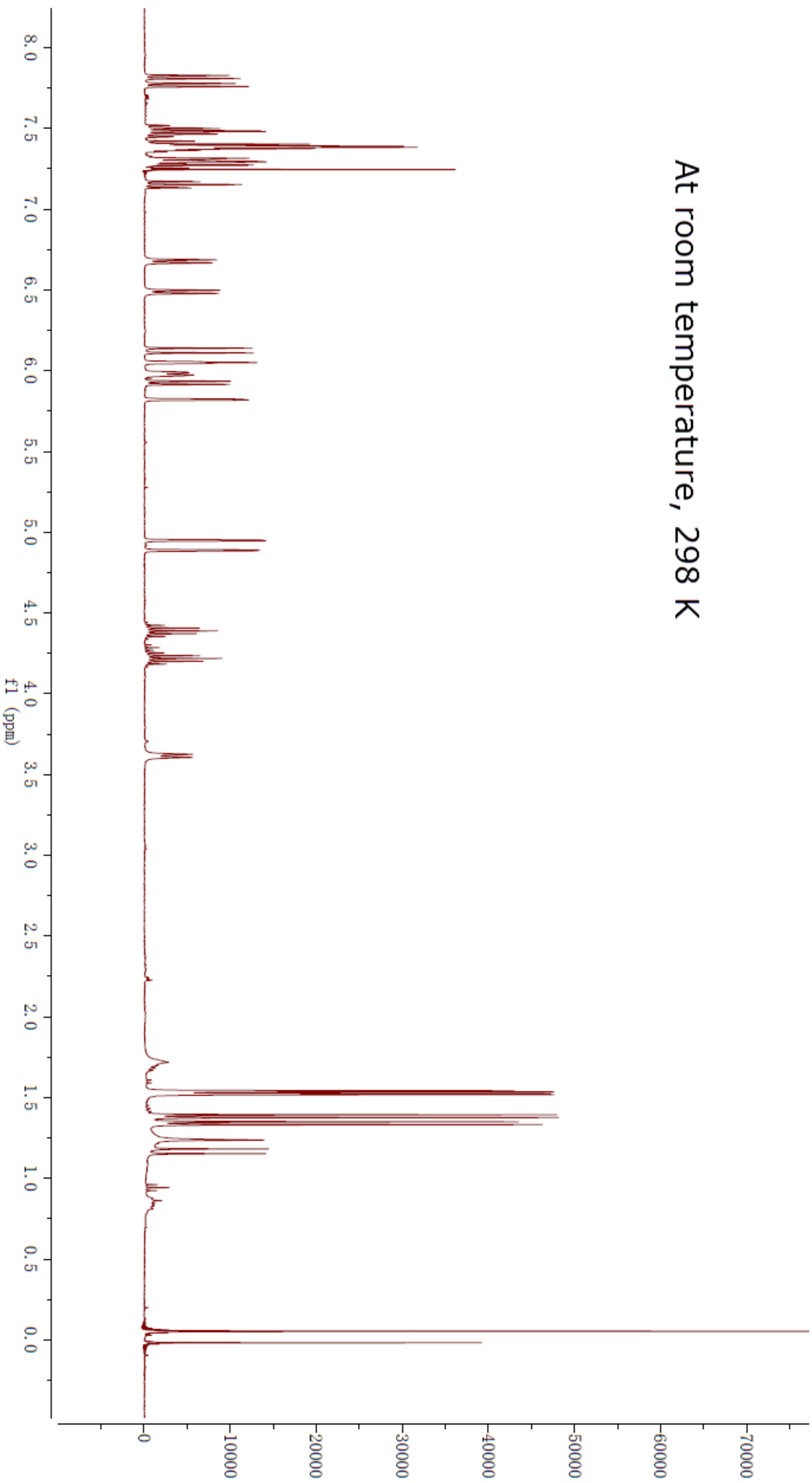

Figure S13. Continued.

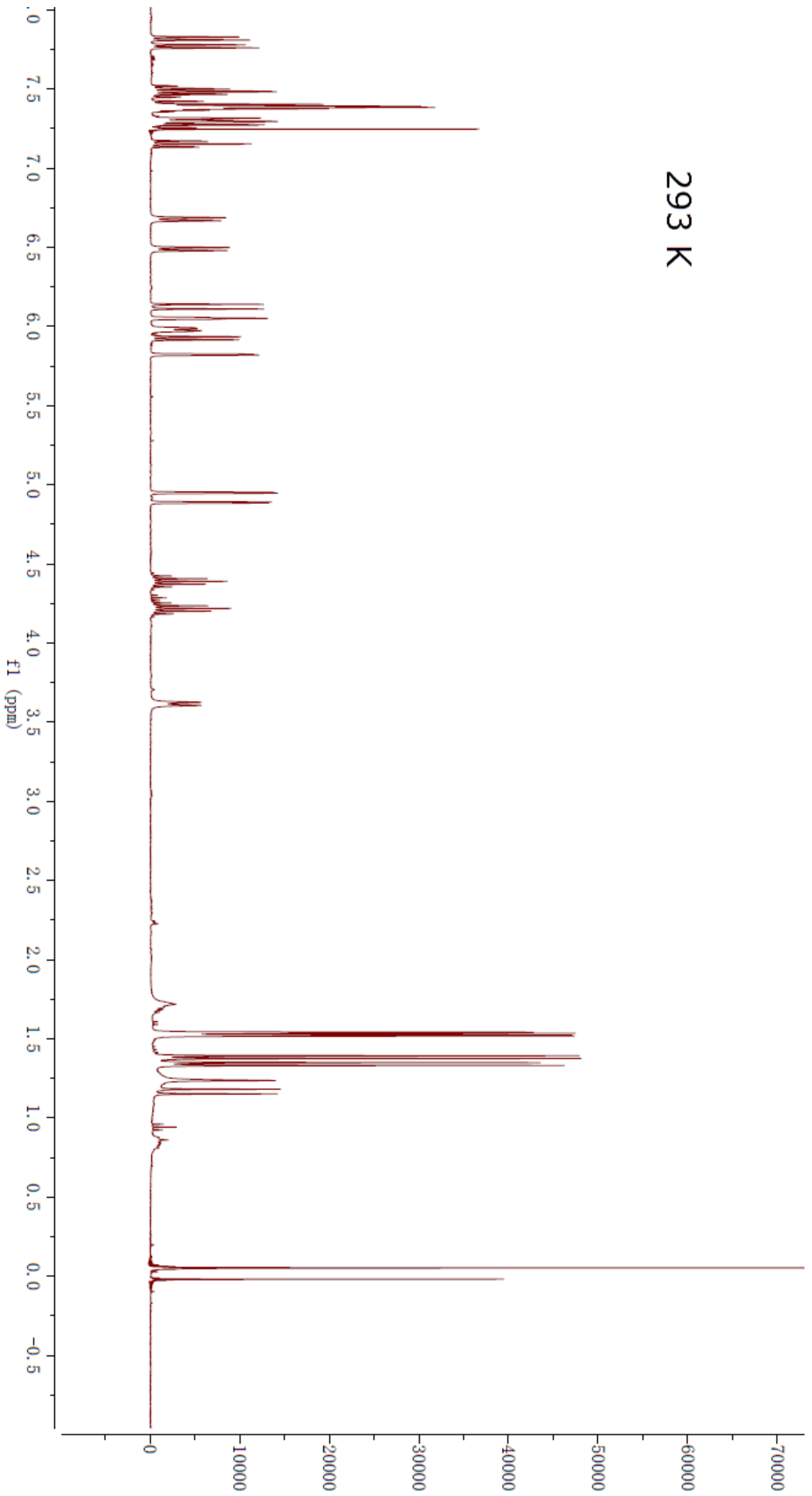

Figure S13. Continued.

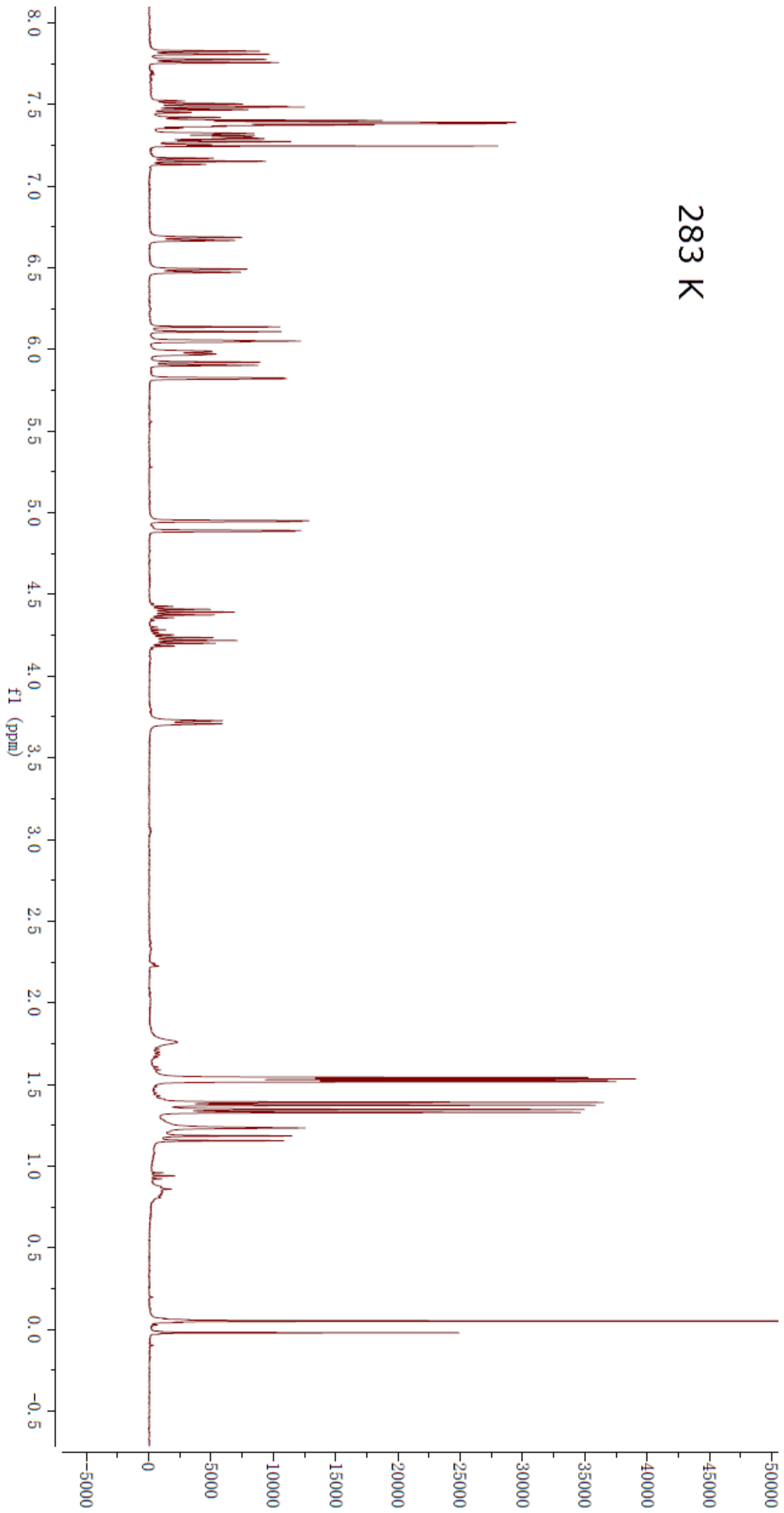

Figure S13. Continued.

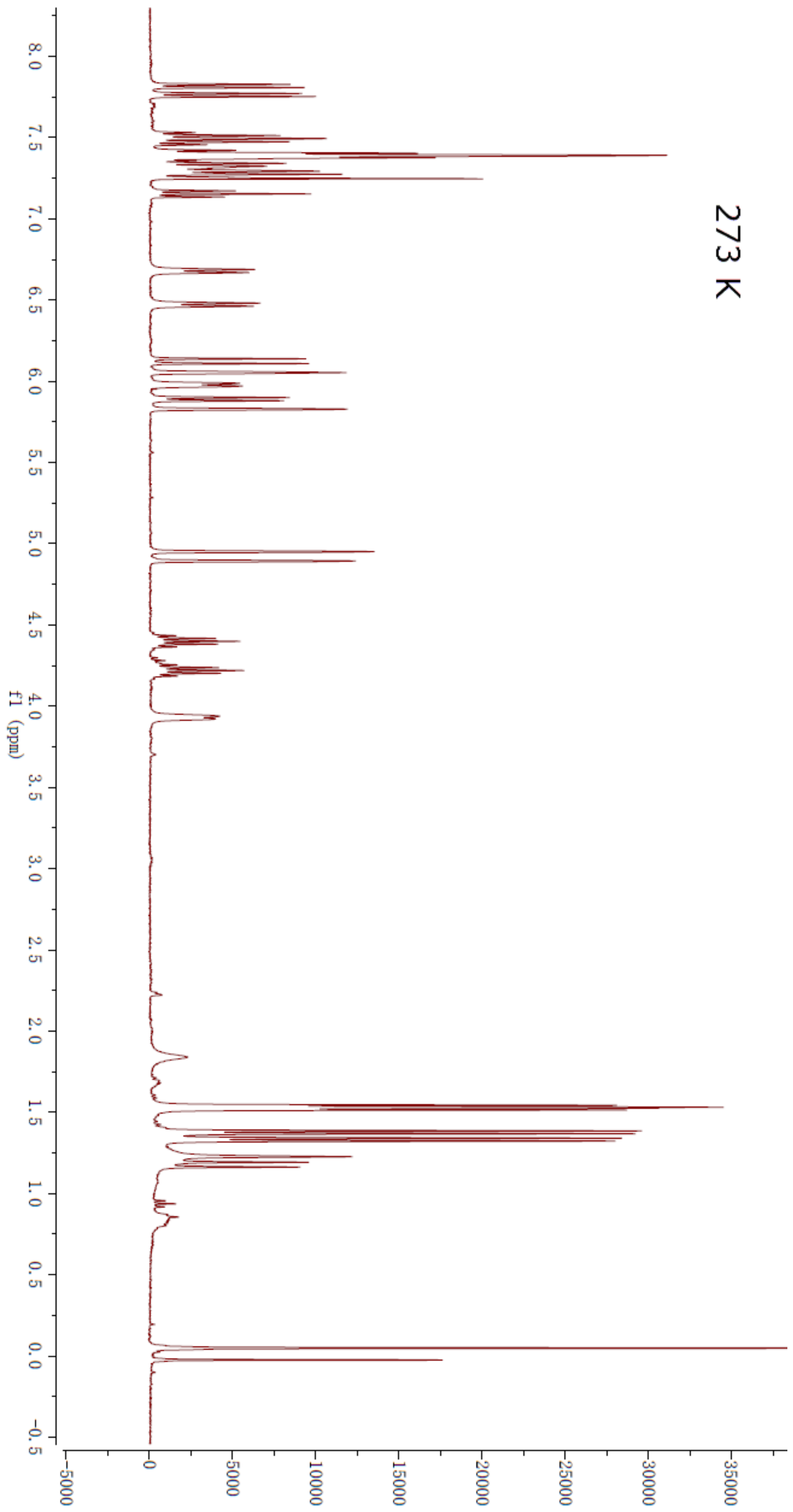

Figure S13. Continued.

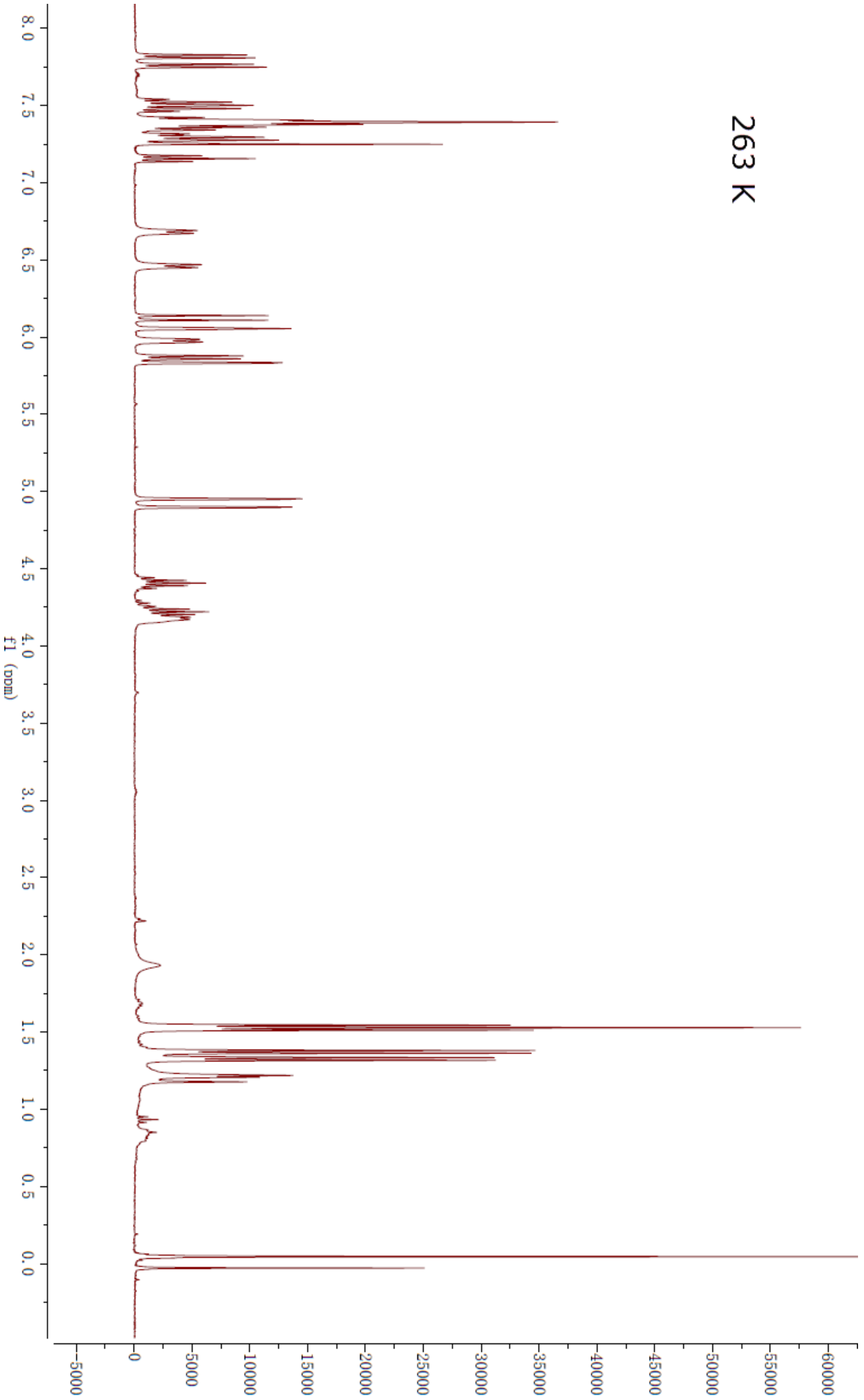

Figure S13. Continued.

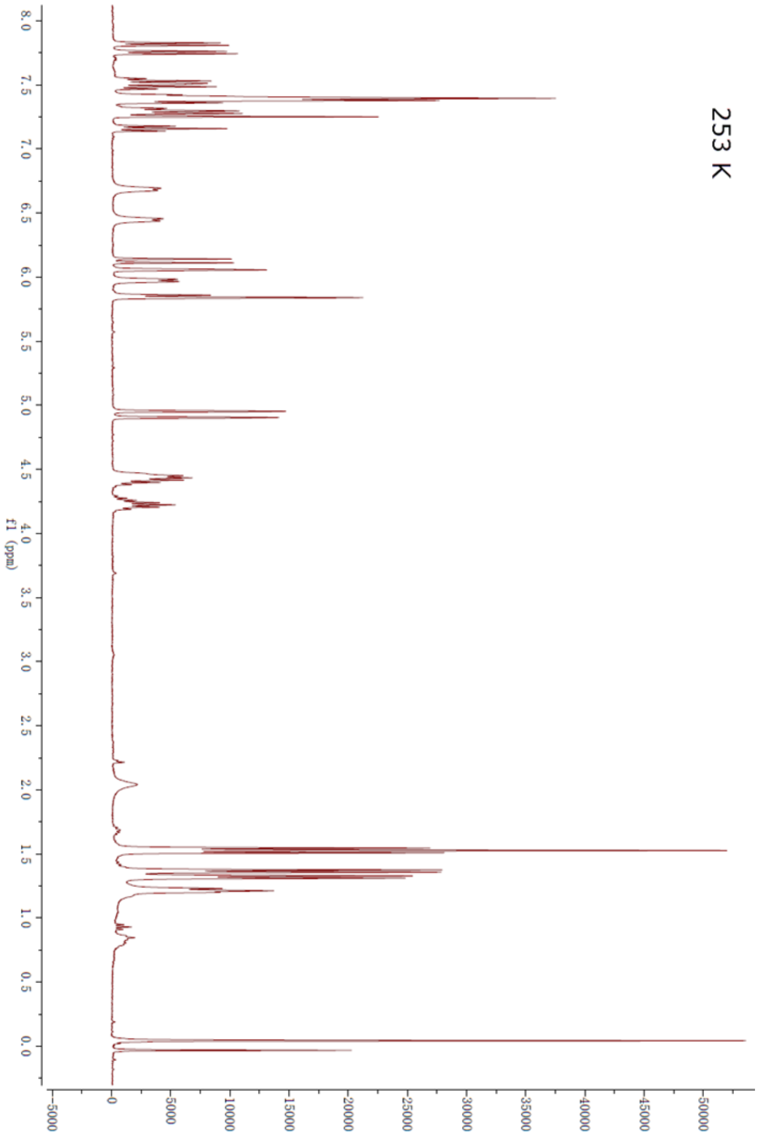

Figure S13. Continued.

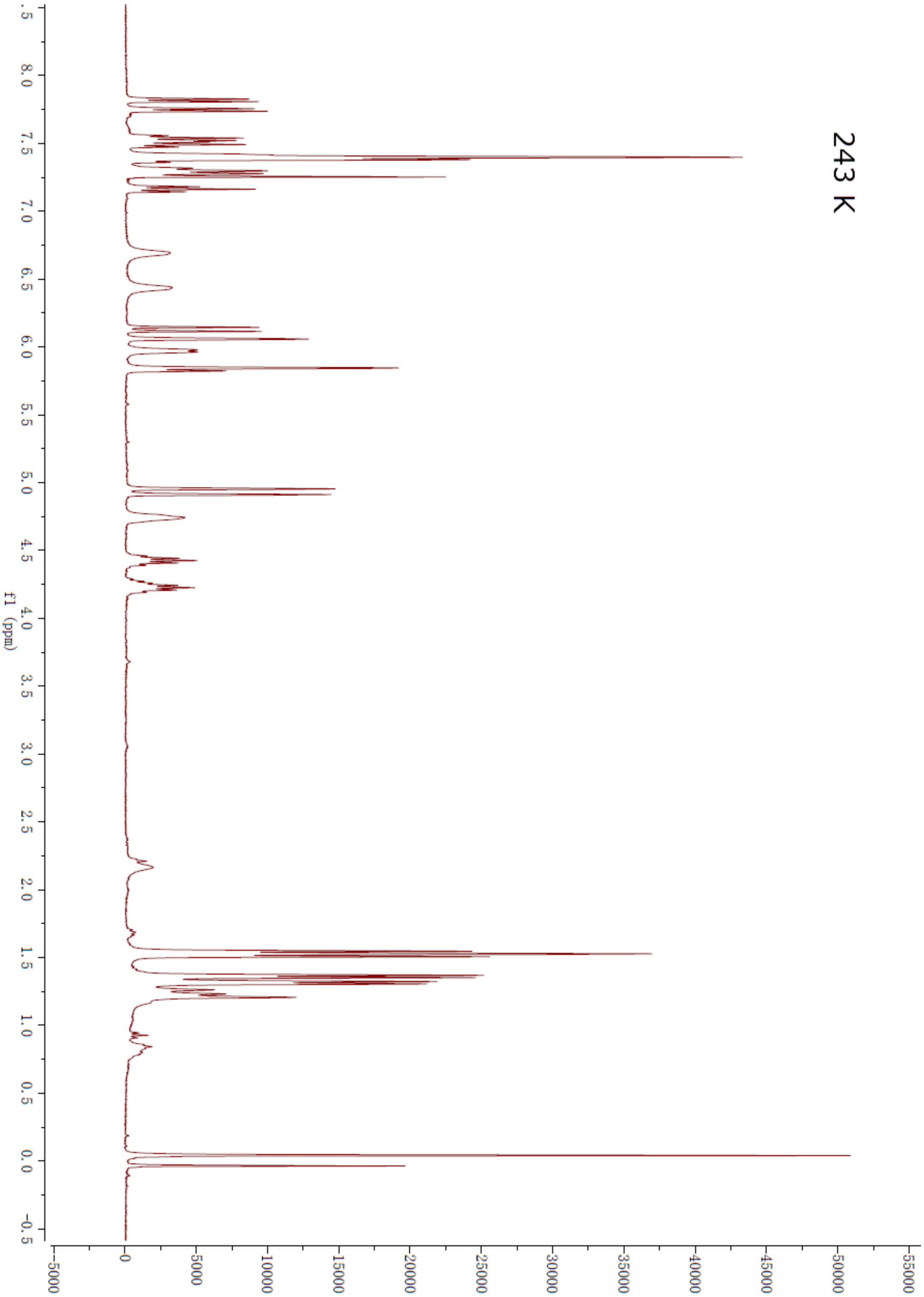

Figure S13. Continued.

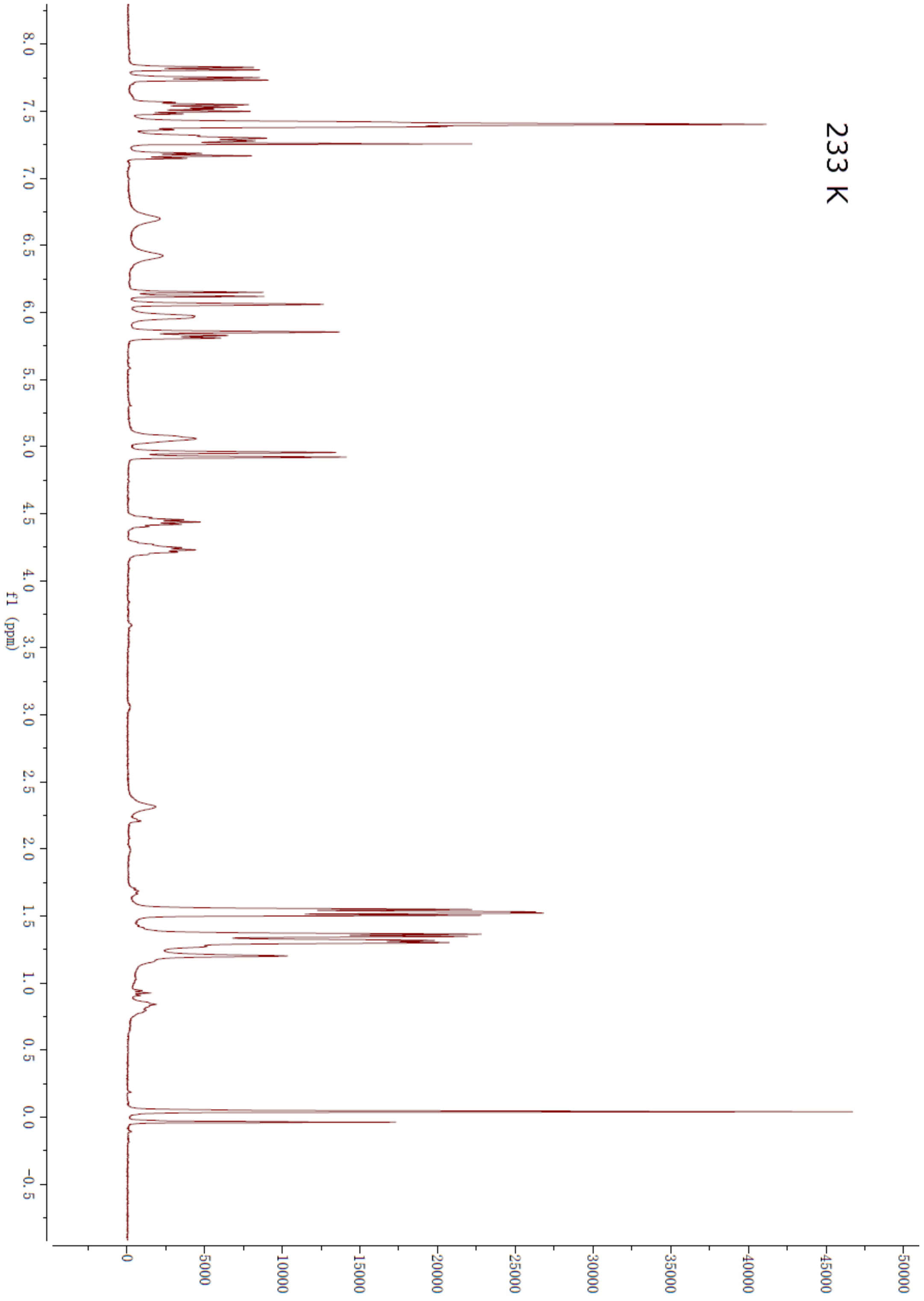

Figure S13. Continued.

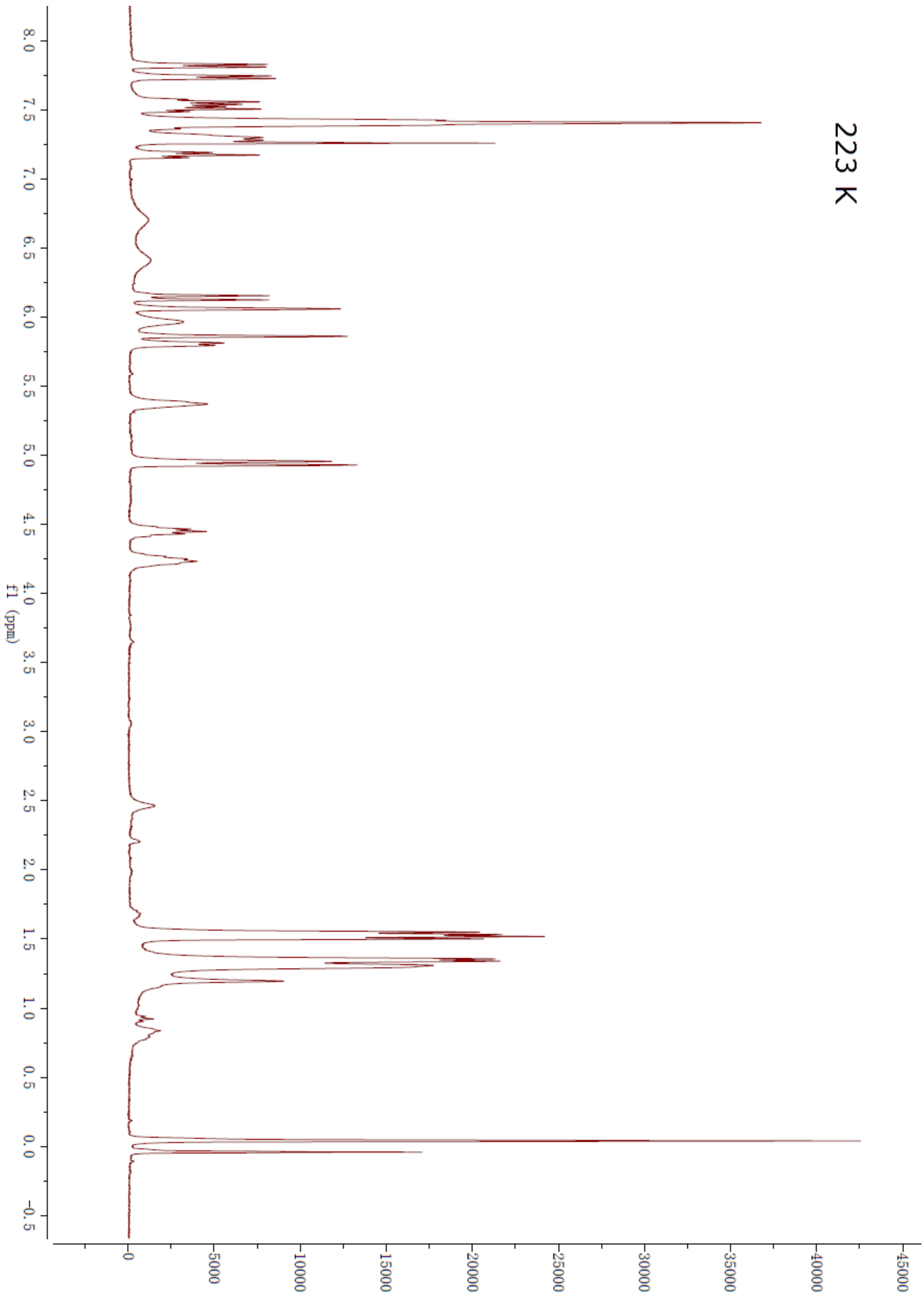

Figure S13. Continued.

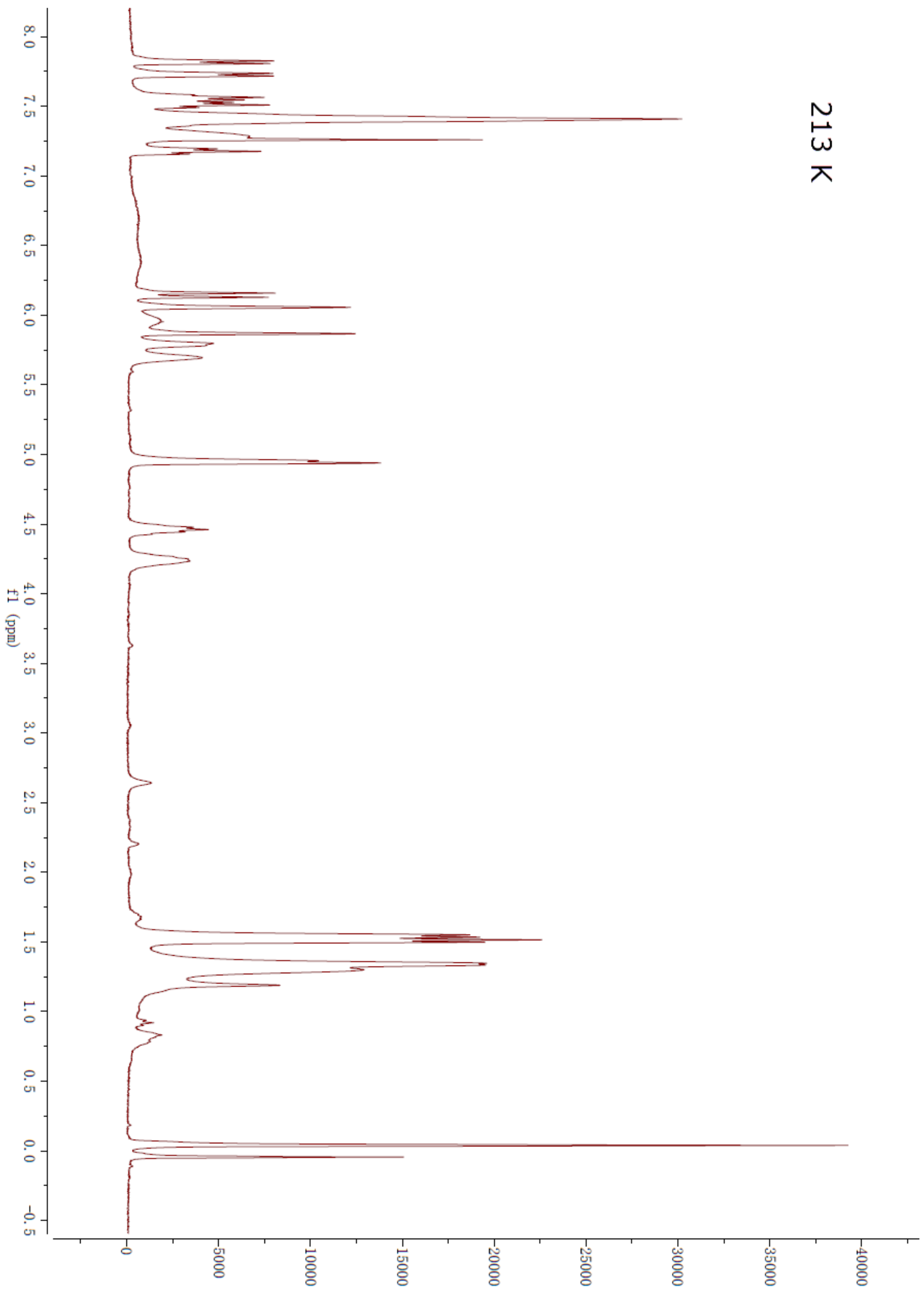

Figure S13. Continued.

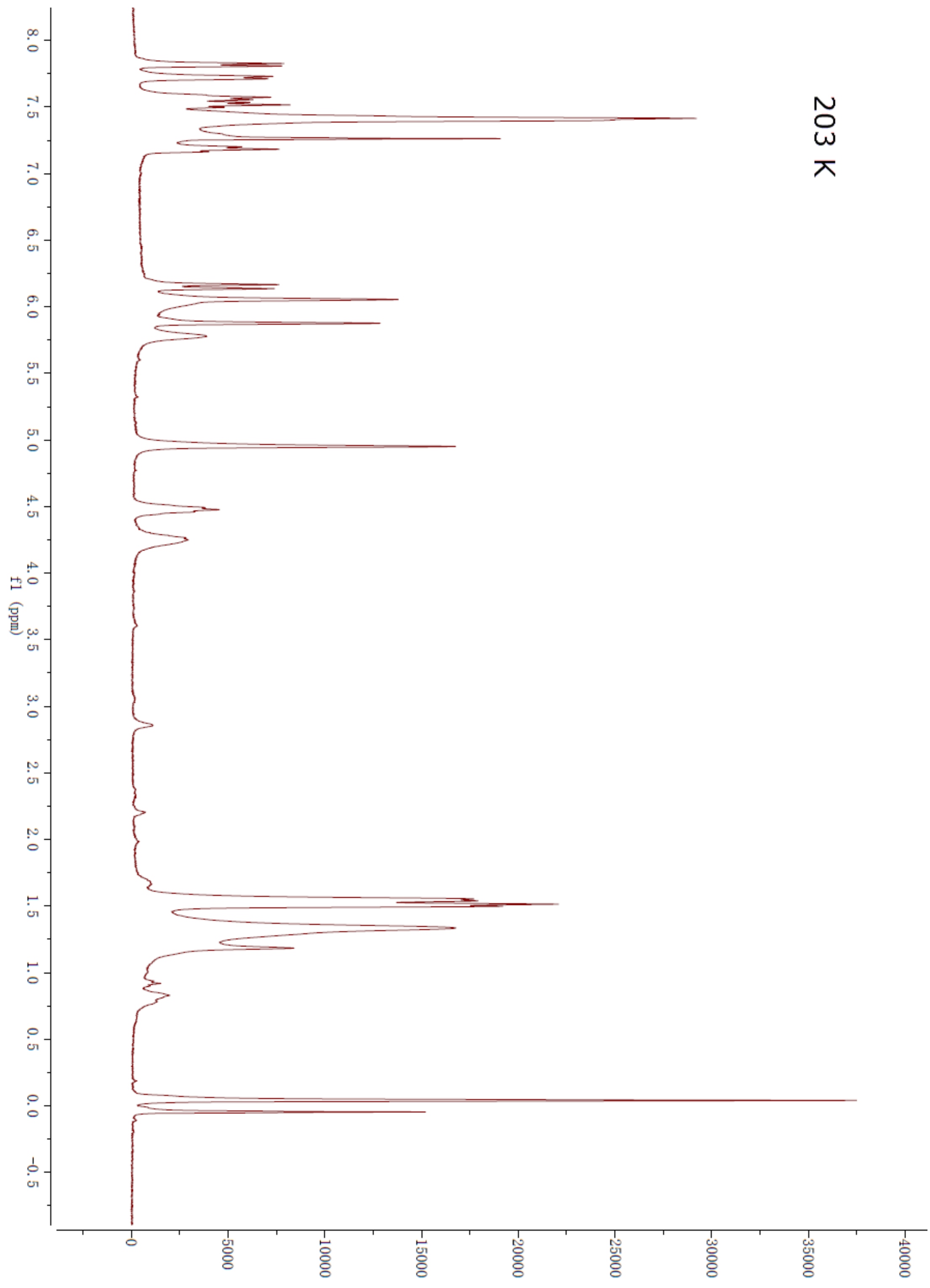

Figure S13. Continued.

Measured in CD_2Cl_2 from 293 K lower to 193 K

It is interesting that the ratio of the two signals near 7.7 ppm are different. This case looks like that measured in CD_3OD.

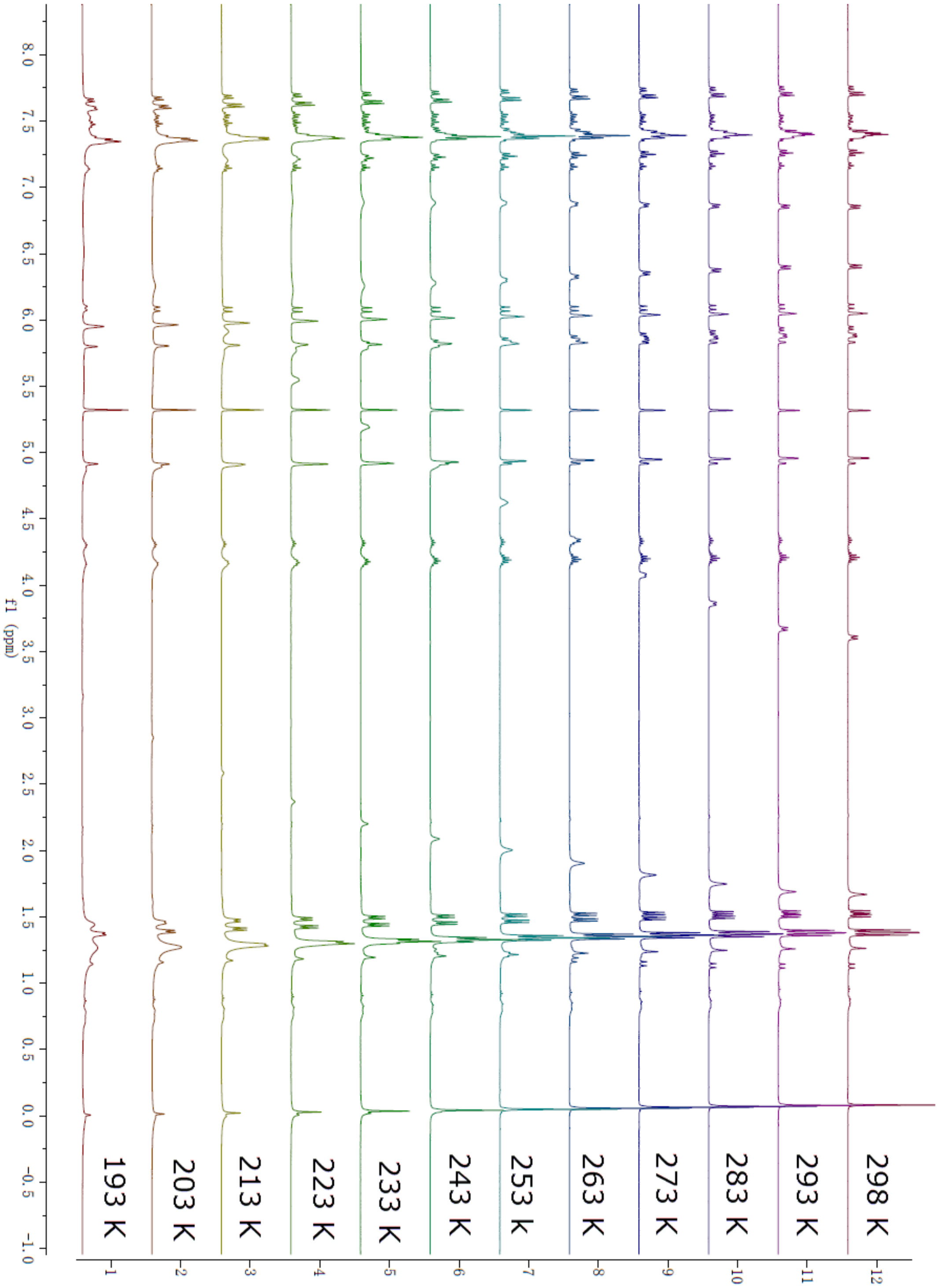

Figure S13. continued.

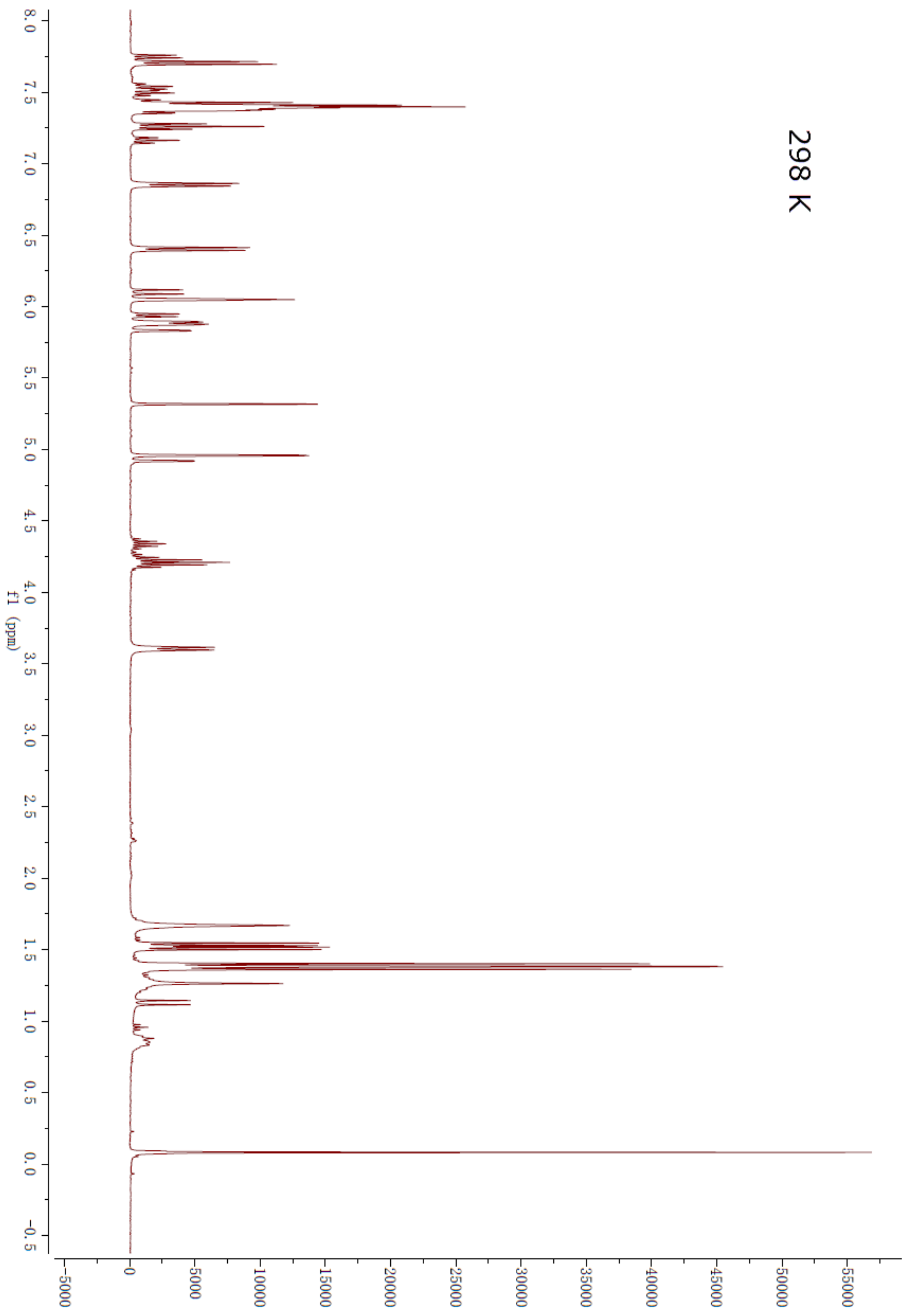

Figure S13. Continued.

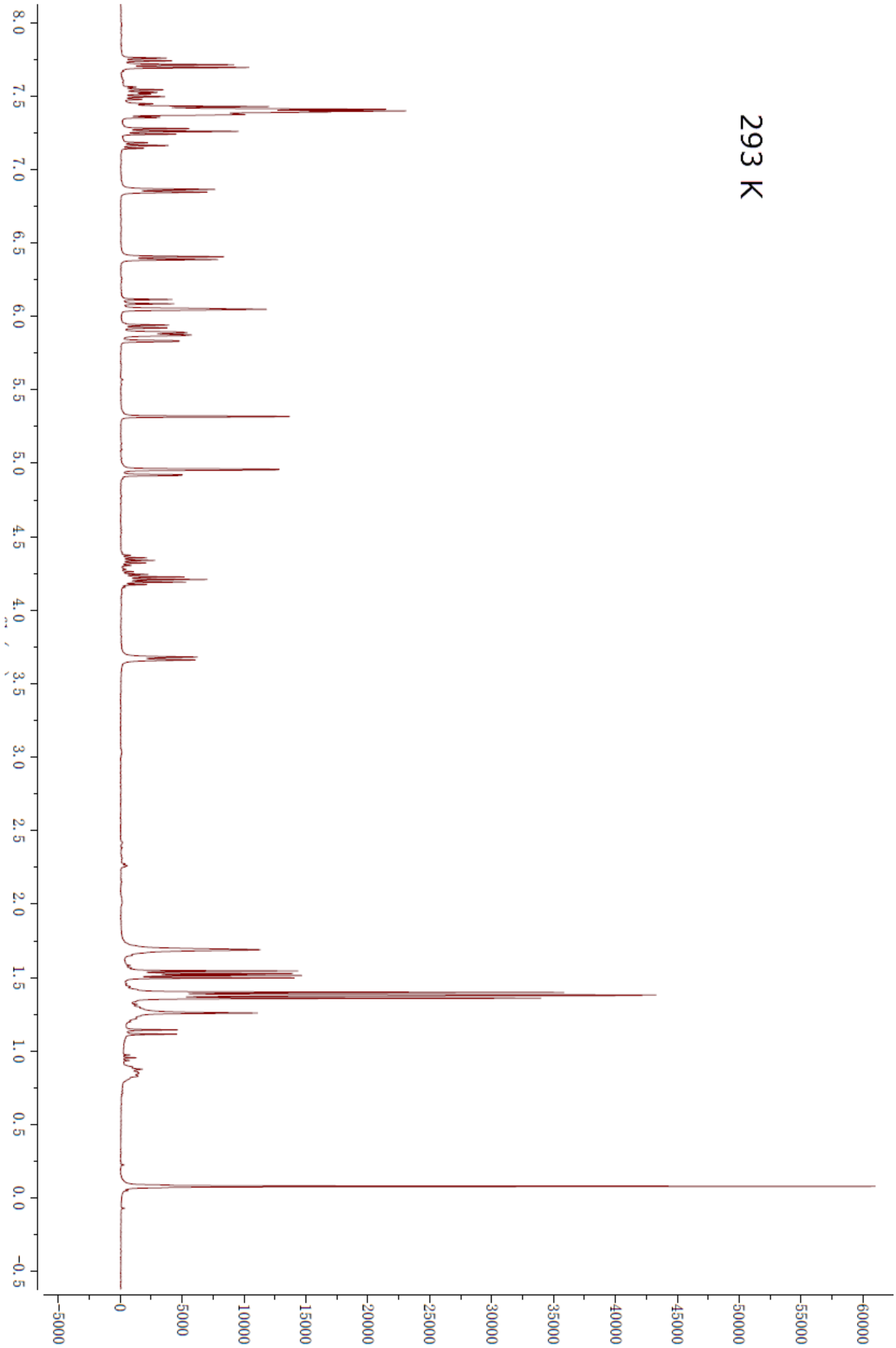

Figure S13. Continued.

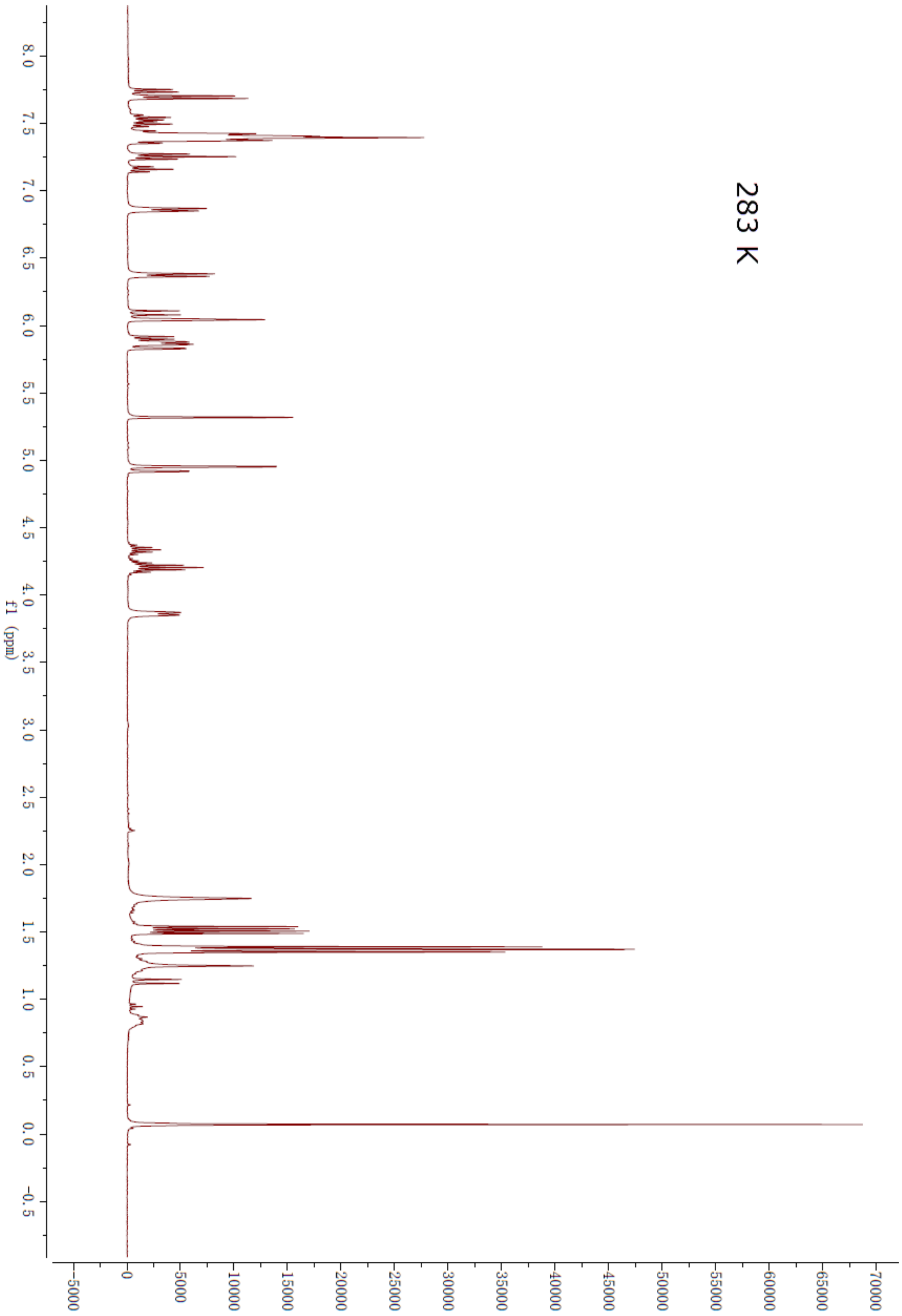

Figure S13. Continued.

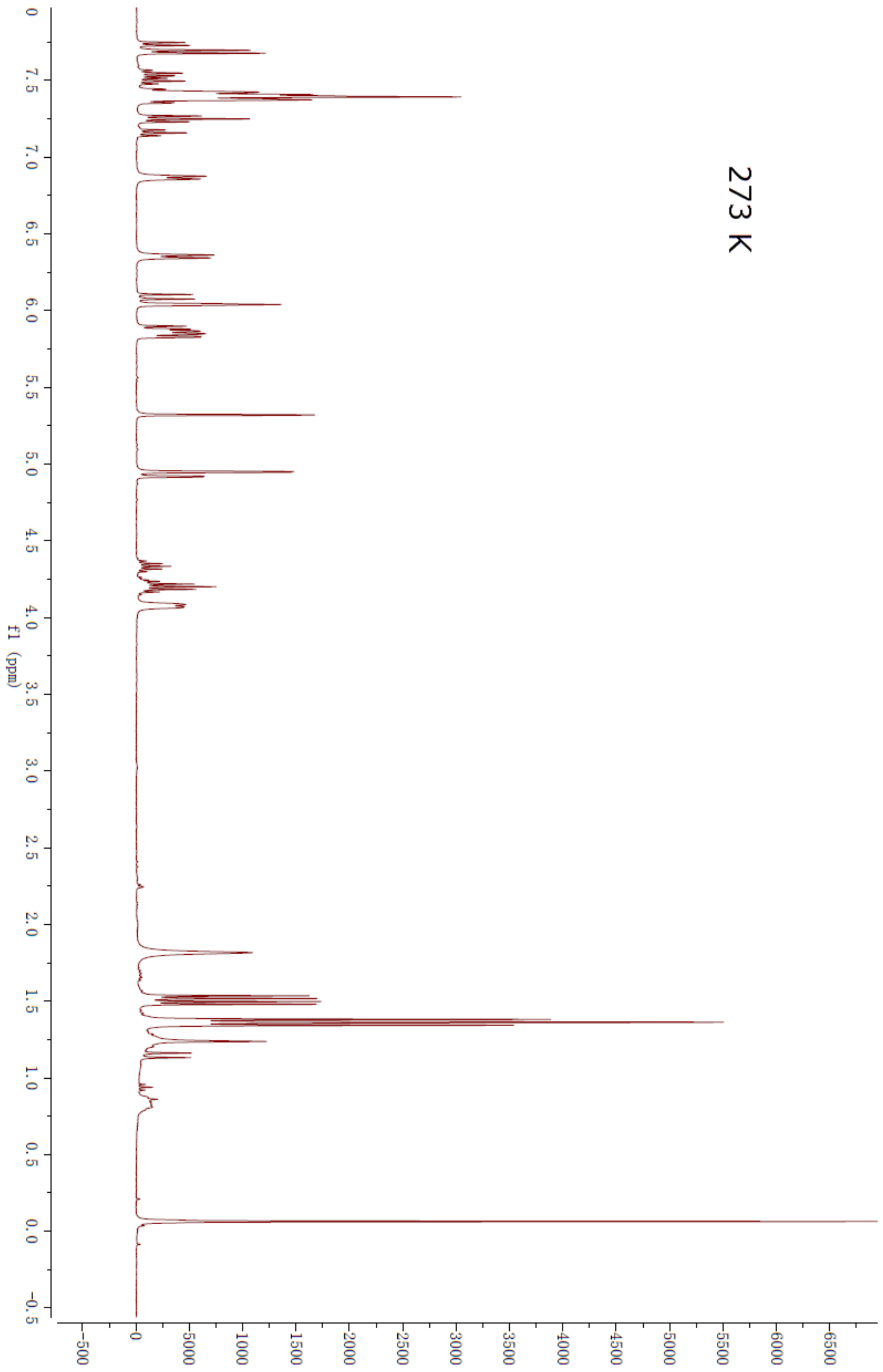

Figure S13. Continued.

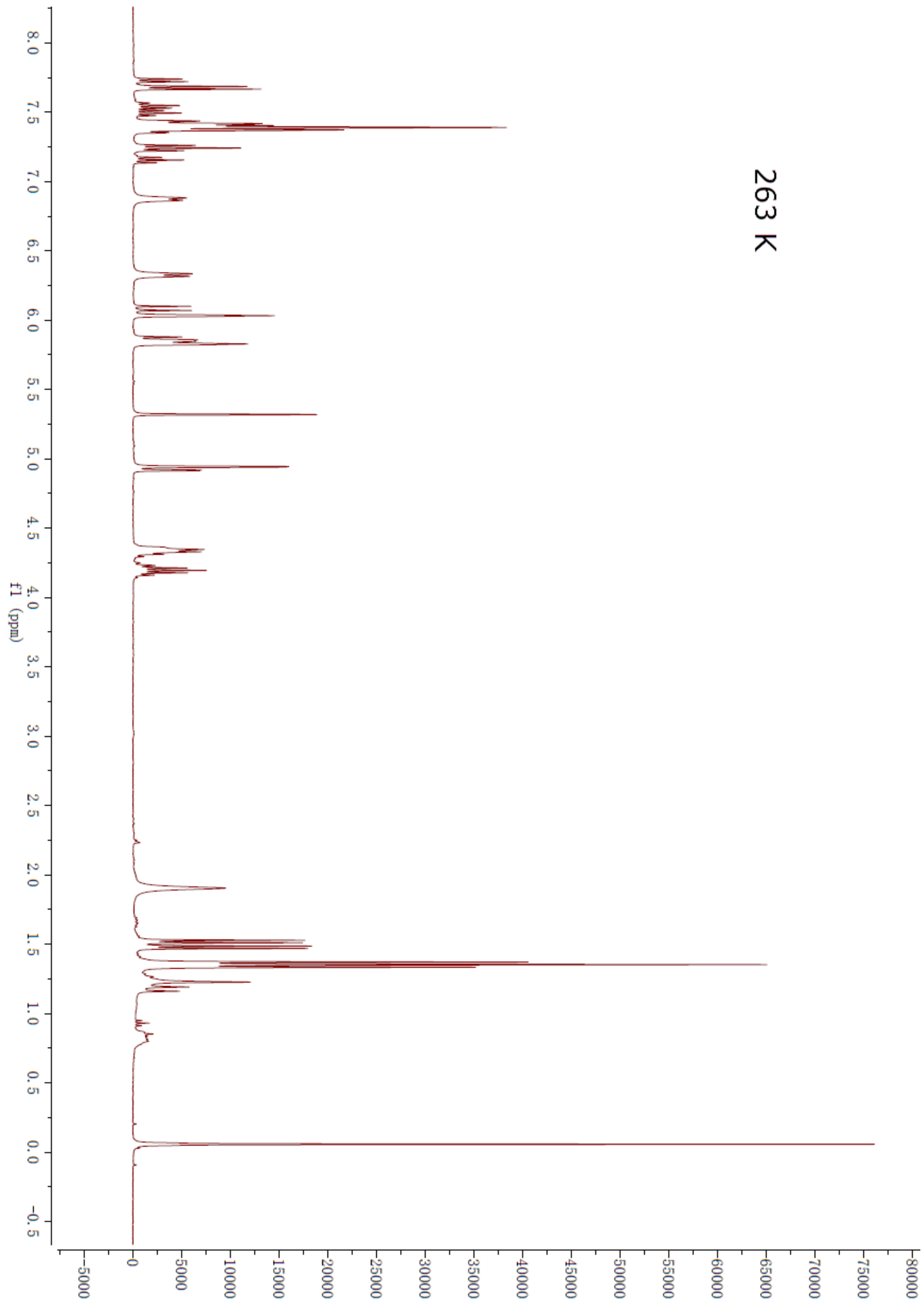

Figure S13. Continued.

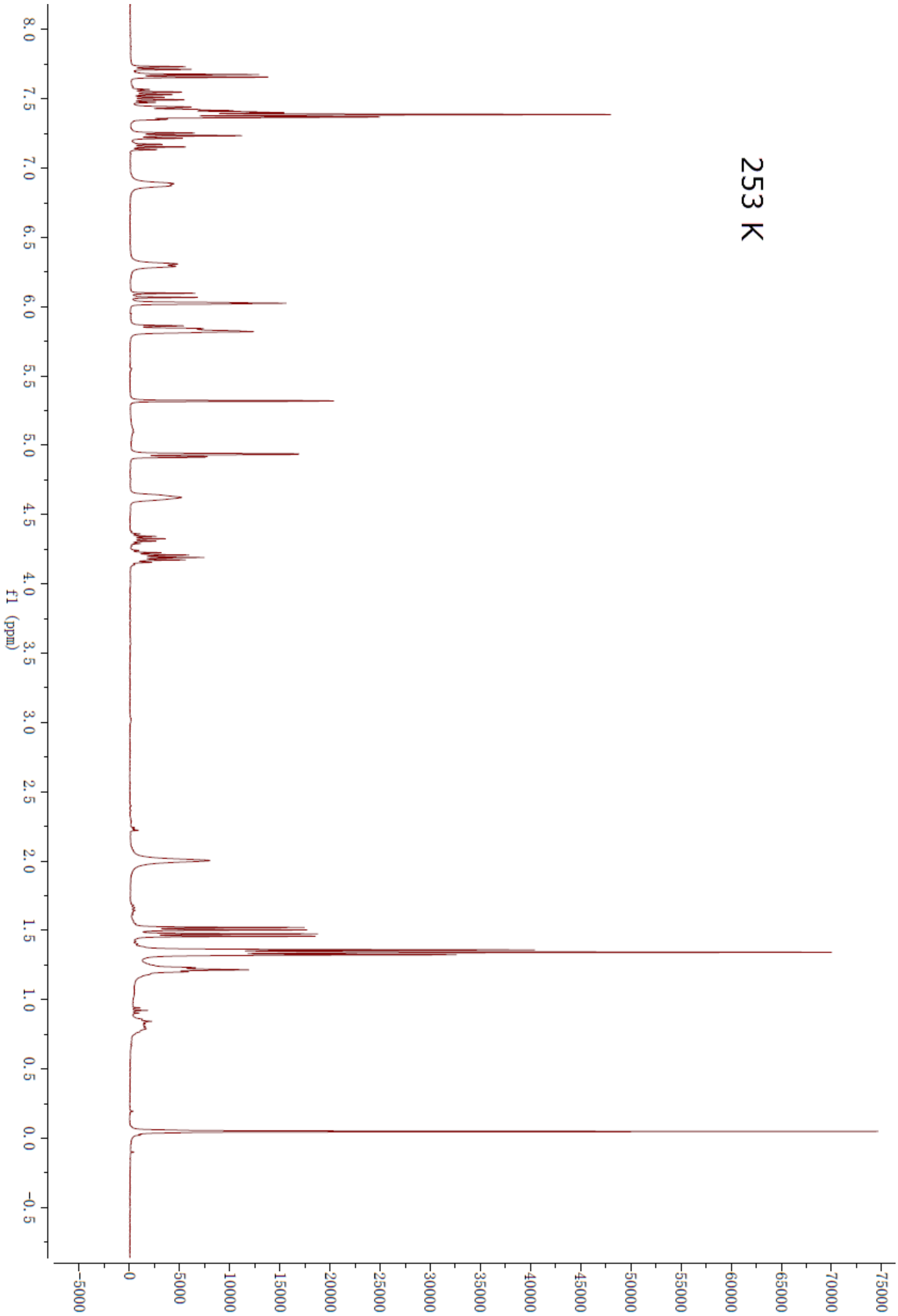

Figure S13. Continued.

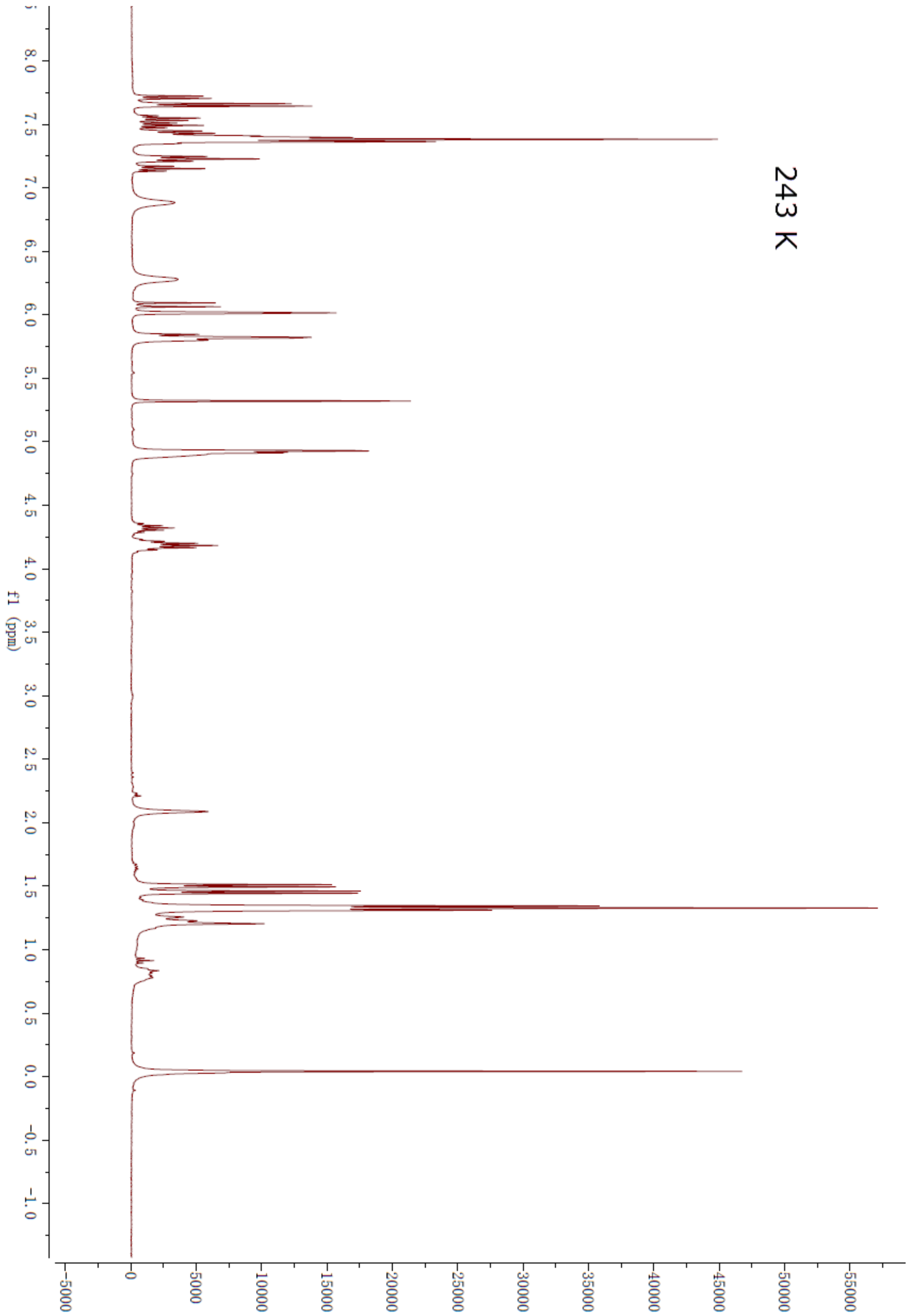

Figure S13. Continued.

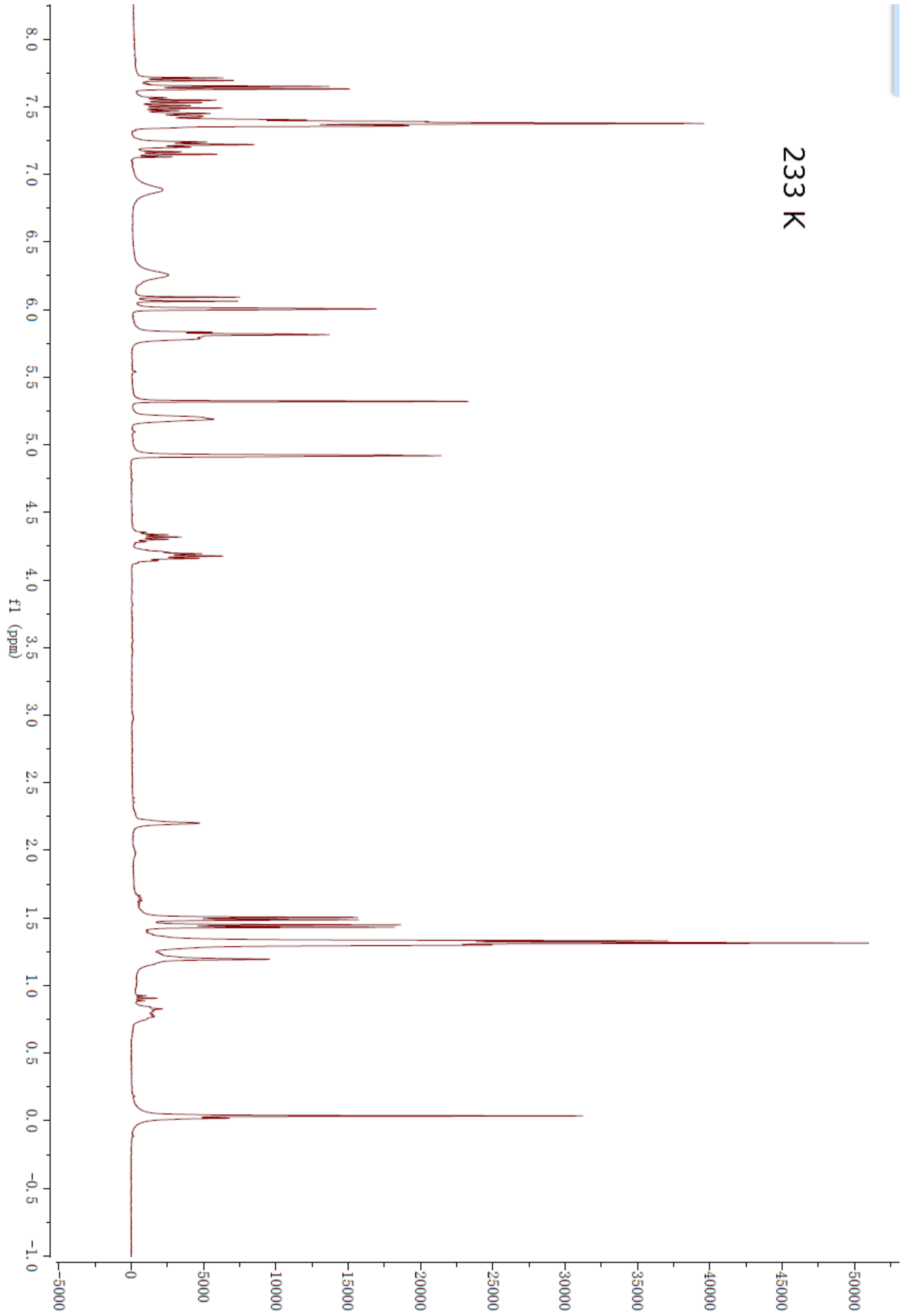

Figure S13. Continued.

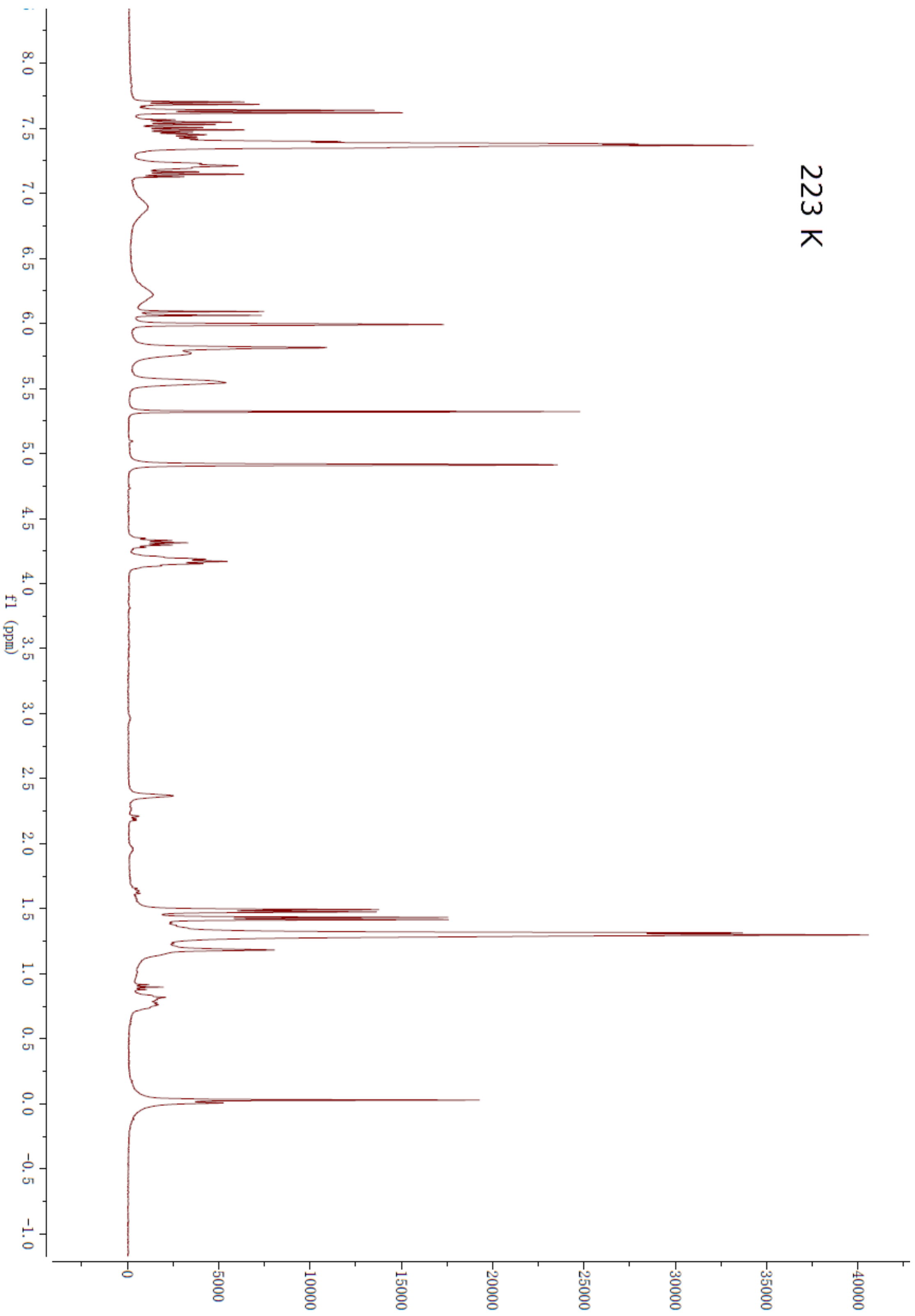

Figure S13. Continued.

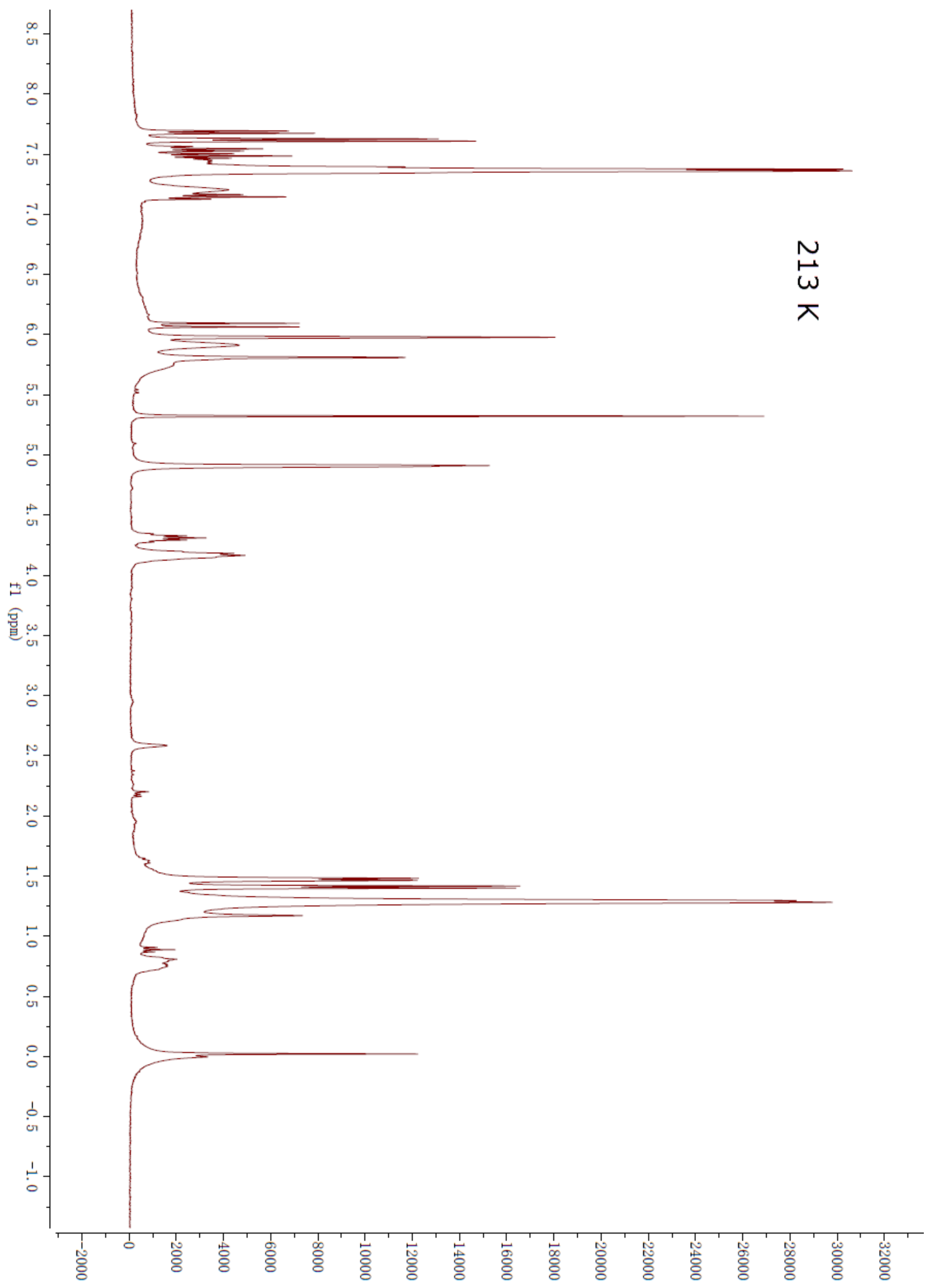

Figure S13. Continued.

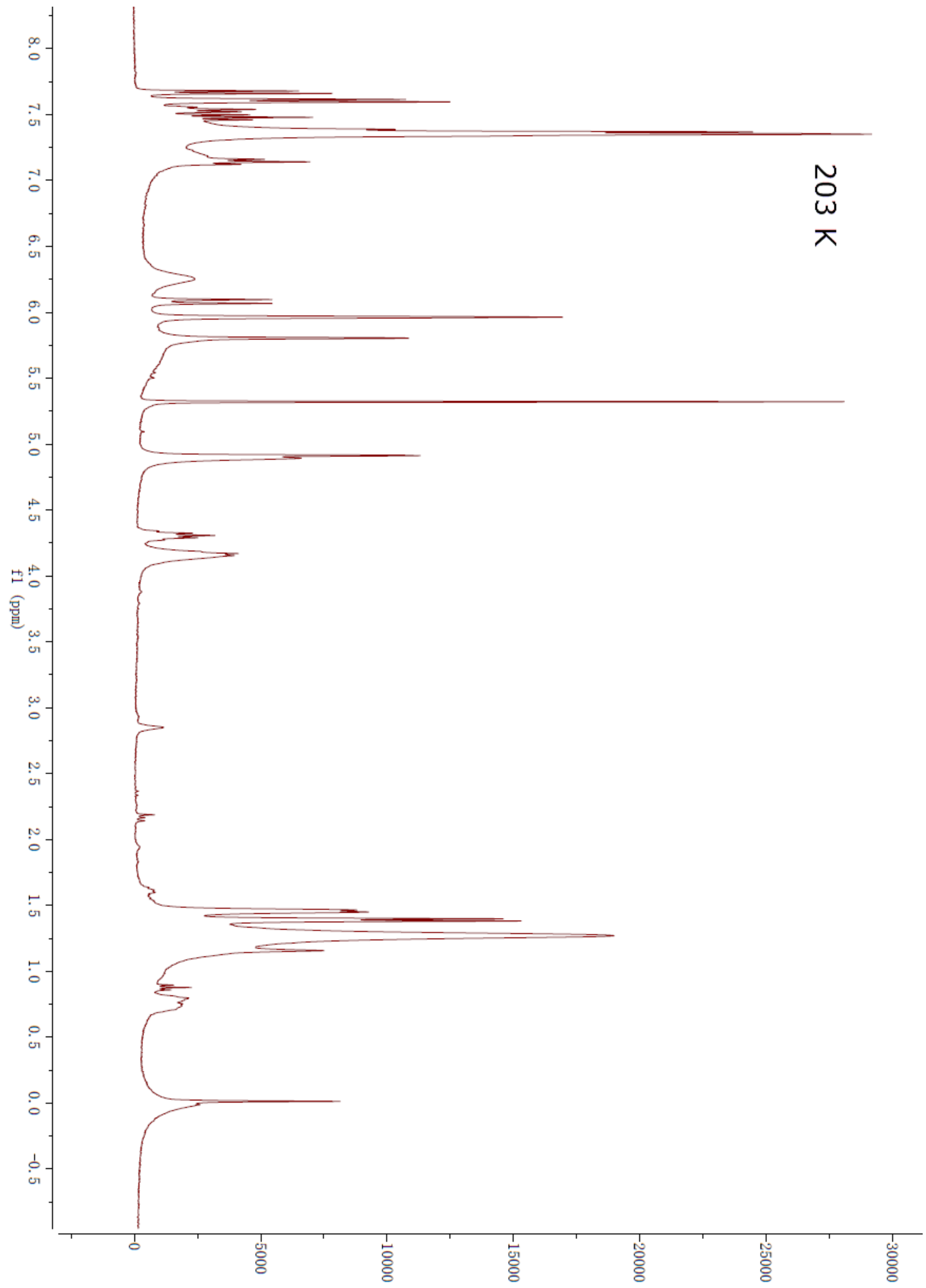

Figure S13. Continued.

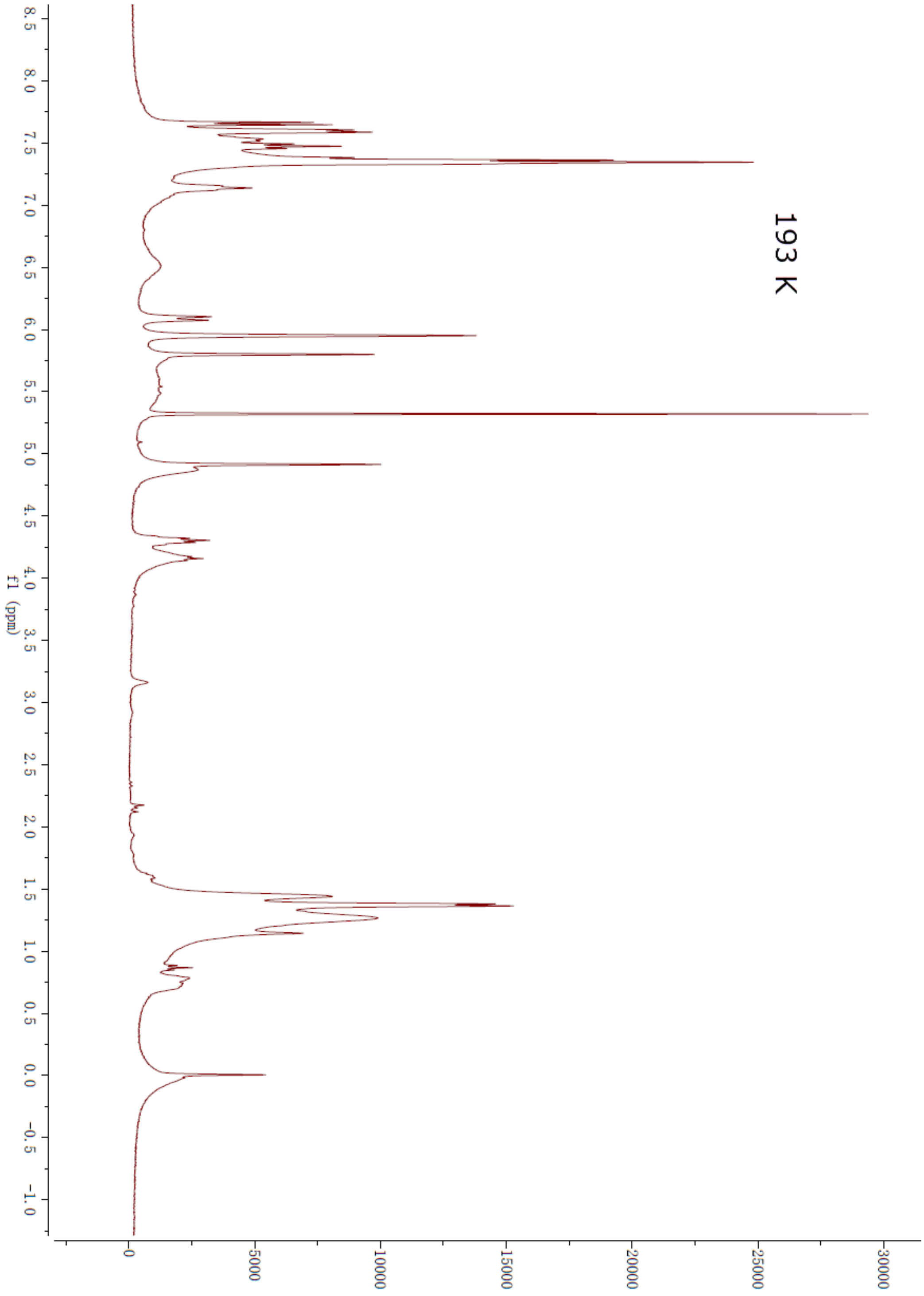

Figure S13. Continued.

The integration of signals at 7.8 ppm and 6.6 ppm in compound **1** under different temperatures

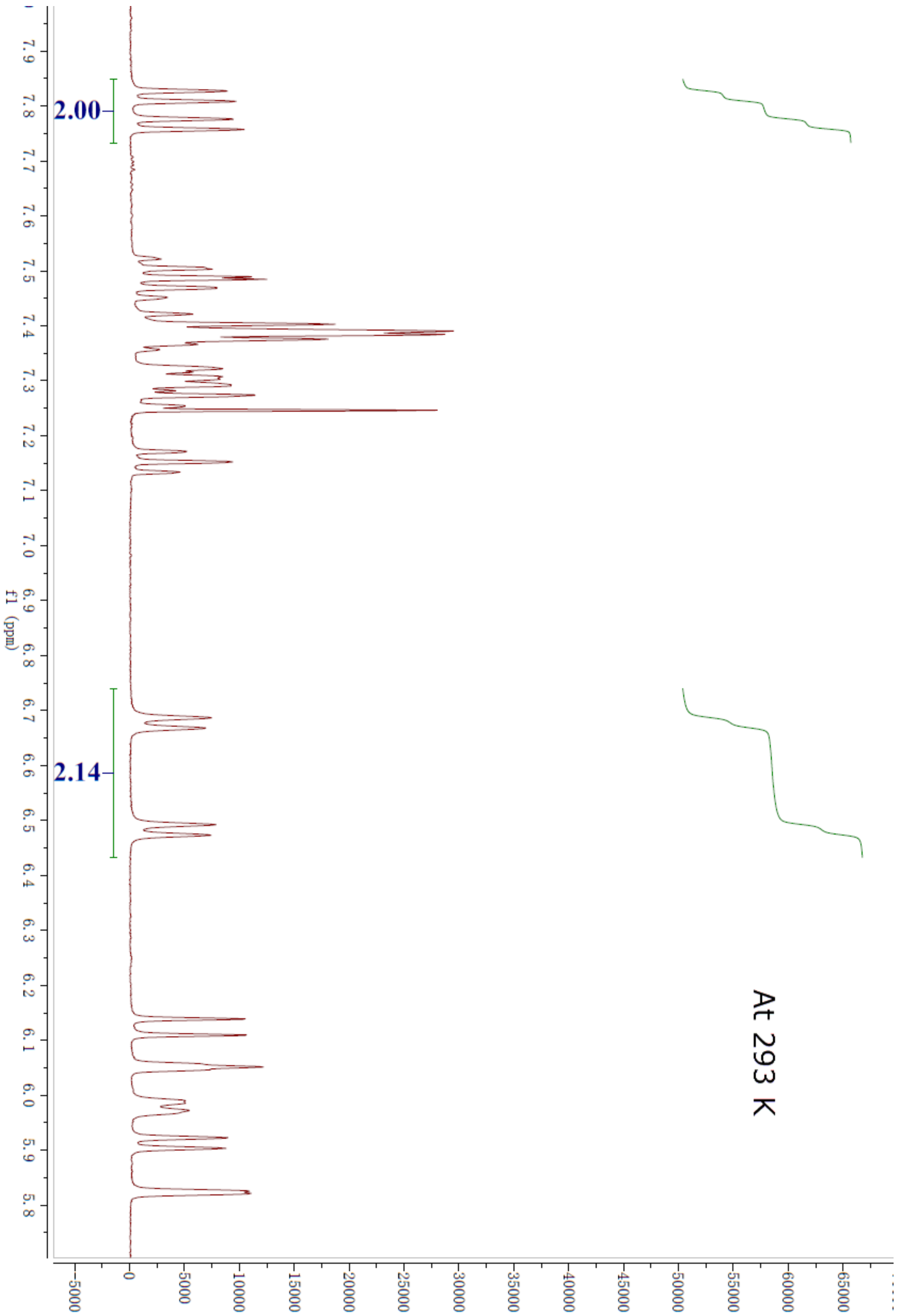

Figure S14. The integration of ^{1}H NMR signals located 6.6 and 7.8 ppm for **4** under different temperatures.

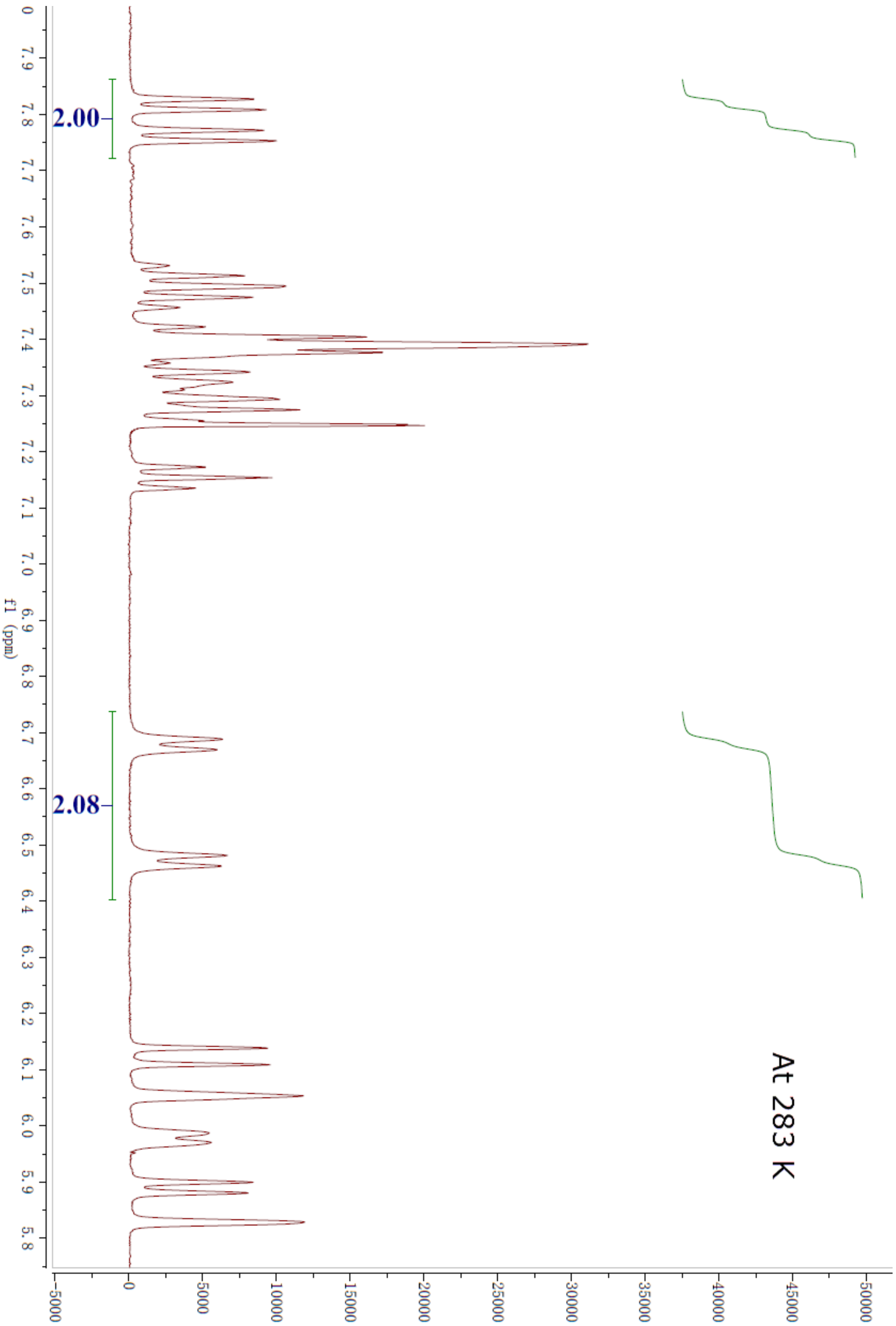

Figure S14. Continued.

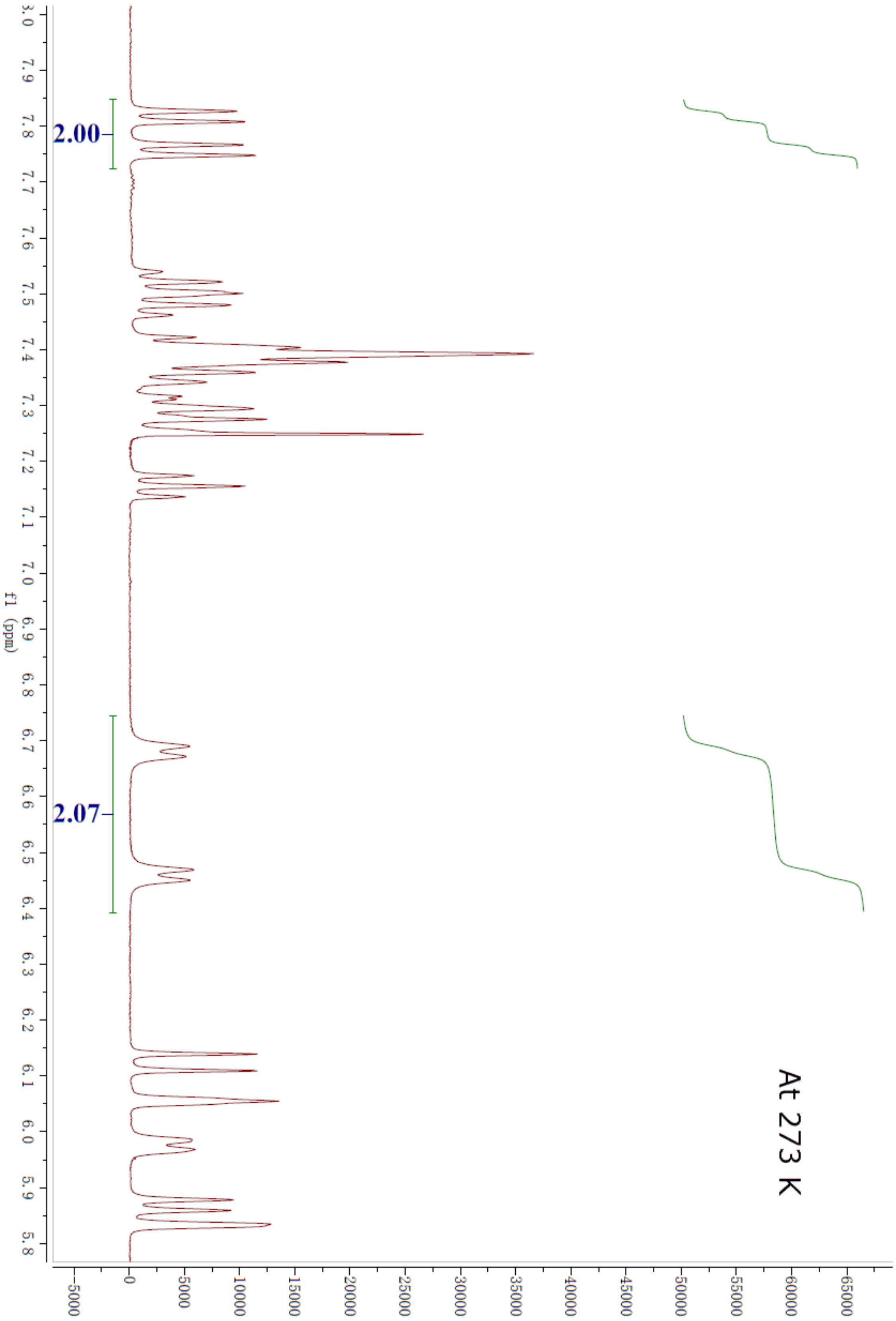

Figure S14. continued.

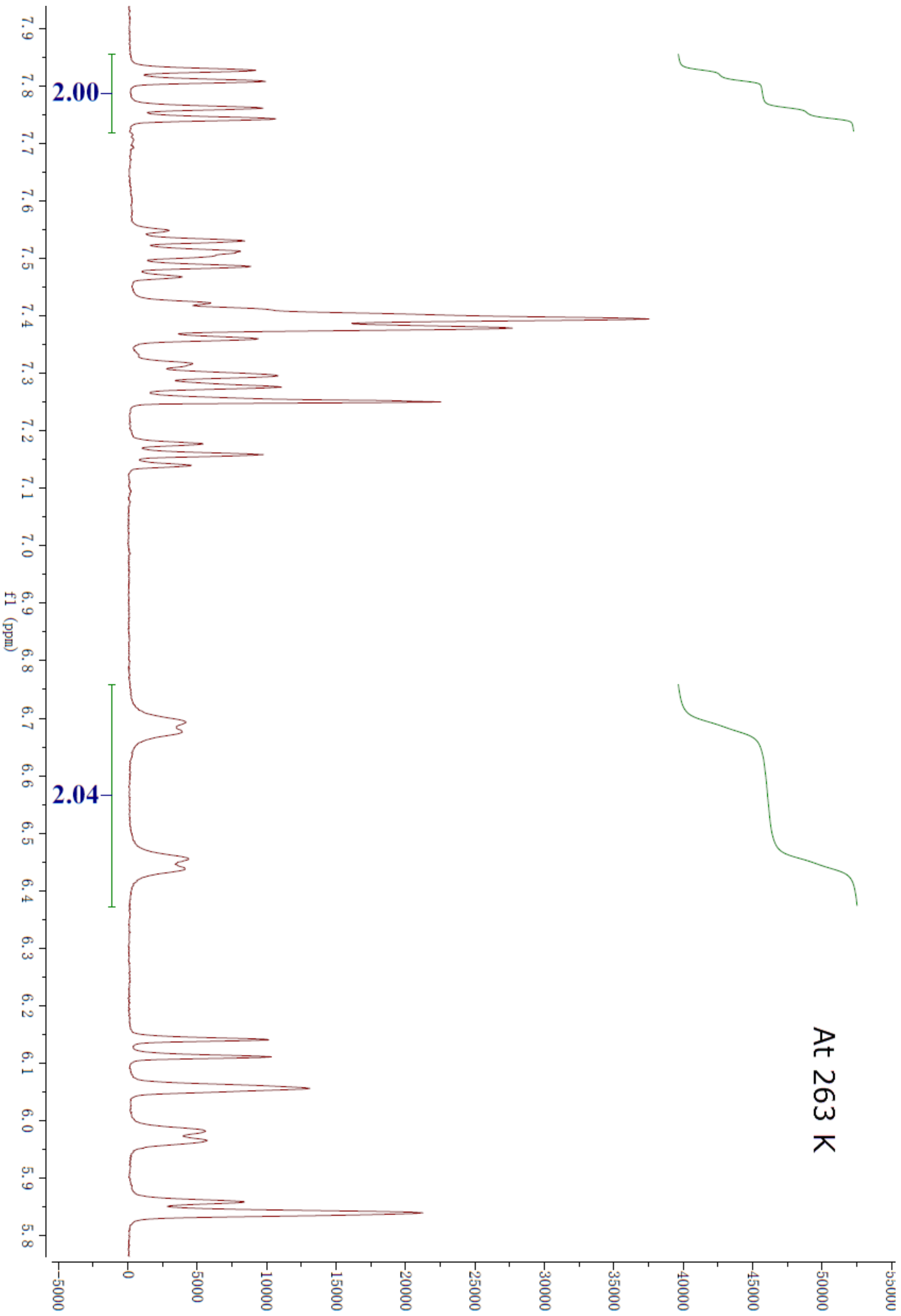

Figure S14**.** Continued.

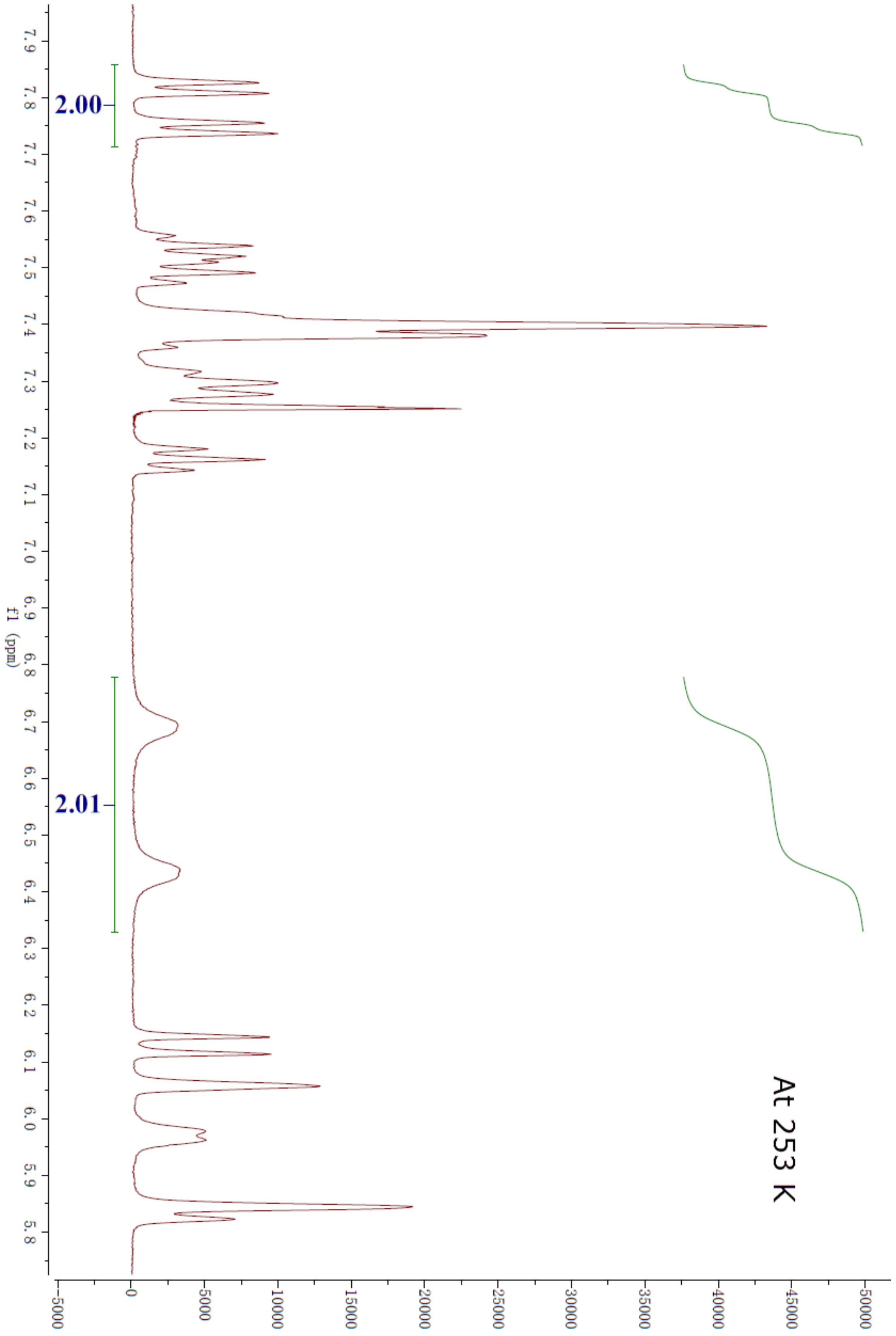

Figure S14. Continued.

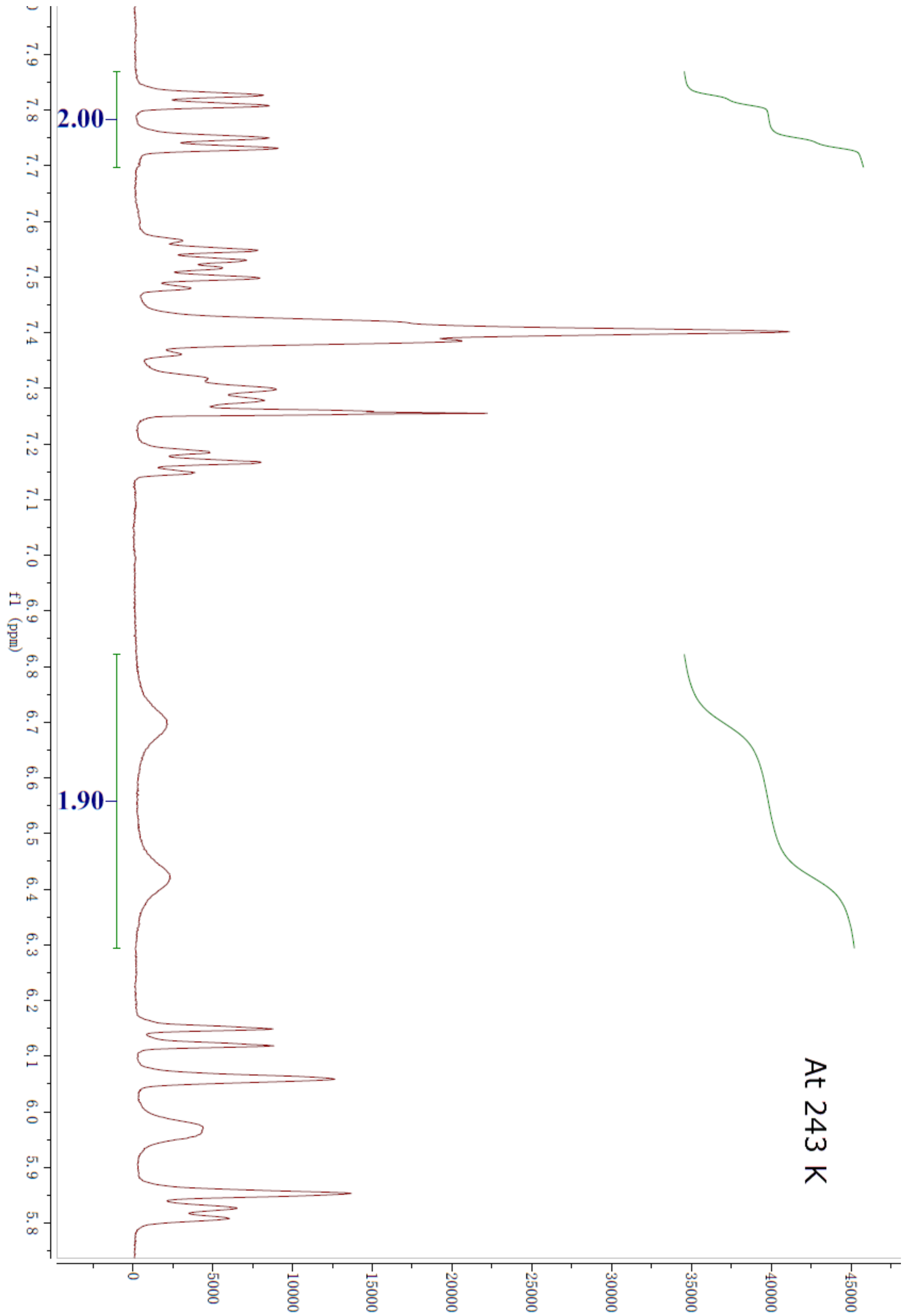

Figure S14. Continued.

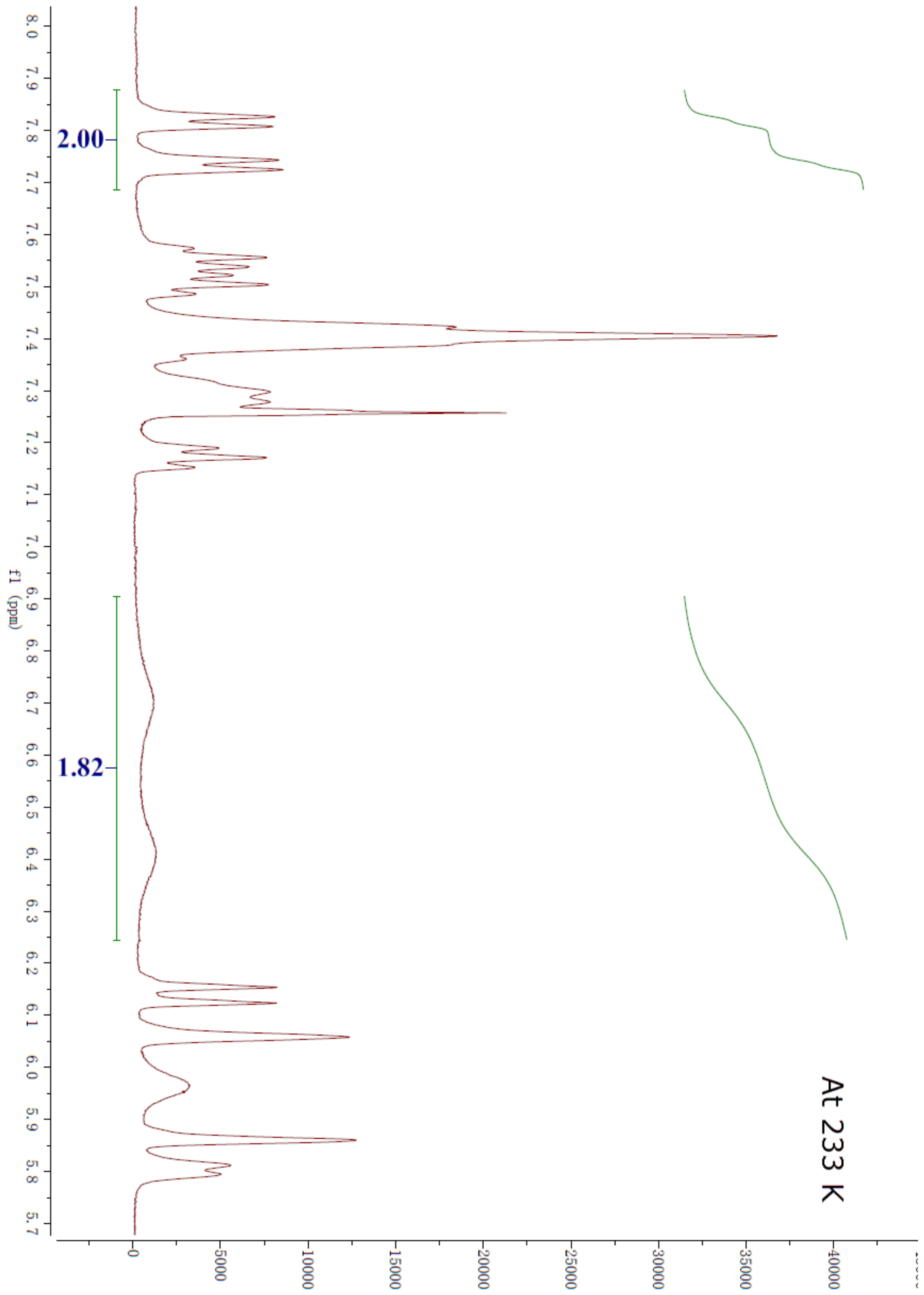

Figure S14. Continued.

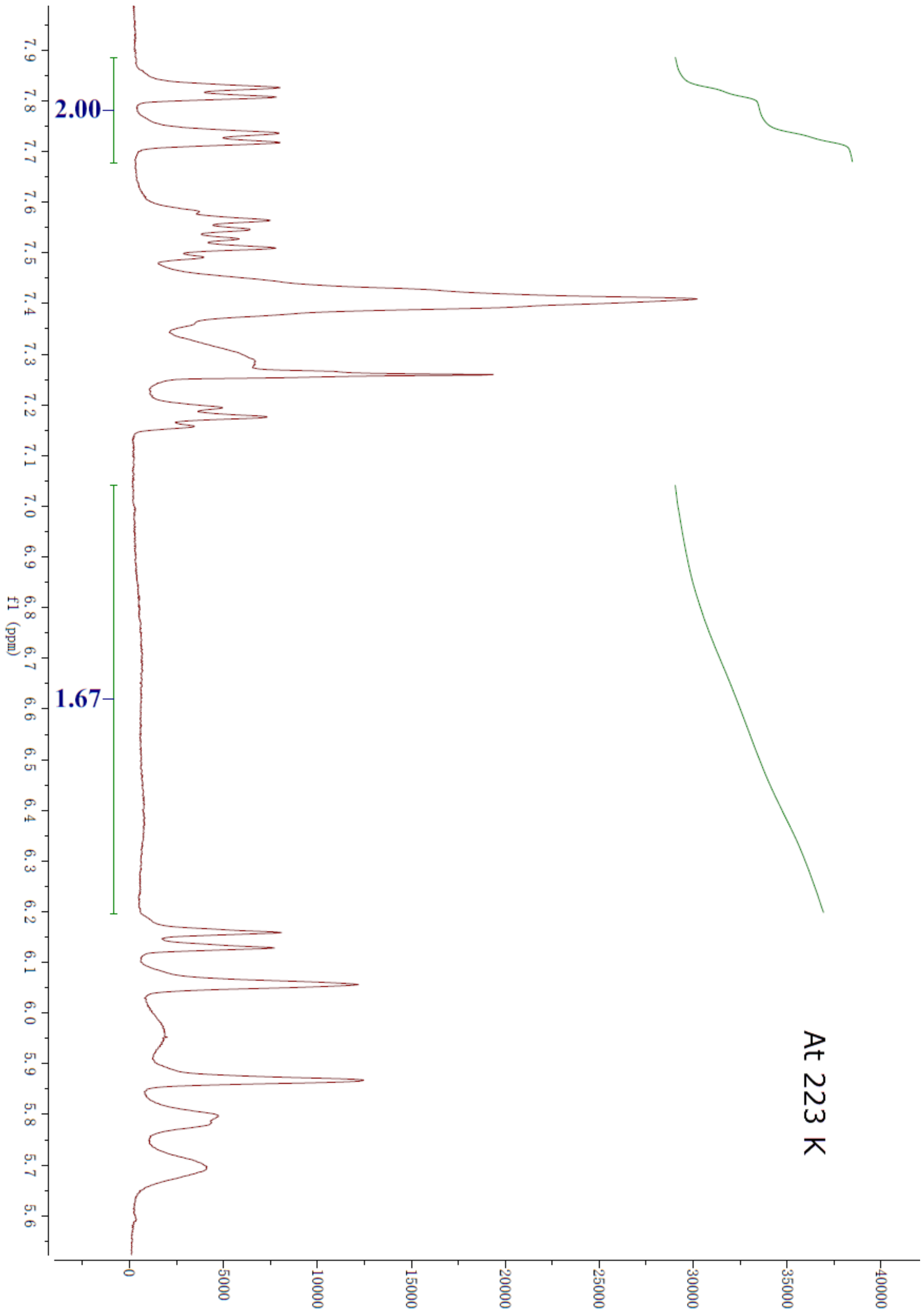

Figure S14. Continued.

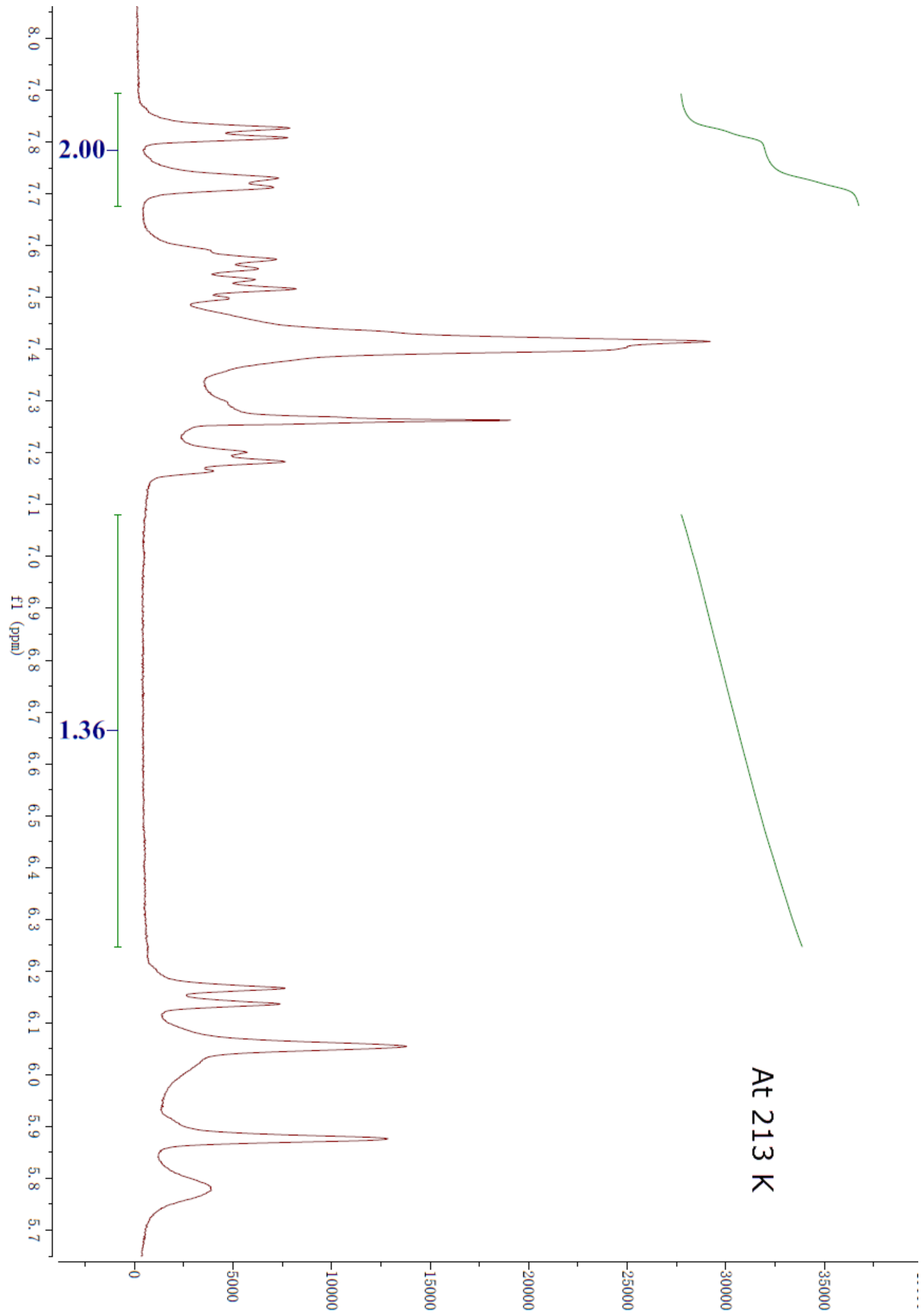

Figure S14. Continued.

Effect of concentration of compound 4 on ^1H NMR in $CDCl_3$

The ^1H NMR spectra were tested under different concentrations at 5.0, 1.67, 0.56, 0.19, 0.062, 0.021, 0.007 and 0.0023 mg/mL of **4** in $CDCl_3$. The corresponding scan numbers were 16, 16, 64, 192, 640, 8192, 20,480 and 40960 times, respectively.

5.0 mg/mL concentration. Original plot

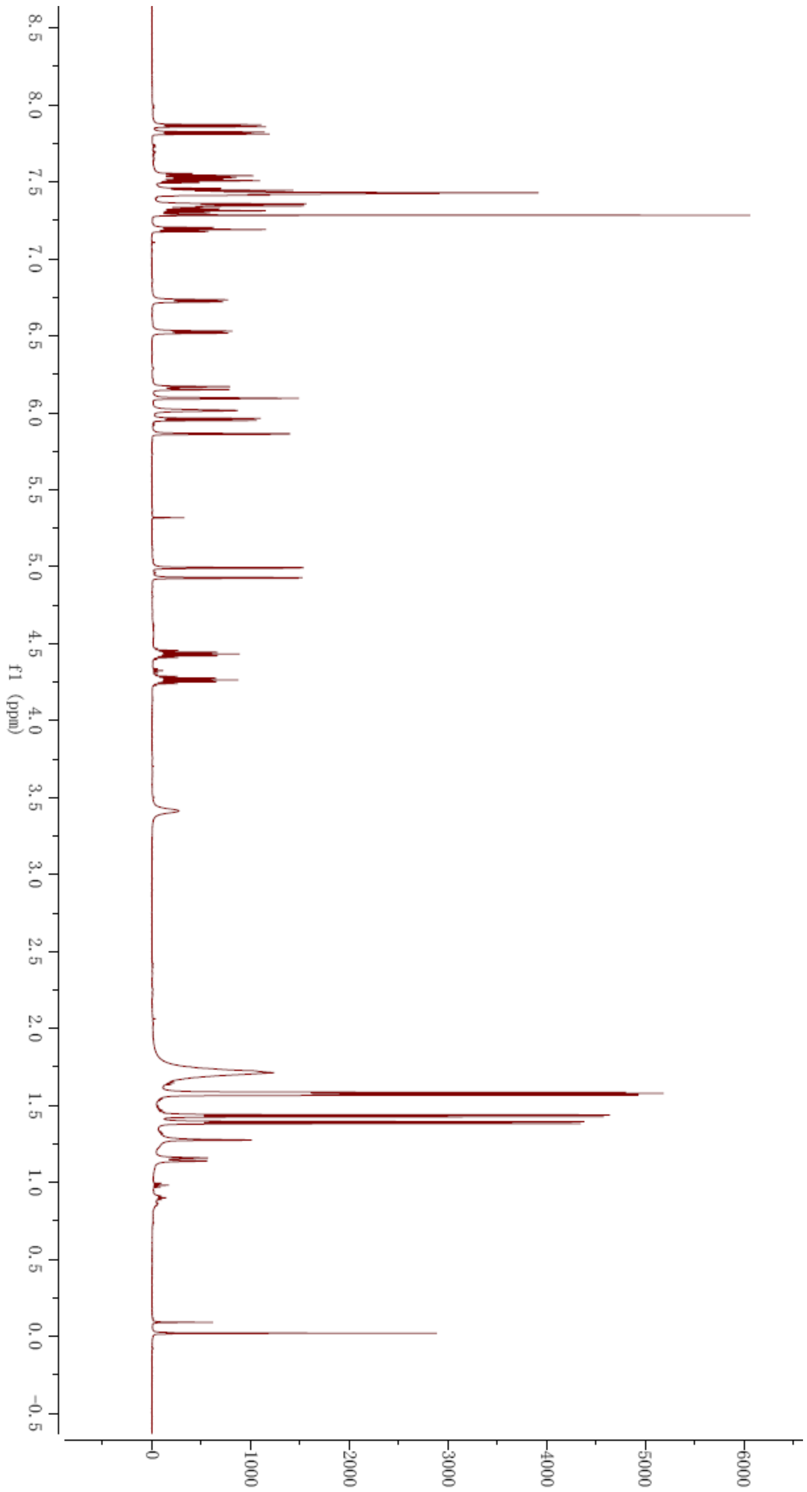

Figure S15. The effect of concentration of compound **4** on the ^1H NMR in $CDCl_3$.

1.67 mg/mL concentration. Original plot

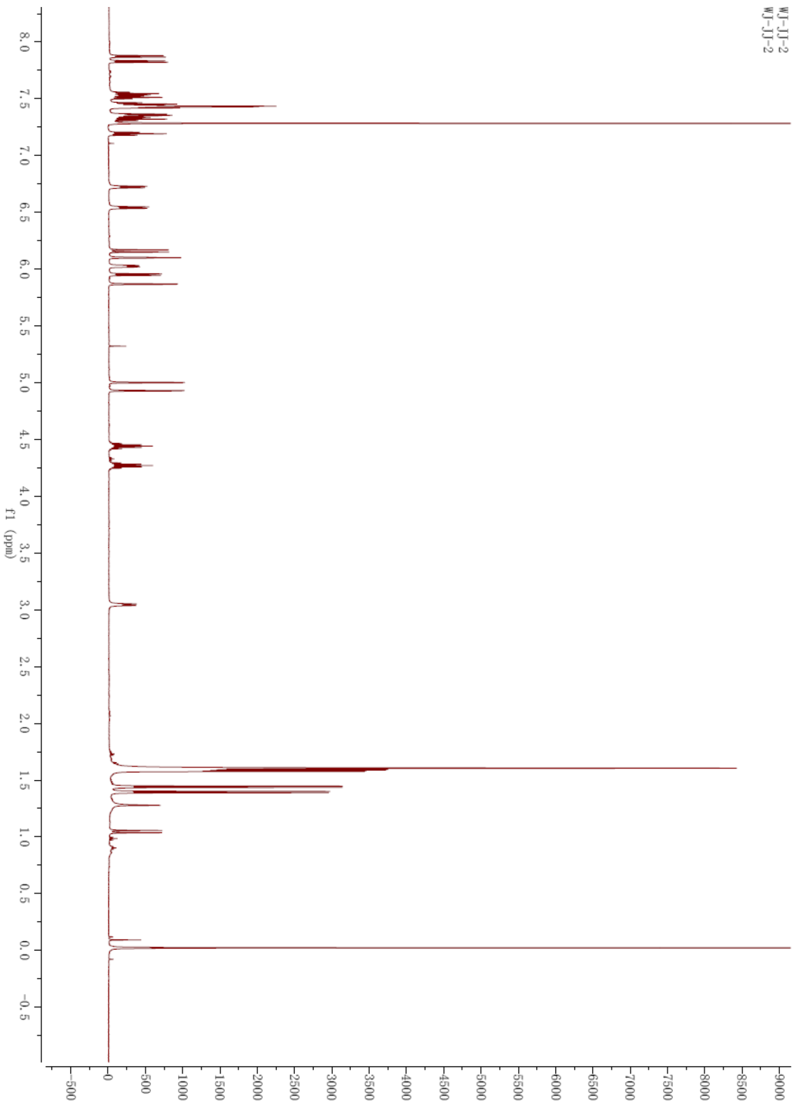

Figure S15. Continued.

0.56 mg/mL concentration. Original plot

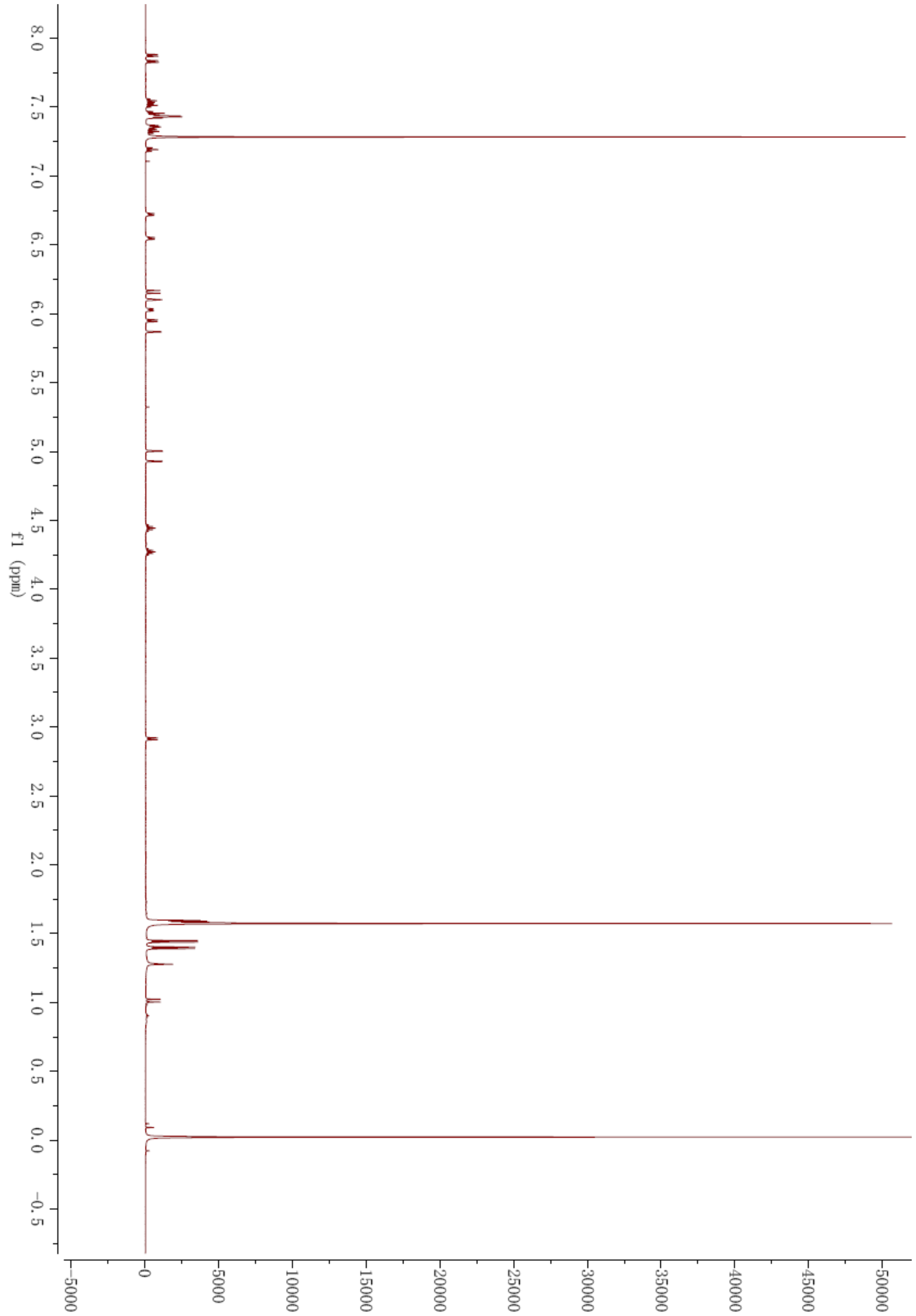

Figure S15. Continued.

Amplified plot for concentration of 0.56 mg/mL

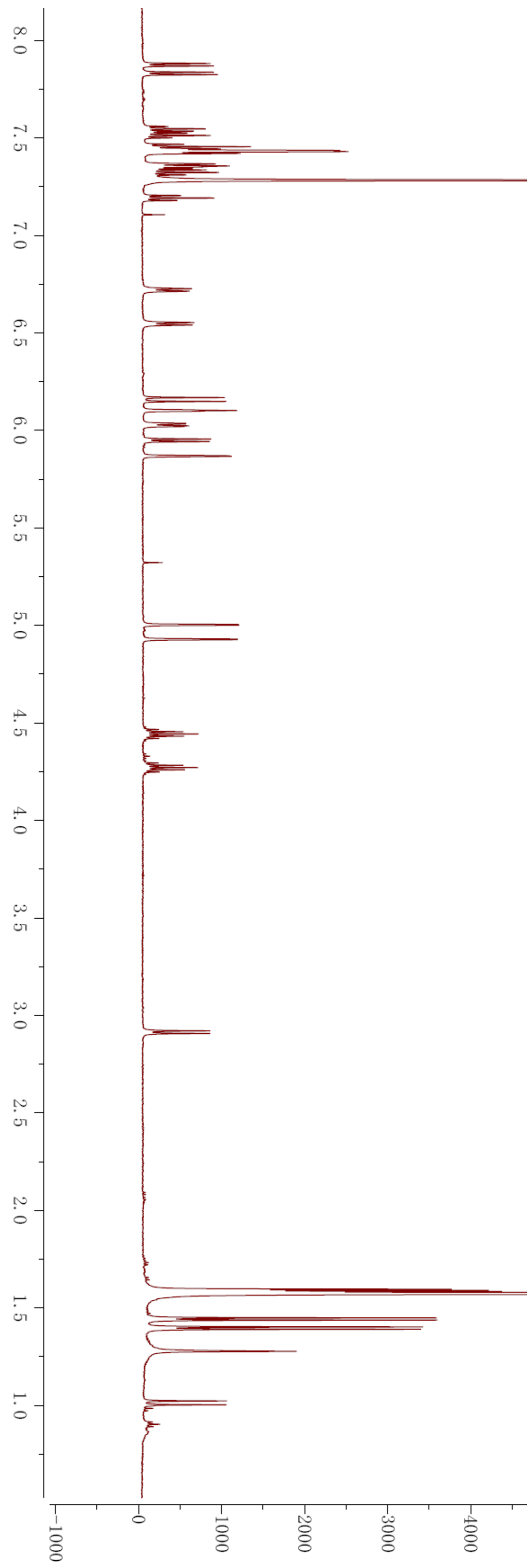

Figure S15. Continued.

0.19 mg/mL concentration. Original plot

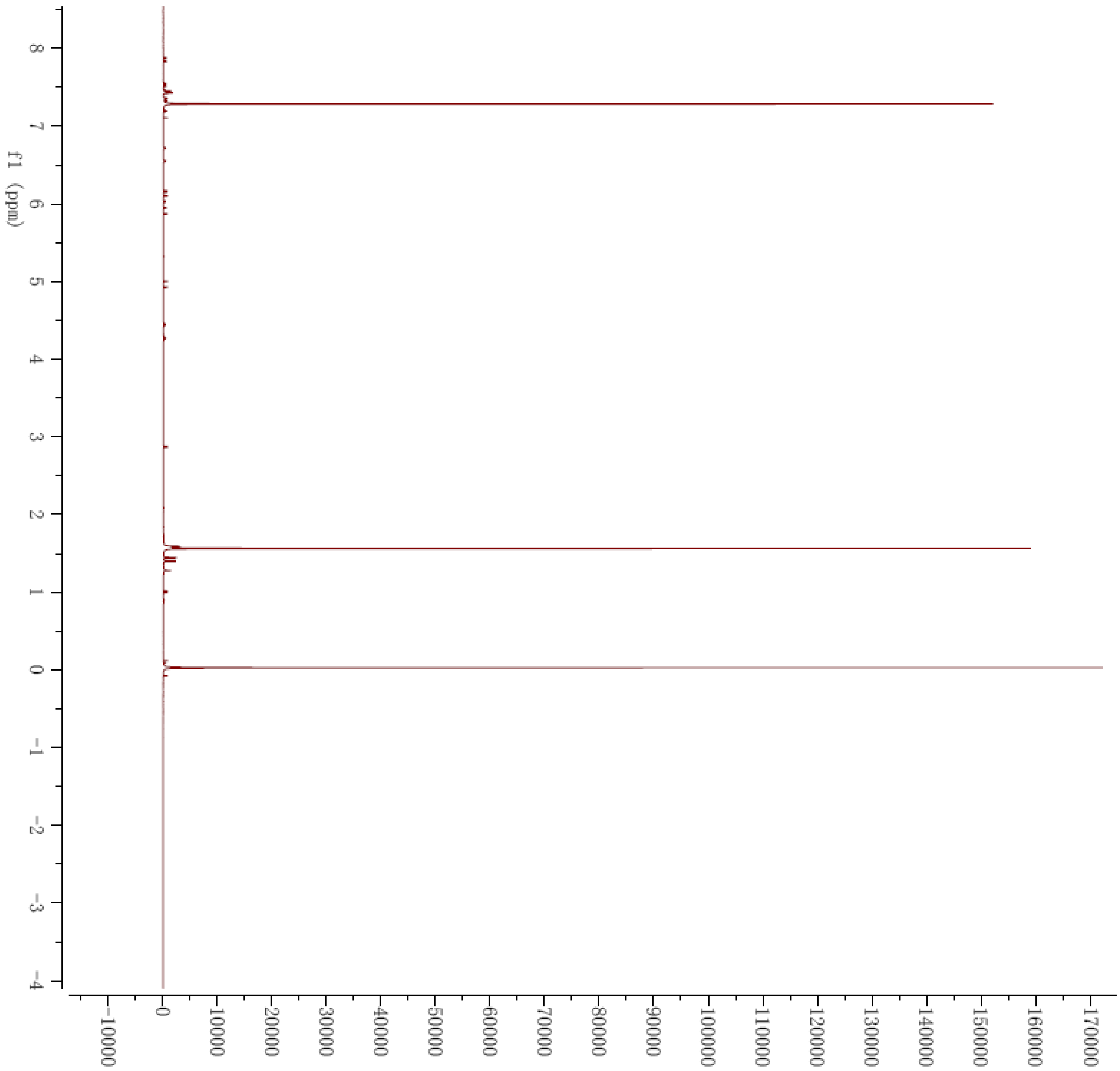

Figure S15. Continued.

Amplified

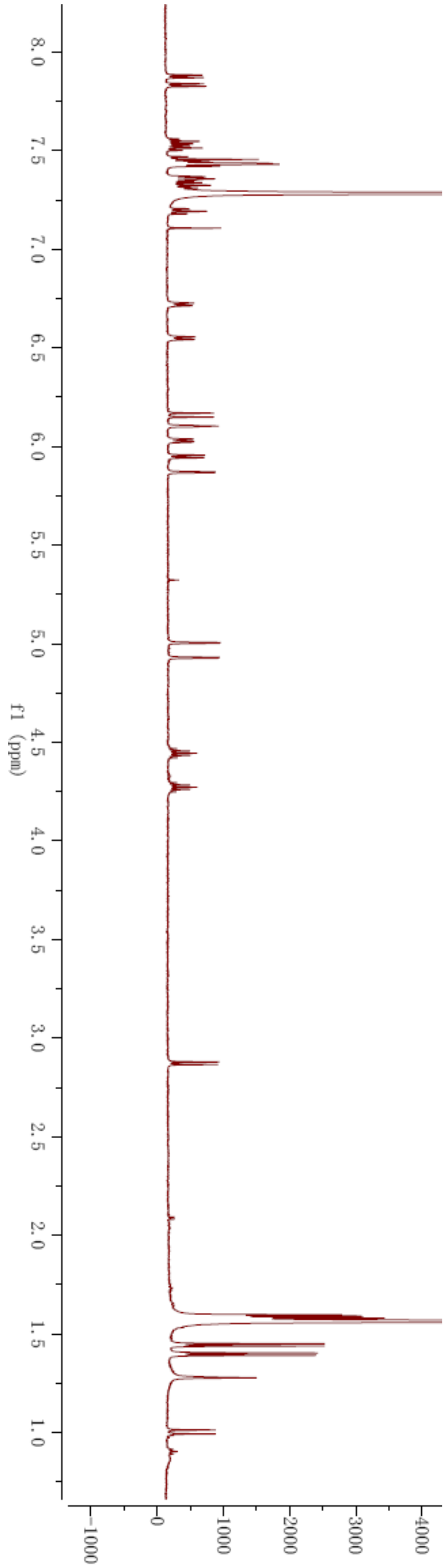

Figure S15. Continued

0.062 mg/mL concentration. Original plot

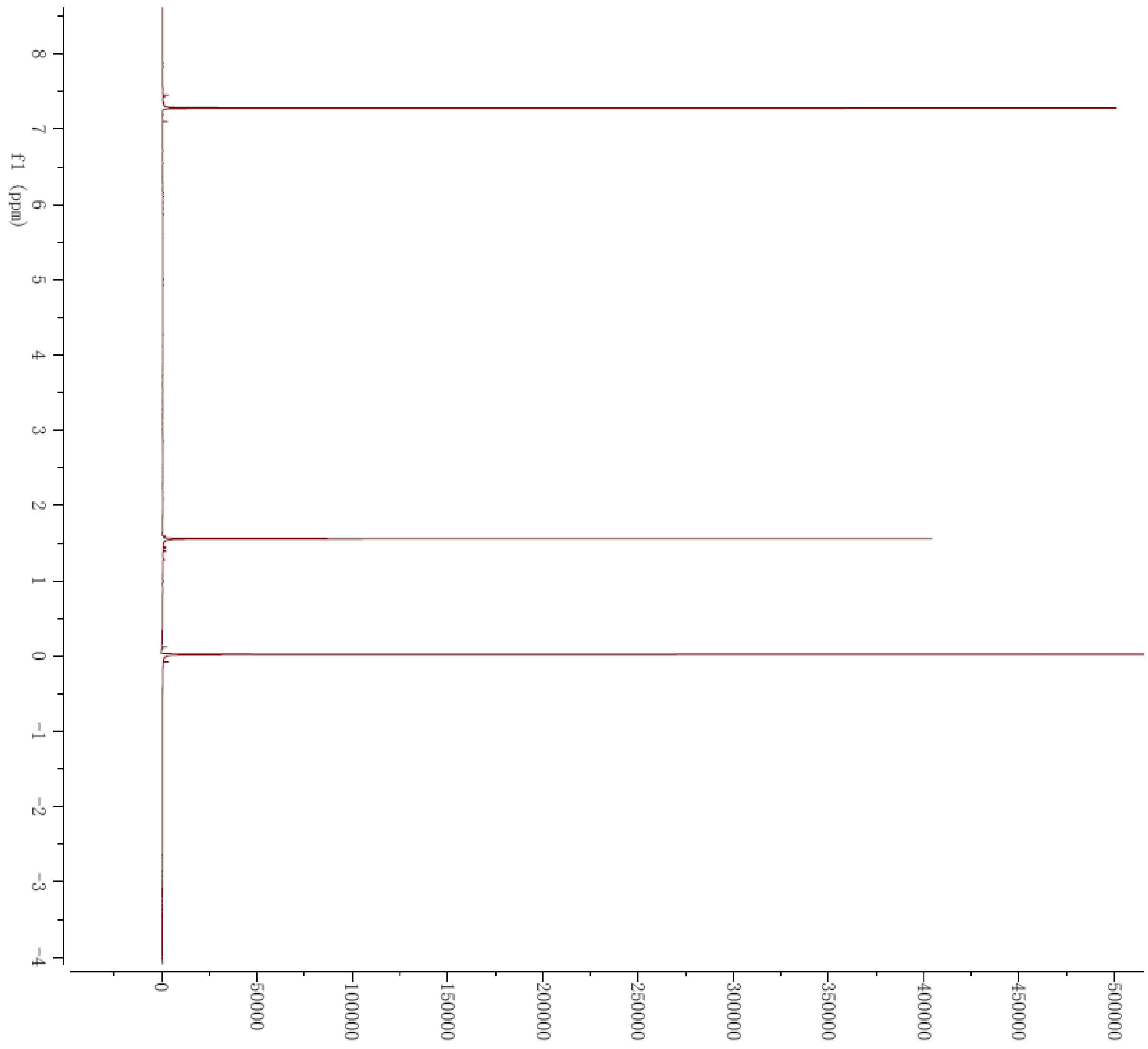

Figure S15. Continued.

Amplified

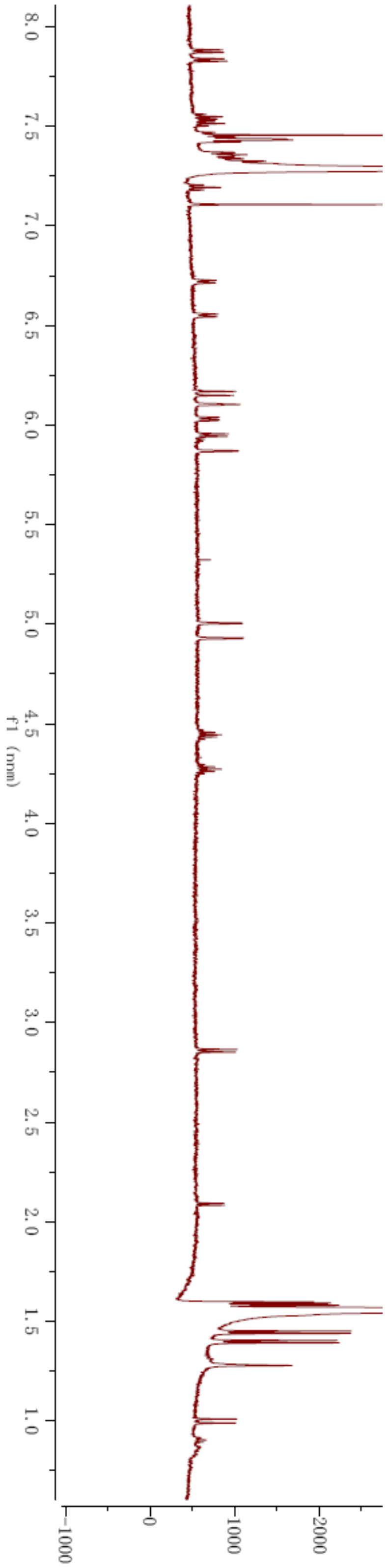

Figure S15. Continued

0.021 mg/mL concentration. Original plot

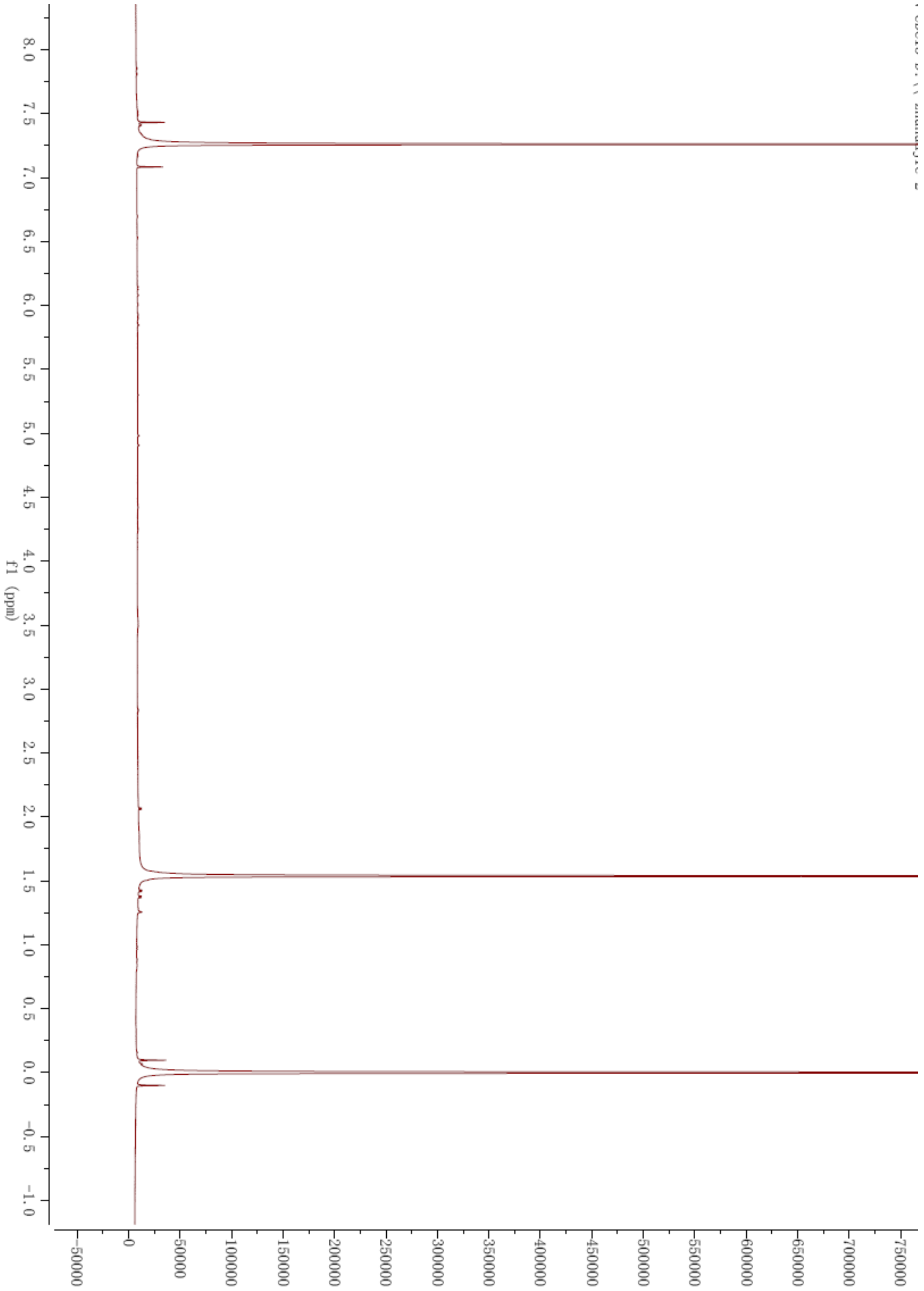

Figure S15. Continued

Amplified

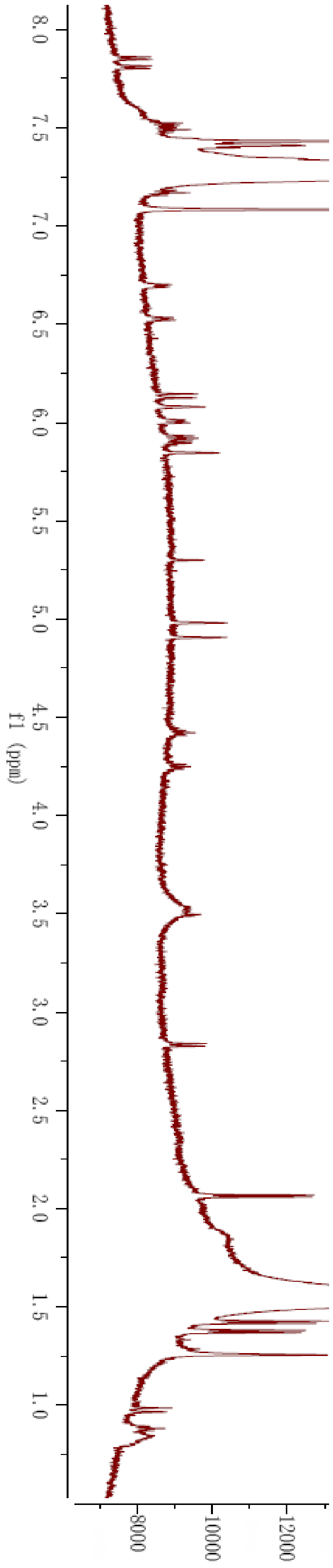

Figure S15. Continued

0.007 mg/mL concentration. Original

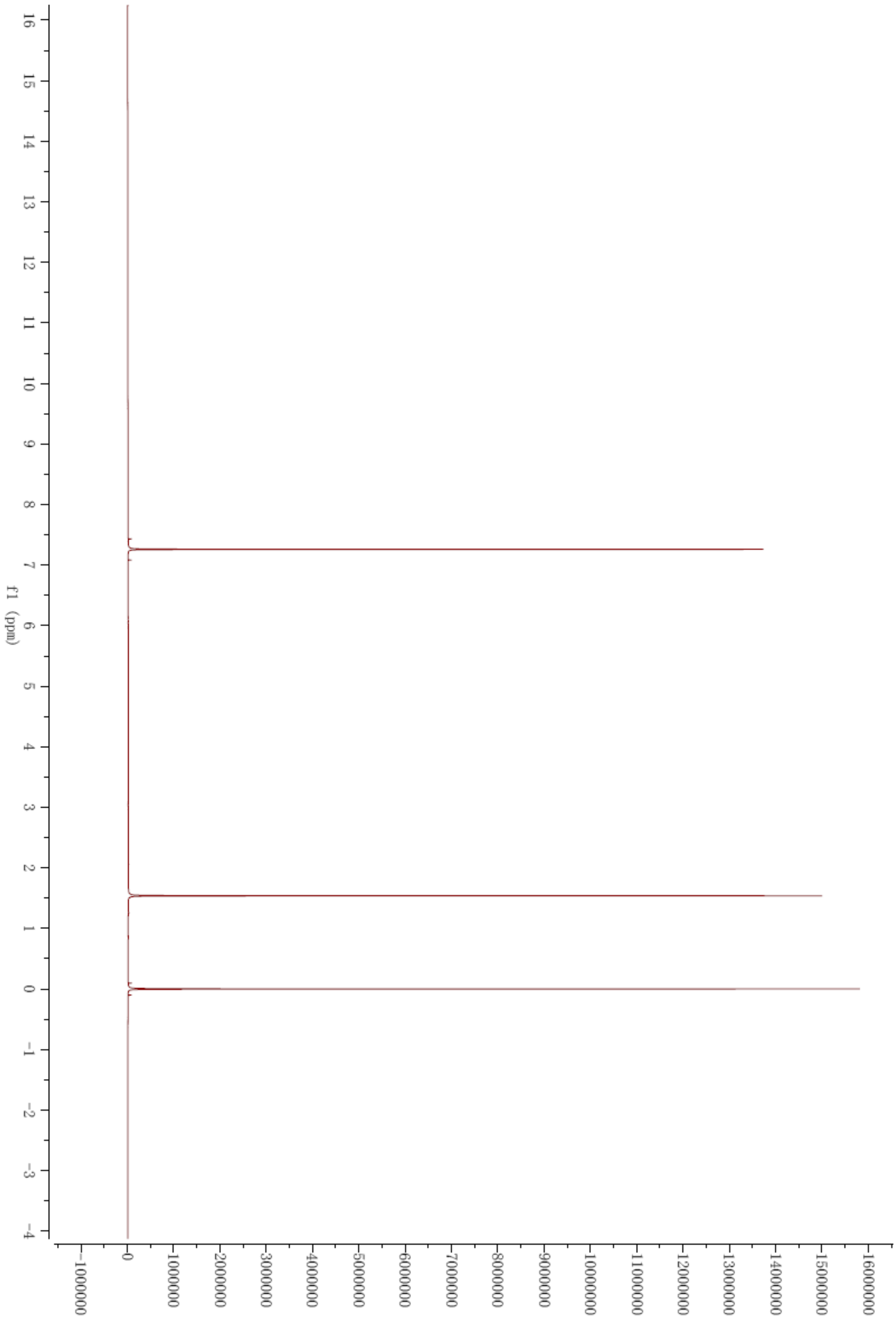

Figure S15. Continued

Amplified

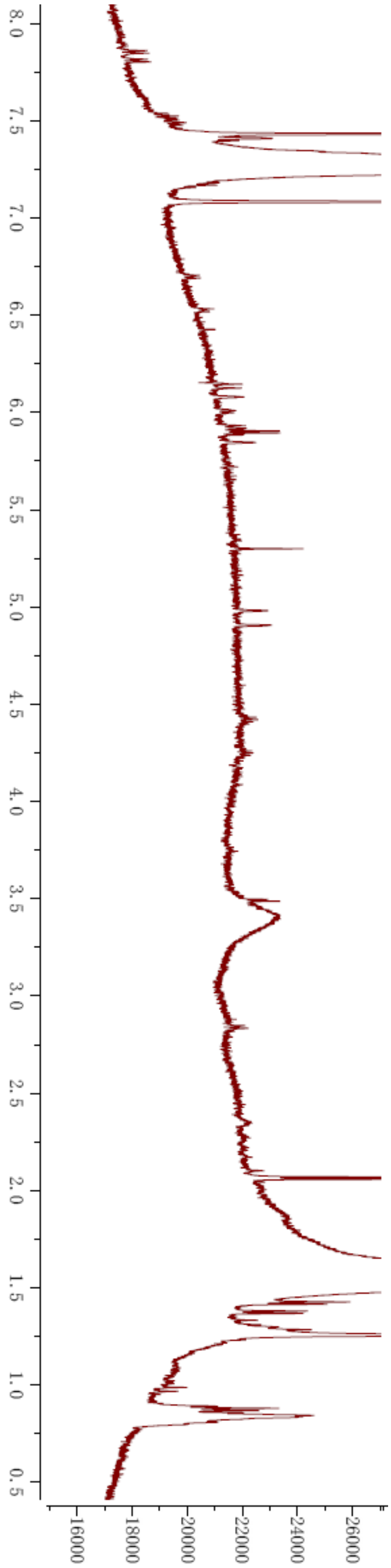

Figure S15. Continued.

0.0023 mg/mL concentration. Original plot:

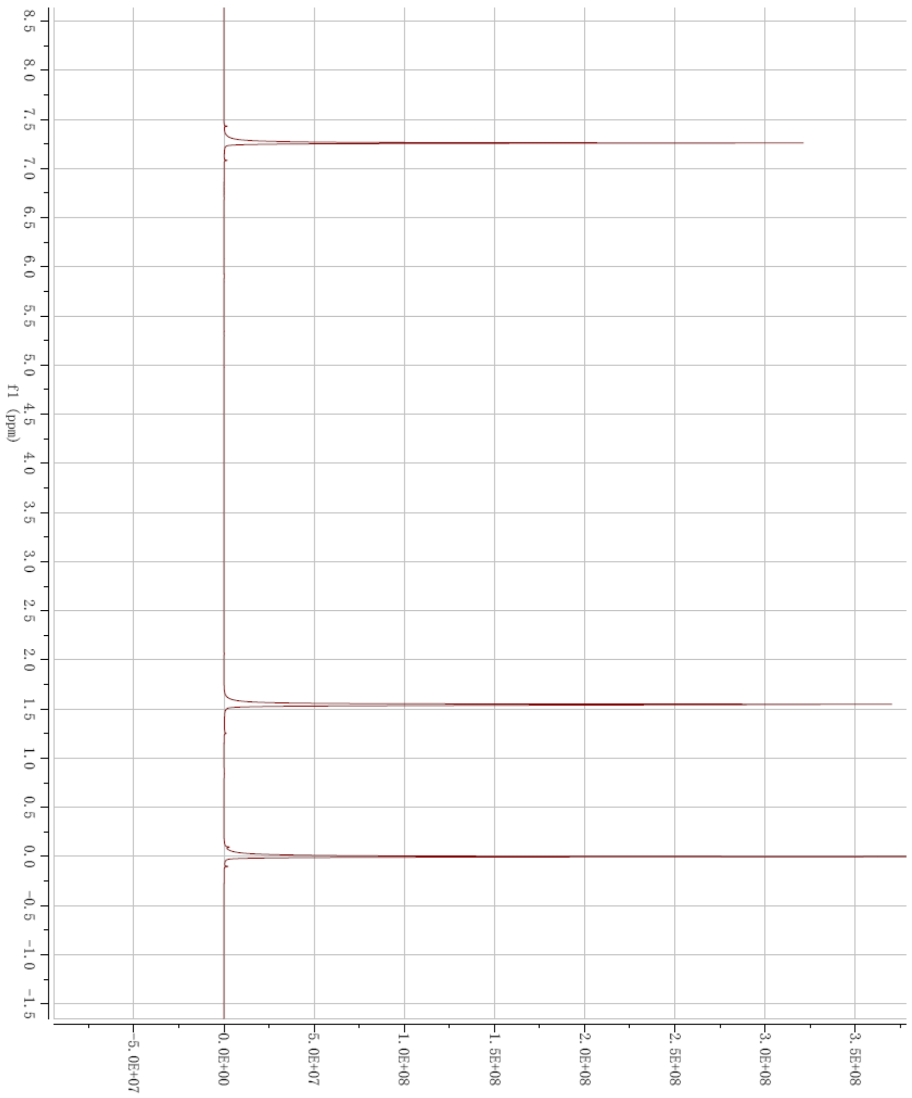

Figure S15. Continued.

Amplified:

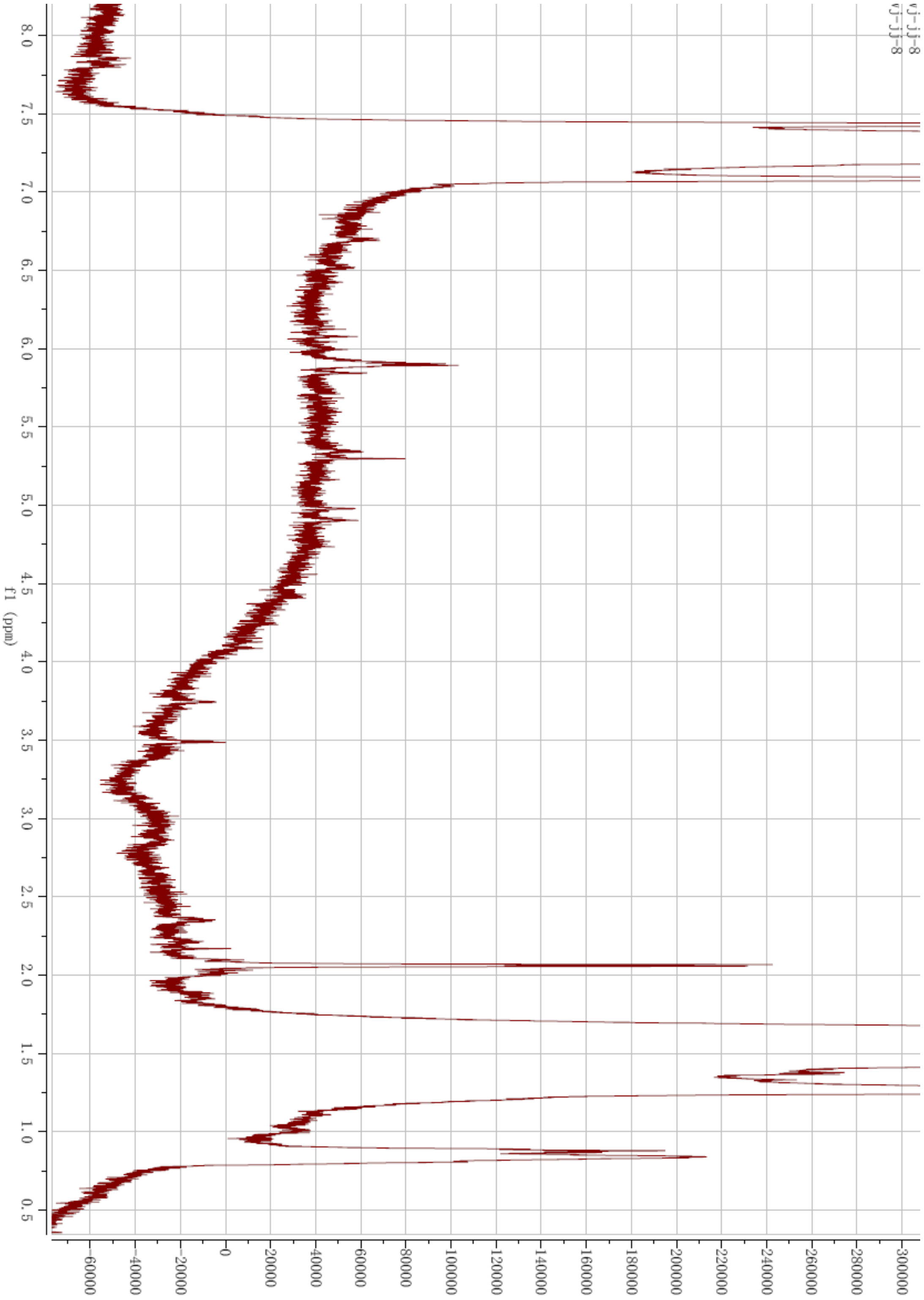

Figure S15. Continued.

NMR spectra for the oxidized product 5

The specific data for the oxidant product **5** are listed below, including ^{1}H NMR spectrum:

Molecular formula: $C_{19}H_{17}NO_3$. HRMS for $C_{19}H_{18}NO_3$ ($M+H^+$): 308.1287. Found: 308.1281. ^{1}H NMR (600MHz, $CDCl_3$, δ, ppm, J/Hz) δ_H: 7.83 (1H, d, J=7.2, H-2), 7.76 (1H, d, J=7.2, H-11), 7.49 (1H, t, J=7.2, H-4), 7.45 (2H, m, H-12, 13), 7.38 (1H, t, J=7.2, H-3), 6.82 (1H, d, J=7.8, H-5), 6.44 (1H, d, J=7.8, H-14), 5.99 (1H, d, J=1.8, H-9), 5.19 (1H, d, J=1.8, H-8), 4.04 (1H, m, H-17), 1.29 (3H, d, J=7.2, H-18), 1.23 (1H, d, J=7.2, H-19).

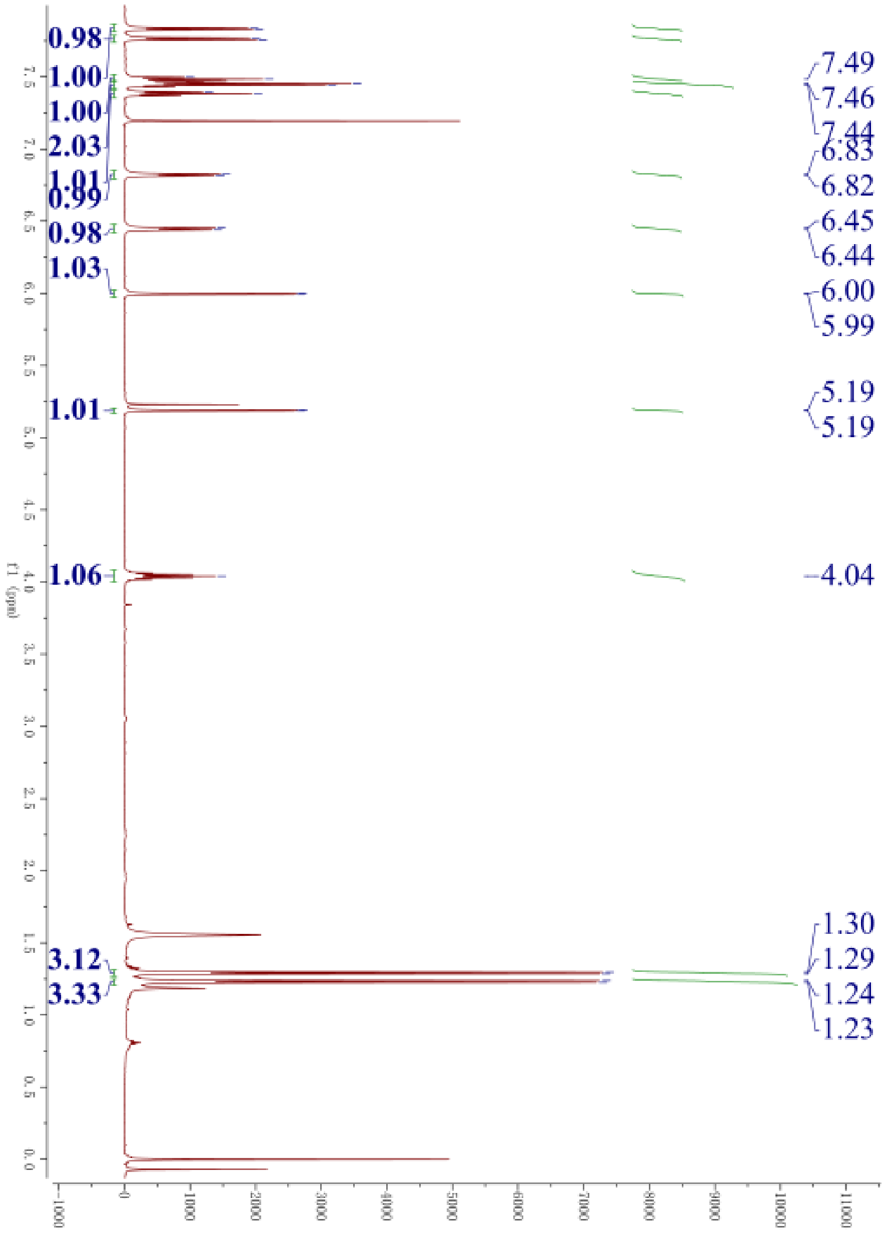

Figure S16. The ^{1}H NMR spectrum for the oxidant product **5.**

^{13}C NMR (151 MHz, $CDCl_3$) δ 169.33 (s), 168.79 (s), 144.85 (s), 139.36 (s), 134.41 (s), 134.24 (s), 131.50 (s), 130.17 (s), 129.49 (s), 127.14 (s), 126.05 (s), 123.98 (s), 122.10 (s), 122.04 (s), 81.08 (s), 61.96 (s), 47.36 (s), 20.36 (s), 20.01 (s).

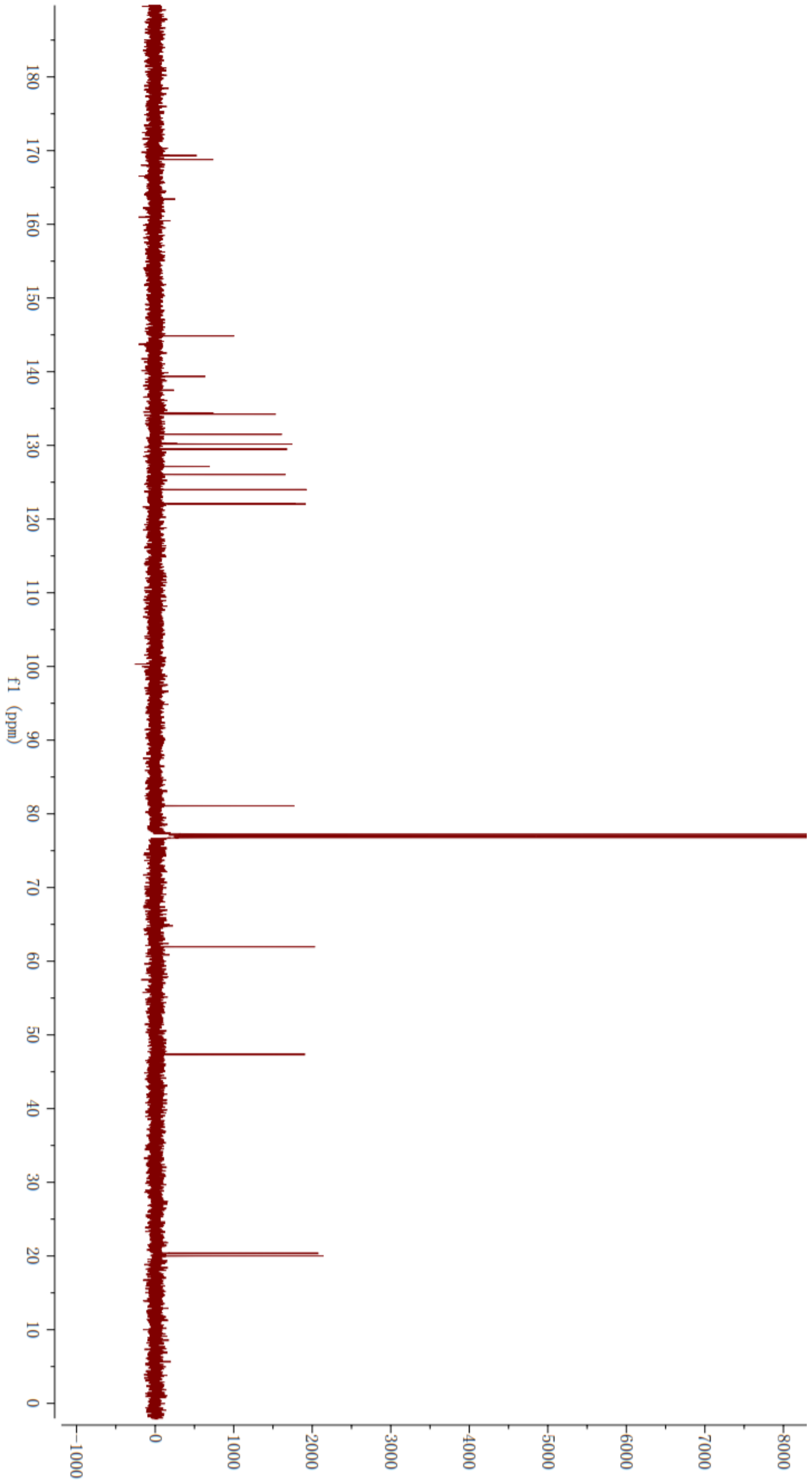

Figure S17. The ^{13}C NMR spectrum for the oxidant product **5**

The PES investigation of the lactone in the gas phase around the single bond of C3-C1'

The PES was performed for compound **5.** The largest barrier was about 11.0 kcal/mol at the B3LYP/6-311+G(d) level in the gas phase. Other two small barriers were about 6.26 can 4.58 kcal/mol.

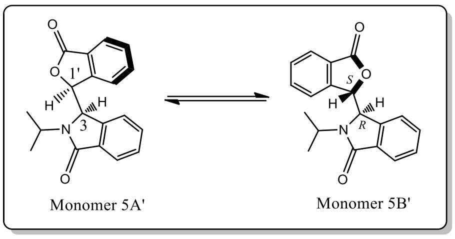

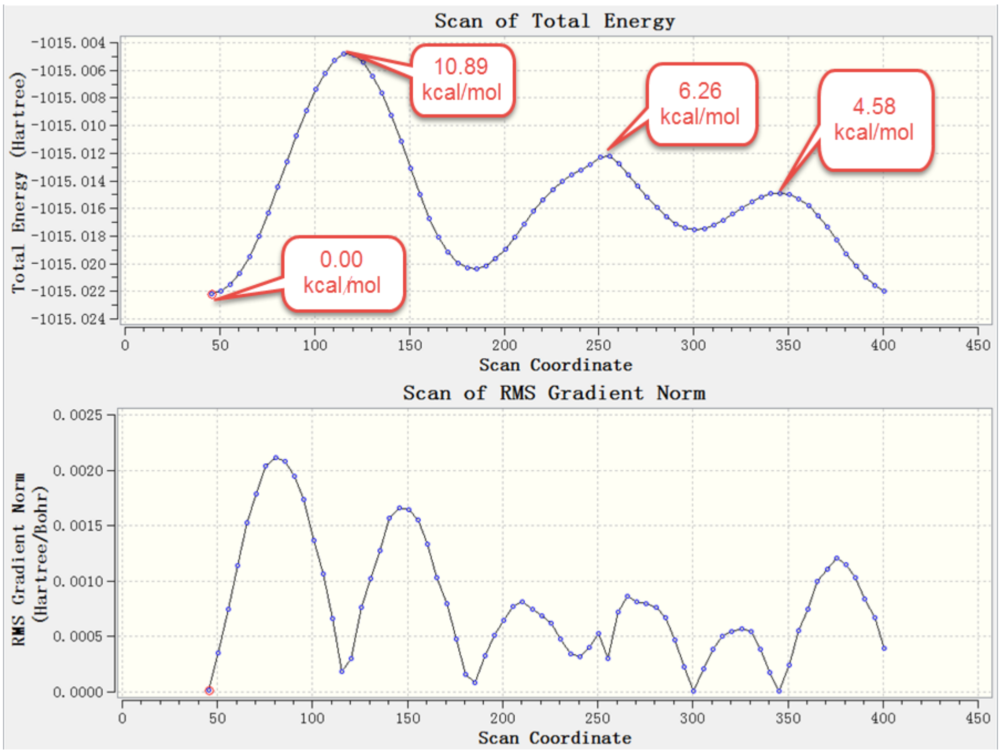

Figure S18. The PES investigation of the lactone in the gas phase around the single bond of C3-C1'.

NMR data of racemates **6-14** in CD_3OD and $CDCl_3$.

Compound **6**

2-ethyl-3-(3-hydroxy-1,3-dihydroisobenzofuran-1-yl)isoindolin-1-one

Indeed, another bond length conformer still existed in a small ratio in CD_3OD. However, most of the signals exhibited normal. HRMS for $C_{18}H_{18}NO_3$ [M+H+]: 296.1287, found: 296.1280. ^1H NMR (600 MHz, CD_3OD) δ 7.88 (dd, J = 12.9, 7.6 Hz, 1H), 7.57 (m, 1H), 7.47 – 7.43 (m, 1H), 7.35-7.28 (m, 4H), 6.75 (d, J = 7.4 Hz, 1H), 6.62 (d, J = 7.5 Hz, 1H), 6.11 (s, 1H), 5.90 (s, 1H), 5.23 (d, J = 15.4 Hz, 1H), 4.51 (d, J = 15.3 Hz, 1H), 1.32 (s, 3H).

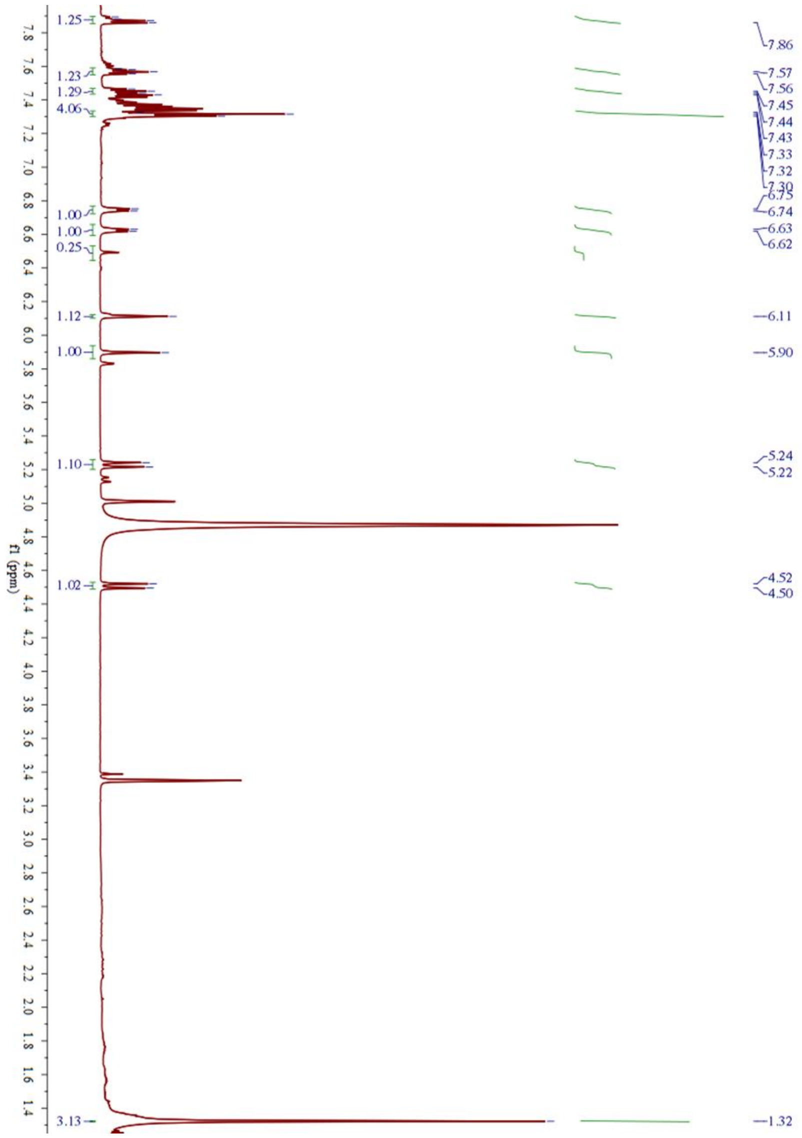

Figure S19. ^1H NMR for compound **6** in CD_3OD. The signal strength ratio is almost 1:0.22 between the two sets of ^1H NMR spectra.

^{13}C NMR (151 MHz, CD_3OD) δ 171.29, 142.98, 142.19, 139.06, 132.84, 130.26, 130.06, 129.78, 124.40, 124.18, 122.32, 102.22, 82.60, 64.29, 45.96, 30.70, 23.69.

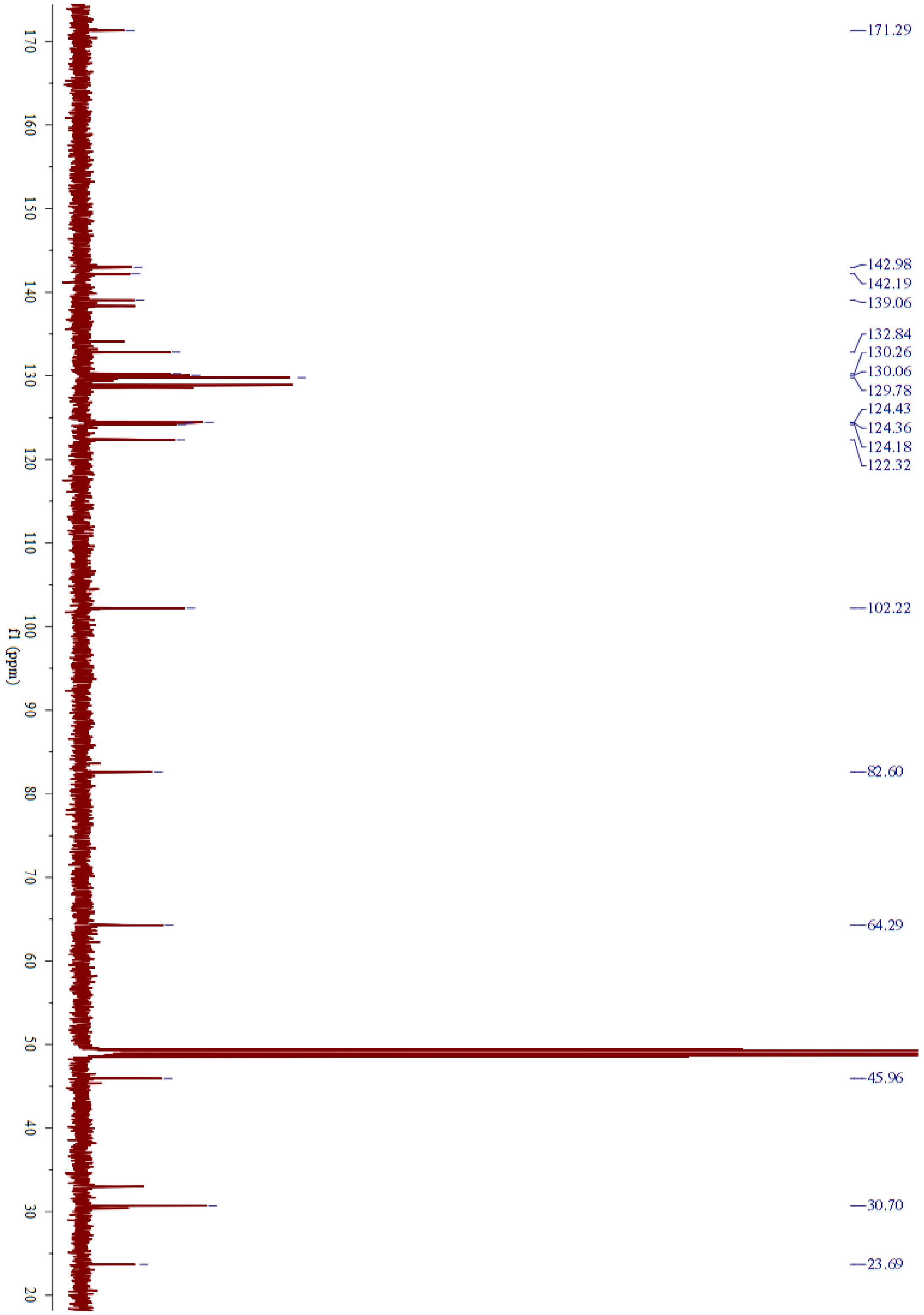

Figure S20 ^{13}C NMR for compound **6** in CD_3OD

2-ethyl-3-(3-hydroxy-1,3-dihydroisobenzofuran-1-yl)isoindolin-1-one. One more set of ^{1}H NMR with about a ratio of 0.3:1 were recorded in $CDCl_3$. The numbers of the proton in the minor contributed conformer are not listed.

^{1}H NMR (600 MHz, CD_3OD) δ 7.89 (d, *J* = 7.5 Hz), 7.87 (d, *J* = 7.6 Hz, 1H), 7.59 (m, 2H), 7.52 (t, *J* = 7.4 Hz, 1H), 7.49 – 7.41 (m, 3H), 7.36 – 7.32 (m), 7.07 (d, *J* = 7.5 Hz), 6.91 (d, *J* = 7.6 Hz, 1H), 6.71 (d, *J* = 7.6 Hz, 1H), 6.58 (d, *J* = 7.6 Hz), 6.47 (s), 6.18 (s, 1H), 6.01 (d, *J* = 2.1 Hz, 1H), 5.91 (d, *J* = 2.1 Hz), 5.30 (d, *J* = 2.3 Hz), 5.23 (d, *J* = 2.1 Hz, 1H), 4.07 – 4.01 (m, 1H), 3.99 (dd, *J* = 12.6, 5.6 Hz, 1H), 3.77 – 3.70 (m), 3.51 (dq, *J* = 14.2, 7.1 Hz, 1H), 1.39 (t, *J* = 7.2 Hz, 3H), 1.36 (t, *J* = 7.2 Hz, 1H).

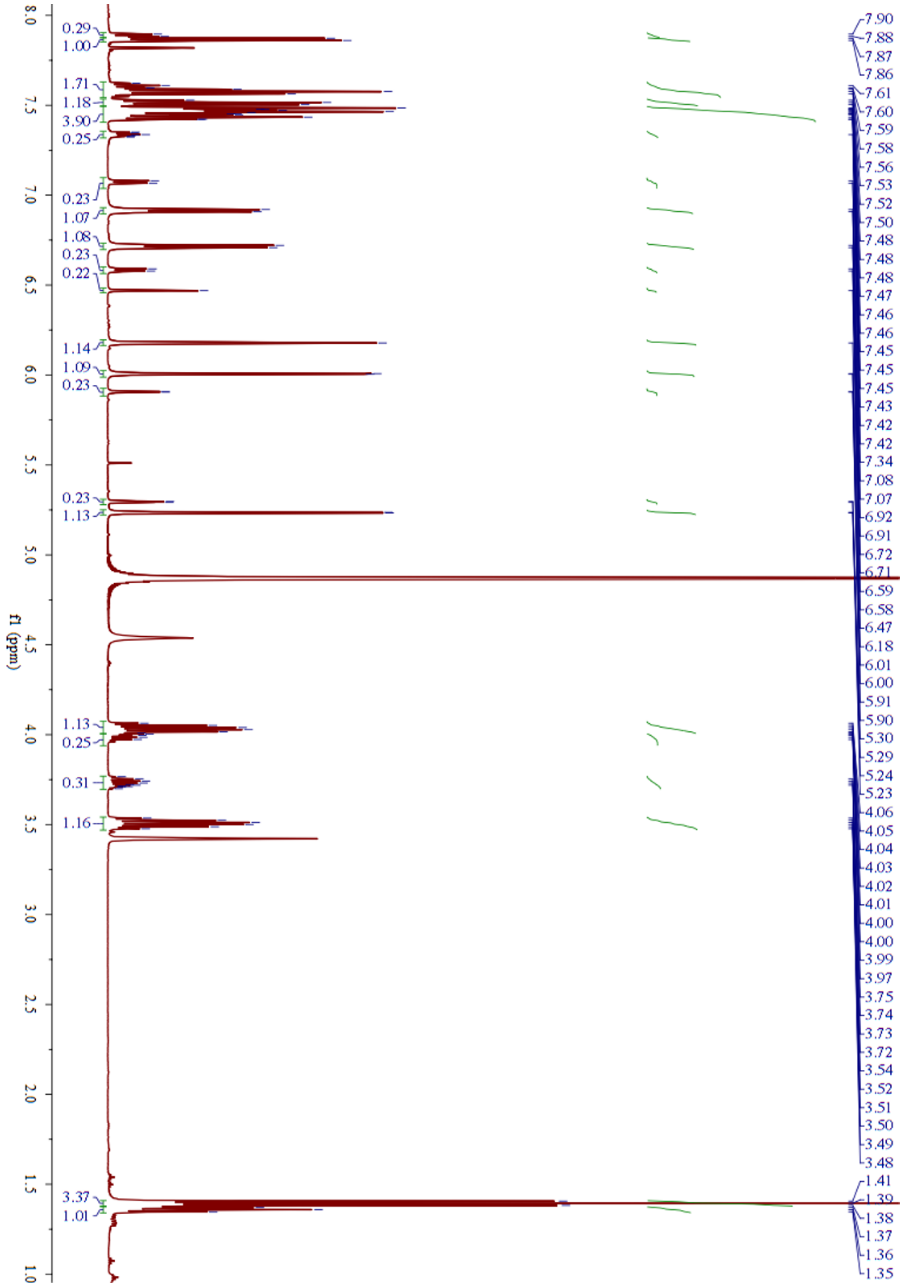

Figure S21 ^{1}H NMR for compound **6** in $CDCl_3$. The signal strength ratio is almost 1:0.20 between the two sets of ^{1}H NMR. The shift of proton of –OH is 4.57 ppm (minor CD_3OD was added to increase its solubility).

The single shift at 1.32 in CD_3OD became two signals at 1.38 and 1.40 ppm in $CDCl_3$. The signals at 1.35 and 1.40 ppm became multiple signals. Other signals may overlap.

Some signals overlapped.

^{13}C NMR (151 MHz, CD_3OD) δ 170.64, 170.44, 142.62, 142.28, 141.57, 141.34, 138.72, 138.30, 134.11, 132.39, 132.18 , 130.10, 129.88, 129.85, 129.65, 129.59, 129.54, 124.13, 124.09, 123.98, 123.88, 123.82, 123.62, 122.14, 122.09, 101.89, 101.54, 82.59, 82.12, 63.90, 63.50, 36.75, 13.57, 13.44.

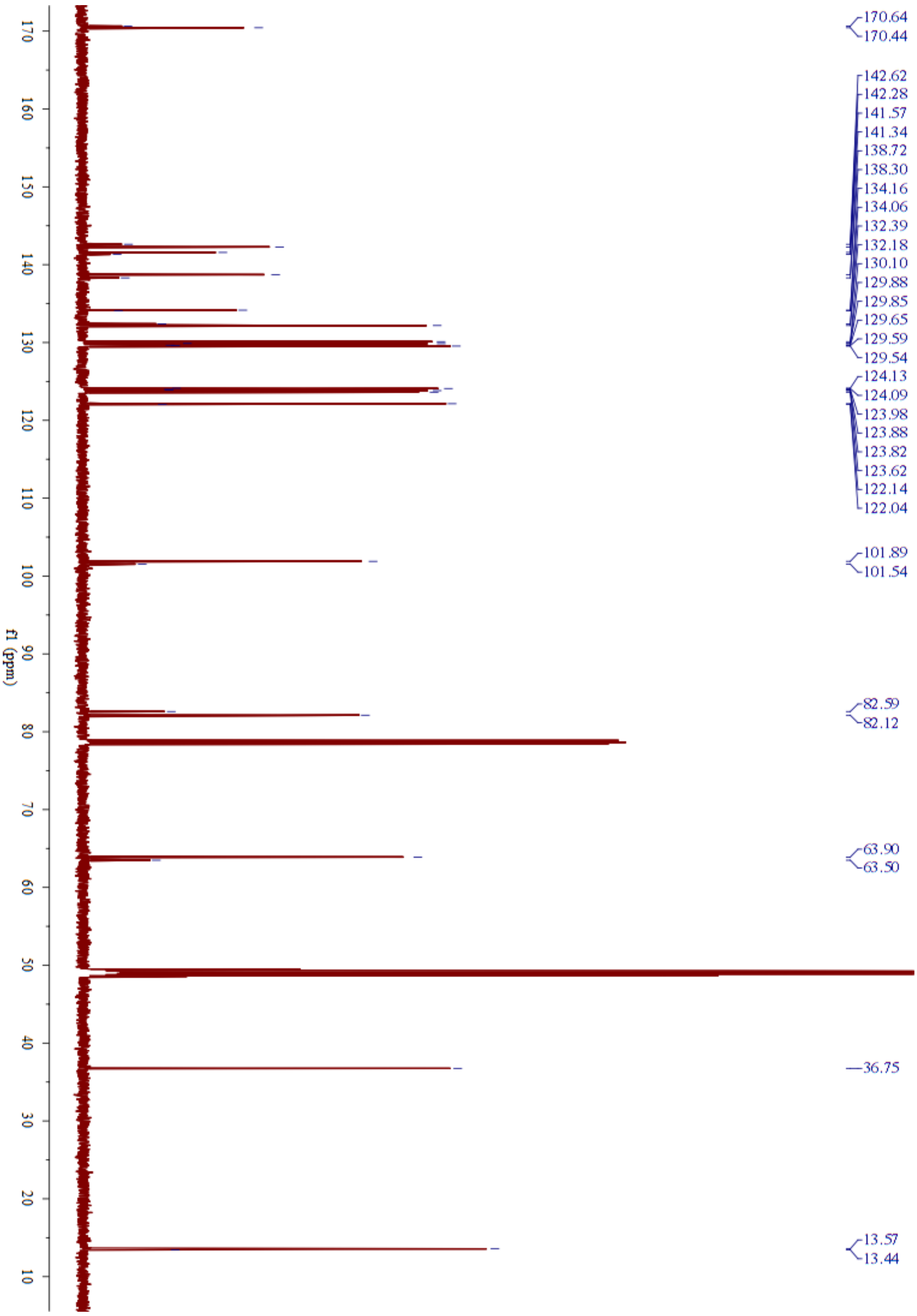

Figure S22 ^{13}C NMR for compound **6** in $CDCl_3$

Compound 7

3-(3-hydroxy-1,3-dihydroisobenzofuran-1-yl)-2-propylisoindolin-1-one

Indeed, another bond length conformer still existed in a small ratio in CD_3OD. HRMS for $C_{19}H_{20}NO_3$ [M+H$^+$]: 310.1443, found 310.1450. ^{1}H NMR (600 MHz, CD_3OD) δ 7.80 (d, J = 7.6 Hz, 1H), 7.52 (t, J = 7.5 Hz, 1H), 7.48 (t, J = 7.4 Hz, 1H), 7.44 – 7.39 (m, 3H), 6.98 (d, J = 7.4 Hz, 1H), 6.61 (d, J = 7.6 Hz, 1H), 6.13 (s, 1H), 5.87 (s, 1H), 5.21 (s, 1H), 3.92 – 3.86 (m, 1H), 3.41 – 3.36 (m, 1H), 1.89 (d, J = 7.6 Hz, 1H), 1.80 – 1.72 (m, 1H), 1.00 (t, J = 7.4 Hz, 3H).

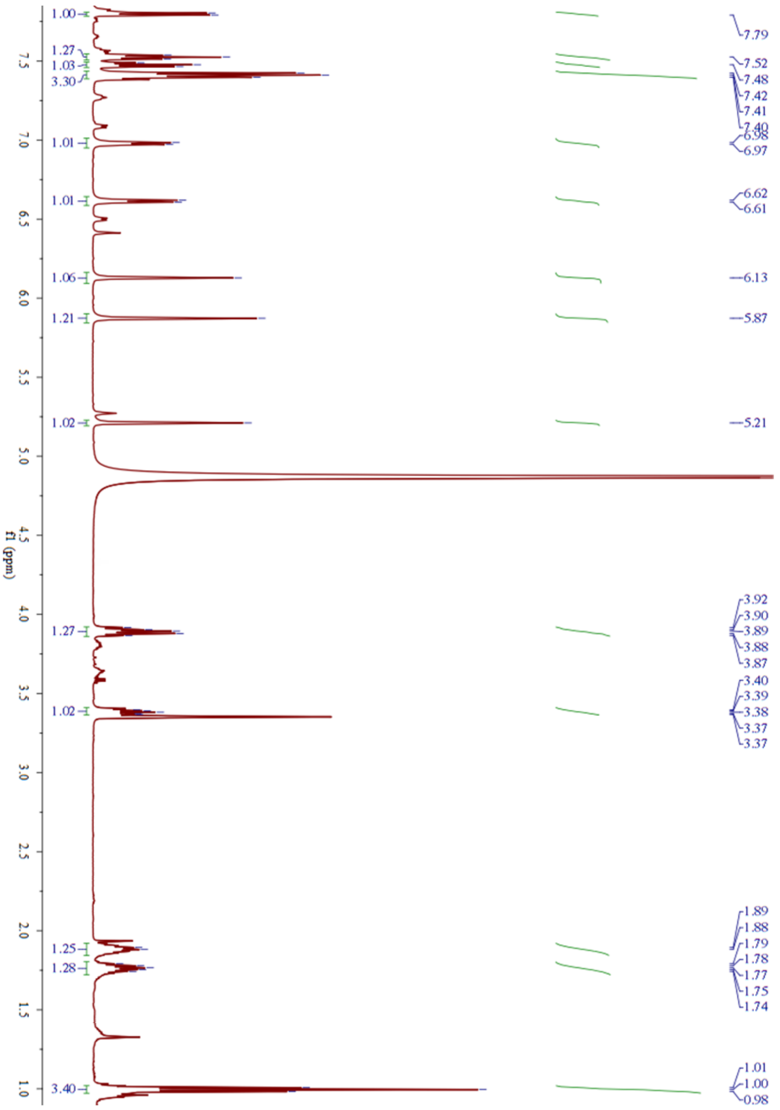

Figure S23. ^{1}H NMR for compound **7** in CD_3OD. The signal strength ratio is almost 1:0.19 between the two sets of ^{1}H NMR.

^{13}C NMR (151 MHz, CD_3OD) δ 171.07, 142.84, 142.12, 141.93, 139.35, 134.49, 132.40, 130.36, 130.12, 129.79, 124.41, 124.03, 122.62, 102.34, 82.28, 64.50, 43.71, 22.27, 11.63.

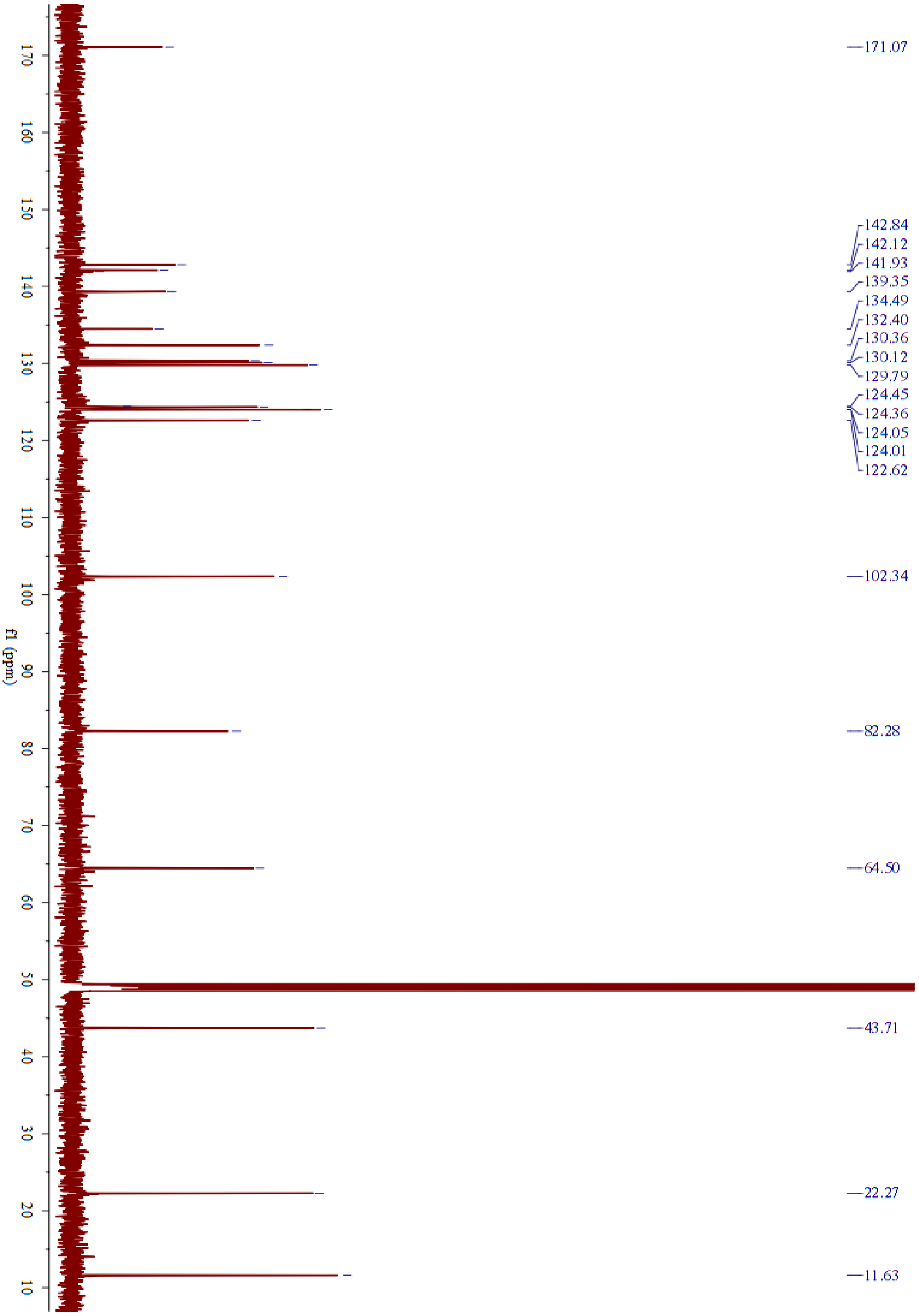

Figure S24. ^{13}C NMR for compound **7** in CD_3OD.

3-(3-hydroxy-1,3-dihydroisobenzofuran-1-yl)-2-propylisoindolin-1-one

^{1}H NMR (600 MHz, $CDCl_3$) δ 7.82 (d, *J* = 7.6 Hz), 7.79 (d, *J* = 7.6 Hz, 1H), 7.45 – 7.39 (m, 2H), 7.39 – 7.34 (m, 3H), 7.34 – 7.30 (m, 1H), 7.25 (m), 6.93 (d, *J* = 7.1 Hz), 6.62 (d, *J* = 7.6 Hz, 1H), 6.58 (d, *J* = 7.6 Hz), 6.34 (d, *J* = 7.5 Hz), 6.20 (s), 5.99 (d, *J* = 1.7 Hz, 1H), 5.97 (d, *J* = 2.0 Hz, 1H), 5.74 (d, *J* = 1.7 Hz), 4.96 (d, *J* = 1.8 Hz, 1H), 4.92 (d, *J* = 2.1 Hz), 3.95 – 3.89 (m), 3.88 – 3.80 (m, 1H), 3.41 (m), 3.13 (m, 1H), 1.85 – 1.71 (m, 1H), 1.76 – 1.75 (m),1.73-1.69 (m, 1H), 1.63-1.58 (m, 1H), 0.94 (t, *J* = 7.4 Hz), 0.89 (t, *J* = 7.4 Hz, 3H).

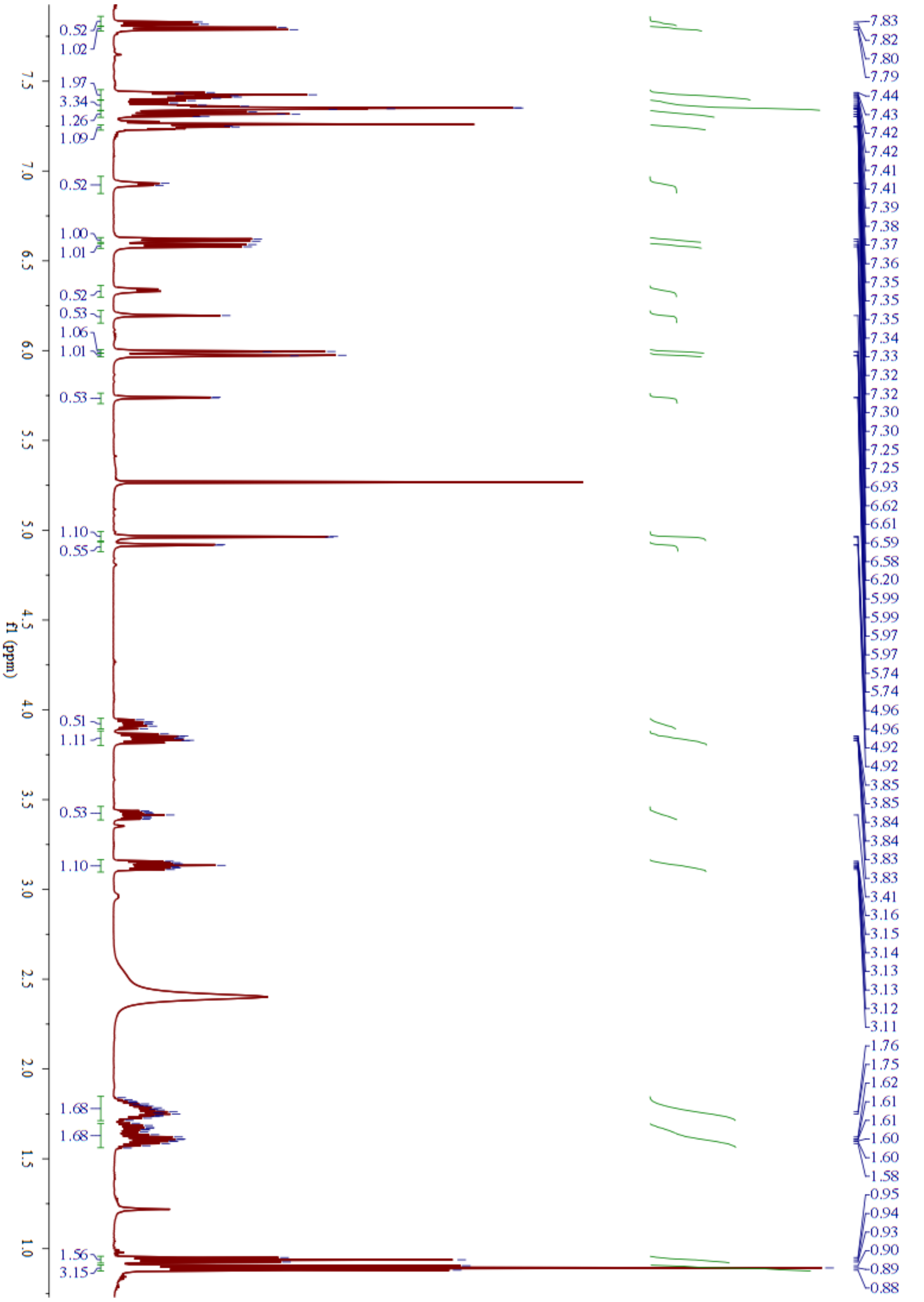

Figure S25. ^{1}H NMR for compound **7** in $CDCl_3$，Shift of proton of –OH is 2.38 ppm. The signal strength ratio is almost 1:0.50 between the two sets of ^{1}H NMR..

^{13}C NMR (151 MHz, $CDCl_3$) δ 169.26, 169.11, 140.99, 140.39, 137.79, 137.62, 134.04, 133.57, 131.23, 130.89, 129.60, 129.36, 129.13, 128.84, 124.01, 123.73, 123.45, 122.50, 121.24, 101.09, 81.77, 80.73, 63.24, 62.66, 42.65, 42.41, 21.51, 21.32, 11.39.

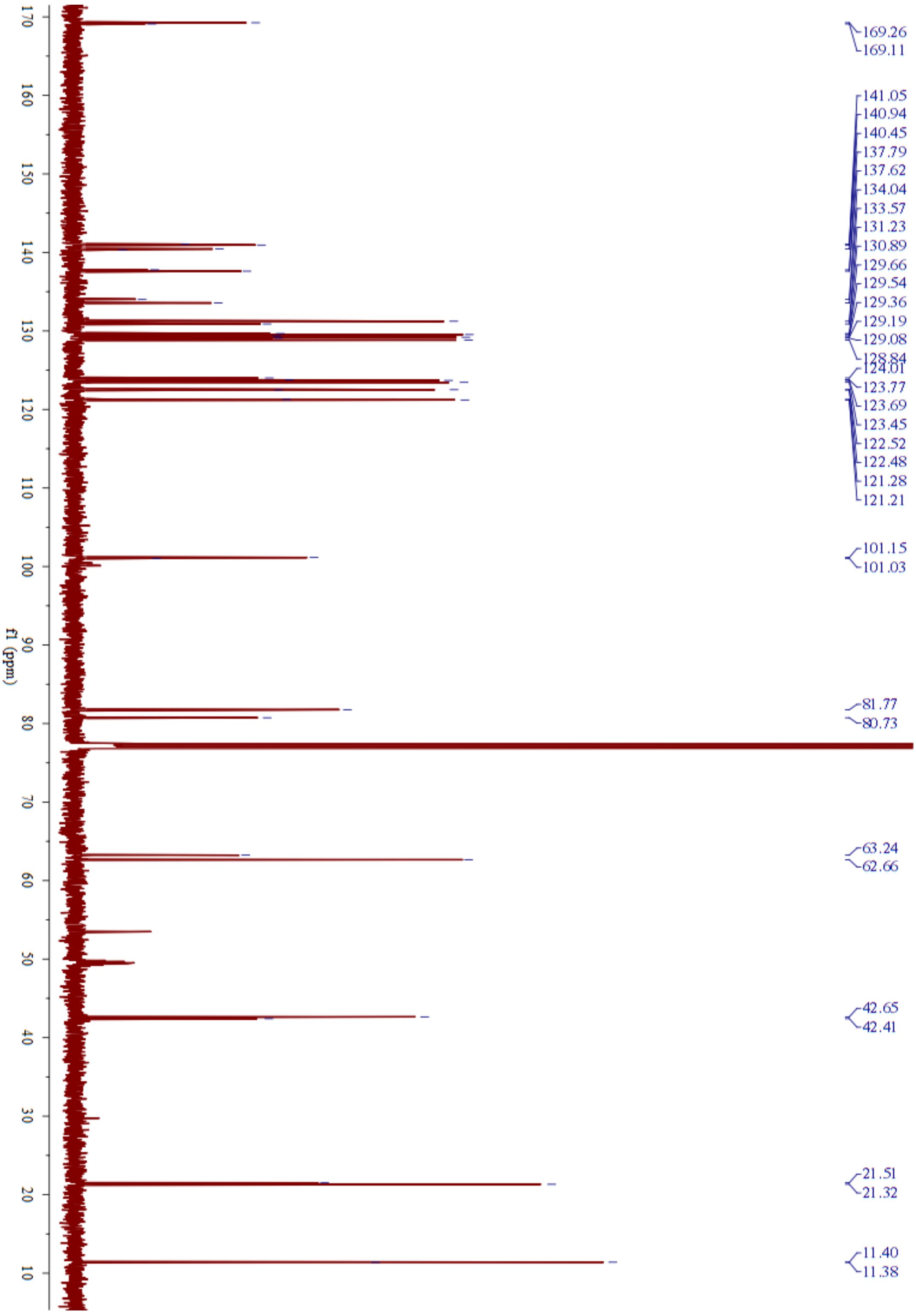

Figure S26. ^{13}C NMR for compound **7** in $CDCl_3$

Compound 8

2-butyl-3-(3-hydroxy-1,3-dihydroisobenzofuran-1-yl)isoindolin-1-one. HRMS for $C_{20}H_{22}NO_3$ [M+H$^+$]: : 324.1600. Found : 324.1607.

^{1}H NMR (600 MHz, CD_3OD) δ 7.78 (d, J = 7.6 Hz, 1H), 7.51 (t, J = 7.5 Hz, 1H), 7.46 (t, J = 7.4 Hz, 1H), 7.42 – 7.37 (m, 3H), 6.94 (d, J = 7.4 Hz, 1H), 6.62 (d, J = 7.6 Hz, 1H), 6.11 (s, 1H), 5.86 (s, 1H), 5.20 (s, 1H), 3.93 (m, 1H), 3.40 – 3.34 (m, 1H), 1.83 – 1.68 (m, 3H), 1.43 – 1.37 (m, 2H), 1.00 (t, J = 7.4 Hz, 3H).

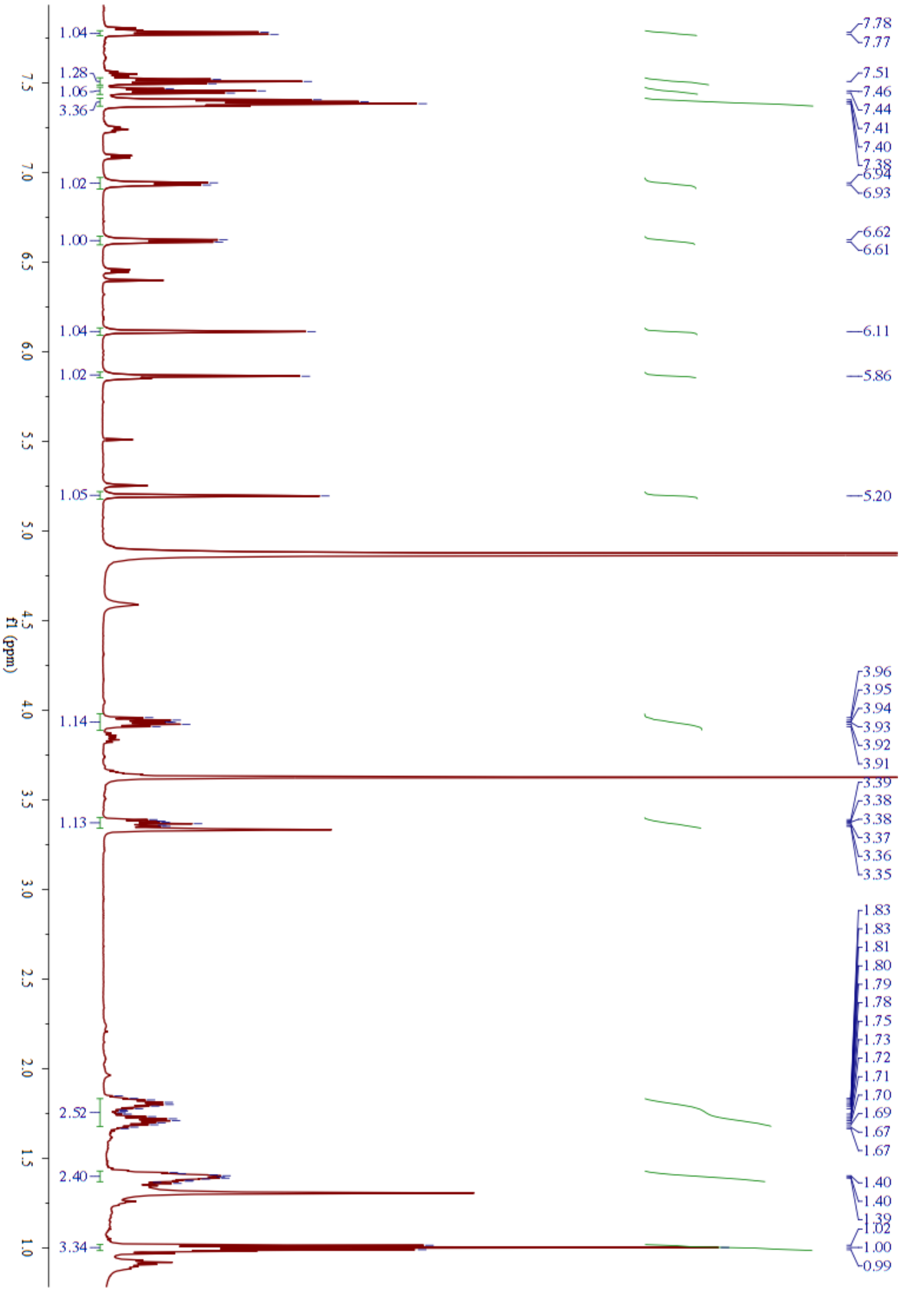

Figure S27. ^{1}H NMR for compound **8** in CD_3OD. The signal strength ratio is near 1:0.20 between the two sets of ^{1}H NMR.

^{13}C NMR (151 MHz, CD3OD) δ 170.96, 142.82, 142.09, 139.31, 134.48, 132.41, 130.36, 130.12, 129.79, 124.35, 124.02, 122.61, 102.31, 82.33, 64.43, 64.27, 41.74, 31.09, 21.13, 14.07.

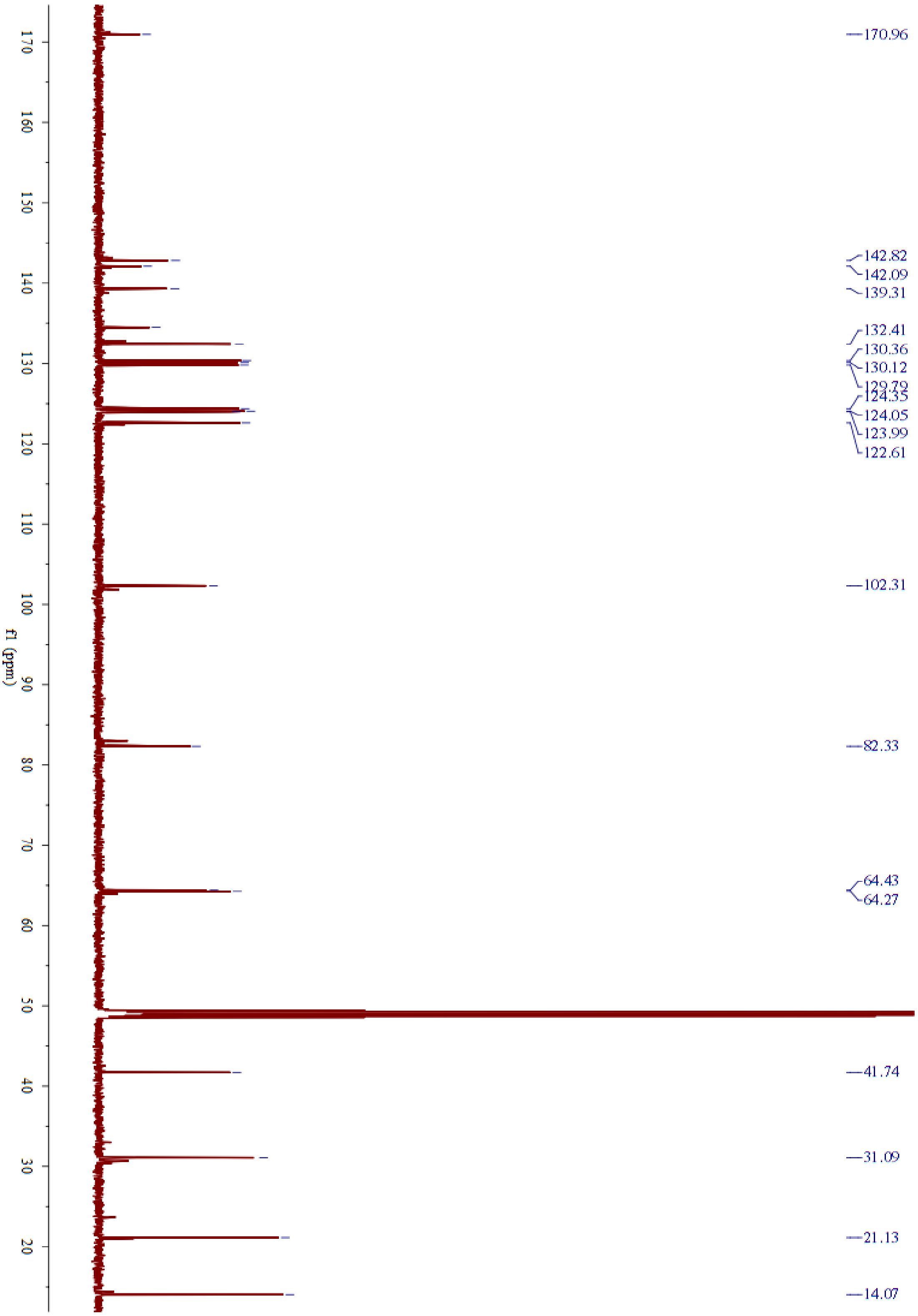

Figure S28. ^{13}C NMR for compound **8** in CD_3OD.

2-butyl-3-(3-hydroxy-1,3-dihydroisobenzofuran-1-yl)isoindolin-1-one

^{1}H NMR (600 MHz, $CDCl_3$) δ 7.86 (d, J = 7.5 Hz), 7.83 (d, J = 7.5 Hz, 1H), 7.50 – 7.33 (m, 5H), 7.26 – 7.22 (m, 1H), 7.18 – 7.15 (m), 6.75 (d, J = 7.6 Hz, 1H), 6.50 (d, J = 7.6 Hz, 1H), 6.18 (d, J = 10.8 Hz), 6.15 (d, J = 7.6 Hz, 1H), 6.10 (s, 1H), 6.03 (s, 1H), 5.78 (d, J = 2.0 Hz), 5.01 (d, J = 1.8 Hz, 1H), 4.89 (d, J = 2.0 Hz), 4.07 (m), 3.92 (m, 1H), 3.44 (m), 3.12 (m, 1H), 1.9-1.75 (m), 1.70 – 1.67 (m, 2H), 1.58-1.46 (m), 1.45 – 1.38 (m, 2H), 1.33 (m), 0.98 (t, J=7.4 Hz), 0.94 (t, J = 7.4 Hz, 2H).

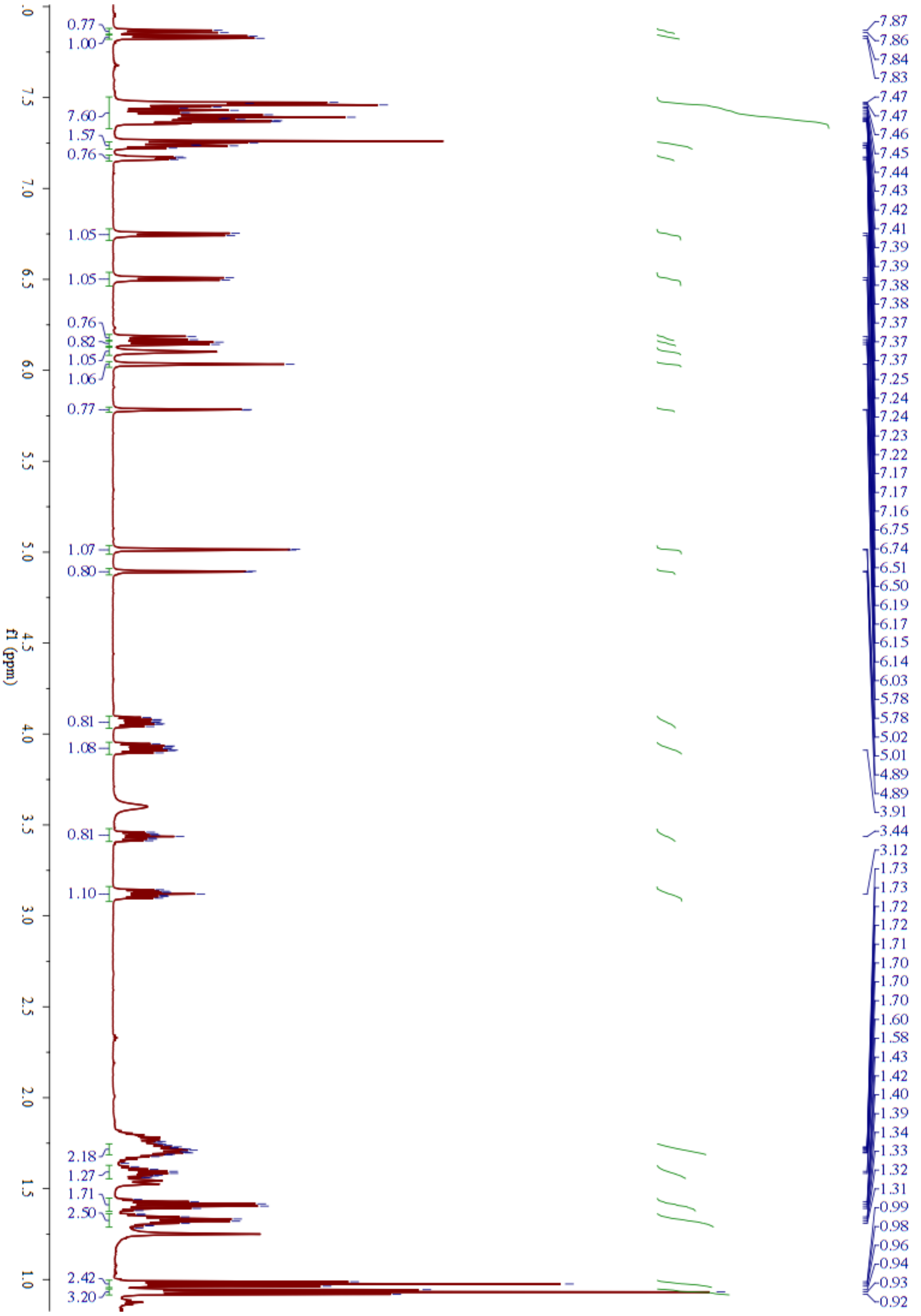

Figure S29. ^{1}H NMR for compound **8** in $CDCl_3$. Proton shift of –OH is 3.70 ppm. The signal strength ratio is near 1:0.75 between the two sets of ^{1}H NMR.

^{13}C NMR (151 MHz, $CDCl_3$) δ 168.81, 168.44, 140.94, 140.46, 140.33, 138.12, 137.63, 134.55, 133.84, 131.19, 130.61, 129.82, 129.53, 129.29, 129.15, 128.85, 124.29, 123.89, 123.49, 122.49, 122.28, 121.38, 101.28, 82.37, 80.42, 63.34, 62.31, 40.79, 40.39, 30.49 , 30.17, 20.31, 13.95.

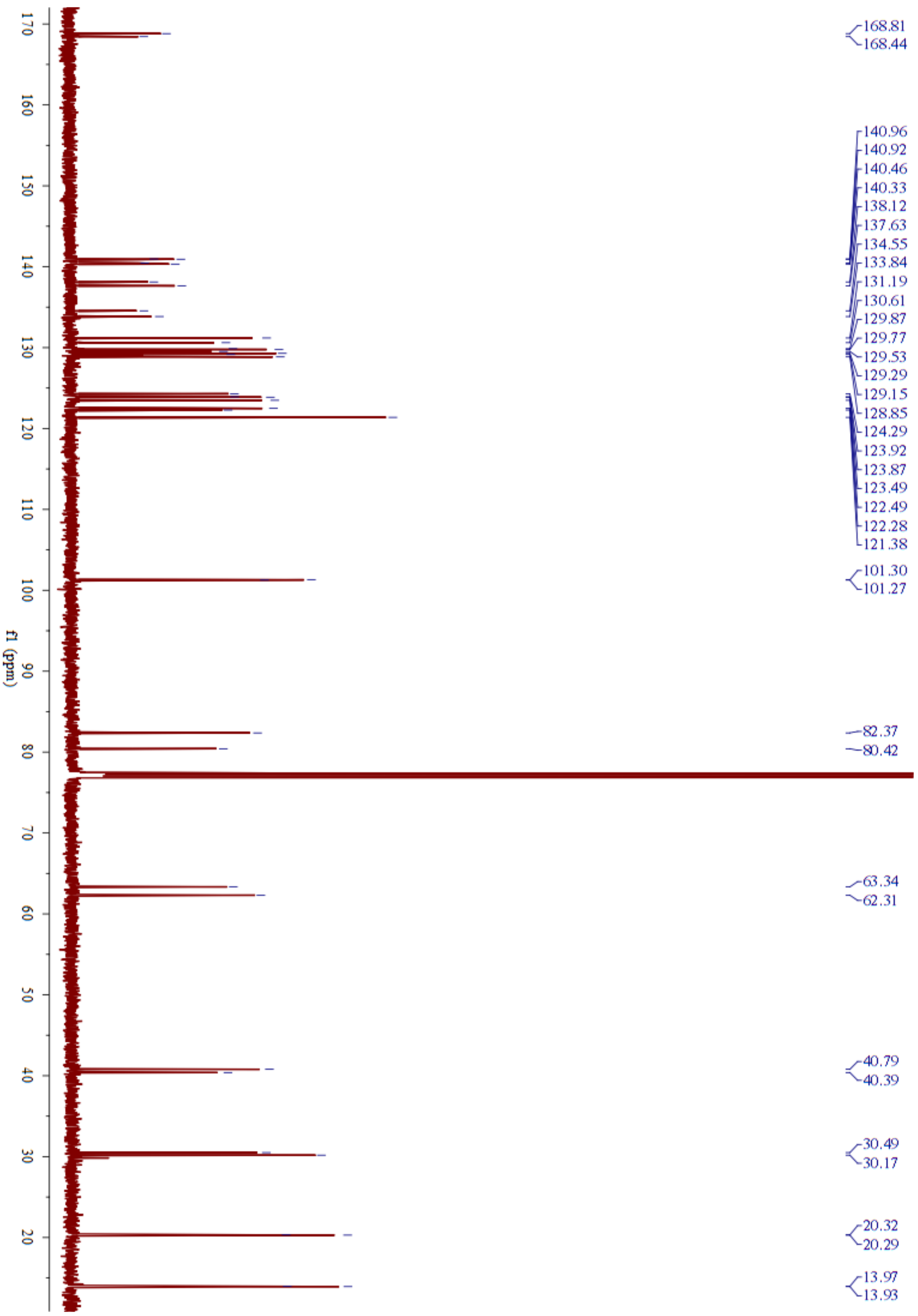

Figure S30. ^{13}C NMR for compound **8** in $CDCl_3$.

Compound **9**

2-(tert-butyl)-3-(3-hydroxy-1,3-dihydroisobenzofuran-1-yl)isoindolin-1-one

HRMS for $C_{20}H_{22}NO_3$ [M+H$^+$]: 324.1600. found 324.1608. ^{1}H NMR (600 MHz, CD_3OD) δ 7.67 (d, *J* = 7.6 Hz, 1H), 7.64 (d, *J* = 7.5 Hz, 1H), 7.58 (t, *J* = 7.4 Hz, 1H), 7.51 (t, *J* = 7.5 Hz, 1H), 7.38 (t, *J* = 7.5 Hz, 1H), 7.31 (d, *J* = 7.5 Hz, 1H), 7.10 (t, *J* = 7.5 Hz, 1H), 6.21 (s, 1H), 5.88 (d, *J* = 7.6 Hz, 1H), 5.32 (s, 1H), 5.21 (d, *J* = 1.9 Hz, 1H), 1.76 (s, 9H).

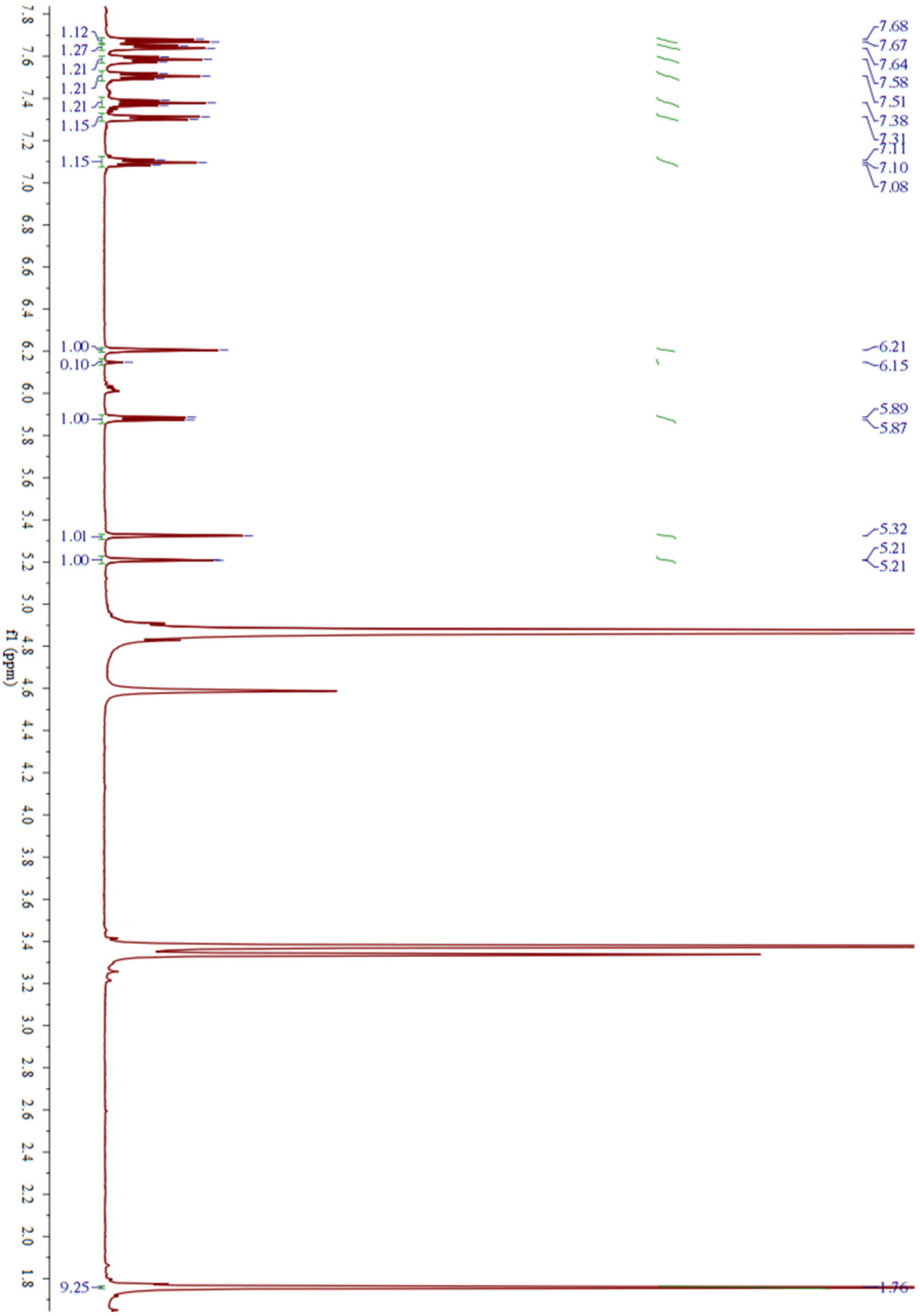

Figure S31. ^{1}H NMR for compound **9** in CD_3OD. The signal strength ratio is 1:0.10 between the two sets of ^{1}H NMR.

^{13}C NMR (151 MHz, CD_3OD) δ 142.49, 142.10, 140.46, 136.32, 131.14, 130.52, 130.20, 129.24, 124.30, 123.53, 123.15, 122.80, 102.53, 83.63, 66.23, 56.52, 49.85, 28.93.

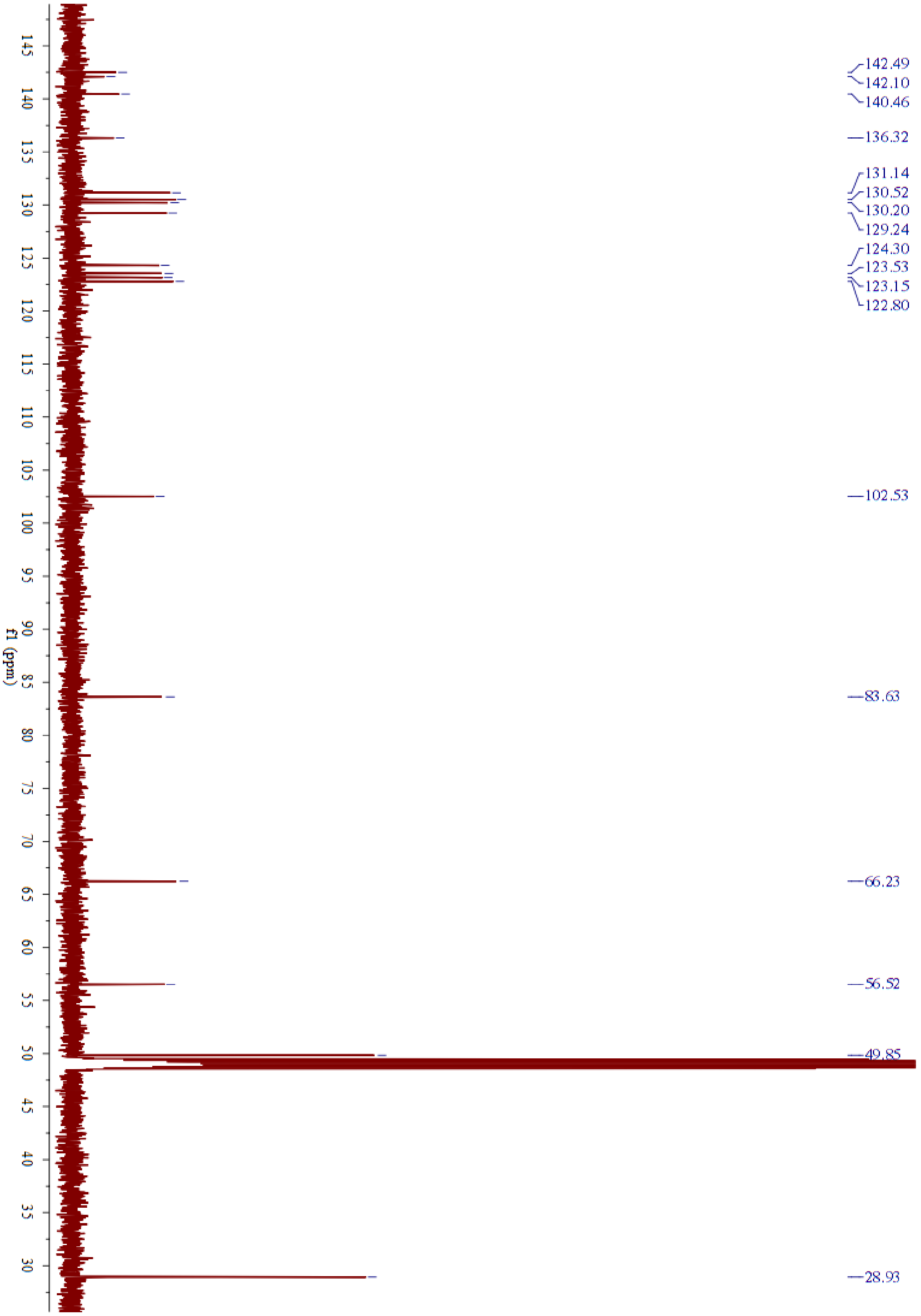

Figure S32. ^{13}C NMR for compound **9** in CD_3OD.

2-(tert-butyl)-3-(3-hydroxy-1,3-dihydroisobenzofuran-1-yl)isoindolin-1-one

The two sets of ^{1}H NMR had 0.7:1 ratio. It is not easy to identify the proton's shift in overlapped signal area. Thus, the two sets of ^{1}H NMR are treated as 1:1 in ^{1}H NMR. Proton numbers are listed for both of the NMR. For example, shift at 7.74 just had 0.70 proton (integration value), we write one proton here like 7.74 (d, J = 7.5 Hz, 1H).

^{1}H NMR (600 MHz, $CDCl_3$) δ 7.82 (d, J = 7.5 Hz, 1H), 7.74 (d, J = 7.5 Hz, 1H), 7.57 – 7.44 (m, 5H), 7.43 – 7.30 (m, 5H), 7.08 (t, J = 7.5 Hz, 1H), 7.03 (t, J = 7.5 Hz, 1H), 6.15 (s, 1H), 6.03 (d, J = 12.5 Hz, 1H), 5.94 (s, 1H), 5.79 (d, J = 7.6 Hz, 1H), 5.77 (d, J = 7.6 Hz, 1H), 5.34 (d, J = 6.0 Hz, 1H), 5.07 (d, J = 1.4 Hz, 1H), 5.02 (d, J = 1.2 Hz, 1H), 1.75 (s, 9H), 1.71 (s, 9H).

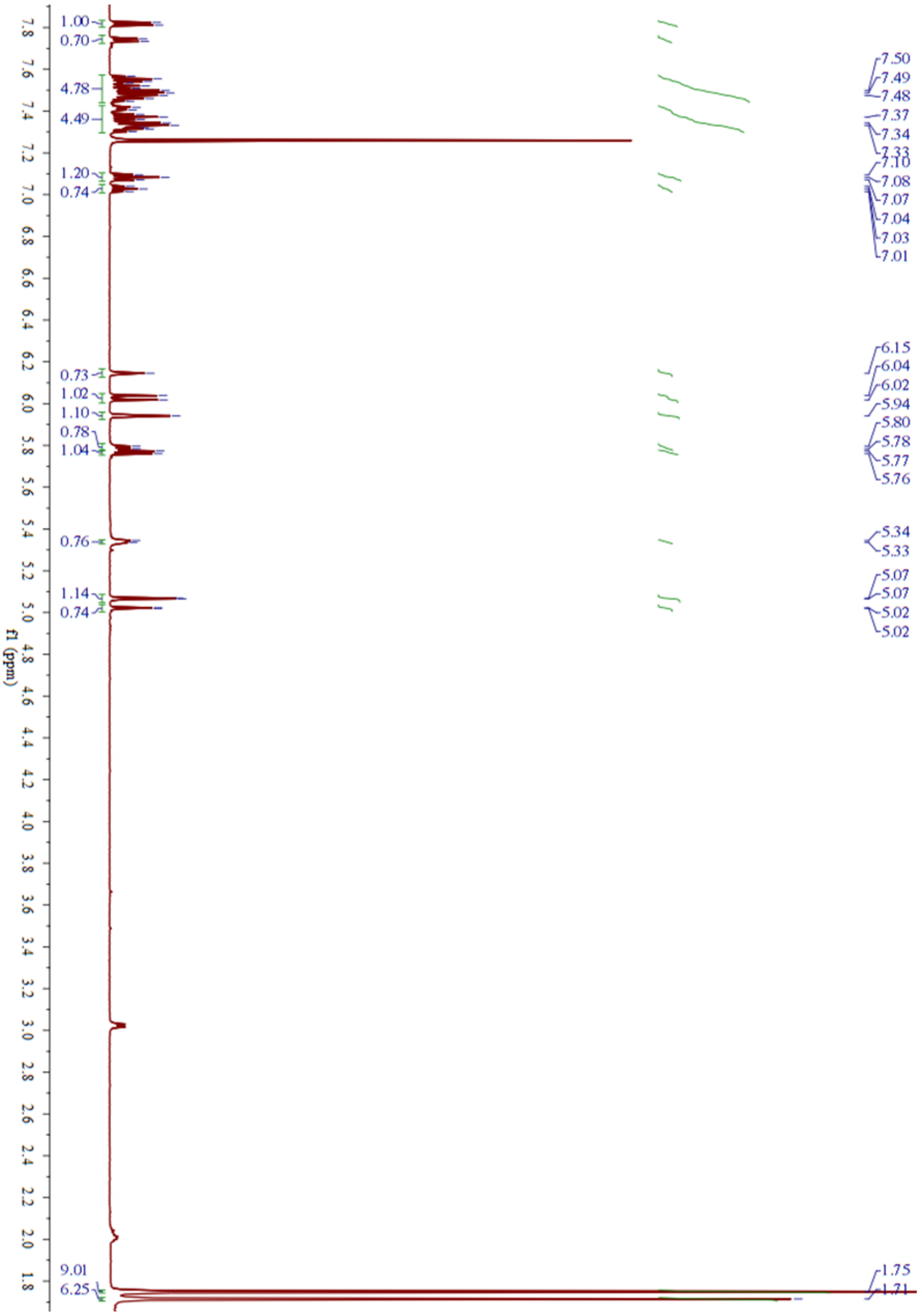

Figure S33. ^{13}C NMR for compound **9** in $CDCl_3$. The shift of proton of –OH is 3.02 ppm. The signal strength ratio is almost 0.7:1.0 between the two sets of ^{1}H NMR.

^{13}C NMR (151 MHz, $CDCl_3$) δ 169.23, 168.60, 140.82, 140.49, 140.45, 140.35, 139.19, 138.88, 136.41, 135.60, 129.84, 129.60, 129.45, 128.92, 128.31, 124.04, 123.71, 122.96, 122.03, 121.84, 121.32, 121.18, 101.55, 101.31, 83.02, 82.78 , 64.68 , 55.18, 29.85 , 28.88.

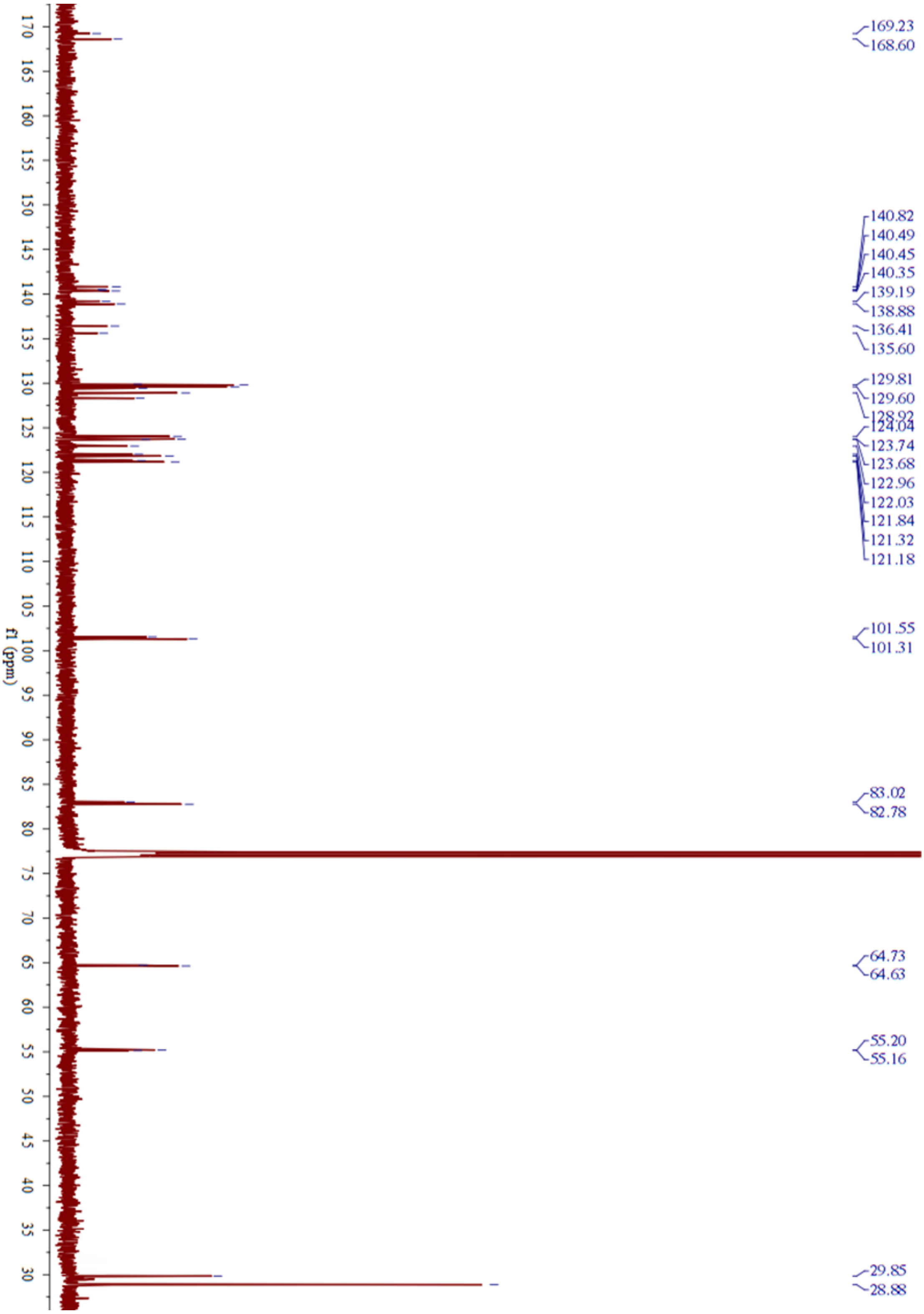

Figure S34. ^{13}C NMR for compound **9** in CD_3Cl_3.

Compound 10

3-(3-hydroxy-1,3-dihydroisobenzofuran-1-yl)-2-isobutylisoindolin-1-one

HRMS calcd for $C_{20}H_{22}NO_3$ [M+H+]: 324.1600. Found : 324.1606. ^{1}H NMR (600 MHz, CD3OD) δ 7.79 (d, J = 7.6 Hz, 1H), 7.52 (t, J = 7.5 Hz, 1H), 7.46 (t, J = 7.4 Hz, 1H), 7.41 – 7.37 (m, 3H), 6.93 (d, J = 7.4 Hz, 1H), 6.64 (d, J = 7.6 Hz, 1H), 6.11 (s, 1H), 5.86 (s, 1H), 5.20 (s, 1H), 3.72 (dd, J = 13.8, 9.8 Hz, 1H), 3.19 (dd, J = 13.8, 5.5 Hz, 1H), 2.35 – 2.24 (m, 1H), 1.05 (d, J = 6.7 Hz, 3H), 0.89 (d, J = 6.6 Hz, 3H).

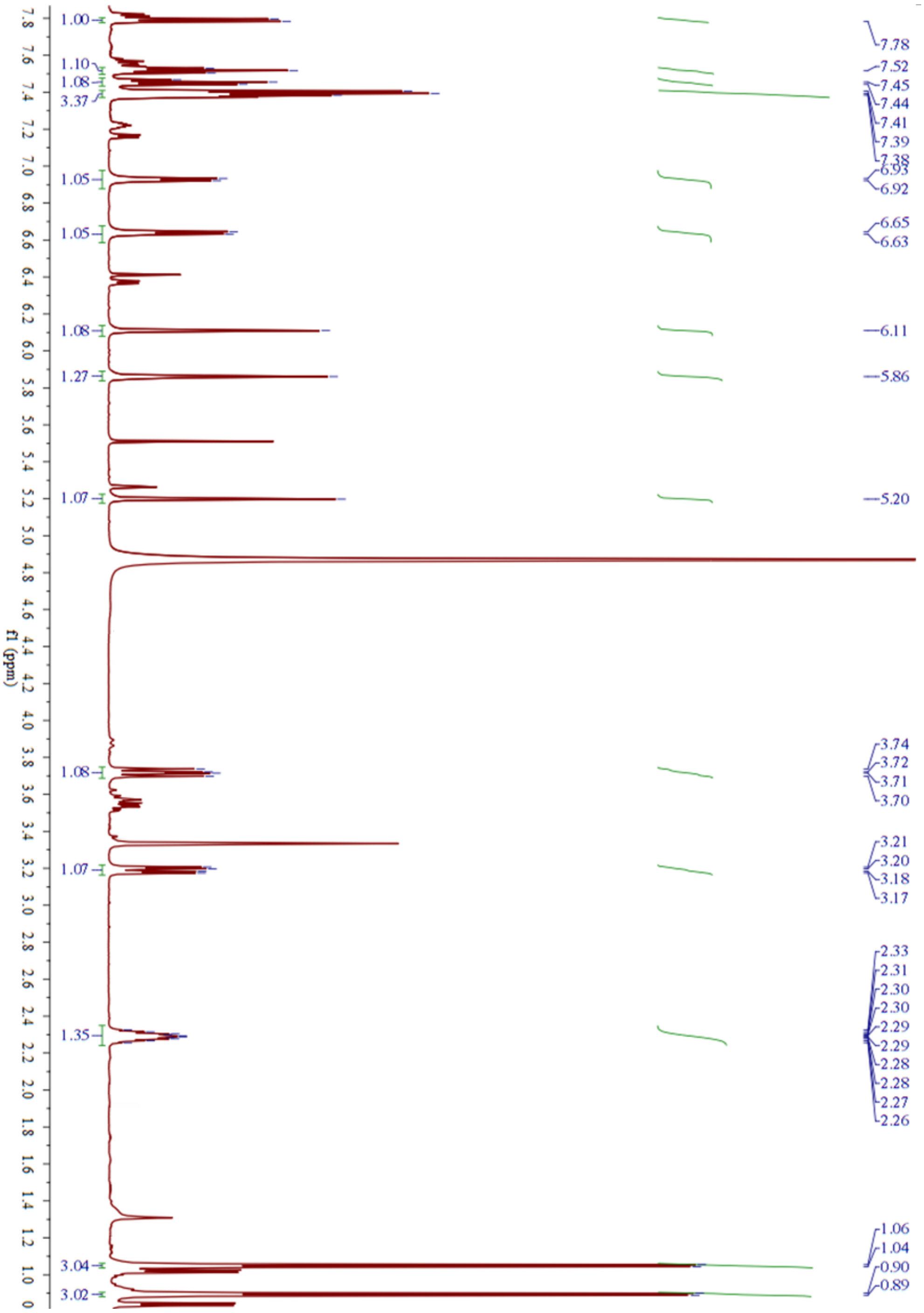

Figure S35. ^{1}H NMR for compound **10** in CD_3OD. The signal strength ratio is almost 1.0:0.22 between the two sets of ^{1}H NMR.

^{13}C NMR (151 MHz, CD_3OD) δ 171.21, 142.75, 142.09, 139.29, 134.36, 132.46, 130.37, 130.12, 129.84, 124.38, 124.10, 122.60, 102.35, 82.26, 64.46, 28.36, 20.80, 20.10.

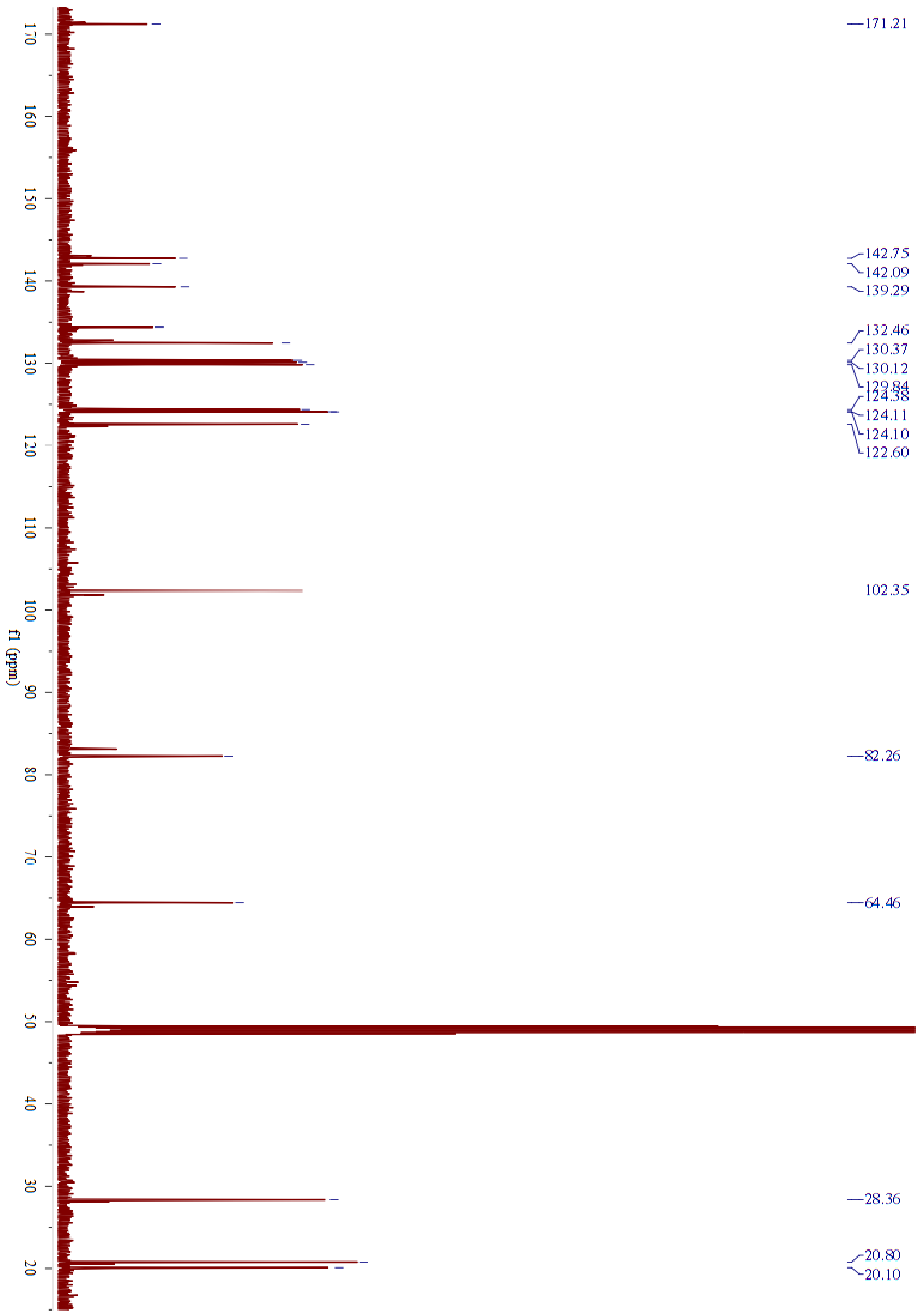

Figure S36. ^{13}C NMR for compound **10** in CD_3OD.

3-(3-hydroxy-1,3-dihydroisobenzofuran-1-yl)-2-isobutylisoindolin-1-one

^{1}H NMR (600 MHz, $CDCl_3$) δ 7.87 (d, *J* = 7.6 Hz, 1H), 7.83 (d, *J* = 7.5 Hz, 1H), 7.49 – 7.41 (m, 3H), 7.40 – 7.33 (m, 4H), 7.27 (d, *J* = 1.4 Hz, 1H), 7.24 (d, *J* = 7.3 Hz, 1H), 7.09 (s, 1H), 6.69 (d, *J* = 7.6 Hz, 1H), 6.55 (d, *J* = 7.6 Hz, 1H), 6.22 (dd, *J* = 7.3, 3.8 Hz, 1H), 6.18 (d, *J* = 7.8 Hz, 1H), 6.02 (d, *J* = 9.8 Hz, 2H), 5.77 (d, *J* = 1.5 Hz, 1H), 5.00 (d, *J* = 1.5 Hz, 1H), 4.90 (d, *J* = 1.9 Hz, 1H), 3.86 (dd, *J* = 14.0, 9.8 Hz, 1H), 3.72 (dd, *J* = 13.9, 9.9 Hz, 1H), 3.27 (dd, *J* = 14.1, 5.5 Hz, 1H), 2.97 (dd, *J* = 13.9, 5.4 Hz, 1H), 2.26 – 2.09 (m, 2H), 1.04 (d, *J* = 6.6 Hz, 3H), 0.97 (d, *J* = 6.7 Hz, 3H), 0.90 (d, *J* = 6.6 Hz, 3H), 0.84 (d, *J* = 6.6 Hz, 3H).

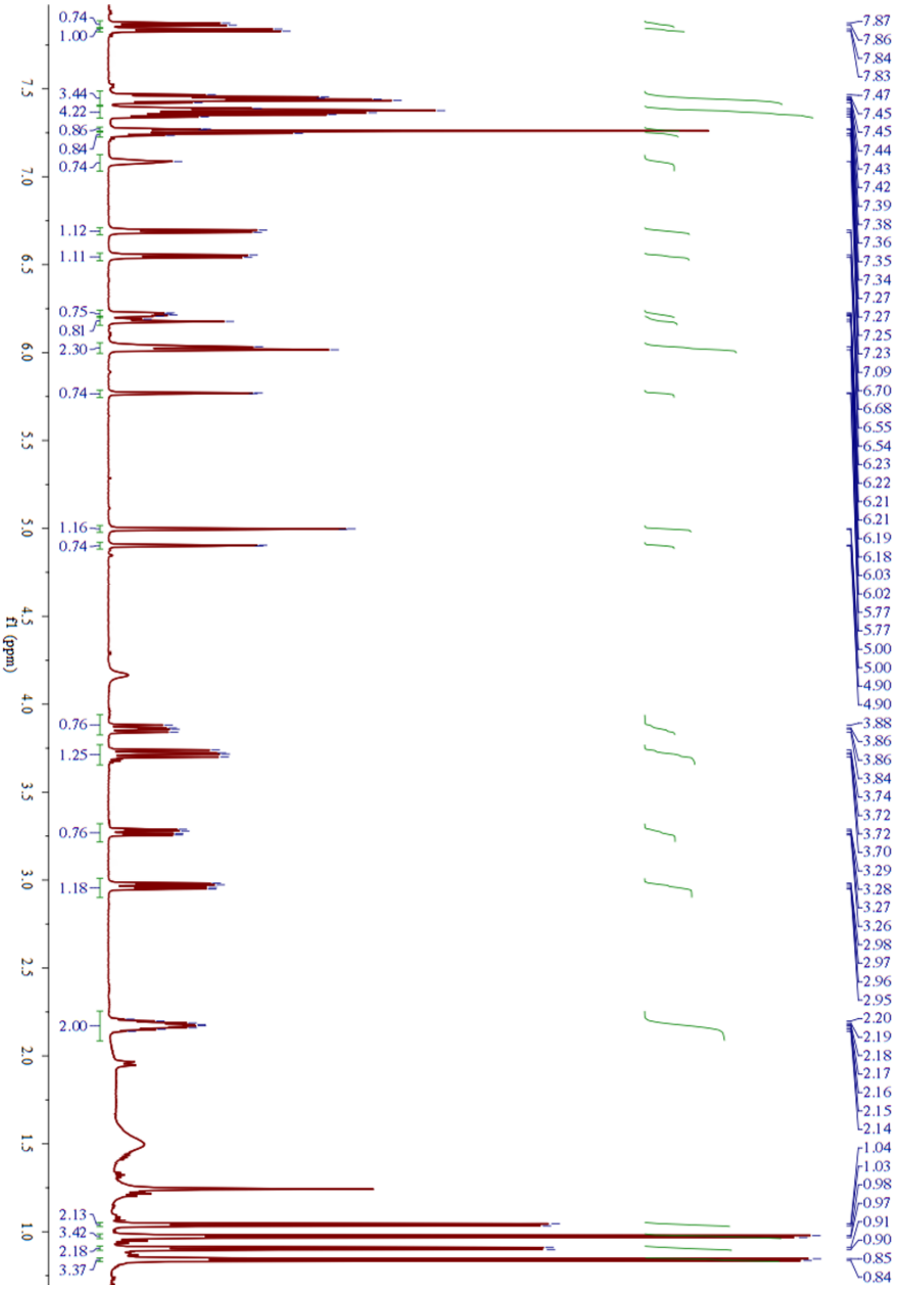

Figure S37. ^{1}H NMR for compound **10** in $CDCl_3$. Shift of proton of –OH is 4.37 ppm. The signal strength ratio is almost 1.0:0.74 between the two sets of ^{1}H NMR

^{13}C NMR (151 MHz, $CDCl_3$) δ 169.33, 169.16, 140.93, 140.49, 137.92, 134.45, 134.12, 130.94, 130.62, 129.48, 129.27, 129.05, 128.78, 128.69, 123.59, 123.25, 122.34, 121.11, 100.97, 100.72, 82.01, 81.26, 65.16, 64.96, 56.00, 55.67, 49.11, 49.04, 48.82, 48.68, 29.42, 24.58.

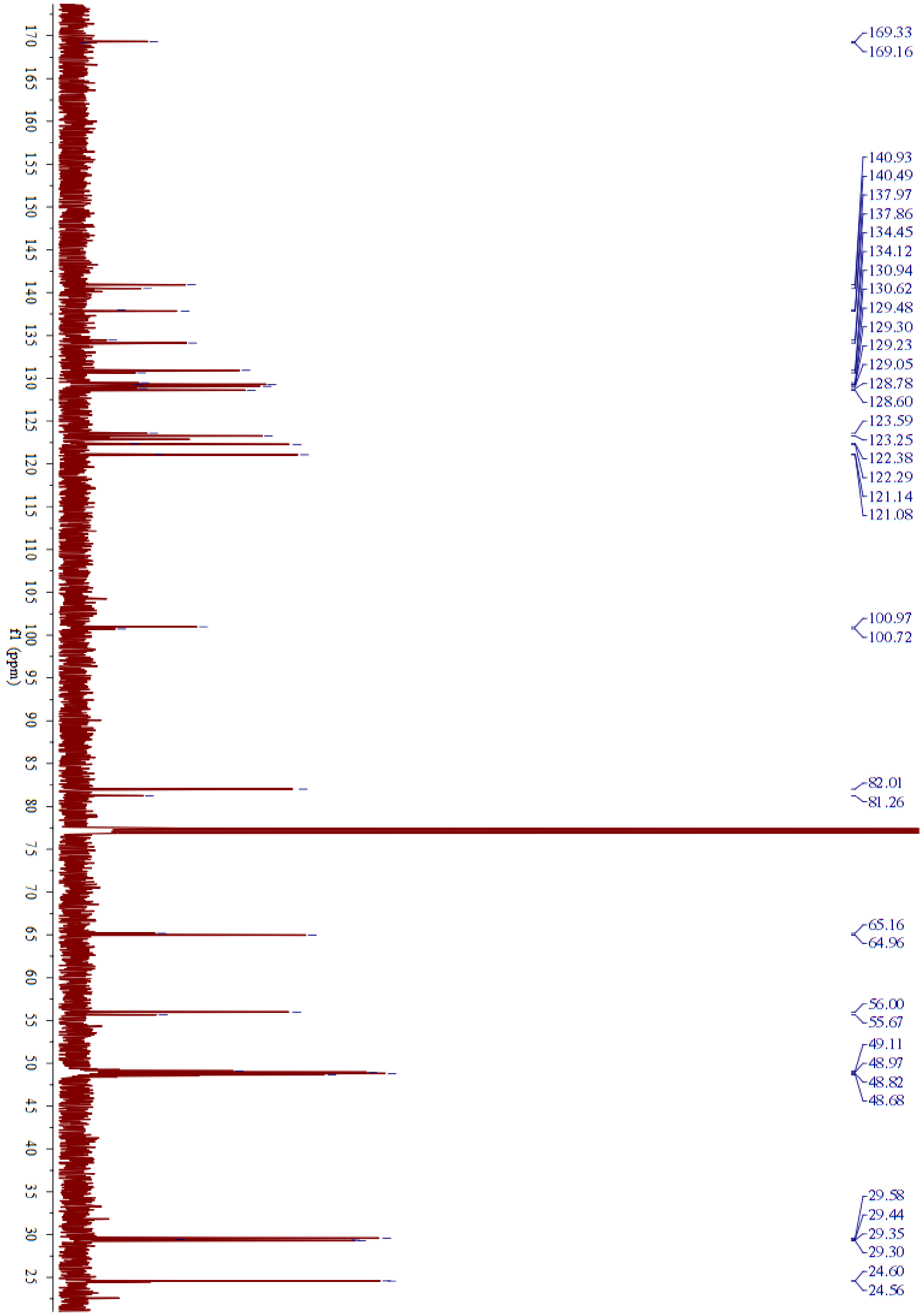

Figure S38. ^{13}C NMR for compound **10** in $CDCl_3$.

Compound 11

2-cyclopentyl-3-(3-hydroxy-1,3-dihydroisobenzofuran-1-yl)isoindolin-1-one

HRMS calcd for $C_{21}H_{22}NO_3$ [M+H$^+$]: 336.1600, found: 336.1605. ^{1}H NMR (600 MHz, CD_3OD) δ 7.75 (d, *J* = 7.6 Hz, 1H), 7.54 – 7.42 (m, 3H), 7.41 (d, *J* = 7.3 Hz, 1H), 7.32 (t, *J* = 7.6 Hz, 1H), 7.11 (d, *J* = 7.3 Hz, 1H), 6.13 (s, 1H), 5.78 (d, *J* = 1.5 Hz, 1H), 5.17 (s, 1H), 4.40 – 4.33 (m, 1H), 2.20 (dd, *J* = 12.2, 8.4 Hz, 1H), 2.17 – 2.12 (m, 1H), 2.12 – 2.00 (m, 2H), 2.00 – 1.87 (m, 4H).

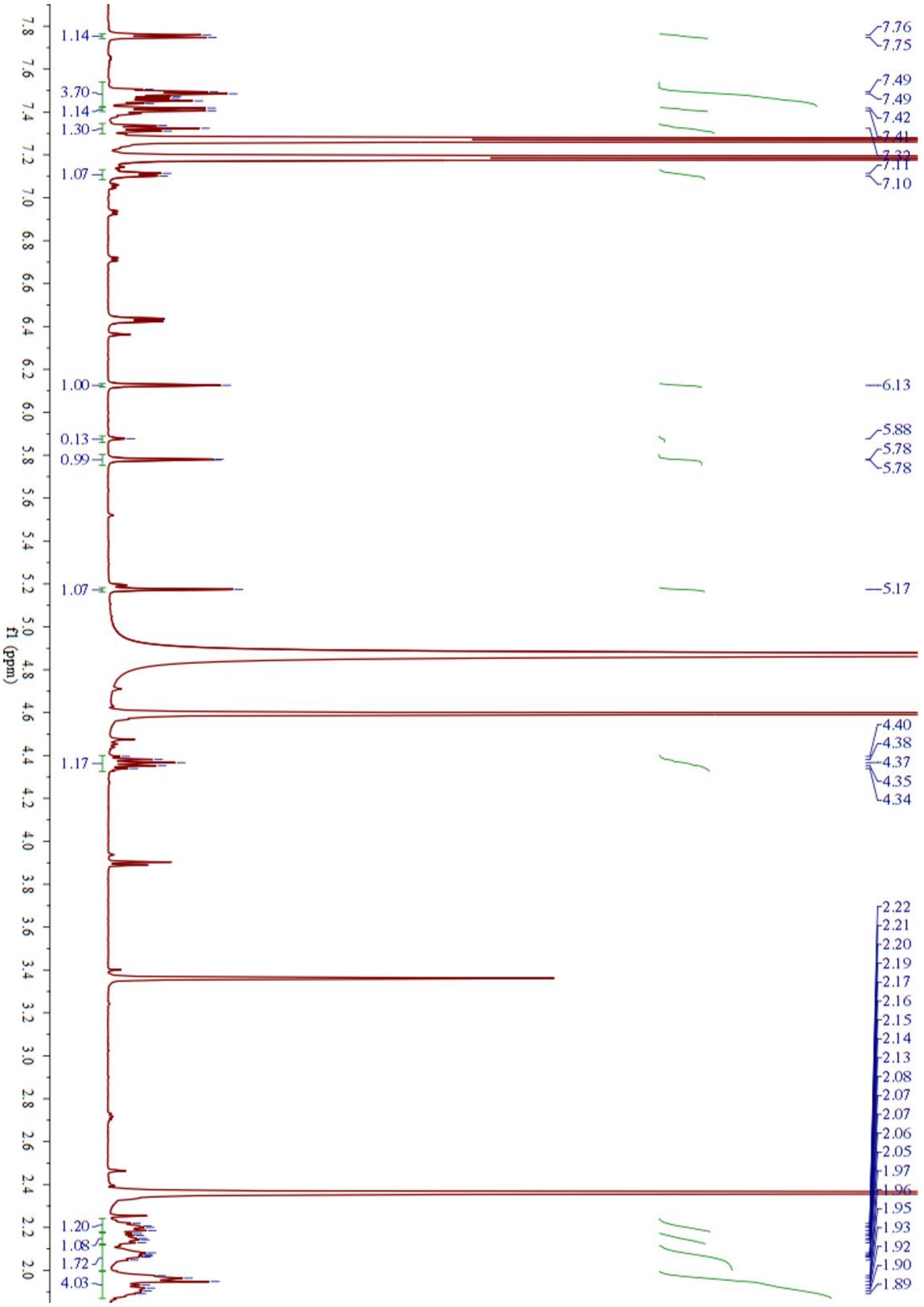

Figure S39. ^{1}H NMR for compound **11** in CD_3OD (minor toluene was used). The signal strength ratio is 1.0:0.14 between the two sets of ^{1}H NMR.

^{13}C NMR (151 MHz, CD_3OD) δ 170.78, 142.81, 142.20, 139.62, 137.98, 132.03, 130.35, 130.11, 129.92, 124.31, 123.78, 123.53, 122.67, 102.36, 82.91, 65.12, 57.40, 30.25, 25.81, 21.14.

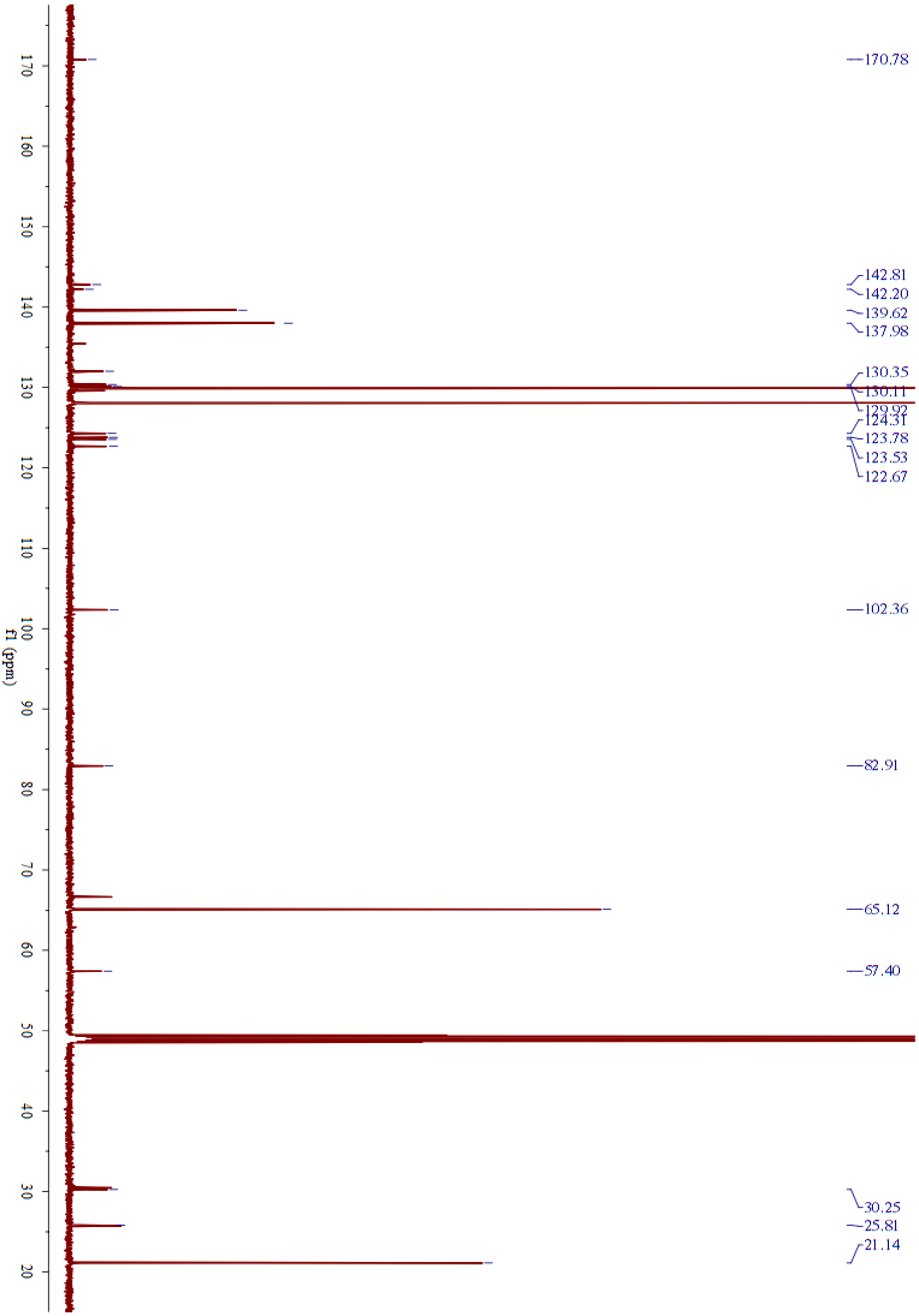

Figure S40. ^{13}C NMR for compound **11** in CD_3OD.

2-cyclopentyl-3-(3-hydroxy-1,3-dihydroisobenzofuran-1-yl)isoindolin-1-one

^{1}H NMR (600 MHz, $CDCl_3$) δ 7.82 (d, *J* = 7.5 Hz, 1H), 7.77 (d, *J* = 7.5 Hz, 1H), 7.49 (ddd, *J* = 18.6, 10.7, 6.8 Hz, 2H), 7.45 – 7.34 (m, 5H), 7.29 (t, *J* = 7.1 Hz, 3H), 7.17 (td, *J* = 7.5, 0.9 Hz, 1H), 6.65 (d, *J* = 7.6 Hz, 1H), 6.53 (d, *J* = 7.6 Hz, 1H), 6.14 (s, 1H), 6.04 (s, 1H), 5.98 (dd, *J* = 10.0, 4.7 Hz, 2H), 5.80 (d, *J* = 1.5 Hz, 1H), 4.96 (d, *J* = 1.8 Hz, 1H), 4.90 (d, *J* = 2.0 Hz, 1H), 4.37 (p, *J* = 8.8 Hz, 1H), 4.22 (p, *J* = 8.8 Hz, 1H), 2.21 – 2.00 (m, 4H), 1.98 – 1.90 (m, 4H), 1.88 – 1.83 (m, 3H), 1.68 – 1.61 (m, 3H), 1.55 (dd, *J* = 11.0, 6.7 Hz, 2H).

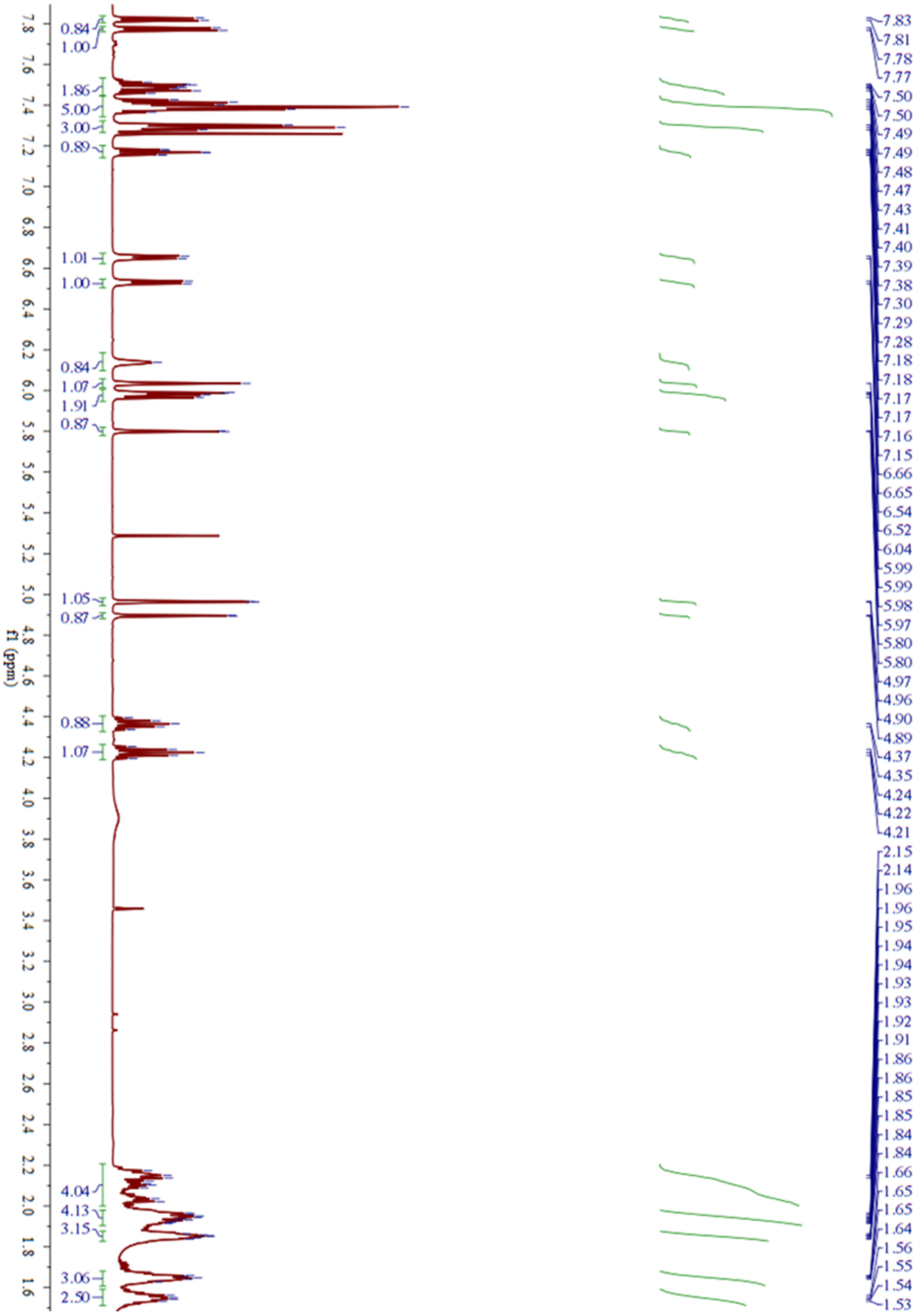

Figure S41. ^{1}H NMR for compound **11** in $CDCl_3$. Shift of proton of –OH is near 3.89 ppm (5.26 ppm is the CD_2Cl_2 signal). The signal strength ratio is almost 1.0:0.84 between the two sets of ^{1}H NMR.

^{13}C NMR (151 MHz, $CDCl_3$) δ 168.78, 168.44, 140.97, 140.50, 138.43, 138.03, 134.70, 130.91, 130.30, 129.84, 129.60, 129.26, 129.07, 128.72, 123.93, 123.41, 122.31, 122.08, 121.35, 101.38, 101.21, 82.83 , 81.32 , 65.15, 64.73, 56.14 , 55.62 , 29.64, 24.85, 24.69.

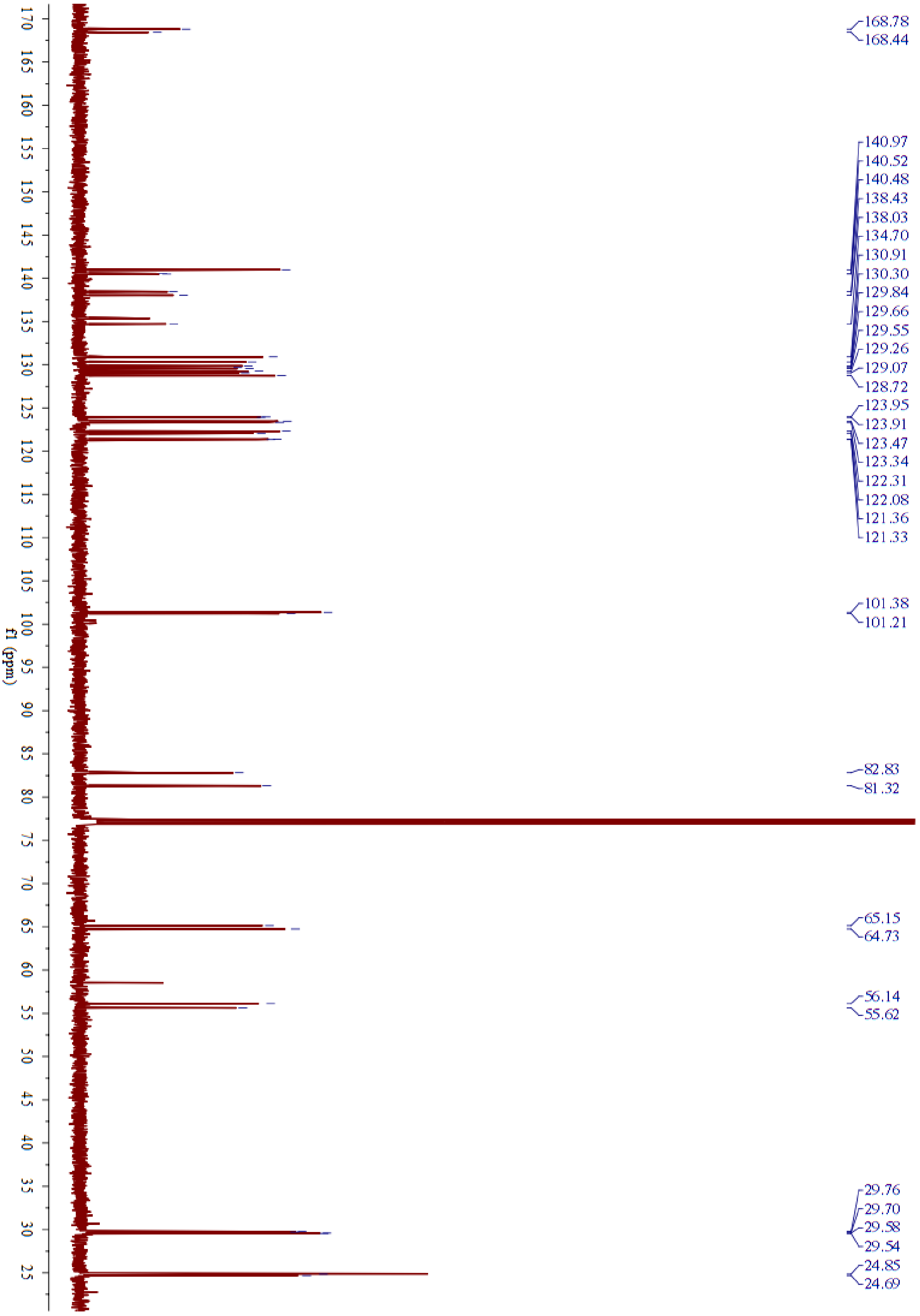

Figure S42. ^{13}C NMR for compound **11** in $CDCl_3$.

Compound **12**

2-cyclohexyl-3-(3-hydroxy-1,3-dihydroisobenzofuran-1-yl)isoindolin-1-one HRMS calcd. for $C_{22}H_{24}NO_3$ [M+H$^+$]: 350.1756, found: 350.1762. ^{1}H NMR (600 MHz, CD_3OD) δ 7.74 (d, *J* = 7.6 Hz, 1H), 7.53 – 7.43 (m, 3H), 7.42 – 7.38 (m, 1H), 7.31 (t, *J* = 7.5 Hz, 1H), 7.16 (d, *J* = 6.9 Hz, 1H), 6.38 (d, *J* = 7.4 Hz, 1H), 6.16 (s, 1H), 5.75 (s, 1H), 5.21 (d, *J* = 11.0 Hz, 1H), 3.89 (ddd, *J* = 12.2, 8.7, 3.6 Hz, 1H), 2.27 (tt, *J* = 15.9, 7.9 Hz, 1H), 2.13 (qd, *J* = 12.6, 3.4 Hz, 1H), 1.96 – 1.90 (m, 2H), 1.82 – 1.71 (m, 2H), 1.50 – 1.39 (m, 2H), 1.38 – 1.25 (m, 2H).

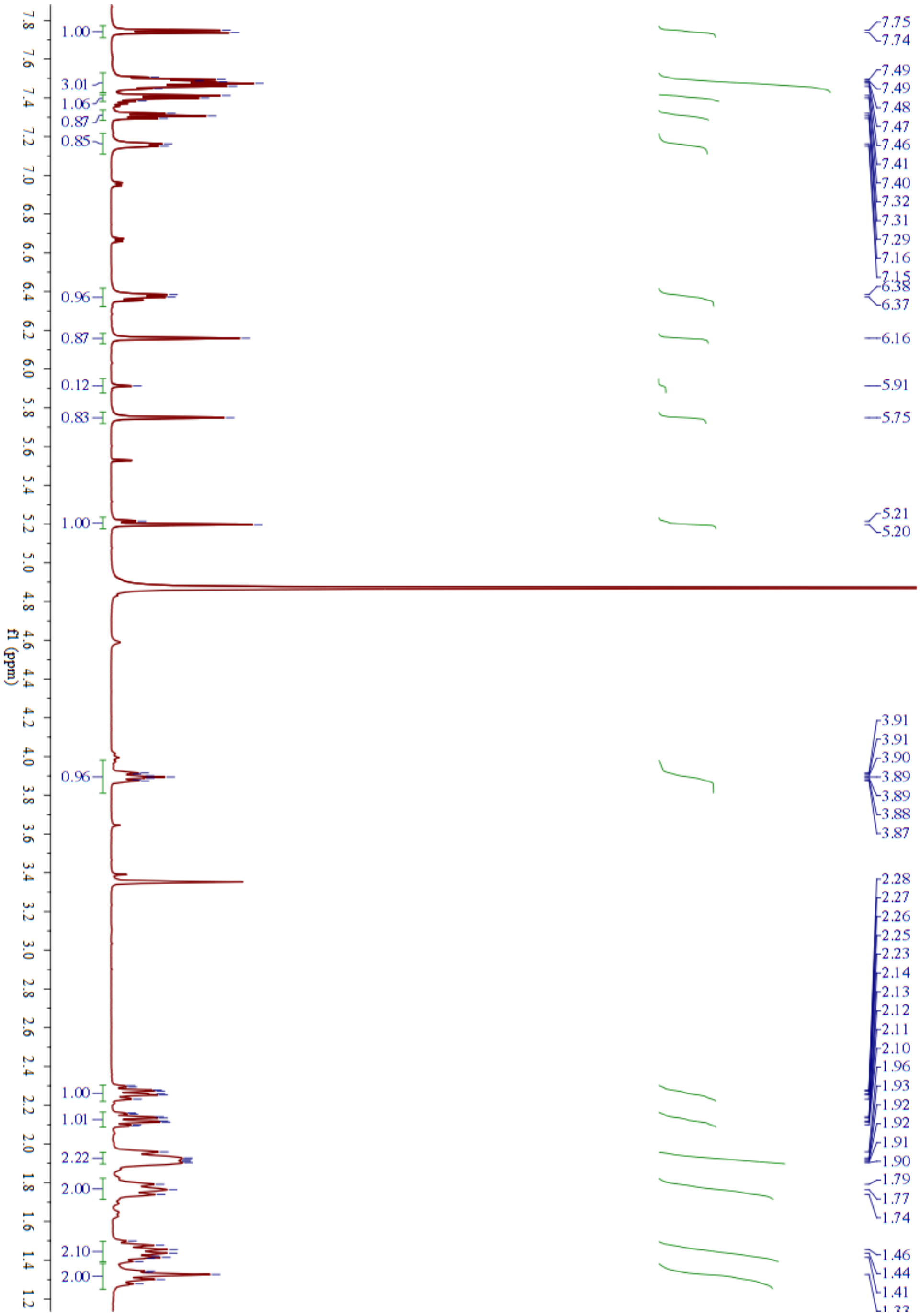

Figure S43. ^{1}H NMR for compound **12** in CD_3OD. The signal strength ratio is 1.0:0.14 between the two sets of ^{1}H NMR.

^{13}C NMR (151 MHz, CD3OD) δ 170.90, 142.95, 142.14, 139.79, 135.35, 132.03, 130.40, 130.13, 129.60, 124.30, 123.82, 123.61, 122.75, 102.35, 82.93, 65.85, 56.44, 31.27, 31.04, 27.40, 27.21, 26.62.

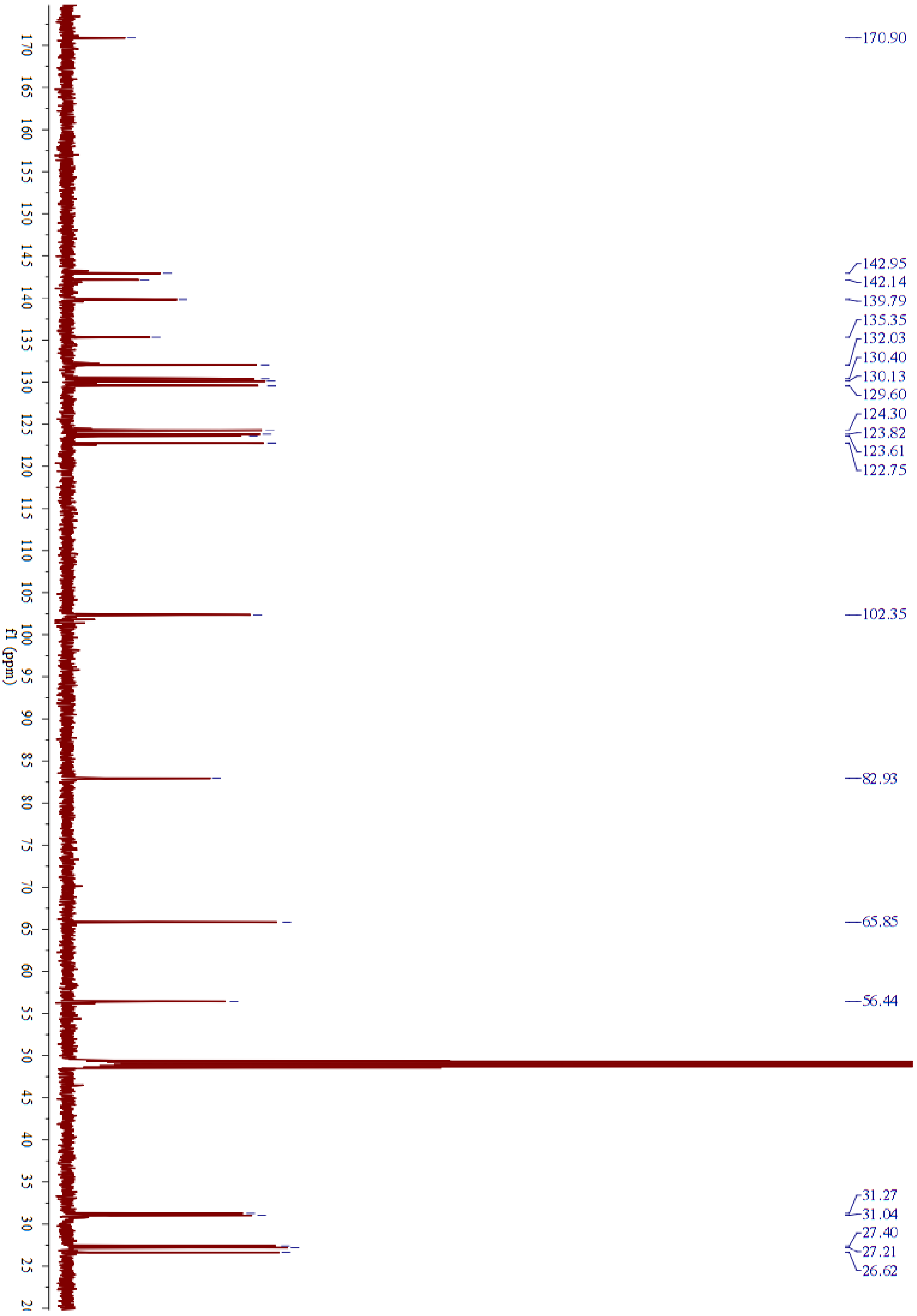

Figure S44. ^{13}C NMR for compound **12** in CD_3OD.

2-cyclohexyl-3-(3-hydroxy-1,3-dihydroisobenzofuran-1-yl)isoindolin-1-one

^{1}H NMR (600 MHz, $CDCl_3$) δ 7.84 (d, J = 7.5 Hz, 1H), 7.79 (d, J = 7.5 Hz, 1H), 7.52 (t, J = 7.4 Hz, 1H), 7.49 (t, J = 7.3 Hz, 1H), 7.42 – 7.38 (m, 5H), 7.38 – 7.31 (m, 3H), 7.27 (d, J = 7.7 Hz, 1H), 7.16 (dd, J = 10.9, 4.2 Hz, 1H), 6.78 (d, J = 7.5 Hz, 1H), 6.42 (d, J = 7.5 Hz, 1H), 6.15 – 6.08 (m, 2H), 5.89 (dd, J = 15.6, 4.5 Hz, 2H), 4.96 (d, J = 1.7 Hz, 1H), 4.91 (d, J = 1.9 Hz, 1H), 3.97 (m, 1H), 3.81 (m, 1H), 2.20 – 1.81 (m, 13H), 1.76 – 1.65 (m, 7H).

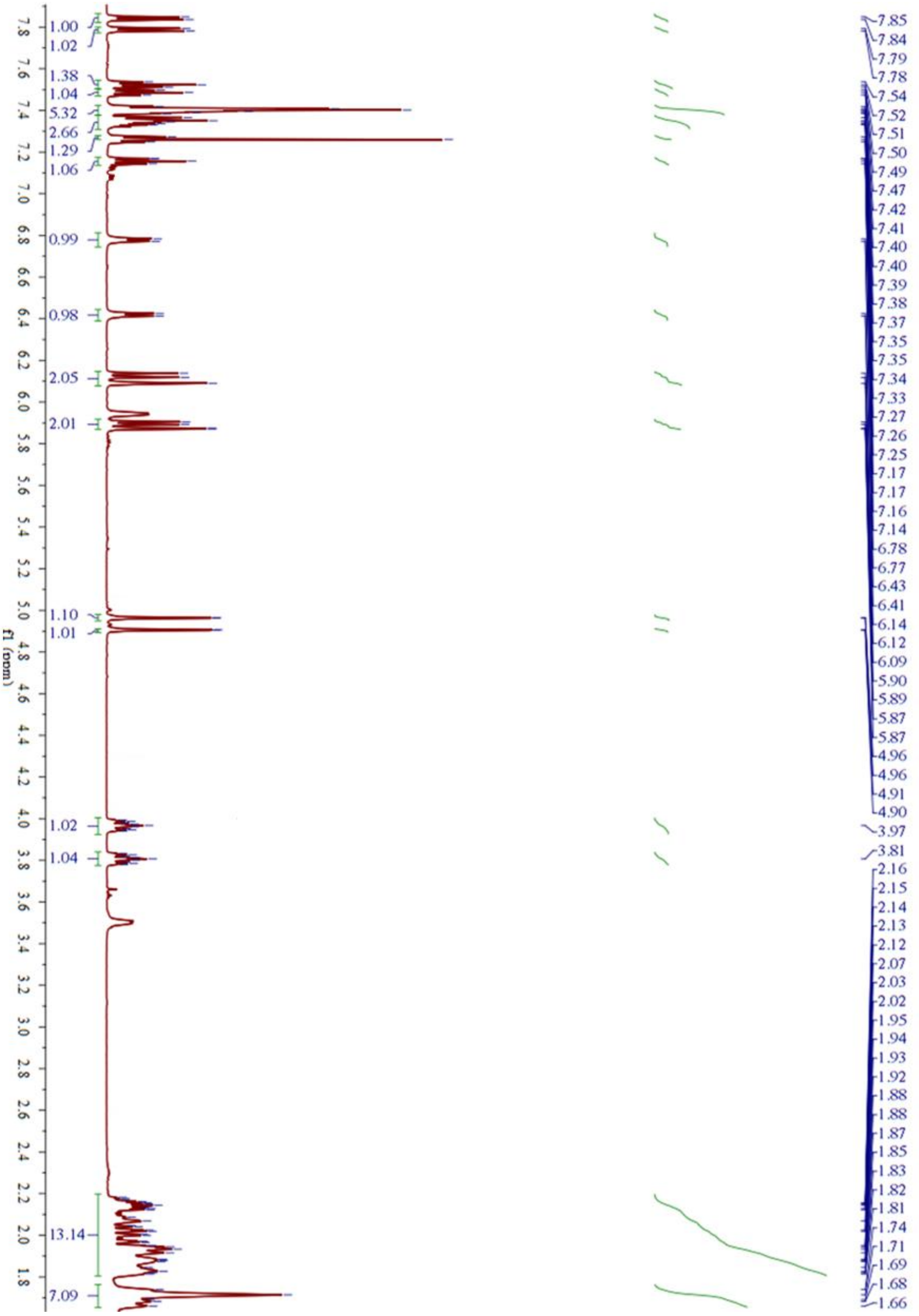

Figure S45. ^{1}H NMR for compound **12** in $CDCl_3$. Shift of the proton of –OH is 3.50 ppm. The shift of proton of –OH is 3.02 ppm. The signal strength ratio is almost 1.0:1.0 between the two sets of ^{1}H NMR.

^{13}C NMR (151 MHz, $CDCl_3$) δ 168.87, 168.29, 141.05, 140.44, 138.54, 138.24, 135.28, 134.64, 130.82, 130.23, 129.82, 129.60, 129.34, 129.08, 128.68, 124.05, 123.51, 122.31, 122.07, 121.40, 101.41, 101.25 , 82.81, 81.45, 64.26, 63.89, 55.01, 54.23, 31.64, 31.20, 30.60, 30.29, 26.50, 25.68.

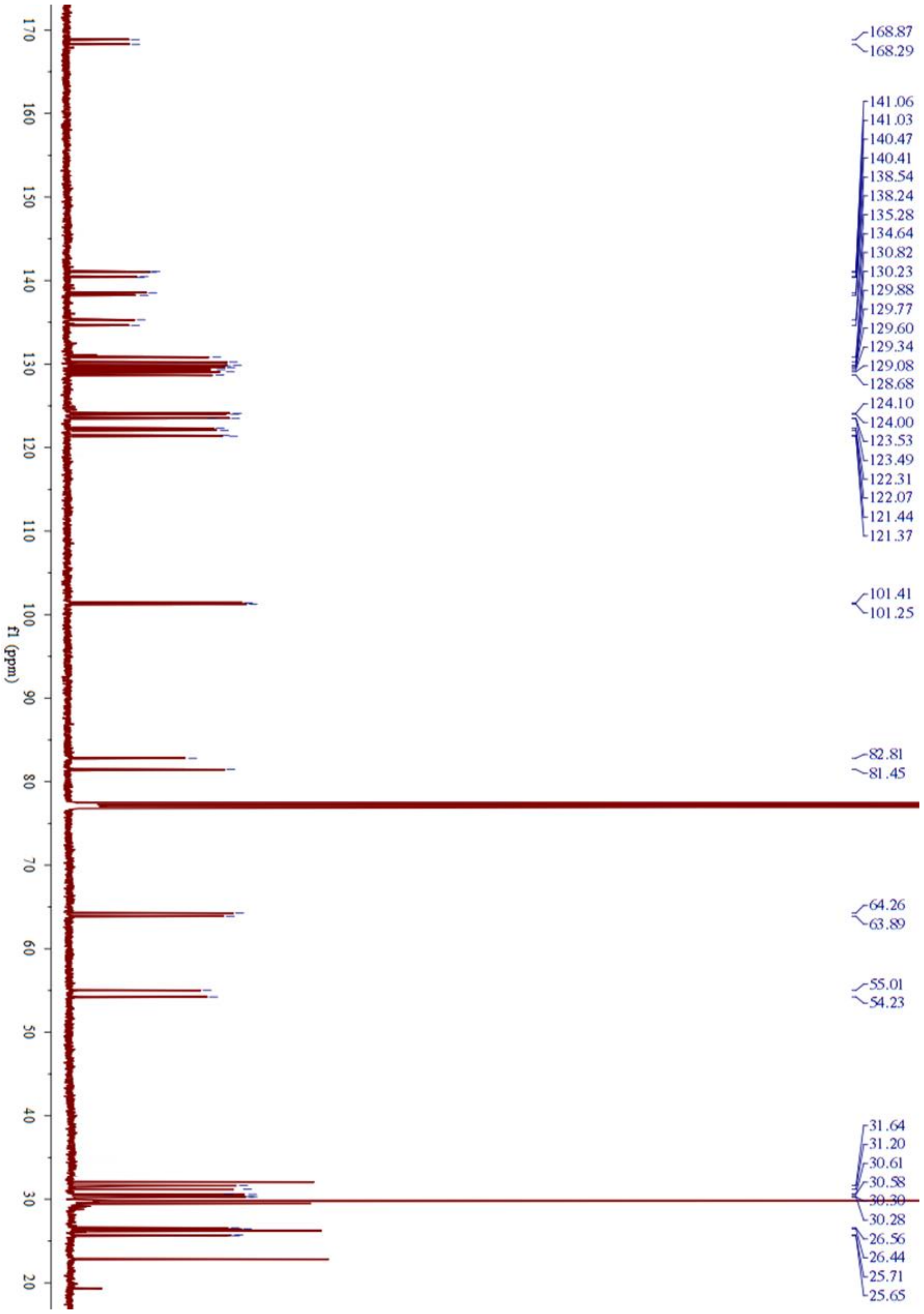

Figure S46. ^{13}C NMR for compound **12** in $CDCl_3$.

Compound 13

3-(3-hydroxy-1,3-dihydroisobenzofuran-1-yl)-2-(pentan-3-yl)isoindolin-1-one

HRMS calcd for $C_{21}H_{24}NO_3$ $[M+H^+]$: 338.1756, found 338.1749. ^1H NMR (600 MHz, CD_3OD) δ 7.76 (d, J = 7.6 Hz, 1H), 7.52 – 7.46 (m, 3H), 7.41 (d, J = 5.7 Hz, 1H), 7.32 – 7.26 (m, 2H), 6.27 (d, J = 6.2 Hz, 1H), 6.11 (s, 1H), 5.72 (s, 1H), 5.09 (d, J = 16.0 Hz, 1H), 3.87 (dt, J = 14.0, 7.1 Hz, 1H), 2.11 – 2.04 (m, 1H), 2.01 – 1.96 (m, 2H), 1.87 – 1.80 (m, 1H), 0.98 (d, J = 15.3 Hz, 3H×2).

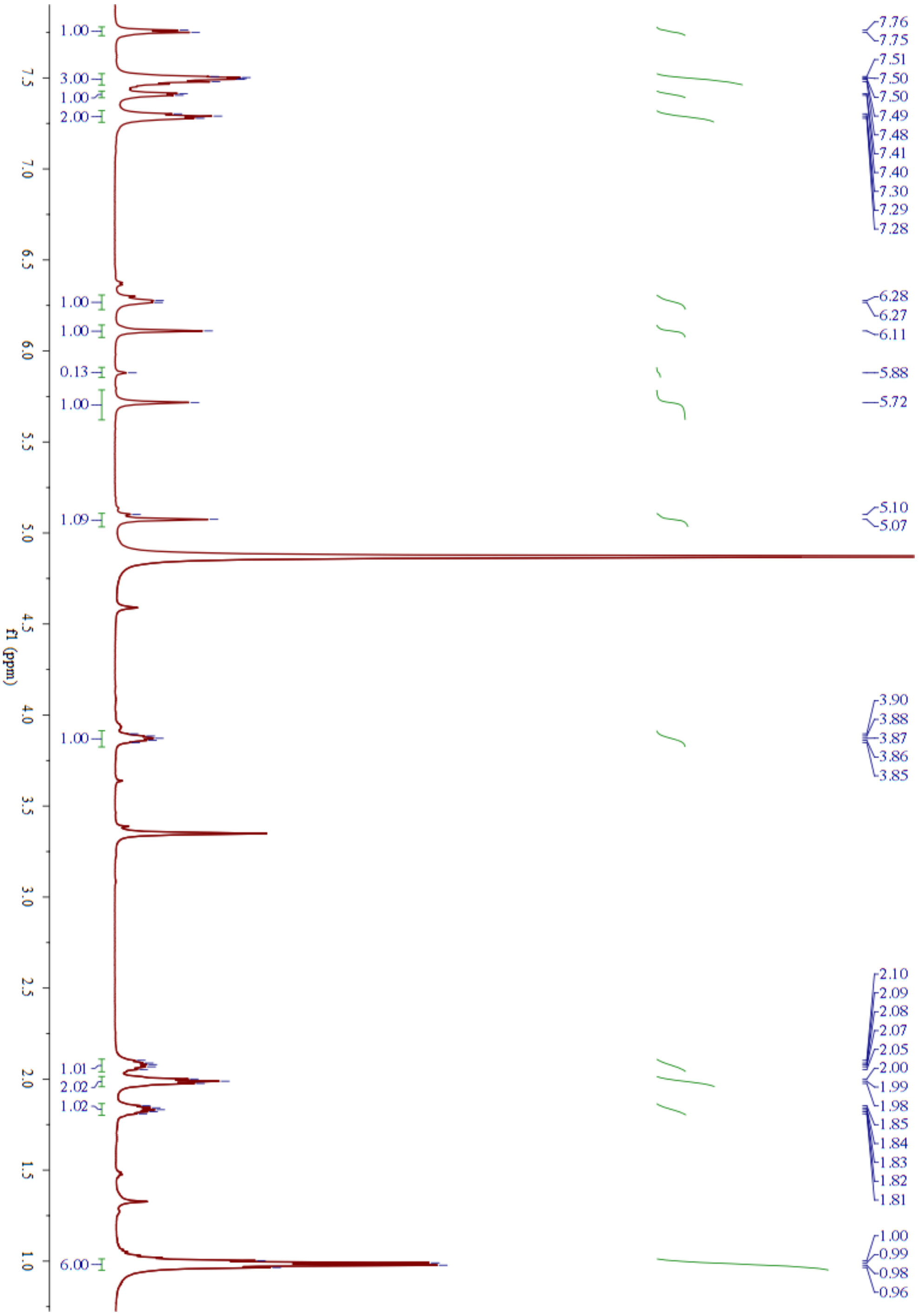

Figure S47. ^1H NMR for compound **13** in CD_3OD. The signal strength ratio is 1.0:0.13 between the two sets of ^1H NMR.

^{13}C NMR (151 MHz, CD3OD) δ 171.61, 142.94, 142.27, 140.01, 135.04, 132.07, 130.46, 130.15, 129.64, 124.34, 123.77, 122.81, 102.31, 82.66, 66.74, 60.54, 27.06, 26.73, 11.99, 11.71.

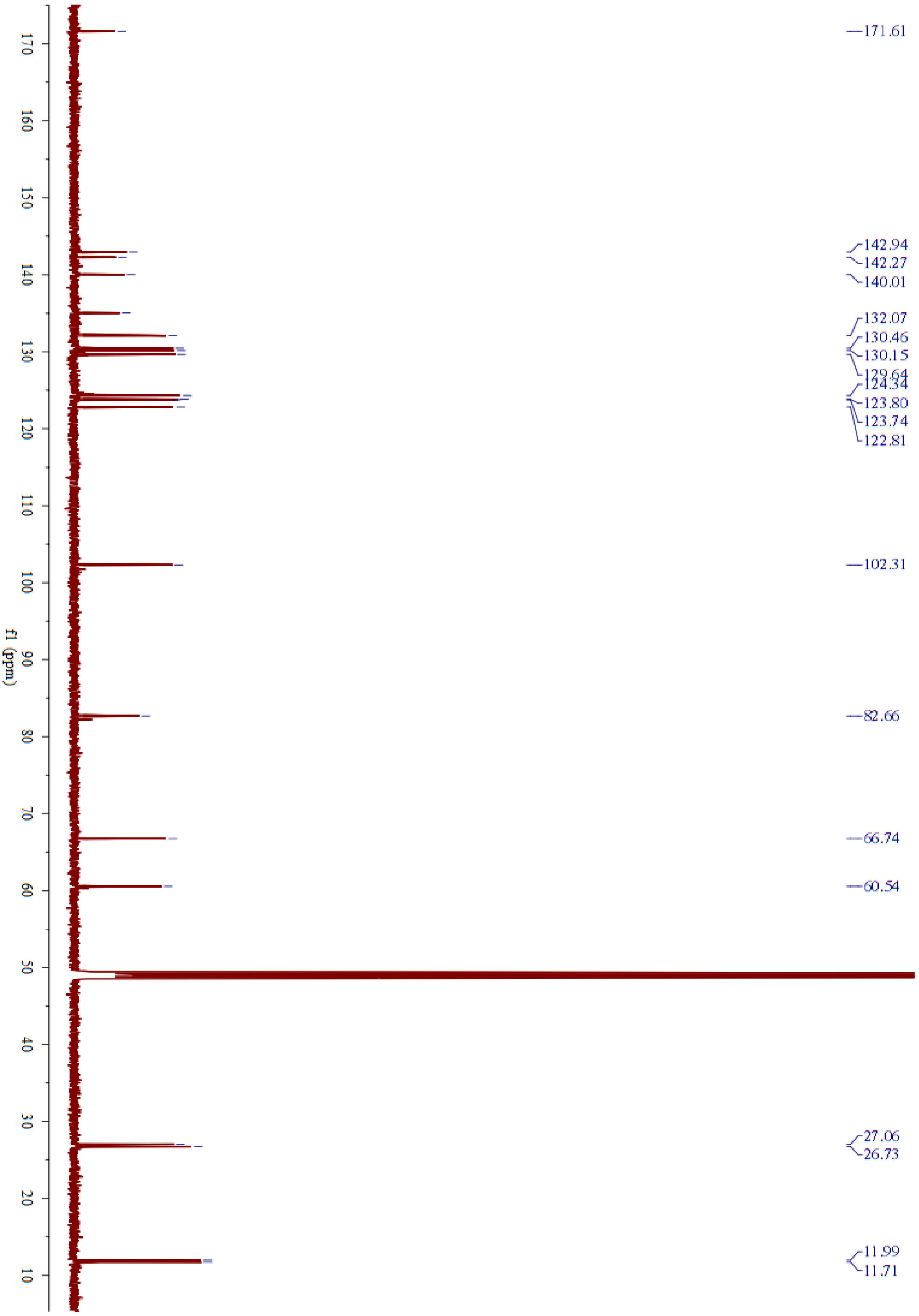

Figure S48. ^{13}C NMR for compound **13** in CD_3OD.

3-(3-hydroxy-1,3-dihydroisobenzofuran-1-yl)-2-(pentan-3-yl)isoindolin-1-one

^{1}H NMR (600 MHz, $CDCl_3$) δ 7.69 (d, J = 7.5 Hz, 1H), 7.64 (d, J = 7.5 Hz, 1H), 7.39 (d, J = 7.3 Hz, 1H), 7.35 (t, J = 7.3 Hz, 1H), 7.26 (dd, J = 9.0, 7.1 Hz, 6H), 7.22 – 7.19 (m, 1H), 7.12 – 7.08 (m, 2H), 7.00 (t, J = 7.5 Hz, 1H), 6.69 (d, J = 7.3 Hz, 1H), 6.20 (d, J = 7.5 Hz, 1H), 5.97 (d, J = 11.9 Hz, 1H), 5.88 (s, 1H), 5.78 (d, J = 5.3 Hz, 1H), 5.67 (d, J = 7.6 Hz, 1H), 5.63 (s, 1H), 4.74 (d, J = 1.4 Hz, 1H), 4.65 (d, J = 1.7 Hz, 1H), 3.73 (m, 1H), 3.62 (m, 1H), 3.42 (d, J = 6.9 Hz, 1H), 1.94 (m, 1H), 1.89 – 1.82 (m, 2H), 1.79 (m, 2H), 1.66 (m, 1H), 1.56 (dd, J = 14.1, 7.5 Hz, 2H), 0.89 (t, J = 7.4 Hz, 3H), 0.83 (t, J = 7.4 Hz, 3H), 0.75 (t, J = 7.4 Hz, 3H). 0.71 (t, J = 7.4 Hz, 3H)

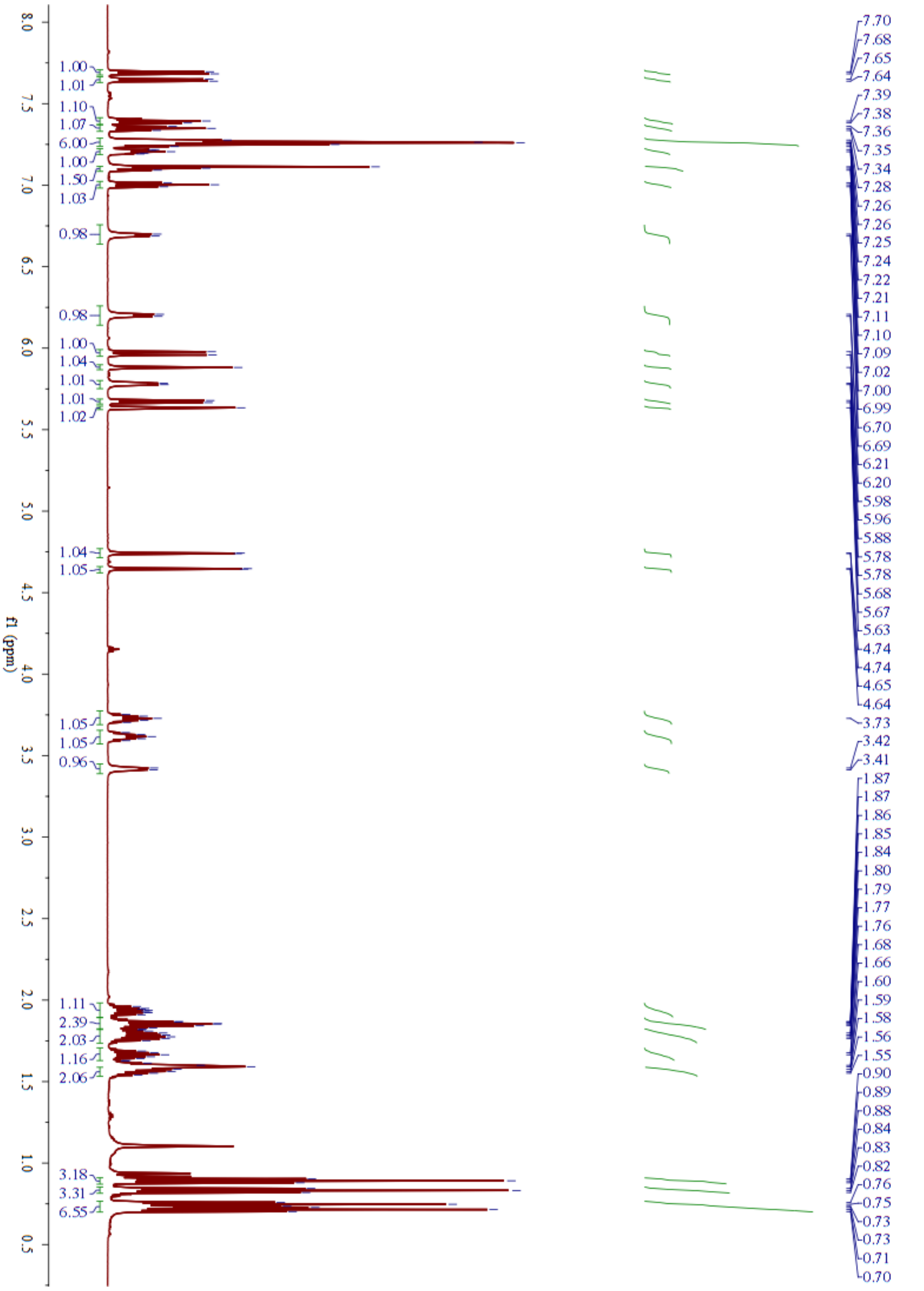

Figure S49. ^{1}H NMR for compound **13** in $CDCl_3$. Shift of proton of hydroxyl group is 3.42 ppm. The signal strength ratio is almost 1.0:1.0 between the two sets of ^{1}H NMR.

^{13}C NMR (151 MHz, $CDCl_3$) δ 169.55, 169.01, 141.08, 140.62, 138.66, 138.47, 135.08, 134.29, 130.87, 130.24, 129.88, 129.68, 129.32, 129.11, 128.67, 124.23, 124.02, 123.56, 122.27, 122.01, 121.39, 101.28, 82.50, 81.26, 65.09, 64.75, 59.12, 58.68, 26.85, 26.25, 25.90, 11.78.

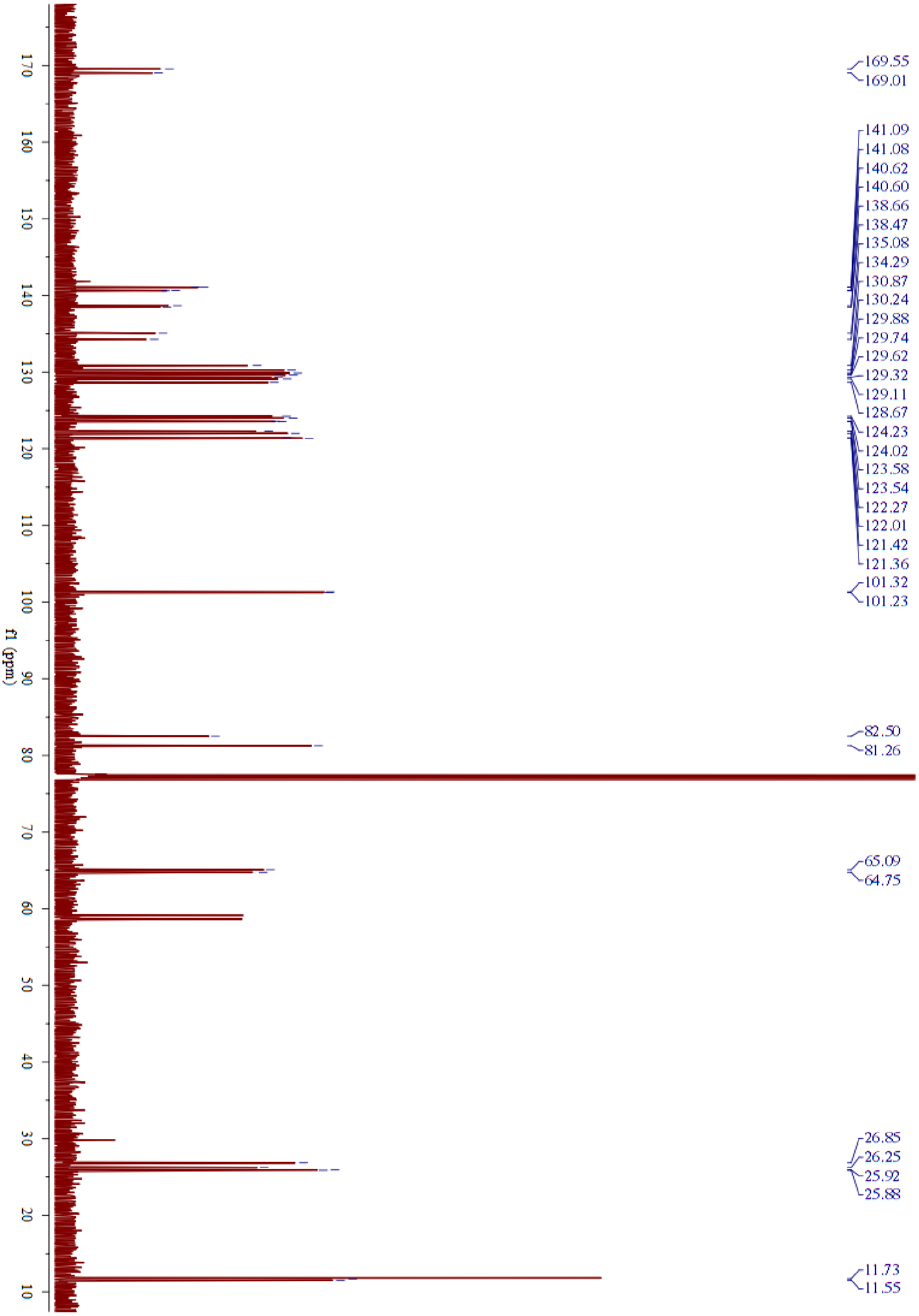

Figure S50. ^{13}C NMR for compound **13** in $CDCl_3$.

Compound 14

3-(3-hydroxy-1,3-dihydroisobenzofuran-1-yl)-2-phenylisoindolin-1-one

HRMS calcd for $C_{22}H_{18}NO_3$ [M+H$^+$]: 344.1287 found: 344.1280. ^{1}H NMR (600 MHz, CD3OD) δ 7.87 (d, *J* = 7.6 Hz, 1H), 7.59 (t, *J* = 7.5 Hz, 1H), 7.53 – 7.47 (m, 3H), 7.45 (t, *J* = 8.5 Hz, 4H), 7.38 (t, *J* = 7.4 Hz, 1H), 7.34 (t, *J* = 7.1 Hz, 1H), 7.29 (d, *J* = 7.5 Hz, 1H), 6.88 (d, *J* = 7.5 Hz, 1H), 6.82 (d, *J* = 7.6 Hz, 1H), 5.92 (s, 1H), 5.87 (s, 1H), 5.25 (d, *J* = 1.8 Hz, 1H).

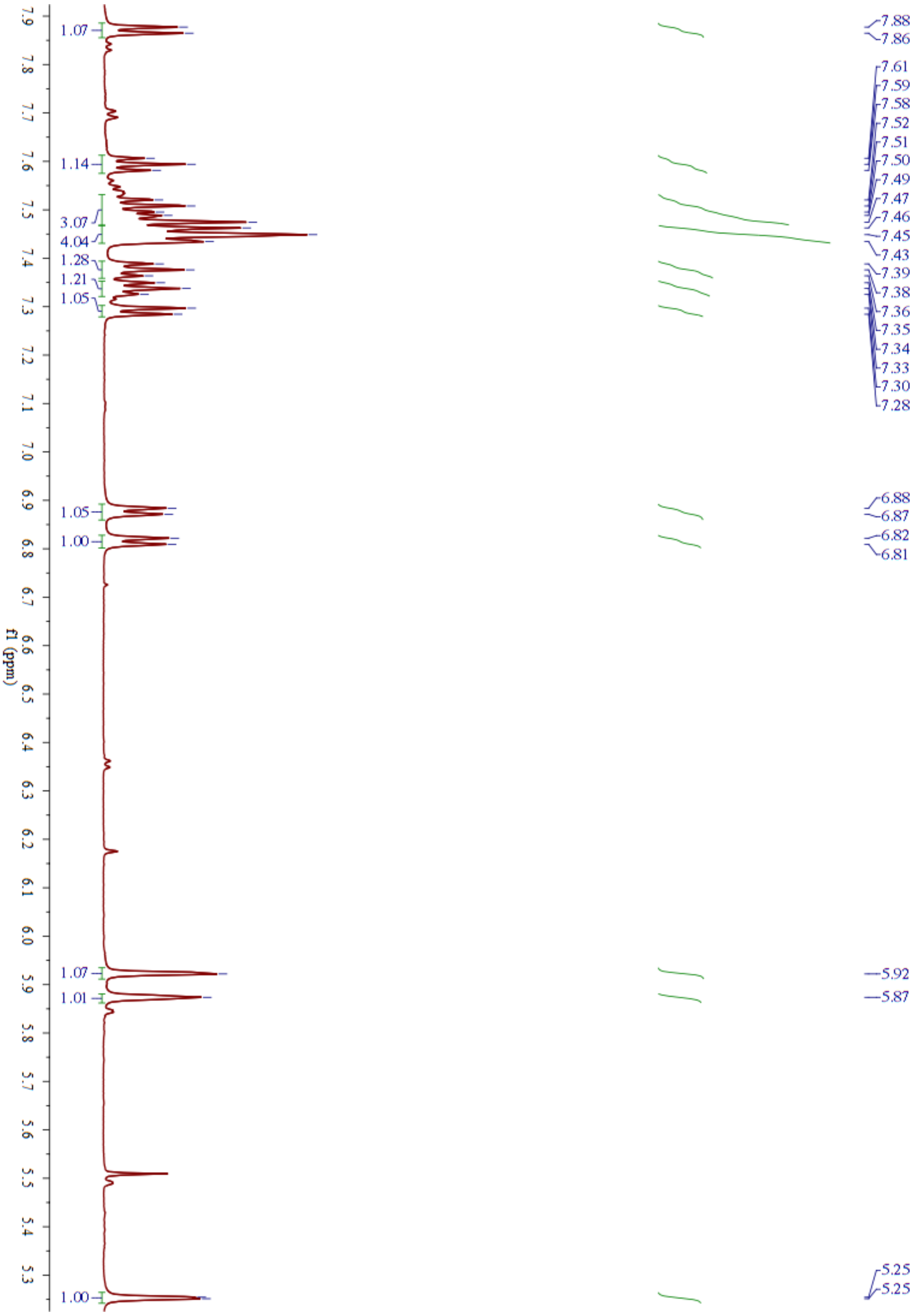

Figure S51. ^{1}H NMR for compound **14** in CD_3OD. The signal strength ratio is almost 1.0:0.14 between the two sets of ^{1}H NMR.

^{13}C NMR (151 MHz, CD3OD) δ 169.69, 142.42, 138.89, 138.08, 134.30, 133.16, 130.22, 129.94, 127.60, 126.77, 124.61, 124.23, 122.48, 102.37, 83.16, 66.77, 33.04, 30.71, 23.70.

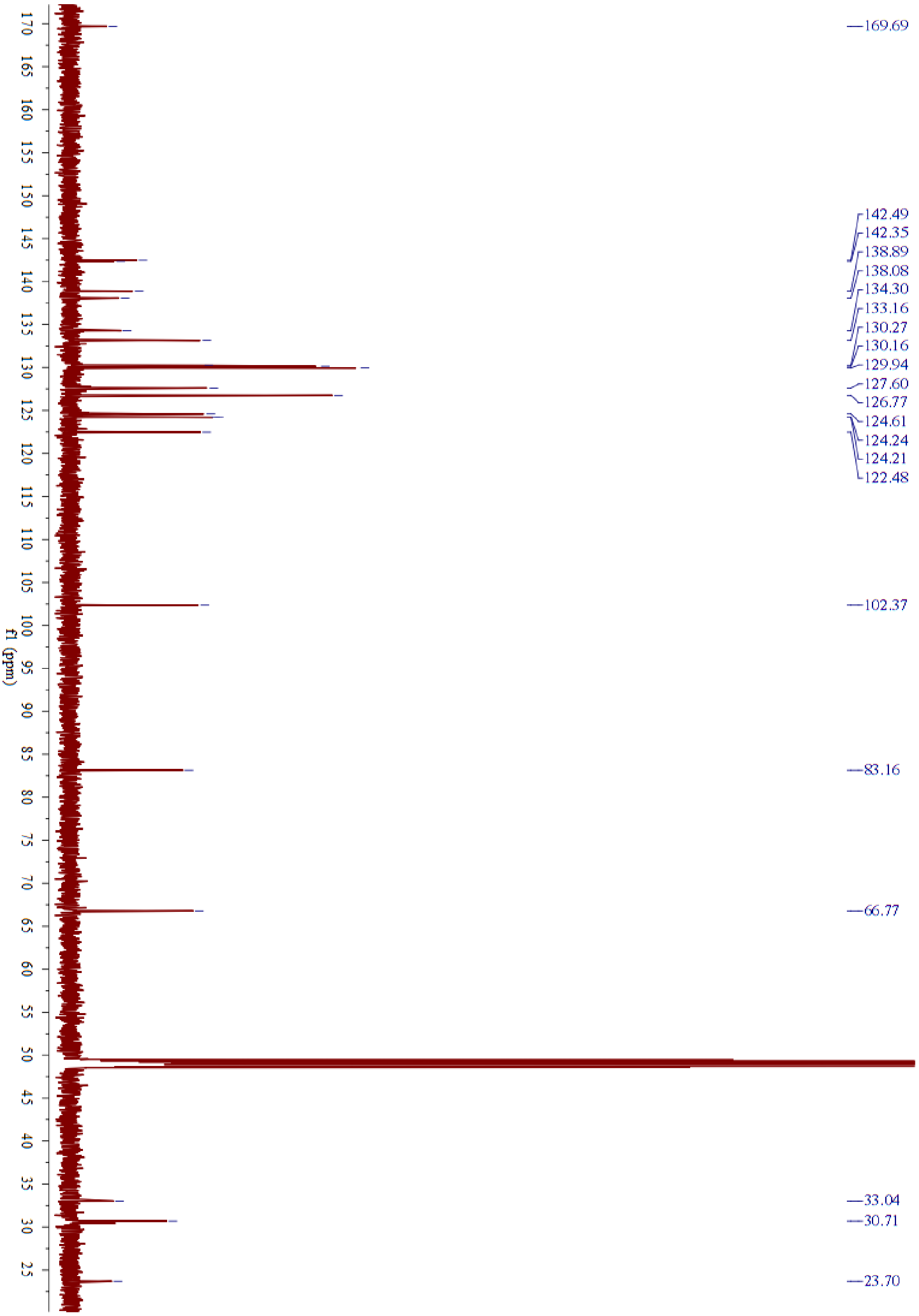

Figure S52. ^{13}C NMR for compound **14** in CD_3OD.

3-(3-hydroxy-1,3-dihydroisobenzofuran-1-yl)-2-phenylisoindolin-1-one

^{1}H NMR (600 MHz, $CDCl_3$) δ 7.91 (d, *J* = 7.6 Hz, 1H), 7.86 (d, *J* = 7.5 Hz, 1H), 7.49 (dd, *J* = 9.1, 5.8 Hz, 2H), 7.43 – 7.39 (m, 2H), 7.38 – 7.30 (m, 5H), 7.22 (ddd, *J* = 10.0, 8.9, 4.4 Hz, 5H), 6.85 (d, *J* = 7.2 Hz, 1H), 6.75 (d, *J* = 7.6 Hz, 1H), 6.56 (d, *J* = 7.2 Hz, 1H), 6.02 (s, 1H), 5.83 (d, *J* = 1.9 Hz, 1H), 5.59 (d, *J* = 1.8 Hz, 1H), 5.55 (d, *J* = 2.3 Hz, 1H).

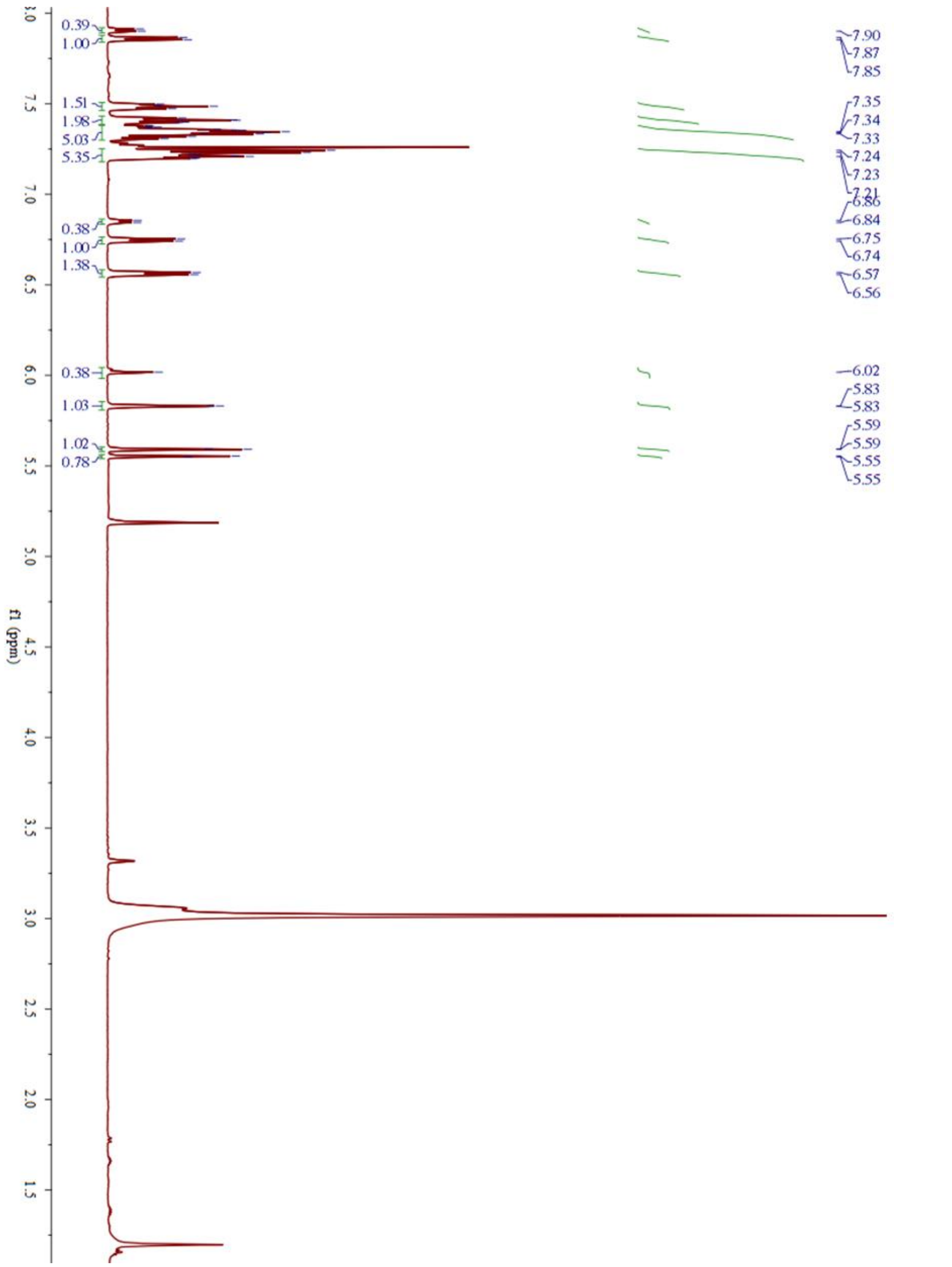

Figure S53. ^{1}H NMR for compound **14** in $CDCl_3$. Proton Shift of –OH is 3.37 ppm. The signal strength ratio is almost 1.0:0.38 between the two sets of ^{1}H NMR.

^{13}C NMR (151 MHz, $CDCl_3$) δ 168.02, 140.74, 140.46, 137.13, 136.66, 133.41, 133.19, 131.97, 131.83, 129.68, 129.34, 129.19, 128.94, 128.58, 128.44, 126.90, 126.44, 125.66, 125.44, 124.47, 124.19, 123.56, 123.25, 122.70, 121.10, 101.08 , 82.40 , 81.31, 65.52 , 65.34.

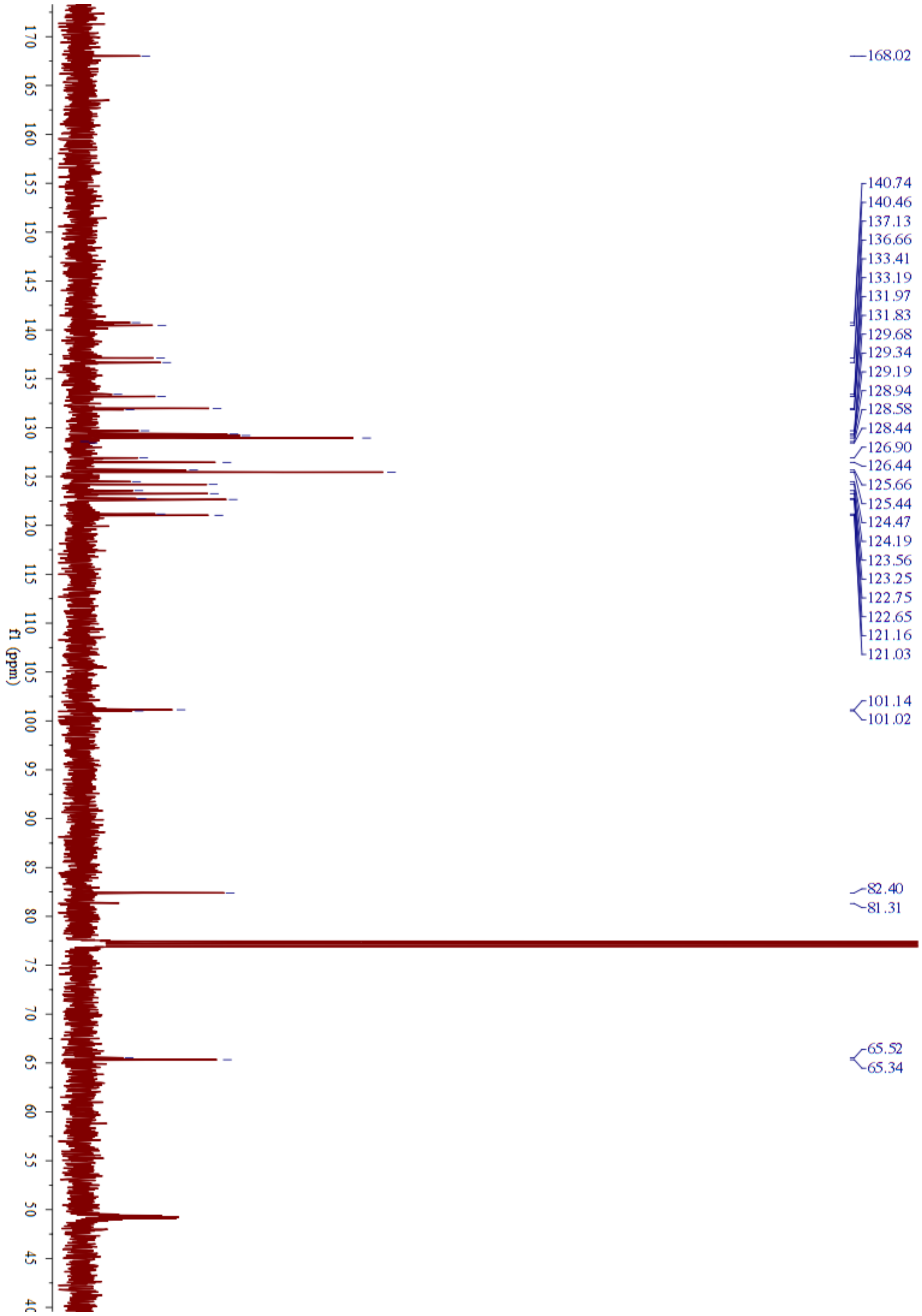

Figure S54. ^{13}C NMR for compound **14** in $CDCl_3$.

NMR data of compounds 15, 16 and 17

The solid NMR were recorded on InfinityPlus 300 using Cross Polarization (CP), rotation rate is10 kHz.

The NMR data in $CDCl_3$ were recorded in Bruck AV400 or DRX500.

IR was recorded in Tensor 27 equipment.

Compound **15**. $C_{16}H_{20}N_2O_3$, mp. 136-138 °C. $[\alpha]_D$ +6.34 ° (*c*, 0.00815 g/ml, EtOAc). HRMS calcd for $C_{16}H_{21}N_2O_3$ [M+H$^+$]: 289.1552. Found : 289.1559. ^{1}H NMR (500 MHz, $CDCl_3$),δ8.20 (brs, 1H), 7.51 (d, *J*=7.7 Hz, 1H), 7.31(d, *J*=8.0 Hz, 1H), 7.17 (m, 1H), 7.08 (m, 1H), 4.03 (t,*J*=6.4 Hz, 1H), 3.84 (dd, *J*=4.9, 8.7 Hz, 1H), 3.72(s,3H), 3.61 (d, *J*=6.9 Hz, 1H), 3.14 (dd, *J*=8.9, 15.8 Hz, 1H), 2.96 (dd, *J*=4.9, 15.8 Hz, 1H), 2.49 (s, 3H), 1.44 (d, *J*=6.2 Hz, 3H). ^{13}C NMR (100 MHz, $CDCl_3$)δ172.8, 136.2, 132.1, 126.6, 121.7,119.3, 118.1, 110.8, 107.6, 69.1, 65.5, 59.4, 51.9, 38.7, 20.9, 20.1.
Solid ^{13}C NMR: 18.2, 19.6, 21.8, 38.1, 52.2, 53.7, 56.0, 68.2, 66.5, 70.7,104.9, 107.4, 111.3, 112.6, 116.7, 119.9, 121.6,127.1, 128.1, 134.6, 136.4, 138.5 and 175.3 ppm. Total 23 signals.

Compound **16**, mp. 92-94 °C. $[\alpha]_D$+6.78 ° (*c*, 0.00885g/ml, EtOAc). HRMS calcd for $C_{16}H_{21}N_2O_3$ [M+H$^+$]: 289.1552. Found : 289.1558. ^{1}H NMR (400 MHz, $CDCl_3$) δ 8.31 (brs, 1H), 7.54(d, *J*=7.7Hz, 1H), 7.35(d, *J*=7.7Hz, 1H), 7.18(m, 1H), 7.13(m, 1H), 3.98(m, 2H), 3.78(s, 3H), 3.63(d, *J*=7.6Hz, 1H), 3.17(dd, *J*=9.0, 16.0Hz, 1H), 3.01(dd, *J*=5.2, 16.0Hz, 1H), 2.49(s, 3H), 1.31(d, *J*=6.1Hz, 3H), ^{13}C NMR (100 MHz, $CDCl_3$): δ172.7, 136.2, 130.6, 126.5, 121.9, 119.4, 118.1, 110.9, 107.8, 67.4, 66.3, 57.6, 52.1, 38.3, 20.0, 18.6.
Solid ^{13}C NMR: 17.7, 21.0, 37.1, 39.6, 55.2, 56.2, 67.3, 68.7, 70.1, 108.6, 109.7, 111.7, 113.0, 119.0, 121.7, 127.7, 130.6, 137.6, 172.3, 175.0 ppm. Total 20 signals.

Compound **17**, mp. 166-167.5 °C. $[\alpha]_D$ +0.41 ° (*c*, 0.00820 g/ml, EtOAc). HRMS calcd. for $C_{21}H_{23}N_2O_3$ [M+H$^+$]: 351.1709. Found: 351.1717. ^{1}H NMR (400 MHz, $CDCl_3$), δ7.36 (m, 6H), 6.99 (m, 2H),6.91(m, 1H), 6.45 (brs, 1H), 5.22 (d,*J*=3.5 Hz, 1H), 4.45 (d, *J*=2.0 Hz, 1H), 3.92(t, *J*=4.3 Hz, 1H), 3.52(s,3H), 3.13 (d, *J*=3.9 Hz, 2H), 2.73(s, 3H). ^{13}C NMR (100 MHz, $CDCl_3$): δ172.9, 140.7, 135.5, 130.3, 128.3,127.6, 125.9,121.5, 118.9, 117.7, 110.5, 108.3, 70.8, 64.5, 61.6, 51.5, 40.1, 23.8. $C_{21}H_{22}N_2O_3$; MS (EI): 349 (M+H).
Solid ^{13}C NMR: 25.5, 41.8, 51.5, 62.8, 64.1 68.7, 109.1, 113.0, 116.3, 120.2, 123.0, 124.7, 126.6, 127.8, 129.6, 131.6, 135.6, 136.3, 141.2, 171.7 ppm.

Compound **15**

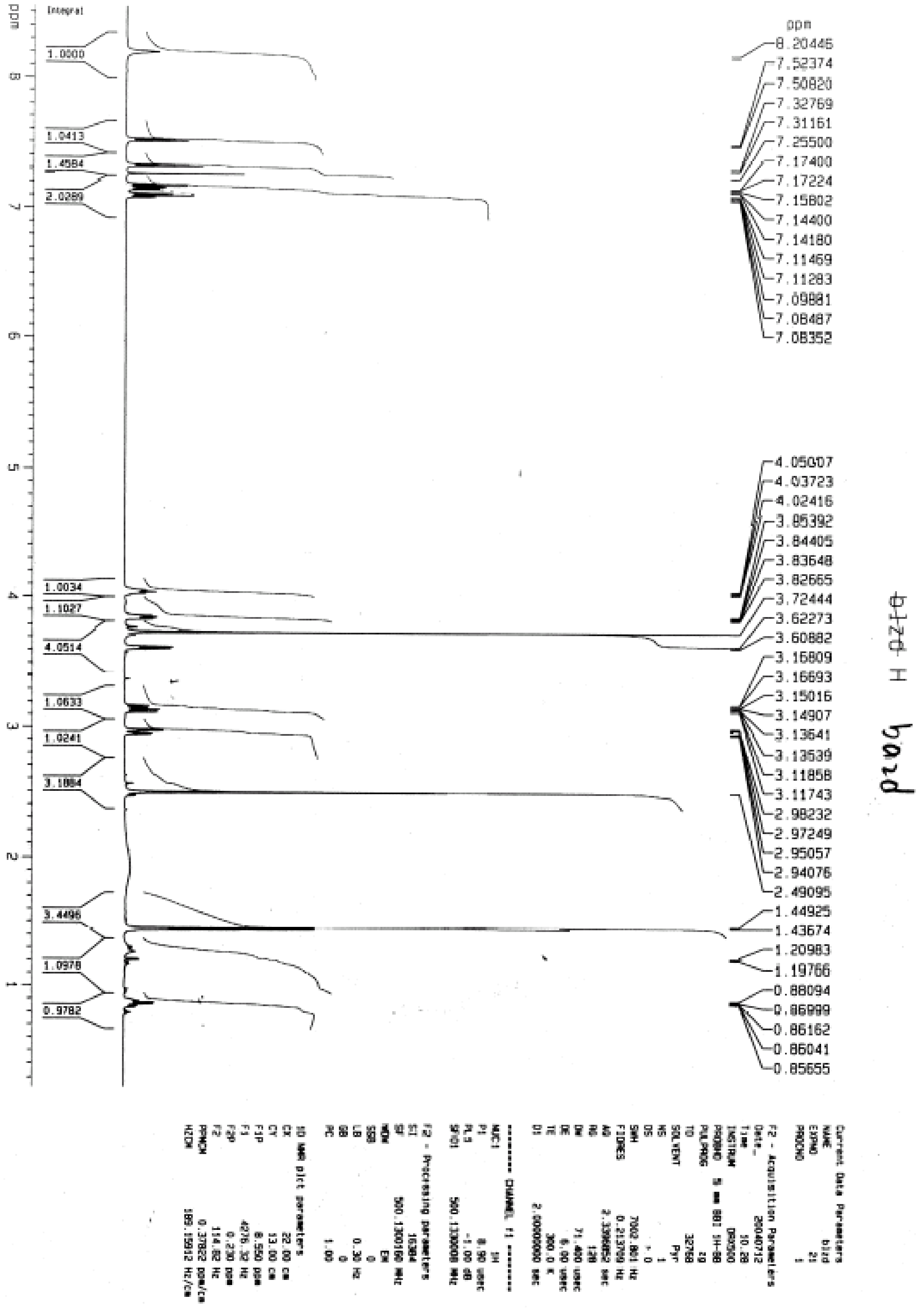

Figure S55. ^{1}H NMR for compound **15** in $CDCl_3$.

Compound **15**

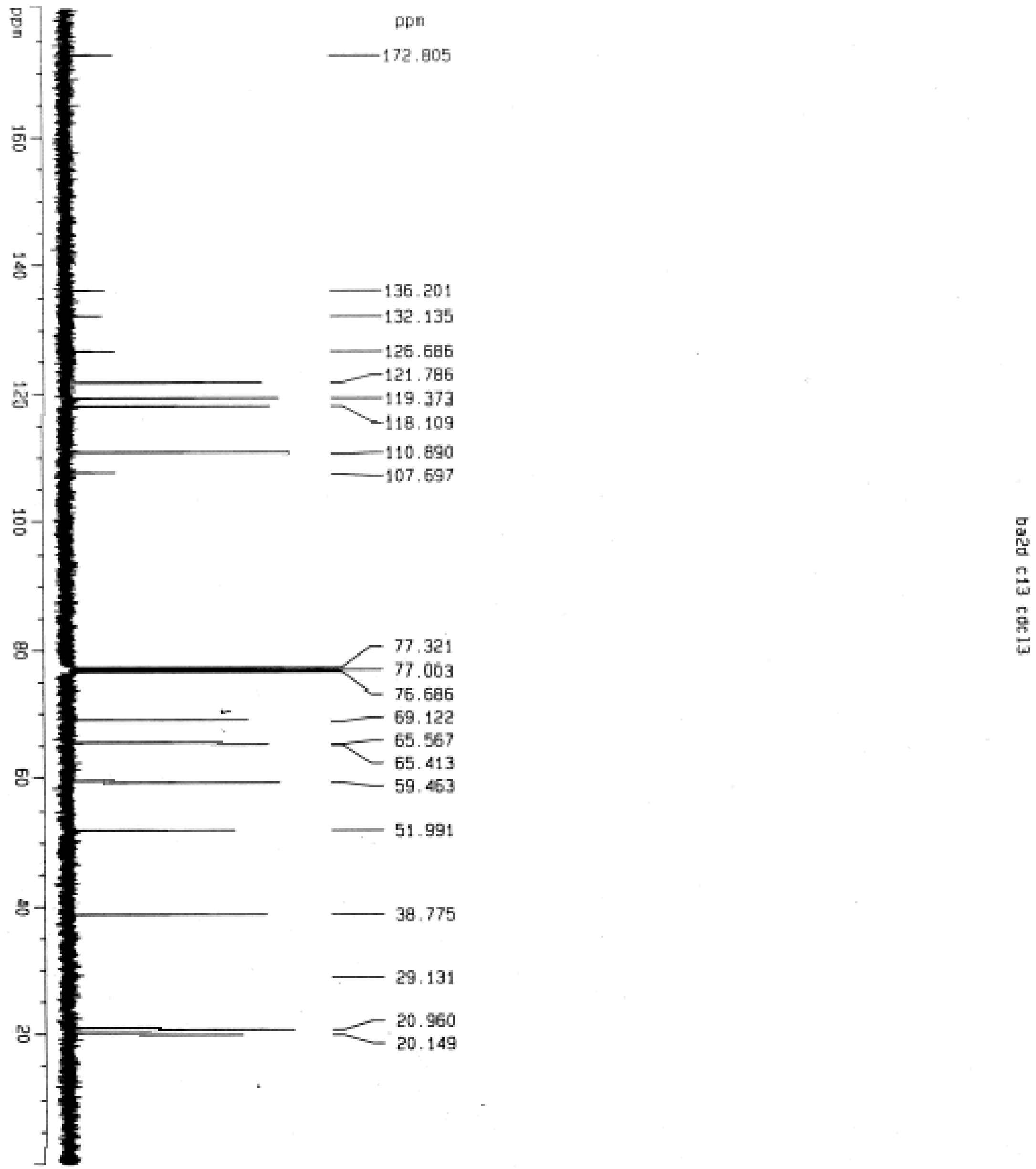

Figure S56. ^{13}C NMR for compound **15** in $CDCl_3$.

Compound **15**

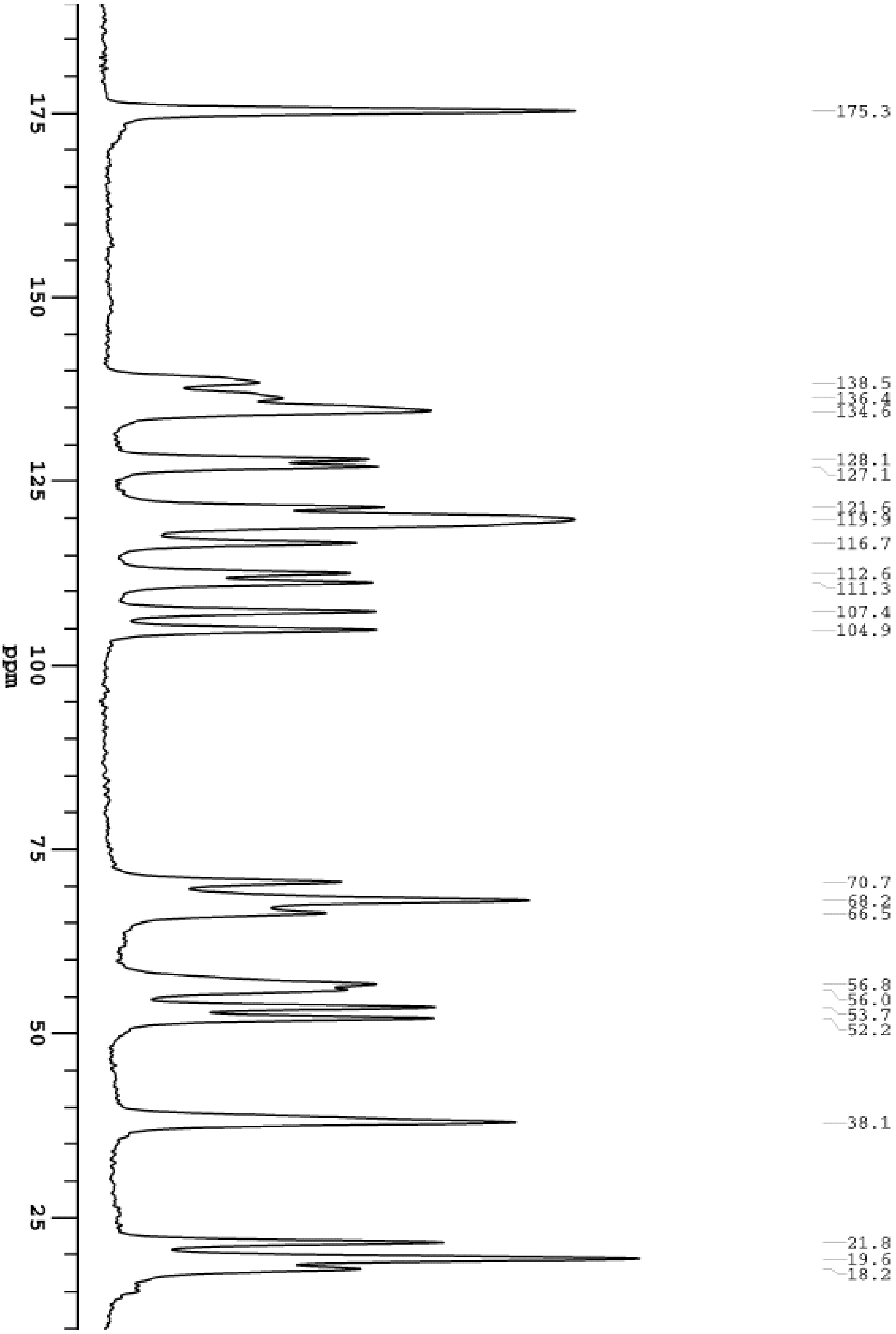

Figure S57. ^{13}C NMR for compound **15** in solid state (^{13}C cross-polarized magic-angle spinning (CP-MAS) NMR).

Compound **16**

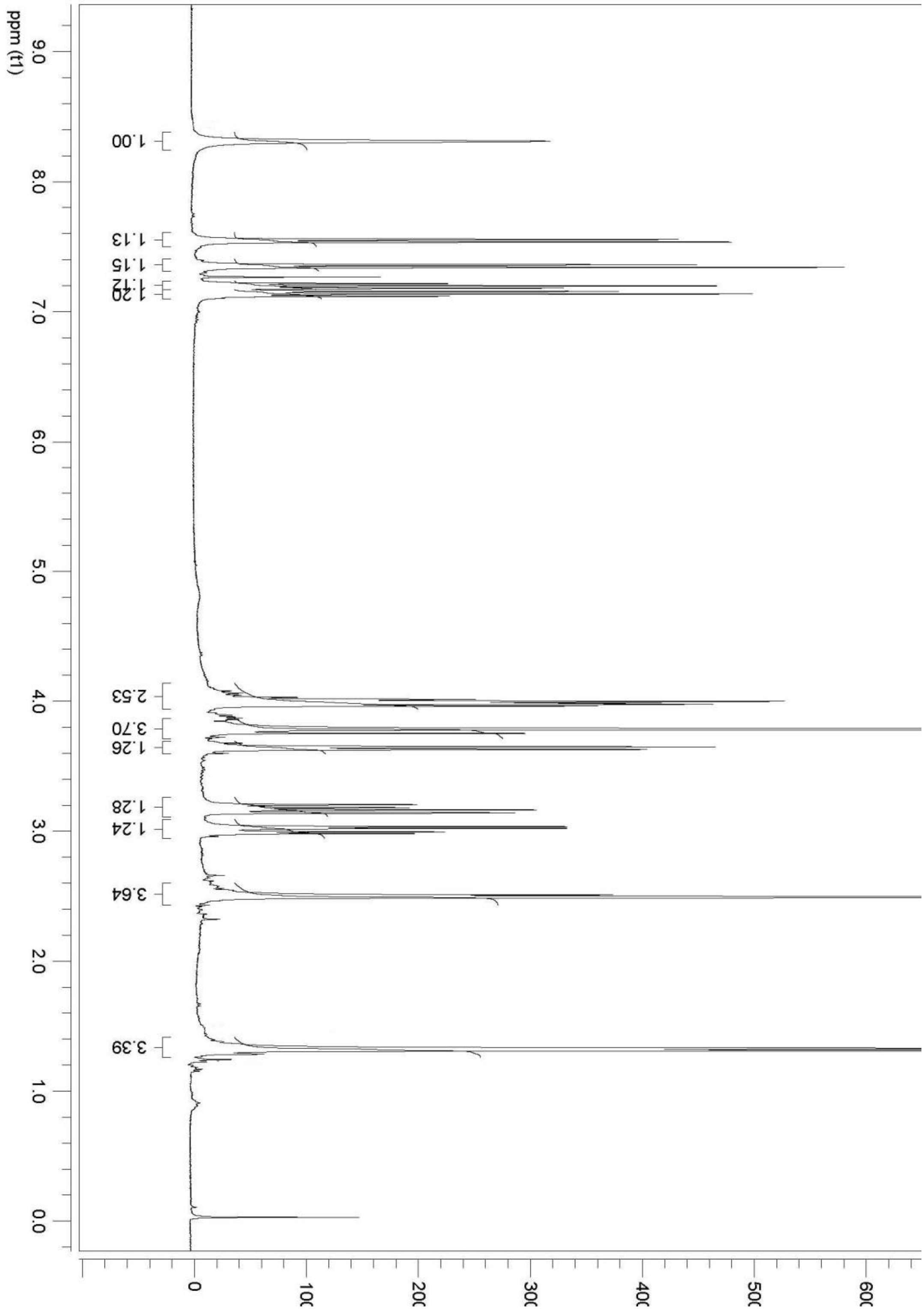

Figure S58. The ^{1}H NMR spectrum for compound **16** in CD_3OD

Compound **16**.

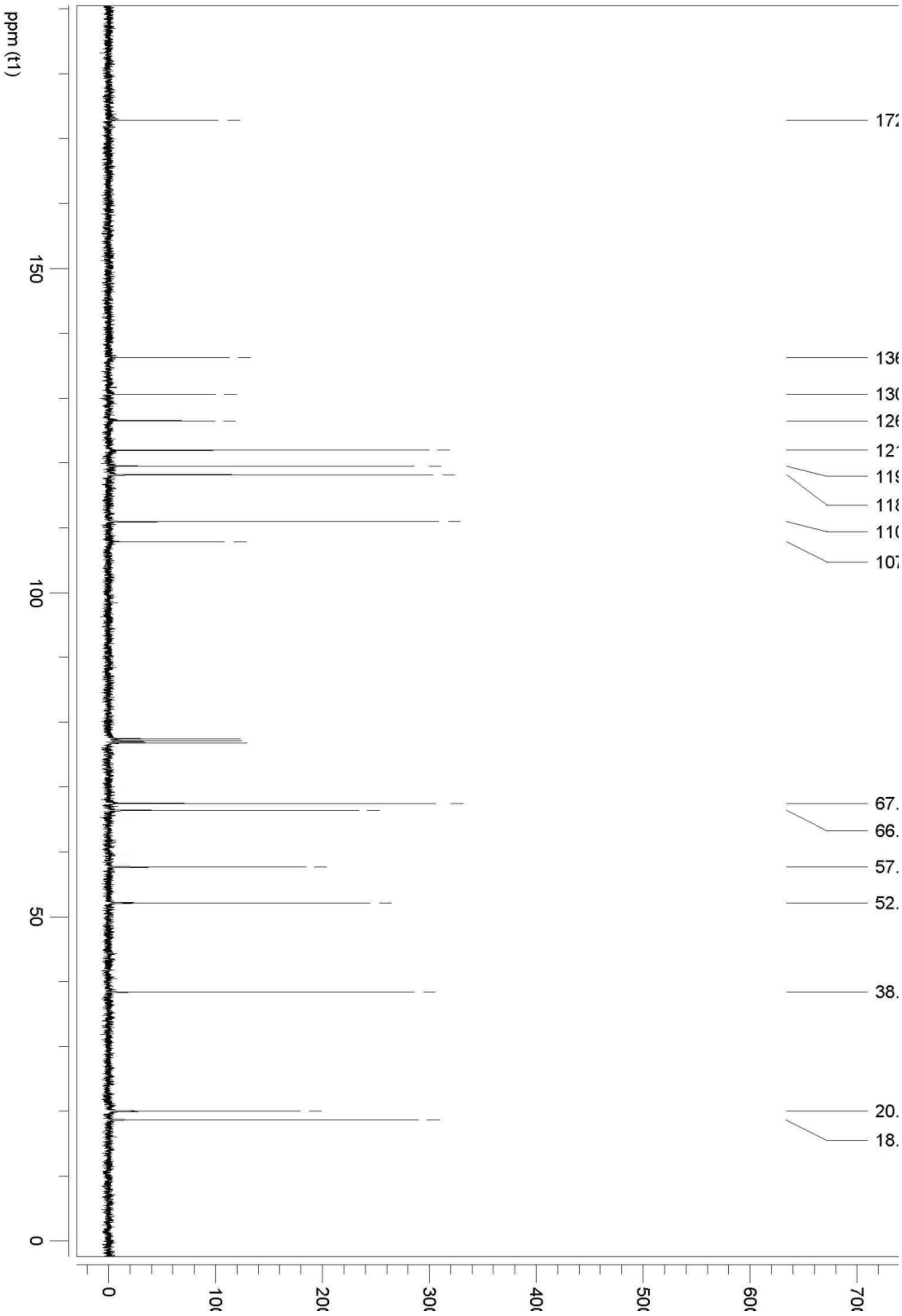

Figure S59. The ^{13}C NMR for compound **16** in CD_3OD.

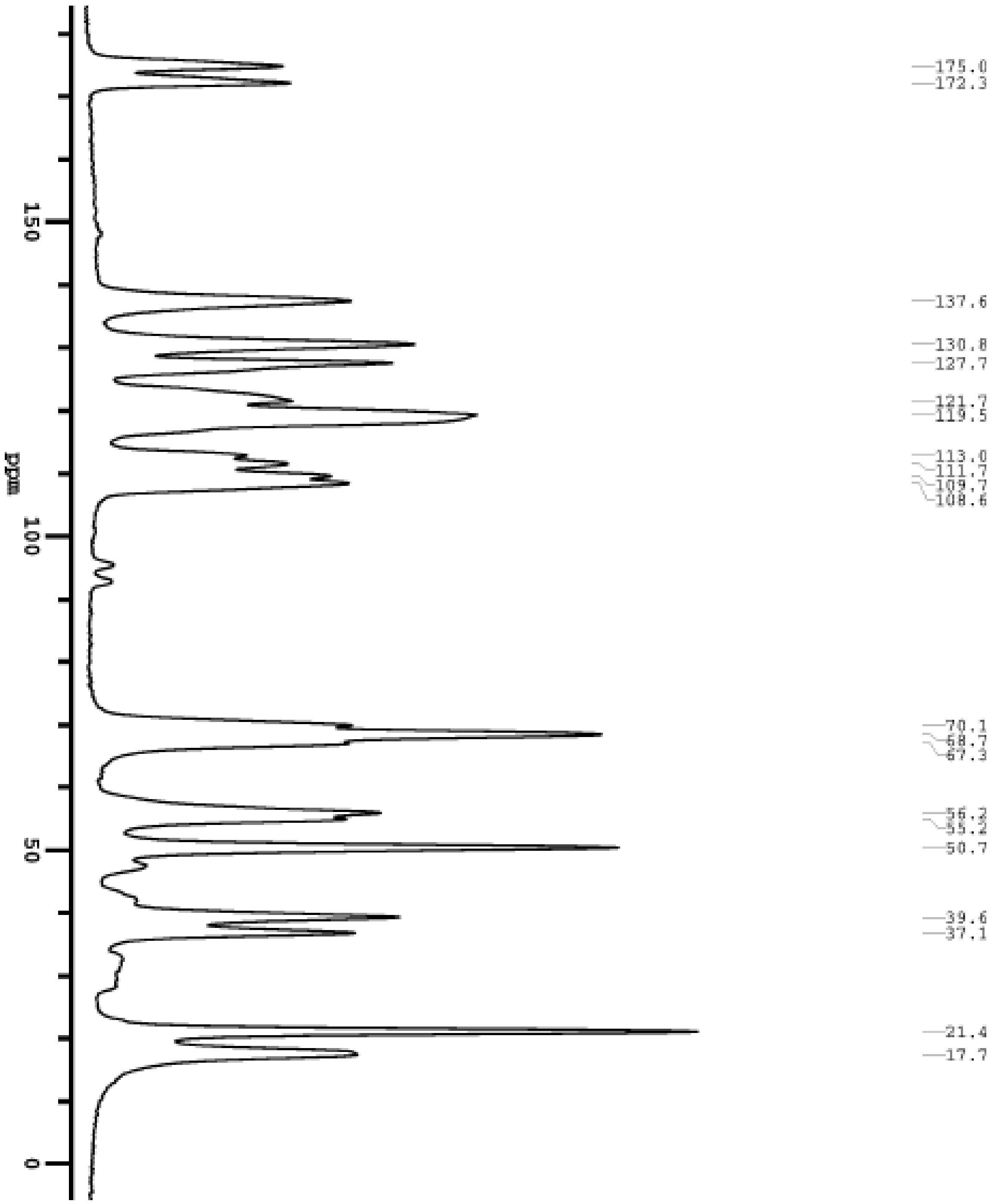

Figure S60. The ^{13}C CP-MAS NMR for compound **16** in solid state.

Compound **17**.

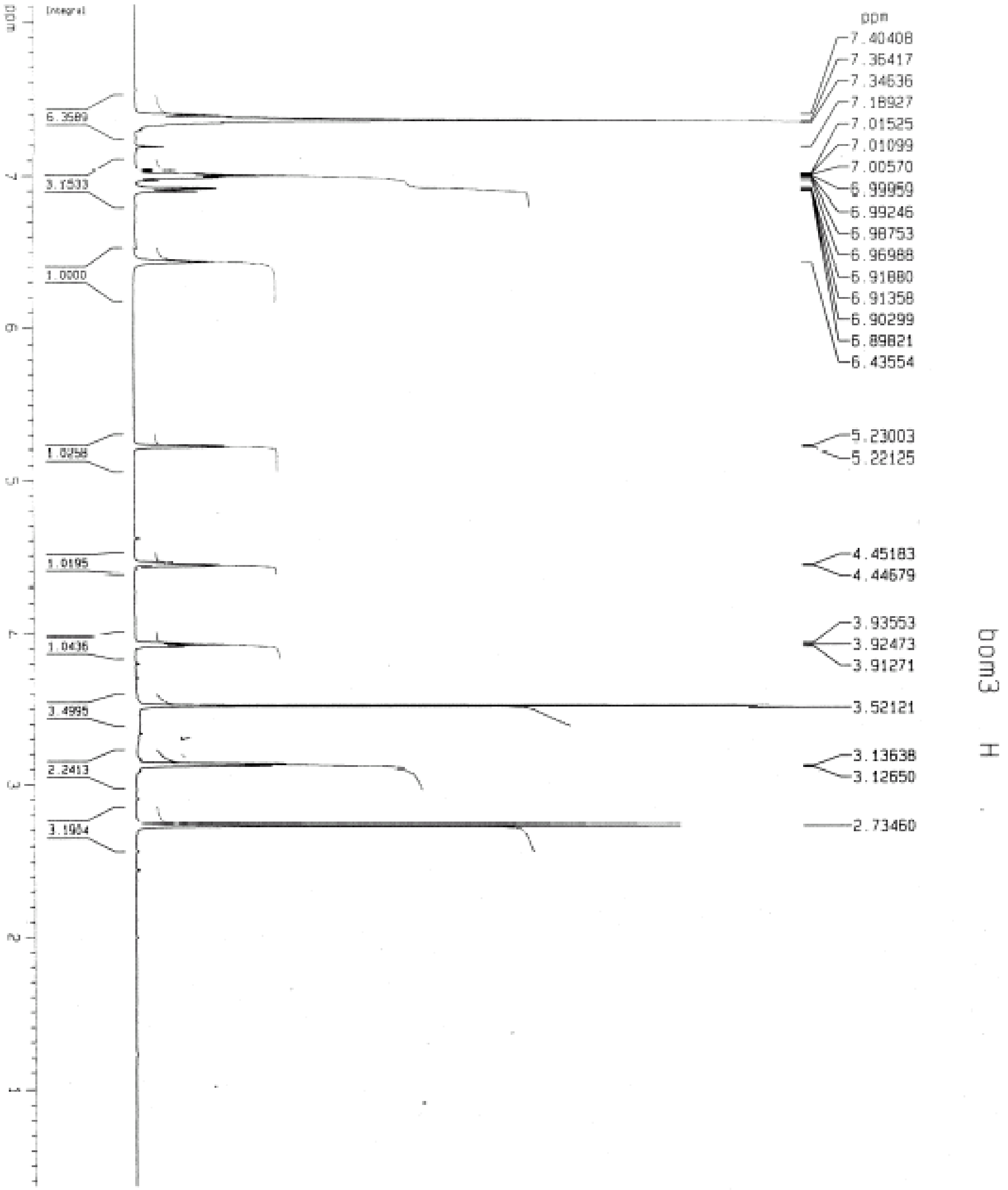

Figure S61. The ^{1}H NMR for **17** in $CDCl_3$.

Compound **17**

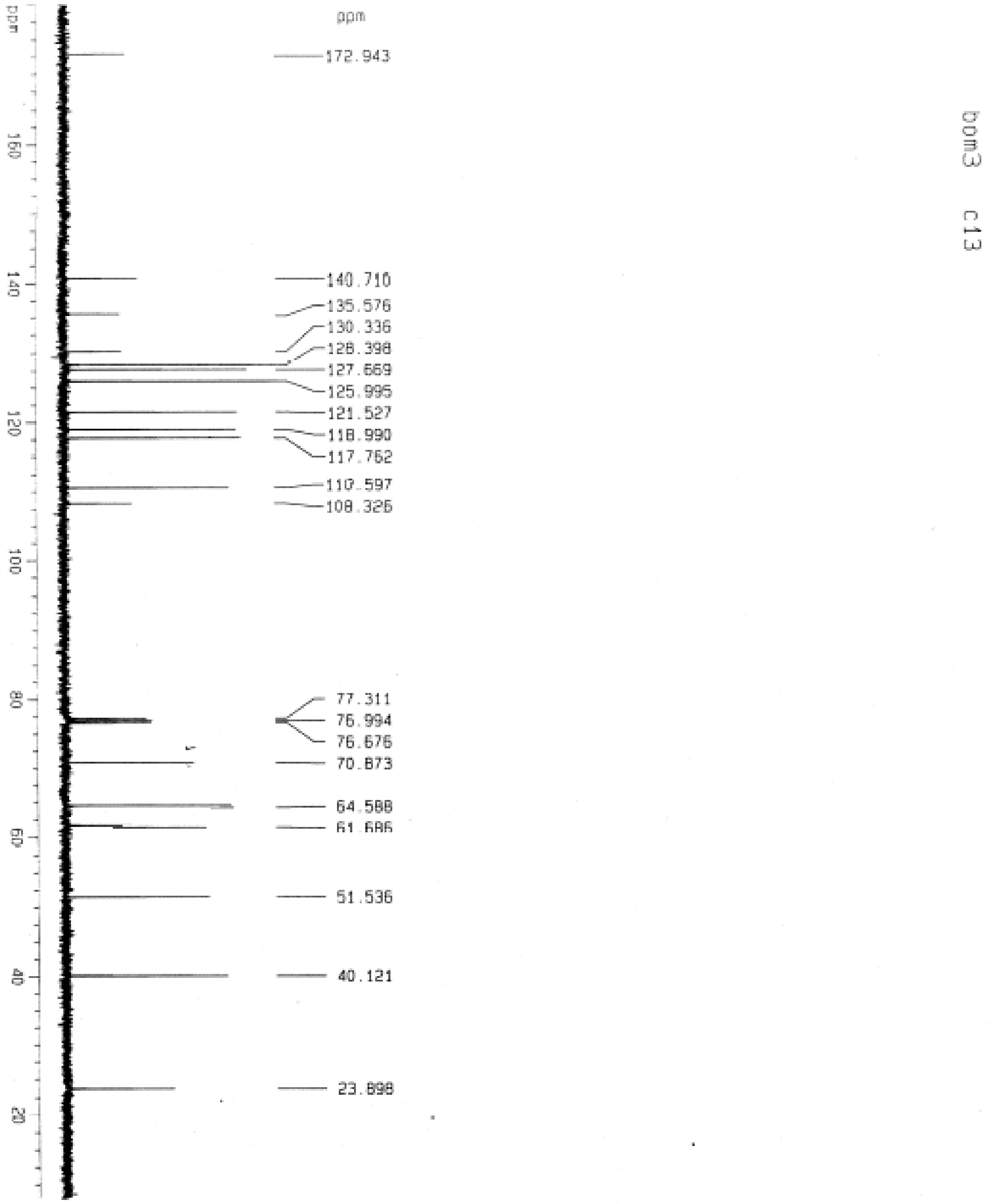

Figure S62**.** The ^{13}C NMR for **17** in $CDCl_3$.

Compound **17** .

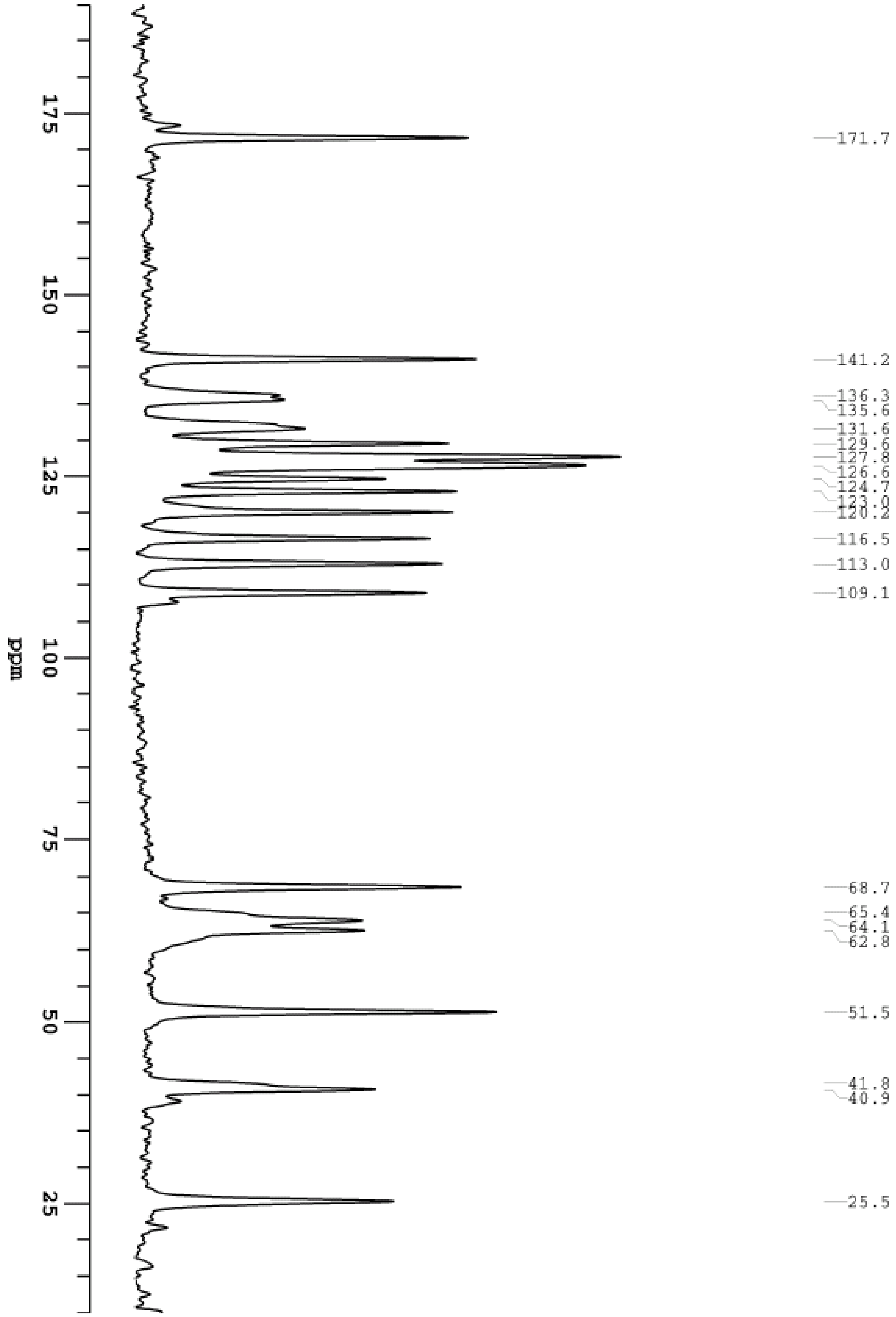

Figure S63. ^{13}C CP-MAS NMR for **17** in solid state.

IR spectra for 15 to 17

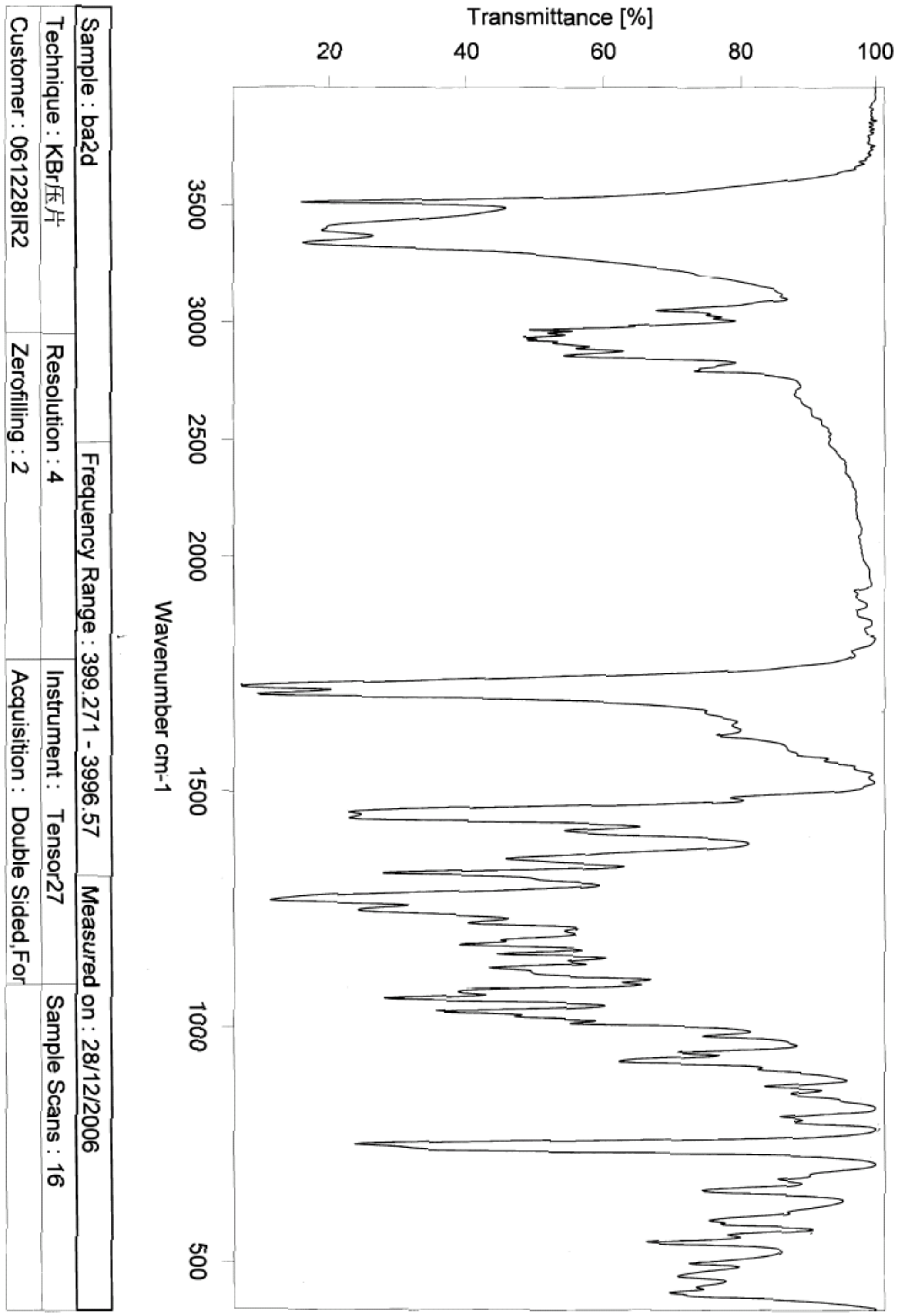

Figure S64**.** The IR for compound **15**

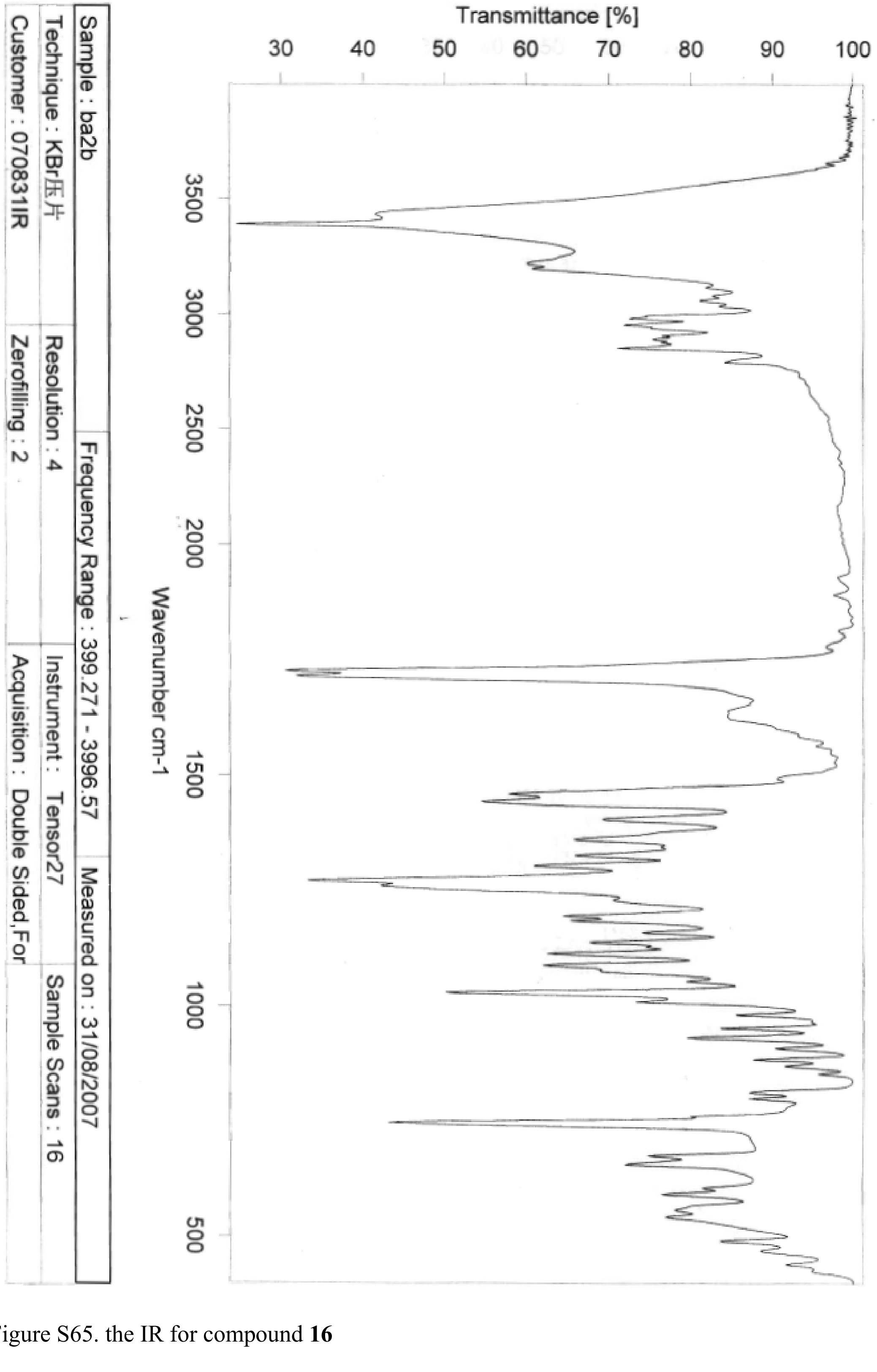

Figure S65. the IR for compound **16**

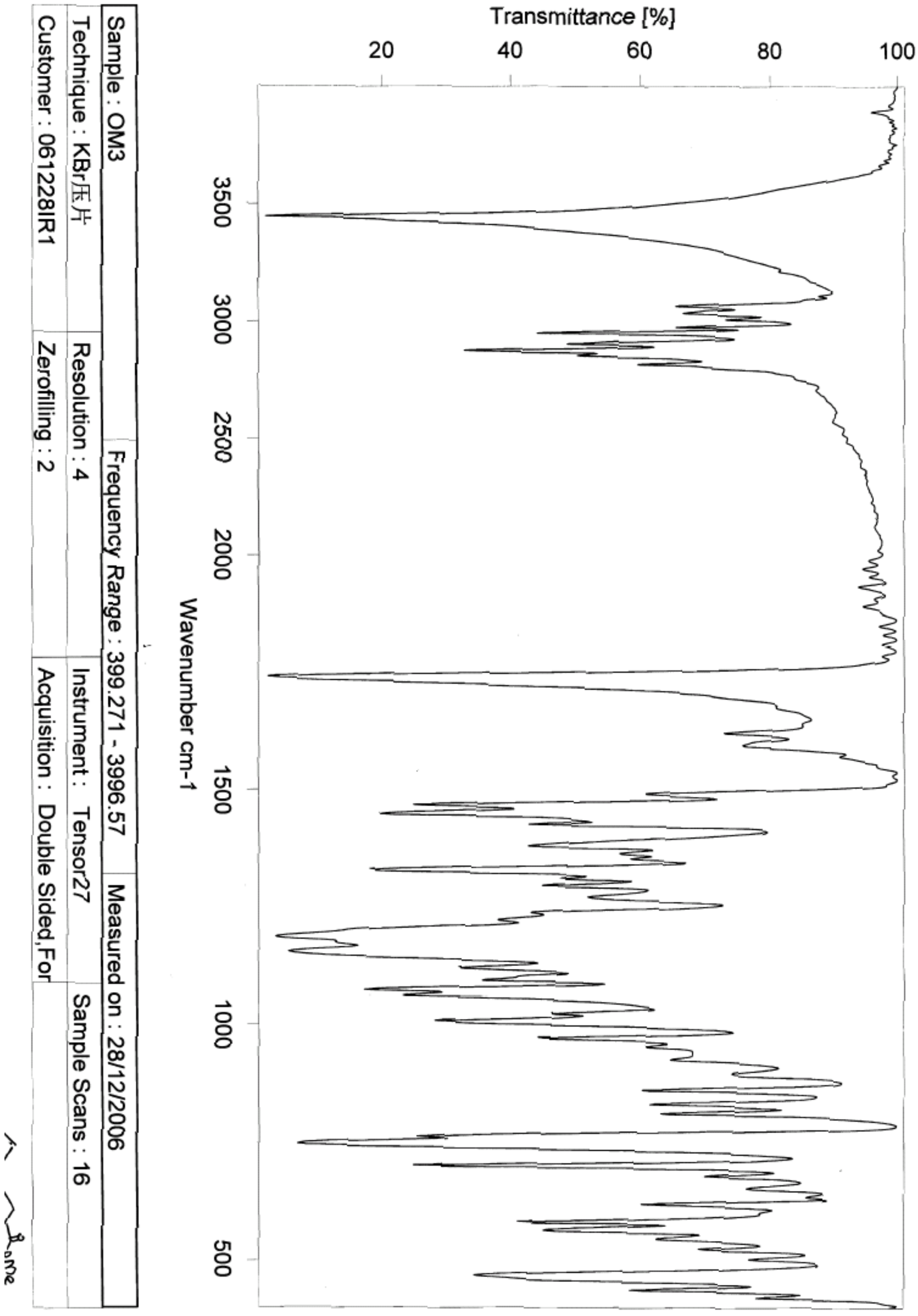

Figure S66**.** The IR for compound **17**.

The calculated ^{13}C NMR spectra (two conformations inside) for crystal structures 15 and 16 using their structural coordinates.

The differences in the chemical shifts of the same carbon atoms in the two geometries **15A** and **15B** or **16A** and **16B** were computed at the B3LYP/6-311+G(2d,p) level in the gas phase using the X-ray geometries (Tables S13 and S14). The large difference predicted in the carbonyl ^{13}C shift between **16A** and **16B** matched the experimental results (calcd. 3.6 ppm vs exp. 2.7 ppm). Similarly, a very small difference (< 0.5 ppm) was predicted between **15A** and **15B**. This result clearly exhibited that the observed ^{13}C CP-MAS NMR spectra for **15** and **16** arose from differences between the two configurations.

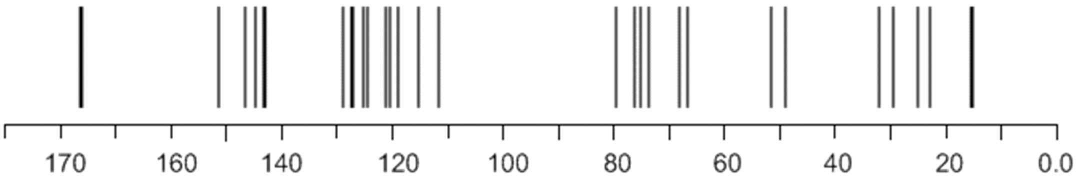

The calculated ^{13}C NMR for crystal **15**

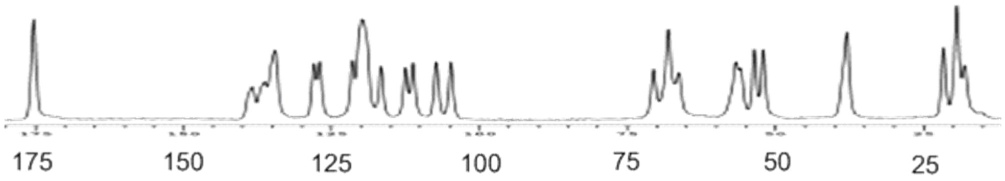

The experiemental ^{13}C NMR for crystal **15**

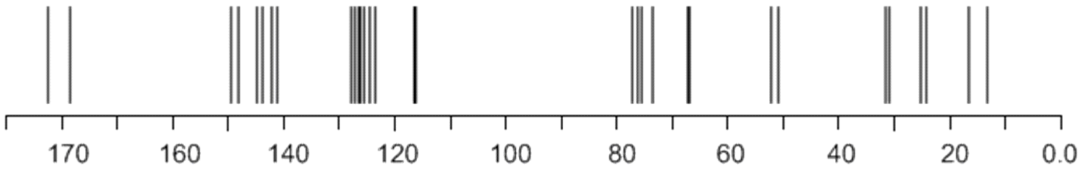

The calculated ^{13}C NMR for crystal **16**

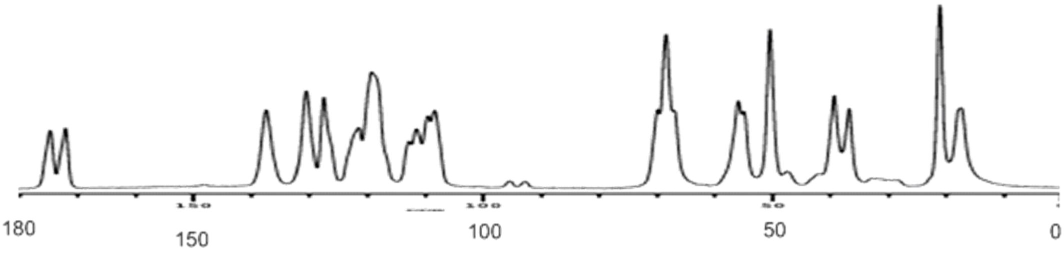

The experiemental ^{13}C NMR for crystal **16**

Figure S67. Comparison of the predicted ^{13}C NMR with the experimental data for **15** and **16** containing two geometries copied from the X-ray structures.

Analysis of crystal structures of 15 and 16.

The X-ray structure of **15** (Table 7) might provide an additional evidence to understand this "collision". The distance of two O atoms between two –OH groups is 2.811 Å. The bond O-H may be condensed first. This change quickly delivered to C1'-O10. Both of the C1'-O10 bond lengths in **15A** and **15B** were condensed to 1.416 Å and 1.413 Å, respectively, while that it was 1.423 Å in **17** (Table10). Thus, then the energy from the condensed bond delivered to the other neighboring bonds, leading to entire molecule to form the pseudo-resonance state. In the structures of **16A** and **16B**, one mole of water was involved in crystal. The O of water was 2.817 Å and 2.897 Å to the O on C-1' in **16A** and **16B** (Table8). The 2.897 Å in **16** is longer than 2.817 Å in **15**. Thus, the elastic force should be small and that may be the reason the differences of the bond lengths between **16A** and **16B** were smaller than those between **15A** and **15B** (Figure ure 4 in text).

The shorter the distance of the O atoms, the bigger the relative length differences will be in **15A** and **15B** via **16A** and **16B**.

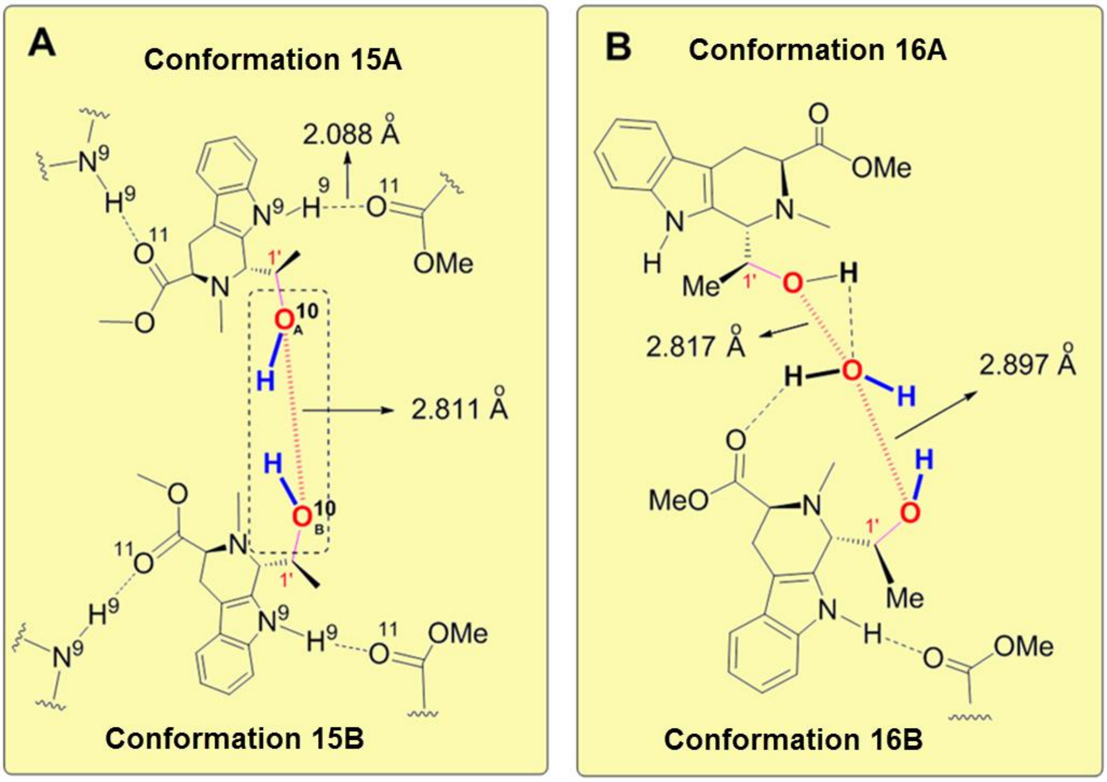

Figure S68. (A) The H-bond patterns among the geometries of **15** in solid state. (B) The interaction of one water molecule with two conformations **16A** and **16B**.

Physical data for compounds 18 to 20.

Compound **18**

$[\alpha]_D$ +6.7 ° ($CHCl_3$, 0.0134 g/ml), IR (KBr): 3523, 3398, 2949, 2892, 2860, 1709, 1464, 1435, 1391, 1366, 1329, 1264, 1240, 1221, 1175, 1125, 1111, 1066, 1038, 747. HRMS calcd for $C_{17}H_{23}N_2O_3$ [M+H$^+$]: 303.1709. found: 303.1715. ^{1}H NMR ($CDCl_3$, 500 MHz) δ: 1.34 (s, 3 H), 1.39 (s, 3 H), 2.40 (s, 3 H), 2.91 (dd, J = 16.0 Hz, 4.2 Hz, 1 H), 3.10 (dd, J = 14.8 Hz, 4.5 Hz, 1 H), 3.56 (s, 1 H), 3.82 (s, 3 H), 4.03-4.06 (m, 1 H), 7.12-7.57 (m, 4 H), 8.33 (s, 1 H). ^{13}C NMR ($CDCl_3$, 125 MHz) δ: 172.8, 136.3, 131.1, 126.6, 121.9, 119.4, 118.1, 110.9, 109.2, 73.9, 69.6, 58.9, 52.1, 39.5, 29.6, 28.3, 27.3, 18.2.

^{13}C NMR in the solid state: 176.2, 137.1 133.7, 128.4, 122.3, 119.5, 111.4, 110.0, 75.5, 68.2, 61.1, 53.6, 36.6, 29.8, 22.9, 18.1.

Compound **19**

$[\alpha]_D$ +239.6 ° (0.0154, $CHCl_3$), HRMS calcd for $C_{17}H_{25}N_2O_2$ [M+H$^+$]: 289.1916. found 289.1910. ^{1}H NMR ($CDCl_3$, 500 MHz) δ: 1.26 (s, 3 H), 1.40 (s, 3 H), 1.45 (d, J = 5.7 Hz, 3 H), 2.57 (s, 3 H), 2.79 (d, J = 15.5 Hz, 1 H), 2.97-3.03 (m, 1 H), 3.01 (d, J = 15.2 Hz, 1 H), 3.35-3.39 (m, 1 H), 3.83-3.86 (m, 1 H), 7.12-7.54 (m, 4 H), 8.09 (s, 1 H). ^{13}C NMR ($CDCl_3$, 125 MHz) δ: 136.4, 129.7, 126.7, 122.1, 119.6, 118.2, 111.0, 109.5, 72.5, 70.4, 67.0, 60.5, 37.3, 29.7, 28.5, 19.9, 15.8. IR (KBr): 3527, 3404, 2966, 2919. 2890, 1455, 1369, 1306, 1270, 1151, 1104, 1075, 1022, 751, 744.

^{13}C NMR in the solid state: 137.5, 130.3, 127.7, 119.7, 110.8, 71.8, 68.2, 60.3, 37.5, 29.9, 20.4, 16.3.

Compound **20**:

$[\alpha]_D$ -31.7 °($CHCl_3$), HRMS calcd for $C_{18}H_{27}N_2O_2$ [M+H$^+$]: 303.2073. found 303.2066. ^{1}H NMR ($CDCl_3$, 500 MHz) δ: 1.27 (s, 3 H), 1.38 (s, 3 H), 1.40 (s, 3 H), 1.41 (s, 3 H), 2.52 (s, 3 H), 2.69 (dd, J = 15.3 Hz, 2.0 Hz, 1 H), 2.86-2.91 (m, 1 H), 3.08 (dd, J = 11.4 Hz, 2.0 Hz, 1 H), 3.50 (s, 1 H), 3.82 (s, 3 H), 4.03-4.06 (m, 1 H), 7.11-7.56 (m, 4 H), 8.41 (s, 1 H). ^{13}C NMR ($CDCl_3$, 125 MHz) δ: 136.2, 131.7, 126.7, 121.5, 119.1, 117.8, 110.8, 73.9, 72.0, 71.4, 63.4, 38.9, 29.7, 28.9, 28.8, 27.4, 15.6. IR (KBr): 3440, 2970, 2929, 1630, 1462, 1370, 1314, 1270, 1234, 1154, 1142, 1098, 1030, 926, 743.

^{13}C NMR in the solid state: 137.2, 131.9, 127.7, 119.7, 111.4, 72.3, 62.4, 38.6, 28.6, 15.7.

NMR plots for 18-20

For **18**

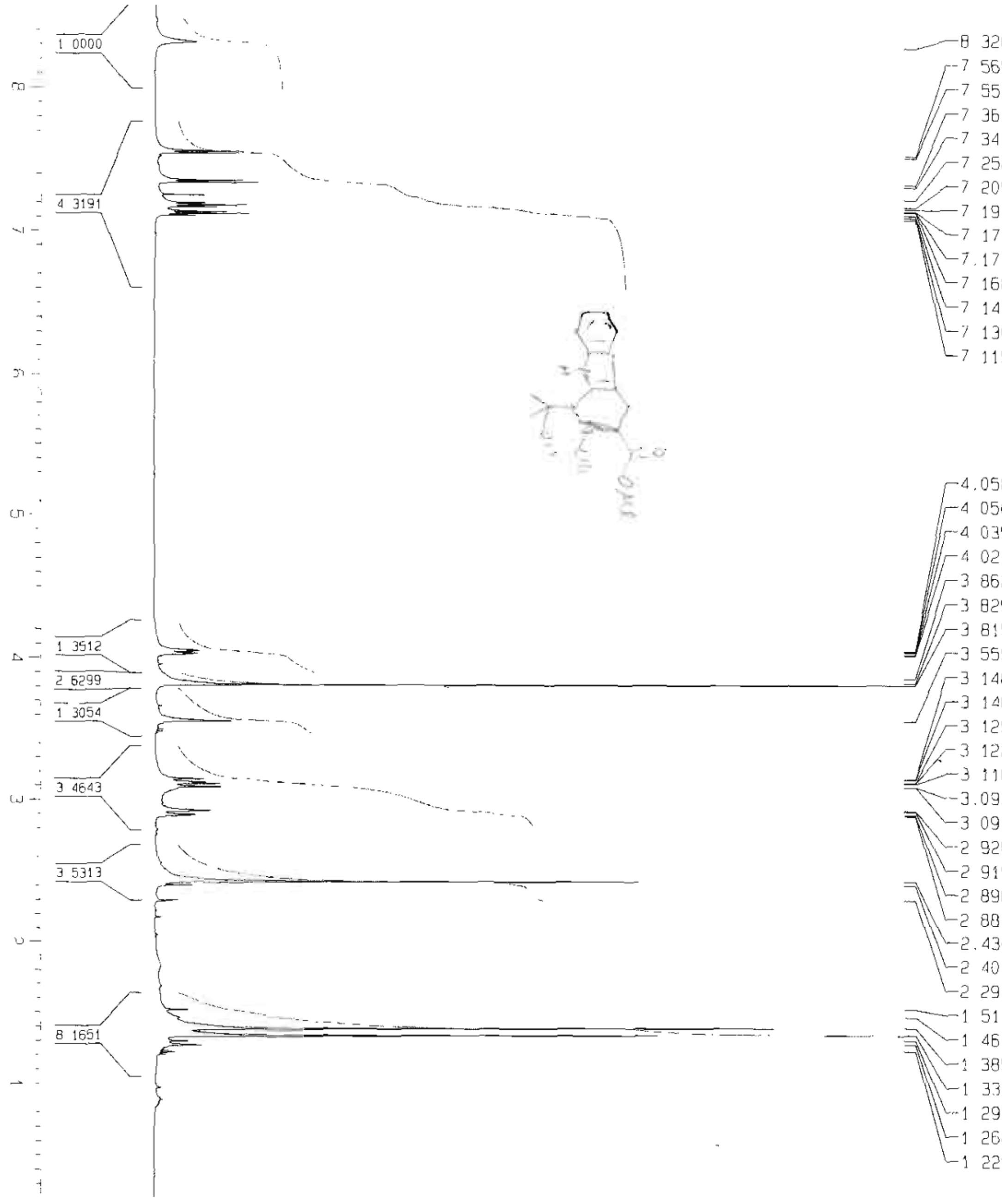

Figure S69. ^{1}H NMR spectrum for compound **18** in $CDCl_3$.

For **18**.

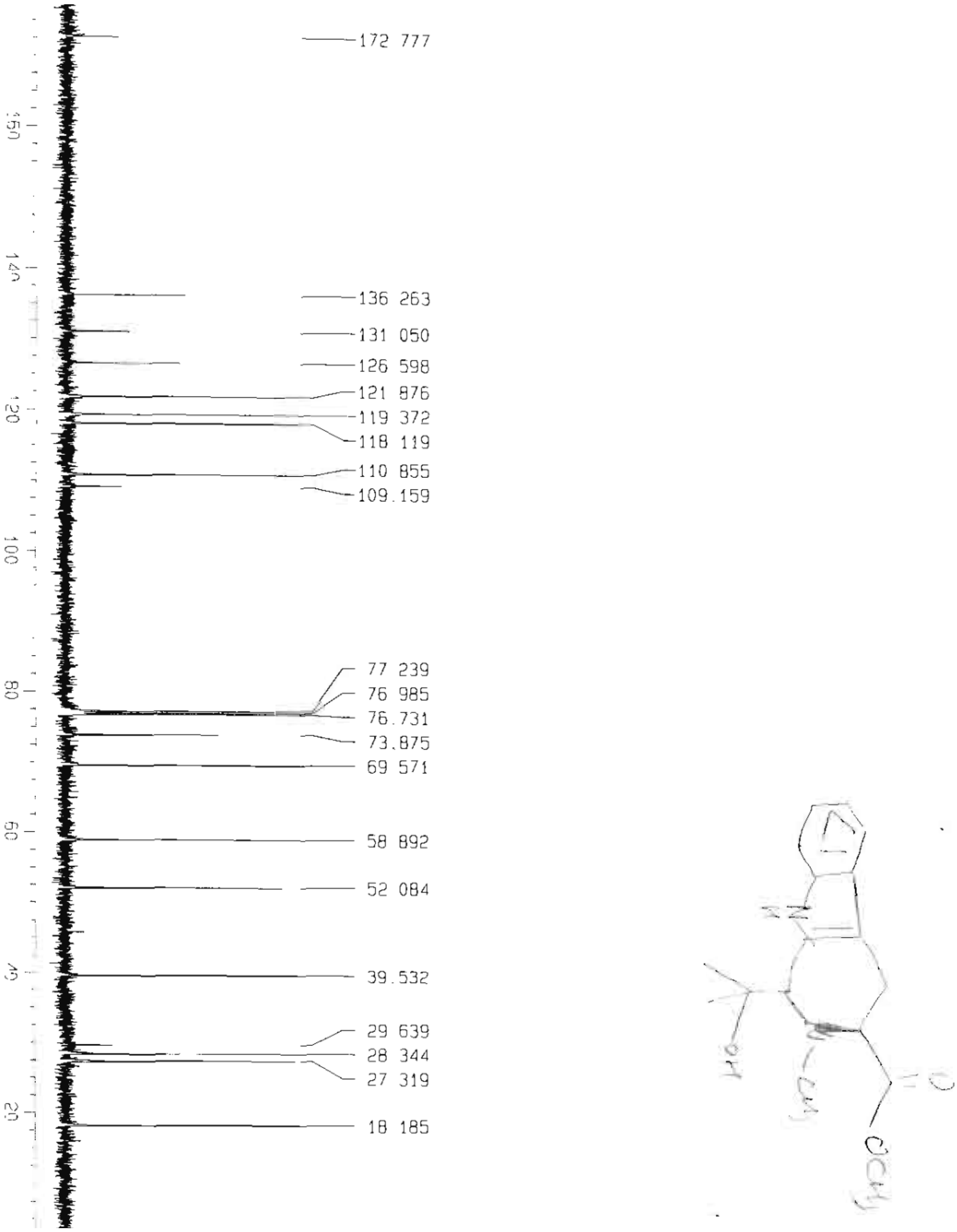

Figure S70. ^{13}C NMR spectrum for compound **18**.

For **18**

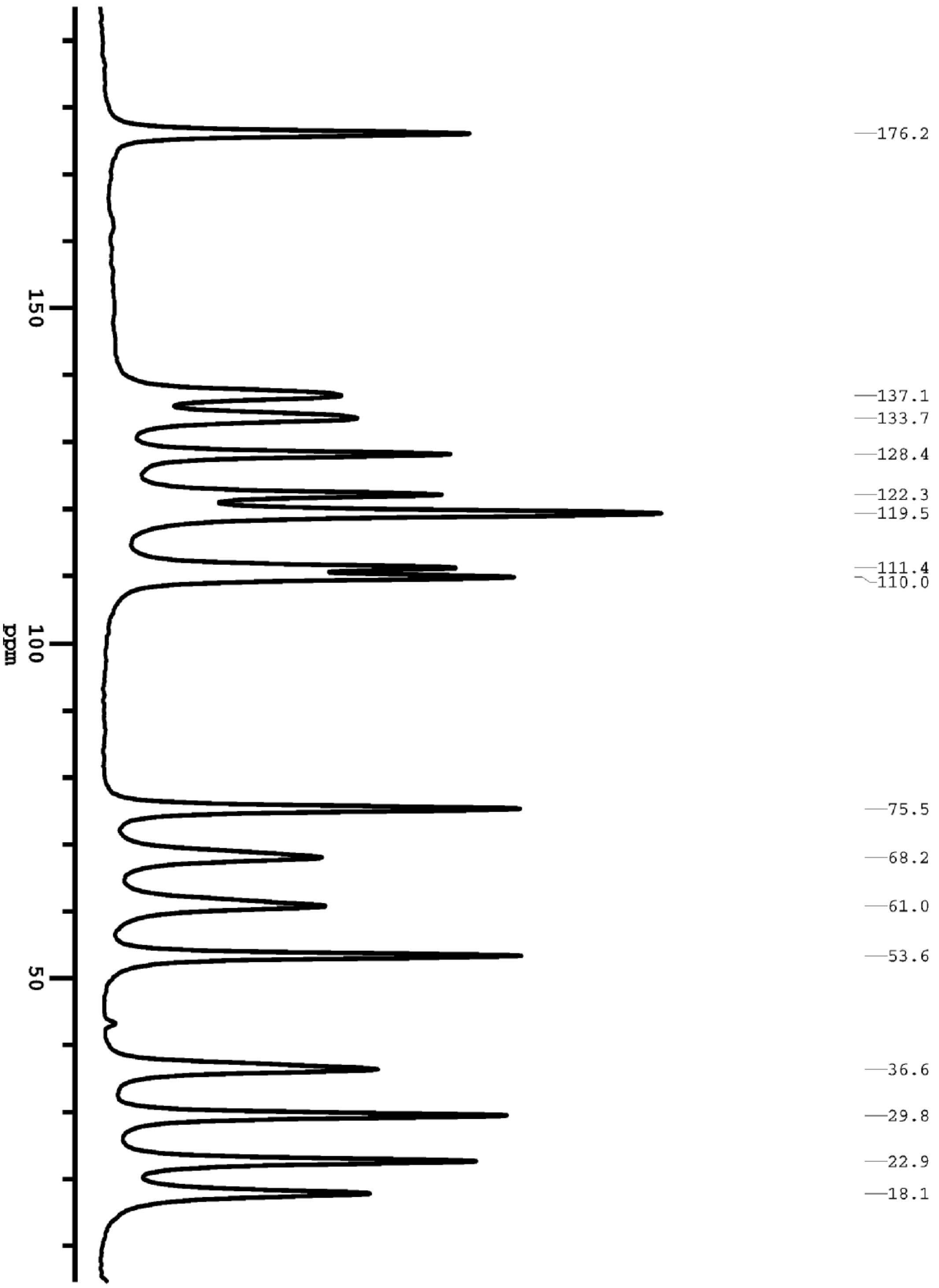

Figure S71. The ^{13}C CP-MAS NMR spectrum for compound **18** in solid state

For **19**

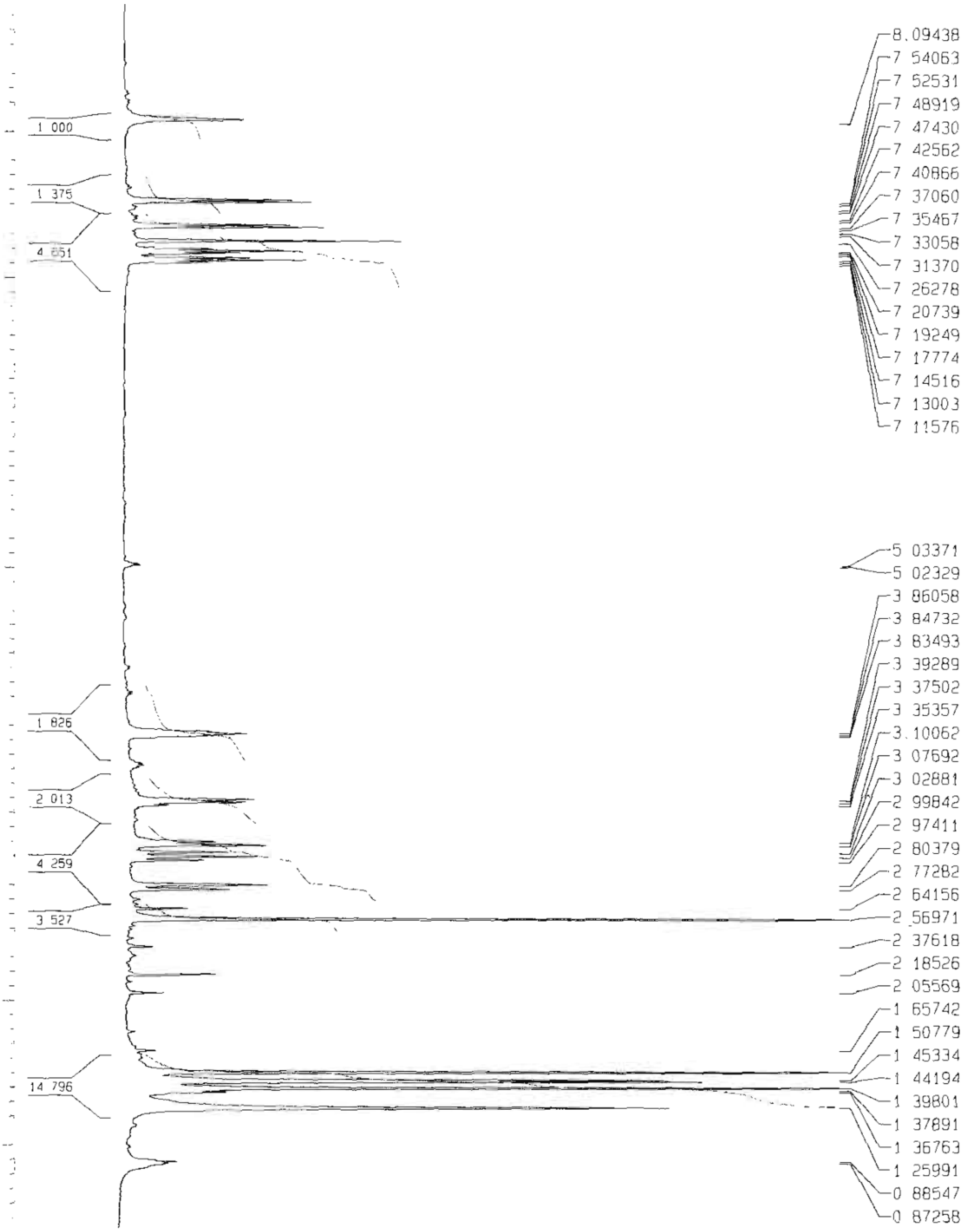

Figure S72. The ^{1}H NMR spectrum for **19** in $CDCl_3$.

For **19**

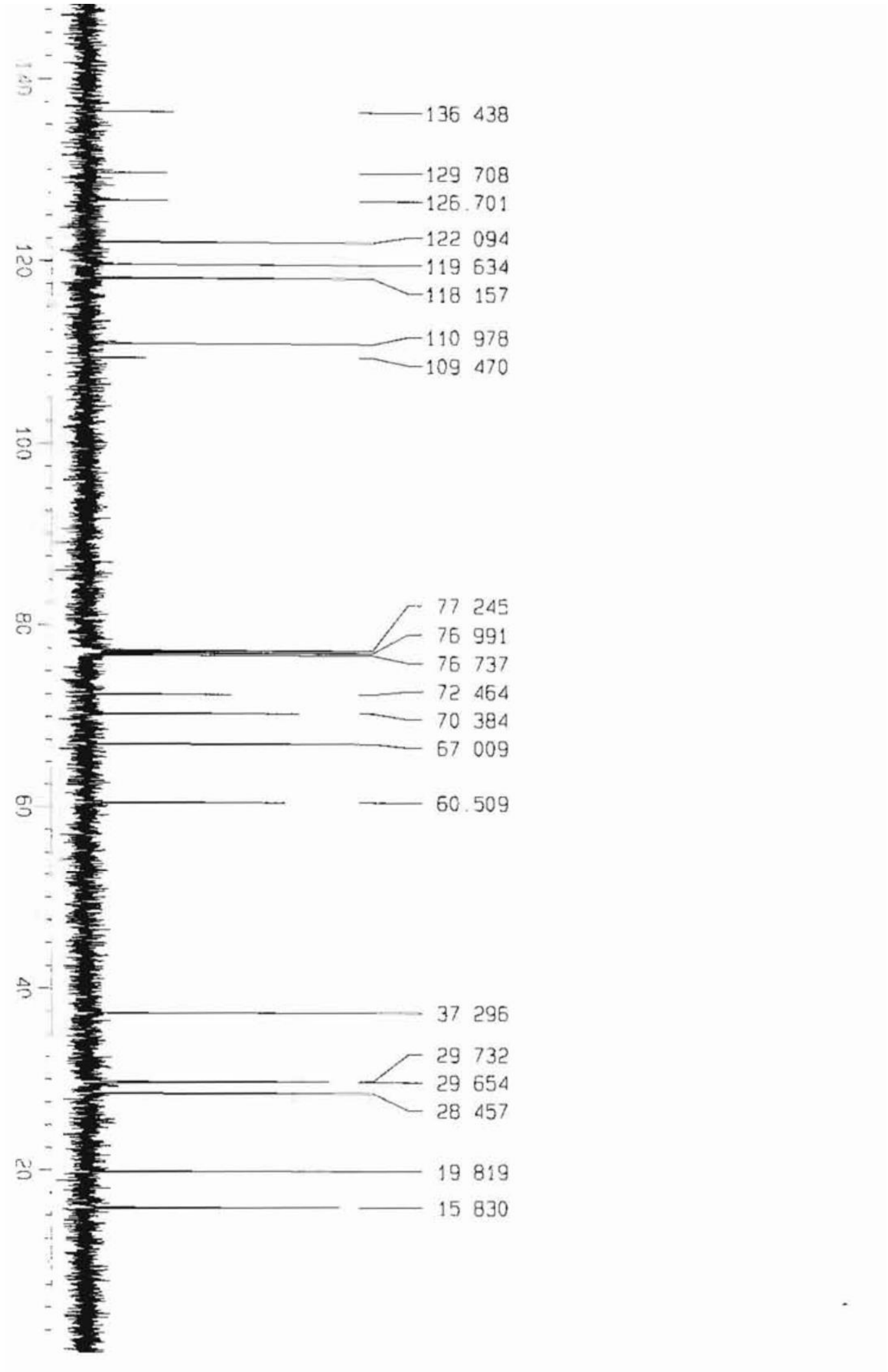

Figure S73. The ^{13}C NMR spectrum for **19** in $CDCl_3$.

For **19**

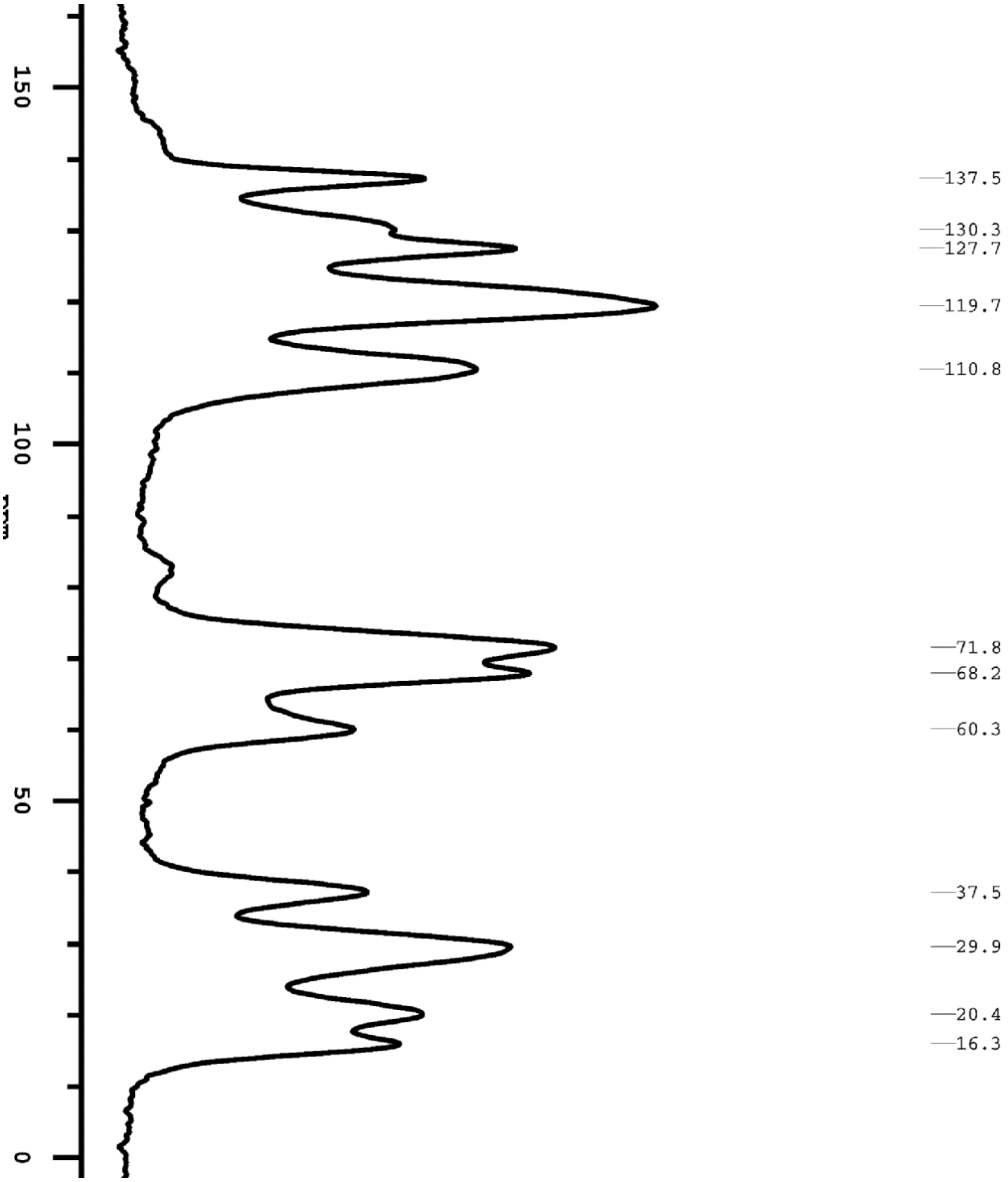

Figure S74. The ^{13}C CP-MAS NMR spectrum for **19** in solid state.

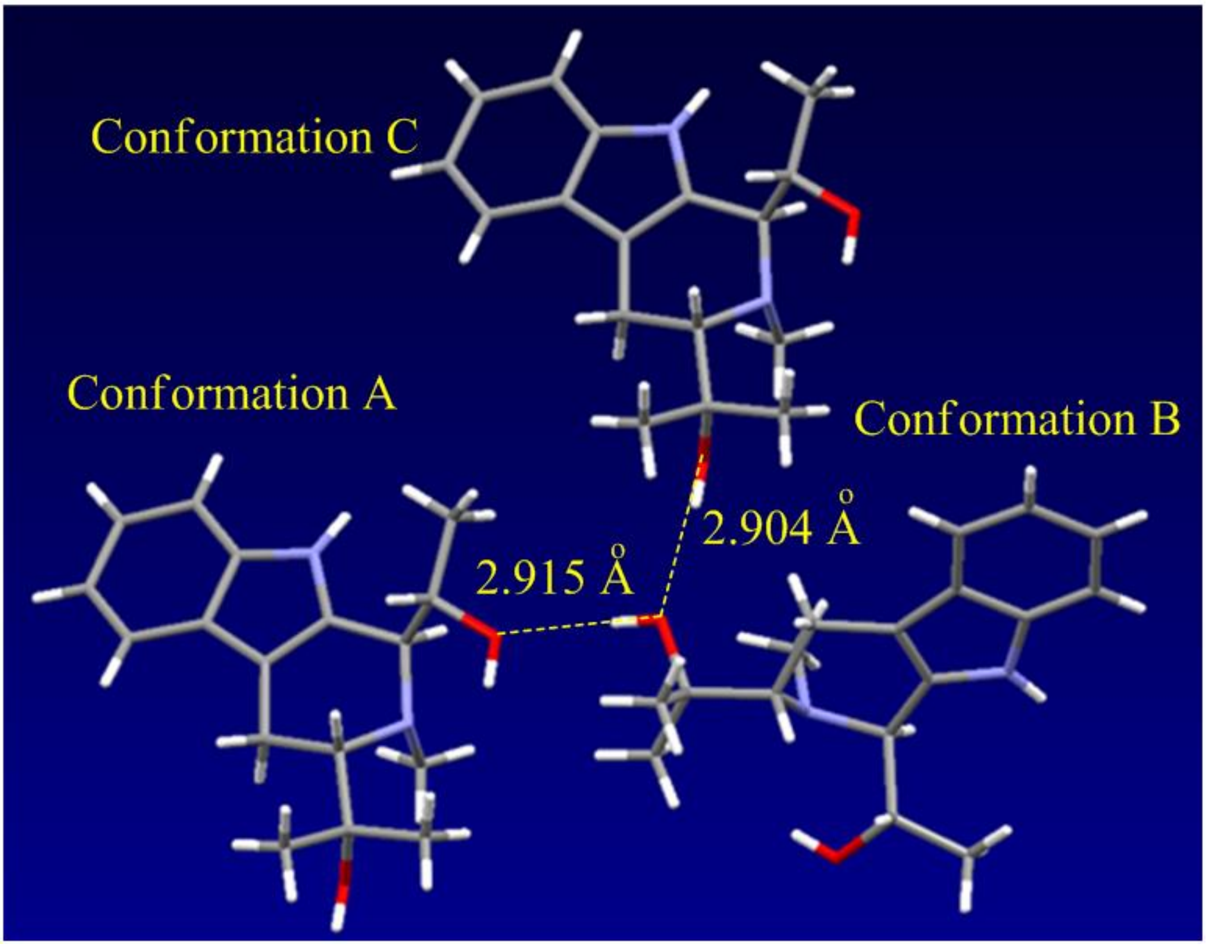

Figure S75. The X-ray structure of **19**. Three OH groups interact in space**.**

For **20**

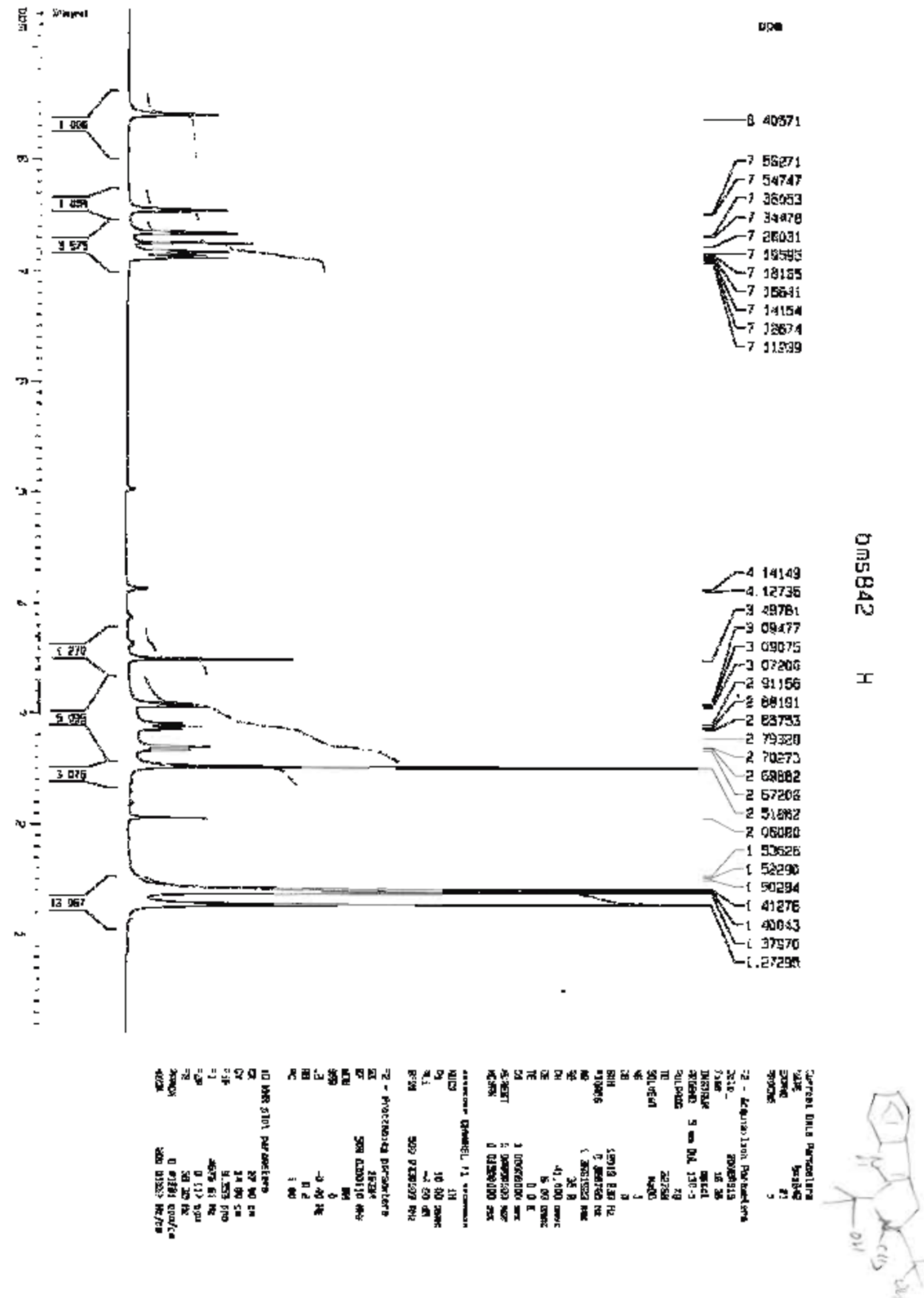

Figure S76. The ^{1}H NMR spectrum for **20** in $CDCl_3$.

For **20**

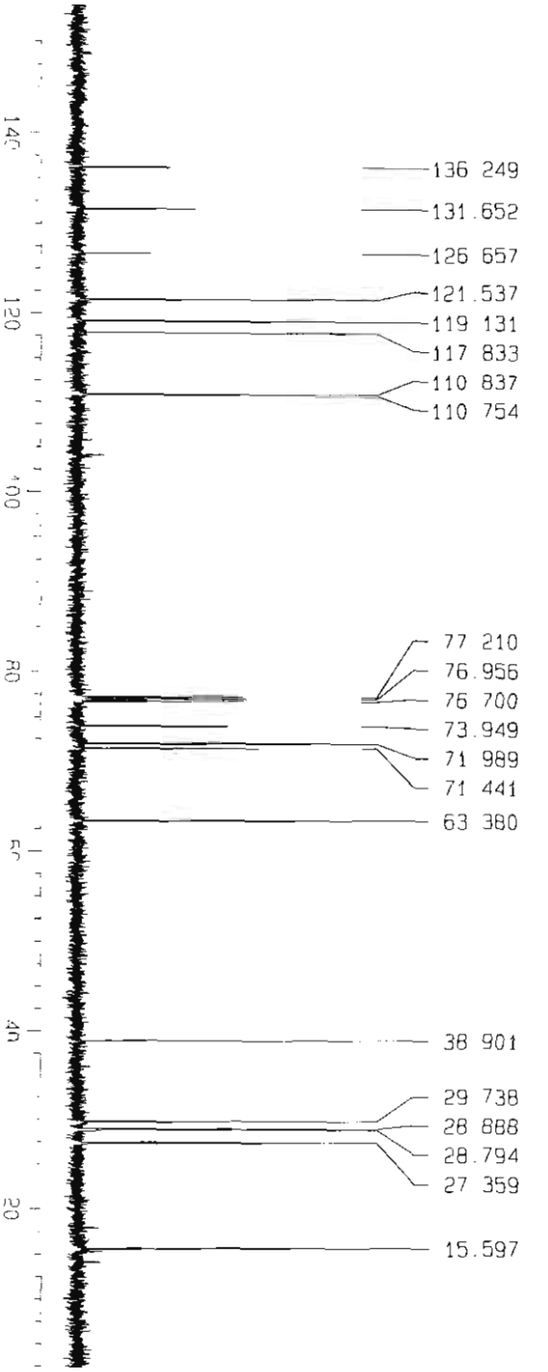

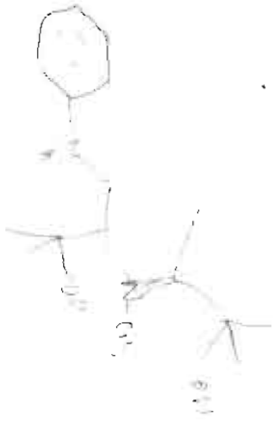

Figure S77. The ^{13}C NMR spectrum for **20** in $CDCl_3$.

For **20**

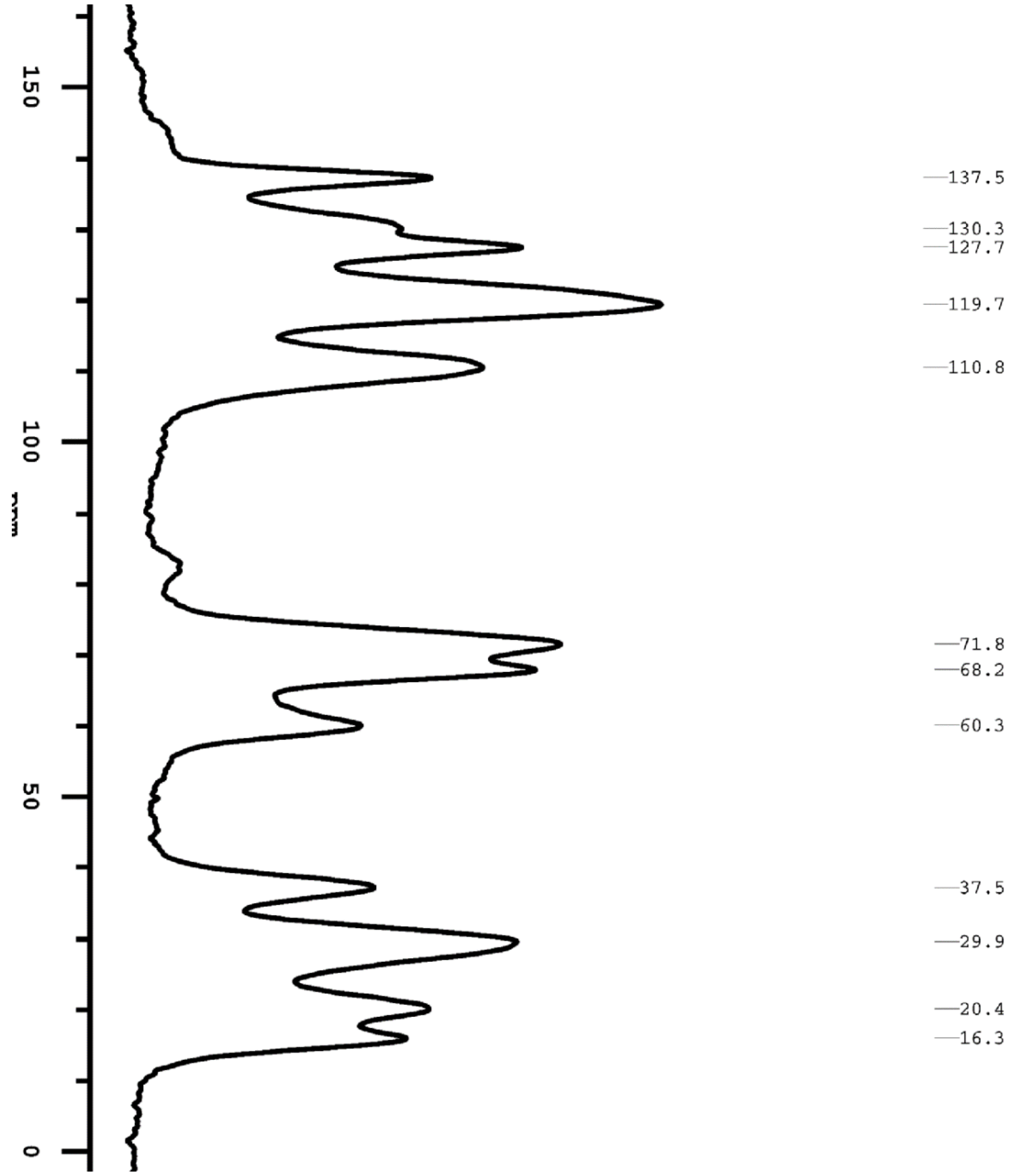

Figure S78. The ^{13}C CP-MAS NMR spectrum for **20** in solid state.

IR spectra for compounds 18 to 20.

For **18**

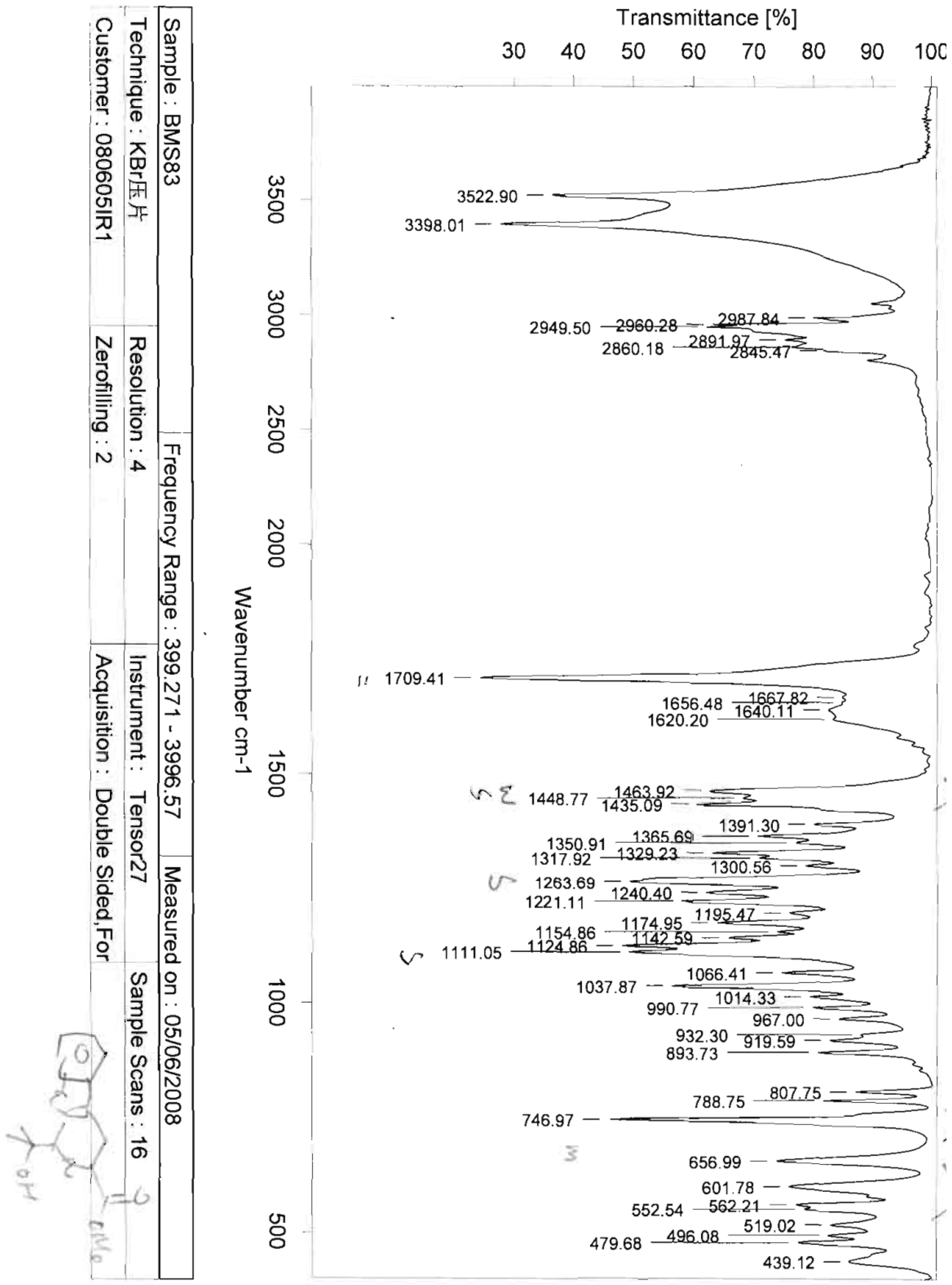

Figure S79. The IR spectrum for compound **18**.

For **19**

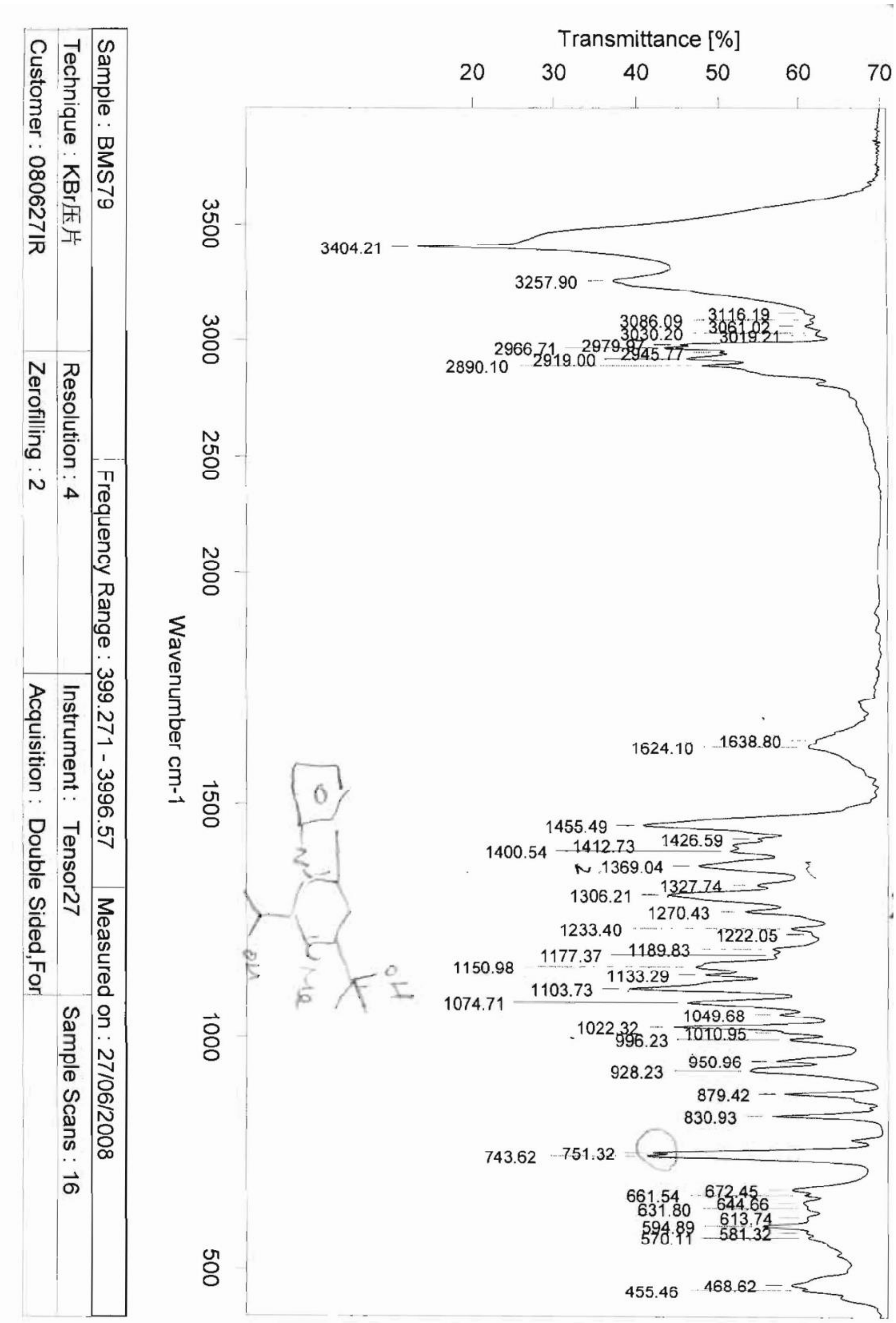

Figure S80**.** The IR spectrum for compound **19**

For **20**

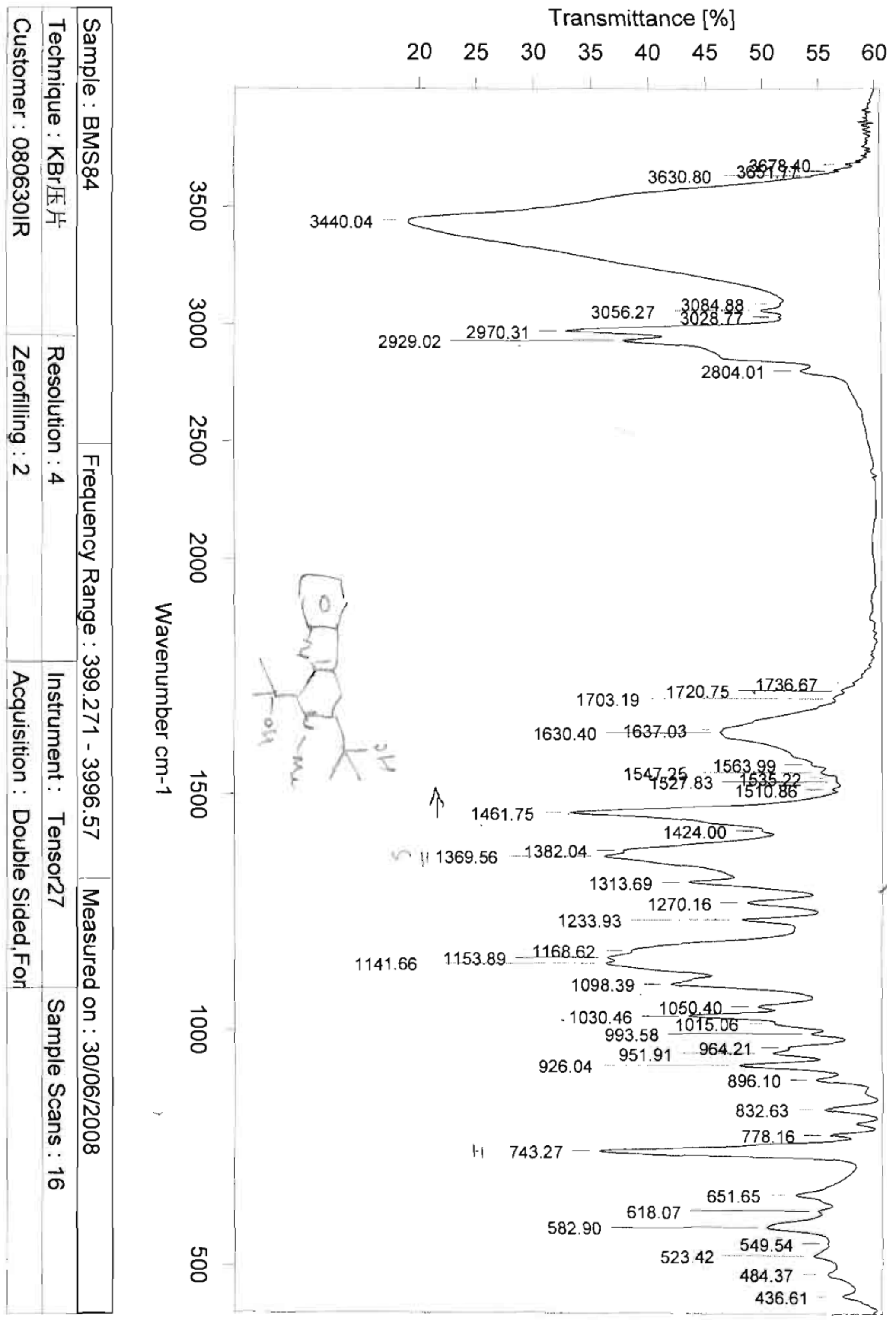

Figure S81. The IR spectrum for compound **20**

The physical data for compounds 21-26:

For compound **21**

White solid; yield 38%; mp 211.9-213.2 °C; $[\alpha]_D^{19}$= -44.44 (MeOH, *c*, 0.855). HRMS calcd for $C_{15}H_{20}N_3O_2$ [M+H+]: 274.1556. Found 274.1562. ^{1}H NMR (400 MHz, CD_3OD): δ 7.44 (d, *J*= 7.6 Hz, 1H), 7.30(d, *J*= 8.0 Hz, 1H), 7.05 (m, 1H), 6.96 (m, 1H), 3.89 (m, 1H), 3.75 (dd, *J*= 5.4, 10.4 Hz, 1H), 3.43 (d, *J*= 7.6 Hz, 1H), 2.87 (m, 2H), 2.31 (s, 3H), 1.36 (d, *J*= 6.2Hz, 3H); ^{13}C NMR (100 MHz, CD_3OD): δ 177.7, 138.1, 133.6, 128.0, 122.1,119.6, 118.6, 111.9, 108.1, 70.8, 68.1, 59.7, 38.0, 21.6, 17.9; IR (KBr) υ_{max} = 3439, 3297, 3239, 1675, 1574, 1459, 1318, 1292, 1254, 1112, 1069, 1025, 941, 745 cm^{-1}.

For compound **22**

White solid; yield 31%; mp 182.4-184.0 °C; $[\alpha]_D^{19}$= -11.56 (MeOH, *c*, 0.865); HRMS calcd for $C_{15}H_{20}N_3O_2$ [M+H+]: 274.1556. Found 274.1551. ^{1}H NMR (500 MHz, CD_3OD): δ 7.33 (d, *J*= 7.7 Hz, 1H), 7.19(d, *J*= 8.0 Hz, 1H), 6.95 (m, 1H), 6.86 (m, 1H), 3.82 (m, 2H), 3.30 (d, *J*= 8.1 Hz, 1H), 2.78 (m, 2H), 2.19 (s, 3H), 1.24 (d, *J*= 5.8 Hz, 3H); ^{13}C NMR (100 MHz, CD_3OD): δ 177.7, 138.3, 131.1, 127.9, 122.4, 119.7, 118.7, 111.9, 109.0, 69.5, 69.3, 57.9, 37.5, 19.9, 18.2.

For compound **23**

White solid; yield 26%; mp 145.9-147.1 °C; $[\alpha]_D^{19}$= +17.71 (EtOAc, *c* 0.960); HRMS calcd for $C_{17}H_{23}N_2O_3$ [M+H+]: 303.1709. Found 303.1702. ^{1}H NMR (400 MHz, $CDCl_3$): δ 8.19 (brs, 1H), 7.53 (d, *J*= 7.7 Hz, 1H), 7.34(d, *J*= 7.9 Hz, 1H), 7.18 (m, 1H), 7.13 (m, 1H), 4.23 (m, 2H), 3.96 (m, 2H), 3.62 (d, *J*= 7.8 Hz, 1H), 3.15 (dd, *J*= 9.4, 16.1 Hz, 1H), 2.99 (dd, *J*= 5.1, 16.0 Hz, 1H), 2.49 (s, 3H), 1.31 (m, 3H); ^{13}C NMR (100 MHz, $CDCl_3$): δ 172.0, 136.1, 130.4, 126.5, 121.9,119.4, 118.1, 110.8, 107.9, 67.3, 66.4, 57.6, 38.2, 19.8, 18.7, 14.1; IR (KBr) υ_{max} = 3371, 3265, 2971, 2885, 1730, 1455, 1371, 1303, 1269, 1177, 1103, 745 cm^{-1}.

For compound **24**:

White solid; yield 26%; mp 151.5-152.6 °C, $[\alpha]_D^{19}$= +36.02 (EtOAc, *c*, 0.805); HRMS calcd for $C_{18}H_{25}N_2O_3$ [M+H$^+$]: 317.1865. Found 317.1868. ^{1}H NMR (500 MHz, $CDCl_3$): δ 8.07 (brs, 1H), 7.52 (d, *J*= 7.7 Hz, 1H), 7.32 (d, *J*= 8.0 Hz, 1H), 7.17 (m, 1H), 7.12 (m, 1H), 5.08 (m, 1H), 4.04 (brs, 1H), 3.95 (m, 1H), 3.87 (dd, *J*= 4.4, 9.0 Hz, 1H), 3.56 (d, *J*= 7.9 Hz, 1H), 3.12 (dd, *J*= 9.6, 16.1 Hz, 1H), 2.94 (dd, *J*= 5.0, 16.0 Hz, 1H), 2.46 (s, 3H), 1.31 (d, *J*= 6.0 Hz, 3H), 1.26 (t, *J*= 6.5 Hz, 6H); ^{13}C NMR (125 MHz, $CDCl_3$): δ 171.6, 136.1, 130.6, 126.6, 121.9, 119.5, 118.1, 110.8, 108.1, 68.3, 67.3, 66.5, 57.6, 38.1, 21.8, 21.7, 19.8, 18.7; IR (KBr) υ_{max} = 3376, 3259, 2976, 1727, 1455, 1371, 1300, 1269, 1181, 1103, 1020, 744 cm^{-1}.

For compound **25**

White solid; yield 28%; mp 150.0-151.1 °C, $[\alpha]_D^{19}$= +23.60 (EtOAc, *c*, 0.805); HRMS calcd for $C_{18}H_{25}N_2O_3$ [M+H$^+$]: 317.1865. Found 317.1860. ^{1}H NMR (500 MHz, $CDCl_3$): δ 8.19 (brs, 1H),

7.53 (d, *J*= 7.7 Hz, 1H), 7.33 (d, *J*= 8.0 Hz, 1H), 7.18 (m, 1H), 7.12 (m, 1H), 5.09 (m, 1H), 4.13 (brs, 1H), 3.96 (m, 1H), 3.89 (dd, *J*= 5.1, 9.4 Hz, 1H), 3.59 (d, *J*= 7.8 Hz, 1H), 3.14 (dd, *J*= 9.4, 15.9 Hz, 1H), 2.96 (dd, *J*= 5.0, 16.0 Hz, 1H), 2.48 (s, 3H), 1.31 (d, *J*= 6.0 Hz, 3H), 1.27 (m, 6H); ^{13}C NMR (125 MHz, $CDCl_3$): δ 171.6, 136.1, 130.6, 126.5, 121.8, 119.4, 118.1, 110.8, 107.9, 68.3, 67.3, 66.4, 57.6, 38.1, 21.76, 21.72, 19.8, 18.7.

For compound **26**

White solid; yield 26%; mp 191.4-192.7 °C; $[\alpha]_D^{19}$= -34.44 (EtOAc, *c*, 0.900); $C_{22}H_{24}N_2O_3$; ^{1}H NMR (500 MHz, $CDCl_3$): δ 8.17 (brs, 1H), 7.60 (d, *J*= 7.7 Hz, 1H), 7.33 (m, 5H), 7.29 (m, 1H), 7.23 (m, 1H), 7.23 (m, 1H), 4.16 (m, 1H), 3.89 (m, 3H), 3.83 (s, 3H), 3.61 (m, 2H), 3.23 (dd, *J*= 10.2, 11.1 Hz, 1H), 3.12 (dd, *J*= 4.7, 11.3 Hz, 1H), 1.23 (d, *J*= 5.9 Hz, 3H); ^{13}C NMR (100 MHz, $CDCl_3$): δ 172.8, 138.3, 136.2, 130.5, 128.9, 128.6, 127.6, 126.6, 122.1, 119.6, 118.3, 110.0, 108.4, 67.3, 62.3, 56.9, 53.2, 52.2, 20.6, 18.9； IR (KBr) υ_{max} = 3315, 2942, 2852, 1737, 1457, 1329, 1302, 1266, 1219, 1141, 1048, 941, 746, 700 cm^{-1}.

NMR spectra form compounds 21 to 26

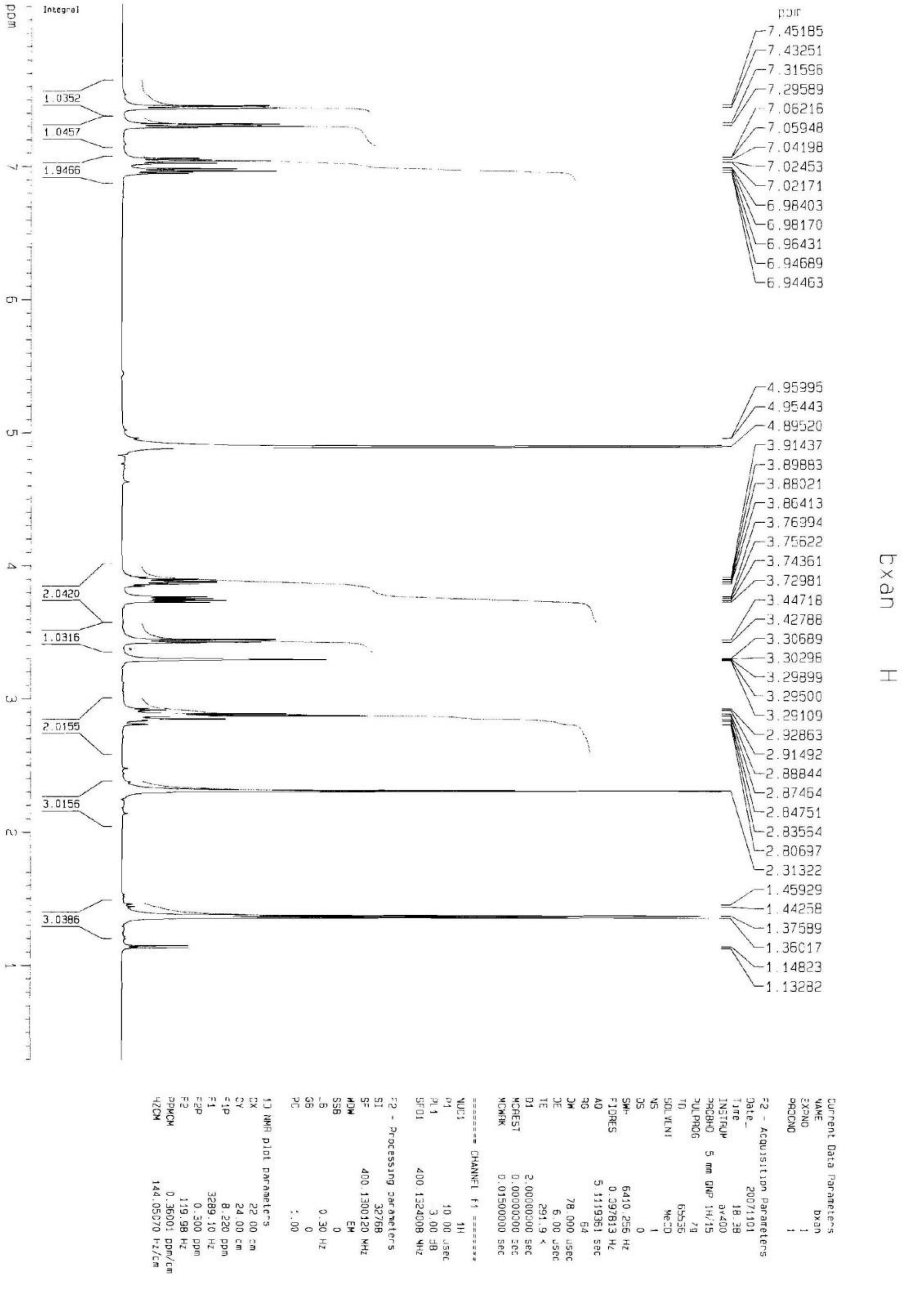

Figure S82. ^{1}H NMR for compound **21**

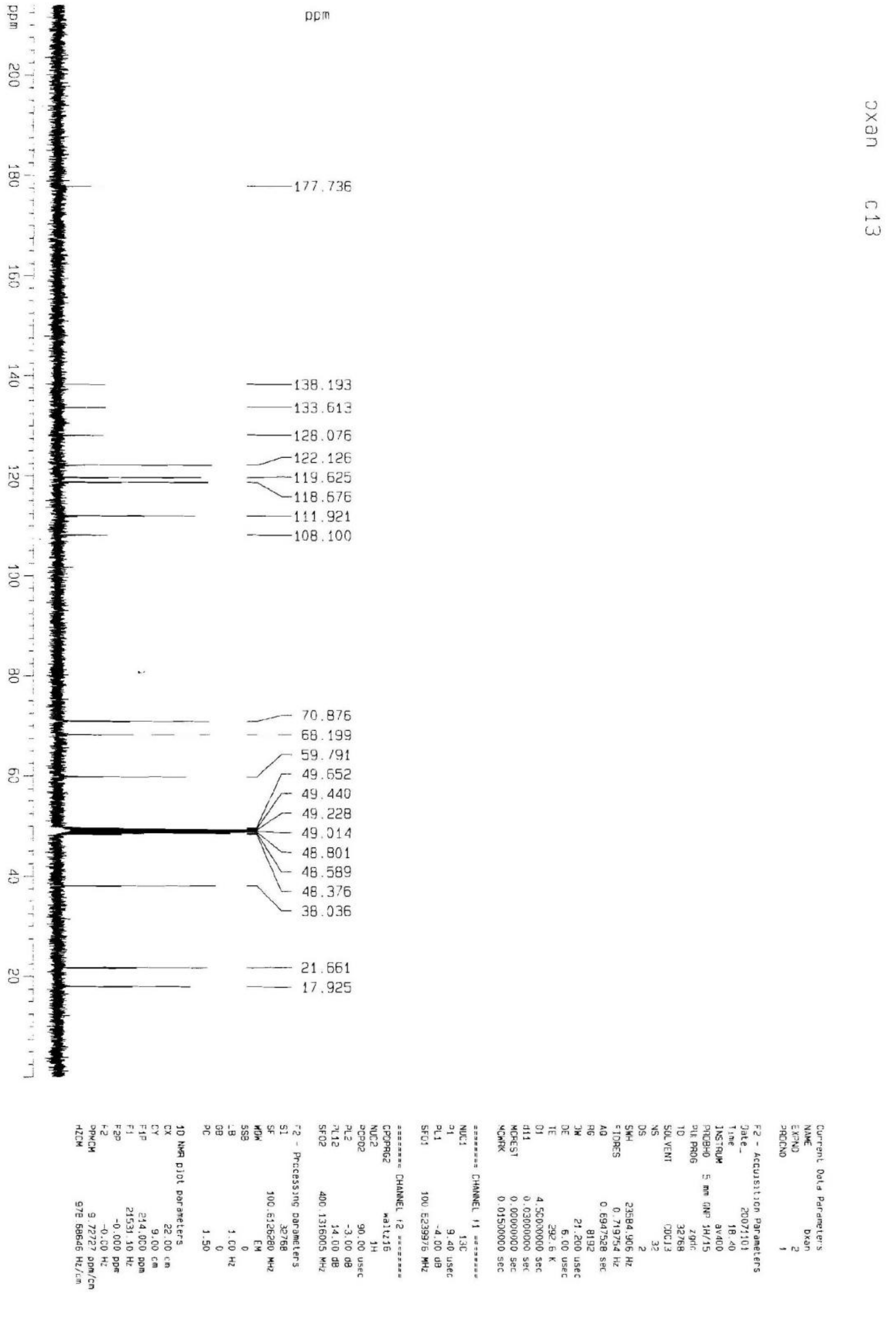

Figure S83. ^{13}C NMR for compound **21.**

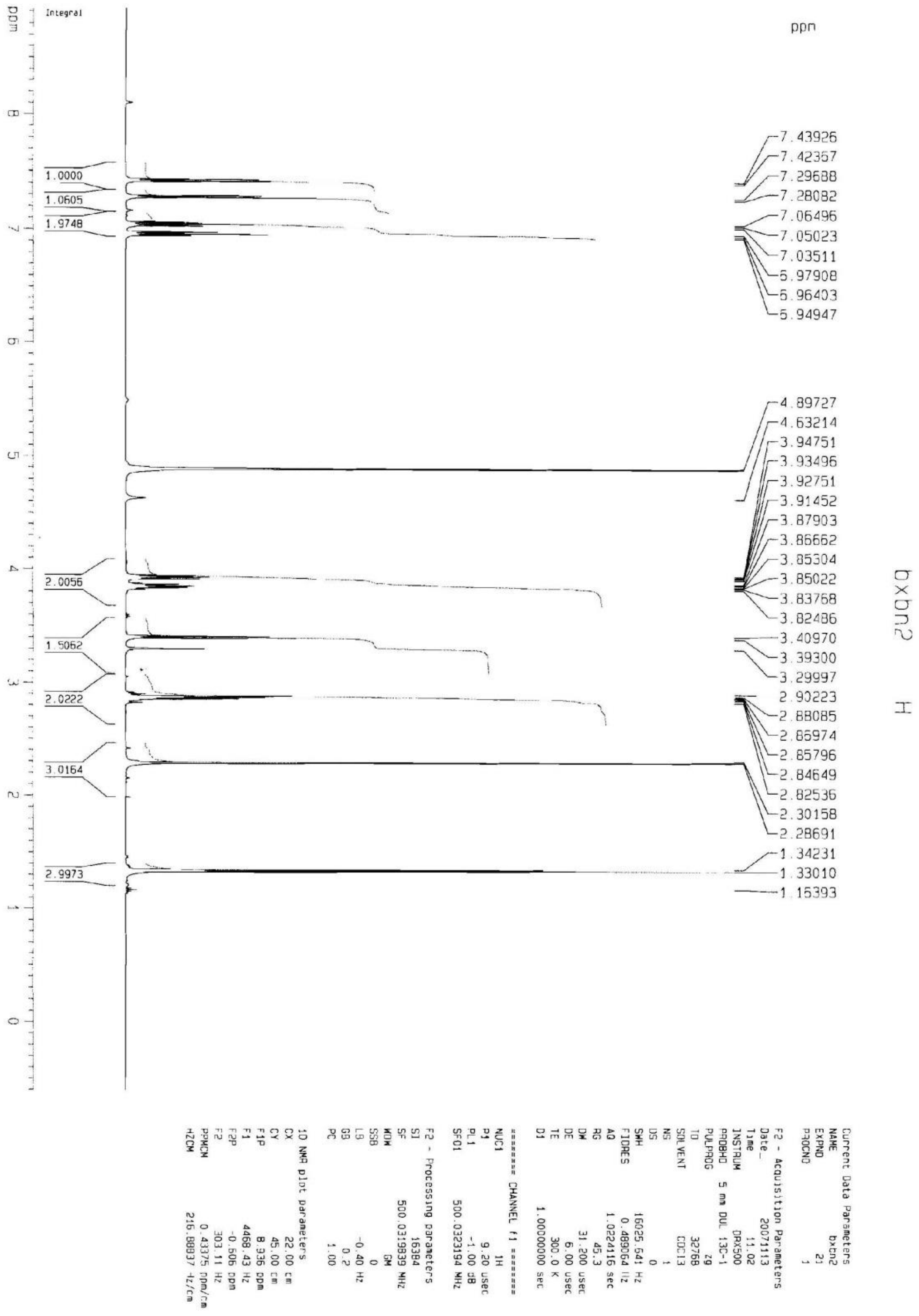

Figure S84. ^{1}H NMR for compound **22**

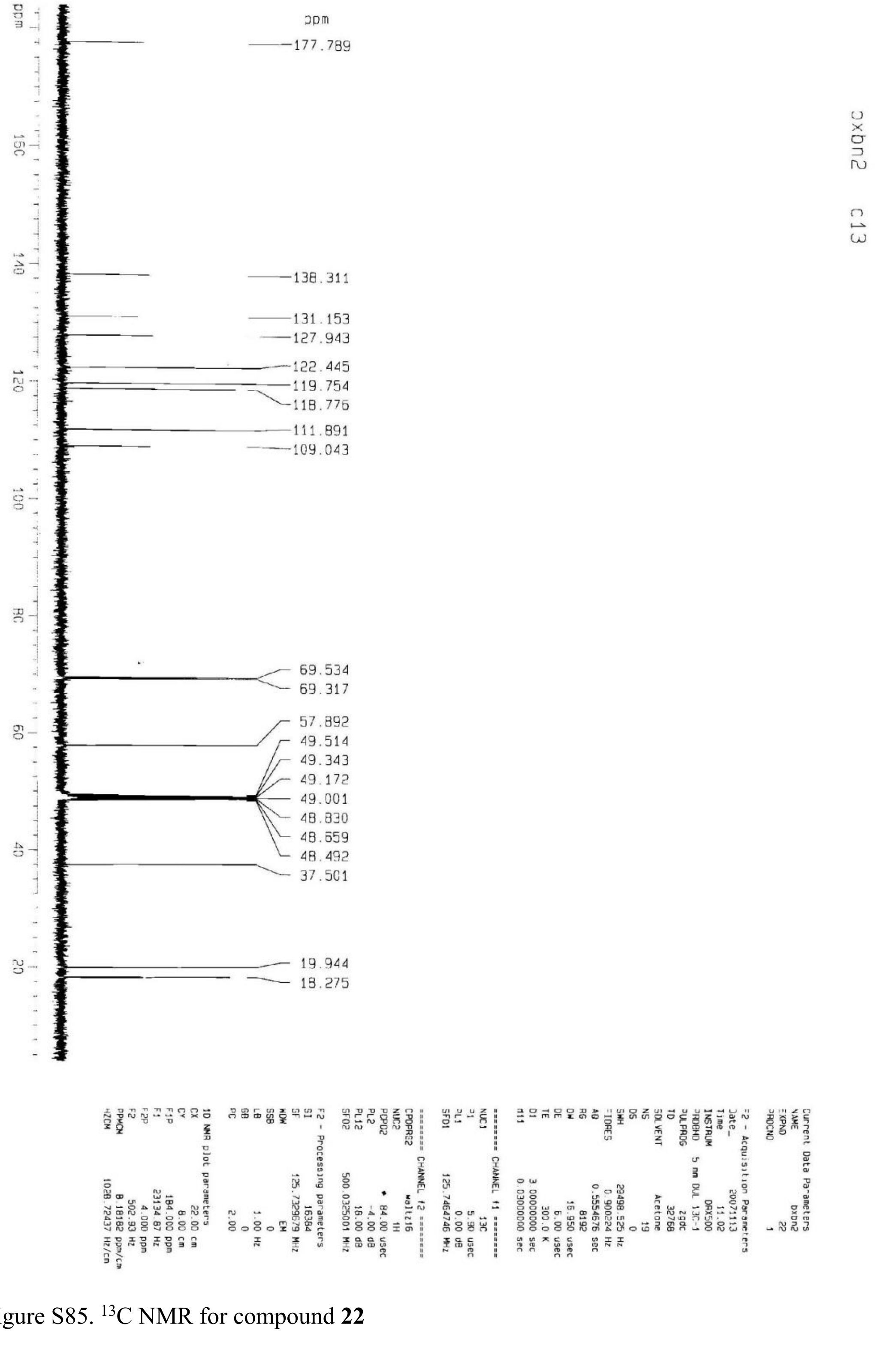

Figure S85. ^{13}C NMR for compound **22**

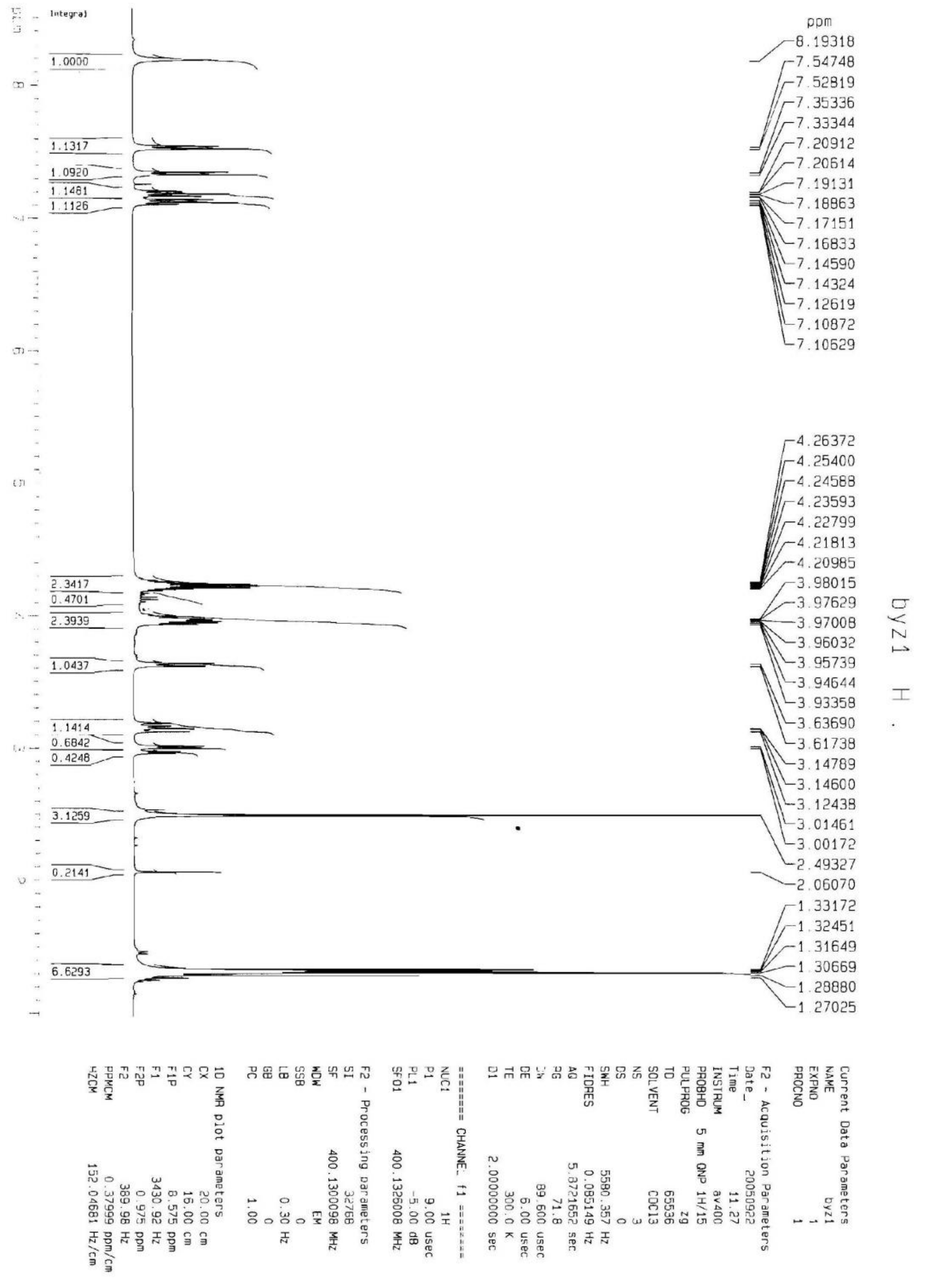

Figure S86. ^{1}H NMR for compound **23**

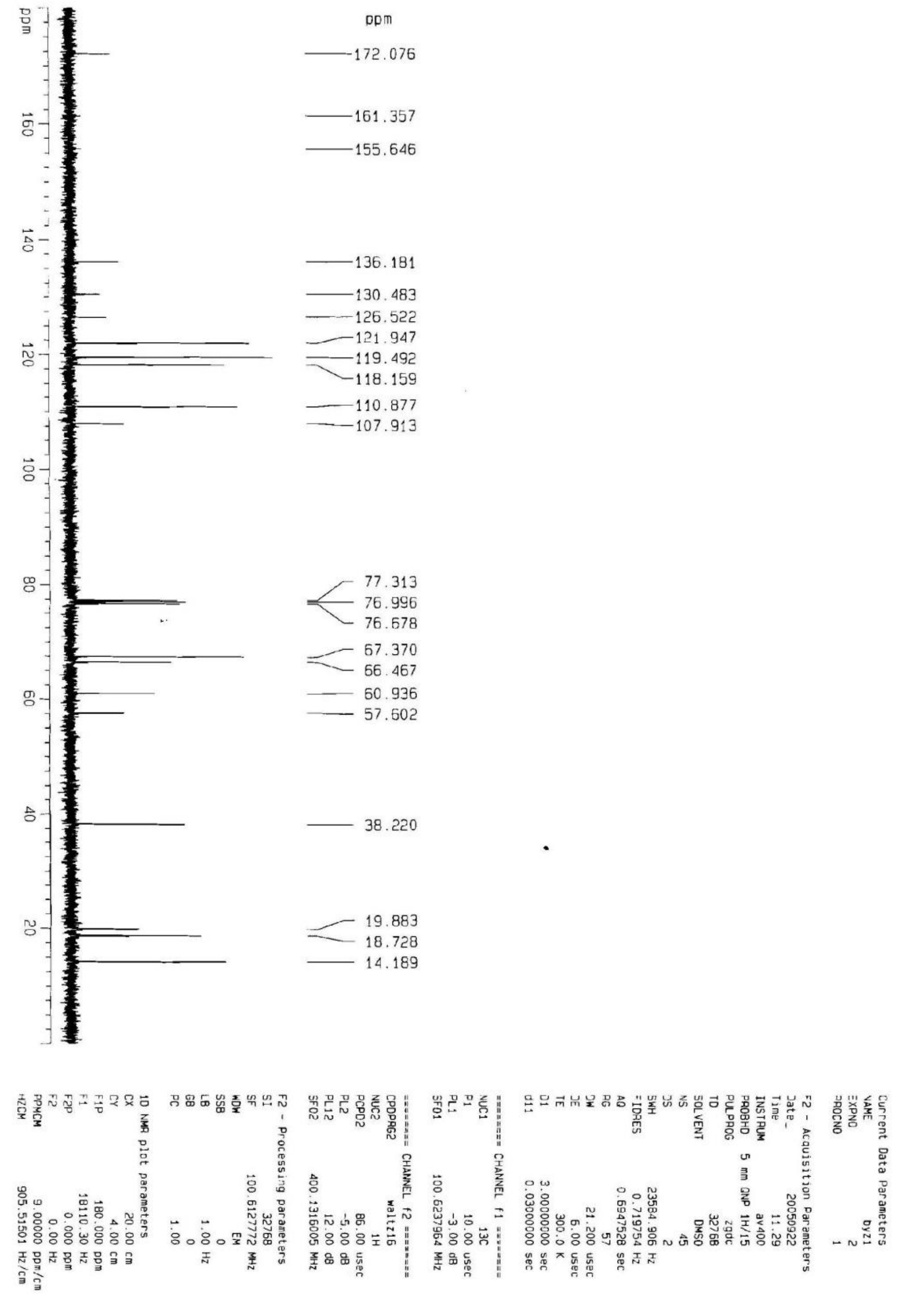

Figure S87. ^{13}C NMR for compound **23.**

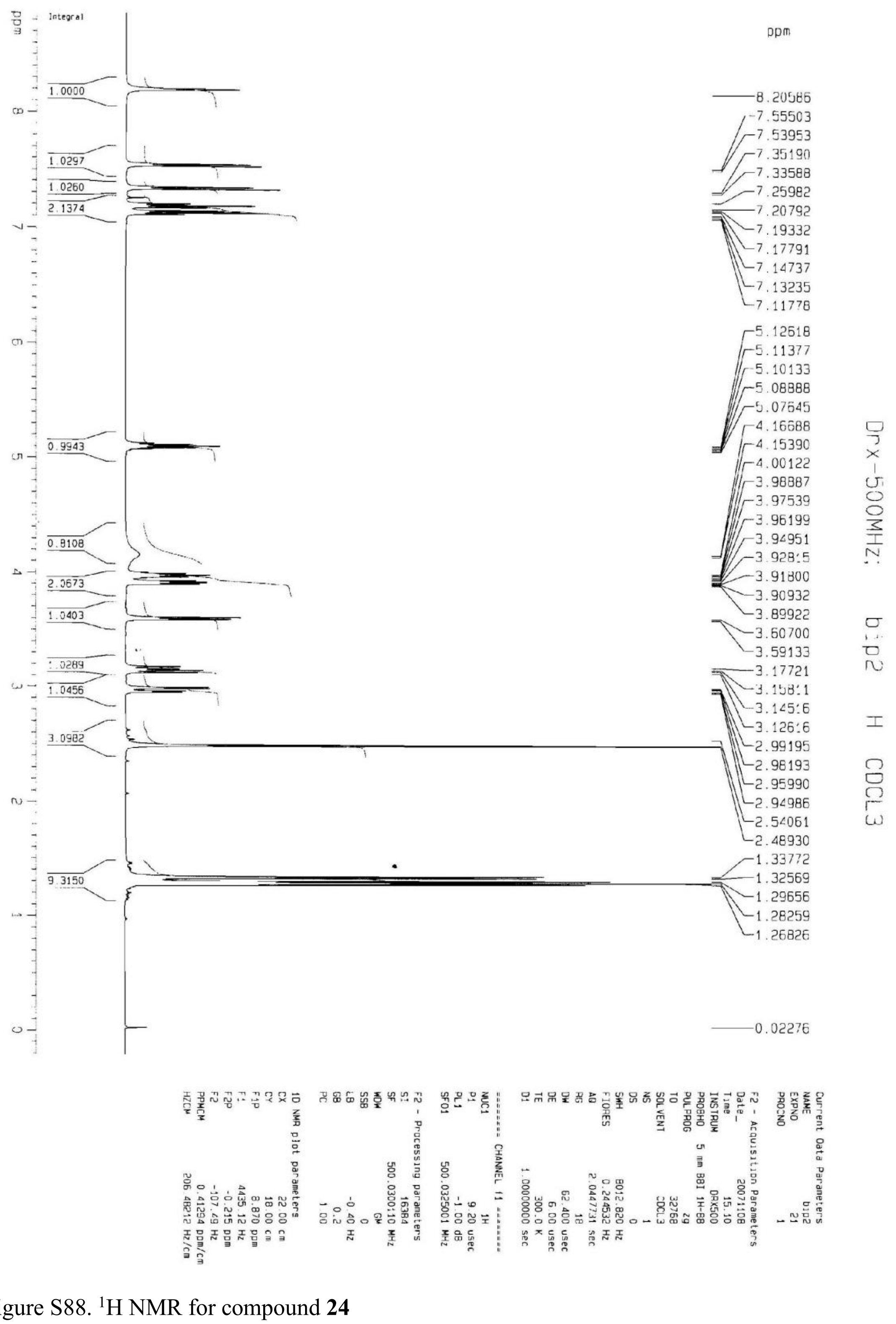

Figure S88. ^{1}H NMR for compound **24**

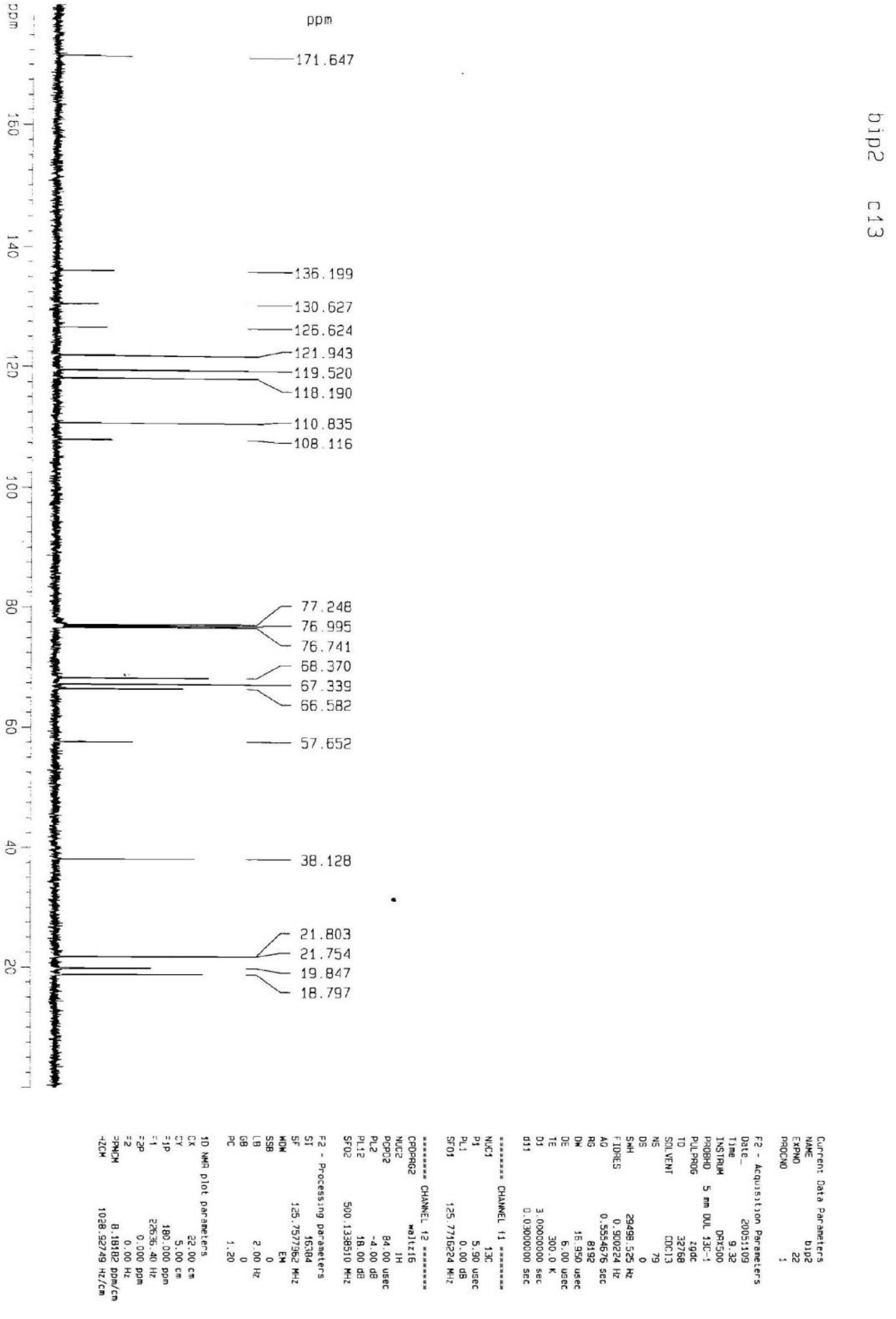

Figure S89. ^{13}C NMR for compound **24**

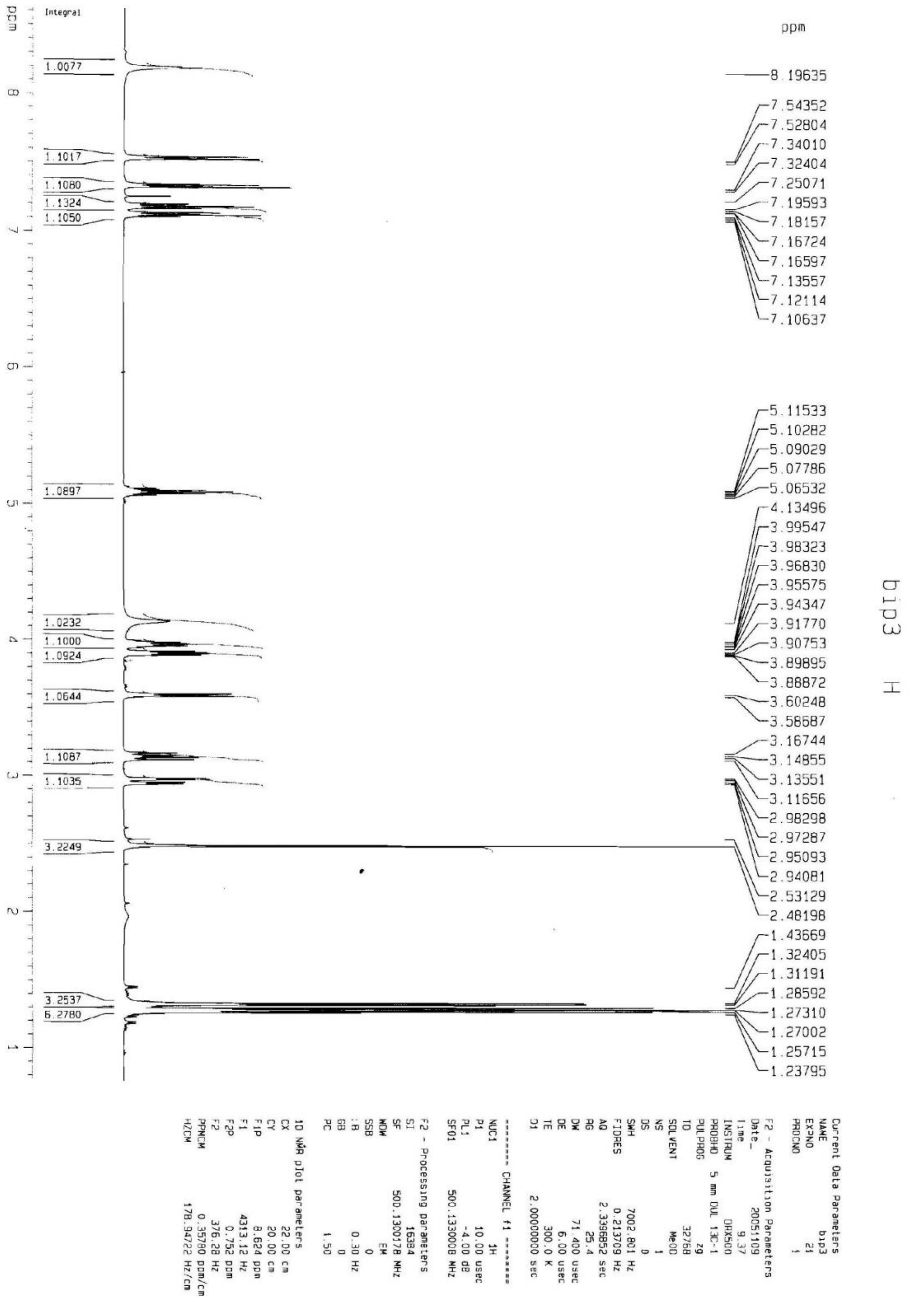

Figure S90. ^{1}H NMR for compound **25**

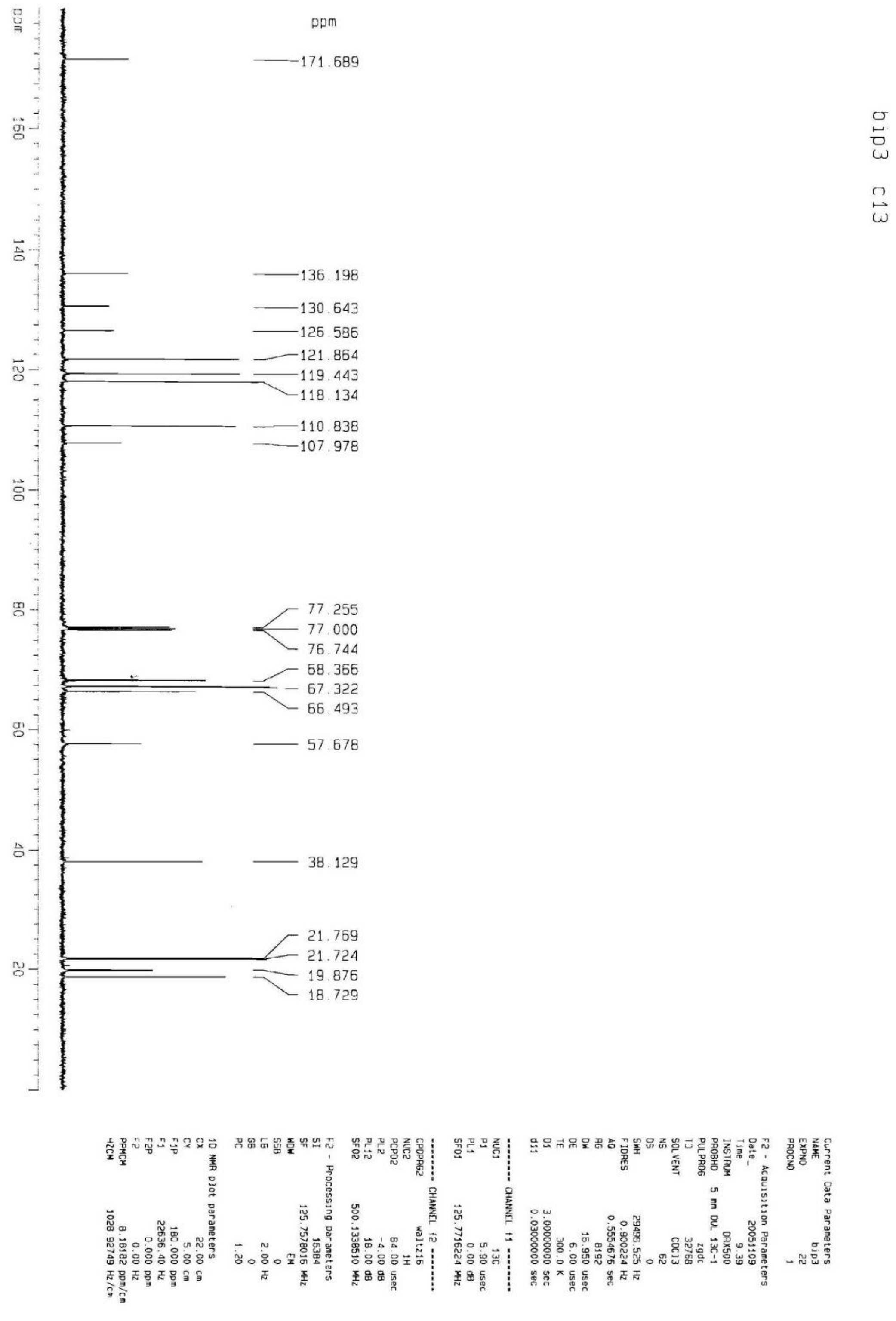

Figure S91. ^{13}C NMR for compound **25**

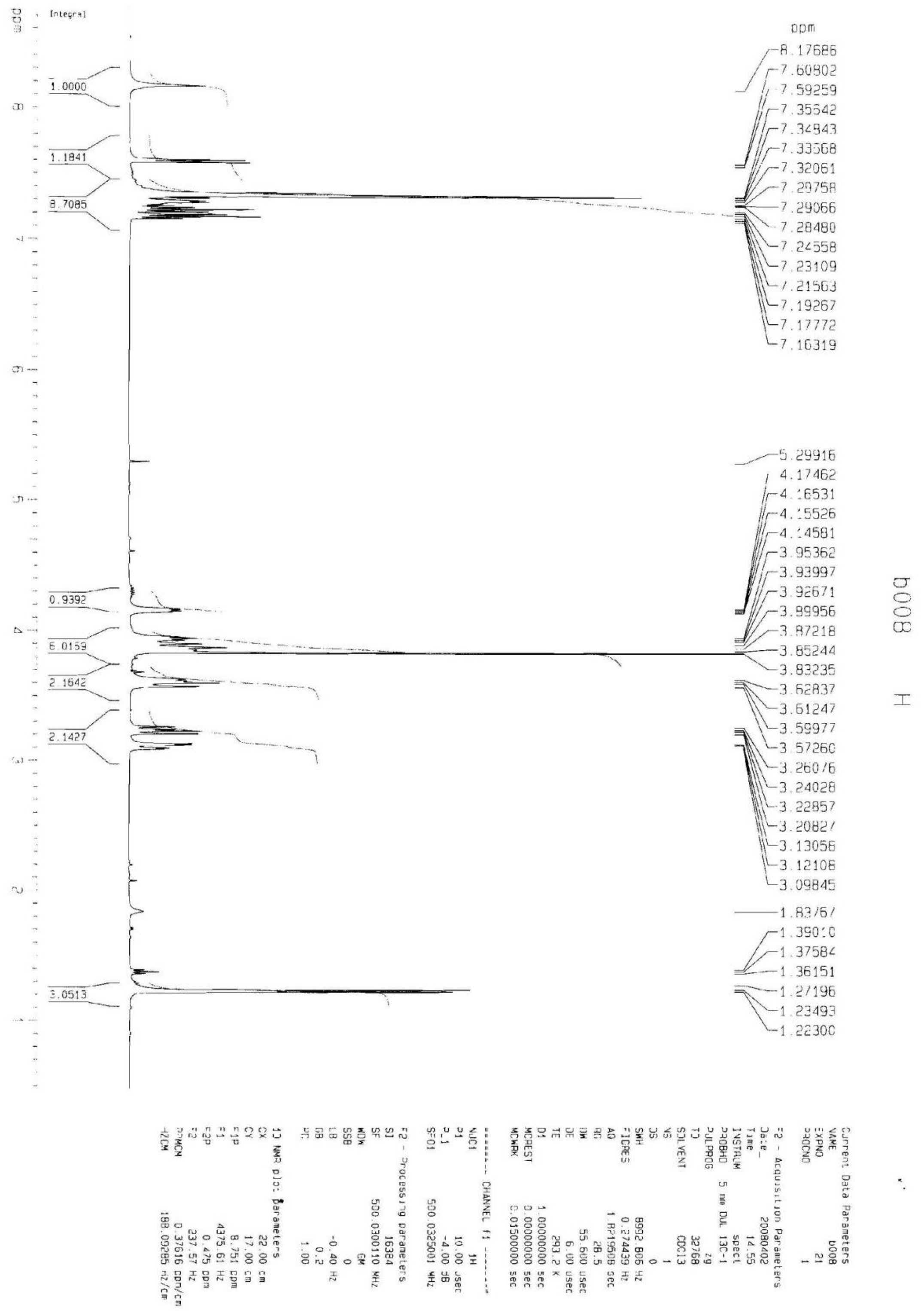

Figure S92. ^{1}H NMR for compound **26**

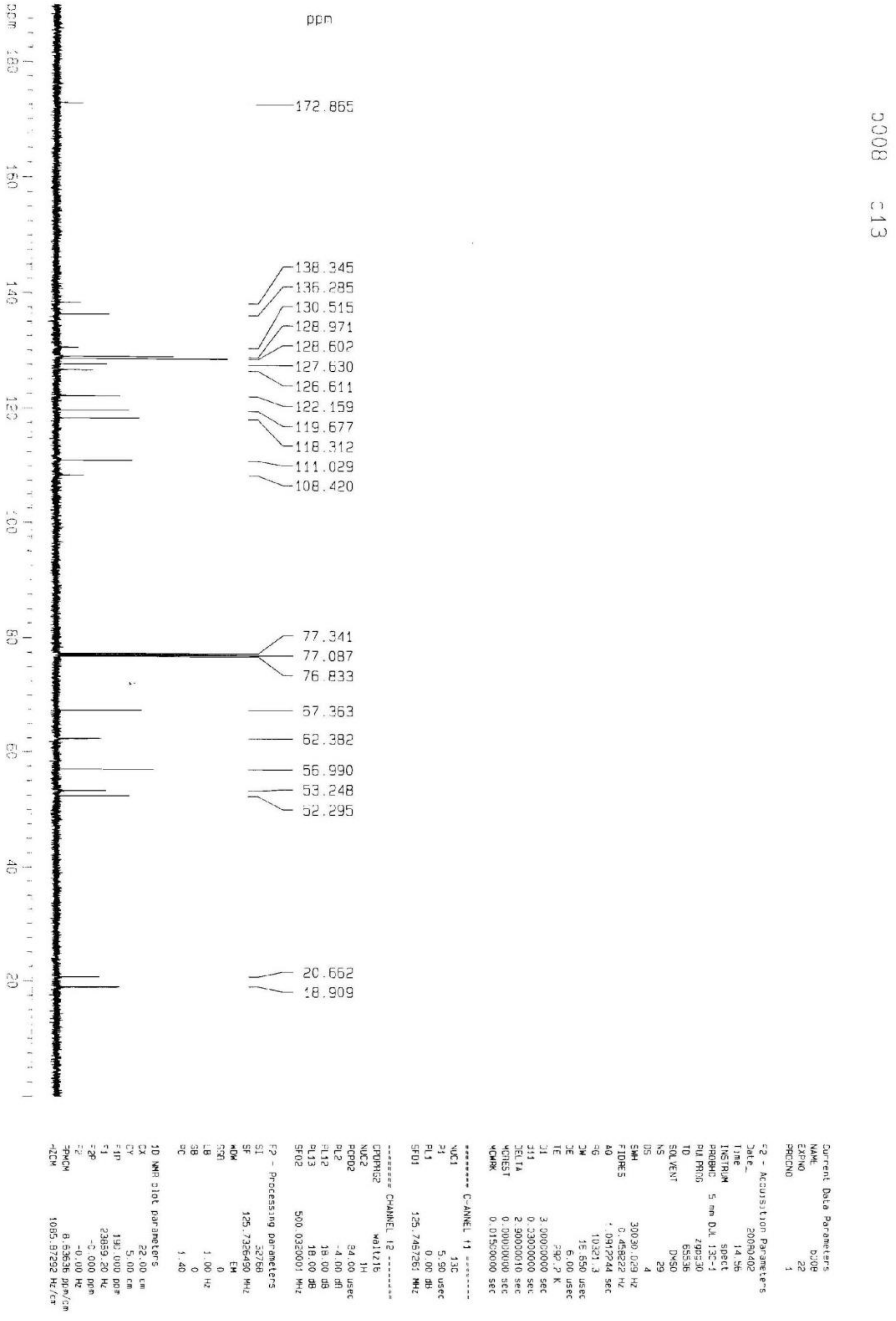

Figure S93. ^{13}C NMR for compound **26**

IR spectra for compounds 21 to 26

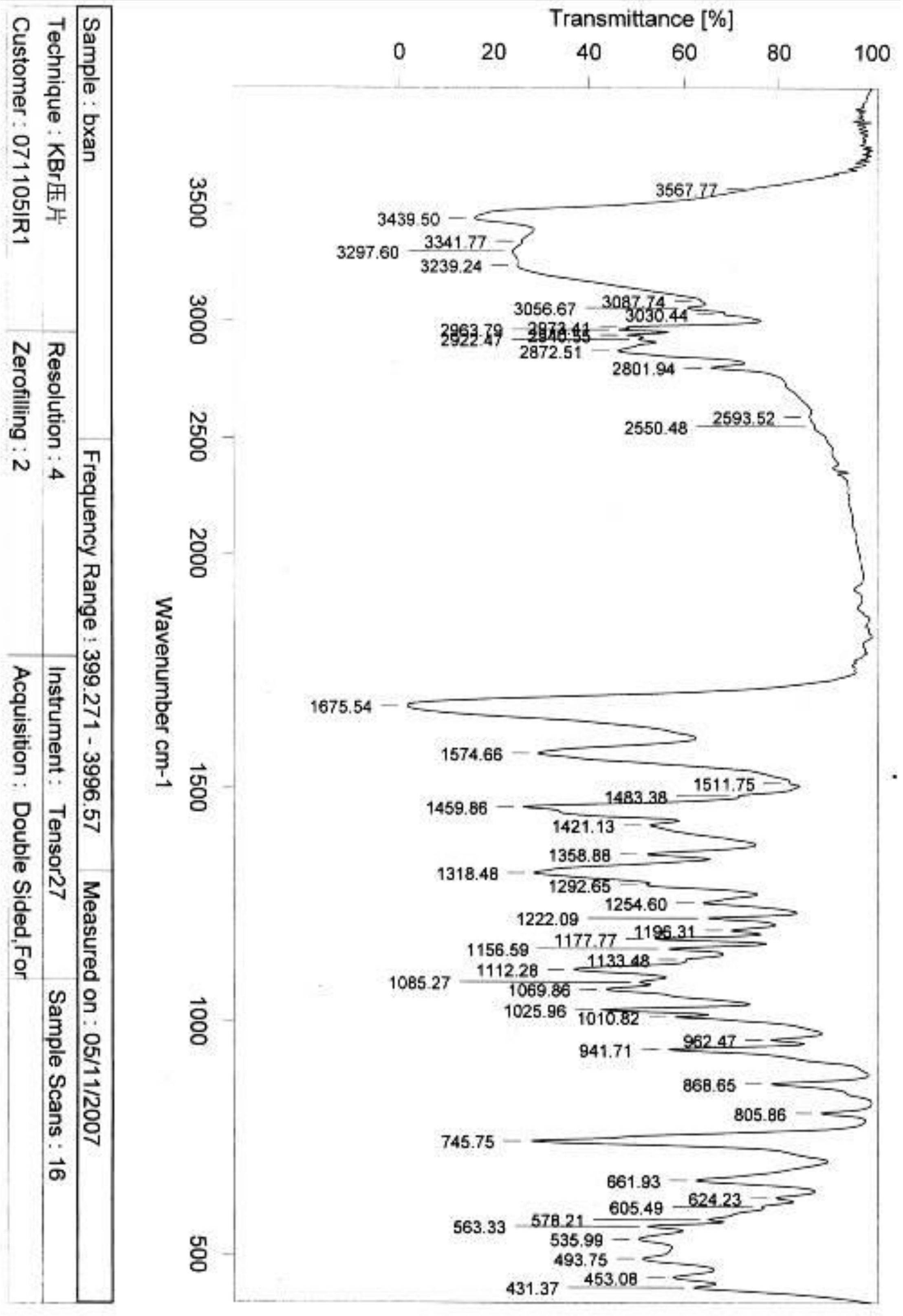

Figure S94. IR for compound **21**.

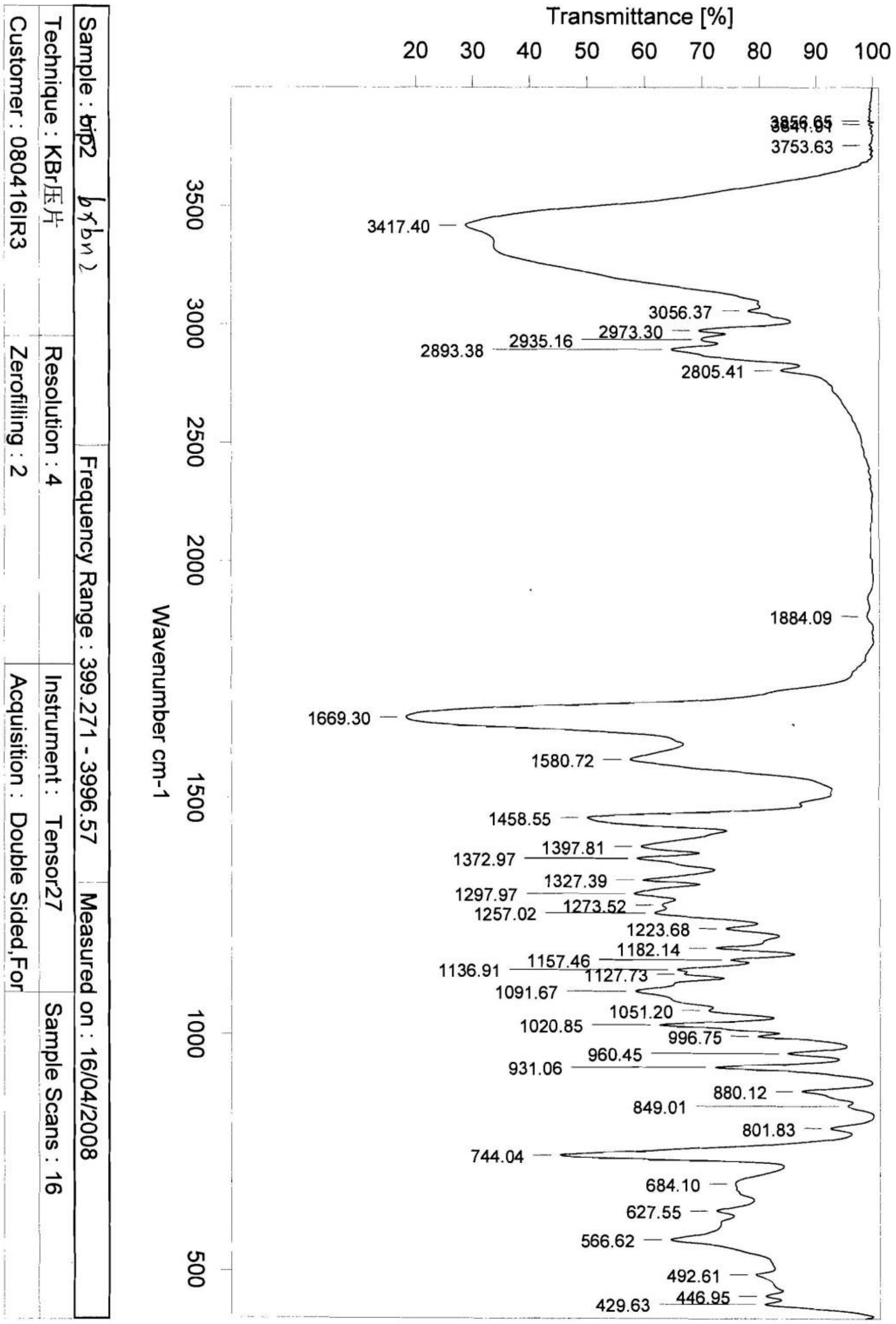

Figure S95. IR for compound **22**

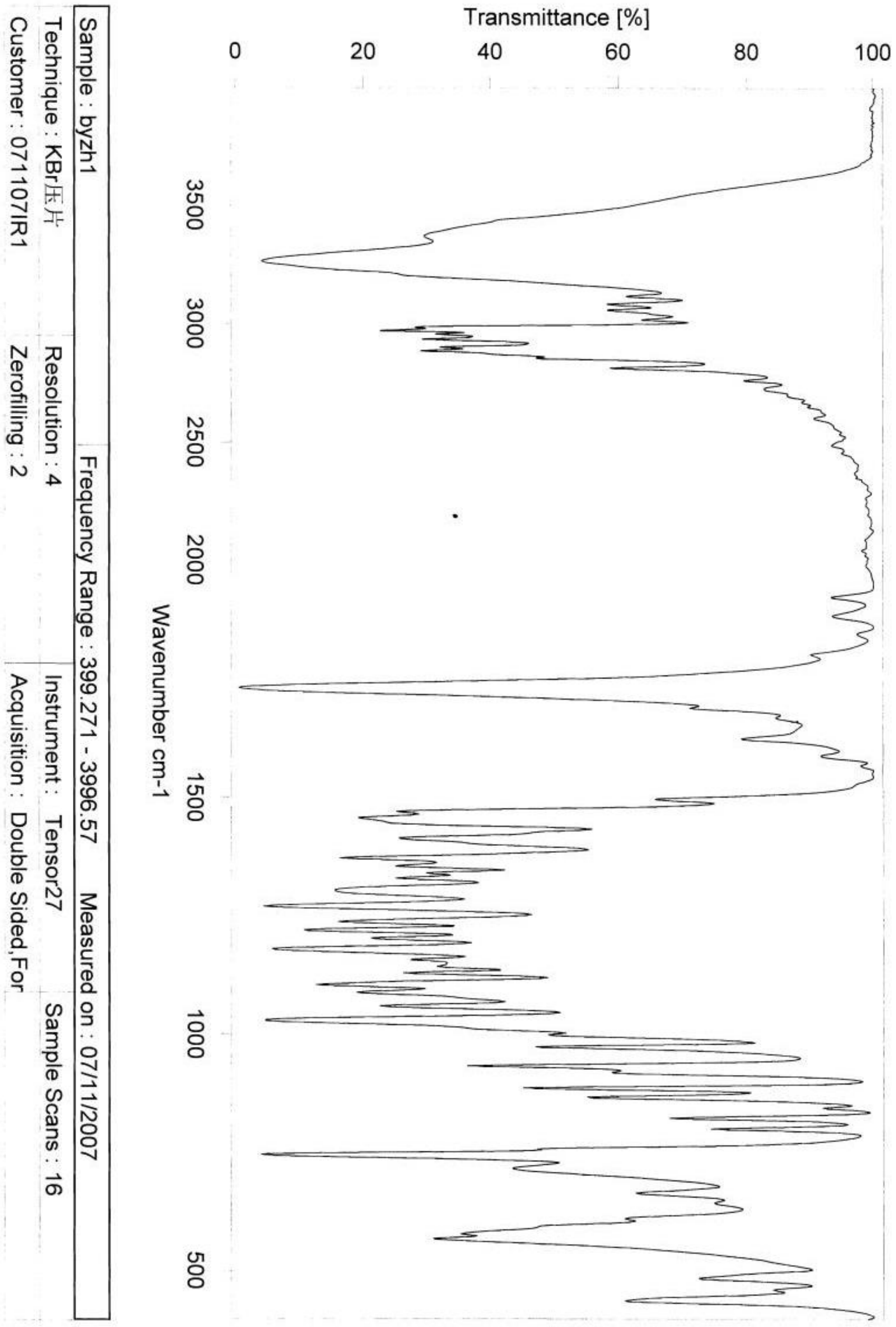

Figure S96. IR for compound **23**

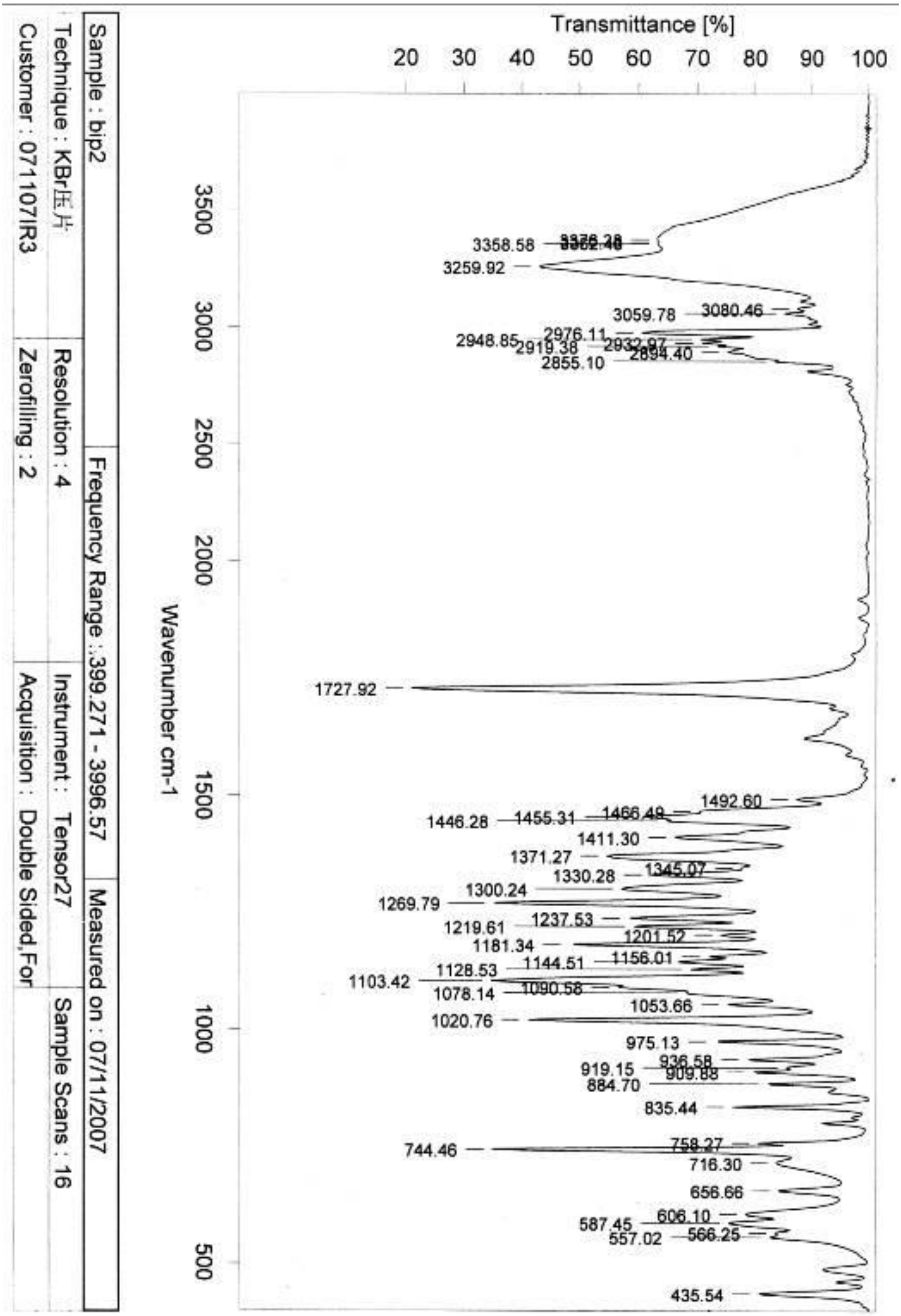

Figure S97. IR for compound **24**

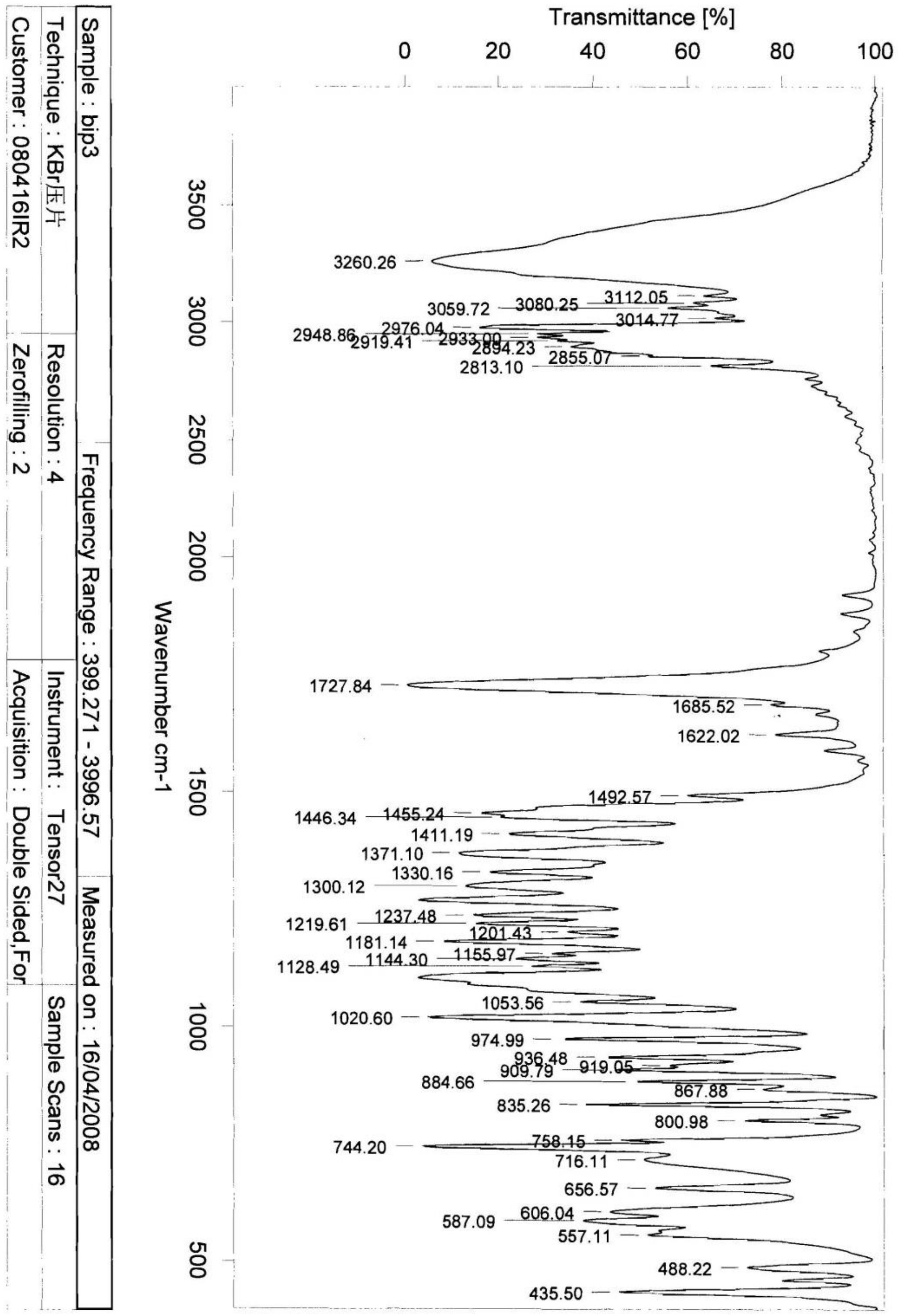

Figure S98. IR for compound **25**

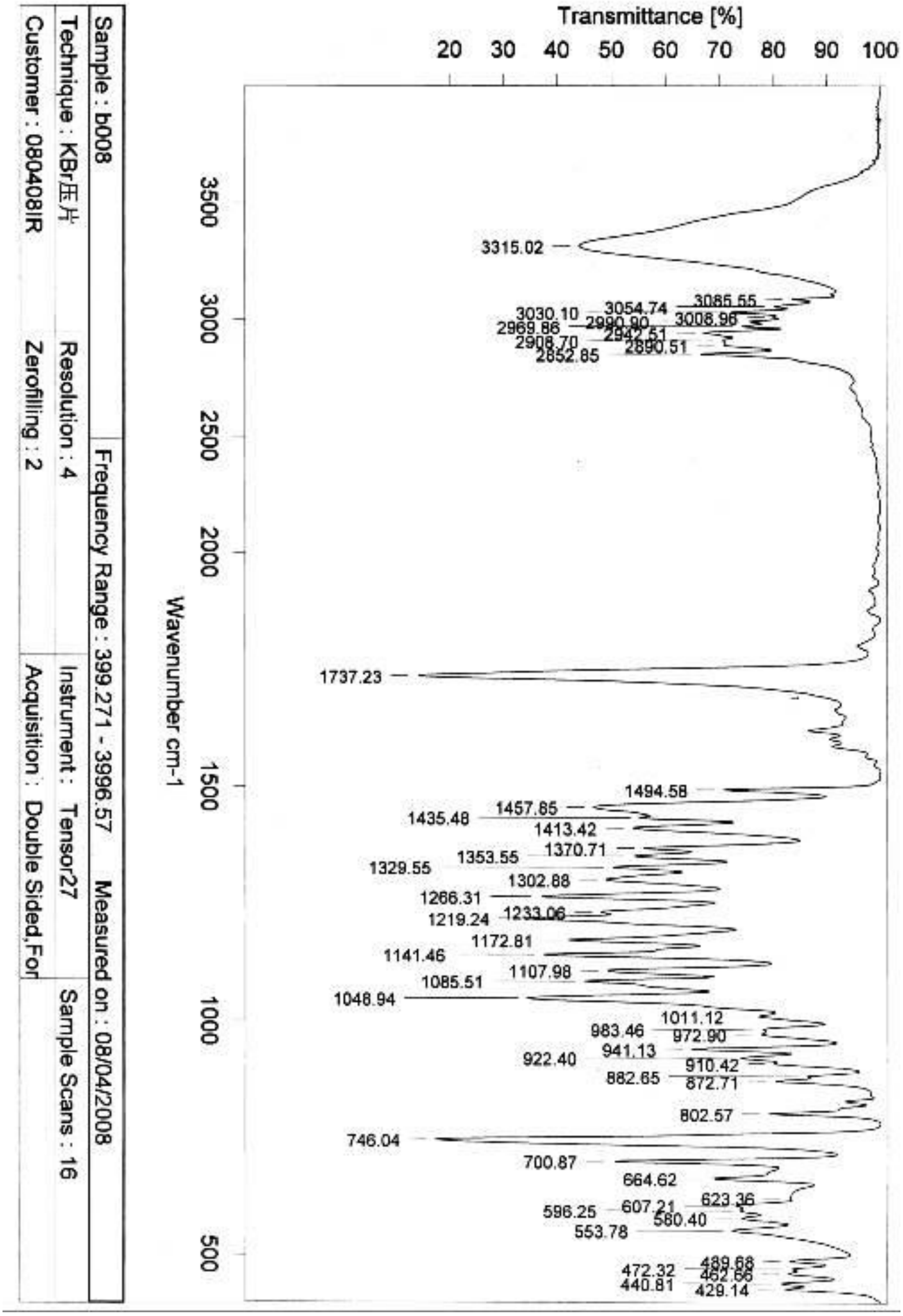

Figure S99. IR for compound **26**

The bong length changes for compounds 30 to 37

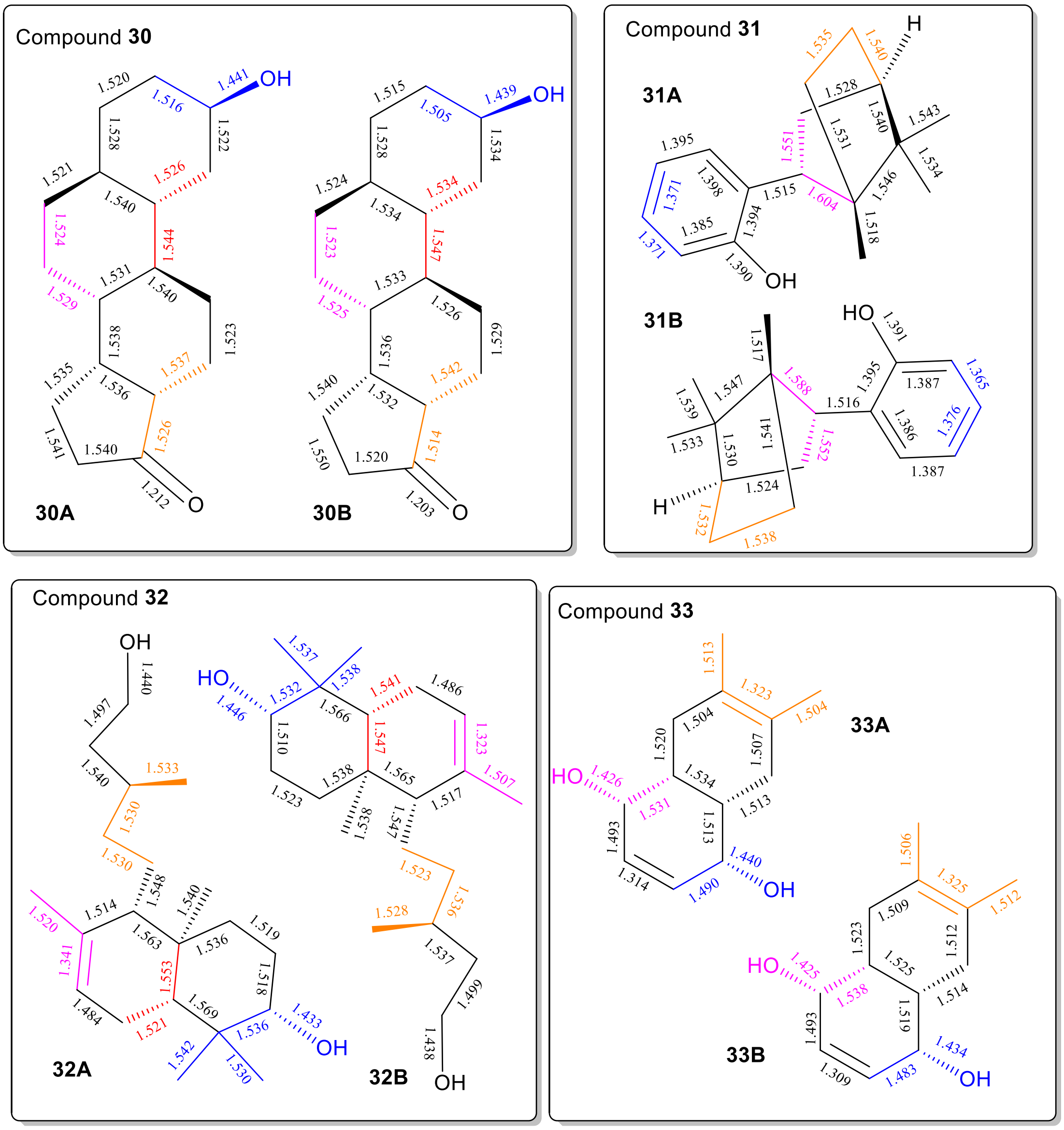

Figure S100. The bond length changes of compounds **30** to **37**.

Compound **34**

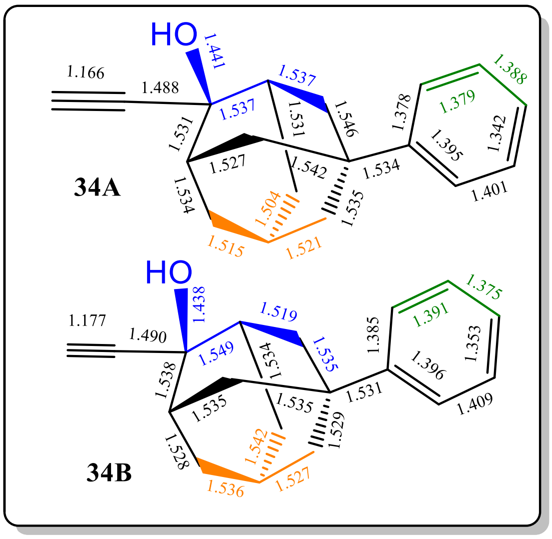

Compound **35**

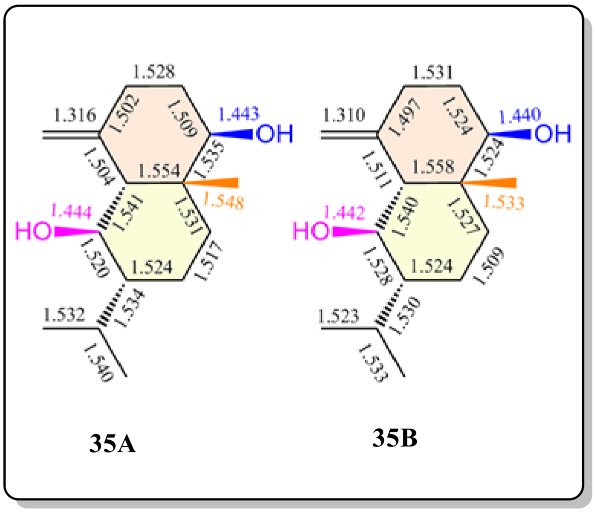

Compound **36**

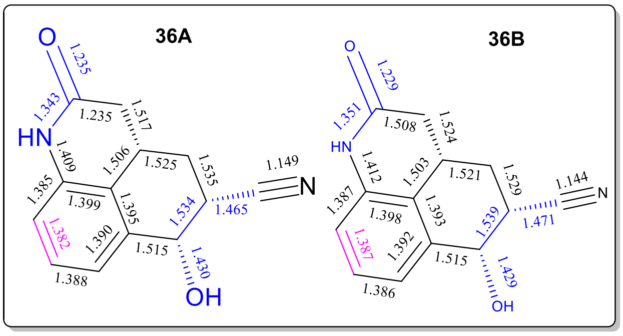

Compound **37**

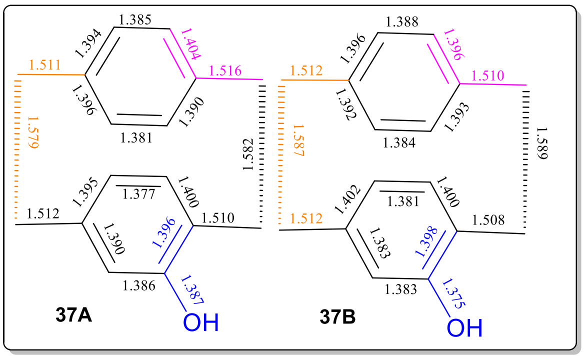

Figure 100. continued

2D NMR for compounds 8, 13 and 9

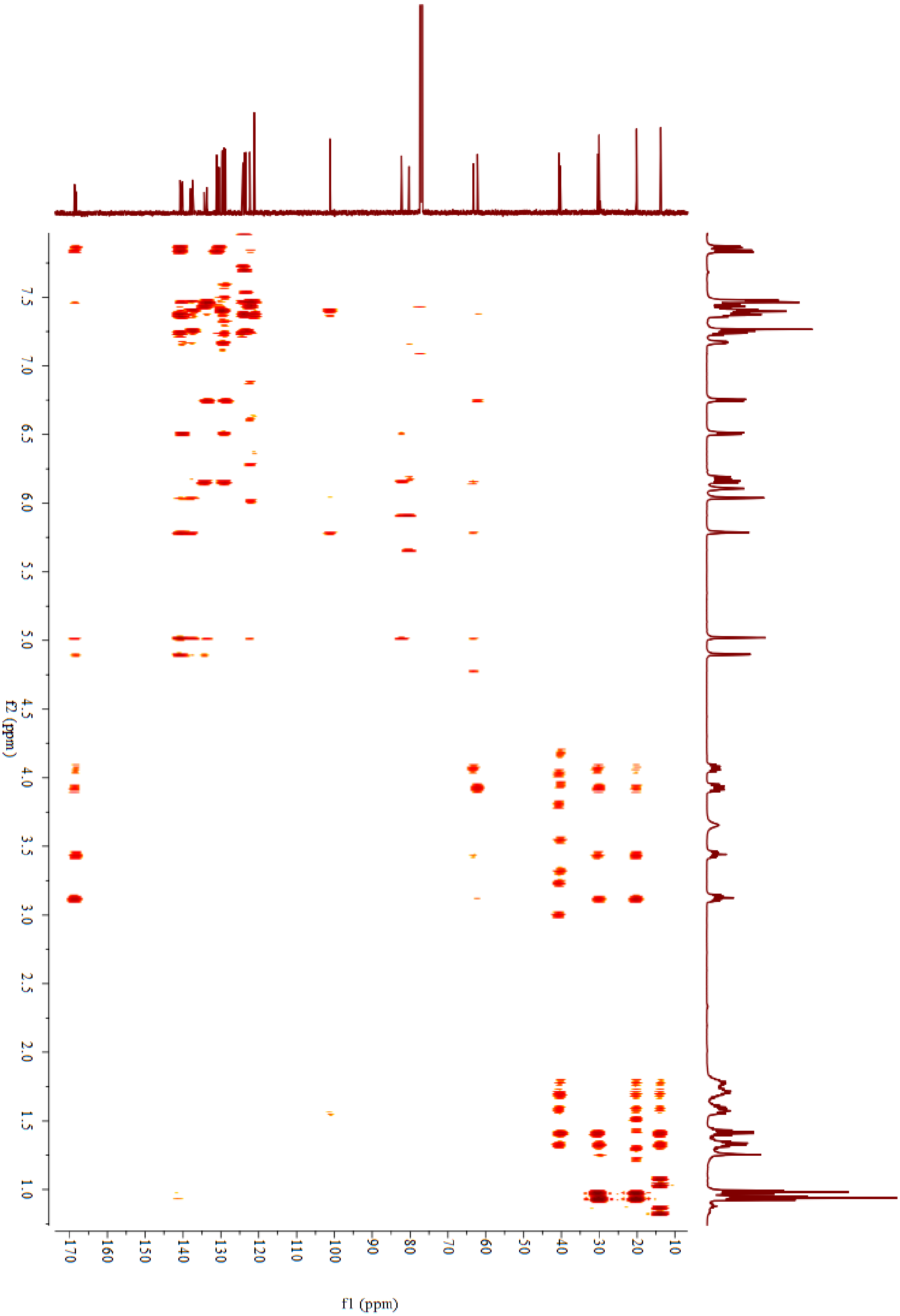

Figure S101. HMBC of compound **8.**

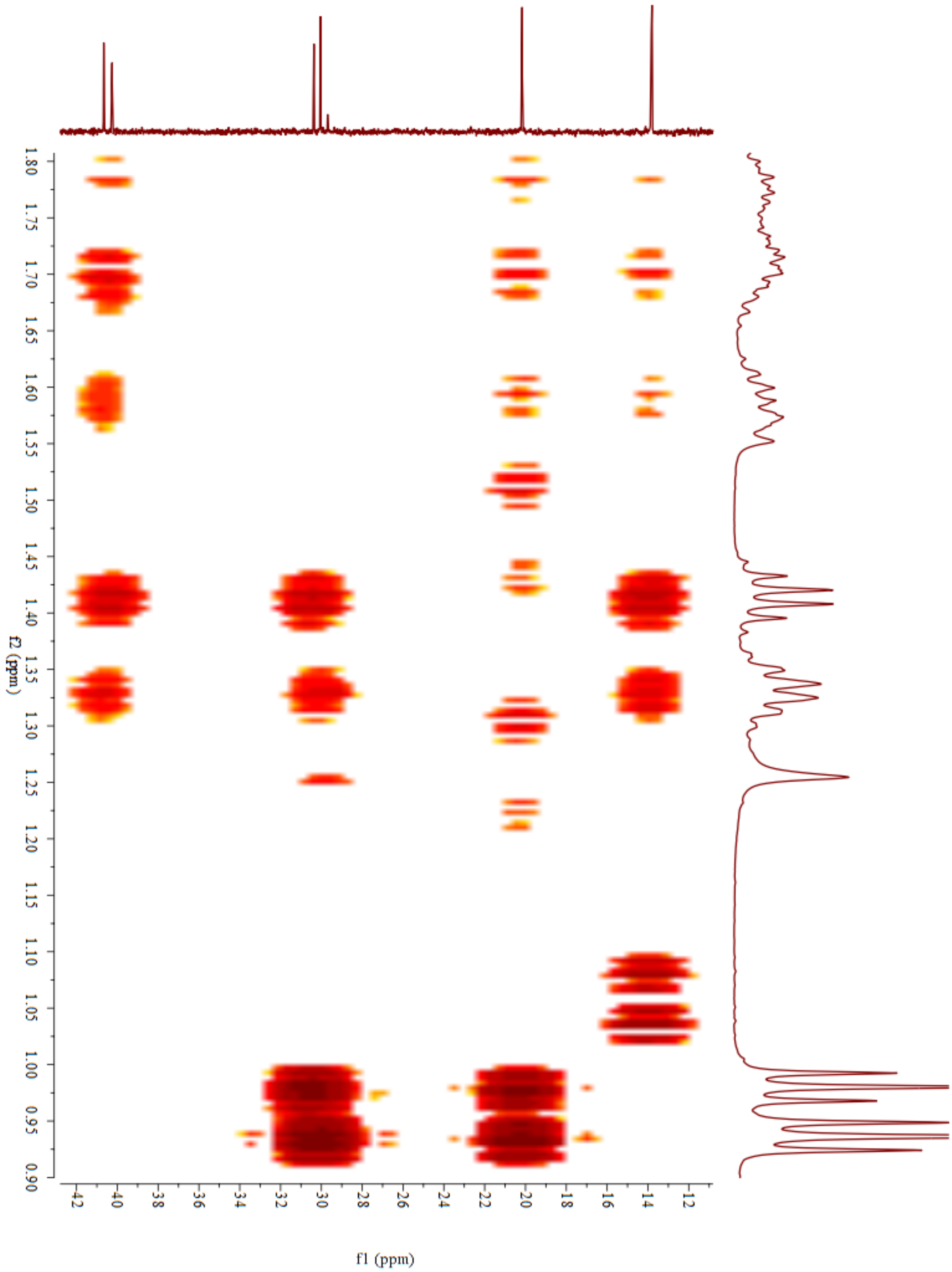

Figure S101. Continued

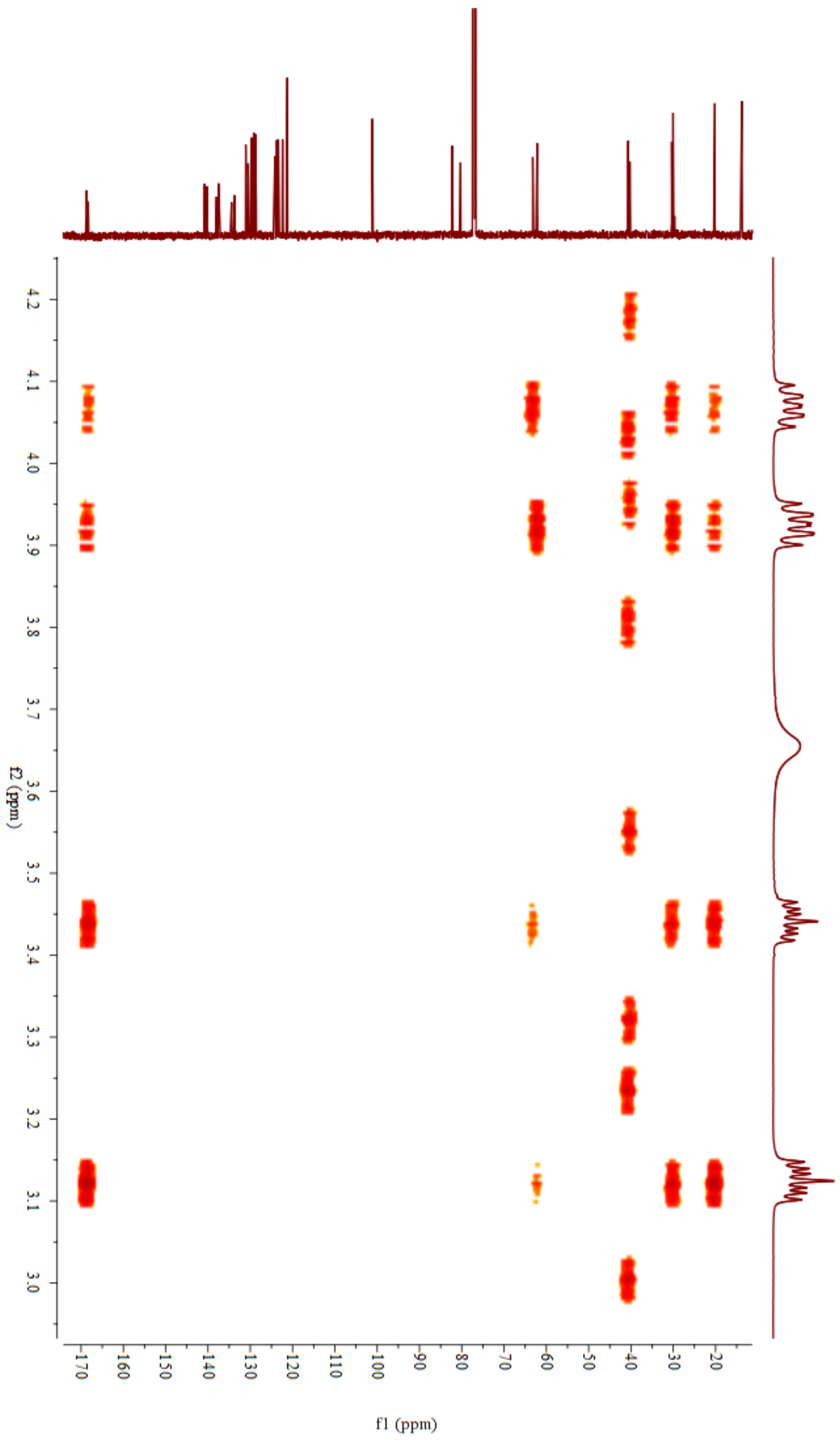

Figure S101. Continued

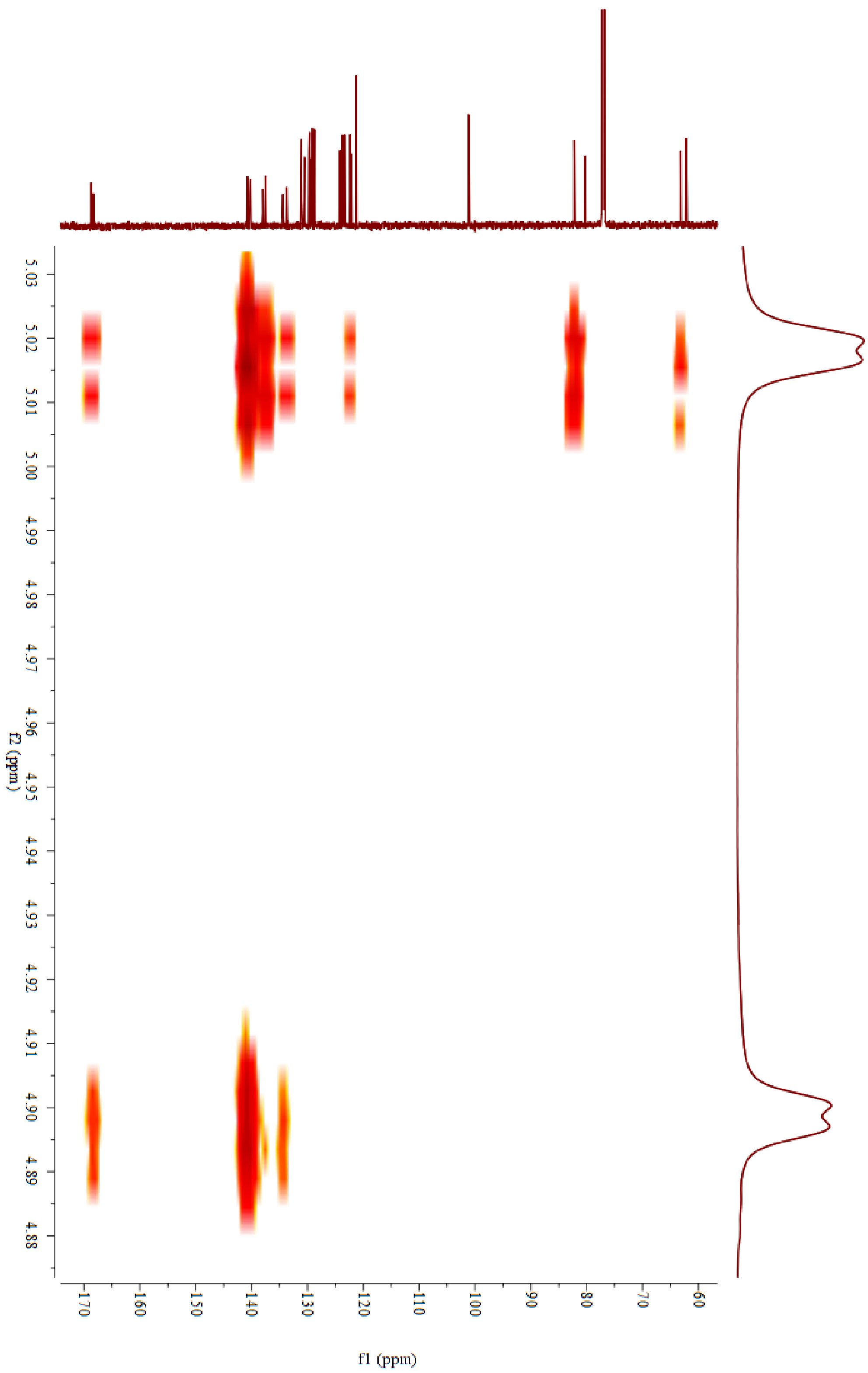

Figure S101. Continued

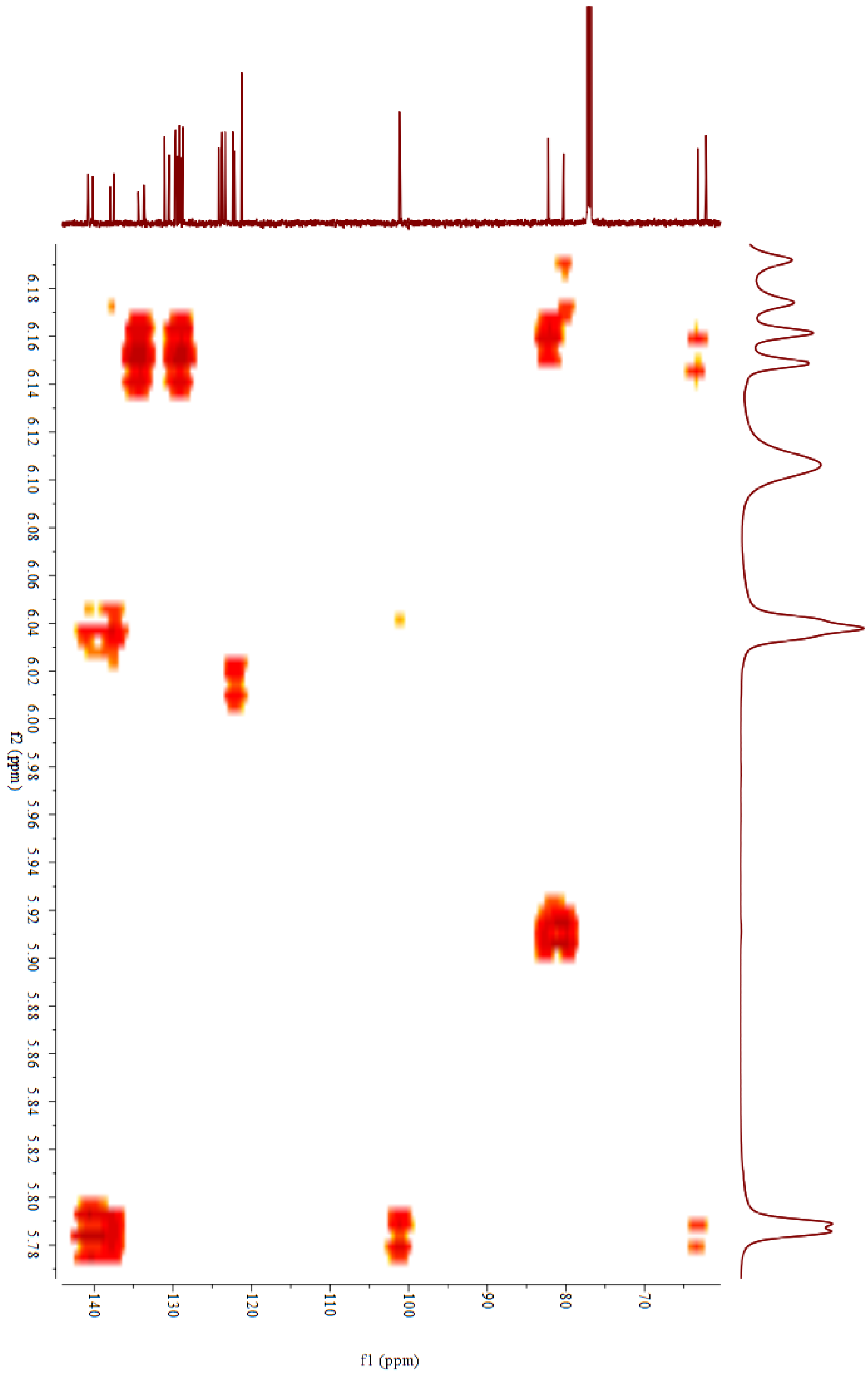

Figure S101. Continued

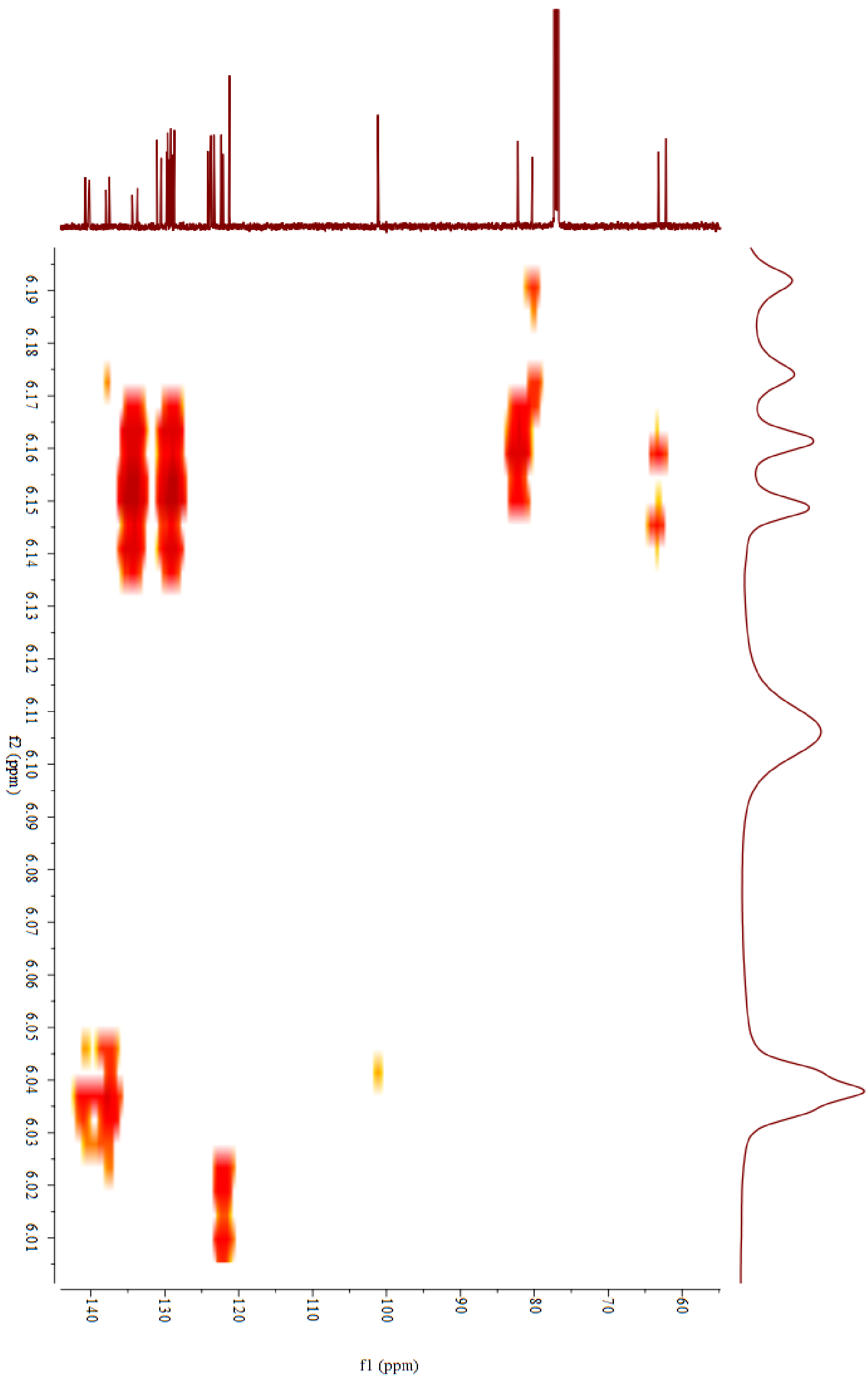

Figure S101. Continued

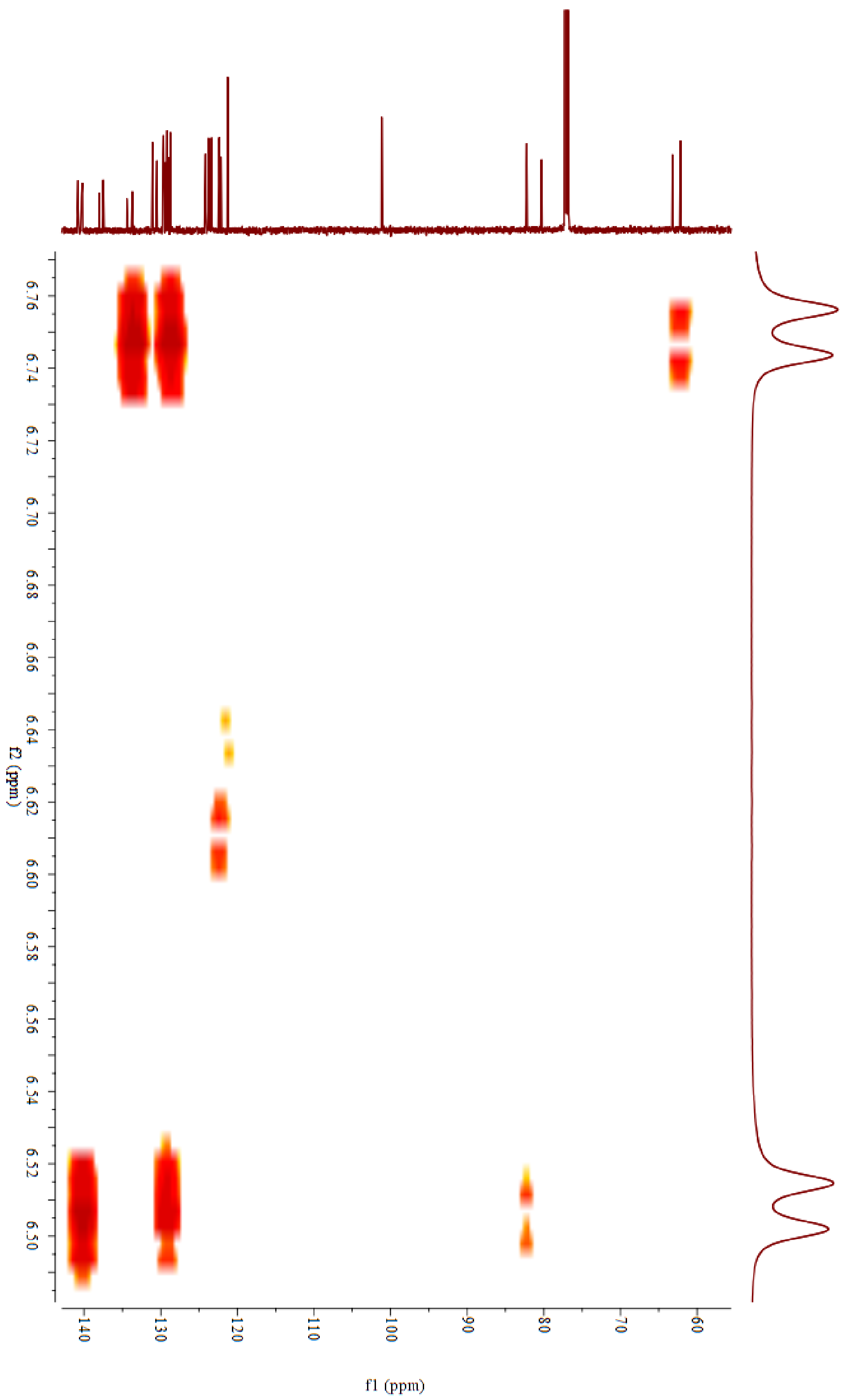

Figure S101. Continued

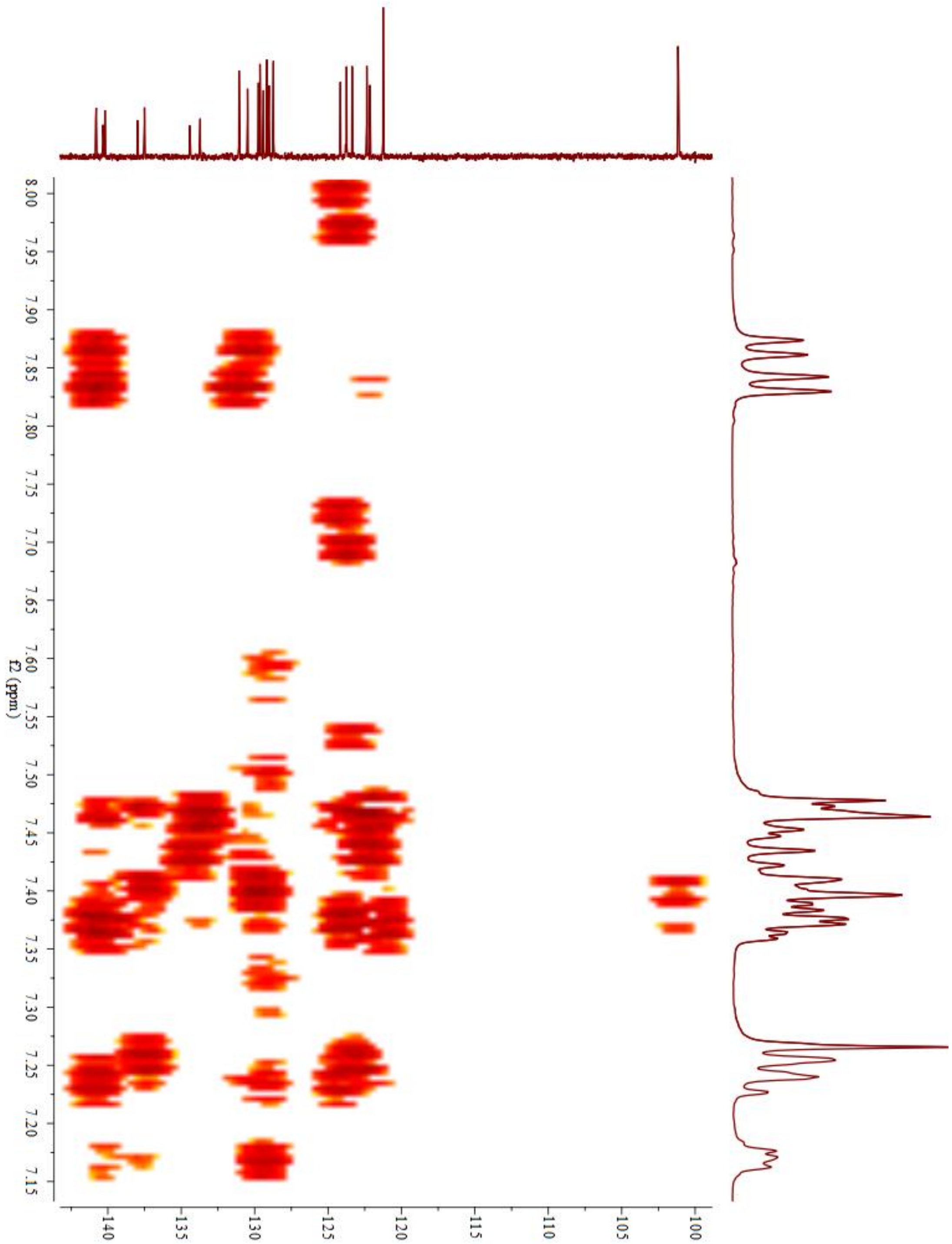

Figure S101. Continued

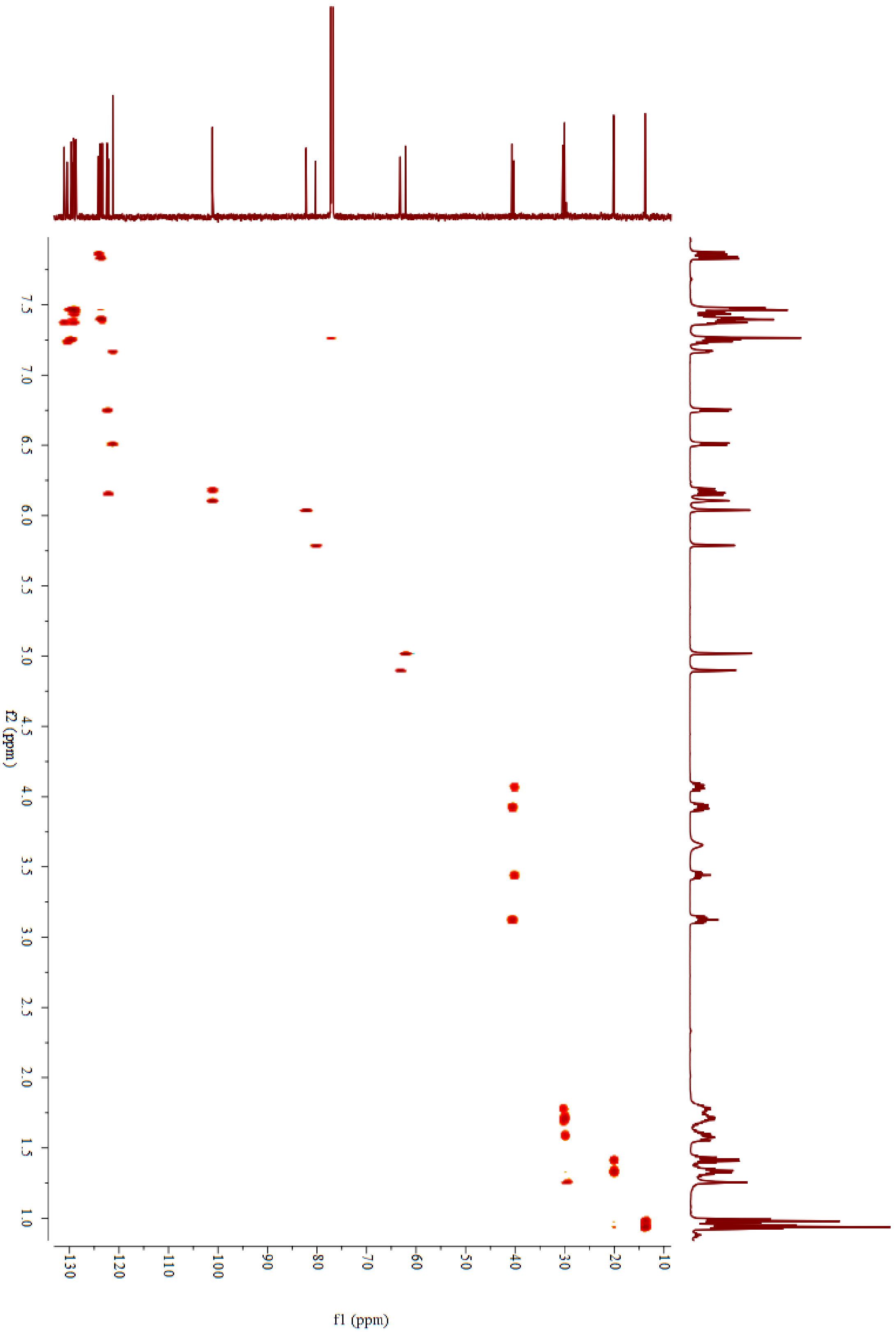

Figure S102. HSQC of compound **8.**

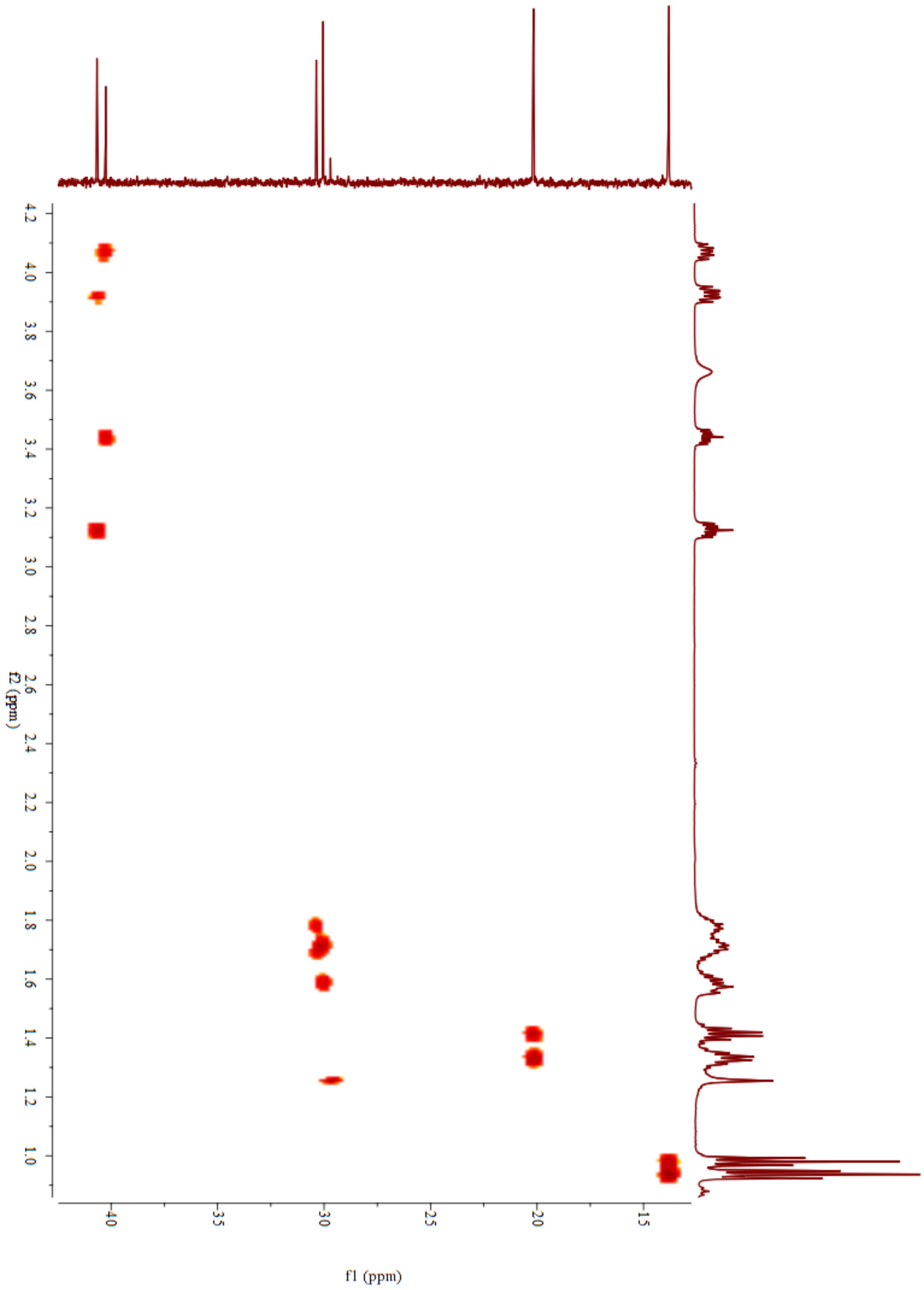

Figure S102. Continued

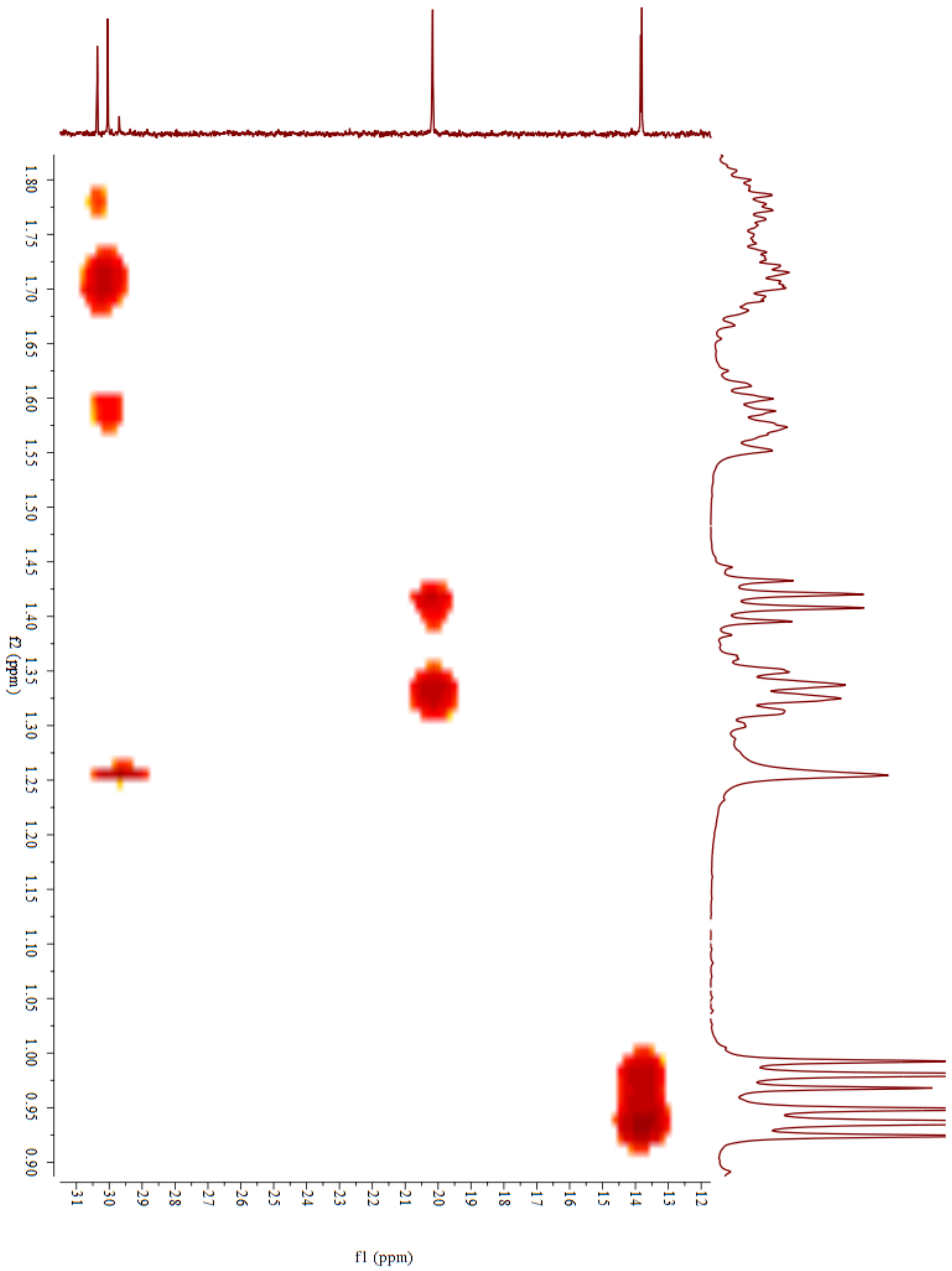

Figure S102. Continued

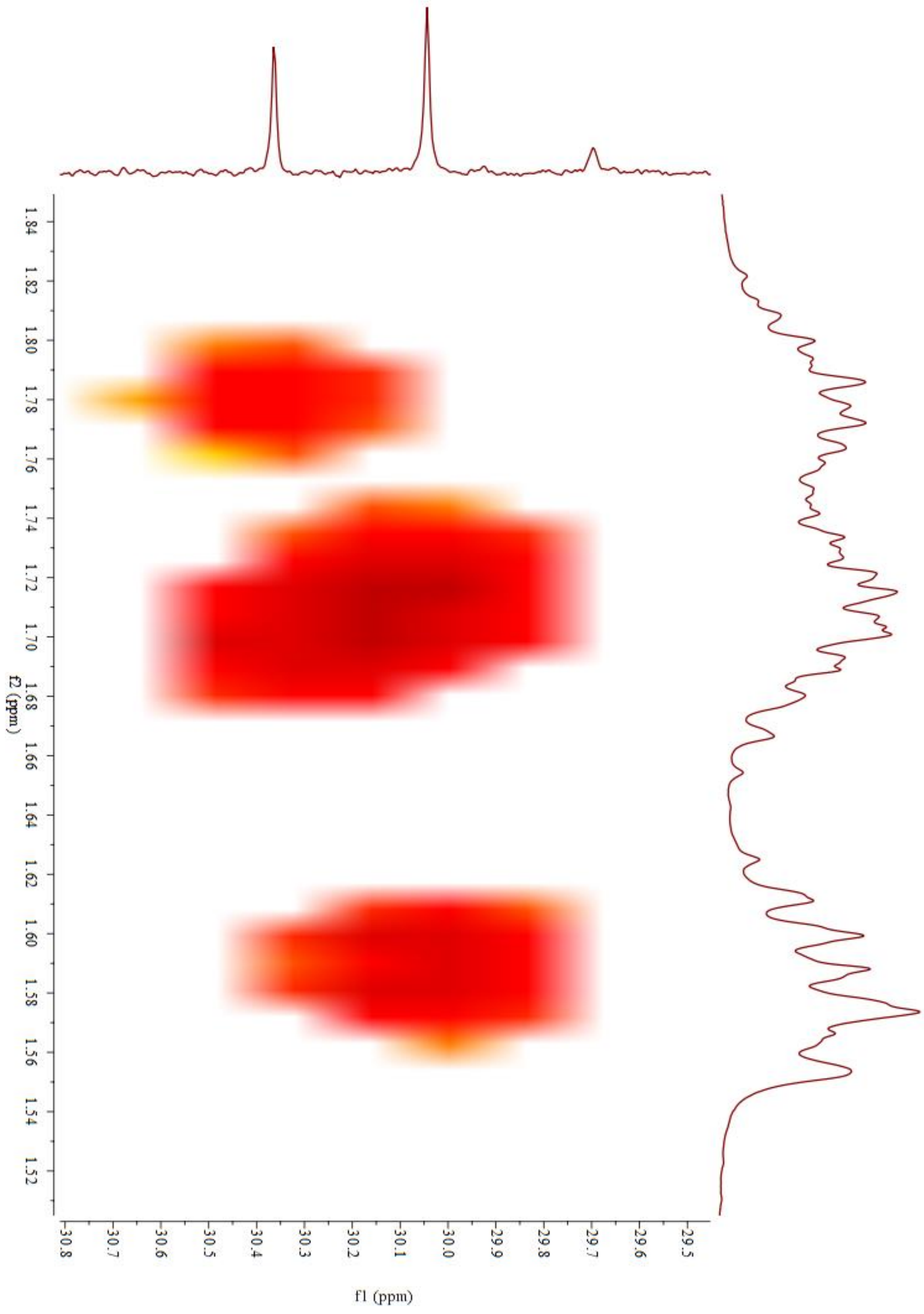

Figure S102. Continued

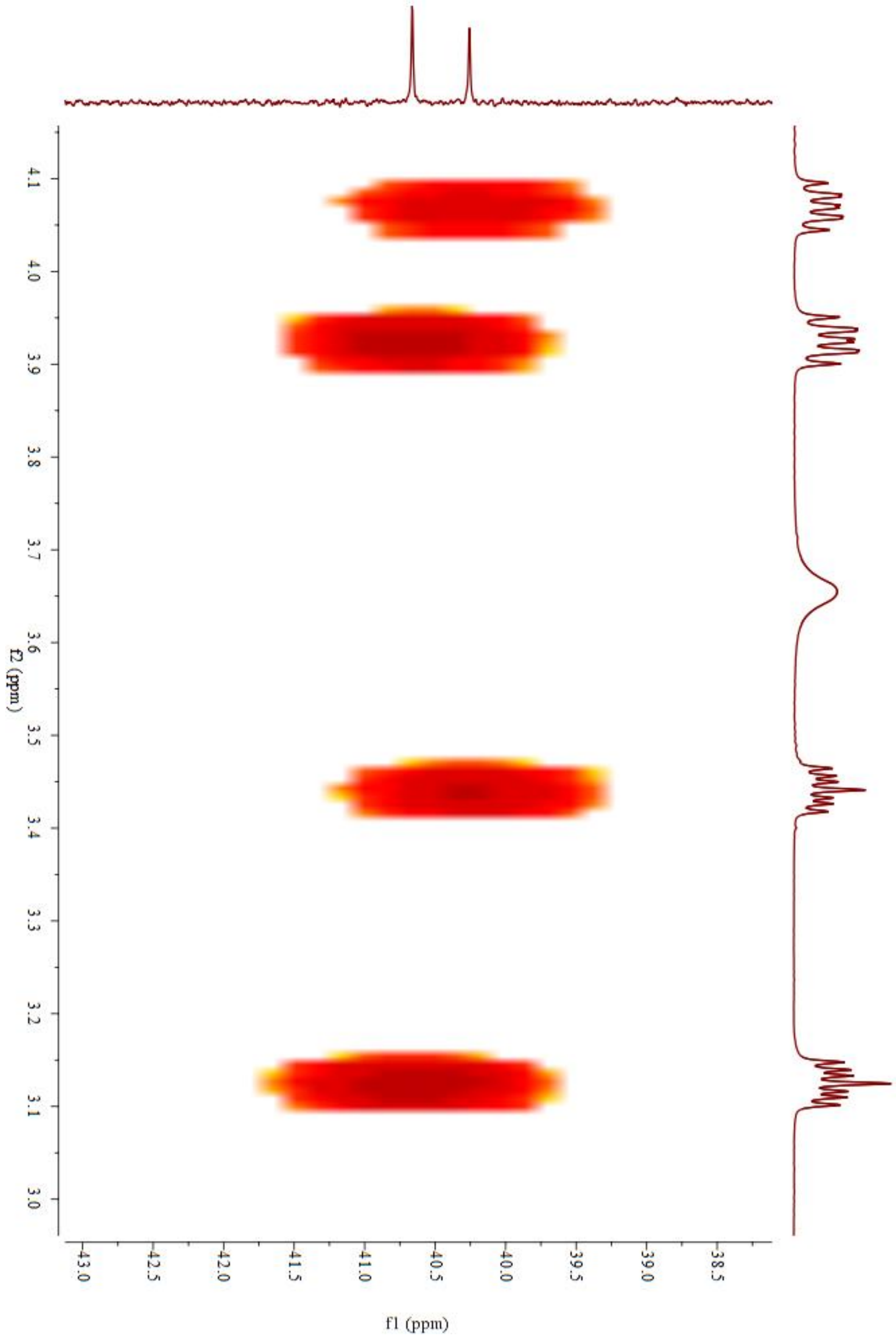

Figure S102. Continued

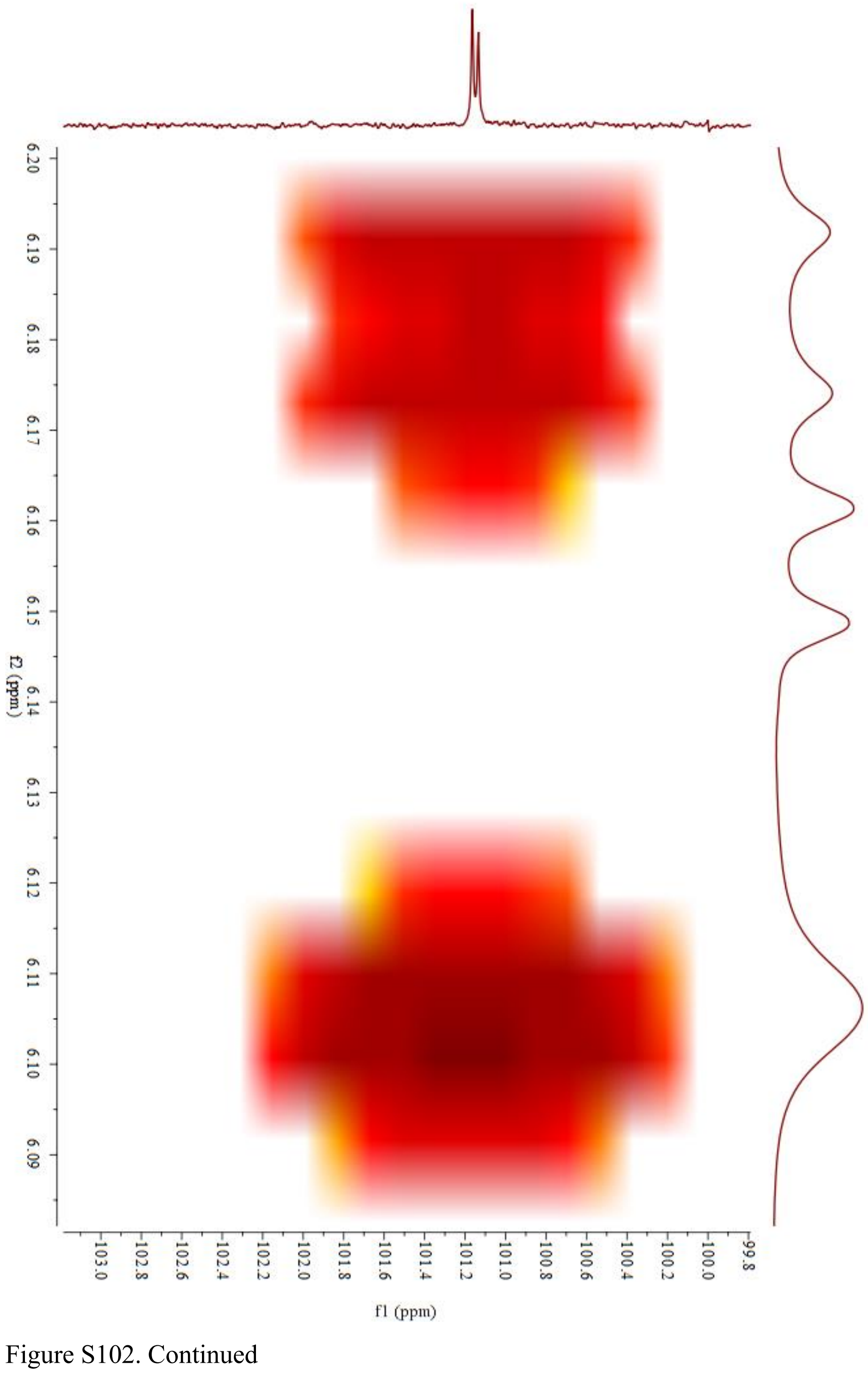

Figure S102. Continued

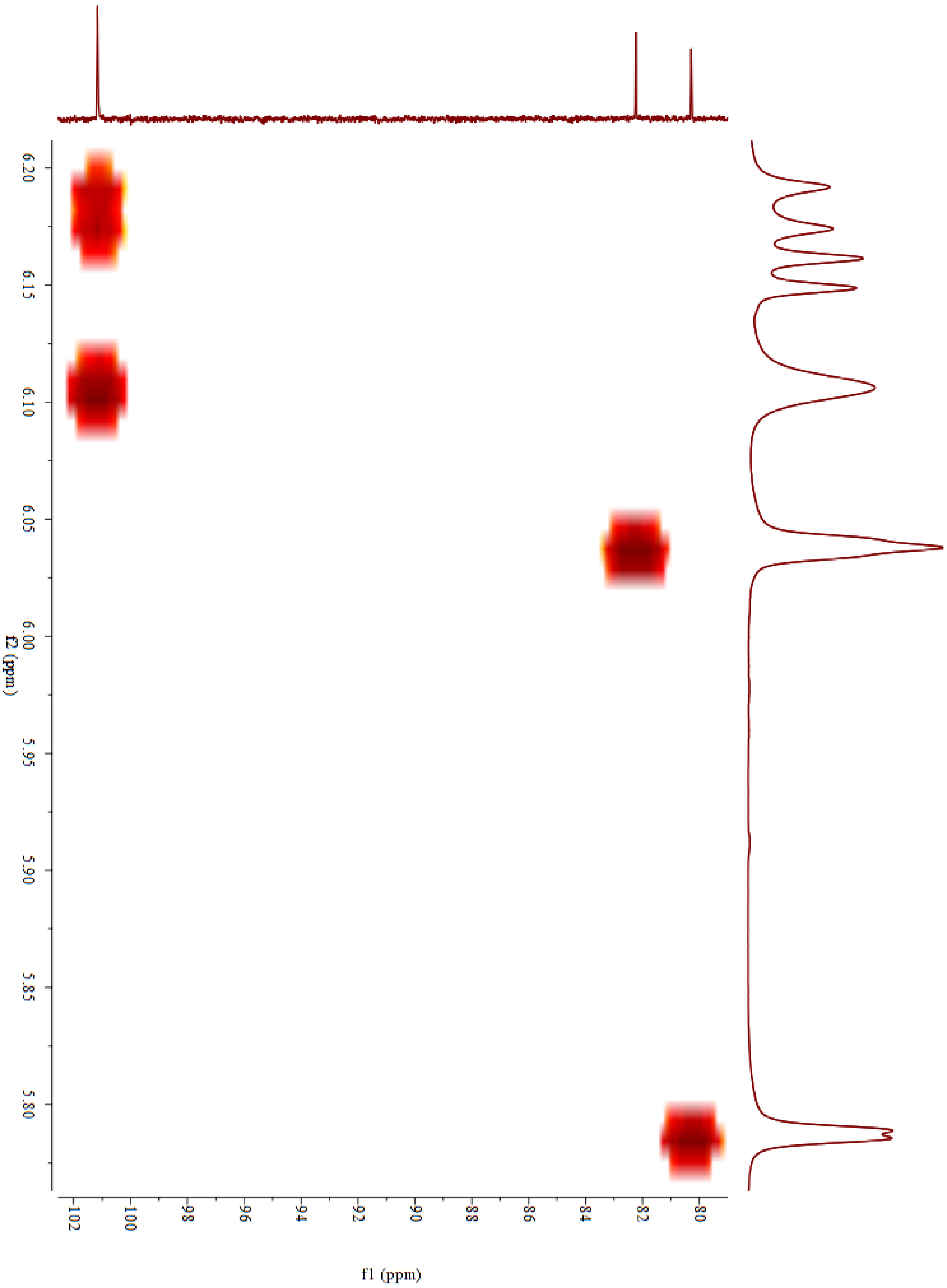

Figure S102. Continued

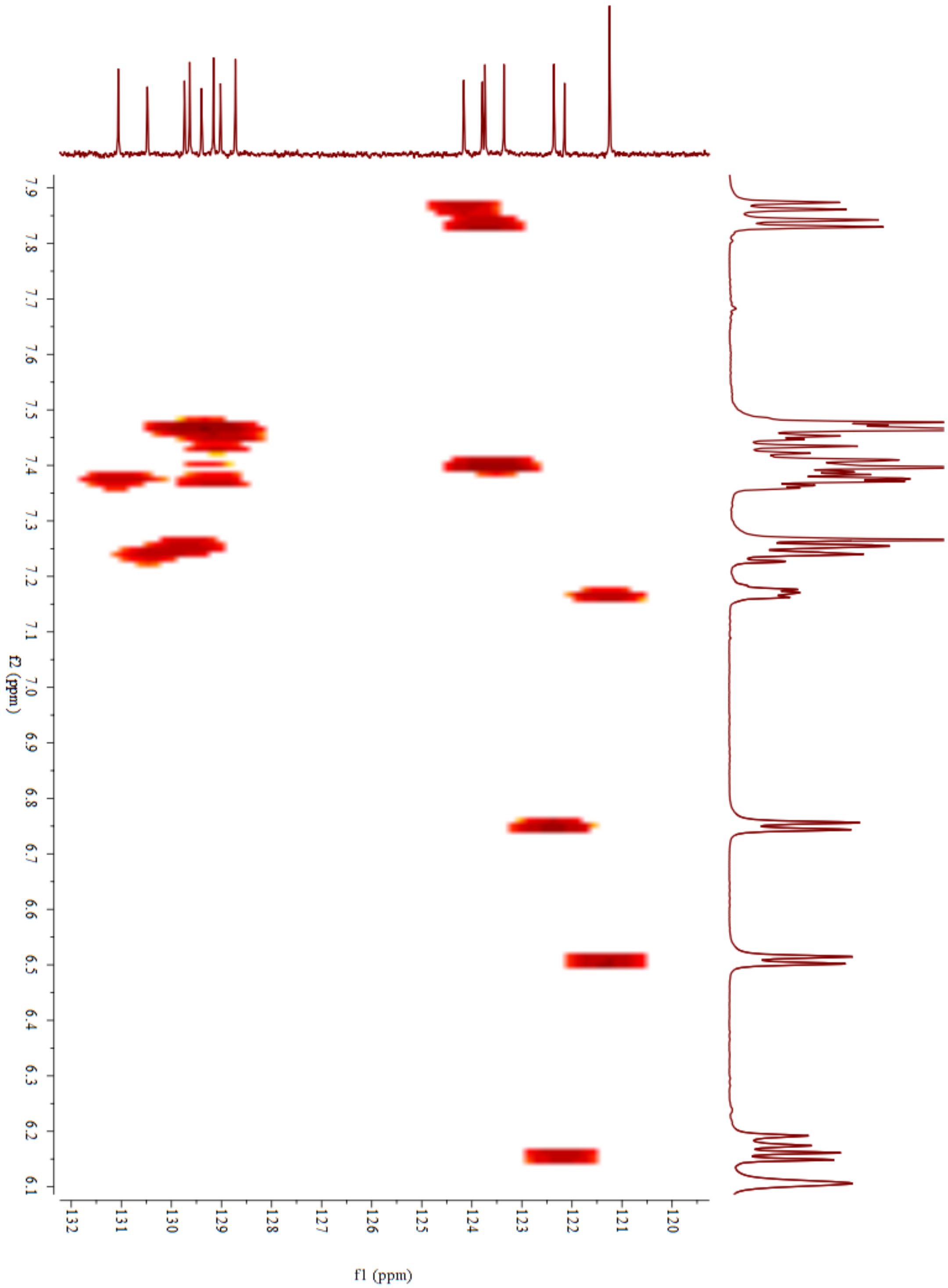

Figure S102. Continued

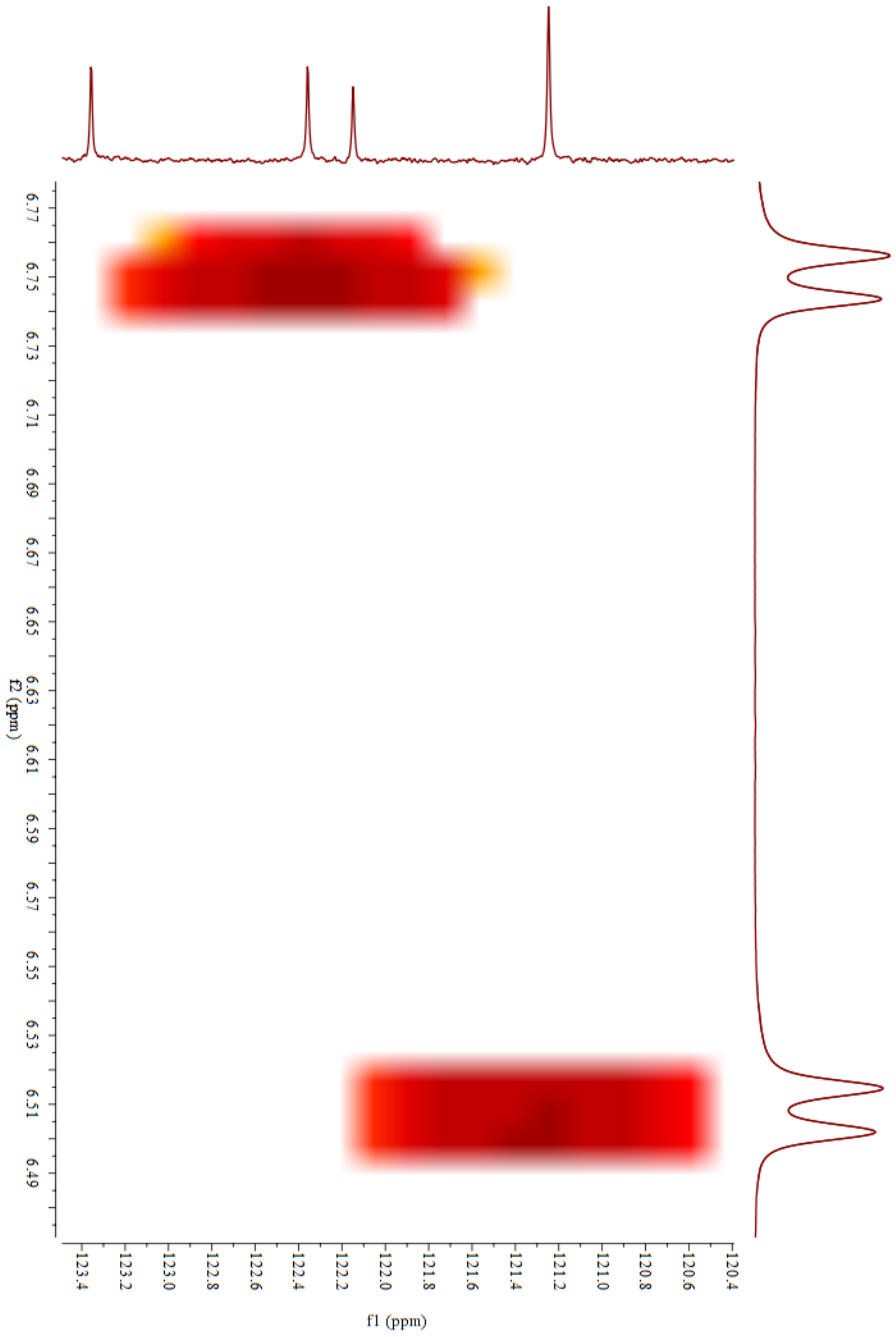

Figure S102. Continued

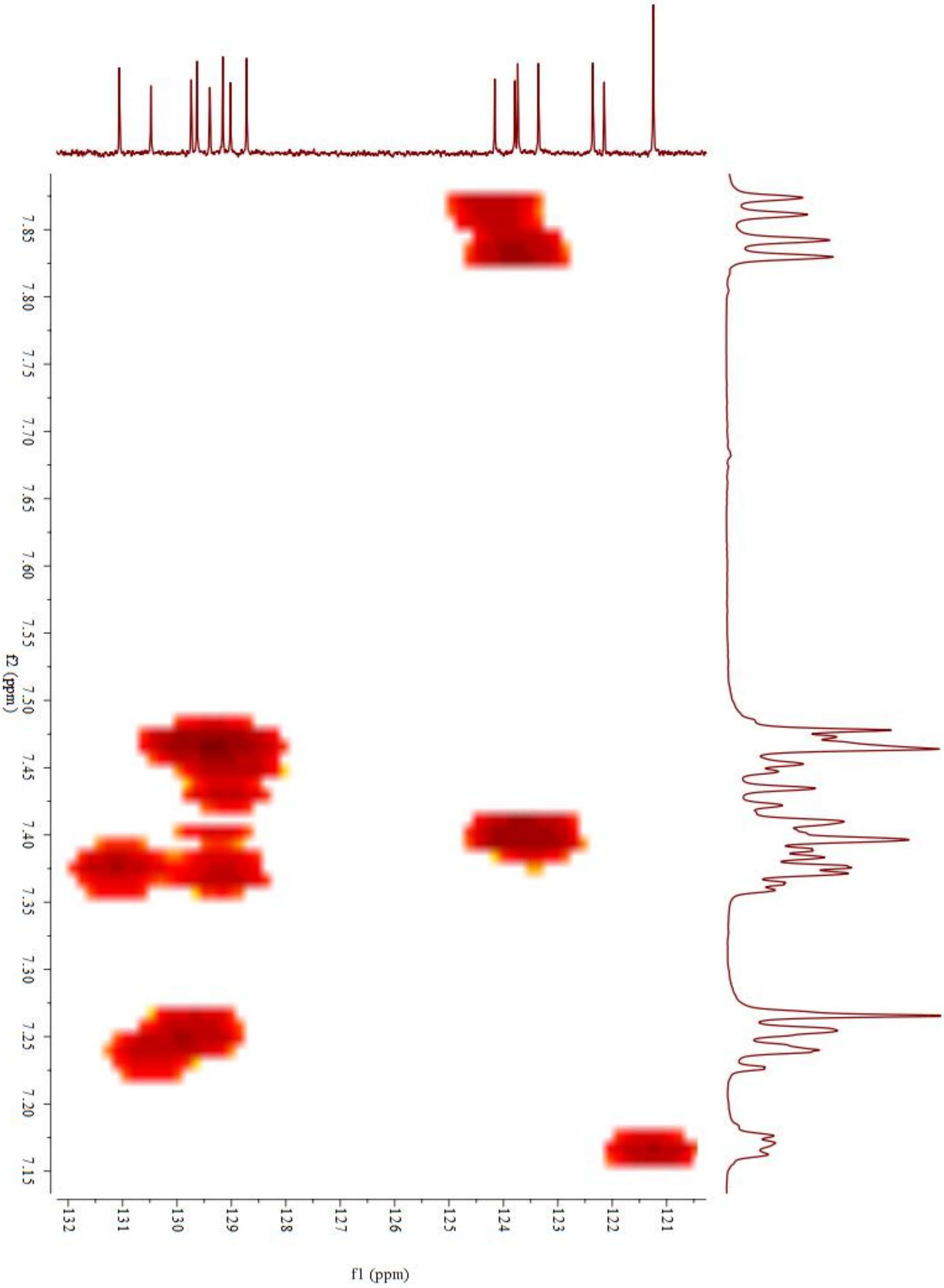

Figure S102. Continued

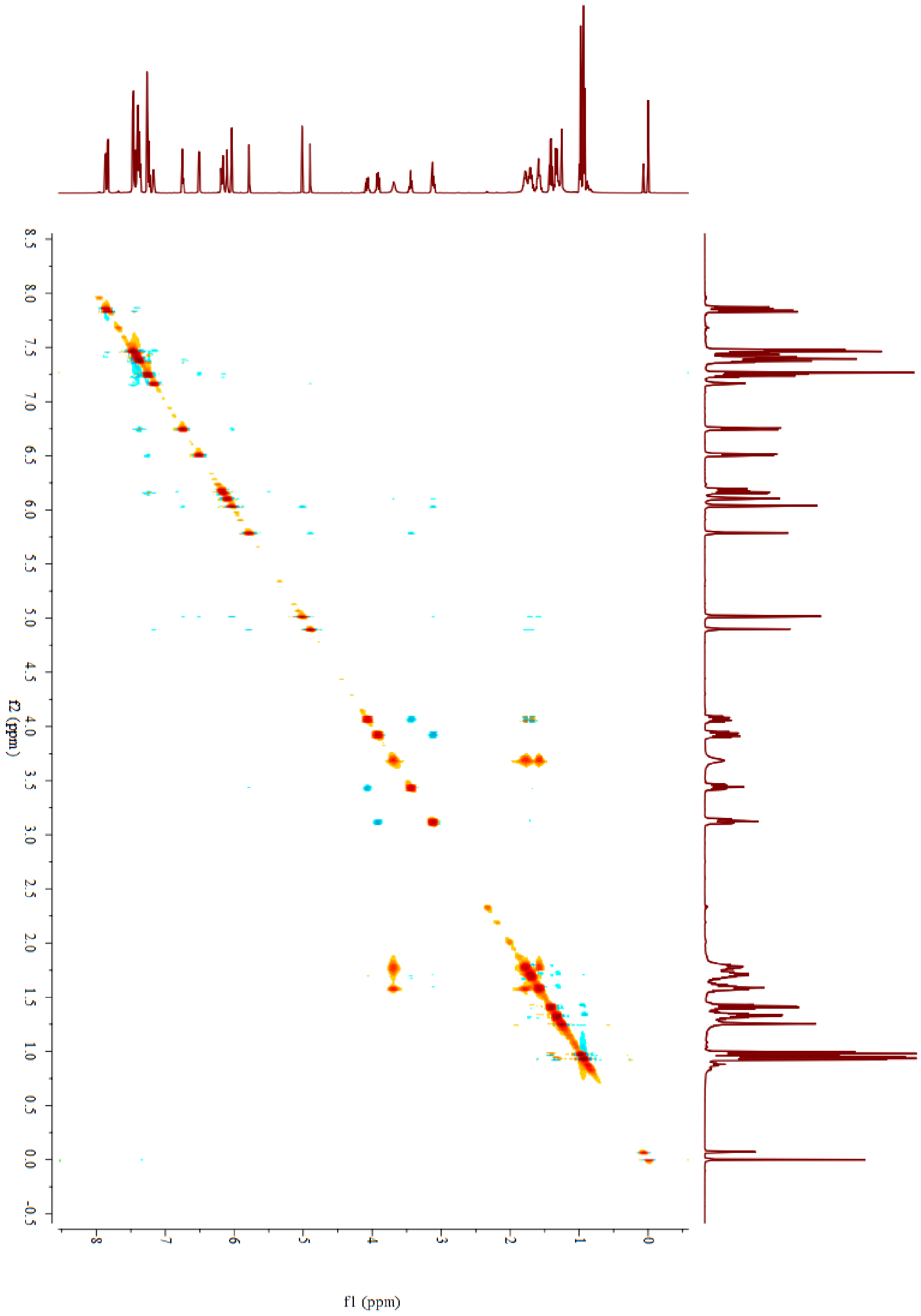

Figure S103. NOSEY of compound **8**

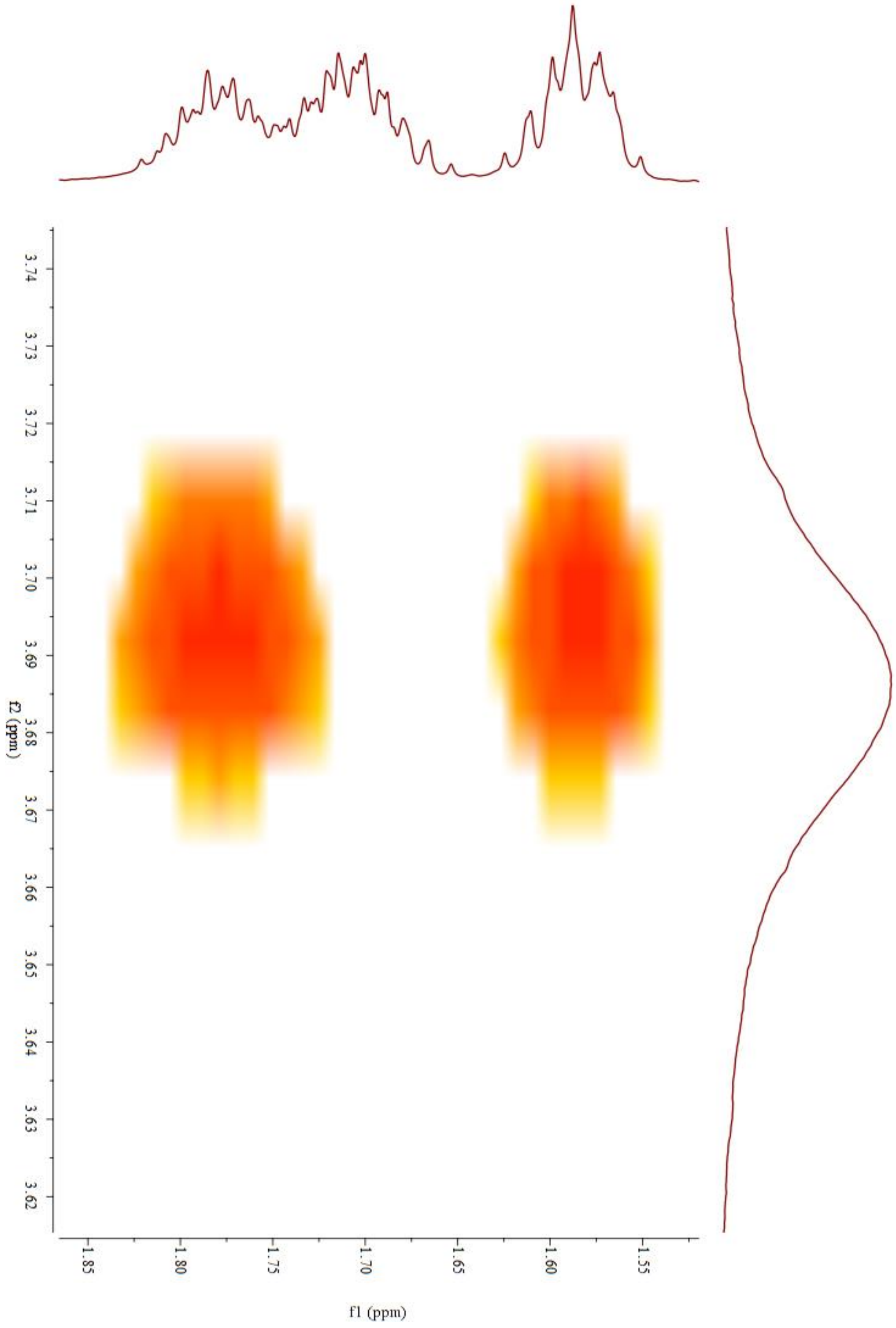

Figure S103. Continued

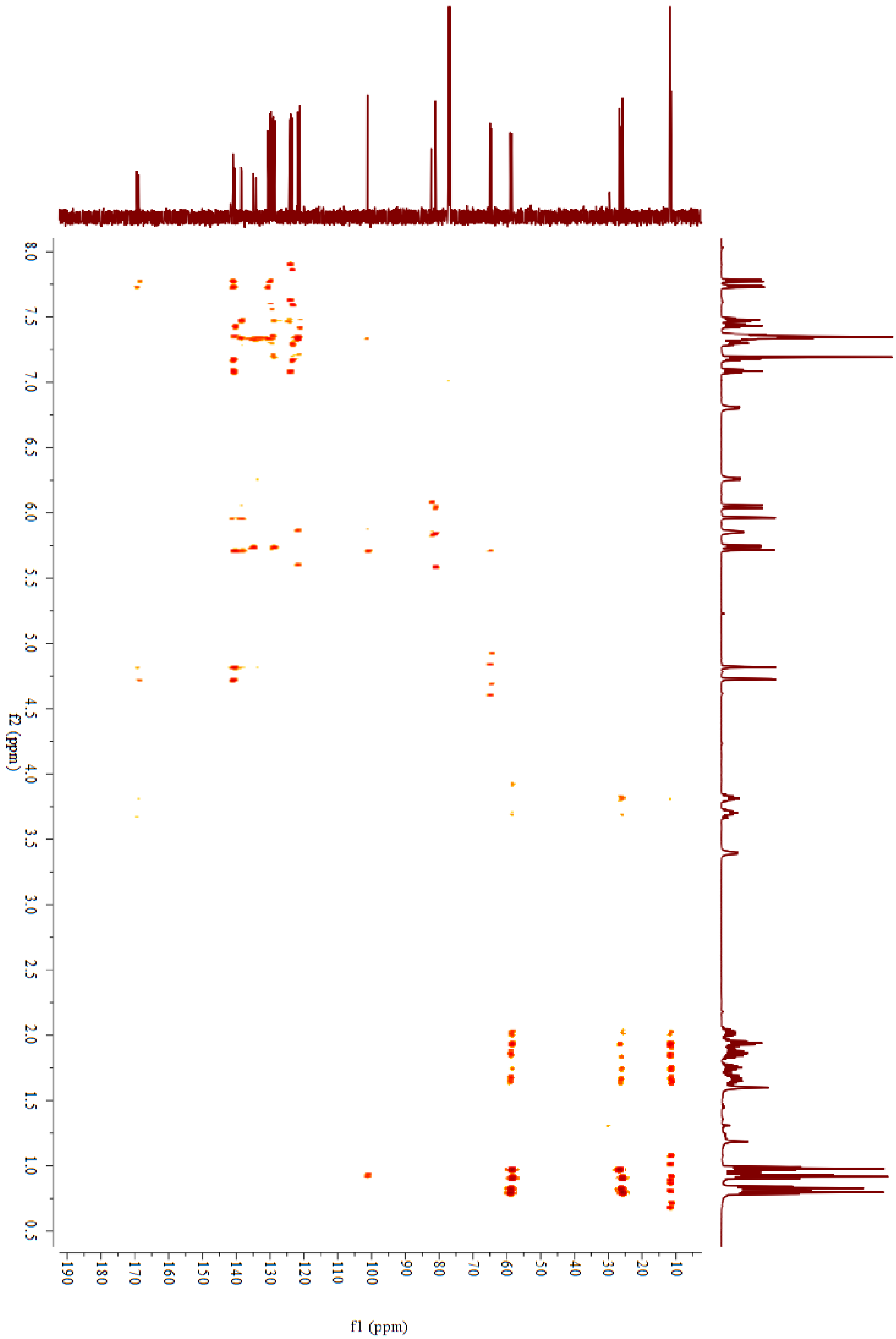

Figure S104. HMBC of compound **13.**

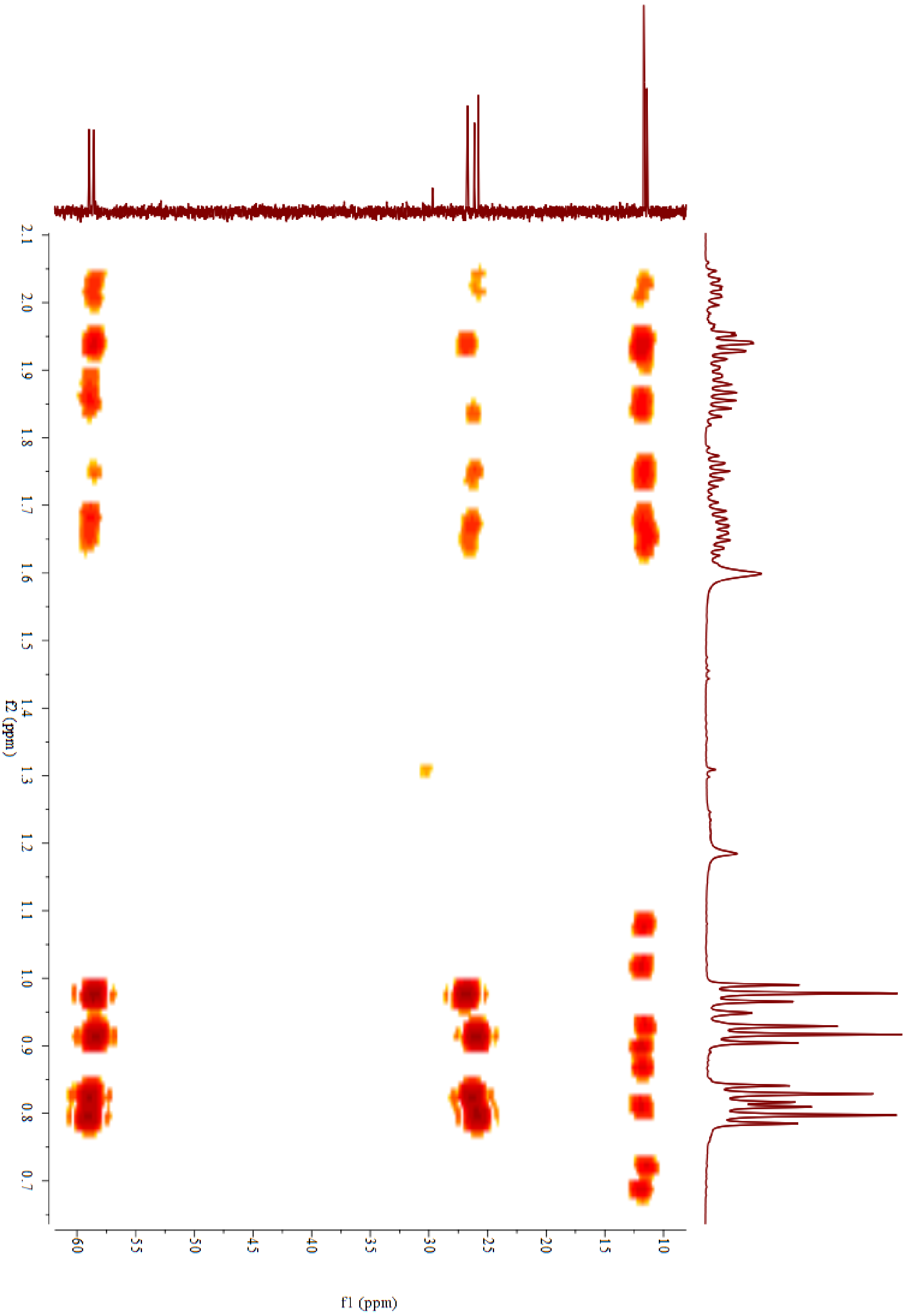

Figure S104. continued

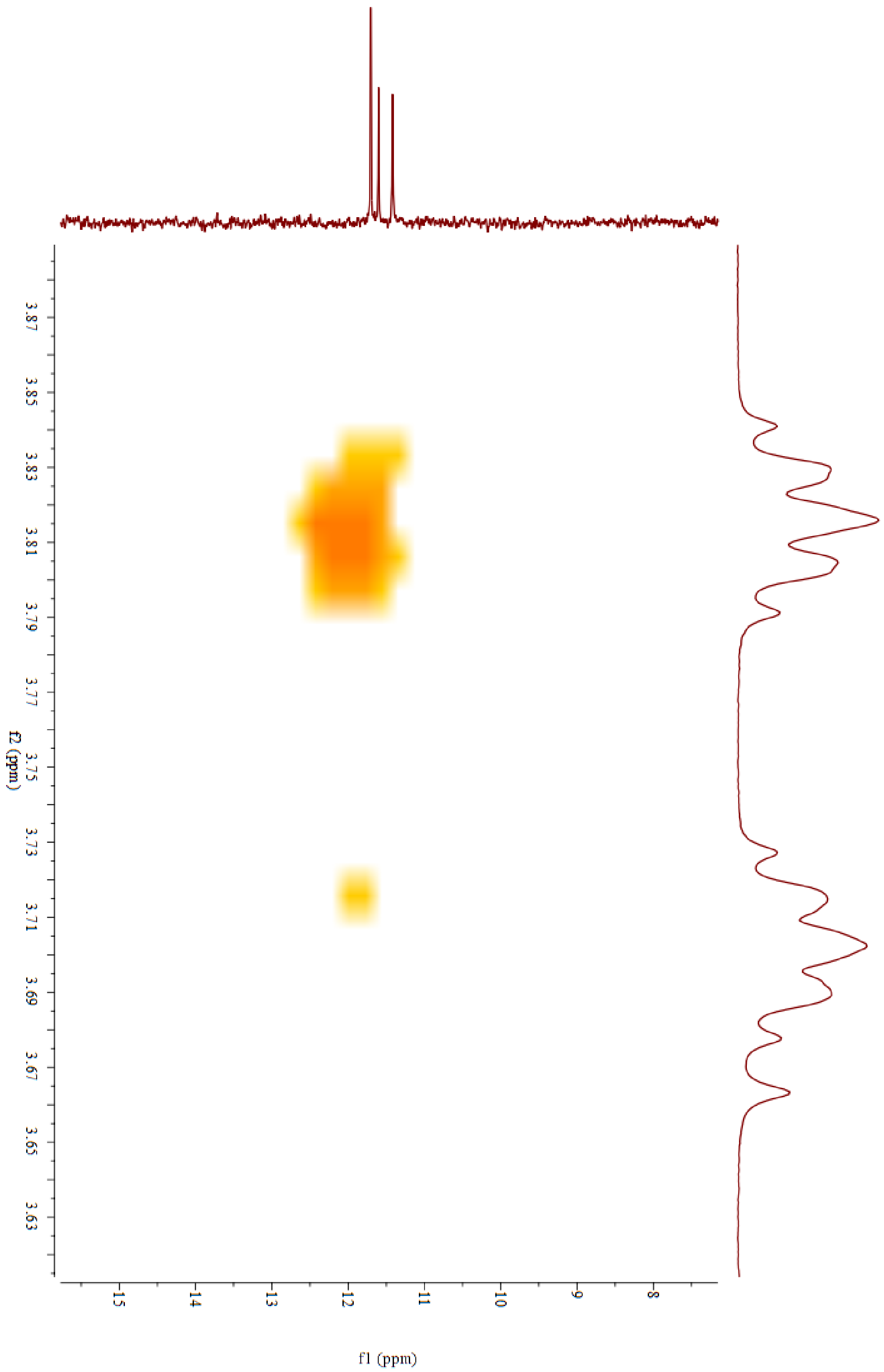

Figure S104. continued

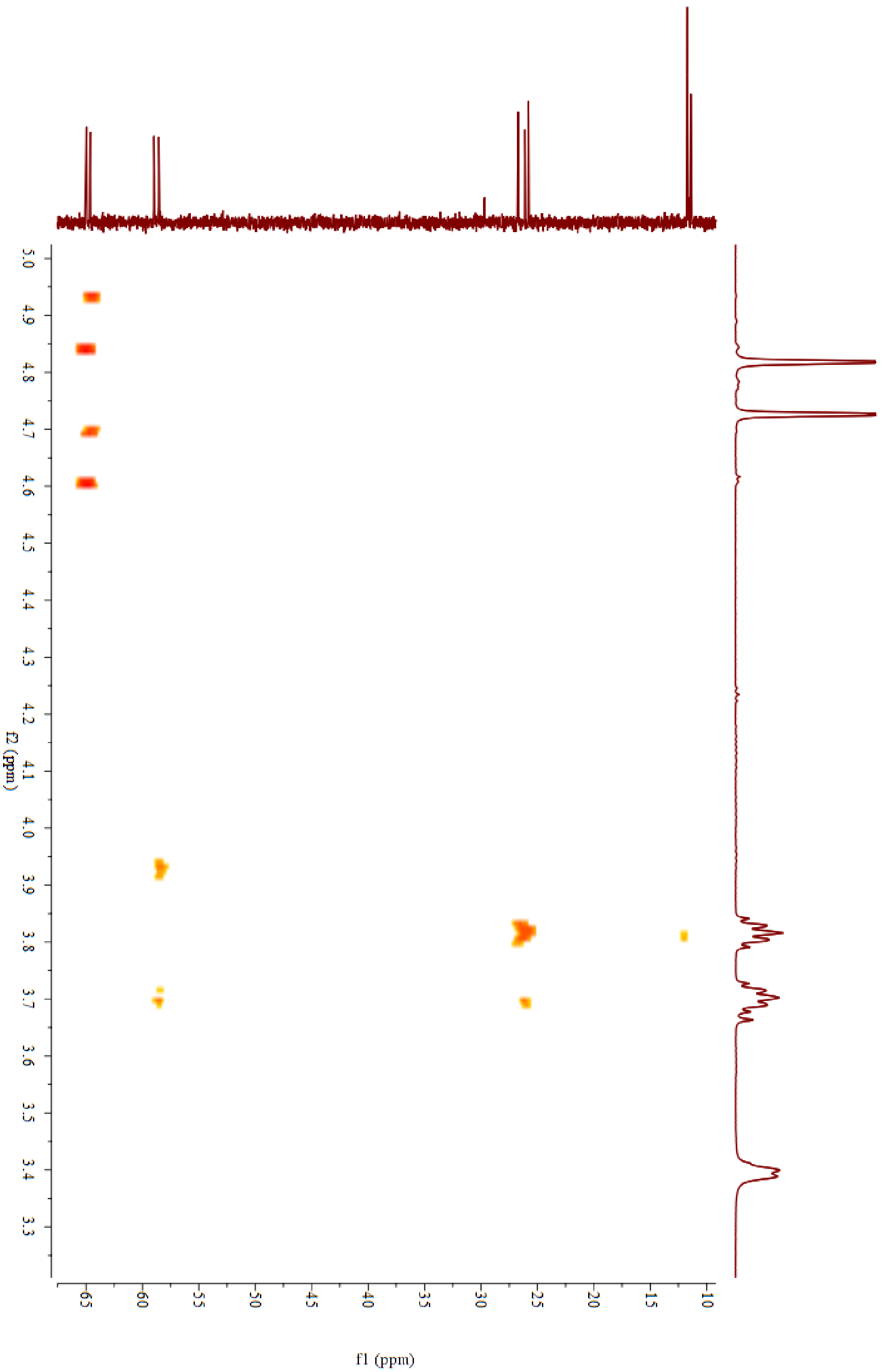

Figure S104. continued

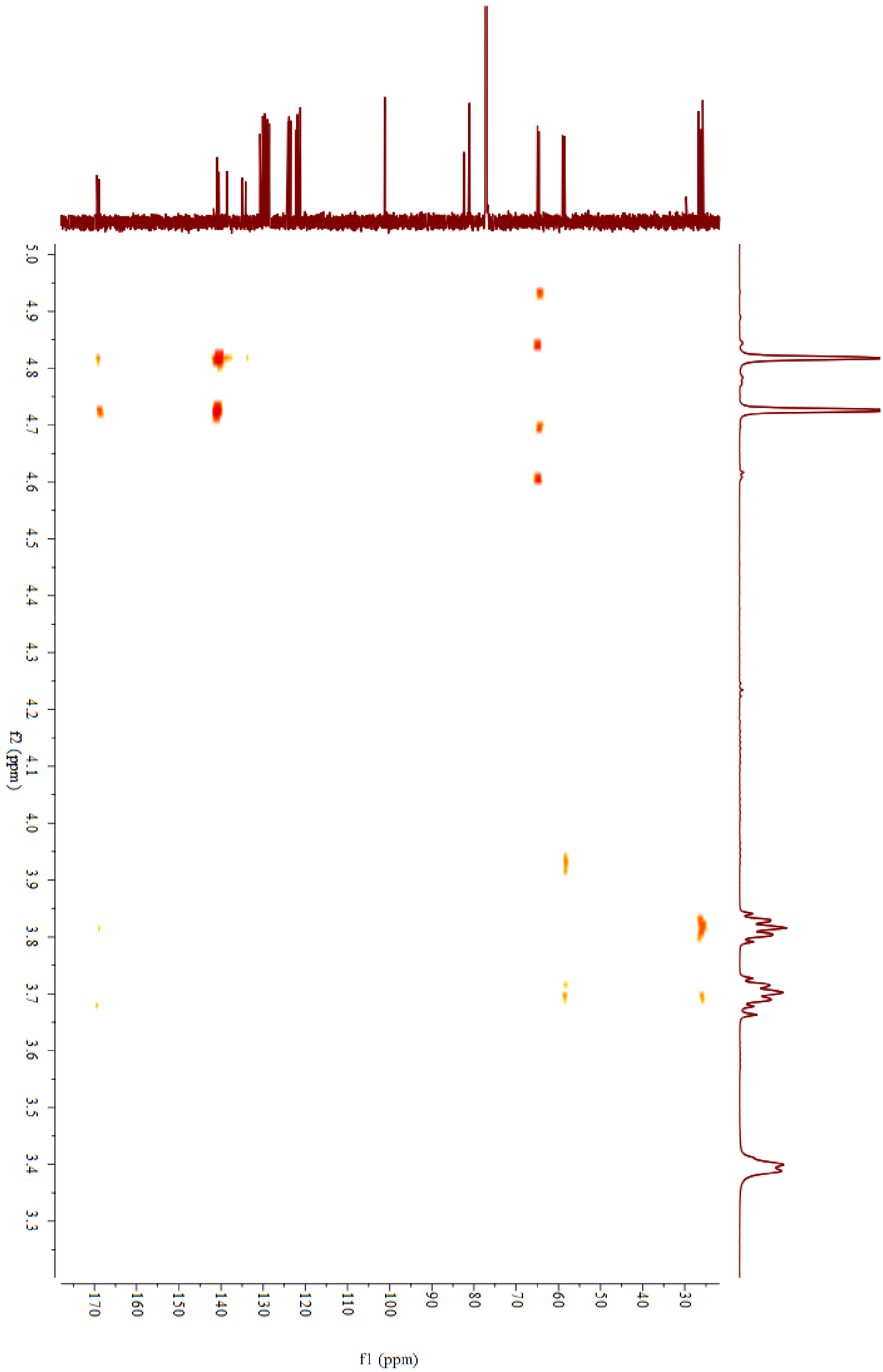

Figure S104. continued

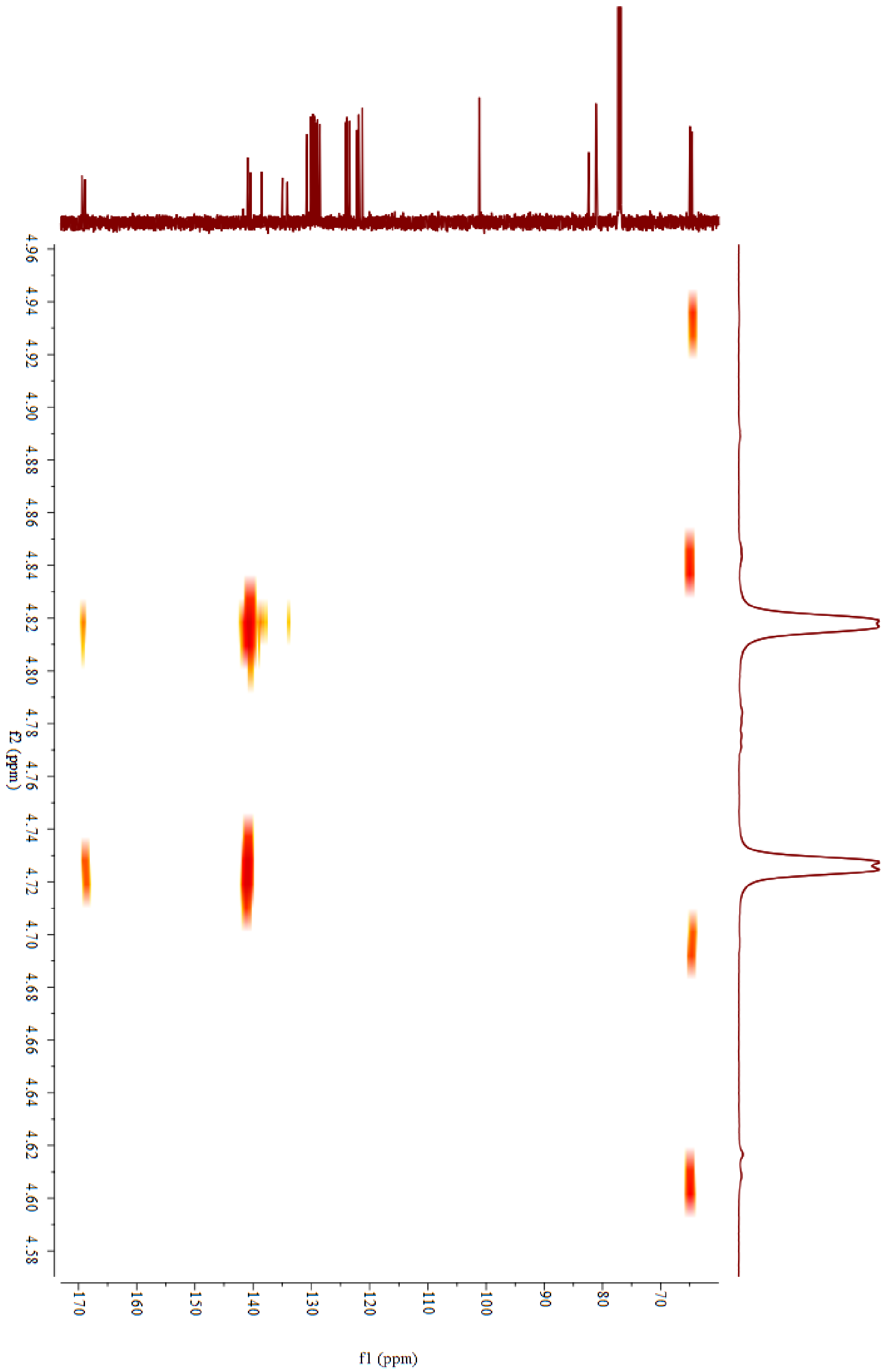

Figure S104. continued

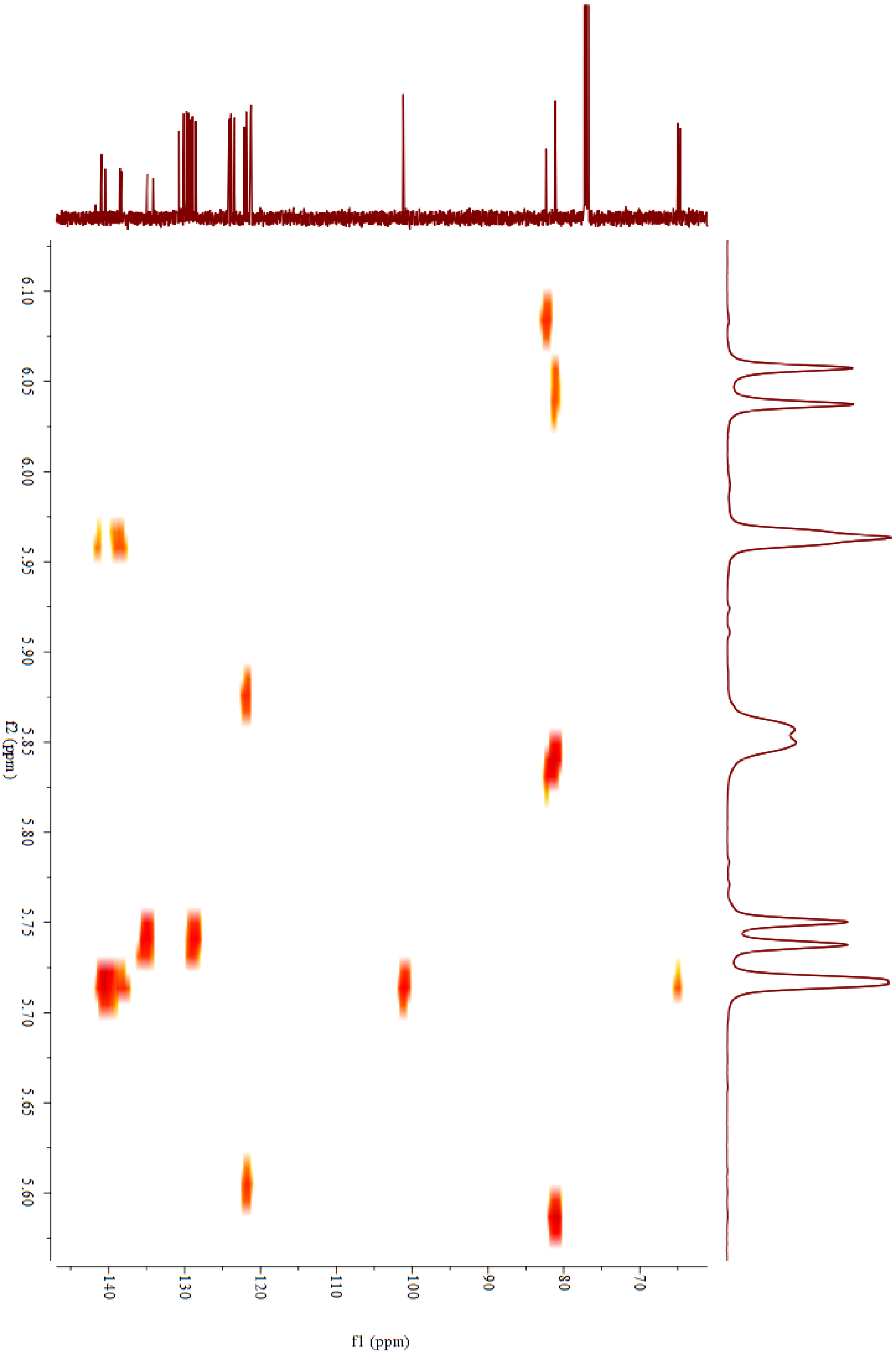

Figure S104. continued

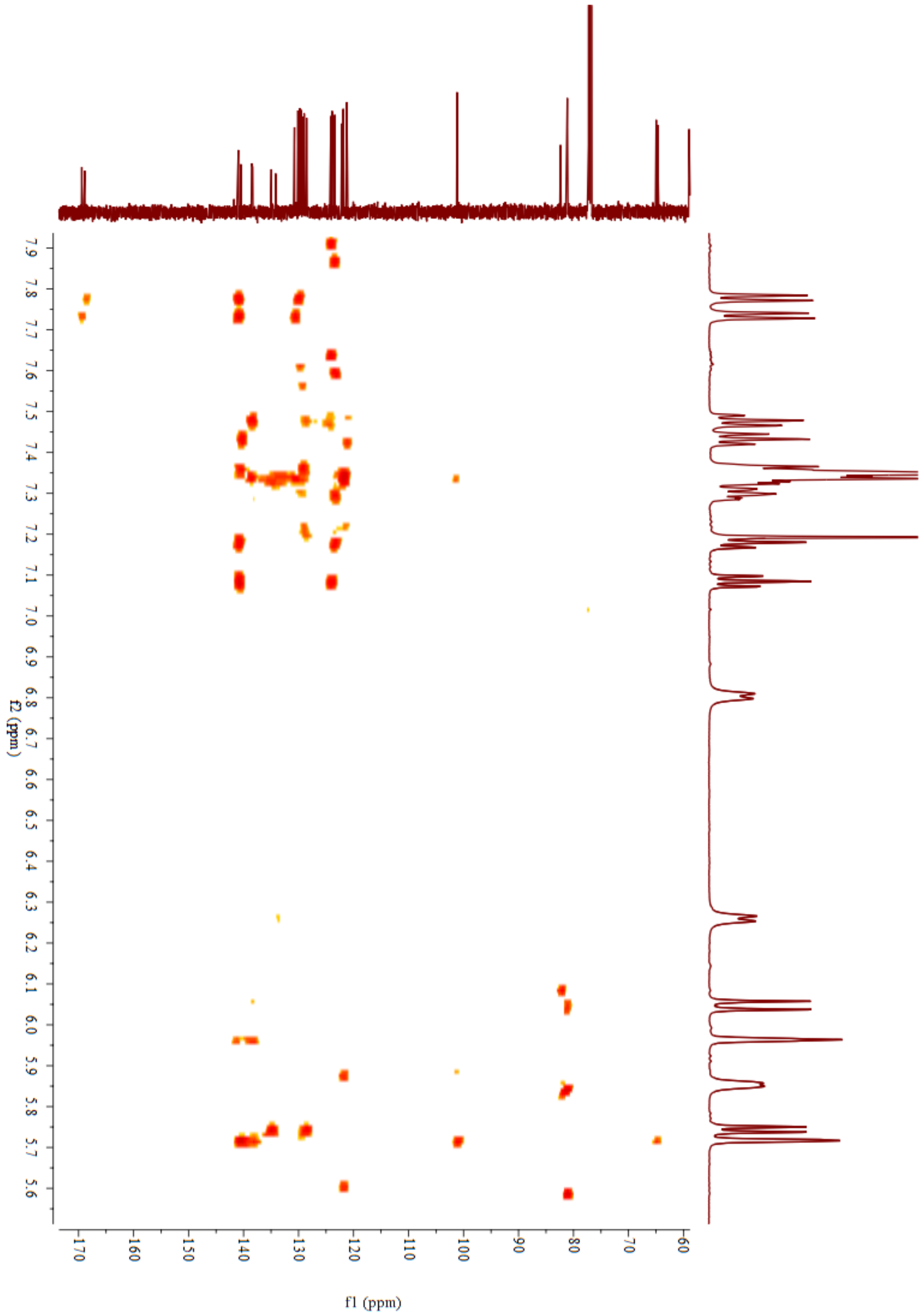

Figure S104. continued

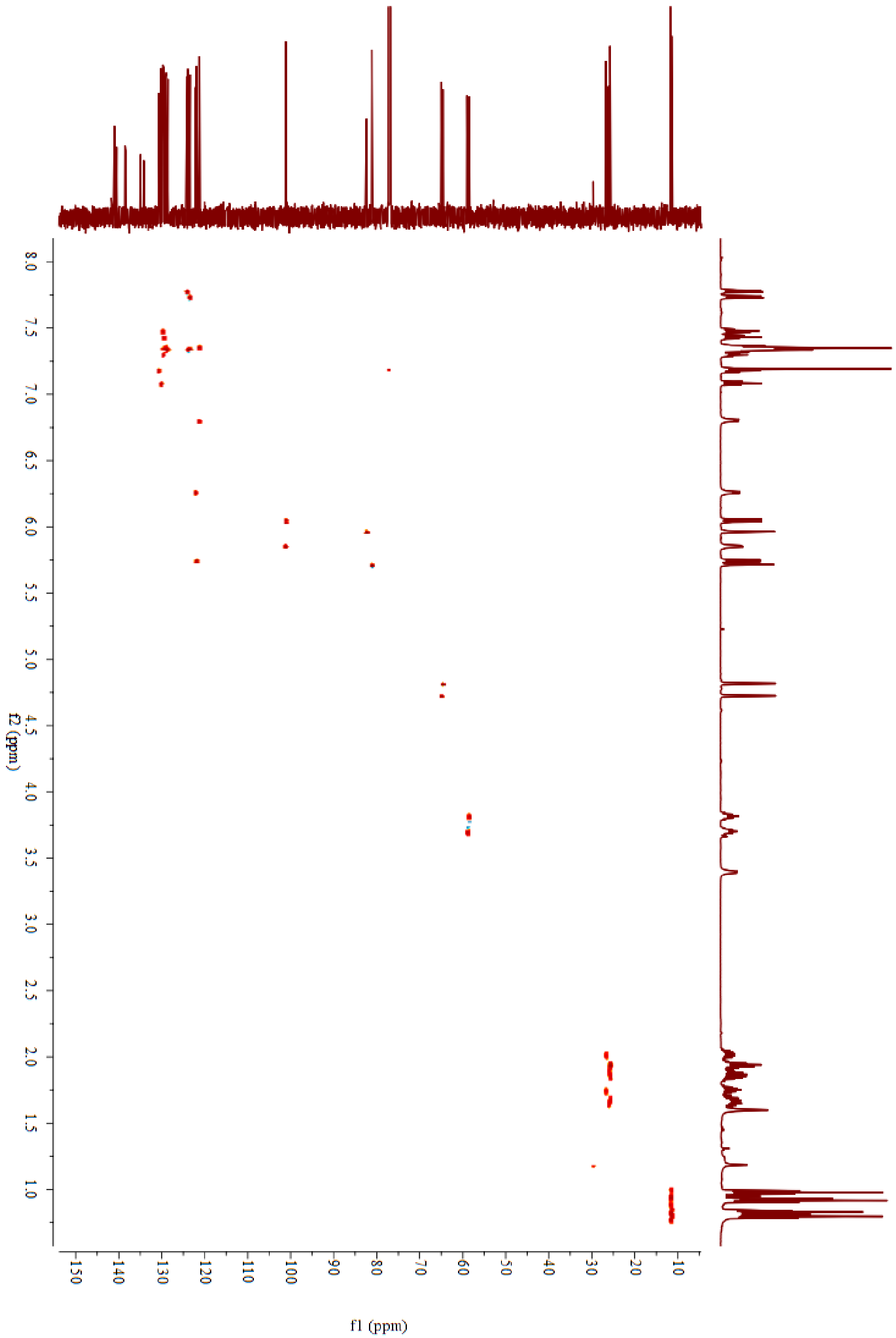

Figure S105. HSQC of compound **13.**

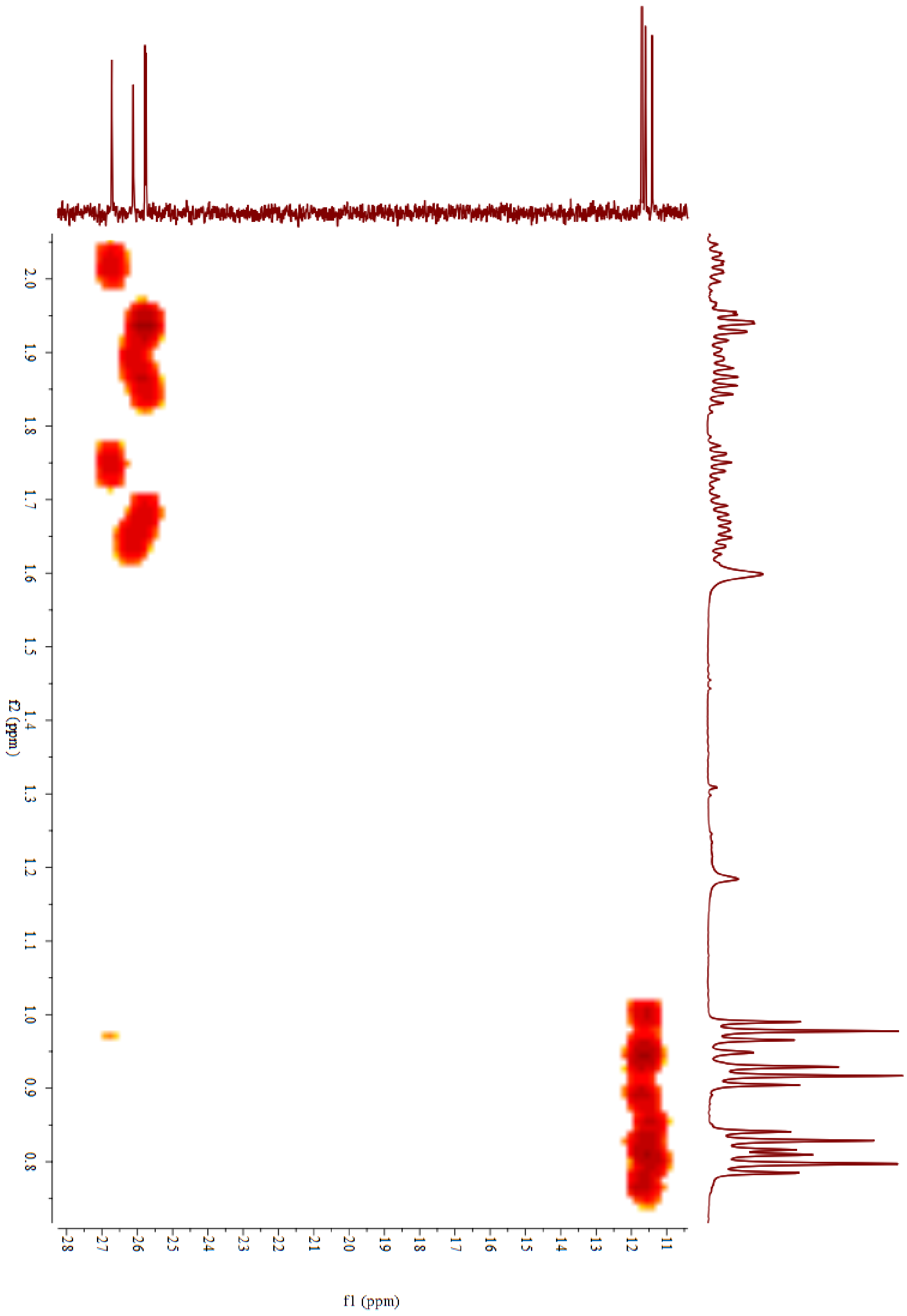

Figure S105. Continued

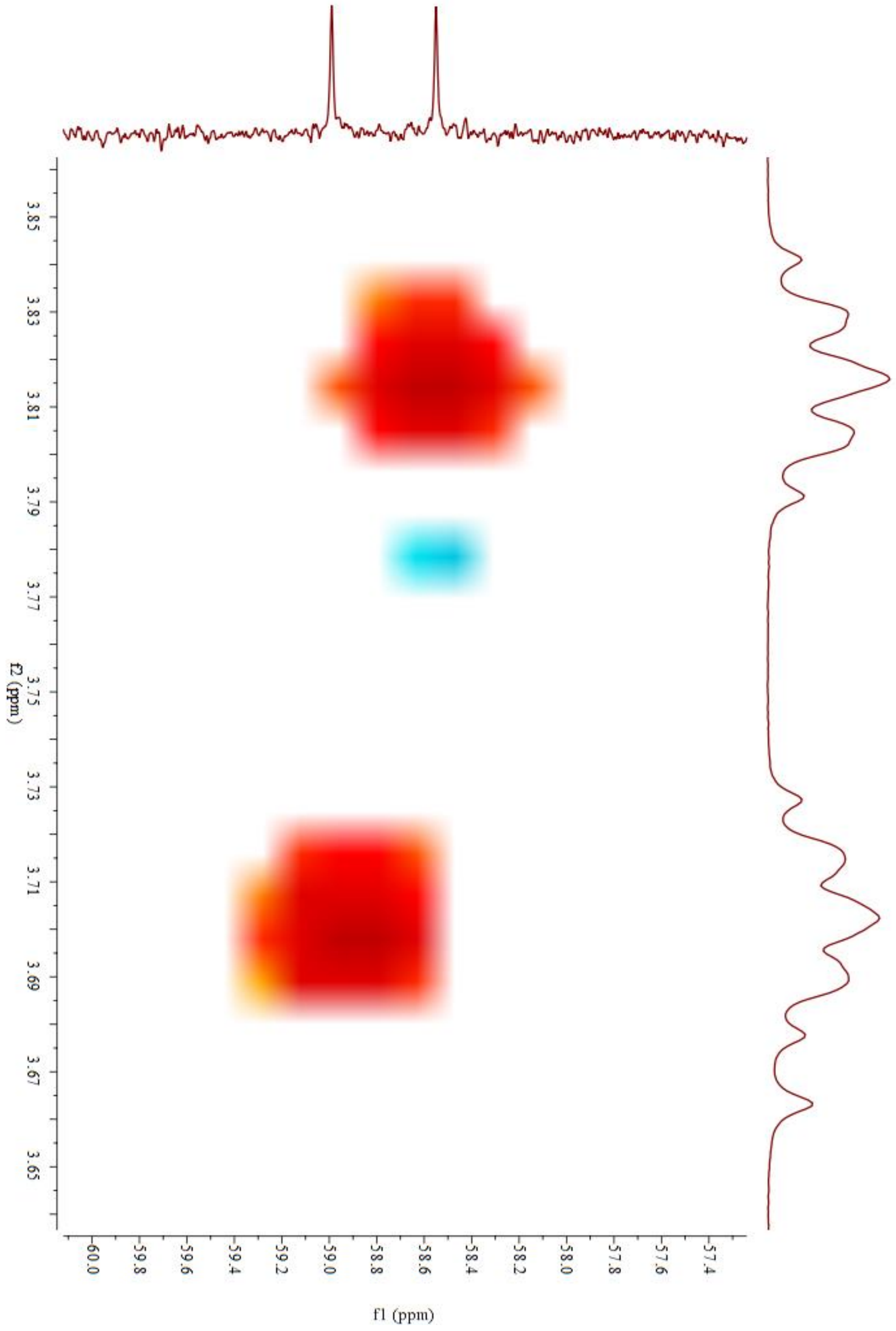

Figure S105. Continued

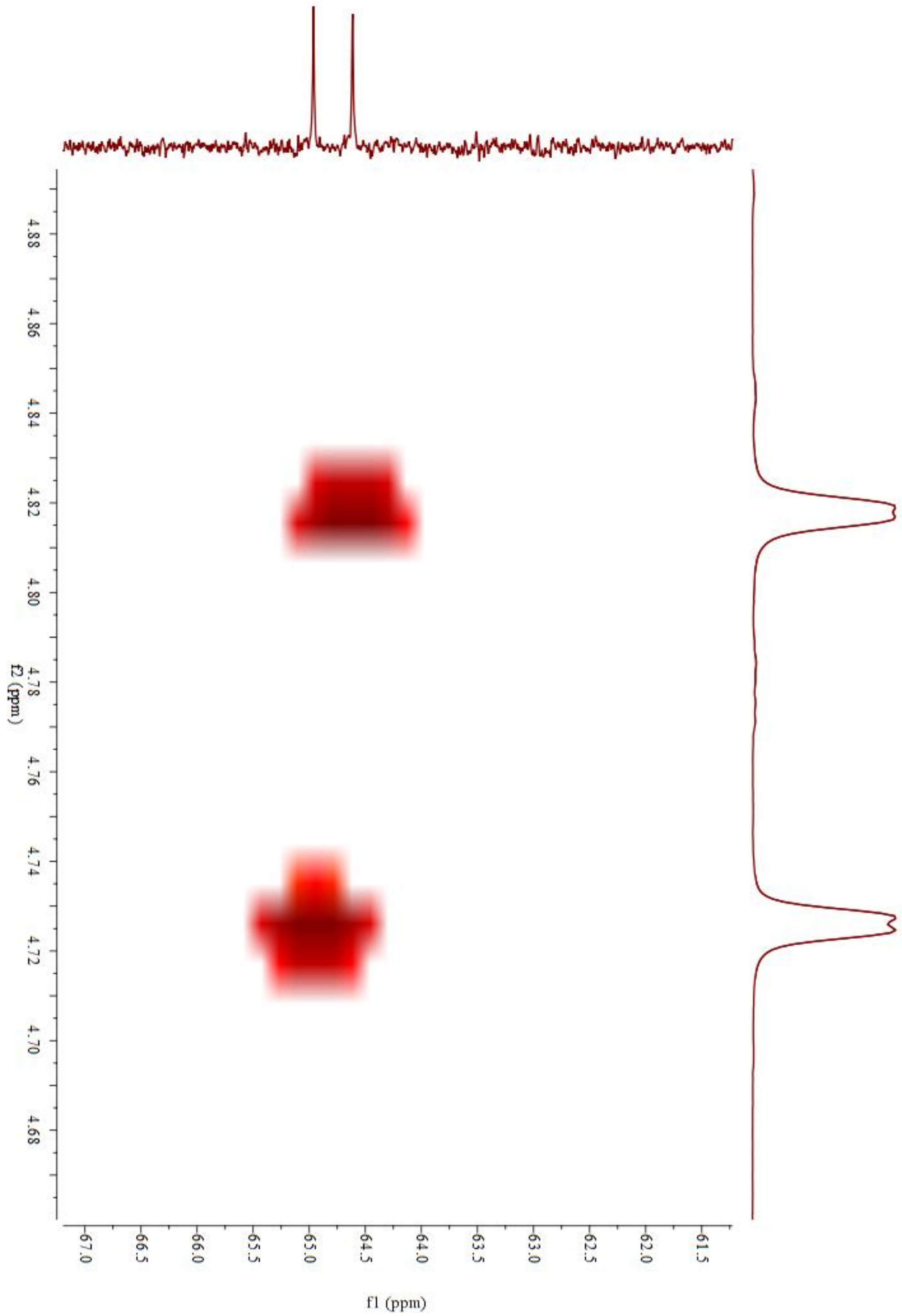

Figure S105. Continued

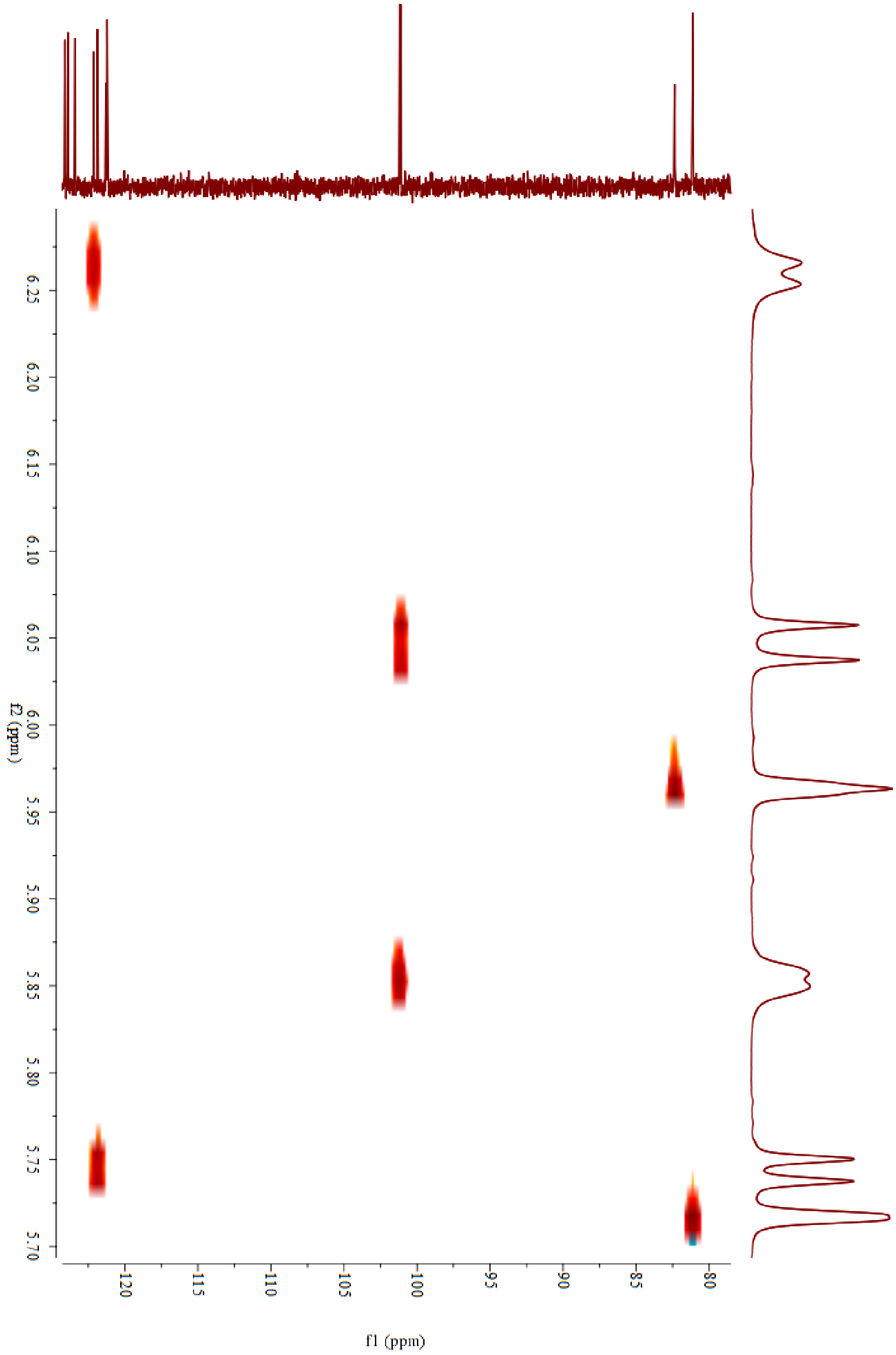

Figure S105. Continued

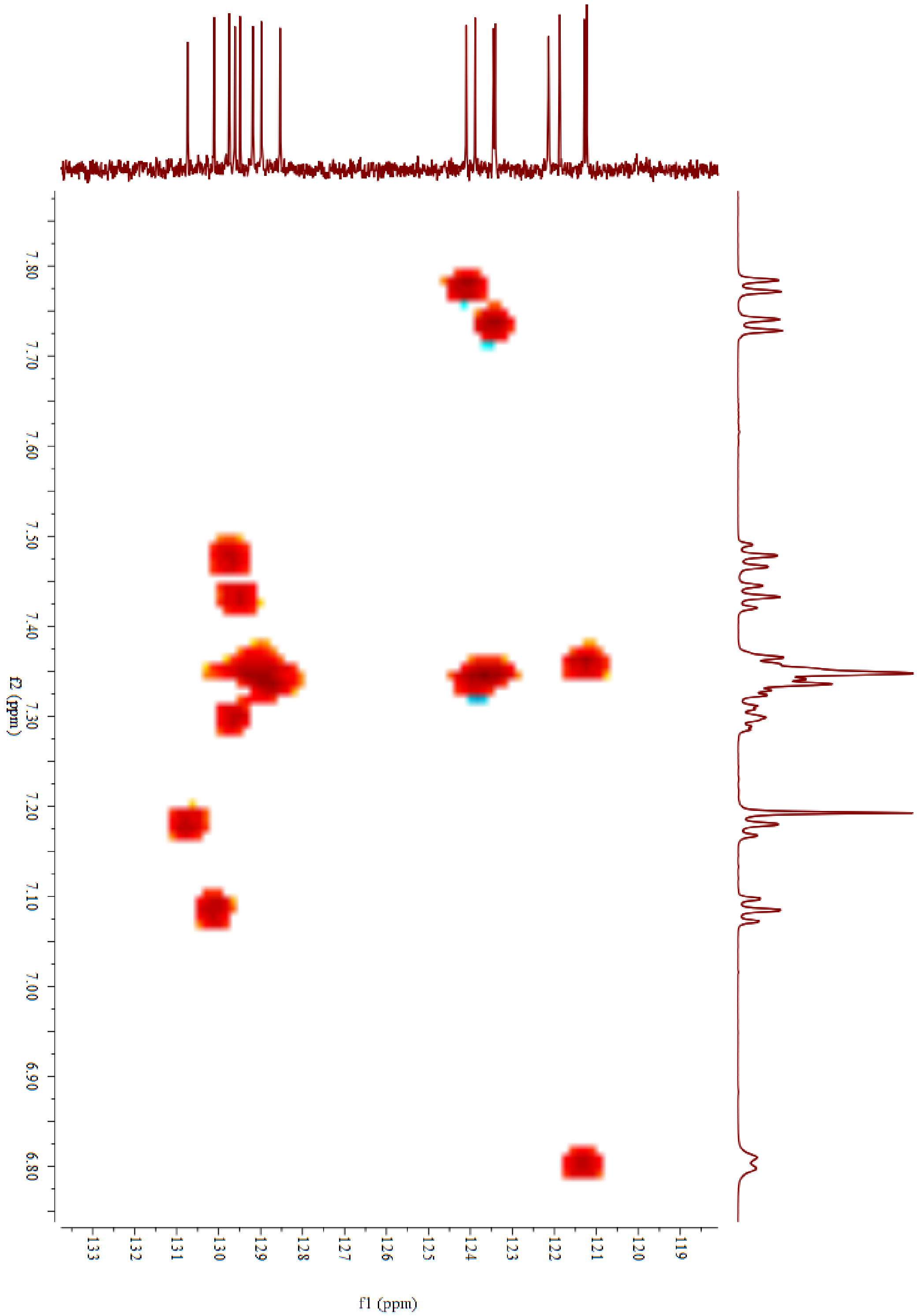

Figure S105. Continued

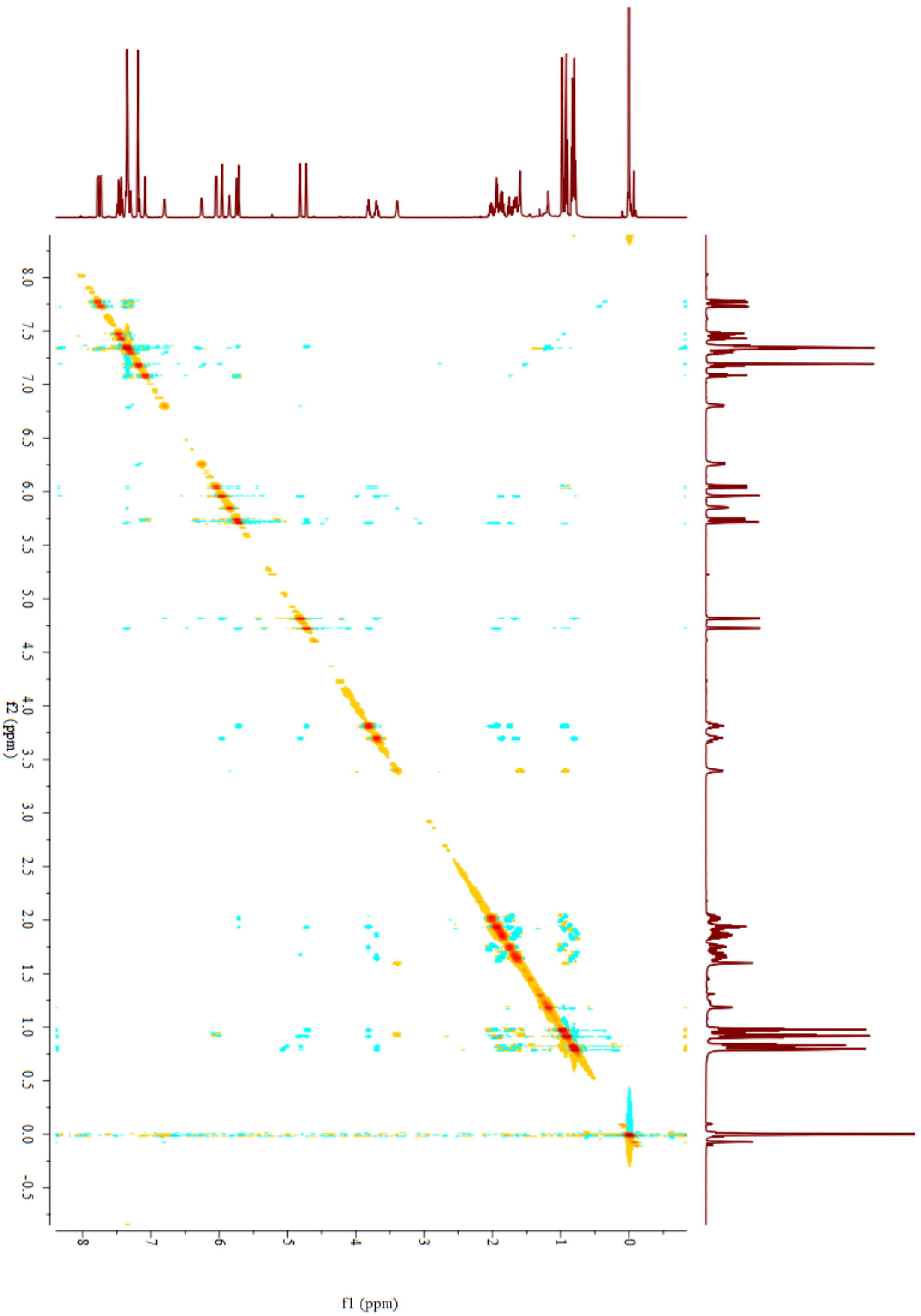

Figure S106. NOSEY of compound **13**

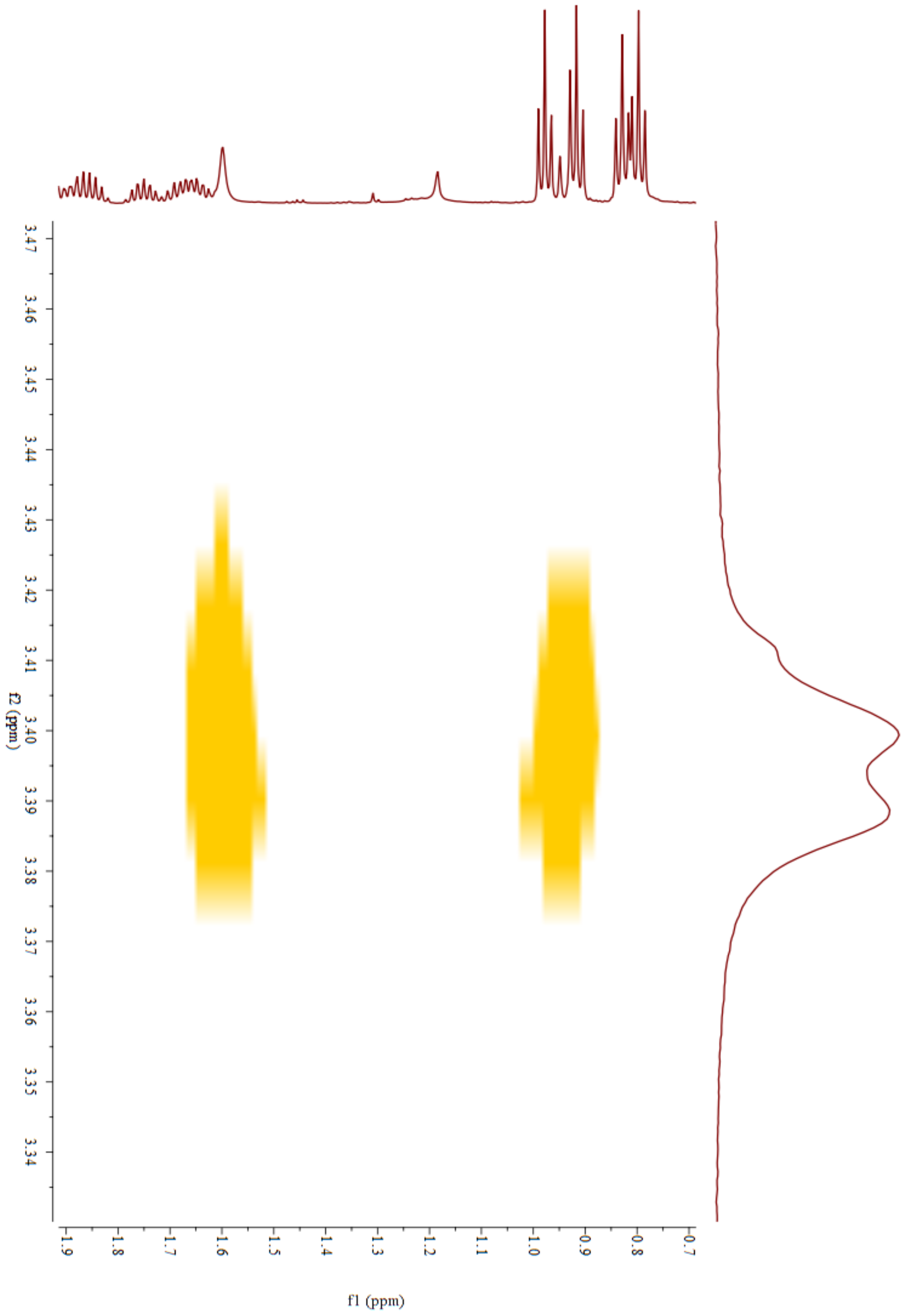

Figure S106. Continued

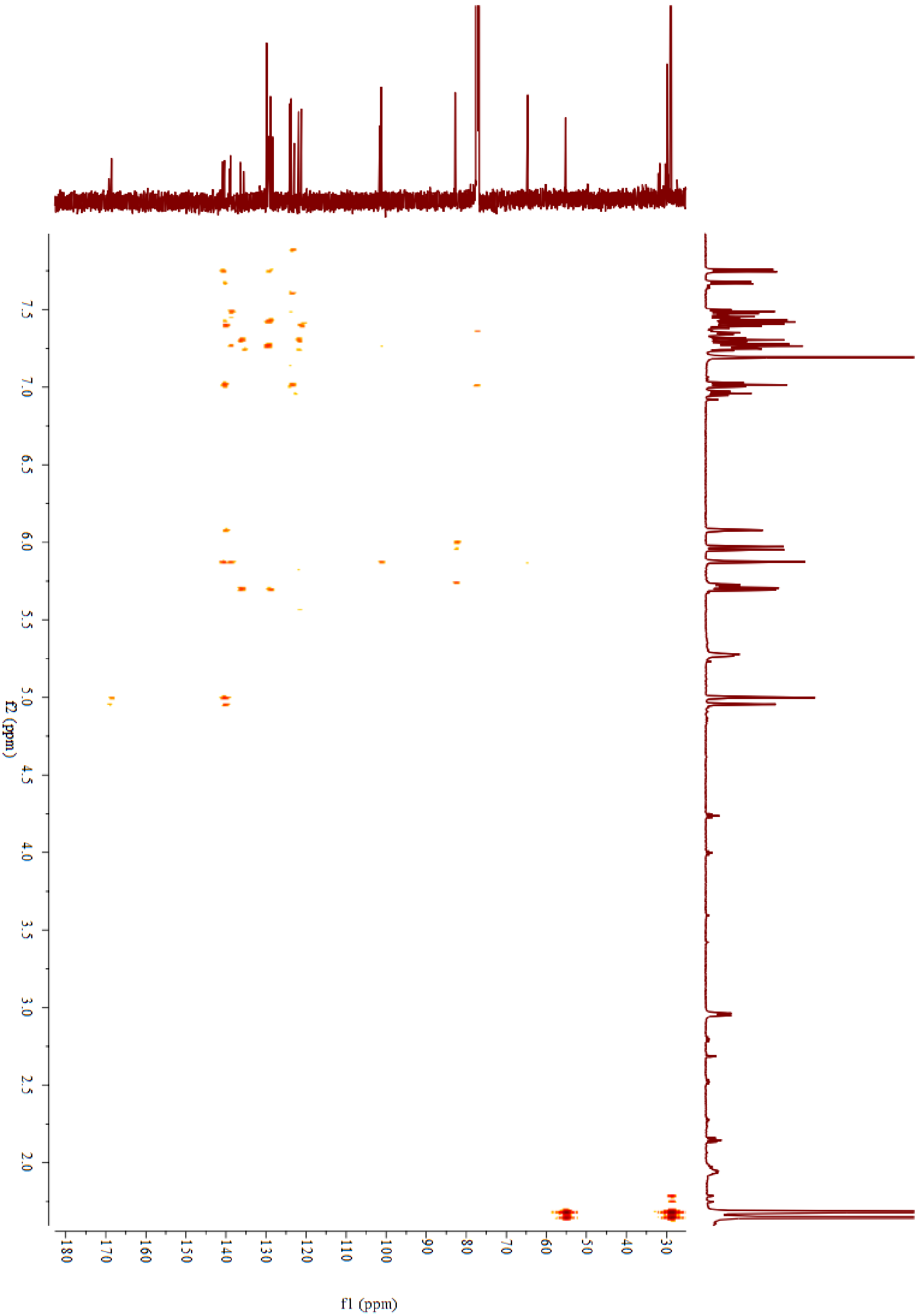

Figure S107. HMBC of compound **9.**

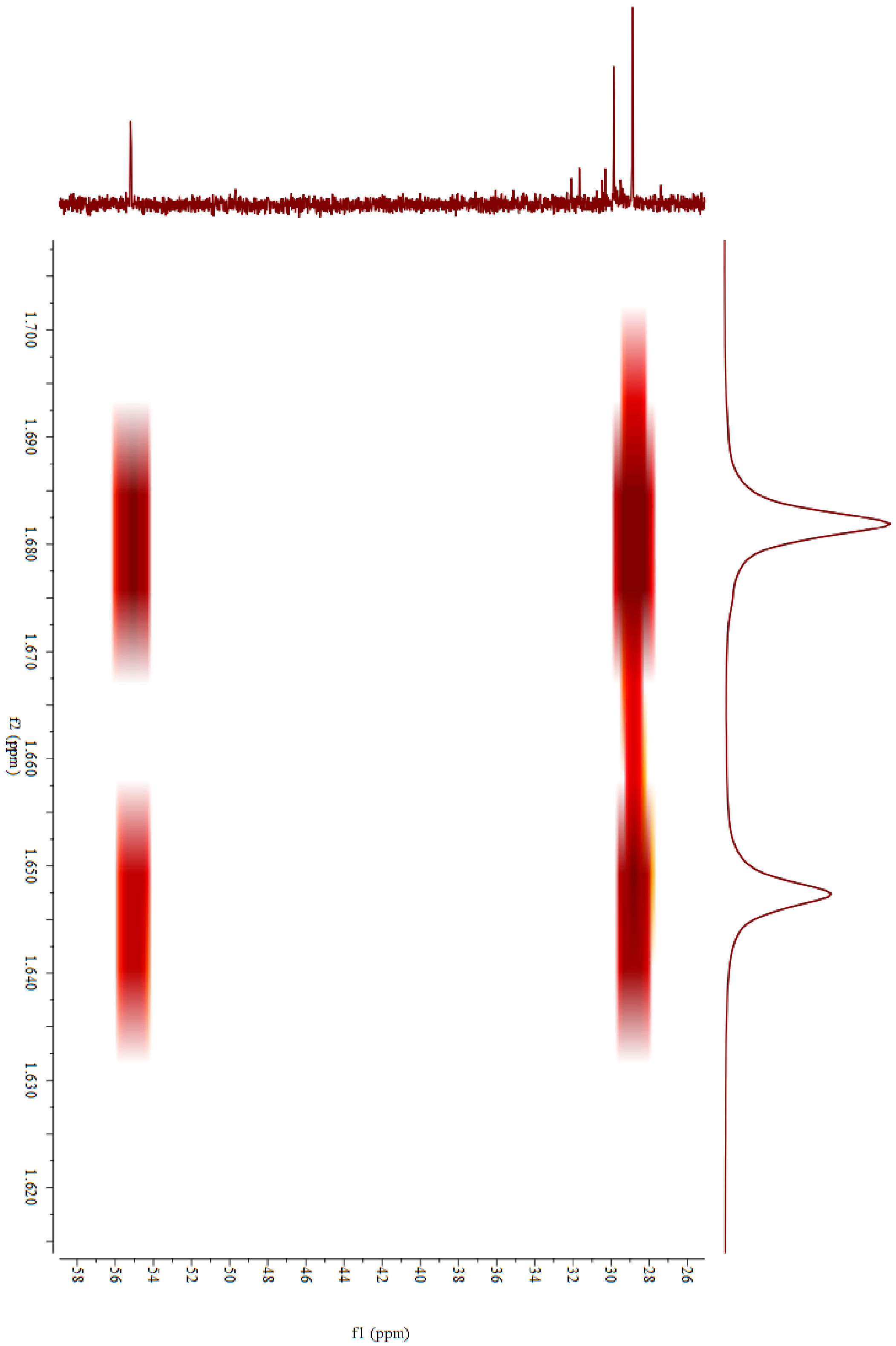

Figure S107. Continued

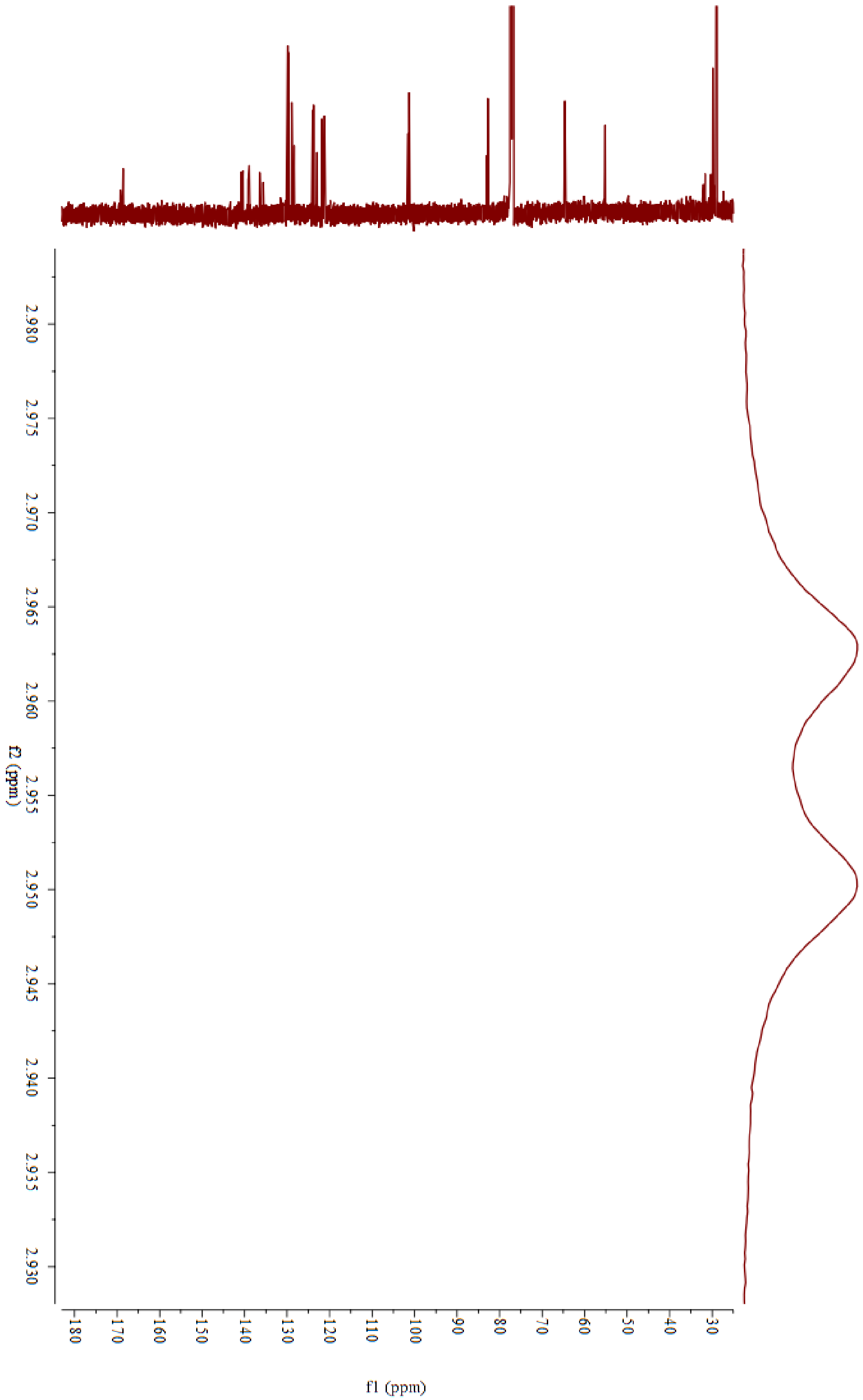

Figure S107. Continued

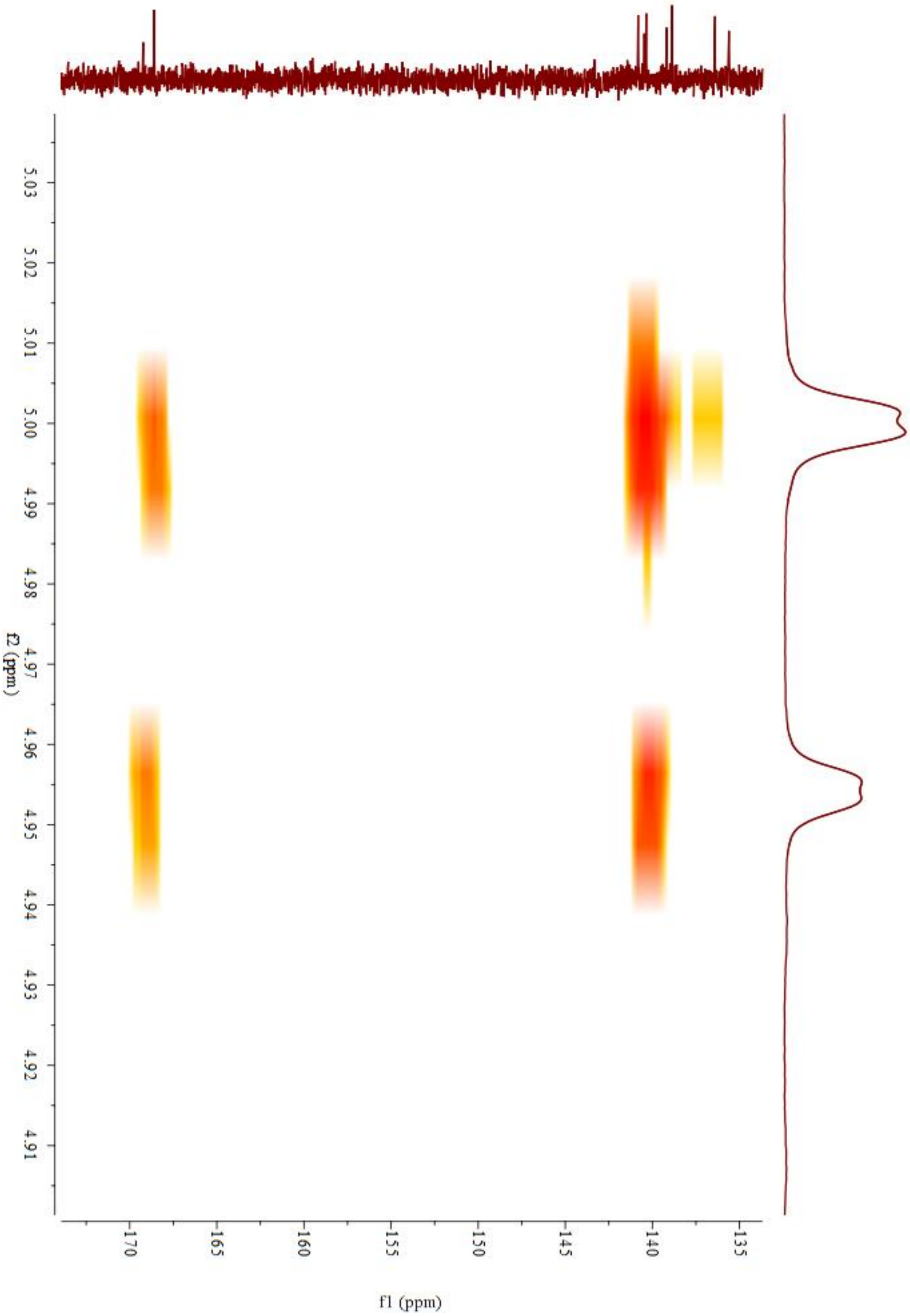

Figure S107. Continued

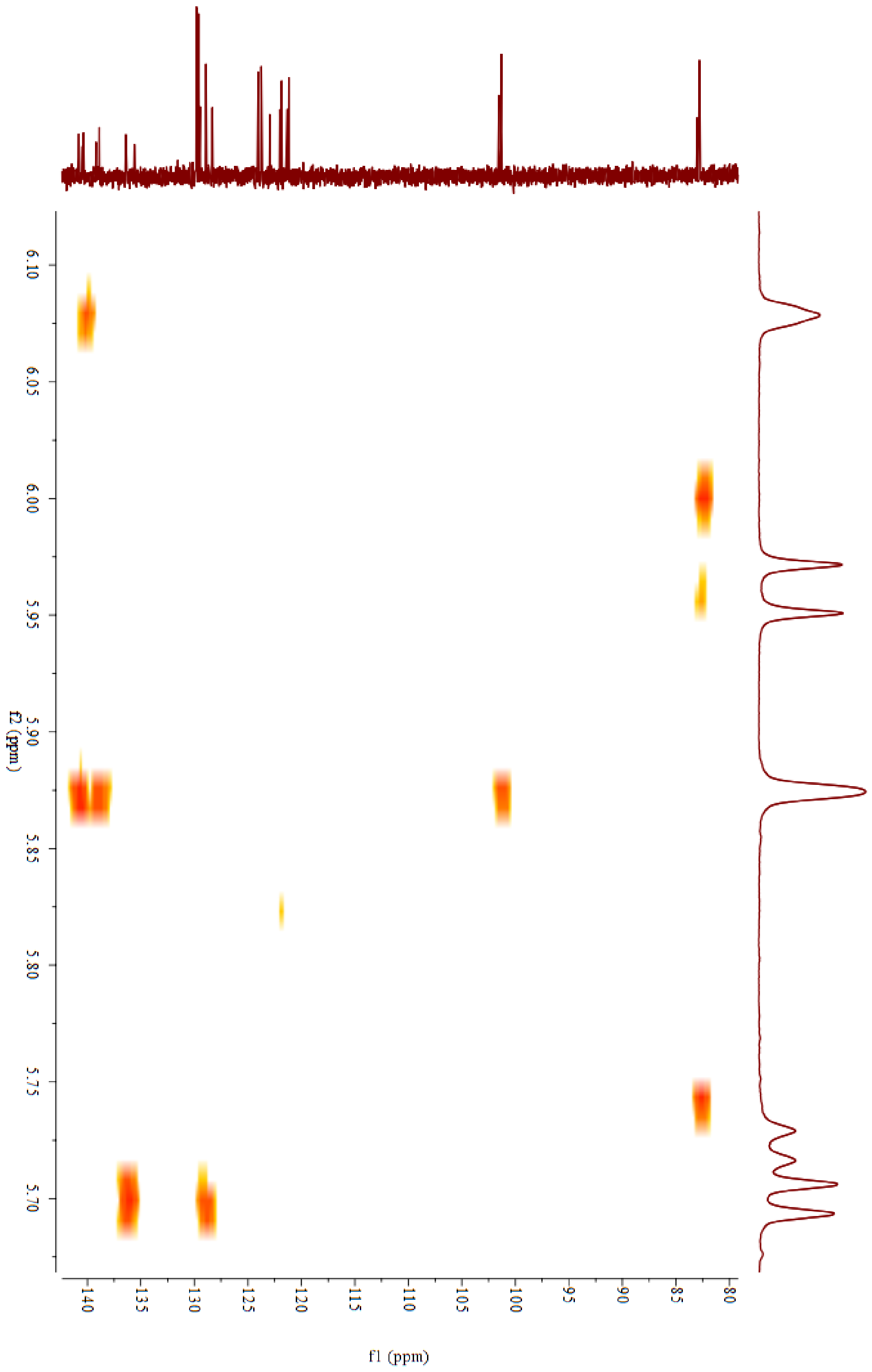

Figure S107. Continued

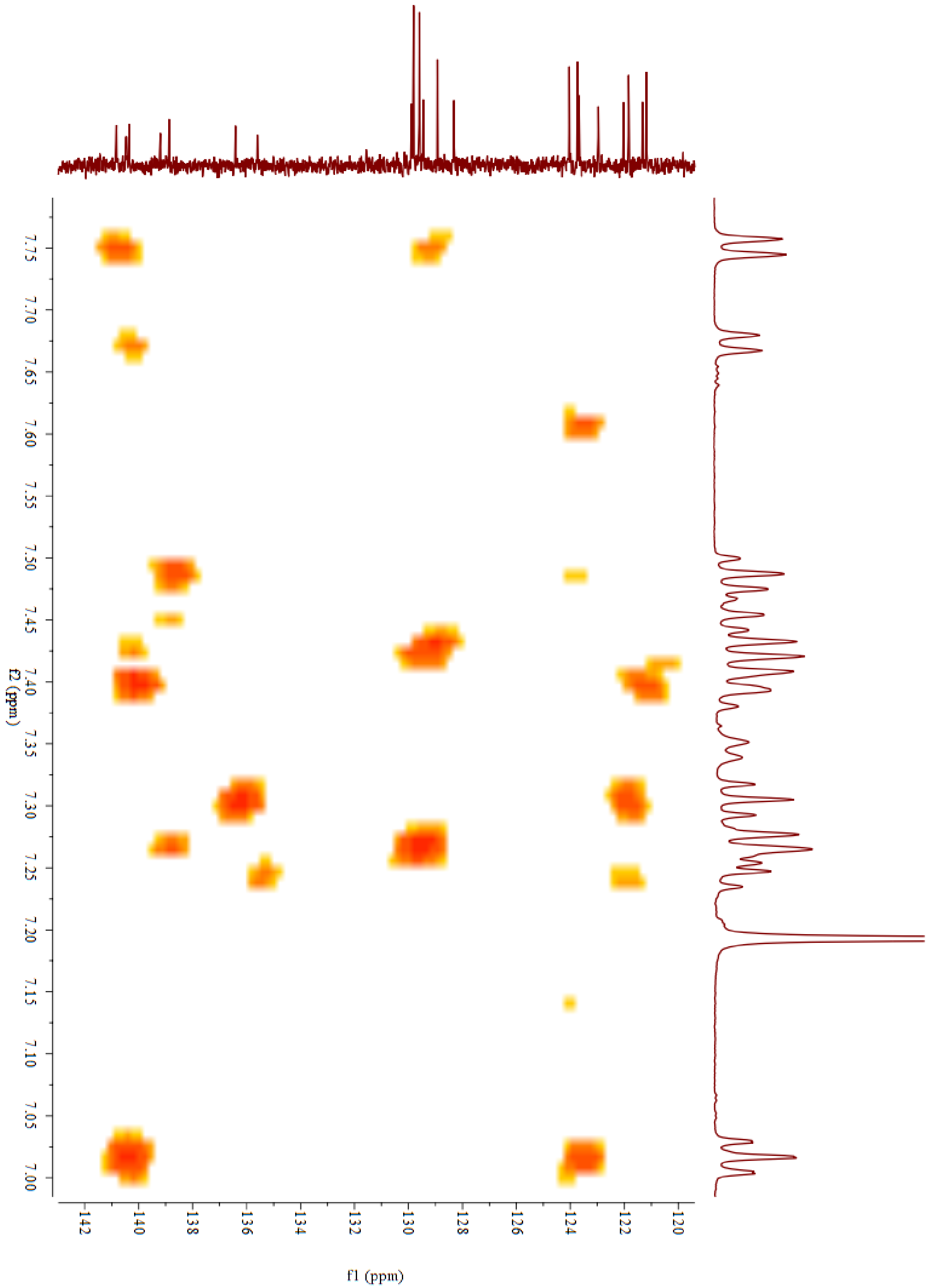

Figure S107. Continued

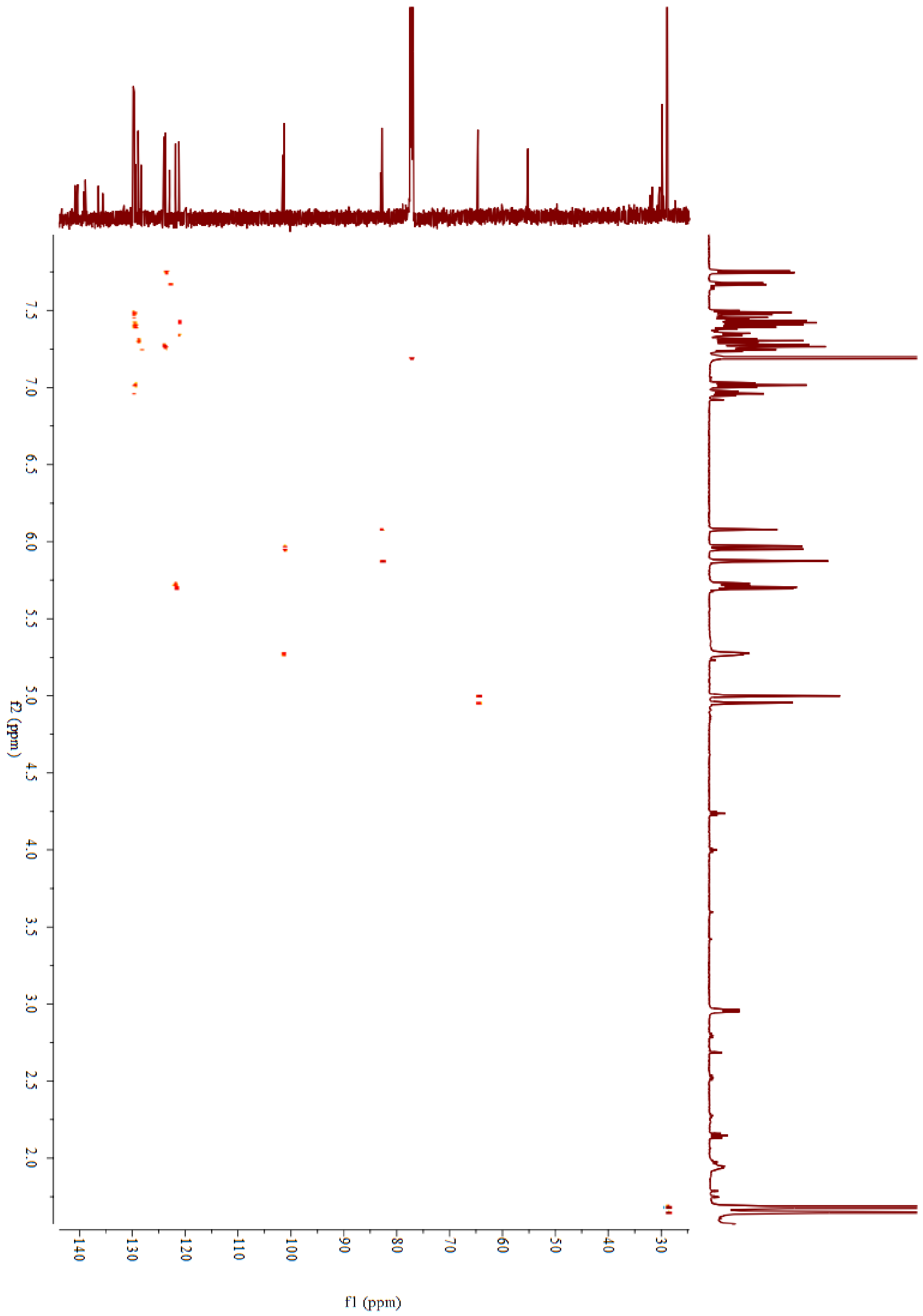

Figure S108. HSQC of compound **9.**

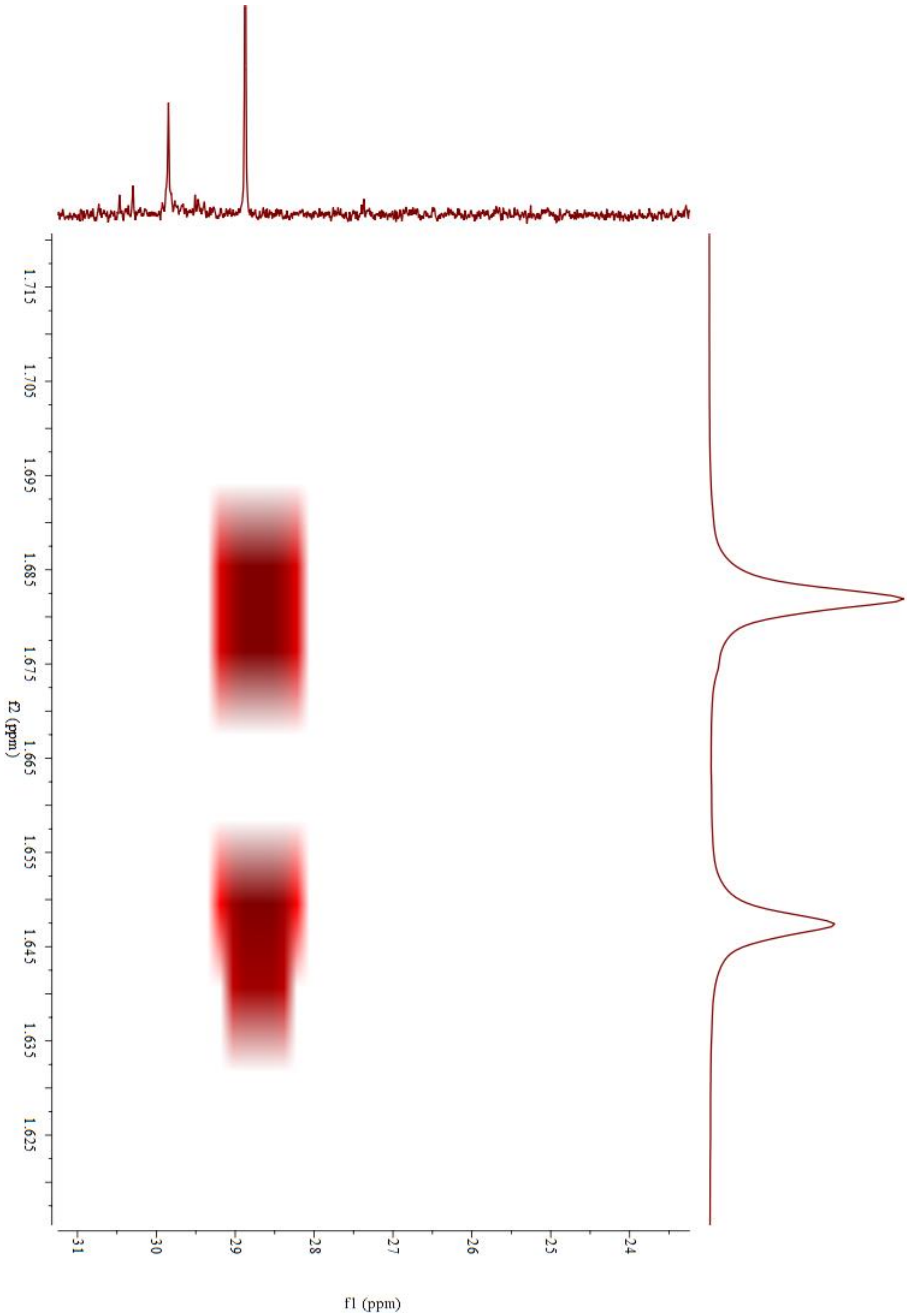

Figure S108. Continued

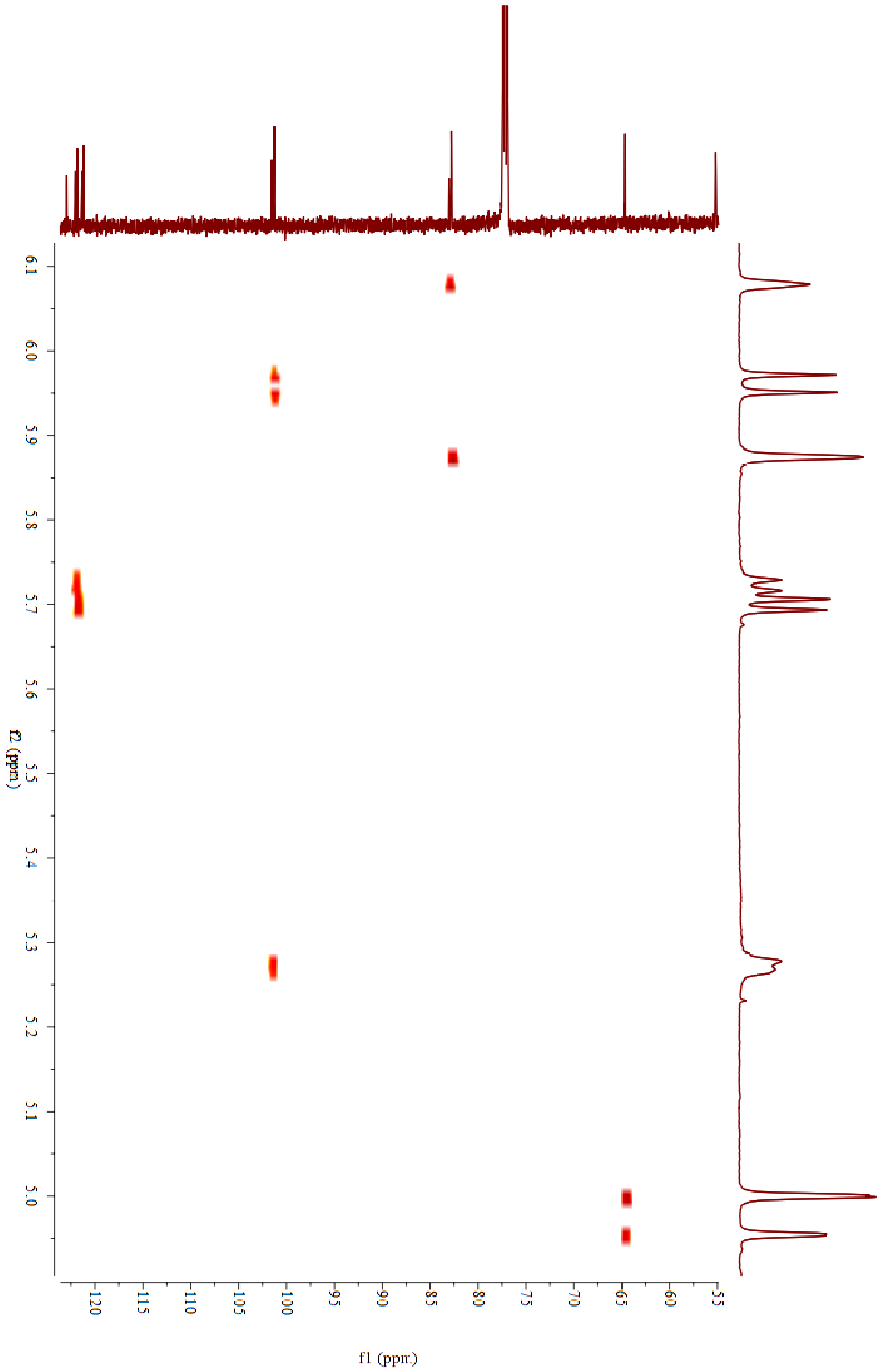

Figure S108. Continued

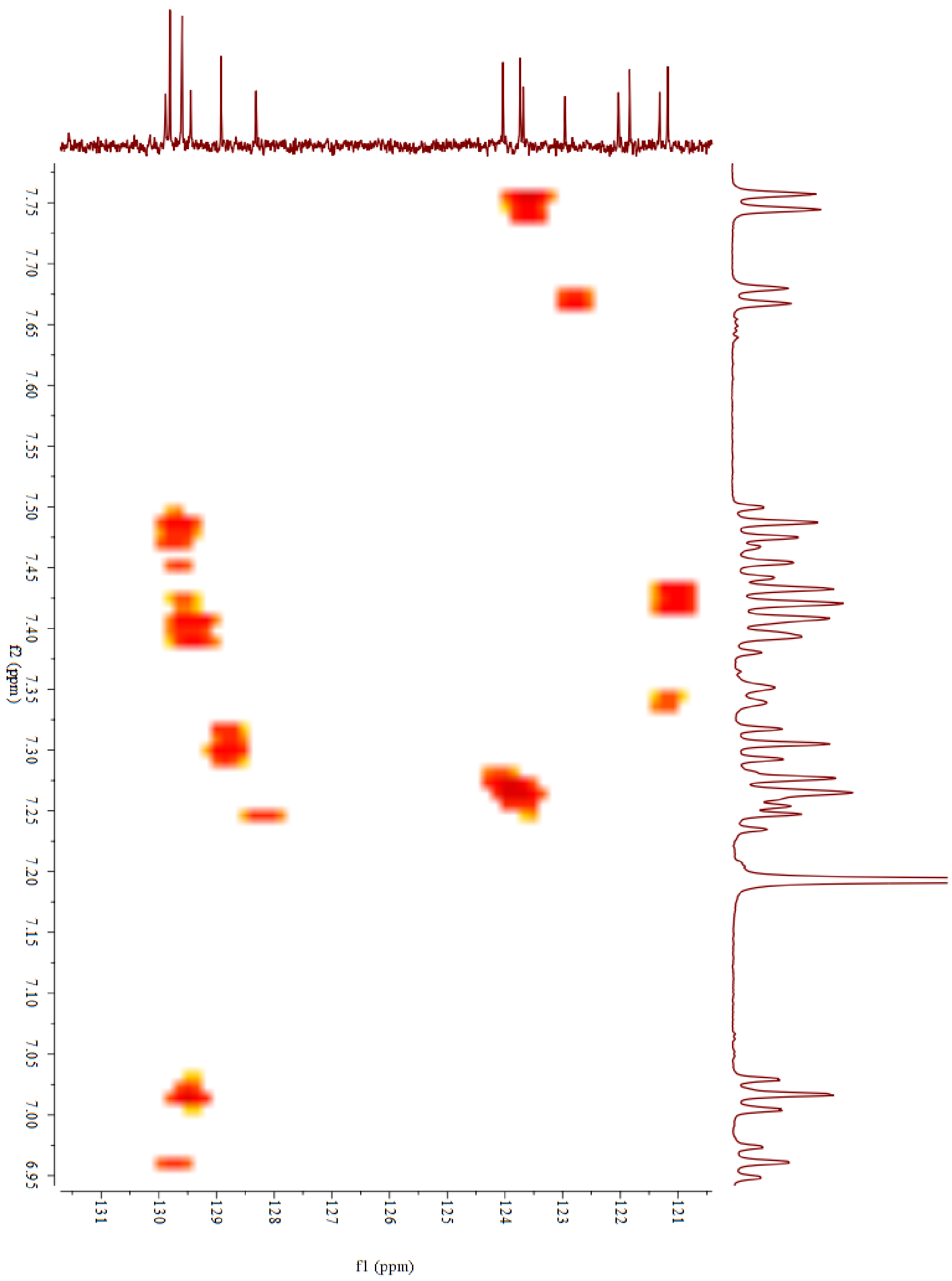

Figure S108. Continued

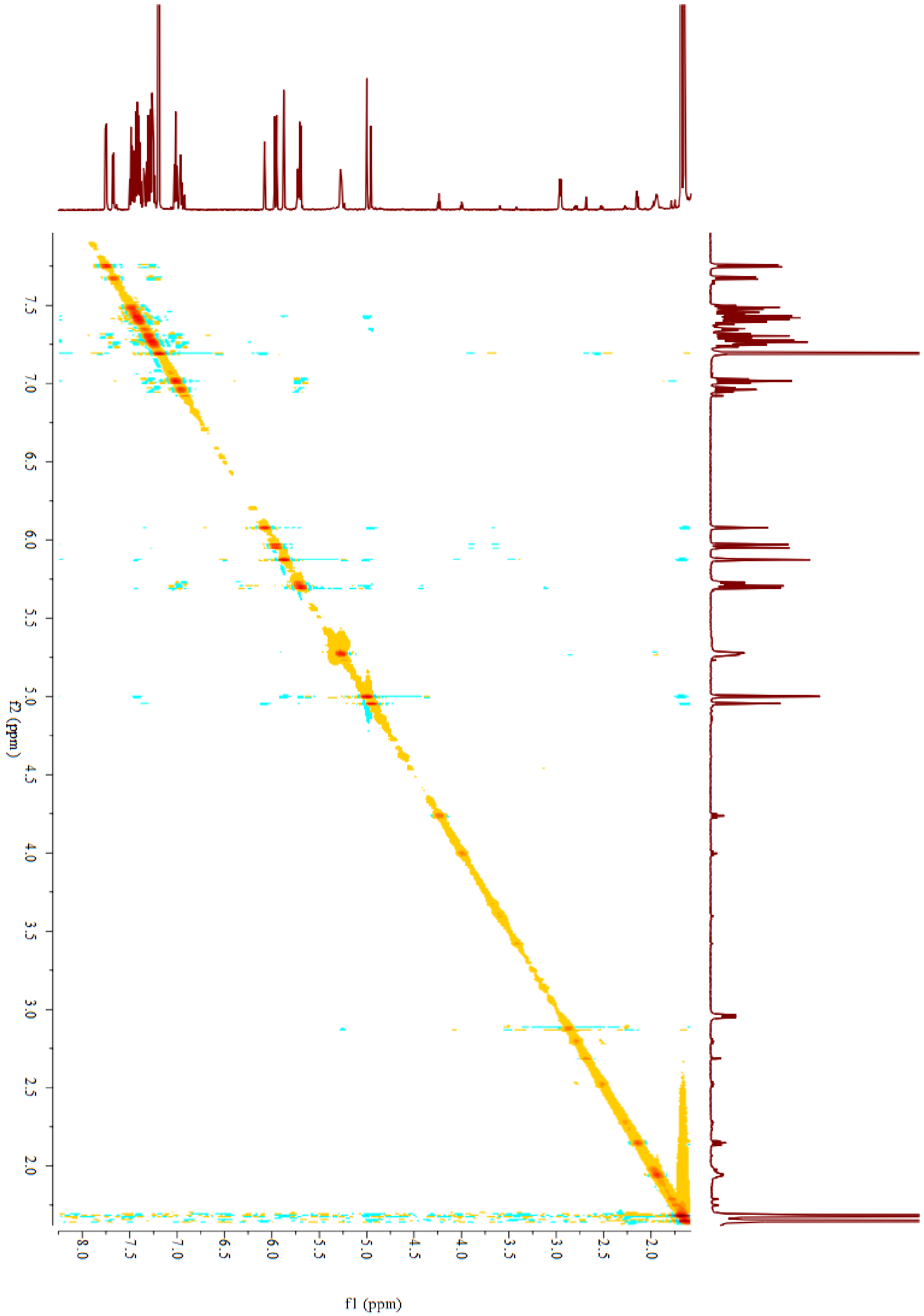

Figure S109. NOSEY of compound **9**

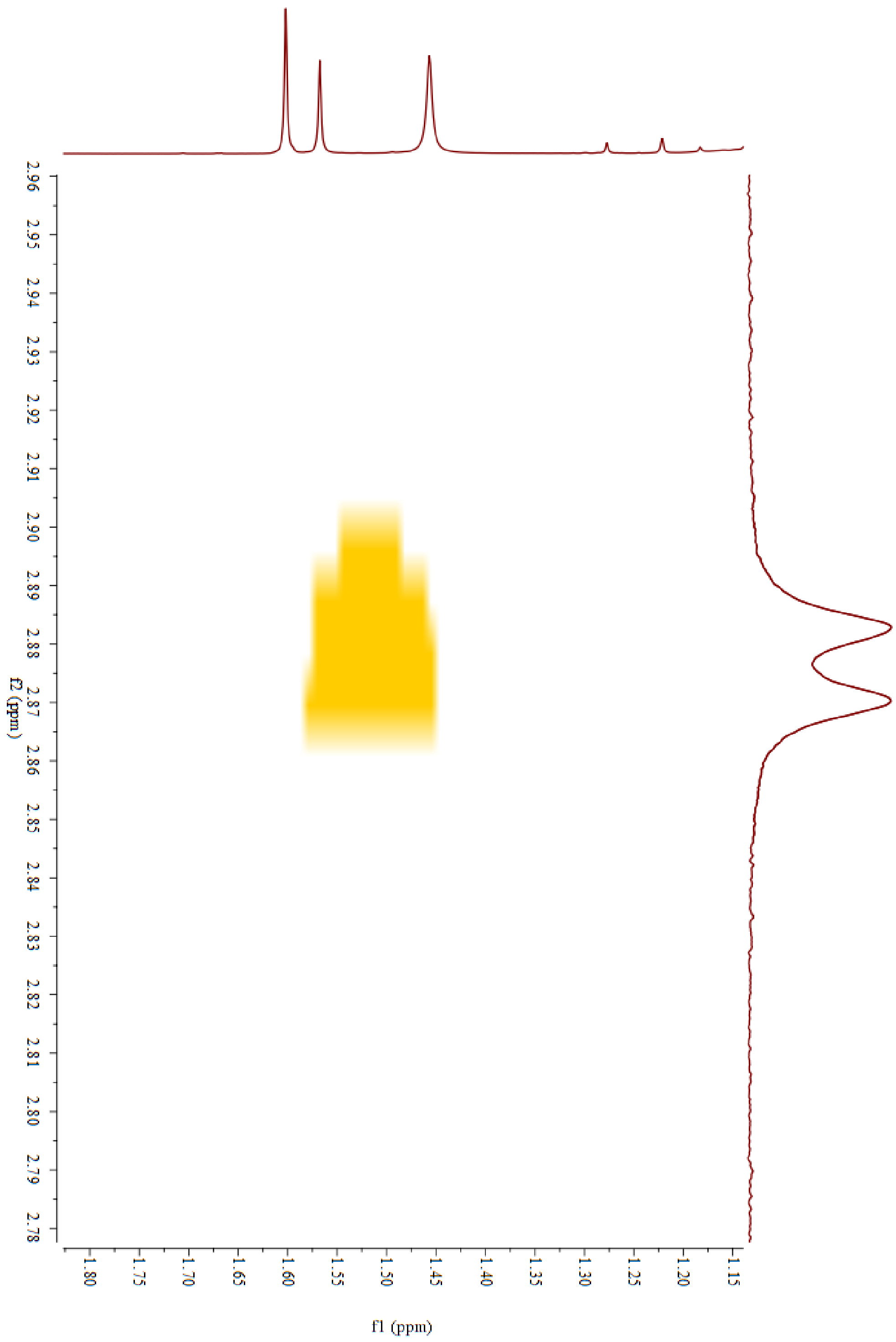

Figure S109. Continued

SEM pictures for 4 and 15.

For enantiomer peak 1 in Figure 3

Growth in methanol

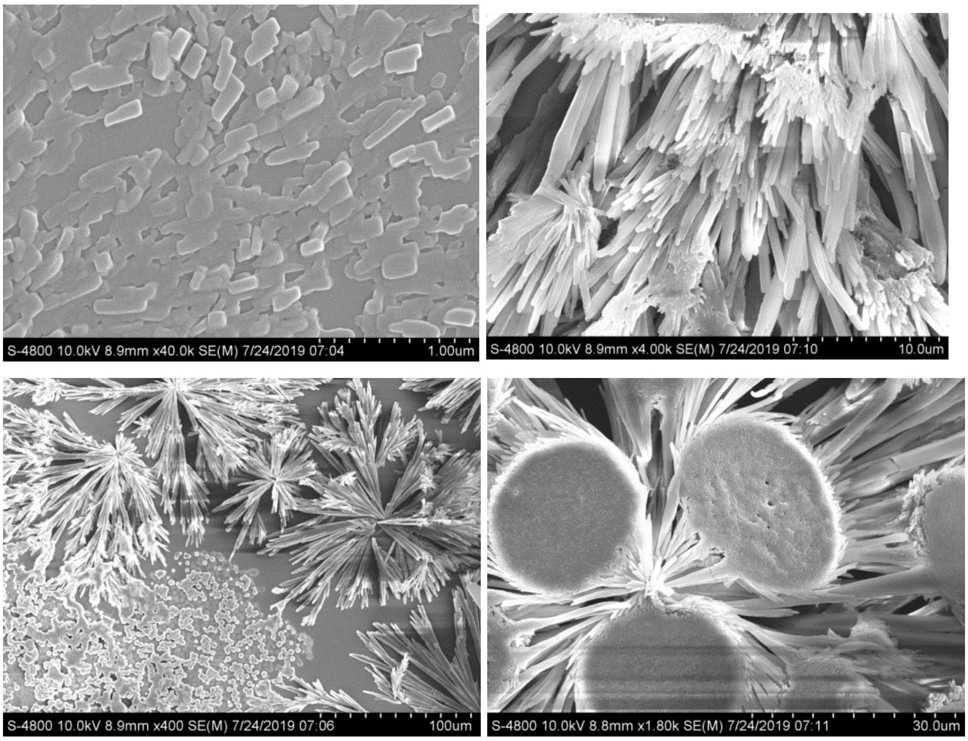

Growth in mixture of methanol/CH_2Cl_2

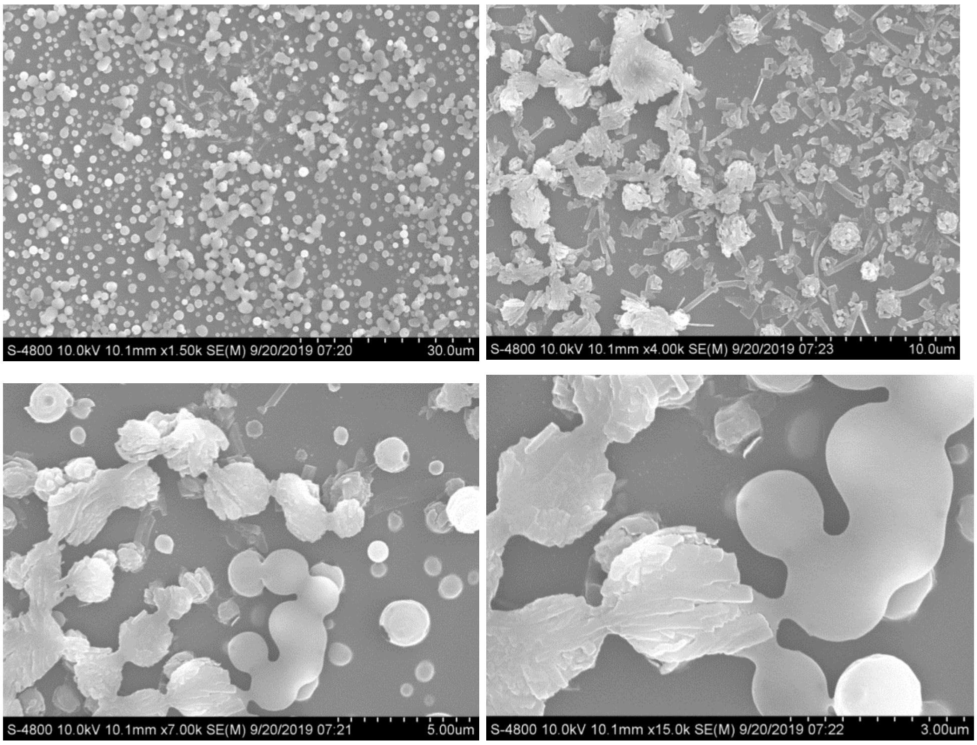

Figure S110. The SEM pictures for compounds **4** and **15** grew in different solvents

For enantiomer peak 4 in Figure 3
Growth in methanol

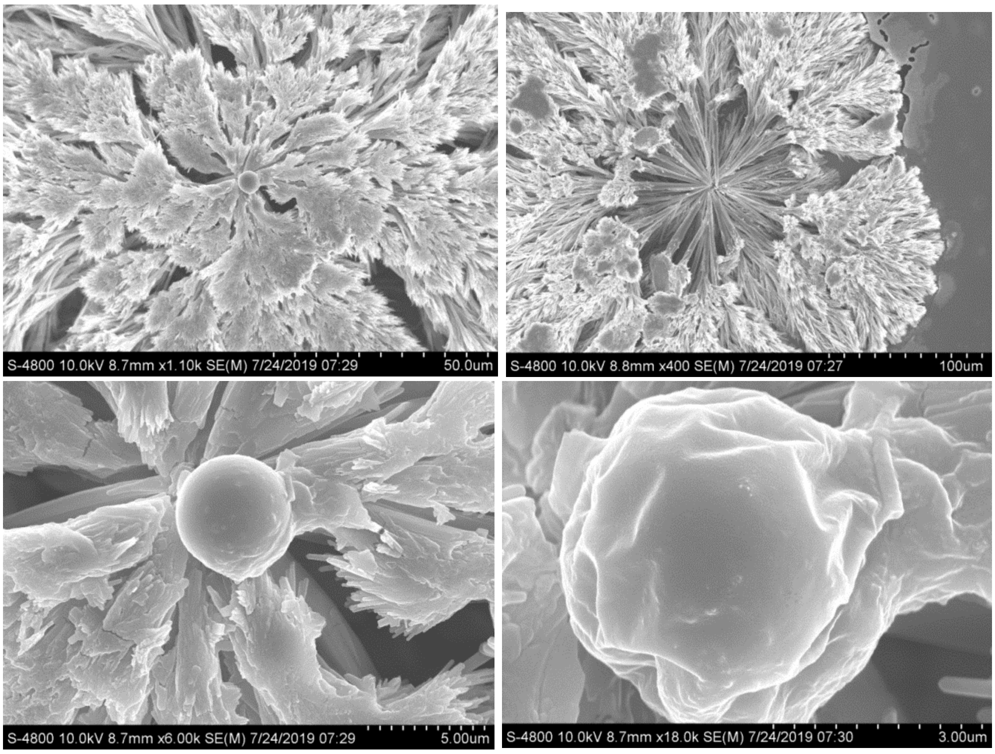

Growth in the mixture of methanol and CH_2Cl_2

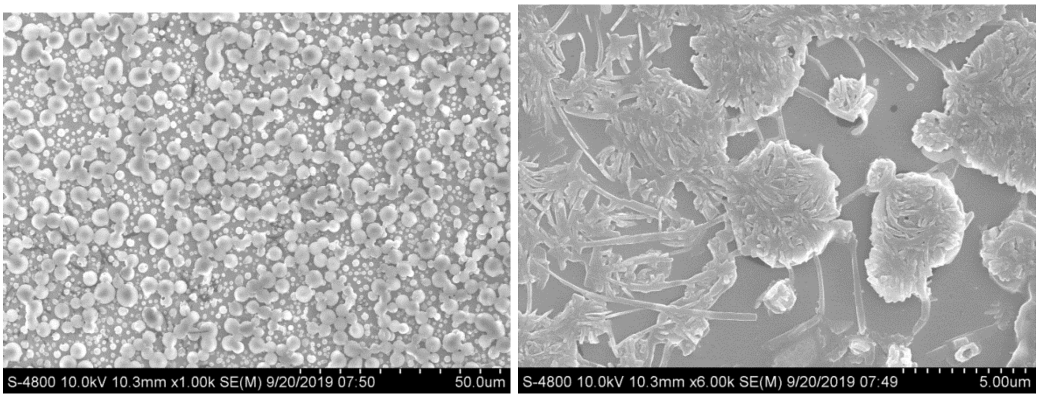

Figure S110**.** continued

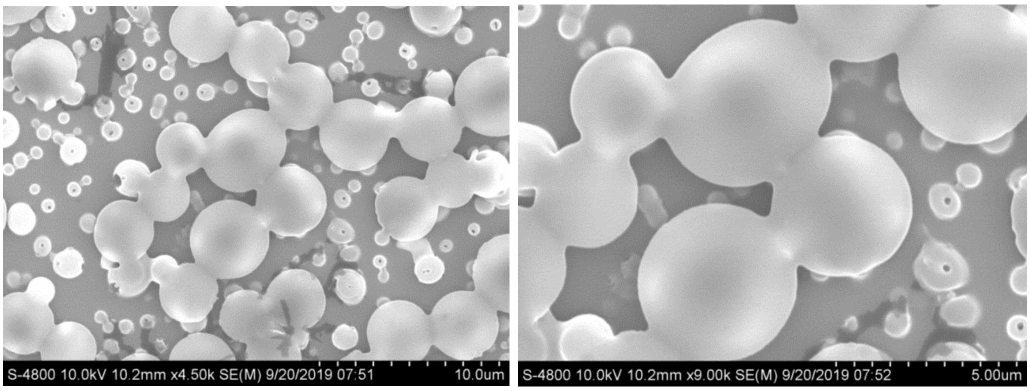

Racemate of **4**

Growth in methanol

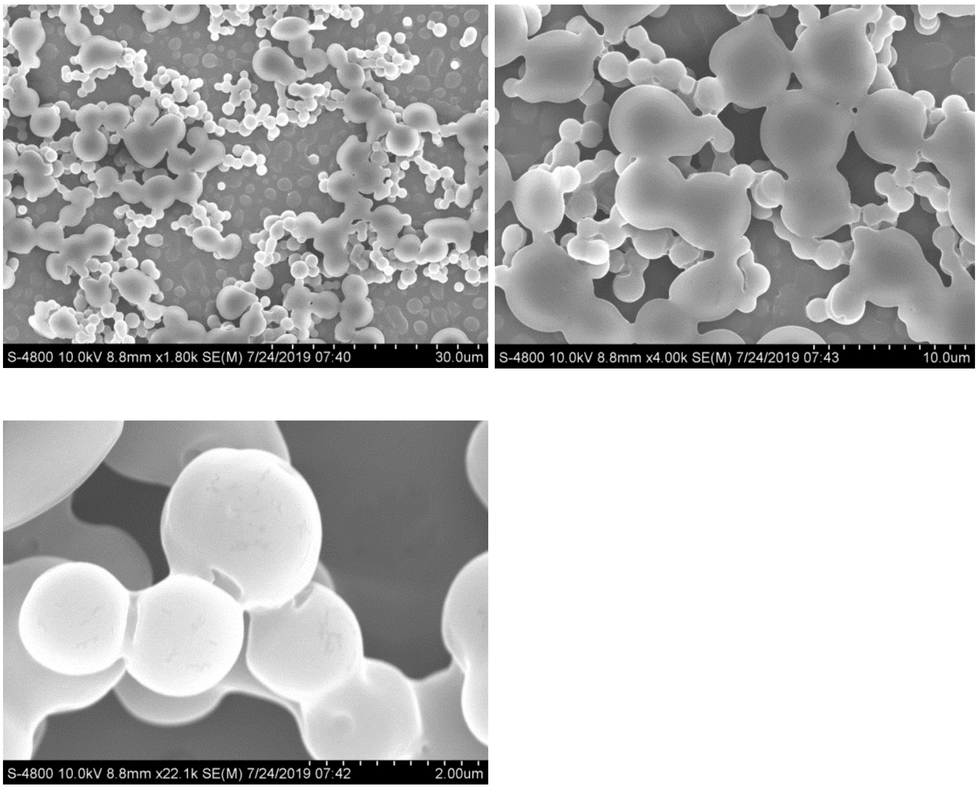

Figure S110**.** continued

Growth in mixture of methanol and CH_2Cl_2

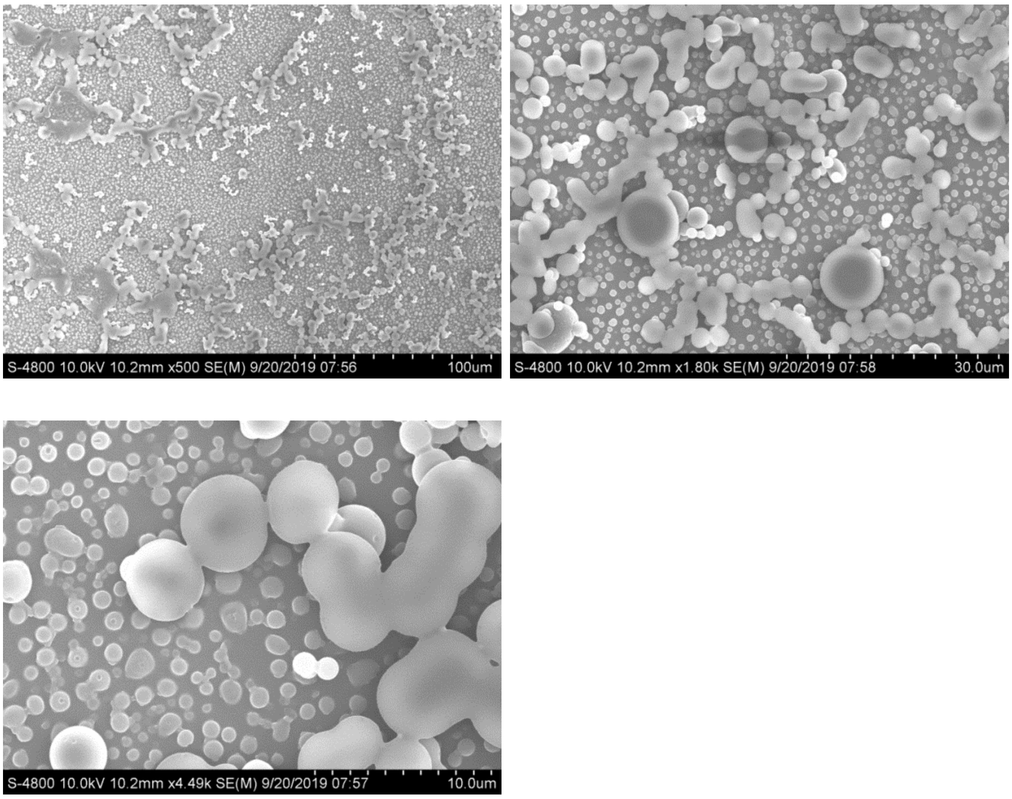

Figure S110. continued

For **15** growth in methanol

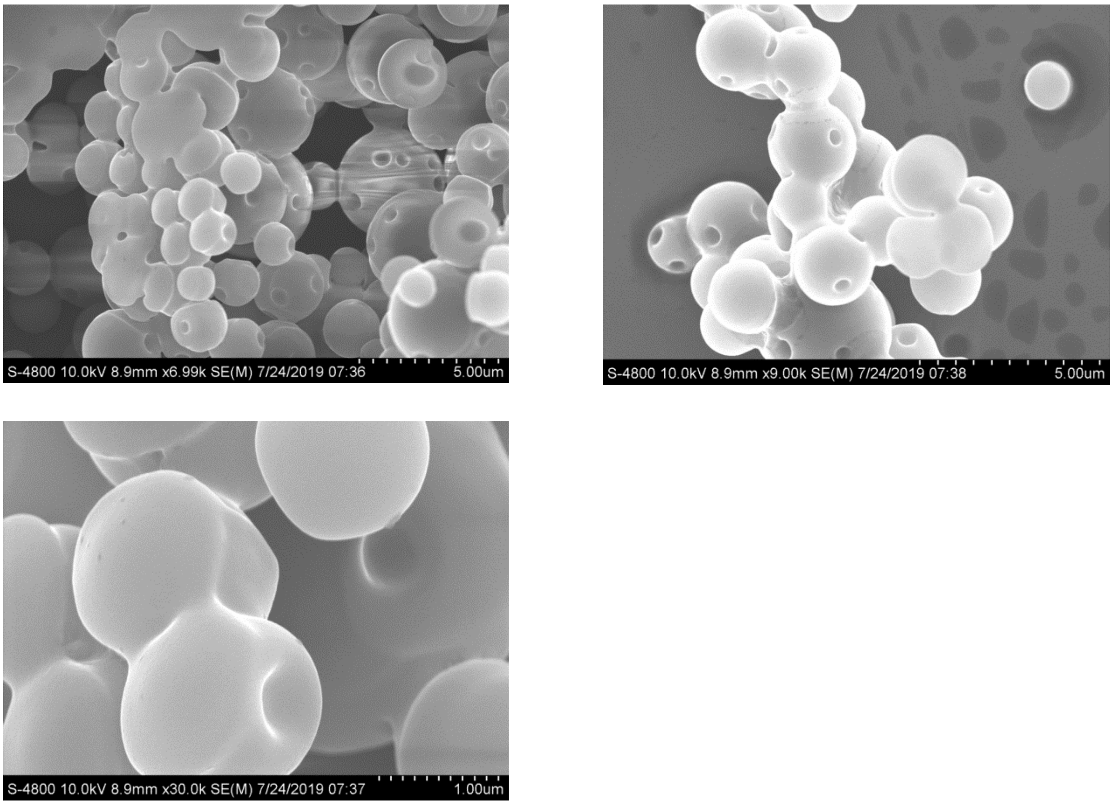

Growth in mixture of methanol/CH_2Cl_2

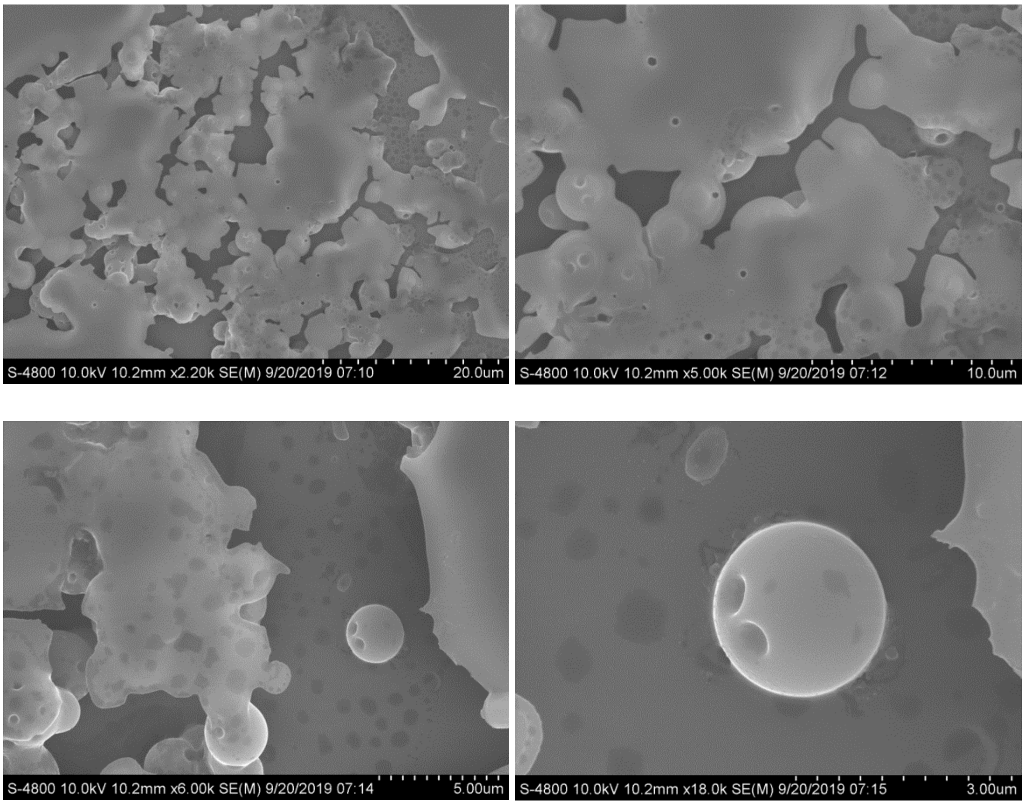

Figure S110. continued .

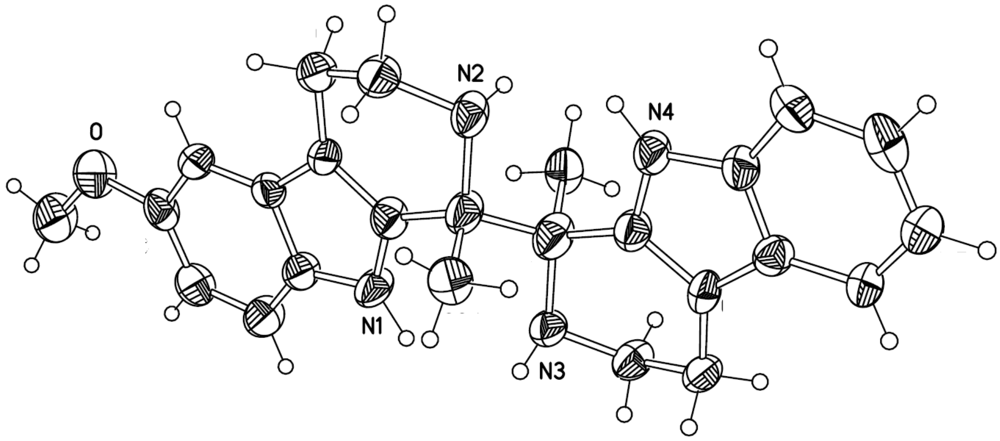

Figure 111. X-ray structure of **38**.

HRMS calcd for : $C_{25}H_{30}N_4O$ [M+H$^+$]: 401.2341, found 401.2349. ^{1}H NMR (600 MHz, CD_2Cl_2) δ 9.86 (s, 1H), 9.74 9S, 1H), 7.40 (d, J = 7.7 Hz, 1H), 7.28 (m, 1H), 7.16 (m, 1H), 7.03 (t, J = 7.4 Hz, 1H), 6.96 (t, J = 7.4 Hz, 1H), 6.87 (s, 1H), 6.68 (dd, J = 8.7, 2.3 Hz, 1H), 3.74 (s, 3H), 3.22 (s, 4H), 2.71 (m, 2H), 2.60 (m, 2H), 1.25 (s, 6H).

^{13}C NMR (151 MHz, CD_2Cl_2) δ 154.40 (s), 136.06, 131.20, 128.00, 127.68, 121.84, 119.34, 118.43, 112.19, 111.56, 110.25, 110.11, 100.71, 60.99, 56.37, 41.43, 25.98, 23.31.

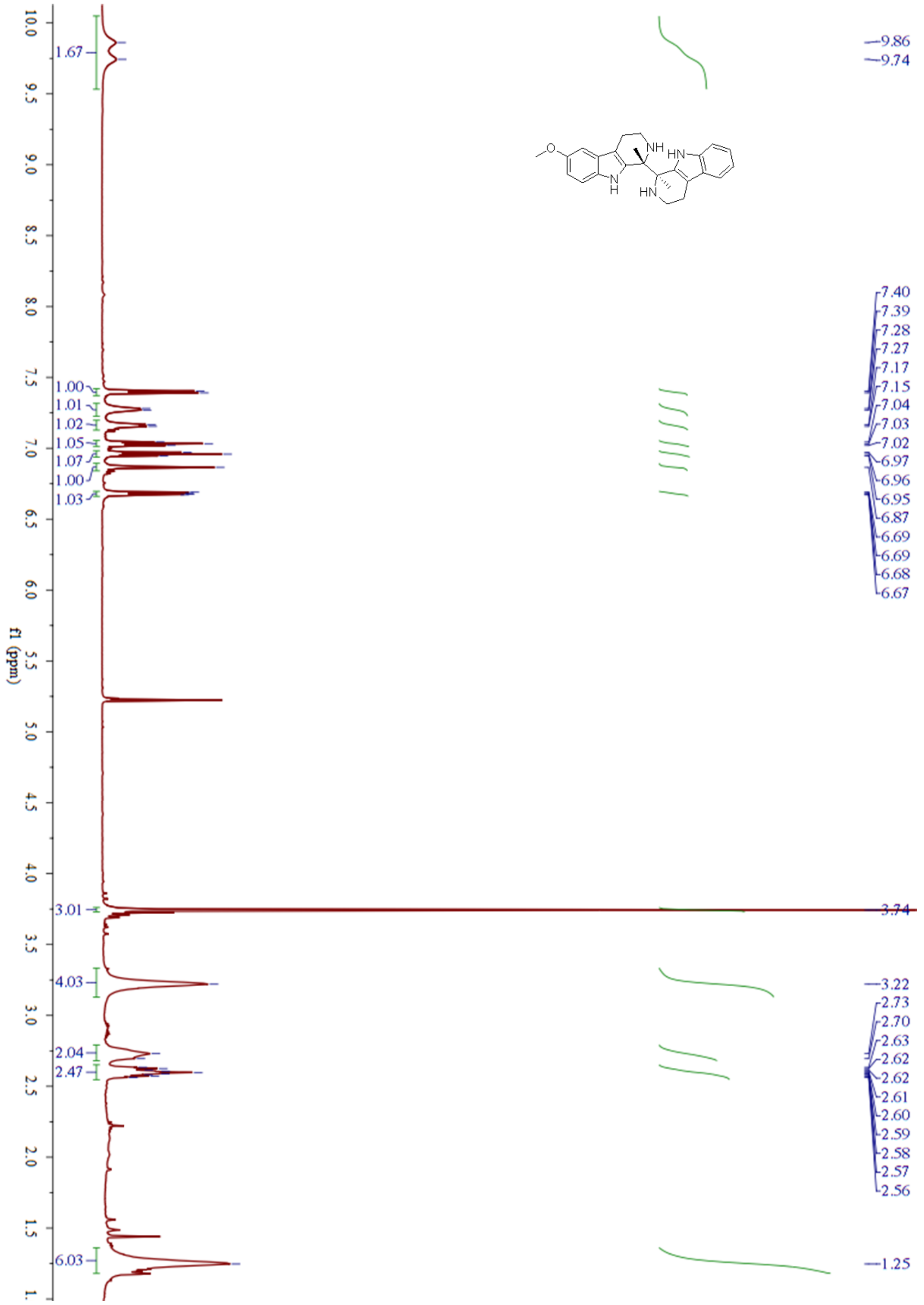

Figure S112. ^{1}H NMR for compound **38** in CD_2Cl_2.

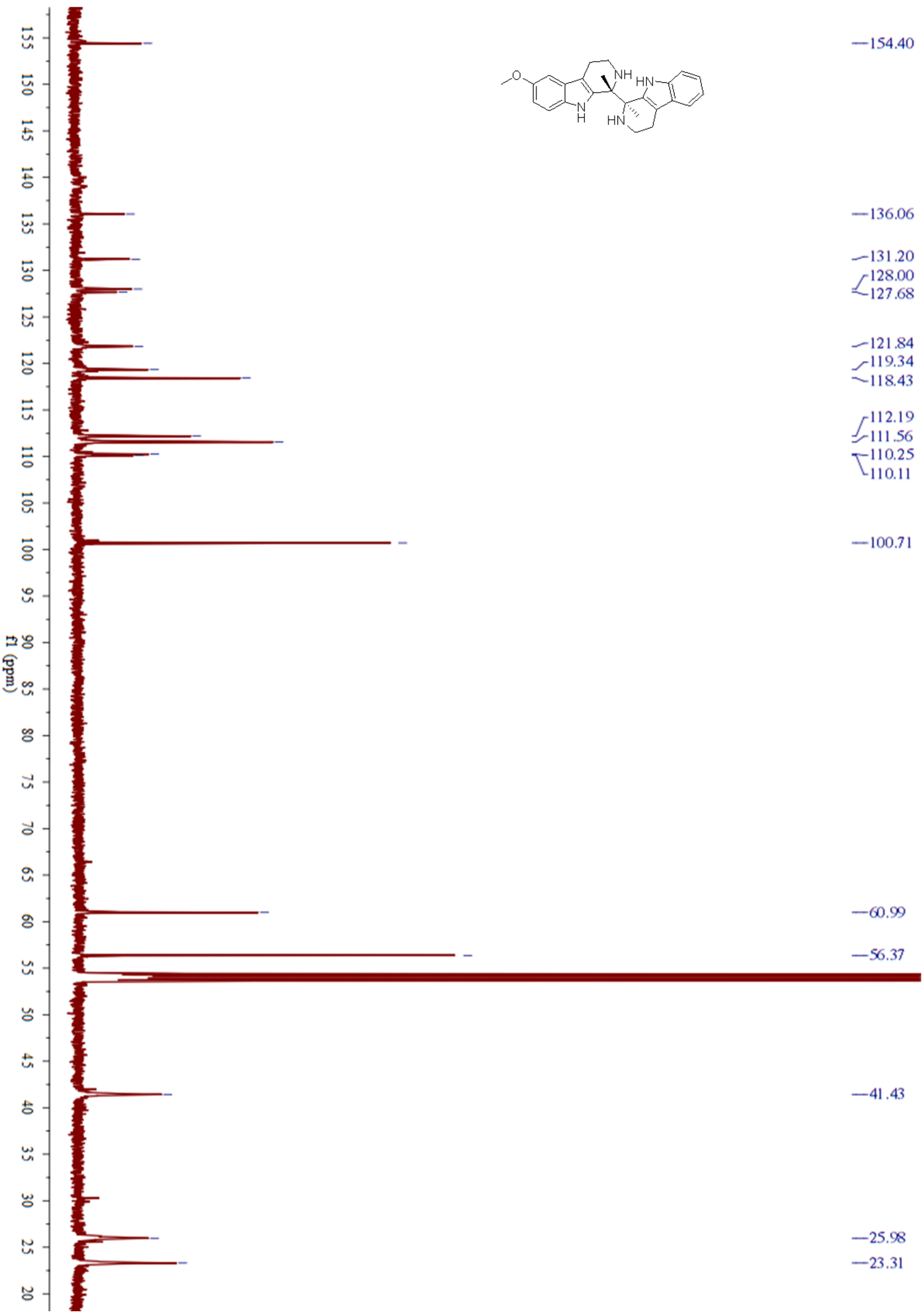

Figure S113. ^{13}CNMR for compound **38** in CD_2Cl_2.

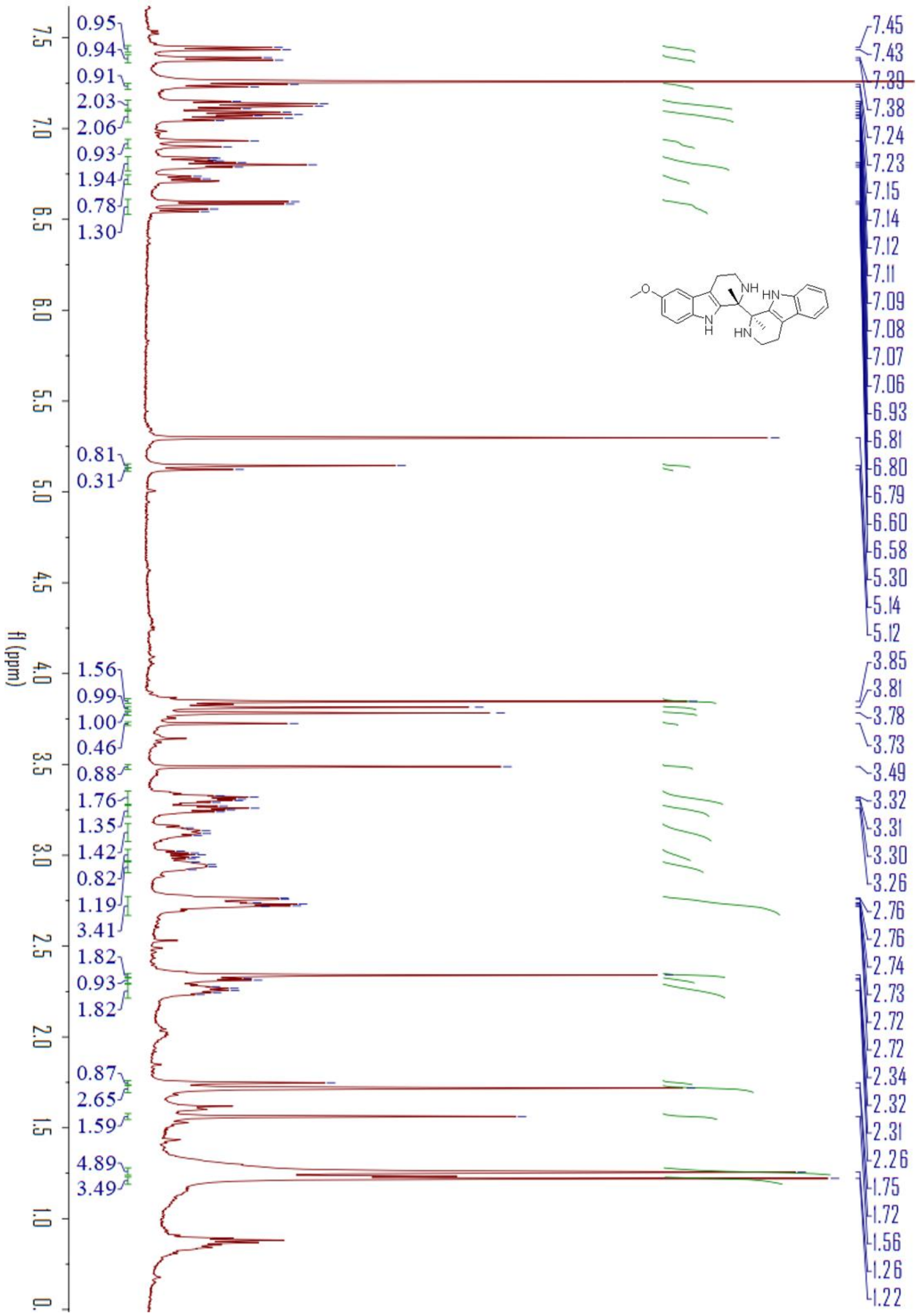

Figure S114. The ^{1}H NMR in $CDCl_3$+CD_3OD (near 3:1, v/v).

Crystal data for raceme 4

Identification code	1_a	
Empirical formula	$C_{19} H_{19} N O_3$	
Formula weight	309.35	
Temperature	296(2) K	
Wavelength	0.71073 Å	
Crystal system	Monoclinic	
Space group	$P2_1/n$	
Unit cell dimensions	a = 9.1741(16) Å	α= 90°.
	b = 9.7563(18) Å	β= 104.021(4)°.
	c = 18.591(4) Å	γ = 90°.
Volume	1614.4(5) $Å^3$	
Z	4	
Density (calculated)	1.273 Mg/m^3	
Absorption coefficient	0.086 mm^{-1}	
F(000)	656	
Crystal size	0.220 x 0.200 x 0.180 mm^3	
Theta range for data collection	2.258 to 25.488°.	
Index ranges	-11<=h<=11, -9<=k<=11, -22<=l<=21	
Reflections collected	9913	
Independent reflections	2967 [R(int) = 0.0596]	
Completeness to theta = 25.242°	100.0 %	
Absorption correction	Semi-empirical from equivalents	
Refinement method	Full-matrix least-squares on F^2	
Data / restraints / parameters	2967 / 1 / 213	
Goodness-of-fit on F^2	1.035	
Final R indices [I>2sigma(I)]	R1 = 0.0607, wR2 = 0.1632	
R indices (all data)	R1 = 0.0998, wR2 = 0.1913	
Extinction coefficient	n/a	
Largest diff. peak and hole	0.521 and -0.276 $e.Å^{-3}$	

Table S1. Atomic coordinates (x 10^4) and equivalent isotropic displacement parameters ($Å^2$x 10^3) for 1_a. U(eq) is defined as one third of the trace of the orthogonalized U^{ij} tensor.

	x	y	z	U(eq)
C(1)	2421(3)	8640(3)	4895(2)	49(1)
C(2)	1497(4)	8552(3)	5378(2)	62(1)
C(3)	2091(4)	8051(4)	6085(2)	72(1)
C(4)	3603(4)	7685(4)	6308(2)	73(1)
C(5)	4530(4)	7779(3)	5820(2)	60(1)
C(6)	3909(3)	8258(3)	5110(2)	47(1)
C(7)	4578(3)	8312(3)	4454(2)	47(1)
C(8)	2062(3)	9110(3)	4104(2)	56(1)
C(9)	5007(3)	6874(3)	4238(2)	44(1)
C(10)	3701(3)	5903(3)	4022(2)	43(1)
C(11)	2733(3)	5401(3)	4428(2)	49(1)
C(12)	1641(3)	4462(3)	4085(2)	60(1)
C(13)	1508(4)	4051(3)	3365(2)	65(1)
C(14)	2460(4)	4565(3)	2955(2)	63(1)
C(15)	3556(3)	5486(3)	3301(2)	48(1)
C(16)	4790(3)	6117(3)	3023(2)	51(1)
C(17)	7100(3)	7469(3)	3596(2)	70(1)
C(18)	8236(4)	6385(4)	3636(3)	107(2)
C(19)	7052(4)	8643(4)	3111(2)	94(1)
N(1)	5603(2)	6880(2)	3578(1)	50(1)
O(1)	1689(3)	10477(2)	4055(2)	81(1)
O(2)	3419(2)	8870(2)	3870(1)	58(1)
O(3)	5045(2)	5950(2)	2406(1)	66(1)

Crystal data for enantiomer of 4

Identification code	y	
Empirical formula	C19 H19 N O3	
Formula weight	309.35	
Temperature	296(2) K	
Wavelength	0.71073 Å	
Crystal system	Orthorhombic	
Space group	$P2_12_12_1$	
Unit cell dimensions	a = 9.0348(16) Å	α= 90°.
	b = 10.2479(19) Å	β= 90°.
	c = 17.661(3) Å	γ= 90°.
Volume	1635.2(5) $Å^3$	
Z	4	
Density (calculated)	1.257 Mg/m^3	
Absorption coefficient	0.085 mm^{-1}	
F(000)	656	
Crystal size	0.120 x 0.100 x 0.100 mm^3	
Theta range for data collection	2.298 to 24.742°.	
Index ranges	-10<=h<=10, -12<=k<=12, -20<=l<=19	
Reflections collected	10933	
Independent reflections	2787 [R(int) = 0.0278]	
Completeness to theta = 24.743°	99.8 %	
Absorption correction	None	
Refinement method	Full-matrix least-squares on F^2	
Data / restraints / parameters	2787 / 0 / 211	
Goodness-of-fit on F^2	1.072	
Final R indices [I>2sigma(I)]	R1 = 0.0339, wR2 = 0.0913	
R indices (all data)	R1 = 0.0394, wR2 = 0.0954	
Absolute structure parameter	0.2(5)	
Extinction coefficient	n/a	
Largest diff. peak and hole	0.273 and -0.145 e.$Å^{-3}$	

Table S2 Atomic coordinates ($\times10^4$) and equivalent isotropic displacement parameters ($Å^2\times10^3$) for Y. U(eq) is defined as one third of the trace of the orthogonalized U^{ij} tensor.

	x	y	z	U(eq)
C(1)	6369(3)	1252(2)	8327(1)	41(1)
C(2)	6593(3)	627(2)	7583(1)	42(1)
C(3)	7945(3)	1023(2)	7298(1)	38(1)
C(4)	8667(3)	1947(2)	7847(1)	39(1)
C(5)	8428(3)	554(2)	6605(1)	44(1)
C(6)	7541(3)	-329(3)	6229(2)	57(1)
C(7)	6214(4)	-743(3)	6528(2)	65(1)
C(8)	5707(3)	-256(3)	7205(2)	58(1)
C(9)	7826(3)	2621(3)	9203(1)	45(1)
C(10)	9320(4)	2249(4)	9523(2)	69(1)
C(11)	7629(4)	4083(3)	9195(2)	63(1)
C(12)	9001(3)	3287(2)	7511(1)	45(1)
C(13)	10061(3)	3235(2)	6860(1)	43(1)
C(14)	9367(3)	3697(3)	6222(1)	46(1)
C(15)	7836(3)	4164(3)	6424(2)	54(1)
C(16)	10079(4)	3685(3)	5532(2)	63(1)
C(17)	11509(4)	3212(3)	5503(2)	71(1)
C(18)	12216(4)	2772(3)	6146(2)	68(1)
C(19)	11500(3)	2764(3)	6837(2)	56(1)
N(1)	7575(2)	1994(2)	8461(1)	42(1)
O(1)	5307(2)	1126(2)	8758(1)	56(1)
O(2)	7690(2)	3838(2)	7208(1)	61(1)
O(3)	7707(2)	5489(2)	6320(1)	67(1)

_

Transition state calculations for 4A and 4B, and low energy computation for epi-4

Table S3 The calculated transition state barriers for two conformers (monomer A and monomer B) with the lowest and second lowest energy.

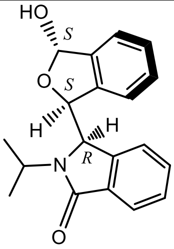<br>Monomer A	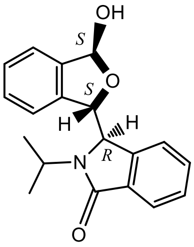<br>Monomer B	The transition state barriers between the conformations A to B were smaller than 10.0 kcal/mol in the gas phase or in liquid (see below). The low barrier indicated that the conversion of geometry from A to B is very easy at room temperature and the conversion rate is fast. It is difficult to observe the two sets of NMR data at room temperature.

These geometries and the transition state structures were performed at the B3LYP/6-311++G(2d,p) level in the gas phase and in solution using PCM model, respectively.

**Monomer-A** 3D geometry	**Monomer-B** 3D geometry	**TS** 3D geometry (between monomer-A and -B)	**Epi-4** in Scheme 1
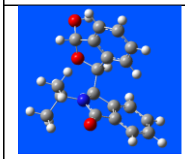	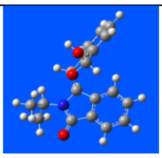	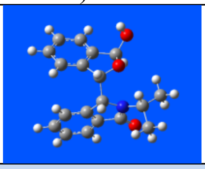	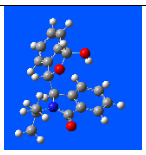

	E (a.u.)	
	In the gas phase	In solution
Monomer-A	-1016.206116	-1016.216891(CH_2Cl_2); -1016.221470(MeOH)
Monomer-B	-1016.205441	-1016.217133(CH_2Cl_2); -1016.222600(MeOH)
Monomer-TS	-1016.190645	-1016.201752(CH_2Cl_2); -1016.206795(MeOH)
Epi-**4**	-1016.207004	-1016.220457 (CH_2Cl_2); -1016.223053 (MeOH)

ΔE	In gas phase	In solution
$\Delta E_{(A\text{-}B)}$ (kcal/mol)	0.423	
$\Delta E_{(B\text{-}A)}$ (kcal/mol)		0.152 (CH_2Cl_2), 0.70 (MeOH)
$\Delta E_{(TS\text{-}A)}$ (kcal/mol) (from geometry A to B)	9.71	9.50 （CH_2Cl_2）; 9.21 （MeOH）
$\Delta E_{(TS\text{-}B)}$ (kcal/mol) (from geometry B to A)	9.28	9.65 （CH_2Cl_2）; 9.91 （MeOH）
Monomer-A : Monomer -B		67:33 (2:1) (in the gas phase)
Monomer-B : Monomer-A		44:56 (1:1.27) (CH_2Cl_2)
Monomer-B : Monomer-A		41.4:58.6 (1:1.41) (MeOH)
Epi-**4**	0.557	2.086 (CH_2Cl_2); 0.284 (MeOH)
Monomer **4**: epi-**4**	0.39:1.00	0.029 : 1 (CH_2Cl_2); 0.63 : 1.00 (MeOH)

Table S4 Coordinated for transitions state structures and the most stable and the second stable monomer geometries, the most stable dimers for **4** in the text.

TS coordinate for **4** at the B3LYP/6-311++G(2d,p) level in the gas phase

Standard orientation

Center Number	Atomic Number	Atomic Type	Coordinates (Angstroms) X	Y	Z
1	6	0	2.087472	-1.173438	-0.446572
2	6	0	3.268202	-1.007974	-1.155252
3	1	0	3.452819	-1.571296	-2.062322
4	6	0	4.222146	-0.126358	-0.653710
5	1	0	5.156699	0.016599	-1.182587
6	6	0	3.992939	0.554140	0.541837
7	1	0	4.755962	1.214111	0.937020
8	6	0	2.798300	0.389074	1.238645
9	1	0	2.644694	0.909064	2.176049
10	6	0	1.833569	-0.463407	0.717600
11	6	0	0.481277	-0.880470	1.249578
12	1	0	0.545041	-1.164001	2.303086
13	6	0	0.959134	-2.140312	-0.697314
14	1	0	0.330827	-1.870043	-1.550832
15	6	0	-0.730782	0.167827	1.126234
16	1	0	-1.135848	0.281832	2.138697
17	6	0	-0.415542	1.535546	0.554414
18	6	0	0.396305	2.556207	1.029388
19	1	0	0.956794	2.455854	1.949584
20	6	0	0.464715	3.744979	0.304809
21	1	0	1.093250	4.551173	0.664464
22	6	0	-0.269565	3.918158	-0.870152
23	1	0	-0.197011	4.852282	-1.414037
24	6	0	-1.111109	2.909222	-1.325700
25	1	0	-1.724487	3.030999	-2.210078
26	6	0	-1.170998	1.732509	-0.594057
27	6	0	-2.086713	0.576498	-0.774539
28	6	0	-2.699276	-1.400803	0.649829
29	1	0	-2.175533	-1.914618	1.457060
30	6	0	-4.011812	-0.830588	1.203428
31	1	0	-3.826624	-0.133486	2.024858
32	1	0	-4.639104	-1.639705	1.584924
33	1	0	-4.564648	-0.306805	0.422721
34	6	0	-2.939135	-2.420753	-0.464750

35	1	0	-3.561708	-3.232634	-0.080779
36	1	0	-1.996817	-2.850607	-0.802672
37	1	0	-3.446747	-1.961648	-1.311355
38	7	0	-1.790250	-0.310728	0.237182
39	8	0	1.344367	-3.459124	-0.939793
40	1	0	1.908602	-3.748831	-0.210097
41	8	0	0.189356	-2.070948	0.514554
42	8	0	-2.966121	0.457134	-1.611794

TS coordinate for **4** at the B3LYP/6-311++G(2d,p) level in chloroform

Standard orientation:

Center Number	Atomic Number	Atomic Type	Coordinates (Angstroms) X	Y	Z
1	6	0	2.092788	-1.178900	-0.443327
2	6	0	3.273897	-1.008844	-1.151707
3	1	0	3.470009	-1.580970	-2.050763
4	6	0	4.214634	-0.106972	-0.660701
5	1	0	5.148528	0.040424	-1.189374
6	6	0	3.972509	0.588634	0.524436
7	1	0	4.725418	1.264505	0.911845
8	6	0	2.777721	0.419080	1.220525
9	1	0	2.613684	0.951136	2.148809
10	6	0	1.825488	-0.453771	0.708659
11	6	0	0.477355	-0.875153	1.245160
12	1	0	0.544489	-1.152612	2.298996
13	6	0	0.981320	-2.165770	-0.681078
14	1	0	0.364346	-1.934930	-1.553686
15	6	0	-0.739725	0.166513	1.123053
16	1	0	-1.152458	0.270708	2.131754
17	6	0	-0.427611	1.536230	0.559758
18	6	0	0.372079	2.559831	1.048635
19	1	0	0.917029	2.459481	1.977592
20	6	0	0.445685	3.749867	0.325922
21	1	0	1.064583	4.558668	0.695861
22	6	0	-0.271300	3.920892	-0.860719
23	1	0	-0.194280	4.855225	-1.403360
24	6	0	-1.102261	2.908723	-1.329587
25	1	0	-1.699583	3.034441	-2.224325
26	6	0	-1.168829	1.730495	-0.599246
27	6	0	-2.076145	0.571051	-0.787310

28	6	0	-2.700044	-1.406287	0.634906
29	1	0	-2.169330	-1.921703	1.435391
30	6	0	-4.007699	-0.843424	1.205910
31	1	0	-3.813867	-0.150313	2.028044
32	1	0	-4.625950	-1.657773	1.590603
33	1	0	-4.574171	-0.318373	0.435630
34	6	0	-2.947887	-2.423624	-0.479859
35	1	0	-3.531574	-3.256150	-0.079916
36	1	0	-2.005878	-2.820968	-0.856715
37	1	0	-3.499827	-1.976955	-1.305177
38	7	0	-1.795145	-0.311472	0.223474
39	8	0	1.391317	-3.489963	-0.872679
40	1	0	1.997446	-3.729213	-0.157335
41	8	0	0.186560	-2.070368	0.511817
42	8	0	-2.948433	0.449611	-1.641484

TS coordinate for **4** at the B3LYP/6-311++G(2d,p) level in methanol

Standard orientation:

Center Number	Atomic Number	Atomic Type	Coordinates (Angstroms) X	Y	Z
1	6	0	2.088586	-1.185214	-0.440182
2	6	0	3.268087	-1.018081	-1.152486
3	1	0	3.465167	-1.597089	-2.046923
4	6	0	4.207102	-0.109043	-0.670992
5	1	0	5.139760	0.036341	-1.202374
6	6	0	3.964846	0.595725	0.509076
7	1	0	4.716455	1.276657	0.890012
8	6	0	2.771919	0.428481	1.209340
9	1	0	2.608731	0.966444	2.133999
10	6	0	1.820849	-0.451117	0.706132
11	6	0	0.474933	-0.870274	1.249453
12	1	0	0.545183	-1.140835	2.304228
13	6	0	0.980430	-2.177232	-0.667044
14	1	0	0.364607	-1.959664	-1.543837
15	6	0	-0.743056	0.169137	1.125092
16	1	0	-1.161217	0.269860	2.131016
17	6	0	-0.430090	1.539331	0.564990
18	6	0	0.362094	2.565653	1.061066
19	1	0	0.896718	2.468836	1.996249
20	6	0	0.439789	3.755081	0.337288

21	1	0	1.052301	4.565951	0.713066
22	6	0	-0.265411	3.923258	-0.857155
23	1	0	-0.185261	4.857158	-1.399996
24	6	0	-1.089412	2.908476	-1.333164
25	1	0	-1.676659	3.034751	-2.234626
26	6	0	-1.160708	1.730516	-0.601845
27	6	0	-2.063575	0.569323	-0.794858
28	6	0	-2.701272	-1.403415	0.628074
29	1	0	-2.173129	-1.916532	1.431248
30	6	0	-4.009777	-0.840268	1.196301
31	1	0	-3.816655	-0.148517	2.019333
32	1	0	-4.628163	-1.655282	1.579084
33	1	0	-4.575895	-0.314387	0.426037
34	6	0	-2.946584	-2.423892	-0.484327
35	1	0	-3.517254	-3.262467	-0.078596
36	1	0	-2.003524	-2.810163	-0.870314
37	1	0	-3.511160	-1.985733	-1.305753
38	7	0	-1.794481	-0.308949	0.218326
39	8	0	1.395222	-3.504270	-0.841557
40	1	0	2.019544	-3.726348	-0.135949
41	8	0	0.182197	-2.070408	0.521717
42	8	0	-2.928754	0.445303	-1.660504

The coordinate for the most stable monomer conformer of **4** in the gas phase (monomer A in text) at the B3LYP/6-311++G(2d,p)

Standard orientation:

Center Number	Atomic Number	Atomic Type	Coordinates (Angstroms) X	Y	Z
1	6	0	2.558775	-0.619980	0.122118
2	6	0	3.345292	-1.051270	1.181981
3	1	0	4.370259	-0.715025	1.285550
4	6	0	2.786782	-1.930847	2.104202
5	1	0	3.375681	-2.277271	2.944898
6	6	0	1.472410	-2.373246	1.951287
7	1	0	1.051667	-3.060182	2.675774
8	6	0	0.692158	-1.943200	0.881122
9	1	0	-0.324419	-2.298383	0.772053
10	6	0	1.247868	-1.048637	-0.026978
11	6	0	0.672966	-0.424106	-1.272582
12	1	0	0.471572	-1.188277	-2.034479

13	6	0	2.933980	0.283067	-1.021256
14	1	0	3.268449	1.277065	-0.717514
15	6	0	-0.634656	0.391229	-1.130860
16	1	0	-0.746965	0.939557	-2.074780
17	6	0	-1.832940	-0.494763	-0.880997
18	6	0	-2.325489	-1.569276	-1.609399
19	1	0	-1.854320	-1.898083	-2.528678
20	6	0	-3.463211	-2.220731	-1.134610
21	1	0	-3.868437	-3.058371	-1.689908
22	6	0	-4.092318	-1.807968	0.043035
23	1	0	-4.975410	-2.332114	0.388211
24	6	0	-3.596951	-0.728169	0.767092
25	1	0	-4.072507	-0.384104	1.677414
26	6	0	-2.464244	-0.087701	0.285764
27	6	0	-1.757316	1.102038	0.830062
28	6	0	0.100427	2.580221	0.011932
29	1	0	0.835990	2.458690	-0.782576
30	6	0	-0.779198	3.789885	-0.327835
31	1	0	-1.275199	3.655016	-1.292196
32	1	0	-0.163706	4.690361	-0.390696
33	1	0	-1.540771	3.944582	0.436749
34	6	0	0.849209	2.763234	1.335440
35	1	0	1.492665	3.644320	1.272838
36	1	0	1.475102	1.895833	1.553095
37	1	0	0.151242	2.898283	2.160605
38	7	0	-0.682979	1.327002	-0.004490
39	8	0	3.983083	-0.203244	-1.821001
40	1	0	3.814038	-1.131902	-2.026972
41	8	0	1.719652	0.433755	-1.761729
42	8	0	-2.075902	1.768527	1.800849

Monomer **4A** in chloroform at the B3LYP/6-311++G(2d,p) level

Standard orientation:

Center Number	Atomic Number	Atomic Type	Coordinates (Angstroms) X	Y	Z
1	6	0	2.561244	-0.617744	0.123227
2	6	0	3.348182	-1.044605	1.184933
3	1	0	4.373514	-0.709665	1.288079
4	6	0	2.788408	-1.918233	2.112674

5	1	0	3.377486	-2.261615	2.954373
6	6	0	1.472580	-2.358974	1.962897
7	1	0	1.051516	-3.042716	2.690121
8	6	0	0.691729	-1.931523	0.891455
9	1	0	-0.324755	-2.287423	0.784955
10	6	0	1.248115	-1.042056	-0.021594
11	6	0	0.671319	-0.422114	-1.268455
12	1	0	0.474288	-1.186530	-2.029480
13	6	0	2.937714	0.274007	-1.027330
14	1	0	3.291822	1.263844	-0.734385
15	6	0	-0.637529	0.390560	-1.130061
16	1	0	-0.752715	0.939835	-2.072037
17	6	0	-1.833569	-0.496214	-0.879851
18	6	0	-2.322894	-1.571749	-1.608778
19	1	0	-1.848627	-1.900849	-2.525862
20	6	0	-3.460585	-2.224149	-1.134929
21	1	0	-3.863602	-3.062822	-1.689946
22	6	0	-4.092050	-1.812096	0.042302
23	1	0	-4.974150	-2.337759	0.387164
24	6	0	-3.598982	-0.731811	0.767451
25	1	0	-4.079878	-0.395351	1.677829
26	6	0	-2.466215	-0.088668	0.286845
27	6	0	-1.758370	1.099834	0.827532
28	6	0	0.098727	2.581296	0.008483
29	1	0	0.830414	2.453200	-0.787729
30	6	0	-0.775091	3.792951	-0.336737
31	1	0	-1.279293	3.650429	-1.295352
32	1	0	-0.152063	4.686911	-0.413467
33	1	0	-1.529727	3.964083	0.431359
34	6	0	0.858344	2.767576	1.324760
35	1	0	1.512210	3.639318	1.246507
36	1	0	1.475699	1.895209	1.546301
37	1	0	0.170470	2.922548	2.154881
38	7	0	-0.689803	1.329811	-0.002979
39	8	0	3.969441	-0.238288	-1.840639
40	1	0	3.799899	-1.175091	-2.010400
41	8	0	1.719059	0.440732	-1.756419
42	8	0	-2.077294	1.767310	1.805683

Monomer **4A** in methanol at the B3LYP/6-311++G(2d,p) level

Standard orientation:

Center Number	Atomic Number	Atomic Type	Coordinates (Angstroms) X	Y	Z
1	6	0	2.564743	-0.612765	0.123582
2	6	0	3.354221	-1.030557	1.187085
3	1	0	4.380466	-0.696773	1.284229
4	6	0	2.795562	-1.894081	2.125189
5	1	0	3.386793	-2.230938	2.967976
6	6	0	1.478410	-2.334368	1.983148
7	1	0	1.059098	-3.012166	2.716922
8	6	0	0.694959	-1.915422	0.909885
9	1	0	-0.321439	-2.272968	0.808318
10	6	0	1.249978	-1.035139	-0.013010
11	6	0	0.670167	-0.426476	-1.263907
12	1	0	0.474928	-1.196763	-2.018543
13	6	0	2.939927	0.263086	-1.039166
14	1	0	3.309102	1.251464	-0.761266
15	6	0	-0.639362	0.385181	-1.130762
16	1	0	-0.755052	0.931875	-2.073670
17	6	0	-1.834772	-0.500959	-0.878606
18	6	0	-2.322776	-1.579212	-1.604312
19	1	0	-1.846801	-1.911527	-2.519083
20	6	0	-3.460762	-2.230067	-1.128931
21	1	0	-3.862572	-3.071218	-1.680867
22	6	0	-4.093868	-1.814280	0.046410
23	1	0	-4.975597	-2.339552	0.392550
24	6	0	-3.601895	-0.731341	0.768305
25	1	0	-4.085052	-0.394042	1.677220
26	6	0	-2.468790	-0.089198	0.286171
27	6	0	-1.760944	1.099933	0.822120
28	6	0	0.098133	2.579488	0.001773
29	1	0	0.828864	2.446170	-0.794045
30	6	0	-0.771260	3.792419	-0.348983
31	1	0	-1.278402	3.645458	-1.305162
32	1	0	-0.143513	4.682276	-0.433076
33	1	0	-1.523121	3.973523	0.419735
34	6	0	0.861228	2.768817	1.315395
35	1	0	1.519409	3.636334	1.229752
36	1	0	1.475920	1.895317	1.539725
37	1	0	0.177295	2.933235	2.147127
38	7	0	-0.694182	1.329578	-0.006021
39	8	0	3.957272	-0.273192	-1.858420
40	1	0	3.777715	-1.211876	-2.008590
41	8	0	1.716878	0.436498	-1.758177

42	8	0	-2.081169	1.769791	1.802170

Monomer **4B** in the gas phase at the B3LYP/6-311++G(2d,p) level

Standard orientation:

Center Number	Atomic Number	Atomic Type	Coordinates (Angstroms) X	Y	Z
1	6	0	-2.601403	-0.385206	0.558313
2	6	0	-3.938702	-0.033831	0.436413
3	1	0	-4.498057	0.324455	1.292547
4	6	0	-4.546189	-0.162159	-0.809959
5	1	0	-5.588828	0.105435	-0.931974
6	6	0	-3.823808	-0.639807	-1.904728
7	1	0	-4.312999	-0.740119	-2.866244
8	6	0	-2.482525	-0.992348	-1.773496
9	1	0	-1.931293	-1.367647	-2.628375
10	6	0	-1.880003	-0.850794	-0.529334
11	6	0	-0.469862	-1.135656	-0.087648
12	1	0	-0.214851	-2.188788	-0.252074
13	6	0	-1.721271	-0.366314	1.782251
14	1	0	-1.555917	0.635369	2.185535
15	6	0	0.614473	-0.282737	-0.797047
16	1	0	0.516898	-0.479436	-1.873916
17	6	0	0.546466	1.202013	-0.511853
18	6	0	-0.449529	2.132364	-0.777544
19	1	0	-1.371679	1.852827	-1.270631
20	6	0	-0.233130	3.457065	-0.397897
21	1	0	-0.997594	4.198548	-0.598502
22	6	0	0.951995	3.845201	0.230295
23	1	0	1.092252	4.881295	0.514363
24	6	0	1.952702	2.912542	0.483052
25	1	0	2.886551	3.189419	0.956732
26	6	0	1.727729	1.598663	0.101685
27	6	0	2.641880	0.431431	0.205440
28	6	0	2.554522	-1.961183	-0.554494
29	1	0	1.773938	-2.549492	-1.045431
30	6	0	3.755460	-1.900887	-1.506076
31	1	0	3.475056	-1.451384	-2.461583
32	1	0	4.126422	-2.910011	-1.700561
33	1	0	4.561312	-1.312013	-1.069247
34	6	0	2.896445	-2.641833	0.775265

35	1	0	3.243409	-3.661766	0.592469
36	1	0	2.022285	-2.684052	1.427480
37	1	0	3.683326	-2.093177	1.292155
38	7	0	1.964960	-0.629559	-0.349299
39	8	0	-2.210332	-1.104905	2.867926
40	1	0	-2.394151	-2.005553	2.569577
41	8	0	-0.470401	-0.893332	1.322174
42	8	0	3.775446	0.411219	0.657018

Monomer **4B** in chloroform at the B3LYP/6-311++G(2d,p) level

Standard orientation:

Center Number	Atomic Number	Atomic Type	Coordinates (Angstroms) X	Y	Z
1	6	0	-2.633245	-0.379710	0.556681
2	6	0	-3.969042	-0.022717	0.427461
3	1	0	-4.545092	0.299406	1.286800
4	6	0	-4.553632	-0.094263	-0.834364
5	1	0	-5.593312	0.181082	-0.963150
6	6	0	-3.811218	-0.523834	-1.936328
7	1	0	-4.282573	-0.579811	-2.910134
8	6	0	-2.472050	-0.882221	-1.797910
9	1	0	-1.905951	-1.218105	-2.658900
10	6	0	-1.891316	-0.794352	-0.538134
11	6	0	-0.489223	-1.101912	-0.087018
12	1	0	-0.251962	-2.158157	-0.254754
13	6	0	-1.780612	-0.429528	1.796404
14	1	0	-1.662209	0.534622	2.294193
15	6	0	0.614628	-0.263535	-0.777639
16	1	0	0.527909	-0.448260	-1.855935
17	6	0	0.575945	1.217346	-0.476580
18	6	0	-0.401512	2.169660	-0.732528
19	1	0	-1.328560	1.912765	-1.228202
20	6	0	-0.159281	3.486351	-0.340262
21	1	0	-0.909072	4.244442	-0.533213
22	6	0	1.033730	3.845660	0.291329
23	1	0	1.194659	4.875814	0.585264
24	6	0	2.015791	2.890802	0.535592
25	1	0	2.952618	3.151646	1.012579
26	6	0	1.765126	1.584082	0.141839
27	6	0	2.652200	0.396692	0.231256

28	6	0	2.525692	-1.978636	-0.582398
29	1	0	1.720905	-2.543637	-1.058395
30	6	0	3.688656	-1.905776	-1.578582
31	1	0	3.377744	-1.419799	-2.506065
32	1	0	4.031006	-2.914258	-1.821080
33	1	0	4.525040	-1.348326	-1.157563
34	6	0	2.906190	-2.703752	0.711896
35	1	0	3.227361	-3.722142	0.481683
36	1	0	2.055480	-2.756750	1.393477
37	1	0	3.721900	-2.188684	1.218385
38	7	0	1.957186	-0.645072	-0.320844
39	8	0	-2.267022	-1.286019	2.801503
40	1	0	-2.497724	-2.136094	2.402042
41	8	0	-0.496911	-0.857150	1.326222
42	8	0	3.794005	0.354118	0.679097

Monomer **4B** in methanol at the B3LYP/6-311++G(2d,p) level

Standard orientation:

Center Number	Atomic Number	Atomic Type	Coordinates (Angstroms) X	Y	Z
1	6	0	-2.646797	-0.372982	0.557434
2	6	0	-3.982785	-0.016382	0.427502
3	1	0	-4.564625	0.290677	1.288429
4	6	0	-4.560059	-0.067227	-0.838736
5	1	0	-5.599247	0.209266	-0.968667
6	6	0	-3.810587	-0.477108	-1.943678
7	1	0	-4.276218	-0.517036	-2.920958
8	6	0	-2.471449	-0.835588	-1.804523
9	1	0	-1.900083	-1.156110	-2.667593
10	6	0	-1.897696	-0.766859	-0.540347
11	6	0	-0.498240	-1.083237	-0.087634
12	1	0	-0.270350	-2.141389	-0.253368
13	6	0	-1.802560	-0.447643	1.800364
14	1	0	-1.704972	0.499734	2.333195
15	6	0	0.613568	-0.254119	-0.773523
16	1	0	0.529176	-0.433837	-1.851892
17	6	0	0.591382	1.224856	-0.465840
18	6	0	-0.375421	2.189117	-0.717485
19	1	0	-1.304525	1.944559	-1.215295
20	6	0	-0.118432	3.501454	-0.319716
21	1	0	-0.859418	4.268708	-0.509864

22	6	0	1.078681	3.845112	0.313497
23	1	0	1.251371	4.872211	0.611120
24	6	0	2.049935	2.878160	0.554155
25	1	0	2.988858	3.129390	1.032370
26	6	0	1.784653	1.575420	0.154885
27	6	0	2.656169	0.376977	0.239323
28	6	0	2.506647	-1.990632	-0.596913
29	1	0	1.694537	-2.538976	-1.078329
30	6	0	3.666115	-1.916082	-1.596516
31	1	0	3.356414	-1.412022	-2.514416
32	1	0	3.992729	-2.925250	-1.856332
33	1	0	4.512850	-1.377240	-1.171466
34	6	0	2.882227	-2.740154	0.684576
35	1	0	3.191186	-3.757704	0.435807
36	1	0	2.031617	-2.798138	1.365983
37	1	0	3.705182	-2.244279	1.198717
38	7	0	1.952345	-0.653762	-0.314562
39	8	0	-2.281218	-1.349901	2.772186
40	1	0	-2.522116	-2.179616	2.336598
41	8	0	-0.506926	-0.832926	1.327299
42	8	0	3.799285	0.320939	0.691384

Computed rotation barriers around C1-C1' bond for 5

Table S5 Computed energy for ketone structure **5** using geometries A' and B'.

The ketone structure **5** was used in geometries A' and B' energy computations. After conformational search and optimizations at the B3LYP/6-31G(d) level in the gas phase, the relative energy from 0-2 kcal/mol **four geometries** were selected for further optimizations at the B3LYP/6-311++G(2d,p) level in liquid. The relative energy between **5A'** and **5B'** was 0.115 kcal/mol in $CHCl_3$ using PCM model. That means the ratio of A to B was about 1:0.82 in solution chloroform. However, the ^1H NMR spectrum did not exhibited any conformer B signals. It is very clear that only one set of NMR signals.

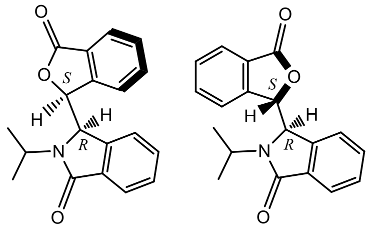 Monomer A' **5** Monomer B' **5**	Then, the transition state calculations were performed at the B3LYP/6-311++G(2d,p) in the gas phase and in solution using PCM model. The transition state barriers were 9.5-10.9 kcal/mol in the gas phase and in the $CHCl_3$. The barriers were very small too.

The relative energy between **5A'** and **5B'** were computed too at the B3LYP/6-311++G(2d,p) level in the gas phase and in solution using PCM model, respectively.

Conf.	$E_{5A'}$	$E_{5B'}$	$E_{(5A'-5B')}$
**5A'**	-1015.02217211(g)*<br>-1015.03387684(l)**		
**5B'**	-	-1015.01993994(g)<br>-1015.03369336(l)	1.401 (g)<br>0.115 (l)
Ratio of 5A':5B'	1:0.09 (g)<br>1:0.82 (l)		

	E.	TS barrier
TS	-1015.00475546 (g)*<br>-1015.01769915 (l)**	
From 5B' to TS to 5A':		10.9 (g)<br>10.1 (l) kcal/mol
From 5A' to TS to 5B'		9.53 (g)<br>10.04 (l) kcal/mol
* the letter "g" means the datum is obtained in the gas phase. ** The letter "l" means the datum was obtained in liquid.		

Computed rotatory barriers around C3-C1’ for compound 9.

Table S6 Investigation of the rotatory barrier around the C3-C1’ of compound **9**.

Potential energy scan (PES) computations were performed at the B3LYP/6-311++G(2d,p) level in the gas phase. Total 71 steps (355°) of computations were performed.

Entry			
1	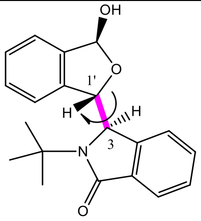 Compound **8**	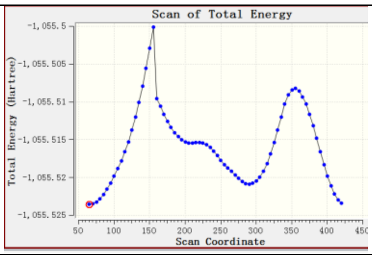	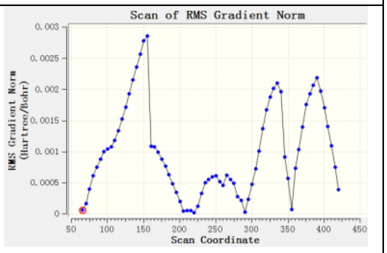
2	The highest energy conformer:		-1055.500169 a.u.
3	The lowest energy conformer:		-1055.523598 a.u.
4	The energy differences is:		14.701 kcal/mol

Crystal data for crystal 15-17 and 19 and computed bond length for 15-17.

Crystal data for compound 15

Crystal **15**'s data: $C_{16}H_{20}O_3N_2$;

M_W = 288.35;

dimensions 0.10×0.20×0.30 mm;

monoclinic system,

space group $P2_1$, a = 9.033 (2) Å,

b = 11.605 (2) Å,

c = 15.369 (2) Å,

β = 106.24 (3)°.

V = 1546.8 (5) Å^3,

Z = 2,

d = 1.238g/cm^3,

T = 295K.

Crystal was used for measurements on a MAC DIP-2030K diffractometer with a graphite monochromator (ω scans, θ_{max} = 25.00°), Mo Kα radiation. The total number of independent reflections measured was 3203, of which 2586 were observed ($|F|^2 \geq 2\delta|F|^2$). Final indices: R_f = 0.0570, wR_2 = 0.1610, S = 1.210, $(\Delta/\sigma)_{max}$ = 0.043, $(\Delta/\rho)_{min}$ = –0.180 e/Å, $(\Delta/\rho)_{max}$ = 0.246 e/Å. The crystal structure of **15** was solved by direct methods using SHELXS-97 (Sheldrich, G. M. University of Gottingen: Gottingen, Germany, 1997) and expanded using difference Fourier techniques, refined by the program and method SHELXL-97 (Sheldrich, G. M. University of Gottingen: Gottingen, Germany, 1997) and the full-matrix least-squares calculations. All the H atoms were placed in geometrically idealized positions and constrained to ride on their parent atoms. The absolute configuration of this compound was not determined.

Table S7 Atomic coordinates ($\times10^4$) and equivalent isotropic displacement parameters ($Å^2\times10^3$) for d4591 (**15**). U(eq) is defined as one third of the trace of the orthogonalized Uij tensor.

	x	y	z	U(eq)
O(1)	6908(8)	2638(8)	3837(6)	97(2)
O(2)	7985(7)	4246(8)	4490(4)	93(2)
O(3)	13225(8)	4647(6)	3088(4)	79(2)
C(1)	12149(9)	3595(7)	4137(5)	60(2)
N(2)	10840(7)	3547(7)	4525(4)	68(2)
C(3)	9492(9)	3003(9)	3860(5)	68(2)
C(4)	9719(9)	1754(9)	3713(6)	71(2)
C(5)	11273(9)	1582(8)	3547(5)	66(2)
C(6)	11979(10)	626(7)	3267(5)	67(2)
C(7)	11494(13)	-543(10)	3003(6)	85(3)
C(8)	12540(14)	-1271(10)	2813(7)	90(3)
C(9)	14023(17)	-953(8)	2891(8)	98(4)
C(10)	14529(13)	116(10)	3105(6)	83(3)
C(11)	13521(11)	935(8)	3312(5)	68(2)
N(12)	13710(8)	2033(6)	3587(4)	63(2)
C(13)	12382(9)	2461(8)	3745(5)	64(2)
C(14)	7984(9)	3249(9)	4067(6)	72(2)
C(15)	12027(10)	4644(8)	3517(6)	70(2)
C(16)	12070(20)	5761(10)	4015(11)	91(5)
C(17)	6534(12)	4605(15)	4641(7)	93(4)
C(18)	11166(11)	3044(10)	5409(6)	79(2)
O(1')	7708(11)	-279(7)	89(7)	90(3)
O(2')	6362(9)	187(7)	1036(5)	93(2)
O(3')	12641(7)	3395(6)	1461(4)	79(2)
C(1')	10083(10)	2864(7)	563(5)	64(2)
N(2')	9006(7)	1896(6)	224(4)	62(2)
C(3')	8285(9)	1492(8)	948(6)	66(2)
C(4')	7302(9)	2402(7)	1241(5)	64(2)
C(5')	8051(9)	3556(9)	1290(5)	66(2)
C(6')	7632(10)	4596(6)	1637(5)	62(2)
C(7')	6548(13)	4942(9)	2084(7)	83(3)
C(8')	6474(16)	6062(11)	2355(7)	97(3)
C(9')	7580(13)	6874(8)	2226(7)	84(3)
C(10')	8668(12)	6599(8)	1800(6)	75(2)
C(11')	8742(10)	5449(8)	1518(5)	71(2)
N(12')	9711(9)	4892(6)	1118(5)	73(2)
C(13')	9319(10)	3748(7)	987(5)	65(2)
C(14')	7451(10)	369(7)	622(6)	68(2)

C(15')	11651(10)	2434(8)	1214(4)	65(2)
C(16')	12400(11)	1491(9)	814(7)	78(2)
C(17')	5410(13)	-801(10)	761(10)	90(3)
C(18')	7844(11)	2200(8)	-619(6)	76(2)
H(3A)	12958	4272	2618	119
H(1A)	13065	3723	4649	72
H(3B)	9416	3378	3278	81
H(4A)	8911	1480	3196	85
H(4B)	9666	1318	4242	85
H(7A)	10498	-789	2965	102
H(8A)	12231	-2015	2622	109
H(9A)	14706	-1503	2791	117
H(10A)	15531	321	3118	100
H(12A)	14546	2421	3655	75
H(15A)	11040	4600	3046	85
H(16A)	12012	6392	3603	182
H(16B)	13019	5811	4493	182
H(16C)	11216	5796	4269	182
H(17A)	6664	5344	4933	155
H(17B)	6224	4051	5019	155
H(17C)	5757	4657	4070	155
H(18A)	12094	3374	5793	119
H(18B)	11297	2227	5365	119
H(18C)	10325	3193	5661	119
H(3'A)	12674	3596	1977	118
H(1'A)	10312	3222	38	76
H(3'B)	9136	1306	1483	80
H(4'A)	6286	2427	811	77
H(4'B)	7182	2201	1831	77
H(7'A)	5866	4401	2199	100
H(8'A)	5712	6290	2619	116
H(9'A)	7558	7620	2442	101
H(10B)	9343	7154	1697	90
H(12B)	10464	5212	969	87
H(15B)	11445	2133	1764	78
H(16D)	13350	1267	1244	118
H(16E)	12608	1765	271	118
H(16F)	11721	838	674	118
H(17D)	4673	-845	1105	149
H(17E)	6039	-1482	865	149
H(17F)	4878	-741	128	149
H(18D)	8346	2430	-1065	114
H(18E)	7225	2825	-507	114
H(18F)	7198	1545	-837	114

Crystal data for 16

Crystal data and structure refinement.

Identification code	1
Empirical formula	$C_{16}H_{21}N_2O_{3.50}$
Formula weight	297.35
Temperature	293(2) K
Wavelength	0.71073 A
Crystal system, space group	Monoclinic, P2(1)
Unit cell dimensions	a = 7.5749(9) A alpha = 90 deg.
	b = 12.7761(15) A beta = 102.738(2) deg.
	c = 16.839(2) A gamma = 90 deg.
Volume	1589.5(3) A^3
Z, Calculated density	4, 1.243 Mg/m^3
Absorption coefficient	0.088 mm^-1
F(000)	636
Crystal size	0.35 x 0.30 x 0.14 mm
Theta range for data collection	2.02 to 28.29 deg.
Limiting indices	-9<=h<=6, -16<=k<=17, -22<=l<=21
Reflections collected / unique	10278 / 6543 [R(int) = 0.0243]
Completeness to theta = 27.50	98.5 %
Absorption correction	Muti-scan
Max. and min. transmission	1.0000 and 0.2486
Refinement method	Full-matrix least-squares on F^2
Data / restraints / parameters	6543 / 1 / 383
Goodness-of-fit on F^2	1.011
Final R indices [I>2sigma(I)]	R1 = 0.0536, wR2 = 0.1165
R indices (all data)	R1 = 0.0921, wR2 = 0.1372
Absolute structure parameter	0.0(12)
Largest diff. peak and hole	0.163 and -0.251 e.A^-3

Table S8. Atomic coordinates ($\times10^4$) and equivalent isotropic is placement parameters (Å$^2\times10^3$) for **16**. U(eq) is defined as one third of the trace of the orthogonalized Uij tensor.

	x	y	z	U(eq)
O(1)	11712(4)	-3456(2)	7842(2)	88(1)
O(1W)	176(3)	4592(2)	7131(1)	62(1)
O(2)	7862(4)	-4773(2)	5673(1)	68(1)
O(3)	7313(3)	-3814(2)	4546(1)	57(1)
O(4)	3399(3)	933(2)	9755(1)	62(1)
O(5)	2172(4)	772(2)	8439(2)	82(1)
O(6)	3115(3)	3270(2)	7065(2)	73(1)

N(1)	9741(3)	-158(2)	7014(1)	47(1)
N(2)	8510(3)	-2973(2)	6629(1)	42(1)
N(3)	9108(3)	3601(2)	8476(1)	45(1)
N(4)	5080(3)	1825(2)	8034(1)	41(1)
C(1)	9093(4)	607(2)	6443(2)	45(1)
C(2)	9147(4)	1697(3)	6516(2)	55(1)
C(3)	8318(5)	2263(3)	5839(2)	63(1)
C(4)	7476(4)	1774(3)	5110(2)	60(1)
C(5)	7444(4)	697(3)	5034(2)	53(1)
C(6)	8270(4)	89(2)	5716(2)	44(1)
C(7)	8460(4)	-1016(2)	5870(2)	44(1)
C(8)	7778(4)	-1932(2)	5324(2)	48(1)
C(9)	8582(4)	-2922(2)	5761(2)	42(1)
C(10)	9671(4)	-2158(2)	7104(2)	43(1)
C(11)	9358(4)	-1140(2)	6661(2)	42(1)
C(12)	11643(4)	-2555(3)	7339(2)	57(1)
C(13)	12904(5)	-1767(3)	7824(2)	74(1)
C(14)	6661(4)	-2904(3)	6751(2)	54(1)
C(15)	7861(4)	-3934(3)	5339(2)	47(1)
C(16)	6771(5)	-4760(3)	4081(2)	68(1)
C(17)	10334(4)	3568(2)	9214(2)	42(1)
C(18)	12003(4)	4061(2)	9467(2)	52(1)
C(19)	12937(4)	3871(3)	10259(2)	61(1)
C(20)	12215(4)	3244(3)	10784(2)	61(1)
C(21)	10562(4)	2770(2)	10536(2)	52(1)
C(22)	9589(4)	2926(2)	9739(2)	40(1)
C(23)	7860(4)	2579(2)	9292(2)	39(1)
C(24)	6485(4)	1890(2)	9545(2)	44(1)
C(25)	4734(4)	1908(2)	8868(2)	40(1)
C(26)	6024(4)	2770(2)	7843(2)	41(1)
C(27)	7614(4)	3006(2)	8528(2)	41(1)
C(28)	4649(4)	3670(2)	7628(2)	49(1)
C(29)	5367(5)	4601(3)	7246(2)	78(1)
C(30)	6075(5)	865(2)	7933(2)	56(1)
C(31)	3305(4)	1133(2)	8980(2)	46(1)
C(32)	2008(5)	272(3)	9948(2)	80(1)

Crystal for compound 17

$C_{21}H_{22}O_3N_2$; M_W = 350.42; dimensions 0.20×0.20×0.30 mm; monoclinic system, space group $P2_12_12_1$, a = 9.548 (2) Å, b = 10.155 (2) Å, c = 18.995 (4) Å. V = 1841.8 (6) Å^3, Z = 4, d = 1.264g/cm^3, T = 295K. Crystal was used for measurements on a MAC DIP-2030K diffractometer with a graphite monochromator (ω scans, θ_{max} = 25.00°), Mo Kα radiation. The total number of independent reflections measured was 1839, of which 1820 were observed ($|F|^2 \geq 2\delta|F|^2$). Final indices: R_f = 0.0750, wR_2 = 0.1720, S = 1.410, $(\Delta/\sigma)_{max}$ = 0.001, $(\Delta/\rho)_{min}$ = –0.216 e/Å, $(\Delta/\rho)_{max}$ = 0.227 e/Å. The crystal structure of **1** was solved by direct methods using SHELXS-97 (Sheldrich, G. M. University of Gottingen: Gottingen, Germany, 1997) and expanded using difference Fourier techniques, refined by the program and method SHELXL-97 (Sheldrich, G. M. University of Gottingen: Gottingen, Germany, 1997) and the full-matrix least-squares calculations. All the H atoms were placed in geometrically idealized positions and constrained to ride on their parent atoms. The absolute configuration of this compound was not determined.

The original .cif missed. However, it was deposited in CCDC (CCDC 687117). *The coordinate for the crystal is listed below (Gaussian09 file format)*

Table S9. The coordinate for the crystal **17**

-- ---
-

Center Number	Atomic Number	Atomic Type	Coordinates (Angstroms) X	Y	Z
1	8	0	2.516256	-1.458784	1.602059
2	8	0	4.194913	-0.389194	0.585370
3	8	0	-1.257557	-1.752902	-2.162534
4	6	0	0.006131	-1.424050	-0.114546
5	7	0	1.190734	-1.912984	-0.871936
6	6	0	2.410861	-1.098871	-0.803420
7	6	0	2.168567	0.283635	-1.407705
8	6	0	0.958597	0.868527	-0.758488
9	6	0	0.578652	2.230178	-0.546875
10	6	0	1.146638	3.476135	-0.857765
11	6	0	0.517013	4.621595	-0.448881
12	6	0	-0.671506	4.577529	0.279939
13	6	0	-1.244973	3.381553	0.616845
14	6	0	-0.620091	2.204104	0.209761
15	7	0	-0.925287	0.885396	0.443024
16	6	0	0.021822	0.079479	-0.161201
17	6	0	3.017518	-1.007801	0.608820
18	6	0	-1.248626	-2.042122	-0.769147

19	6	0	1.452632	-3.347805	-0.634501
20	6	0	4.916708	-0.300590	1.838869
21	6	0	-2.523424	-1.529481	-0.137537
22	6	0	-3.378136	-0.645895	-0.788741
23	6	0	-4.434799	-0.058393	-0.125848
24	6	0	-4.675656	-0.374726	1.189744
25	6	0	-3.880684	-1.293877	1.832578
26	6	0	-2.821177	-1.874076	1.178085
27	1	0	-0.485908	-1.722633	-2.440126
28	1	0	0.069164	-1.722616	0.816525
29	1	0	3.078545	-1.541150	-1.369923
30	1	0	2.936991	0.855413	-1.253868
31	1	0	2.029360	0.209883	-2.364971
32	1	0	1.941722	3.522061	-1.338088
33	1	0	0.889331	5.446284	-0.662472
34	1	0	-1.079496	5.371476	0.541561
35	1	0	-2.034087	3.354473	1.107808
36	1	0	-1.600197	0.603741	0.895238
37	1	0	-1.213549	-3.016134	-0.652950
38	1	0	2.218743	-3.622529	-1.144545
39	1	0	0.688598	-3.862168	-0.906407
40	1	0	1.621067	-3.492703	0.299649
41	1	0	5.747767	0.157729	1.698732
42	1	0	5.090715	-1.184099	2.171704
43	1	0	4.389217	0.183853	2.479095
44	1	0	-3.235211	-0.447224	-1.686267
45	1	0	-4.982926	0.550013	-0.567323
46	1	0	-5.375859	0.032556	1.645312
47	1	0	-4.060786	-1.524080	2.716776
48	1	0	-2.296610	-2.502281	1.617848

Bond lengths in crystals 15, 16 (conformations A and B) and 17

Table S10. Bond lengths in crystals **15, 16** (conformations A and B) and in **17**

Entry	Bond	Bond length (r) (Å)) **15**A / **15**B / **17**	delta r (Å), (**15**A-**17**) / (**15**B-**17**) / (**15A-15B**) [a]
1	C1-N2	1.467 / 1.482 / 1.489	-0.022 / -0.007 / -0.015
2	N2-C3	1.493 / 1.513 / 1.468	0.025 / 0.045 / -0.020
3	C3-C4	1.489 / 1.526 / 1.528	-0.039 / -0.002 / -0.037
4	C4-C4'	1.509 / 1.492 / 1.493	0.016 / -0.001 / 0.017
5	C4'-C5'	1.406 / 1.414 / 1.429	-0.023 / -0.015 / -0.008
6	C5'-C5	1.448 / 1.402 / 1.404	0.044 / 0.002 / 0.046
7	C5-C6	1.358 / 1.372 / 1.370	-0.012 / 0.002 / -0.014
8	C6-C7	1.362 / 1.426 / 1.395	-0.033 / 0.031 / -0.064
9	C7-C8	1.331 / 1.363 / 1.368	-0.037 / -0.005 / -0.032
10	C8-C8'	1.412 / 1.410 / 1.394	0.018 / 0.016 / 0.002
11	C8'-N9	1.338 / 1.365 / 1.374	-0.036 / -0.009 / -0.027
12	N9-C9'	1.382 / 1.374 / 1.383	-0.001 / -0.009 /0.008
13	C9'-C1	1.486 / 1.484 / 1.504	-0.018 / -0.020 / 0.002
14	C1-C1'	1.530 / 1.570 / 1.544	-0.014 / 0.026 / -0.040
15	C1'-C2'	1.501 / 1.505 / -	- / - / -0.004 [b]
16	C1'-O10	1.416 / 1.413 / 1.423	-0.007 / -0.010 / 0.003
17	N2-C1''	1.431 / 1.464 / 1.478	-0.046 / -0.014 / -0.033
18	C3-C1'''	1.510 / 1.518 / 1.540	-0.030 / -0.022 / -0.008
19	C1'''-O11	1.175 / 1.182 / 1.200	-0.025 / -0.018 / -0.007
20	C1'''-O12	1.327 / 1.329 / 1.330	-0.003 / -0.001 / -0.002
21	O12-C2'''	1.455 / 1.425 / 1.449	0.006 / 0.024 / 0.030
22	C4'-C9'	1.402 / 1.370 / 1.363	0.039 / 0.007 / 0.032
23	C5'-C8'	1.422 / 1.457 / 1.418	0.004 / 0.039 / -0.035

Entry	Bond	Bond length (r, Å), **16**A / **16**B / **17**	Delta r (Å), (**16**A-**17**) / (**16**B-**17**) / (**16**A-**16**B) [a]
1	C1-N2	1.474 / 1.479 / 1.489	-0.015 / -0.010 / 0.005
2	N2-C3	1.488 / 1.476 / 1.464	0.024 / 0.013 / 0.012
3	C3-C4	1.548 / 1.522 / 1.528	0.020 / -0.006 /*0.026*
4	C4-C4'	1.495 / 1.507 / 1.493	0.003 / 0.014 / -0.012
5	C4'-C5'	1.429 / 1.437 / 1.429	0.000 / 0.008 / -0.008
6	C5'-C5	1.395 / 1.412 / 1.404	-0.009 / 0.008 / -0.018
7	C5-C6	1.371 / 1.382 / 1.370	0.001 / 0.012 / -0.011
8	C6-C7	1.391 / 1.399 / 1.395	-0.004 / 0.004 / -0.008
9	C7-C8	1.386 / 1.379 / 1.368	0.019 / 0.011 / 0.008
10	C8-C8'	1.392 / 1.397 / 1.394	-0.002 / 0.003 / -0.005
11	C8'-N9	1.377 / 1.383 / 1.374	0.003 / 0.009 / -0.005
12	N9-C9'	1.382 / 1.392 / 1.383	-0.001 / 0.009 / -0.010
13	C9'-C1	1.502 / 1.493 / 1.504	-0.002 / -0.012 / 0.010
14	C1-C1'	1.540 / 1.545 / 1.544	-0.004 / 0.001 / -0.004
15	C1'-C2'	1.510 / 1.499 / -	- / - / 0.010 [b]
16	C1'-O10	1.423 / 1.423 / 1.423	0/0/0
17	N2-C1''	1.469 / 1.463 / 1.478	-0.009 / -0.016 / 0.007
18	C3-C1'''	1.509 / 1.518 /1.540	-0.031 /-0.022 / -0.009
19	C1'''-O11	1.197 / 1.210 / 1.200	-0.003 / 0.010 / -0.013
20	C1'''-O12	1.316 / 1.316 / 1.330	-0.014 /-0.014 / -0.001
21	O12-C2'''	1.442 / 1.449 / 1.449	-0.007 / 0 / -0.007
22	C4'-C9'	0.820 / 0.822 / 0.821	-0.001 / 0.001 / -0.001
23	C5'-C8'	1.374 / 1.365 / 1.363	0.011 / 0.002 / 0.009

[a] (**15A-17**) means, for example, the C1-N2 bond length in **15A** was substrate from the corresponding bond length in **17**. [b] The C1' in **17** is sp^2 hybridization, and that in A or B in **15** and **16** is sp^3 hybridization. No needs to compare to bond length with different hybridization atoms.

Predicted ^{13}C NMR for 15, 16 and 17

Table S11. The calculated ^{13}C NMR for conformations A and B for crystal **15** and **16** using B3LYP/6-311+(2d,p) method.

C-No.	σ [a]		δ [b]		σ [a]		δ [b]	
	**15**A	**15**B	**15**A	**15**B	**16**A	**16**B	**16**A	**16**B
1	122.8	121.1	70.1	71.6	122.9	119.1	70.0	73.3
3	130.5	128.8	63.5	64.9	130.4	130.0	63.6	63.9
4	178.3	175.9	22.1	24.2	175.6	176.7	24.5	23.5
4'	62.8	71.2	122.0	114.8	66,5	66.7	118.8	118.7
5'	46.9	47.1	135.8	135.6	49.5	48.5	133.5	134.4
5	64.8	67.4	120.3	118.0	68.9	65.6	116.8	119.6
6	71.9	66.7	114.2	118.7	67.8	66.1	117.7	119.2
7	73.6	64.2	112.7	120.8	65.0	64.1	120.1	120.9
8	81.6	77.8	105.8	109.1	76.6	76.8	110.1	109.9
8'	45.5	38.4	137.0	143.1	42.0	40.7	140.0	141.1
9'	43.4	47.4	138.8	135.3	45.7	46.7	136.8	136.0
1'	120.1	116.5	72.5	75.6	121.0	120.2	71.7	72.4
2'	186.3	186.8	15.2	14.8	184.9	188.6	16.4	13.2
2''	171	168.4	28.4	30.7	169.3	168.6	29.9	30.5
1'''	11.5	11	**166.4**	**166.8**	9.0	4.8	**168.6**	**172.2**
2'''	147	149.9	49.2	46.7	147.9	146.2	48.4	49.9

[a] Magnetic shielding values. [b] Correction function obtained in the crystal **17** NMR computations was used in the correction: $\delta = 176.35 - 0.865 \times \sigma$.

Table S12. Calculated ^{13}C NMR for crystal **17** at the B3LYP/6-311+G(2d,p) level using the coordinate of X-ray structure.

Atom No.	Experimental δ	Calculated σ [a]	Corrected δ	δ (ppm) [b]
C1	65.4	122.8	70.0	-4.6
C3	64.1	124.0	69.1	-5.0
C4	25.5	168.3	30.6	-5.1
C4'	109.1	69.3	116.4	-7.3
C5'	127.8	50.2	132.8	-5.0
C5	120.2	69.0	116.6	3.6
C6	123	69.2	116.5	6.5
C7	116.5	67.5	117.9	-1.4
C8	113.1	78.1	108.7	4.4
C8'	136.3	43.0	139.1	-2.8
C9'	135.6	47.8	134.9	0.7
C1'	68.7	116.4	75.6	-6.9
C2a'	141.3	34.1	146.8	-5.5
C3a'	131.6	56.1	127.8	3.8
C4a'	129.6	59.0	125.3	4.3
C5a'	126.7	61.5	123.1	3.6
C6a'	127.8	60.8	123.7	4.1
C7a'	124.7	62.6	122.2	2.5
C1''	41.8	165.4	33.2	8.6
C1'''	171.7	3.9	173.1	-1.4
C2'''	51.5	147.5	48.7	2.8

[a] Magnetic shielding values. [b] $\Delta\delta = \delta_{exp} - \delta_{calcd}$. The function was used in correction of crystal **7** NMR data: $\delta = 176.35 - 0.865 \times \sigma$.

Crystal for compound 19

Identification code	080604b
Empirical formula	C17 H24 N2 O2
Formula weight	288.38
Temperature	298(2) K
Wavelength	0.71073 A
Crystal system, space group	Orthorhombic, P2(1)2(1)2
Unit cell dimensions	a = 27.376(4) A alpha = 90 deg.
	b = 12.1401(19) A beta = 90 deg.
	c = 9.8049(16) A gamma = 90 deg.
Volume	3258.6(9) A^3
Z, Calculated density	8, 1.176 Mg/m^3
Absorption coefficient	0.077 mm^-1
F(000)	1248
Crystal size	0.15 x 0.10 x 0.08 mm
Theta range for data collection	1.49 to 28.39 deg.
Limiting indices	-36<=h<=36, -15<=k<=7, -12<=l<=12
Reflections collected / unique	21507 / 7749 [R(int) = 0.1173]
Completeness to theta = 28.39	98.3 %
Absorption correction	Mulit-scan
Max. and min. transmission	0.9938 and 0.9885
Refinement method	Full-matrix least-squares on F^2
Data / restraints / parameters	7749 / 0 / 392
Goodness-of-fit on F^2	0.851
Final R indices [I>2sigma(I)]	R1 = 0.0662, wR2 = 0.1454
R indices (all data)	R1 = 0.2304, wR2 = 0.2286
Absolute structure parameter	1(3)
Extinction coefficient	0.0074(12)
Largest diff. peak and hole	0.177 and -0.205 e.A^-3

Table S13. Atomic coordinates (×10^4) and equivalent isotropic displacement parameters (A^2× 10^3) for 080604B(**19**) U(eq) is defined as one third of the trace of the orthogonalized Uij tensor.

	x	y	z	U(eq)
N(1)	8090(2)	3492(4)	-769(4)	63(1)
N(2)	8945(1)	3843(3)	2202(4)	51(1)
N(3)	2926(1)	9370(4)	4025(4)	58(1)
N(4)	4165(1)	8433(4)	2953(4)	64(1)
O(1)	8193(1)	4131(4)	3880(3)	75(1)
O(2)	9978(1)	2727(4)	2535(3)	77(1)
O(3)	4546(2)	8072(3)	76(4)	84(1)
O(4)	4300(1)	7413(5)	5315(4)	102(2)
C(1A)	9097(2)	2671(4)	2193(5)	52(1)
C(2A)	9177(2)	2228(5)	745(5)	59(1)
C(3A)	8762(2)	2572(5)	-149(5)	53(1)
C(4A)	8612(2)	2194(5)	-1459(5)	54(1)
C(5A)	8786(2)	1384(5)	-2358(5)	66(2)
C(6A)	8534(2)	1189(5)	-3552(6)	74(2)
C(7A)	8125(2)	1804(6)	-3894(6)	84(2)
C(8A)	7948(2)	2603(5)	-3031(6)	73(2)
C(9A)	8188(2)	2786(5)	-1820(5)	59(1)
C(1B)	8436(2)	3364(4)	246(5)	53(1)
C(2B)	8454(2)	3983(4)	1563(5)	53(1)
C(3B)	8060(2)	3656(5)	2603(5)	61(2)
C(4B)	7554(2)	4043(6)	2261(6)	86(2)
C(5B)	9304(2)	4591(5)	1592(6)	75(2)
C(1D)	9524(2)	2415(5)	3174(5)	58(2)
C(2D)	9540(2)	1197(6)	3468(6)	89(2)
C(3D)	9464(2)	3055(5)	4498(5)	77(2)
C(3)	3793(2)	8605(5)	4014(5)	61(2)
C(6)	3191(2)	8625(4)	2069(5)	54(1)
C(7)	3984(2)	7666(5)	1886(5)	61(2)
C(9)	2707(2)	9021(4)	1841(5)	55(1)
C(11)	2552(2)	9503(5)	3085(5)	55(1)
C(14)	2388(2)	9057(5)	726(6)	65(2)
C(15)	1946(2)	9554(5)	865(7)	75(2)
C(16)	3541(2)	8104(5)	1098(5)	61(2)
C(18)	3311(2)	8860(4)	3373(5)	53(1)
C(20)	4393(2)	7192(5)	984(5)	66(2)
C(22)	4825(2)	6810(7)	1833(7)	100(2)
C(23)	4334(2)	9507(6)	2413(6)	89(2)

C(25)	4209(2)	6259(5)	116(7)	89(2)
C(26)	1800(2)	10023(5)	2092(7)	75(2)
C(27)	3808(2)	7662(5)	5037(5)	69(2)
C(28)	3551(2)	7930(6)	6353(5)	86(2)
C(33)	2096(2)	10000(5)	3221(6)	67(2)

Table S14 The energy of the two kinds of dimers and their coordinates:

	E of dimer of two (*S,R,R*)-**4**	Dimer of (*S,R,R*)-4 and (*R,S,S*)-**4**
Energy （a.u.）	2032.3202332	2032.3223449
Energy difference	1.325 kcal/mol	

Dimer of two same (*S,R,R*)-**4**:

HF=-2032.3202332

Standard orientation:

Center Atomic Atomic Coordinates (Angstroms)

Number Number Type X Y Z

----

1 6 0 -4.302036 -1.692413 -0.015331

2 6 0 -5.225007 -2.619067 0.455149

3 1 0 -4.895404 -3.555157 0.893562

4 6 0 -6.583852 -2.325735 0.335623

5 1 0 -7.322903 -3.033253 0.697512

6 6 0 -7.003289 -1.132660 -0.261347

7 1 0 -8.063946 -0.923288 -0.356893

8 6 0 -6.070693 -0.212774 -0.743388

9 1 0 -6.406378 0.704621 -1.218303

10 6 0 -4.716622 -0.504814 -0.602625

11 6 0 -3.497358 0.275798 -1.025021

12 1 0 -3.470328 0.378631 -2.118130

13 6 0 -2.797623 -1.778150 -0.040552

14 1 0 -2.340296 -1.832535 0.954039

15 6 0 -3.397357 1.700284 -0.416293

16 1 0 -4.296919 2.254077 -0.718648

17 6 0 -3.211695 1.730430 1.084112

18 6 0 -4.037256 1.291721 2.113538

19 1 0 -5.002787 0.841026 1.914942

20 6 0 -3.591828 1.447730 3.428993

21 1 0 -4.223157 1.114174 4.246499

22 6 0 -2.350387 2.028056 3.711289

23 1 0 -2.031409 2.136834 4.742790

24 6 0 -1.528040 2.471510 2.677216

25 1 0 -0.566351 2.933610 2.872165

26 6 0 -1.980981 2.313402 1.373056

27 6 0 -1.333709 2.730982 0.105500

28 6 0 -2.009539 2.743375 -2.322688

29 1 0 -2.936752 2.424999 -2.810038

30 6 0 -1.875177 4.256747 -2.534167

31 1 0 -2.735379 4.790618 -2.120687

32 1 0 -1.822601 4.477983 -3.603750
33 1 0 -0.972360 4.641543 -2.060115
34 6 0 -0.852844 1.954069 -2.946801
35 1 0 -0.827173 2.127574 -4.026797
36 1 0 -0.970251 0.882970 -2.772054
37 1 0 0.108107 2.259860 -2.532490
38 7 0 -2.203352 2.414236 -0.897606
39 8 0 -2.384575 -2.854861 -0.814338
40 1 0 -1.454174 -3.058453 -0.599936
41 8 0 -2.387086 -0.509094 -0.602263
42 8 0 -0.242172 3.289067 -0.016959
43 6 0 4.302115 1.692399 -0.016371
44 6 0 5.224832 2.619667 0.453400
45 1 0 4.894983 3.556248 0.890575
46 6 0 6.583745 2.326290 0.334763
47 1 0 7.322601 3.034298 0.696094
48 6 0 7.003509 1.132527 -0.260603
49 1 0 8.064220 0.923117 -0.355470
50 6 0 6.071172 0.212008 -0.741928
51 1 0 6.407106 -0.705929 -1.215621
52 6 0 4.717024 0.504144 -0.602098
53 6 0 3.498008 -0.277041 -1.024124
54 1 0 3.471656 -0.381494 -2.117094
55 6 0 2.797705 1.777962 -0.042149
56 1 0 2.340116 1.832883 0.952296
57 6 0 3.397667 -1.700658 -0.413361
58 1 0 4.297635 -2.254734 -0.713984
59 6 0 3.210541 -1.728569 1.086906
60 6 0 4.035047 -1.288203 2.116468
61 1 0 5.000734 -0.837725 1.918139
62 6 0 3.588353 -1.442260 3.431724
63 1 0 4.218851 -1.107398 4.249337
64 6 0 2.346709 -2.022309 3.713693
65 1 0 2.026751 -2.129578 4.745048
66 6 0 1.525428 -2.467440 2.679495
67 1 0 0.563623 -2.929394 2.874219
68 6 0 1.979621 -2.311267 1.375538
69 6 0 1.333711 -2.730979 0.107988
70 6 0 2.012041 -2.747246 -2.319441
71 1 0 2.939563 -2.429232 -2.806435
72 6 0 1.878636 -4.261052 -2.528385
73 1 0 2.738647 -4.793778 -2.113035
74 1 0 1.827324 -4.484225 -3.597629
75 1 0 0.975488 -4.645427 -2.054617

76 6 0 0.855529 -1.959654 -2.946047
77 1 0 0.830813 -2.135266 -4.025724
78 1 0 0.972336 -0.888161 -2.773308
79 1 0 -0.105612 -2.265086 -2.531902
80 7 0 2.204266 -2.415554 -0.894745
81 8 0 2.384682 2.854138 -0.816655
82 8 0 2.387480 0.508510 -0.603286
83 8 0 0.242466 -3.289565 -0.014761
84 1 0 1.454265 3.057848 -0.602400

Dimer of (*S*,*R*,*R*)-**4** and (*R*,*S*,*S*)-**4**
HF=-2032.3223449
Standard orientation:

Center Atomic Atomic Coordinates (Angstroms)
Number Number Type X Y Z
----
1 6 0 4.308294 -1.526773 0.476671
2 6 0 5.228496 -2.529706 0.195560
3 1 0 4.897711 -3.544854 0.003085
4 6 0 6.585366 -2.204678 0.184230
5 1 0 7.322515 -2.971484 -0.031225
6 6 0 7.006154 -0.900269 0.462234
7 1 0 8.065716 -0.665121 0.459455
8 6 0 6.076480 0.099411 0.753897
9 1 0 6.413472 1.106357 0.983012
10 6 0 4.723915 -0.231005 0.749933
11 6 0 3.508649 0.616011 1.031827
12 1 0 3.550003 1.018446 2.052487
13 6 0 2.806388 -1.615336 0.560561
14 1 0 2.333500 -1.822651 -0.406573
15 6 0 3.319919 1.817639 0.062312
16 1 0 4.219127 2.445372 0.129091
17 6 0 3.023027 1.426225 -1.367876
18 6 0 3.792404 0.738209 -2.299560
19 1 0 4.791480 0.389677 -2.064328
20 6 0 3.243494 0.503129 -3.563119
21 1 0 3.829299 -0.029066 -4.305936
22 6 0 1.955083 0.941164 -3.888066
23 1 0 1.554068 0.741004 -4.876230
24 6 0 1.188048 1.635824 -2.954510
25 1 0 0.188273 1.984178 -3.189684
26 6 0 1.745443 1.869774 -1.702580

27 6 0 1.183187 2.619695 -0.553932
28 6 0 2.017136 3.319789 1.710091
29 1 0 2.961370 3.123375 2.227509
30 6 0 1.905732 4.836480 1.509187
31 1 0 2.748497 5.219434 0.927114
32 1 0 1.908331 5.342177 2.478960
33 1 0 0.982886 5.095100 0.989353
34 6 0 0.879009 2.753054 2.567089
35 1 0 0.902909 3.204020 3.563527
36 1 0 0.969831 1.670352 2.674820
37 1 0 -0.089392 2.970954 2.115889
38 7 0 2.137666 2.616386 0.420184
39 8 0 2.401697 -2.555220 1.493866
40 1 0 1.489583 -2.833597 1.272390
41 8 0 2.401319 -0.272509 0.935445
42 8 0 0.085630 3.178273 -0.480491
43 6 0 -4.308294 1.526770 -0.476597
44 6 0 -5.228482 2.529699 -0.195429
45 1 0 -4.897686 3.544841 -0.002939
46 6 0 -6.585353 2.204677 -0.184062
47 1 0 -7.322491 2.971481 0.031437
48 6 0 -7.006154 0.900276 -0.462084
49 1 0 -8.065717 0.665133 -0.459276
50 6 0 -6.076494 -0.099400 -0.753802
51 1 0 -6.413499 -1.106339 -0.982931
52 6 0 -4.723928 0.231011 -0.749881
53 6 0 -3.508676 -0.616002 -1.031844
54 1 0 -3.550066 -1.018410 -2.052512
55 6 0 -2.806390 1.615328 -0.560536
56 1 0 -2.333471 1.822621 0.406588
57 6 0 -3.319923 -1.817651 -0.062363
58 1 0 -4.219130 -2.445385 -0.129146
59 6 0 -3.023036 -1.426246 1.367828
60 6 0 -3.792418 -0.738236 2.299511
61 1 0 -4.791493 -0.389703 2.064276
62 6 0 -3.243514 -0.503164 3.563075
63 1 0 -3.829325 0.029023 4.305893
64 6 0 -1.955103 -0.941197 3.888024
65 1 0 -1.554094 -0.741043 4.876191
66 6 0 -1.188060 -1.635845 2.954465
67 1 0 -0.188284 -1.984195 3.189640
68 6 0 -1.745449 -1.869787 1.702529
69 6 0 -1.183182 -2.619694 0.553877
70 6 0 -2.017109 -3.319767 -1.710153

71 1 0 -2.961340 -3.123371 -2.227583
72 6 0 -0.878974 -2.753011 -2.567129
73 1 0 -0.969815 -1.670312 -2.674865
74 1 0 -0.902847 -3.203983 -3.563565
75 1 0 0.089423 -2.970891 -2.115909
76 6 0 -1.905671 -4.836455 -1.509248
77 1 0 -1.908239 -5.342151 -2.479022
78 1 0 -2.748438 -5.219428 -0.927190
79 1 0 -0.982828 -5.095052 -0.989397
80 7 0 -2.137660 -2.616384 -0.420240
81 8 0 -2.401733 2.555234 -1.493833
82 1 0 -1.489608 2.833602 -1.272389
83 8 0 -2.401338 0.272510 -0.935466
84 8 0 -0.085620 -3.178262 0.480429

Neutron diffraction experimental results

Table S15. Neutron diffraction experimental records

```
10-May-17 15:37 Protocol ON
 # d4
 Diffractometer parameters
  Wavelength [0.90850] ?

  Zero mark positions:   0.000   0.000   0.000   0.000
  ?

  Angular limits:  2th+   omg+   chi+   phi+   2th-   omg-   chi-   phi-
               120.0   60.0  181.0  181.0  -45.0  -25.0 -181.0 -181.0
  ?

  Max 2th-omg, shadow of cradle:   0.0   0.0   0.0   0.0
  ?

  Angle(opt-cnt), d(crystal-cnt), d(crystal-source):   0.00  420.0 2000.0
  ?

  Viewing position (2th, omg): -30.0  55.0
  ?

  Speed 1 and 2:  0.50  3.00 deg/sec ?

 # lg
 Laue group: -1 ? 2/m
 Data stored in chon.pm4
 # rc
 Enter cell constants
  a:    10.0000 ? 9.033
  b:    10.0000 ? 11.605
  c:    10.0000 ? 15.369
  beta :    90.00 ? 106.24

 Cell          9.0330   11.6050   15.3690   90.000  106.240   90.000
 Reciprocal   0.115306  0.086170  0.067770   90.000   73.760   90.000
 Volume       1546.82
 a_R     =    7.3105,  alpha_R =    48.6451

 Orienting     0.11530606 -0.00000000  0.01895270
 Matrix        0.00000000  0.08616975 -0.00000000
 (A* B* C*)    0.00000000  0.00000000  0.06506604
```

```
Data stored in chon.pm4
# ca 1 0 0
Angles =    6.0048   3.0024   0.0000   0.0000
# ca 4 0 0
Angles =   24.1874  12.0937   0.0000   0.0000
# ca 0 4 0
Angles =   18.0158   9.0079   0.0000 -90.0000
# ca 0 0 4
Angles =   14.1465   7.0733  73.7600   0.0000
# ca 0 0 6
Angles =   21.2882  10.6441  73.7600   0.0000
# th 0 0 0 0
# dr
# ca 4 0 0
Angles =   24.1874  12.0937   0.0000   0.0000
# dr
# ca 8 0 0
Angles =   49.5457  24.7729   0.0000   0.0000
# ca 12 0 0
Angles =   77.8840  38.9420   0.0000   0.0000
# xi 2
Mode (1=HKL, 2=Angles, 3=Film-Coord, 4=HKL+Angles) [1] ?

Enter H K L - Terminate with a blank line
Seq#   1 ? 3 1 1
   19.91   9.95   9.85  -13.29
Seq#   2 ? 4 0 0
   24.19  12.09   0.00    0.00
Seq#   3 ? 0 4 0
   18.02   9.01   0.00  -90.00
Seq#   4 ? 0 0 4
   14.15   7.07  73.76    0.00
Seq#   5 ? 5 3 1
   34.48  17.24   5.72  -23.46
Seq#   6 ? 5 2 0
   31.73  15.86   0.00  -16.64
Seq#   7 ? 3 4 1
   26.58  13.29   7.38  -43.37
Seq#   8 ? 2 7 3
   36.87  18.43  16.29  -64.52
Seq#   9 ? 9 5 3
   65.59  32.80   9.42  -21.49 ** Bisection not possible
Seq#  10 ? 9 6 7
   76.19  38.09  19.59  -23.83
```

```
Seq#  11 ? 9 7 8
   81.13   40.56   21.32  -26.89 ** Bisection not possible
Seq#  12 ?

 11 entries added to list 2   New n =  11
233 free entries left
# sv
Step scan parameters
 Nstep     :   21 ? 31
 Stepwidths:   0.0000   0.0500   0.0000   0.0000 ? 0 0 0 3
 Time/step :   0.50 sec ?

Data stored in chon.pm4
# ca 4 0 0
Angles =    24.1874   12.0937    0.0000    0.0000
# co 1 1
    1      0
# co 1 1
    0      0
# co 1 1
  116   1754
# co 1 1
  173   3130
# co 1 1
  160   3177
# co 1 1
  162   3260
# ss
Scan centre =    24.1874   12.0937    0.0000    0.0000
Scan range  =     0.0000    0.0000    0.0000   90.0000  ( 31 steps, stepmode)
  144   3244 |                        :          o
  136   3112 |                    :          o
  150   3340 |                          :          o
  140   3236 |                        :        o
  182   3482 +                             :                      o
  124   3258 |                        : o
  126   3146 |                     :    o
  126   3178 |                      :   o
  138   3152 |                     :         o
  168   3248 +                        :                     o
  152   3178 |                      :              o
  154   3210 |                       :               o
  172   3236 |                        :                     o
  152   3398 |                           :          o
```

```
  160   3328 +                        :                o
  162   3260 |                      :                  o
  154   3244 |                      :              o
  130   3134 |                  :      o
  158   3238 |                      :                o
  148   3192 +                    :             o
  166   3296 |                       :                  o
  140   3212 |                     :         o
  162   3288 |                       :                o
  160   3236 |                      :                o
  168   3222 +                     :                     o
  156   3316 |                        :             o
  158   3260 |                      :                o
  148   3212 |                     :            o
  150   3214 |                     :             o
  168   3406 +                          :                o
  156   3218 |                     :                 o
```

Centre at point 16.000   Angles =   24.1874   12.0937   0.0000   0.0000

I(int)=   136.80 Sigma=108.46 FWHM=37.4167 Imax(5)=   31.60 Temp=   0.00K

** Int. too low

ph 90

ss

Scan centre =   24.1874   12.0937   0.0000   90.0000

Scan range =   0.0000   0.0000   0.0000   90.0000 (31 steps, stepmode)

```
  144   3298 |                          :       o
  162   3258 |                        :               o
  134   3188 |                      :      o
  124   3160 |                     :  o
  158   3092 +                  :                  o
  146   3266 |                         :         o
  132   3158 |                     :      o
  166   3164 |                     :                   o
  156   3282 |                         :            o
  172   3270 +                         :                  o
  132   3236 |                        :    o
  146   3318 |                          :        o
  170   3212 |                       :                   o
  138   3230 |                       :       o
  164   3384 +                            :            o
  184   3276 |                         :                        o
  160   3118 |                    :                 o
  184   3228 |                       :                         o
  158   3196 |                      :               o
  170   3266 +                         :                  o
```

```
  152   3082 |                      :                 o
  148   3060 |                     :                o
  154   3314 |                           :           o
  166   3310 |                           :                o
  172   3216 +                        :                     o
  124   3380 |                        o    :
  140   3312 |                           :     o
  138   3280 |                          :      o
  144   3202 |                        :          o
  156   3204 +                        :              o
  122   3130 |                      :   o
Centre at point  16.000      Angles =    24.1874   12.0937    0.0000   90.0000
I(int)=   923.40  Sigma=111.95  FWHM=5.6741  Imax( 18)=    42.14  Temp=   0.00K
** Int. too low
# ph 180
# ss
Scan centre =    24.1874   12.0937    0.0000  180.0000
Scan range  =     0.0000    0.0000    0.0000   90.0000  ( 31 steps, stepmode)
  142   3370 |                           :        o
  176   3372 |                            :                  o
  144   3238 |                       :            o
  150   3332 |                          :           o
  118   3232 +                       :    o
  108   3228 |                       : o
  128   3328 |                          :     o
  180   3192 |                      :                        o
  144   3286 |                         :          o
  144   3286 +                         :          o
  136   3230 |                       :          o
  176   3366 |                           :                   o
  148   3212 |                      :              o
  154   3226 |                       :               o
  122   3214 +                      :      o
  128   3350 |                           :    o
  148   3216 |                       :             o
  134   3242 |                       :          o
  126   3426 |                             :  o
  172   3338 +                          :                  o
  174   3292 |                         :                   o
  146   3238 |                       :             o
  130   3262 |                        :       o
  154   3324 |                          :             o
  128   3112 +                    :           o
  132   3286 |                         :       o
```

```
  140   3300 |                         :         o
  152   3370 |                          :           o
  140   3262 |                        :          o
  190   3278 +                         :                        o
  134   3272 |                        :        o
Centre at point   4.214      Angles =    24.1874   12.0937    0.0000  144.6429
I(int)=   -84.00  Sigma=104.77  FWHM=19.7059  Imax( 30)=    44.00  Temp=   0.00K
** Unbalanced background
# ph 270
# ss
Scan centre =    24.1874   12.0937    0.0000  -90.0000
Scan range  =     0.0000    0.0000    0.0000   90.0000  ( 31 steps, stepmode)
  160   3214 |                      :                 o
  146   3294 |                         :          o
  162   3150 |                    :                    o
  142   3168 |                     :             o
  122   3198 +                     :       o
  148   3250 |                       :            o
  180   3326 |                         :                    o
  122   3430 |                           o  :
  146   3190 |                     :              o
  160   3360 +                          :             o
  136   3328 |                         :       o
  178   3270 |                        :                    o
  148   3304 |                         :          o
  148   3128 |                    :               o
  142   3272 +                        :          o
  136   3236 |                       :         o
  192   3274 |                        :                        o
  172   3136 |                    :                       o
  156   3394 |                           :           o
  162   3400 +                           :             o
  110   3380 |                        o   :
  150   3364 |                          :          o
  144   3152 |                    :              o
  178   3442 |                            :                o
  112   3406 +                         o   :
  164   3372 |                          :              o
  186   3370 |                          :                     o
  166   3308 |                         :               o
  158   3324 |                         :              o
  122   3282 +                        :    o
  142   3228 |                      :            o
Centre at point  16.000      Angles =    24.1874   12.0937    0.0000  -90.0000
```

```
I(int)=    64.20  Sigma=106.88  FWHM=18.6265  Imax( 17)=    41.08  Temp=   0.00K
** Int. too low
# ca 0 0 4
Angles =    14.1465    7.0733   73.7600    0.0000
# ca 0 4 0
Angles =    18.0158    9.0079    0.0000  -90.0000
# dr
# ca 4 0 0
Angles =    24.1874   12.0937    0.0000    0.0000
# ch 90
# dr
# ca 0 4 0
Angles =    18.0158    9.0079    0.0000  -90.0000
# ch 90
# dr
# co 1 1
   119   6073
# sv
Step scan parameters
 Nstep    :  31 ?

 Stepwidths:   0.0000   0.0000   0.0000   3.0000 ? 0 0.3 0 0
 Time/step :   0.50 sec ?

Data stored in chon.pm4
# ss
Scan centre =    18.0158    9.0077   90.0000  -90.0000
Scan range  =     0.0000    9.0000    0.0000    0.0000  ( 31 steps, stepmode)
    96   6190 |                         :          o
    98   6184 |                         :            o
   114   6270 |                           :                o
   102   6178 |                          :             o
    88   6162 +                        :        o
    88   5934 |                    :            o
    84   6258 |                         :    o
    82   6234 |                         :   o
    88   6142 |                        :        o
   120   6122 +                        :                       o
   102   6052 |                       :                o
    94   6152 |                        :          o
    78   6346 |                          o :
   100   6164 |                         :             o
   122   6128 +                         :                        o
   114   5884 |                    :                         o
```

```
  124   6150 |                         :                        o
   84   6134 |                         :      o
  108   6168 |                         :                 o
   96   6196 +                         :          o
  122   6174 |                          :                      o
  114   6088 |                         :                    o
   86   6170 |                         :       o
   86   6168 |                         :       o
   88   6154 +                         :        o
  100   6074 |                        :               o
  100   6090 |                         :              o
  120   6114 |                         :                      o
   90   6198 |                          :       o
   72   6242 +                         o  :
   70   6150 |                        o :
Centre at point  16.000      Angles =    18.0158    9.0077   90.0000  -90.0000
I(int)=    25.50  Sigma=84.71  FWHM=0.8717  Imax( 17)=    29.35  Temp=   0.00K
** Int. too low
# ca 4 0 0
Angles =    24.1874   12.0937    0.0000    0.0000
# dr
# co 1 1
    3     5
# ch 0 0
# dr
# co 1 1
    2   2706
# co 1 1
    2   5988
# co 1 1
    3   6115
# sv
Step scan parameters
 Nstep     :   31 ? 181
 Stepwidths:   0.0000   0.3000   0.0000   0.0000 ? 0 0 0 0.2
 Time/step :   0.50 sec ?

Data stored in chon.pm4
# ca 4 0 0
Angles =    24.1874   12.0937    0.0000    0.0000
# ss
Scan centre =    24.1874   12.0937    0.0000    0.0000
Scan range  =     0.0000    0.0000    0.0000   36.0000  (181 steps, stepmode)
    8   6042 |      o                        :
```

```
10   6014 |       o              :
 4   6024 |  o                   :
 2   6122 | o                    :
 0   5972 +o                     :
 2   5964 | o                    :
 2   6134 | o                    :
 0   6088 |o                     :
 4   6056 |  o                   :
 2   6046 + o                    :
 6   5918 |    o                 :
 4   5826 |  o                   :
 4   6148 |  o                   :
 4   5790 |  o                   :
 4   5892 +  o                   :
 4   6138 |  o                   :
 0   5794 |o                     :
 4   6158 |  o                   :
 6   5982 |    o                 :
 6   5912 +    o                 :
 2   5954 | o                    :
 4   5952 |  o                   :
 0   6008 |o                     :
10   6120 |       o              :
 6   5980 +    o                 :
 6   5874 |    o                 :
 2   5838 | o                    :
 6   5962 |    o                 :
 4   6012 |  o                   :
 2   5838 + o                    :
 4   5826 |  o                   :
 4   5684 |  o                   :
 2   5854 | o                    :
 6   5784 |    o                 :
 2   5900 + o                    :
 6   6076 |    o                 :
 4   5820 |  o                   :
 4   5724 |  o                   :
 4   5758 |  o                   :
 2   5776 + o                    :
 2   5866 | o                    :
 2   5872 | o                    :
 6   5738 |    o                 :
 8   5776 |      o               :
 2   5818 + o                    :
```

```
4   5746 |   o                       :
0   5764 |o                           :
2   5778 | o                         :
2   5630 | o                        :
2   5818 + o                         :
4   5658 |   o                       :
0   5662 |o                           :
2   5760 | o                         :
0   5692 |o                           :
4   5540 +   o                      :
4   5662 |   o                       :
4   5574 |   o                      :
0   5602 |o                          :
4   5480 |   o                      :
4   5508 +   o                      :
2   5470 | o                        :
4   5536 |   o                      :
0   5392 |o                          :
0   5514 |o                          :
8   5430 +       o                  :
0   5392 |o                          :
2   5426 | o                        :
2   5326 | o                      :
2   5224 | o                      :
0   5298 +o                        :
6   5278 |     o                  :
4   5178 |   o                    :
4   5146 |   o                    :
6   5096 |     o                  :
4   5022 +   o                   :
0   4970 |o                       :
4   5138 |   o                    :
6   5068 |     o                  :
2   4982 | o                     :
0   4962 +o                       :
6   4954 |     o                 :
6   5006 |     o                 :
6   4882 |     o                 :
4   4732 |   o                  :
8   4772 +       o               :
2   4760 | o                     :
6   4788 |     o                 :
4   4826 |   o                   :
2   4906 | o                     :
```

```
6  4694 +     o                          :
0  4738 |o                                :
0  4458 |o                                :
4  4456 |   o                            :
6  4570 |     o                          :
6  4504 +     o                          :
4  4412 |   o                           :
2  4390 | o                             :
4  4538 |   o                            :
2  4428 | o                             :
2  4252 + o                             :
0  4264 |o                               :
4  4296 |   o                           :
4  4158 |   o                           :
0  4082 |o                              :
4  3990 +   o                          :
0  4064 |o                              :
2  4002 | o                            :
6  4222 |     o                         :
6  3994 |     o                        :
0  3946 +o                              :
0  3842 |o                             :
4  3870 |   o                          :
2  3854 | o                            :
6  3888 |     o                        :
8  3688 +       o                     :
6  3680 |     o                       :
0  3726 |o                             :
4  3794 |   o                         :
2  3708 | o                           :
6  3708 +     o                       :
4  3772 |   o                         :
0  3706 |o                             :
2  3592 | o                           :
4  3520 |   o                        :
2  3662 + o                           :
0  3518 |o                            :
0  3536 |o                            :
2  3694 | o                           :
2  3602 | o                           :
8  3560 +       o                     :
6  3546 |     o                      :
2  3546 | o                          :
0  3486 |o                            :
```

```
12  3558 |      o        :
 2  3496 + o             :
 2  3676 | o             :
 2  3478 | o             :
 6  3522 |   o           :
 2  3344 | o             :
 4  3526 +  o            :
 4  3358 |  o            :
 4  3594 |  o            :
 2  3468 | o             :
 2  3484 | o             :
 0  3378 +o              :
 0  3326 |o              :
 2  3286 | o             :
 6  3350 |   o           :
10  3466 |     o         :
 6  3388 +   o           :
 6  3454 |   o           :
 6  3422 |   o           :
 2  3392 | o             :
 0  3448 |o              :
 2  3514 + o             :
 4  3374 |  o            :
 2  3586 | o             :
 2  3436 | o             :
 2  3388 | o             :
 2  3402 + o             :
 6  3438 |   o           :
 4  3416 |  o            :
 4  3362 |  o            :
 0  3376 |o              :
 2  3306 + o             :
 6  3430 |   o           :
 4  3432 |  o            :
 2  3472 | o             :
12  3448 |      o        :
 4  3490 +  o            :
 0  3370 |o              :
 2  3296 | o             :
 2  3464 | o             :
 2  3272 | o             :
 2  3342 + o             :
 0  3348 |o              :
 6  3408 |   o           :
```

```
   6   3258 |     o                  :
   2   3418 | o                      :
   0   3428 +o                        :
   2   3180 | o                     :
Centre at point  21.207      Angles =    24.1874   12.0937    0.0000  -13.9586
I(int)=    -4.29  Sigma=39.39  FWHM=4.6278  Imax(169)=     9.02  Temp=   0.00K

# ph 36
# ss
Scan centre =    24.1874   12.0937    0.0000   36.0000
Scan range  =     0.0000    0.0000    0.0000   36.0000  (181 steps, stepmode)
   0   3340 |o                            :
   6   3442 |     o                        :
   4   3342 |   o                        :
   0   3298 |o                           :
   6   3360 +     o                      :
   8   3394 |       o                      :
  10   3456 |         o                      :
   2   3472 | o                            :
   0   3306 |o                           :
   4   3284 +   o                      :
   4   3280 |   o                      :
   8   3442 |       o                      :
   4   3212 |   o                     :
   0   3292 |o                           :
   4   3232 +   o                     :
   6   3200 |     o                   :
   6   3278 |     o                    :
   2   3330 | o                        :
   2   3430 | o                          :
   4   3478 +   o                         :
   2   3226 | o                     :
   0   3408 |o                             :
   4   3386 |   o                        :
   4   3286 |   o                      :
   8   3314 +      o                   :
   0   3284 |o                          :
   4   3380 |   o                        :
   6   3348 |     o                     :
  10   3300 |         o                  :
   4   3222 +   o                     :
   4   3316 |   o                      :
   4   3248 |   o                     :
   6   3178 |     o                   :
```

```
4  3330 |  o              :
8  3284 +    o           :
6  3350 |   o             :
4  3218 |  o            :
2  3330 | o               :
4  3234 |  o            :
4  3412 +  o               :
2  3234 | o             :
6  3322 |   o            :
0  3164 |o              :
4  3384 |  o              :
2  3234 + o             :
0  3204 |o              :
4  3266 |  o             :
4  3190 |  o            :
8  3254 |    o           :
0  3430 +o                 :
4  3434 |  o               :
6  3370 |   o             :
10 3220 |     o         :
4  3366 |  o              :
2  3286 + o              :
2  3222 | o             :
6  3210 |   o           :
6  3456 |   o              :
0  3178 |o              :
4  3318 +  o             :
6  3252 |   o            :
4  3324 |  o             :
8  3366 |    o            :
4  3308 |  o             :
2  3320 + o              :
4  3262 |  o             :
10 3424 |     o            :
4  3398 |  o              :
8  3446 |    o             :
6  3320 +   o            :
10 3340 |     o           :
4  3210 |  o            :
2  3362 | o               :
2  3422 | o                :
4  3292 +  o             :
0  3332 |o                :
2  3296 | o              :
```

```
6  3356 |   o             :
4  3346 |  o              :
2  3376 + o               :
4  3282 |  o             :
2  3204 | o             :
2  3352 | o               :
4  3298 |  o             :
2  3370 + o               :
4  3244 |  o            :
4  3226 |  o            :
4  3302 |  o             :
6  3246 |   o           :
4  3404 +  o              :
8  3290 |    o           :
6  3290 |   o            :
2  3124 | o            :
2  3302 | o              :
2  3246 + o             :
2  3446 | o                :
6  3336 |   o             :
4  3418 |  o               :
2  3318 | o              :
0  3324 +o                :
6  3278 |   o            :
2  3382 | o               :
4  3374 |  o              :
0  3348 |o                :
4  3404 +  o              :
0  3342 |o                :
2  3300 | o              :
0  3316 |o                :
4  3270 |  o             :
4  3148 +  o           :
10  3386 |     o           :
0  3472 |o                  :
2  3326 | o              :
6  3278 |   o            :
0  3442 +o                 :
6  3300 |   o            :
6  3186 |   o           :
4  3356 |  o              :
0  3128 |o              :
4  3370 +  o              :
2  3196 | o             :
```

```
8   3286 |       o                    :
2   3388 | o                            :
2   3328 | o                          :
6   3362 +     o                      :
2   3180 | o                      :
6   3394 |     o                        :
2   3242 | o                        :
4   3272 |   o                      :
2   3312 + o                        :
4   3332 |   o                        :
2   3076 | o                  :
4   3348 |   o                        :
8   3252 |       o                  :
6   3304 +     o                    :
6   3154 |     o                  :
0   3206 |o                         :
6   3242 |     o                  :
6   3300 |     o                    :
6   3200 +     o                  :
4   3090 |   o                  :
0   3116 |o                       :
8   3210 |       o                :
6   3306 |     o                    :
2   3362 + o                          :
2   3124 | o                    :
4   3292 |   o                      :
4   3270 |   o                    :
4   3200 |   o                  :
10   3232 +         o                :
4   3252 |   o                    :
8   3336 |       o                  :
2   3266 | o                      :
0   3314 |o                         :
0   3400 +o                           :
8   3258 |       o                :
2   3272 | o                      :
4   3214 |   o                    :
4   3294 |   o                      :
0   3198 +o                       :
2   3250 | o                      :
2   3328 | o                        :
10   3158 |         o              :
4   3206 |   o                  :
4   3274 +   o                    :
```

```
   4   3154 |   o                   :
   4   3300 |   o                      :
   0   3344 |o                          :
   4   3260 |   o                     :
   2   3186 + o                     :
   4   3340 |   o                      :
   4   3216 |   o                    :
   8   3302 |      o                   :
   6   3234 |     o                  :
  10   3364 +        o                  :
   8   3320 |      o                   :
   0   3228 |o                        :
   6   3254 |     o                   :
   2   3250 | o                       :
   0   3272 +o                         :
   0   3296 |o                          :
Centre at point 119.327     Angles =    24.1874   12.0937    0.0000   41.6654
I(int)=    1.22  Sigma=41.92  FWHM=2.3342  Imax(175)=    6.29  Temp=   0.00K

# ph 72
# ss
Scan centre =    24.1874   12.0937    0.0000   72.0000
Scan range  =    0.0000    0.0000    0.0000   36.0000  (181 steps, stepmode)
   2   3278 | o                      :
   4   3368 |   o                     :
   2   3284 | o                       :
   0   3238 |o                        :
   4   3386 +   o                      :
   2   3322 | o                       :
   4   3420 |   o                      :
   2   3316 | o                       :
   4   3282 |   o                    :
   2   3272 + o                      :
   4   3424 |   o                      :
   0   3312 |o                          :
   4   3166 |   o                 :
   4   3306 |   o                   :
  14   3280 +           o            :
   2   3424 | o                        :
   2   3362 | o                        :
   2   3446 | o                         :
   6   3268 |     o                  :
   2   3488 + o                          :
   4   3260 |   o                    :
```

```
 0  3430 |o                                      :
 0  3260 |o                                  :
 6  3366 |   o                                :
 4  3356 +  o                                 :
 8  3262 |    o                             :
10  3306 |     o                               :
 0  3470 |o                                      :
 2  3158 | o                               :
 0  3328 +o                                   :
 4  3182 |  o                               :
 4  3358 |  o                                 :
10  3238 |     o                              :
 8  3258 |    o                             :
 4  3254 +  o                                :
 2  3246 | o                                :
 0  3396 |o                                     :
 0  3316 |o                                   :
 2  3190 | o                               :
 4  3272 +  o                                :
 8  3462 |    o                                 :
 4  3280 |  o                               :
 4  3438 |  o                                   :
 8  3402 |    o                                :
 2  3348 + o                                  :
 0  3264 |o                                  :
 2  3398 | o                                   :
 4  3296 |  o                                :
 2  3284 | o                                 :
 6  3460 +   o                                  :
 4  3384 |  o                                  :
 2  3260 | o                                :
 2  3286 | o                                 :
 2  3320 | o                                 :
 4  3216 +  o                               :
 8  3244 |    o                             :
 0  3342 |o                                    :
 4  3304 |  o                                :
 2  3334 | o                                  :
 2  3426 + o                                   :
 4  3222 |  o                              :
 6  3378 |   o                                :
 4  3252 |  o                               :
 0  3266 |o                                  :
 8  3346 +    o                               :
```

```
 4  3228 |    o                          :
 4  3250 |    o                           :
12  3214 |            o                  :
 4  3362 |    o                             :
 6  3178 +      o                       :
 8  3336 |        o                         :
 6  3440 |      o                             :
 2  3220 |  o                            :
 2  3376 |  o                               :
 4  3566 +    o                                 :
 4  3298 |    o                            :
 2  3482 |  o                                  :
 2  3330 |  o                              :
 8  3268 |        o                       :
 2  3256 +  o                             :
 0  3314 |o                                 :
 2  3156 |  o                           :
 0  3322 |o                                 :
 4  3422 |    o                              :
 0  3330 +o                                 :
 2  3362 |  o                               :
 4  3148 |    o                         :
 0  3410 |o                                   :
10  3298 |          o                      :
 6  3232 +      o                         :
 4  3268 |    o                           :
 4  3240 |    o                           :
 4  3286 |    o                            :
 6  3324 |      o                          :
 4  3220 +    o                          :
 0  3302 |o                                 :
10  3190 |          o                    :
10  3348 |          o                       :
 2  3290 |  o                              :
 8  3288 +        o                        :
 2  3250 |  o                             :
 4  3392 |    o                              :
 4  3236 |    o                           :
 4  3130 |    o                        :
10  3236 +          o                     :
 6  3324 |      o                          :
 4  3422 |    o                              :
 0  3270 |o                                :
 2  3182 |  o                            :
```

```
6  3258 +   o           :
4  3314 |  o            :
2  3328 | o             :
2  3282 | o             :
2  3174 | o            :
2  3148 + o            :
2  3346 | o              :
6  3402 |   o            :
2  3376 | o              :
0  3236 |o              :
2  3246 + o             :
0  3224 |o              :
4  3372 |  o             :
8  3070 |    o        :
6  3276 |   o           :
2  3444 + o               :
2  3274 | o             :
4  3242 |  o            :
0  3282 |o                :
0  3292 |o                :
10  3350 +    o           :
2  3352 | o              :
2  3200 | o            :
2  3304 | o             :
6  3294 |   o           :
6  3152 +   o          :
4  3398 |  o             :
2  3288 | o             :
8  3164 |    o         :
4  3304 |  o            :
0  3178 +o             :
6  3204 |   o          :
2  3220 | o            :
2  3222 | o            :
2  3346 | o              :
0  3482 +o                 :
2  3356 | o              :
2  3224 | o            :
6  3280 |   o           :
2  3210 | o            :
4  3168 +  o           :
6  3204 |   o          :
2  3148 | o            :
6  3410 |   o            :
```

2 3278 | o :
2 3284 + o :
8 3258 | o :
2 3156 | o :
6 3314 | o :
2 3496 | o :
0 3296 +o :
6 3198 | o :
0 3348 |o :
0 3420 |o :
2 3228 | o :
0 3214 +o :
6 3258 | o :
4 3356 | o :
4 3284 | o :
2 3348 | o :
4 3288 + o :
2 3176 | o :
0 3252 |o :
2 3294 | o :
0 3244 |o :
4 3254 + o :
4 3342 | o :
2 3326 | o :
6 3180 | o :
4 3128 | o :
4 3404 + o :
2 3302 | o :
Centre at point 91.000 Angles = 24.1874 12.0937 0.0000 72.0000
I(int)= 14.57 Sigma=37.94 FWHM=1.8694 Imax(15)= 10.67 Temp= 0.00K
** Int. too low
# ph 108
# ss
Scan centre = 24.1874 12.0937 0.0000 108.0000
Scan range = 0.0000 0.0000 0.0000 36.0000 (181 steps, stepmode)
0 3362 |o :
4 3328 | o :
0 3396 |o :
4 3270 | o :
4 3236 + o :
2 3244 | o :
0 3350 |o :
2 3476 | o :
8 3284 | o :

```
 0  3232 +o                    :
 2  3326 | o                    :
 4  3084 |   o               :
10  3256 |       o            :
 4  3212 |   o                :
 4  3374 +   o                  :
 8  3218 |      o             :
 4  3360 |   o                  :
 4  3138 |   o               :
 8  3222 |      o             :
 2  3204 + o                  :
 0  3326 |o                     :
 6  3074 |     o            :
 2  3276 | o                   :
10  3270 |       o            :
 0  3116 +o                  :
 6  3360 |     o                :
 2  3304 | o                   :
 0  3334 |o                     :
 2  3338 | o                    :
 0  3278 +o                   :
 2  3226 | o                 :
 2  3320 | o                   :
 4  3338 |   o                  :
 2  3218 | o                 :
 4  3304 +   o                :
 2  3424 | o                     :
 2  3298 | o                   :
 2  3336 | o                    :
 6  3216 |     o              :
 2  3272 + o                  :
 4  3262 |   o                :
 0  3286 |o                    :
 2  3148 | o               :
 2  3416 | o                     :
 2  3274 + o                  :
 2  3136 | o               :
 6  3326 |     o                :
 4  3442 |   o                  :
 2  3272 | o                  :
 0  3200 +o                  :
 4  3306 |   o                :
 2  3246 | o                  :
 0  3300 |o                    :
```

```
4 3372 |   o                              :
2 3180 + o                           :
2 3296 | o                             :
2 3350 | o                              :
4 3218 |   o                         :
0 3344 |o                                :
4 3278 +   o                          :
8 3246 |       o                      :
0 3264 |o                              :
4 3250 |   o                          :
0 3296 |o                               :
8 3340 +       o                         :
2 3200 | o                          :
0 3398 |o                                 :
2 3272 | o                            :
6 3354 |     o                           :
0 3102 +o                          :
8 3374 |       o                         :
6 3306 |     o                          :
4 3398 |   o                             :
0 3226 |o                             :
6 3368 +     o                           :
2 3200 | o                          :
4 3418 |   o                              :
2 3300 | o                             :
2 3274 | o                            :
6 3354 +     o                          :
8 3304 |       o                       :
2 3226 | o                           :
6 3246 |     o                        :
2 3354 | o                              :
0 3470 +o                                   :
2 3224 | o                           :
2 3410 | o                                :
8 3236 |       o                     :
2 3336 | o                              :
6 3240 +     o                        :
4 3324 |   o                            :
8 3214 |       o                     :
2 3234 | o                           :
0 3130 |o                           :
2 3292 + o                             :
8 3318 |       o                       :
4 3354 |   o                            :
```

```
2  3374 | o                        :
6  3246 |    o                  :
4  3302 +  o                      :
2  3236 | o                     :
2  3276 | o                      :
0  3242 |o                       :
2  3210 | o                     :
6  3304 +    o                    :
6  3176 |    o                 :
0  3362 |o                          :
2  3230 | o                     :
4  3168 |  o                   :
2  3152 + o                   :
0  3344 |o                          :
2  3152 | o                   :
2  3326 | o                       :
2  3274 | o                      :
8  3124 +     o                :
4  3416 |  o                        :
2  3154 | o                   :
0  3388 |o                         :
6  3070 |    o               :
4  3390 +  o                      :
4  3238 |  o                   :
0  3230 |o                       :
6  3292 |    o                  :
4  3260 |  o                    :
6  3338 +    o                   :
4  3184 |  o                   :
10  3238 |       o               :
6  3318 |    o                  :
6  3296 |    o                  :
2  3362 + o                       :
6  3324 |    o                   :
8  3298 |     o                  :
0  3286 |o                        :
4  3342 |  o                     :
2  3278 + o                      :
6  3170 |    o                 :
8  3350 |     o                  :
0  3334 |o                          :
4  3378 |  o                       :
8  3214 +     o                 :
2  3082 | o                  :
```

```
   0   3184 |o                    :
   6   3172 |    o                :
   2   3318 | o                     :
   0   3306 +o                      :
   8   3228 |      o              :
   4   3154 |   o                :
   2   3192 | o                  :
   0   3144 |o                   :
   8   3212 +      o              :
   6   3218 |    o                :
   0   3226 |o                     :
   2   3124 | o                  :
   2   3298 | o                     :
   8   3226 +      o              :
   4   3144 |   o                :
   4   3304 |   o                  :
   0   3362 |o                       :
   2   3336 | o                      :
   4   3374 +   o                    :
   4   3340 |   o                   :
   6   3222 |    o                :
   0   3260 |o                     :
   6   3248 |    o                 :
   4   3192 +   o                :
   0   3240 |o                    :
   6   3230 |    o                :
   4   3098 |   o               :
   2   3280 | o                   :
   2   3338 + o                     :
   6   3454 |    o                      :
   0   3236 |o                    :
   4   3262 |   o                 :
   6   3420 |    o                    :
   6   3290 +    o                  :
   2   3212 | o                  :
   2   3150 | o                :
   2   3380 | o                     :
   4   3246 |   o                 :
   2   3244 + o                   :
   6   3356 |    o                  :
```
Centre at point 181.000 Angles = 24.1874 12.0937 0.0000 126.0000

I(int)= 0.93 Sigma=38.95 FWHM=5.2446 Imax(127)= 6.65 Temp= 0.00K

** Unsuitable scanwidth

ph 144

```
# ss
Scan centre =    24.1874   12.0937    0.0000  144.0000
Scan range  =     0.0000    0.0000    0.0000   36.0000  (181 steps, stepmode)
    2   3364 | o                    :
    4   3202 |   o                  :
    0   3416 |o                     :
    2   3144 | o                   :
    0   3280 +o                     :
    6   3254 |     o                :
    4   3202 |   o                  :
    0   3302 |o                     :
    4   3174 |   o                 :
    2   3182 + o                   :
    2   3246 | o                    :
    2   3308 | o                    :
    2   3332 | o                    :
    8   3290 |       o              :
    0   3320 +o                     :
    2   3286 | o                    :
    2   3234 | o                    :
   12   3286 |           o          :
    0   3384 |o                     :
    0   3262 +o                     :
    2   3302 | o                    :
    6   3484 |     o                :
    6   3318 |     o                :
    0   3370 |o                     :
    4   3186 +   o                 :
    6   3234 |     o                :
    2   3358 | o                    :
    8   3236 |       o              :
    2   3254 | o                    :
    4   3296 +   o                  :
    4   3242 |   o                  :
    6   3360 |     o                :
    4   3216 |   o                  :
    0   3238 |o                     :
    4   3264 +   o                  :
    2   3348 | o                    :
    2   3282 | o                    :
    6   3310 |     o                :
    2   3262 | o                    :
    2   3268 + o                    :
    8   3218 |       o              :
```

```
 2  3292 | o             :
 6  3410 |   o           :
 2  3212 | o             :
 8  3316 +    o          :
 2  3238 | o             :
 4  3442 |  o            :
 0  3328 |o               :
 8  3282 |    o          :
 0  3180 +o              :
 8  3350 |    o          :
 2  3360 | o             :
 4  3340 |  o            :
 4  3302 |  o            :
 4  3412 +  o            :
 6  3266 |   o           :
 4  3280 |  o            :
 4  3436 |  o            :
 0  3278 |o               :
 0  3524 +o                :
 4  3234 |  o            :
 4  3412 |  o            :
 4  3294 |  o            :
10  3270 |     o          :
 6  3262 +   o           :
 4  3222 |  o            :
 4  3336 |  o            :
 0  3478 |o               :
 4  3282 |  o            :
 4  3274 +  o            :
 4  3284 |  o            :
 2  3398 | o             :
 6  3288 |   o           :
 6  3274 |   o           :
 6  3180 +   o          :
 2  3338 | o             :
 0  3298 |o               :
 8  3212 |    o          :
 6  3352 |   o           :
 2  3296 + o             :
 2  3264 | o             :
 6  3198 |   o           :
 2  3162 | o            :
 4  3374 |  o            :
 2  3314 + o             :
```

```
0   3268 |o             :
2   3406 | o           :
0   3212 |o             :
6   3360 |   o         :
0   3348 +o             :
4   3314 |  o          :
6   3254 |   o         :
0   3520 |o              :
4   3184 |  o         :
2   3326 + o           :
0   3314 |o             :
8   3332 |    o        :
0   3264 |o             :
6   3204 |   o         :
4   3164 +  o         :
4   3158 |  o         :
4   3276 |  o          :
2   3232 | o           :
0   3340 |o             :
4   3264 +  o          :
4   3322 |  o          :
6   3314 |   o         :
4   3334 |  o          :
6   3434 |   o         :
8   3370 +    o        :
4   3386 |  o          :
6   3406 |   o         :
2   3312 | o           :
2   3146 | o          :
0   3226 +o             :
0   3328 |o             :
4   3336 |  o          :
4   3298 |  o          :
6   3254 |   o         :
2   3148 + o          :
2   3340 | o           :
0   3172 |o            :
0   3164 |o            :
4   3208 |  o          :
2   3244 + o           :
0   3402 |o             :
6   3110 |   o        :
4   3260 |  o          :
4   3196 |  o          :
```

```
0   3104 +o                    :
4   3210 |   o                 :
4   3312 |   o                 :
2   3336 | o                   :
2   3134 | o                  :
4   3156 +   o                :
2   3170 | o                  :
2   3300 | o                   :
2   3392 | o                   :
2   3288 | o                   :
2   3332 + o                   :
0   3170 |o                    :
2   3300 | o                   :
4   3368 |   o                 :
0   3342 |o                     :
4   3222 +   o                 :
4   3258 |   o                 :
0   3260 |o                     :
2   3274 | o                   :
6   3124 |     o              :
4   3304 +   o                 :
2   3200 | o                   :
2   6350 | o                           :
0   5966 |o                            :
4   6100 |   o                        :
0   6166 +o                             :
4   6226 |   o                         :
8   6178 |       o                     :
2   6168 | o                           :
0   6060 |o                            :
2   6148 + o                          :
6   6256 |     o                       :
2   6222 | o                           :
4   6100 |   o                        :
8   6264 |       o                     :
4   6184 +   o                         :
6   6288 |     o                       :
0   6398 |o                             :
2   6120 | o                          :
0   6120 |o                            :
4   6142 +   o                        :
4   6240 |   o                         :
4   6180 |   o                         :
12   6318 |          o                  :
```

```
   2   6358 | o                          :
   2   6104 + o                         :
   8   6112 |      o                    :
   8   6324 |      o                     :
   2   6106 | o                         :
   2   6158 | o                          :
   8   6284 +      o                    :
   0   6274 |o                           :
Centre at point 181.000      Angles =    24.1874   12.0937    0.0000  162.0000
I(int)=    -2.28  Sigma=39.06  FWHM=1.6971  Imax( 18)=     8.86  Temp=   0.00K
** Unsuitable scanwidth
# ph -36
# ss
Scan centre =    24.1874   12.0937    0.0000  -36.0000
Scan range  =     0.0000    0.0000    0.0000   36.0000  (181 steps, stepmode)
   4   6108 |   o                       :
   2   6032 | o                         :
   4   6260 |   o                       :
   6   6018 |     o                    :
   2   6072 + o                          :
   4   6116 |   o                       :
   6   5938 |     o                    :
   6   5972 |     o                    :
   2   5978 | o                        :
   4   6176 +   o                       :
   2   6018 | o                        :
   2   5870 | o                        :
   2   5996 | o                        :
   2   6010 | o                        :
   2   6128 + o                         :
   4   5896 |   o                      :
   4   6186 |   o                       :
   4   6144 |   o                       :
   6   5918 |     o                    :
   4   5944 +   o                      :
   4   6014 |   o                      :
   6   5870 |     o                    :
   0   6060 |o                           :
   2   6112 | o                         :
   4   5906 +   o                      :
   4   6102 |   o                       :
   6   6010 |     o                    :
   4   5906 |   o                      :
   4   5898 |   o                      :
```

```
 6  6098 |   o                     :
 2  6026 | o                       :
 4  5886 |  o                      :
10  5860 |     o                   :
 2  6006 | o                       :
 2  5834 + o                       :
 0  5894 |o                        :
10  5966 |     o                   :
 2  6024 | o                       :
 2  5858 | o                       :
 4  5964 +  o                      :
 6  5888 |   o                     :
 0  5908 |o                        :
10  6060 |     o                   :
 0  5862 |o                        :
10  5776 +     o                   :
 6  5802 |   o                     :
 2  5742 | o                       :
 6  5732 |   o                     :
 2  5952 | o                       :
 4  5748 +  o                      :
 2  5706 | o                       :
 0  5660 |o                        :
10  5772 |     o                   :
 2  5876 | o                       :
14  5772 +       o                 :
 4  5824 |  o                      :
10  5750 |     o                   :
 4  5766 |  o                      :
 2  5674 | o                       :
 4  5838 +  o                      :
 6  5788 |   o                     :
 4  5578 |  o                      :
 2  5856 | o                       :
 0  5580 |o                        :
 4  5478 +  o                      :
 0  5624 |o                        :
 2  5718 | o                       :
 2  5544 | o                       :
10  5526 |     o                   :
 4  5598 +  o                      :
 2  5434 | o                       :
 6  5496 |   o                     :
10  5496 |     o                   :
```

```
6 5364 |   o             :
6 5426 +   o              :
4 5354 |  o              :
2 5414 | o               :
10 5364 |     o           :
8 5458 |    o             :
2 5308 + o               :
2 5270 | o               :
2 5324 | o               :
2 5344 | o               :
2 5114 | o               :
2 5384 + o               :
2 5296 | o               :
2 5304 | o               :
4 5212 |  o              :
4 5130 |  o              :
2 5230 + o               :
0 4992 |o                :
4 5060 |  o             :
2 5024 | o              :
2 5076 | o              :
4 5058 +  o             :
6 5000 |   o            :
4 4978 |  o             :
0 4866 |o                :
2 4868 | o              :
2 4878 + o              :
0 4628 |o               :
4 4928 |  o             :
2 4908 | o              :
2 4462 | o             :
6 4674 +   o            :
0 4690 |o               :
8 4542 |    o           :
4 4372 |  o            :
4 4422 |  o            :
4 4476 +  o            :
2 4364 | o             :
10 4532 |     o         :
4 4374 |  o            :
6 4352 |   o           :
4 4414 +  o            :
4 4272 |  o            :
6 4092 |   o          :
```

```
 2  3994 | o             :
 2  3978 | o             :
 2  3968 + o             :
 4  4052 |  o            :
 4  4034 |  o            :
10  3970 |     o         :
 8  3930 |    o          :
 2  4034 + o             :
 0  3892 |o               :
 6  3942 |   o           :
 6  3944 |   o           :
 6  3824 |   o          :
 0  3820 +o              :
 0  3904 |o               :
 2  3764 | o            :
 6  3750 |   o          :
 2  3772 | o            :
 0  3614 +o              :
 2  3678 | o            :
 0  3778 |o              :
 4  3690 |  o           :
 6  3638 |   o          :
 0  3460 +o             :
 4  3610 |  o           :
 2  3510 | o           :
 0  3558 |o             :
 2  3646 | o            :
 4  3472 +  o          :
 6  3460 |   o         :
 4  3374 |  o          :
 4  3410 |  o          :
 0  3378 |o             :
 4  3490 +  o          :
 4  3328 |  o          :
 0  3428 |o             :
 2  3288 | o           :
 2  3438 | o           :
 0  3374 +o             :
 2  3404 | o           :
10  3420 |     o        :
 2  3336 | o           :
 4  3346 |  o          :
 2  3362 + o           :
 4  3336 |  o          :
```

```
    6   3398 |     o                  :
    4   3358 |   o                    :
    6   3358 |     o                  :
    4   3404 +   o                    :
    2   3416 | o                      :
    4   3350 |   o                    :
    4   3340 |   o                    :
    0   3322 |o                       :
    2   3260 + o                     :
    2   3394 | o                      :
    2   3196 | o                     :
    0   3302 |o                       :
    0   3258 |o                       :
    0   3270 +o                       :
    2   3296 | o                      :
    2   3222 | o                     :
    4   3352 |   o                    :
    0   3488 |o                       :
    0   3310 +o                       :
    2   3274 | o                      :
Centre at point  91.000      Angles =    24.1874   12.0937    0.0000  -36.0000
I(int)=    15.78  Sigma=37.77  FWHM=6.8966  Imax( 55)=    10.60  Temp=   0.00K
** Int. too low
# ph -72
# ss
Scan centre =    24.1874   12.0937    0.0000  -72.0000
Scan range  =     0.0000    0.0000    0.0000   36.0000  (181 steps, stepmode)
    2   3368 | o                          :
    8   3362 |      o                     :
    0   3210 |o                         :
    8   3098 |      o                :
    2   3272 + o                        :
    0   3278 |o                           :
    6   3256 |     o                    :
    4   3302 |   o                        :
    4   3276 |   o                      :
    0   3392 +o                             :
    4   3262 |   o                      :
    0   3212 |o                         :
    8   3216 |      o                  :
    0   3380 |o                           :
    0   3300 +o                         :
    0   3316 |o                         :
    4   3282 |   o                      :
```

```
0   3380 |o                :
6   3244 |   o           :
0   3130 +o             :
2   3340 | o              :
2   3180 | o            :
4   3258 |  o            :
4   3328 |  o             :
8   3330 +    o           :
4   3384 |  o              :
8   3476 |    o             :
6   3334 |   o            :
0   3348 |o                :
8   3246 +    o          :
6   3368 |   o             :
8   3390 |    o            :
2   3296 | o              :
4   3330 |  o             :
6   3266 +   o           :
0   3408 |o                 :
8   3252 |    o          :
2   3310 | o              :
0   3284 |o               :
0   3374 +o                :
4   3396 |  o              :
4   3240 |  o            :
2   3430 | o               :
0   3206 |o              :
4   3518 +  o               :
0   3278 |o               :
2   3396 | o               :
6   3450 |   o              :
4   3376 |  o              :
2   3340 + o              :
4   3430 |  o              :
0   3428 |o                 :
8   3368 |    o            :
6   3406 |   o             :
2   3218 + o             :
0   3374 |o                :
8   3448 |    o             :
4   3410 |  o              :
4   3378 |  o              :
6   3372 +   o             :
6   3146 |   o          :
```

```
 4  3328 |   o                             :
 0  3220 |o                              :
 8  3394 |       o                          :
 2  3384 + o                                :
 4  3242 |   o                           :
10  3318 |          o                      :
 6  3404 |     o                             :
 0  3350 |o                                 :
 6  3220 +     o                        :
 2  3334 | o                               :
 2  3394 | o                                :
 2  3208 | o                            :
 0  3276 |o                               :
14  3310 +              o                  :
10  3252 |          o                     :
 6  3342 |     o                           :
 4  3492 |   o                                 :
 2  3330 | o                               :
 6  3252 +     o                         :
 2  3300 | o                              :
 0  3252 |o                               :
 6  3330 |     o                           :
 0  3246 |o                               :
 4  3320 +   o                             :
 4  3358 |   o                             :
 4  3226 |   o                          :
 4  3276 |   o                           :
 6  3364 |     o                            :
 6  3328 +     o                           :
 0  3312 |o                                :
 6  3264 |     o                         :
 2  3258 | o                             :
 6  3264 |     o                         :
 2  3156 + o                           :
 0  3248 |o                               :
 0  3430 |o                                   :
12  3450 |            o                        :
 2  3320 | o                               :
 4  3286 +   o                            :
 0  3258 |o                               :
 6  3202 |     o                        :
 0  3288 |o                                :
 2  3370 | o                                :
 4  3110 +   o                        :
```

```
4   3274 |   o                    :
2   3148 | o                    :
6   3202 |     o                :
6   3436 |     o                    :
4   3318 +   o                  :
4   3164 |   o                :
4   3196 |   o                :
2   3190 | o                  :
4   3418 |   o                    :
0   3198 +o                     :
0   3312 |o                      :
4   3212 |   o                :
0   3300 |o                      :
6   3336 |     o                :
4   3198 +   o                :
2   3206 | o                  :
0   3266 |o                     :
2   3240 | o                  :
4   3378 |   o                    :
4   3270 +   o                :
2   3240 | o                  :
2   3172 | o                :
2   3232 | o                :
2   3224 | o                :
8   3330 +       o              :
8   3288 |       o            :
6   3278 |     o              :
8   3266 |       o            :
0   3224 |o                   :
4   3302 +   o                :
6   3220 |     o              :
2   3168 | o                :
6   3244 |     o              :
0   3288 |o                     :
2   3292 + o                  :
4   3296 |   o                :
0   3266 |o                   :
2   3280 | o                  :
2   3180 | o                :
8   3308 +       o            :
0   3200 |o                   :
4   3202 |   o                :
4   3204 |   o                :
6   3338 |     o                :
```

```
    8   3302 +       o                    :
    8   3186 |       o                :
    0   3202 |o                        :
    0   3300 |o                          :
    4   3364 |   o                        :
    0   3314 +o                          :
    4   3128 |   o                   :
    2   3252 | o                       :
    6   3222 |     o                 :
    4   3216 |   o                   :
    2   3164 + o                    :
    6   3252 |     o                 :
    0   3282 |o                          :
    2   3260 | o                       :
    2   3344 | o                         :
    0   3310 +o                          :
    0   3230 |o                        :
    6   3404 |     o                      :
    8   3168 |       o                :
    4   3298 |   o                     :
    0   3358 +o                           :
    2   3286 | o                       :
    4   3308 |   o                     :
    2   3290 | o                       :
   10   3382 |        o                  :
    4   3376 +   o                       :
    4   3310 |   o                     :
    0   3304 |o                          :
    2   3388 | o                          :
    6   3220 |     o                 :
    0   3196 +o                        :
    8   3396 |       o                   :
Centre at point  91.000      Angles =    24.1874   12.0937    0.0000  -72.0000
I(int)=    11.35  Sigma=38.44  FWHM=4.7450  Imax( 75)=    10.73  Temp=   0.00K
** Int. too low
# ph -108
# ss
Scan centre =    24.1874   12.0937    0.0000 -108.0000
Scan range  =     0.0000    0.0000    0.0000   36.0000  (181 steps, stepmode)
    2   3360 | o                          :
    6   3318 |     o                      :
    2   3400 | o                           :
    0   3346 |o                            :
    6   3228 +     o                   :
```

```
 2 3372 | o                :
 2 3282 | o              :
 0 3328 |o                 :
 4 3312 |  o              :
 8 3242 +    o           :
 8 3448 |    o             :
 2 3300 | o               :
 6 3372 |   o              :
 6 3374 |   o              :
 4 3340 +  o              :
 4 3160 |  o            :
 4 3336 |  o              :
 0 3448 |o                  :
 6 3290 |   o             :
 2 3324 + o               :
 4 3208 |  o             :
 0 3254 |o                :
 4 3344 |  o              :
 2 3248 | o              :
 2 3256 + o              :
 0 3206 |o               :
 4 3310 |  o              :
 2 3312 | o               :
 2 3086 | o            :
 2 3220 + o              :
 2 3252 | o              :
 4 3198 |  o             :
 8 3196 |    o           :
 0 3064 |o              :
 2 3432 + o                :
 0 3122 |o              :
 4 3144 |  o            :
 2 3306 | o               :
10 3244 |     o          :
 4 3340 +  o              :
 4 3066 |  o           :
 0 3238 |o               :
 8 3100 |    o         :
 4 3182 |  o            :
 6 3232 +   o            :
 2 3236 | o              :
 8 3276 |    o           :
 2 3354 | o               :
 2 3184 | o             :
```

```
0   3078 +o                    :
2   3130 | o                   :
2   3298 | o                       :
0   3208 |o                       :
0   3306 |o                        :
8   3216 +      o                 :
2   3234 | o                     :
2   3202 | o                     :
2   3248 | o                      :
4   3108 |   o                 :
2   3224 + o                     :
4   3206 |   o                   :
0   3356 |o                          :
0   3248 |o                        :
6   3148 |     o               :
4   3166 +   o                  :
0   3180 |o                      :
6   3268 |     o                  :
0   3270 |o                        :
0   3154 |o                      :
2   3254 + o                      :
0   3220 |o                       :
4   3364 |   o                     :
2   3238 | o                     :
2   3320 | o                       :
2   3276 + o                      :
0   3250 |o                        :
4   3236 |   o                   :
4   3332 |   o                     :
6   3202 |     o                 :
2   3216 + o                     :
4   3258 |   o                    :
4   3240 |   o                    :
8   3280 |      o                 :
8   3234 |      o                :
0   3176 +o                      :
0   3380 |o                           :
6   3294 |     o                   :
2   3248 | o                      :
4   3230 |   o                   :
4   3184 +   o                  :
6   3154 |     o                :
0   3214 |o                       :
0   3374 |o                           :
```

2 3284 | o :
2 3028 + o :
8 3186 | o :
8 3306 | o :
6 3356 | o :
2 3234 | o :
0 3228 +o :
8 3252 | o :
4 3206 | o :
2 3258 | o :
6 3284 | o :
2 3278 + o :
8 3200 | o :
0 3212 |o :
0 3218 |o :
4 3148 | o :
2 3158 + o :
4 3060 | o :
4 3346 | o :
2 3354 | o :
4 3162 | o :
4 3386 + o :
2 3114 | o :
8 3058 | o :
0 3258 |o :
2 3222 | o :
10 3150 + o :
0 3310 |o :
4 3190 | o :
2 3242 | o :
2 3140 | o :
10 3224 + o :
2 3228 | o :
6 3310 | o :
4 3324 | o :
2 3336 | o :
6 3170 + o :
2 3174 | o :
2 3100 | o :
6 3220 | o :
8 3256 | o :
0 3208 +o :
4 3286 | o :
4 3450 | o :

```
4  3150 |  o            :
2  3296 | o               :
2  3330 + o                :
2  3214 | o               :
8  3262 |    o            :
2  3314 | o               :
8  3238 |    o            :
2  3292 + o                :
6  3246 |   o             :
4  3206 |  o              :
2  3124 | o              :
4  3294 |  o              :
6  3178 +   o            :
0  3148 |o               :
0  3268 |o                :
4  3432 |  o                :
8  3210 |    o            :
8  3342 +    o             :
0  3212 |o                :
2  3342 | o                :
0  3112 |o               :
4  3322 |  o              :
2  3354 + o                :
2  3180 | o              :
0  3144 |o               :
12  3198 |      o         :
4  3044 |  o           :
2  3164 + o              :
2  3330 | o                :
0  3368 |o                 :
4  3384 |  o               :
4  3270 |  o              :
4  3384 +  o               :
2  3276 | o               :
2  3004 | o           :
6  3412 |   o              :
8  3286 |    o             :
4  3250 +  o              :
2  3406 | o                :
2  3248 | o               :
2  3138 | o              :
10  3200 |     o          :
4  3228 +  o             :
8  3082 |    o          :
```

```
Centre at point  14.444     Angles =    24.1874   12.0937    0.0000 -123.3112
I(int)=    -1.89  Sigma=39.45  FWHM=4.6490  Imax(163)=     8.28  Temp=   0.00K

# ph -75
# dr
# co 1 5
   53  16278
# co 5 1
    1   3185     14   3321     15   3348      9   3253     15   3163
Average Intensity =      10.800 cps, standard deviation =     5.381 cps
Average Monitor   =    3254.000 cps, standard deviation =    72.620 cps
# ch 1
# dr
# co 5 1
   10   3342      7   3282     11   3214     10   3237     10   3149
Average Intensity =       9.600 cps, standard deviation =     1.356 cps
Average Monitor   =    3244.800 cps, standard deviation =    64.836 cps
# ch 2
# dr
# co 5 1
    2   3256      4   3231      2   3246      9   3184      5   3258
Average Intensity =       4.400 cps, standard deviation =     2.577 cps
Average Monitor   =    3235.000 cps, standard deviation =    27.232 cps
# ch -1
# dr
# co 5 1
    7   3283      4   3233      2   3333      2   3167      4   3310
Average Intensity =       3.800 cps, standard deviation =     1.833 cps
Average Monitor   =    3265.200 cps, standard deviation =    59.331 cps
# ch 0.5
# dr
# co 5 1
    8   6290     17   6146     13   6114      8   6145     13   6201
Average Intensity =      11.800 cps, standard deviation =     3.429 cps
Average Monitor   =    6179.200 cps, standard deviation =    62.088 cps
# sv
Step scan parameters
 Nstep     :  181 ? 21
 Stepwidths:   0.0000   0.0000   0.0000   0.2000 ? 0 0 0.1 0
 Time/step :   0.50 sec ?

Data stored in chon.pm4
# ss
Scan centre =    24.1870   12.0935    0.5000  -75.0000
```

```
Scan range  =    0.0000    0.0000    2.0000    0.0000  ( 21 steps, stepmode)
   10   6076 |        o                   :
    6   5940 |     o                   :
   10   5942 |        o               :
   14   6120 |           o               :
   10   5974 +        o                 :
   16   6146 |            o              :
    8   6030 |      o                   :
   12   6134 |         o                 :
   16   6086 |            o             :
    6   5916 +     o                   :
    6   5866 |     o                 :
    6   6054 |     o                     :
    6   5876 |     o                  :
   10   6170 |        o                    :
    6   5932 +     o                   :
   14   6036 |           o              :
    4   5790 |   o                  :
    2   5872 | o                     :
    6   6116 |     o                       :
    8   6056 +      o                   :
   10   5812 |        o             :
Centre at point   1.000      Angles =    24.1870   12.0935   -0.5000  -75.0000
I(int)=     1.10  Sigma=19.42  FWHM=0.3407  Imax(  9)=     7.59  Temp=   0.00K
** Unsuitable scanwidth
# co 1 1
    0     4
# ch 0 0
# dr
# ca 0 0 4
Angles =    14.1465    7.0733   73.7600    0.0000
# ch 0
# dr
# sv
Step scan parameters
 Nstep     :   21 ? 181
 Stepwidths:   0.0000   0.0000   0.1000   0.0000 ? 0 0 0 0.5
 Time/step :   0.50 sec ?

Data stored in chon.pm4
# sv
Step scan parameters
 Nstep     :  181 ? 121
 Stepwidths:   0.0000   0.0000   0.0000   0.5000 ?
```

```
 Time/step :   0.50 sec ?

Data stored in chon.pm4
# pp
     14.1465    7.0732   -0.0000    0.0000
# ss
Scan centre =    14.1465    7.0732    0.0000    0.0000
Scan range  =     0.0000    0.0000    0.0000   60.0000  (121 steps, stepmode)
     0     0 |o                      :
     0     2 |o                       :
     0     0 |o                      :
     0     0 |o                      :
     0     8 +o                          :
     0     2 |o                       :
     0     0 |o                      :
     0     2 |o                       :
     2     0 | o                     :
     0     2 +o                       :
     0     0 |o                      :
     0     0 |o                      :
     0     4 |o                        :
     0     2 |o                       :
     0     0 +o                      :
     0     4 |o                        :
     0     4 |o                        :
     0     6 |o                         :
     0     4 |o                        :
     0     0 +o                      :
     0     0 |o                      :
     0     2 |o                       :
     0     4 |o                        :
     0     0 |o                      :
     0     2 +o                       :
     2     0 | o                     :
     0     2 |o                       :
     0     0 |o                      :
     4     0 |   o                   :
     0     0 +o                      :
     0     0 |o                      :
     2     0 | o                     :
     0     6 |o                         :
     2     0 | o                     :
     0     0 +o                      :
```

```
2    0 | o                   :
2    0 | o                   :
0    2 |o                     :
0    0 |o                    :
0    4 +o                      :
0    2 |o                     :
2    4 | o                    :
0    0 |o                    :
0    2 |o                     :
4    2 +   o                 :
0    0 |o                    :
2    2 | o                   :
0    0 |o                    :
0    0 |o                    :
0    4 +o                      :
0    2 |o                     :
0    0 |o                    :
0    0 |o                    :
0    2 |o                     :
0    0 +o                    :
0    0 |o                    :
4    0 |   o                :
0    2 |o                     :
0    0 |o                    :
0    4 +o                      :
0    2 |o                     :
0    2 |o                     :
0    4 |o                      :
0    0 |o                    :
0    2 +o                     :
0    2 |o                     :
0    2 |o                     :
0    2 |o                     :
0    0 |o                    :
0    0 +o                    :
0    0 |o                    :
0    0 |o                    :
2    0 | o                  :
0    4 |o                      :
0    0 +o                    :
0    2 |o                     :
0    0 |o                    :
2    2 | o                   :
0    0 |o                    :
```

```
 0    2 +o                   :
 2    2 | o                  :
 0    4 |o                    :
 0    0 |o                   :
 0    4 |o                    :
 0    4 +o                    :
 0    0 |o                   :
 0    4 |o                    :
 0    0 |o                   :
 0    4 |o                    :
 0    2 +o                   :
 0    2 |o                   :
 0    0 |o                   :
 0    0 |o                   :
 0    4 |o                    :
 2    6 + o                   :
 0    4 |o                    :
 0    2 |o                   :
 0    2 |o                   :
 0    0 |o                   :
 2    0 + o                 :
 0    6 |o                     :
 0    2 |o                   :
 2    2 | o                  :
 0    0 |o                   :
 0    4 +o                    :
 0    8 |o                      :
 2    0 | o                 :
 2    0 | o                 :
 0    2 |o                   :
 2    4 + o                  :
 2    0 | o                 :
 0    0 |o                   :
 2    0 | o                 :
 0    0 |o                   :
 0    0 +o                   :
 0    0 |o                   :
 0    0 |o                   :
 0    0 |o                   :
 0    2 |o                   :
 0    4 +o                    :
 0    0 |o                   :
```

Centre at point   1.000   Angles =   14.1465   7.0732   0.0000 -30.0000

I(int)=   3.83 Sigma=10.61 FWHM=4.0008 Imax(29)=   3.81 Temp=   0.00K

```
** Unsuitable scanwidth
# sv
Step scan parameters
 Nstep    :  121 ? 241
 Stepwidths:   0.0000   0.0000   0.0000   0.5000 ?

 Time/step :   0.50 sec ?

Data stored in chon.pm4
# ph 0
# dr
# ss
Scan centre =    14.1465    7.0730    0.0000    0.0000
Scan range  =     0.0000    0.0000    0.0000  120.0000  (241 steps, stepmode)
   10   5470 |        o                  :
    2   5276 | o                        :
    8   5364 |      o                    :
    6   5060 |    o                     :
    8   4976 +      o                   :
    2   4958 | o                        :
    8   5044 |      o                   :
   10   5058 |        o                 :
    2   5044 | o                        :
   10   4878 +        o                 :
   12   4808 |          o               :
    8   4900 |      o                   :
    6   4784 |    o                     :
    4   5002 |  o                       :
    6   4706 +    o                     :
    2   4560 | o                       :
    4   4776 |  o                       :
    2   4656 | o                        :
    8   4492 |      o                  :
    4   4544 +  o                      :
    4   4262 |  o                     :
    6   4310 |    o                   :
    4   4296 |  o                     :
    8   4256 |      o                 :
    2   4168 + o                      :
    0   4102 |o                       :
    6   4142 |    o                   :
    4   4090 |  o                    :
    4   4096 |  o                    :
    8   4068 +      o                :
```

```
 2   6152 | o                        :
 4   6362 |   o                      :
 6   6144 |     o                    :
10   6266 |         o                 :
 6   6406 +     o                    :
 6   6250 |     o                    :
 4   6272 |   o                      :
 6   6134 |     o                    :
12   6244 |           o               :
 6   6174 +     o                    :
 6   6260 |     o                    :
 2   6392 | o                        :
 8   6052 |       o                  :
 6   6188 |     o                    :
 8   6154 +       o                  :
12   6274 |           o               :
 6   6134 |     o                    :
 4   6186 |   o                      :
 4   6212 |   o                      :
12   6122 +           o              :
 6   6246 |     o                    :
10   5994 |         o                :
 6   6050 |     o                    :
 8   6126 |       o                  :
 8   6092 +       o                  :
10   6210 |         o                :
 8   6086 |       o                  :
 8   6126 |       o                  :
 0   6236 |o                          :
 2   6070 + o                        :
 6   6096 |     o                    :
 0   6068 |o                         :
 2   6214 | o                        :
12   6132 |           o              :
 4   6144 +   o                      :
 2   6036 | o                        :
10   6134 |         o                :
 4   6394 |   o                      :
 2   5986 | o                       :
 6   5944 +     o                   :
 6   6322 |     o                    :
 4   6042 |   o                      :
 0   6052 |o                         :
 2   6144 | o                        :
```

```
12  6110 +      o               :
 4  5952 |  o                   :
 4  6170 |  o                   :
 6  6148 |   o                  :
 8  6212 |    o                 :
 2  6058 + o                    :
 6  6052 |   o                  :
 4  6208 |  o                   :
 4  6242 |  o                   :
12  6124 |      o               :
 4  6070 +  o                   :
 6  6082 |   o                  :
 6  6116 |   o                  :
 6  6254 |   o                  :
12  5972 |      o               :
 6  6008 +   o                  :
12  6220 |      o               :
 6  6104 |   o                  :
 6  6106 |   o                  :
 6  6224 |   o                  :
 8  6236 +    o                 :
 2  6118 | o                    :
16  6004 |        o             :
 6  6058 |   o                  :
 8  6186 |    o                 :
 8  6116 +    o                 :
 8  6376 |    o                 :
 8  6184 |    o                 :
12  6028 |      o               :
 4  6252 |  o                   :
 4  6150 +  o                   :
 8  6060 |    o                 :
 0  6126 |o                     :
 4  6166 |  o                   :
 4  6214 |  o                   :
 2  6084 + o                    :
 6  5954 |   o                  :
 8  6134 |    o                 :
 6  6070 |   o                  :
 2  6090 | o                    :
 4  6098 +  o                   :
 6  6052 |   o                  :
 4  6080 |  o                   :
 6  6158 |   o                  :
```

```
 4  6104 |  o               :
 6  6138 +   o              :
 8  6080 |    o             :
 2  5984 | o               :
14  5868 |       o         :
12  6036 |      o           :
 2  6160 + o                :
 8  6000 |    o             :
12  6048 |      o           :
 6  5832 |   o             :
 6  6174 |   o              :
 6  6062 +   o              :
 8  6014 |    o             :
10  5922 |     o           :
 6  6088 |   o              :
 2  6038 | o                :
12  5882 +      o          :
 6  5914 |   o             :
 2  6114 | o                :
14  6008 |       o          :
14  5888 |       o         :
 8  5972 +    o            :
 6  5976 |   o             :
 4  5930 |  o              :
10  5678 |     o          :
 8  5886 |    o            :
 6  5866 +   o             :
12  5780 |      o          :
12  5756 |      o         :
 6  5884 |   o             :
 2  5782 | o               :
 8  5554 +    o           :
12  5626 |      o         :
12  5934 |      o          :
 6  5666 |   o            :
 4  5522 |  o             :
 8  5810 +    o            :
 4  5472 |  o             :
 4  5596 |  o             :
 2  5794 | o               :
 2  5616 | o              :
 2  5444 + o              :
 4  5562 |  o             :
 4  5524 |  o             :
```

```
 2  5470 | o               :
 6  5430 |   o             :
 4  6066 +  o               :
10  6294 |      o            :
 4  6092 |  o               :
 8  6302 |    o             :
 8  6182 |    o             :
 2  6112 + o                :
 4  6250 |  o               :
 8  6218 |    o             :
28  6158 |               o   :
14  6360 |        o          :
14  6344 +        o          :
10  6206 |      o           :
10  6158 |      o           :
 8  6202 |    o             :
 4  6196 |  o               :
 4  6228 +  o               :
 6  6242 |   o              :
10  6160 |      o           :
 6  6290 |   o              :
 8  6152 |    o             :
 6  6254 +   o              :
 8  6288 |    o             :
16  6240 |         o         :
10  6206 |      o           :
 4  6274 |  o               :
 4  6326 +  o               :
 0  6402 |o                  :
16  6348 |         o         :
 4  6186 |  o               :
 0  6292 |o                  :
 6  6230 +   o              :
 8  5998 |    o             :
 6  6206 |   o              :
 6  6126 |   o              :
 6  6362 |   o              :
12  6136 +       o           :
 8  6120 |    o             :
 8  6204 |    o             :
 6  6016 |   o              :
12  6134 |       o           :
 8  6320 +    o             :
 4  6234 |  o               :
```

```
   6   6068 |     o                        :
  12   6180 |          o                   :
  10   5998 |        o                     :
   8   6070 +      o                       :
   2   6092 | o                            :
  12   6278 |          o                    :
  10   6226 |        o                     :
   4   6106 |   o                          :
   4   5914 +   o                         :
  12   6048 |          o                   :
   0   5946 |o                             :
   2   5876 | o                           :
   2   5964 | o                           :
   6   5896 +     o                       :
  14   6222 |            o                 :
   2   6118 | o                            :
   6   6206 |     o                        :
   0   6166 |o                              :
   2   6114 + o                            :
   6   5992 |     o                       :
   6   6204 |     o                        :
   6   6046 |     o                        :
   4   5910 |   o                         :
   2   6122 + o                            :
   4   6116 |   o                          :
   0   5938 |o                             :
  10   6008 |        o                     :
   8   6036 |       o                      :
   6   6016 +     o                        :
   6   5862 |     o                       :
   0   6100 |o                              :
  10   6198 |        o                     :
   6   6076 |     o                        :
   4   6120 +   o                          :
   2   5980 | o                           :
Centre at point 121.000      Angles =    14.1465    7.0730    0.0000    0.0000
I(int)=    72.11  Sigma=60.08  FWHM=26.3966  Imax(173)=    22.21  Temp=   0.00K
** Unsuitable scanwidth
# ph 120
# ss
Scan centre =    14.1465    7.0730    0.0000  120.0000
Scan range  =     0.0000    0.0000    0.0000  120.0000  (241 steps, stepmode)
   4   6182 |   o                           :
   4   6238 |   o                           :
```

```
 4  5906 |  o                 :
 4  6156 |  o                 :
14  6210 +       o            :
 4  6072 |  o                 :
 4  6024 |  o                 :
 4  6078 |  o                 :
 4  6206 |  o                 :
 2  6280 + o                  :
 6  6068 |   o                :
 2  6406 | o                  :
 8  6032 |    o               :
 4  6154 |  o                 :
 2  6100 + o                  :
 8  6110 |    o               :
 4  6162 |  o                 :
10  6040 |     o              :
 6  5962 |   o                :
14  6010 +       o            :
 6  6186 |   o                :
10  6240 |     o              :
 6  6182 |   o                :
10  6088 |     o              :
 2  6206 + o                  :
 6  6190 |   o                :
10  6106 |     o              :
10  5884 |     o              :
 4  6020 |  o                 :
 8  6032 +    o               :
 4  6082 |  o                 :
 2  6190 | o                  :
 6  6338 |   o                :
 8  6054 |    o               :
 2  6256 + o                  :
 0  6342 |o                   :
 6  6146 |   o                :
 6  6086 |   o                :
 6  6030 |   o                :
 8  5858 +    o               :
 8  6110 |    o               :
 4  6122 |  o                 :
 2  6014 | o                  :
 4  6108 |  o                 :
 4  6182 +  o                 :
 2  6226 | o                  :
```

```
 2  6176 | o                 :
 6  6274 |   o               :
 4  6068 |  o               :
 8  5880 +    o             :
 4  6054 |  o               :
 6  6264 |   o               :
18  6022 |         o         :
 0  6160 |o                  :
 2  6114 + o               :
 2  5952 | o               :
 6  6154 |   o             :
 4  6168 |  o               :
 8  5936 |    o             :
 2  6010 + o               :
 8  5982 |    o             :
 8  6102 |    o             :
 4  6222 |  o               :
 6  6036 |   o             :
 8  5964 +    o             :
10  6290 |     o             :
12  6126 |      o           :
 6  6018 |   o             :
 4  6288 |  o               :
12  5968 +      o           :
10  6096 |     o           :
 8  6032 |    o             :
 4  5910 |  o               :
 6  5978 |   o             :
 6  6016 +   o             :
 6  6326 |   o               :
12  5874 |      o           :
10  6082 |     o           :
 4  6168 |  o               :
 4  6038 +  o               :
 4  6004 |  o               :
14  6248 |       o           :
 6  5890 |   o             :
 8  6066 |    o             :
 8  6164 +    o             :
 8  6038 |    o             :
 6  6144 |   o             :
 0  5906 |o                  :
 0  5968 |o                  :
 6  6210 +   o               :
```

```
 6  5970 |   o                  :
 6  6056 |   o                  :
 6  6024 |   o                  :
 4  5964 |  o                   :
 4  5974 +  o                   :
 6  5884 |   o                  :
 4  5798 |  o                   :
 4  6078 |  o                   :
 6  6000 |   o                  :
 2  6048 + o                    :
 8  6196 |    o                 :
 2  6078 | o                    :
 4  6050 |  o                   :
 2  5982 | o                    :
 4  6162 +  o                   :
 6  6032 |   o                  :
 4  5972 |  o                   :
 8  5992 |    o                 :
10  5970 |     o                :
 6  5964 +   o                  :
16  6058 |        o             :
 4  5932 |  o                   :
 4  5832 |  o                   :
 6  5986 |   o                  :
 4  6022 +  o                   :
 4  5784 |  o                   :
 6  5800 |   o                  :
 8  5810 |    o                 :
 8  5856 |    o                 :
 8  5876 +    o                 :
10  5908 |     o                :
10  5658 |     o                :
 6  5836 |   o                  :
 4  5704 |  o                   :
 0  5694 +o                     :
12  5754 |      o               :
 2  5648 | o                    :
 2  5668 | o                    :
 4  5612 |  o                   :
 2  5654 + o                    :
 6  5624 |   o                  :
 8  5646 |    o                 :
 2  5556 | o                    :
 8  5392 |    o                 :
```

```
 6   5428 +     o                      :
 4   5544 |   o                         :
12   5302 |          o                  :
 2   5390 | o                          :
 2   5466 | o                          :
 8   5312 +       o                    :
12   5132 |          o                 :
 4   5318 |   o                        :
 8   5118 |       o                   :
10   5086 |         o                 :
14   5180 +            o              :
 6   5004 |     o                     :
 6   5196 |     o                     :
 6   4904 |     o                     :
10   5062 |         o                 :
 2   4888 + o                        :
 4   4806 |   o                      :
 0   4808 |o                          :
 8   5000 |       o                   :
 4   4672 |   o                      :
 4   4618 +   o                      :
 6   4808 |     o                    :
 4   4822 |   o                      :
 6   4424 |     o                   :
 4   4518 |   o                     :
 4   4476 +   o                     :
 4   4498 |   o                     :
10   4612 |         o                 :
 8   4556 |       o                 :
10   4362 |         o                :
 6   4454 +     o                   :
 6   4220 |     o                  :
 4   4296 |   o                     :
12   4056 |          o              :
 2   4200 | o                      :
 2   3970 + o                      :
 8   3948 |       o                :
 2   4030 | o                      :
 8   3788 |       o               :
 6   3848 |     o                 :
 4   3920 +   o                   :
 2   3716 | o                     :
 6   3736 |     o                 :
 6   3886 |     o                 :
```

```
 4  3796 |   o                  :
 2  3768 + o                    :
 4  3662 |   o                  :
 6  3692 |     o                :
 0  3660 |o                     :
 4  3706 |   o                  :
 2  3602 + o                   :
14   3712 |           o          :
 6  3658 |     o                :
 4  3544 |   o                 :
 2  3468 | o                   :
 6  3462 +     o               :
 6  3464 |     o               :
 4  3580 |   o                 :
 2  3554 | o                   :
 8  3438 |       o             :
 2  3618 + o                   :
16   3356 |             o        :
 2  3410 | o                   :
 2  3642 | o                    :
 4  3396 |   o                 :
 8  3562 +       o             :
 0  3452 |o                     :
10   3532 |        o            :
10   3406 |        o            :
 6  3486 |     o               :
 2  3480 + o                   :
 2  3354 | o                   :
10   3520 |        o            :
 6  3454 |     o               :
 2  3450 | o                   :
 2  3468 + o                   :
 2  3408 | o                   :
 6  3424 |     o               :
 4  3498 |   o                 :
 2  3376 | o                   :
 8  3500 +       o             :
 2  3230 | o                  :
10   3230 |        o           :
 2  3440 | o                   :
 2  3566 | o                   :
 2  3304 + o                  :
10   3296 |        o           :
 2  3300 | o                  :
```

```
   6   3492 |     o                 :
   2   3304 | o                    :
  12   3386 +         o            :
  12   3456 |         o            :
   6   3412 |     o                :
   4   3300 |   o                  :
   8   3326 |      o               :
   4   3438 +   o                  :
   4   3636 |   o                   :
   2   3330 | o                    :
   6   3406 |     o                :
   4   3362 |   o                  :
   6   3472 +     o                :
   2   3400 | o                    :
  14   3538 |           o          :
  10   3336 |        o             :
   0   3382 |o                     :
  10   3458 +        o             :
   4   3410 |   o                  :
Centre at point  45.530      Angles =    14.1465    7.0730    0.0000   82.2648
I(int)=     7.19  Sigma=58.22  FWHM=16.7996  Imax( 53)=    12.26  Temp=   0.00K

# ph -120
# ss
Scan centre =    14.1465    7.0730    0.0000 -120.0000
Scan range  =     0.0000    0.0000    0.0000  120.0000  (241 steps, stepmode)
  10   3194 |        o             :
  10   3298 |        o             :
   6   3344 |     o                :
   2   3236 | o                    :
   4   3282 +   o                  :
   4   3362 |   o                  :
   4   3310 |   o                  :
   0   3414 |o                     :
  12   3398 |         o            :
   4   3138 +   o                  :
   6   3460 |     o                :
   2   3240 | o                    :
  12   3286 |         o            :
   6   3140 |     o                :
   2   3292 + o                    :
   6   3416 |     o                :
   4   3256 |   o                  :
   2   3420 | o                    :
```

```
 4 3342 |  o           :
 6 3304 +   o          :
 2 3208 | o            :
 4 3288 |  o           :
 4 3378 |  o           :
 6 3406 |   o          :
 6 3210 +   o          :
 6 3384 |   o          :
 8 3158 |    o         :
 4 3318 |  o           :
12 3356 |      o       :
 6 3374 +   o          :
 8 3252 |    o         :
 2 3312 | o            :
 6 3352 |   o          :
10 3206 |     o        :
 8 3334 +    o         :
 2 3324 | o            :
 4 3328 |  o           :
 2 3382 | o            :
 8 3190 |    o         :
 4 3414 +  o           :
10 3344 |     o        :
 0 3344 |o             :
 8 3326 |    o         :
 2 3050 | o            :
 8 3356 +    o         :
 2 3268 | o            :
10 3256 |     o        :
 4 3206 |  o           :
 4 3104 |  o           :
 6 3166 +   o          :
 6 3178 |   o          :
 8 3206 |    o         :
24 3172 |            o :
16 3220 |        o     :
 4 3162 +  o           :
12 3064 |      o       :
 4 3226 |  o           :
14 3256 |       o      :
 4 3218 |  o           :
 2 3164 + o            :
 2 3220 | o            :
 6 3198 |   o          :
```

```
 2   3342 | o               :
 8   3312 |    o            :
 6   3246 +   o             :
 2   3166 | o               :
 4   3284 |  o              :
 4   3308 |  o              :
 6   3284 |   o             :
 6   3376 +   o             :
 2   3210 | o               :
 6   3280 |   o             :
 0   3194 |o                :
10   3364 |     o           :
 2   3402 + o               :
 2   3314 | o               :
 4   3274 |  o              :
 2   3426 | o               :
 4   3312 |  o              :
 4   3312 +  o              :
 4   3196 |  o              :
 8   3228 |    o            :
 6   3274 |   o             :
 2   3330 | o               :
 6   3220 +   o             :
 6   3206 |   o             :
 8   3334 |    o            :
 6   3244 |   o             :
10   3242 |     o           :
 8   3420 +    o            :
 4   3296 |  o              :
 6   3276 |   o             :
 4   3336 |  o              :
 8   3416 |    o            :
 6   3458 +   o             :
10   3302 |     o           :
14   3200 |       o         :
 4   3220 |  o              :
 0   3350 |o                :
18   3142 +         o       :
 4   6148 |  o                   :
 6   6116 |   o                  :
 8   6294 |    o                 :
 2   6294 | o                    :
 6   6122 +   o                  :
 0   6182 |o                     :
```

```
12   6340 |          o                   :
 4   6136 |   o                         :
 4   6046 |   o                         :
 6   6156 +     o                       :
 2   6118 | o                           :
 8   6052 |       o                     :
 6   6338 |     o                        :
 2   6246 | o                            :
10   6026 +        o                     :
 4   6058 |   o                         :
 2   6226 | o                            :
 4   6246 |   o                          :
 0   6182 |o                              :
10   6408 +        o                      :
 4   6088 |   o                         :
12   6084 |          o                  :
 2   6172 | o                            :
10   6262 |        o                     :
 6   6172 +     o                        :
 4   6150 |   o                         :
 8   6168 |       o                      :
 6   6218 |     o                        :
12   6098 |          o                  :
 2   6208 + o                            :
 4   6178 |   o                          :
 8   6092 |       o                     :
 6   6140 |     o                       :
 8   6154 |       o                     :
 6   6294 +     o                        :
 8   6198 |       o                      :
 4   6128 |   o                         :
 4   6152 |   o                         :
 4   6184 |   o                          :
 8   6190 +       o                      :
 4   6216 |   o                          :
14   6094 |            o                 :
 6   6192 |     o                        :
 0   6162 |o                              :
10   6230 +        o                      :
 6   6048 |     o                       :
14   6116 |            o                 :
 2   6222 | o                            :
10   6158 |        o                     :
 6   6080 +     o                       :
```

```
 8  6206 |    o            :
 4  6138 |  o              :
 8  6240 |    o            :
 2  5978 | o               :
 2  6116 + o               :
10  6128 |     o           :
 4  6140 |  o              :
 2  5918 | o               :
 2  6032 | o               :
14  6250 +       o         :
 2  5996 | o               :
 6  5932 |   o             :
10  6242 |     o           :
 6  6336 |   o             :
 6  5976 +   o             :
10  5888 |     o           :
10  6152 |     o           :
14  6052 |       o         :
10  5954 |     o           :
 6  5878 +   o             :
10  5952 |     o           :
 4  6054 |  o              :
 6  6094 |   o             :
14  6064 |       o         :
 2  6008 + o               :
10  6040 |     o           :
 8  6076 |    o            :
 6  6004 |   o             :
 6  5872 |   o             :
12  6162 +      o          :
 6  5872 |   o             :
 4  6108 |  o              :
 4  6100 |  o              :
 4  6102 |  o              :
 2  6228 + o               :
12  6026 |      o          :
 4  6088 |  o              :
12  6348 |      o          :
12  6026 |      o          :
14  6352 +       o         :
14  6000 |       o         :
 6  6070 |   o             :
 8  6224 |    o            :
 2  6050 | o               :
```

```
 4  5954 +  o                :
 6  6090 |   o               :
 4  5962 |  o                :
 2  5944 | o                 :
 6  6106 |   o               :
 6  5914 +   o               :
 8  6026 |    o              :
 0  6002 |o                   :
14  6132 |       o            :
 2  5868 | o                 :
 8  6026 +    o              :
12  6064 |      o             :
 6  5826 |   o               :
 8  5976 |    o              :
 6  6006 |   o               :
12  6044 +      o             :
12  5916 |      o             :
 2  5822 | o                 :
 4  5922 |  o                :
 2  5800 | o                :
 2  6020 + o                 :
 2  5916 | o                 :
 8  5762 |    o             :
 8  5838 |    o              :
 0  6004 |o                   :
 2  5728 + o                :
 6  5902 |   o               :
 4  5728 |  o               :
10  5668 |     o              :
 2  5946 | o                 :
 6  5594 +   o              :
10  5846 |     o               :
 8  5714 |    o             :
 8  5714 |    o             :
 2  5482 | o               :
10  5706 +     o              :
 2  5542 | o                :
 4  5594 |  o               :
 6  5316 |   o             :
16  5668 |        o           :
 6  5244 +   o             :
 6  5414 |   o             :
 0  5376 |o                 :
 4  5280 |  o              :
```

```
   10   5420 |         o                    :
    6   5344 +     o                        :
    8   5088 |       o                    :
Centre at point 121.000      Angles =    14.1465    7.0730    0.0000 -120.0000
I(int)=    45.11  Sigma=59.63  FWHM=67.1172  Imax( 53)=    18.44  Temp=   0.00K
** Int. too low
# ph 26.5
# dr
# sv
Step scan parameters
 Nstep     :  241 ? 11
 Stepwidths:   0.0000   0.0000   0.0000   0.5000 ? 0.1 0 0 0
 Time/step :   0.50 sec ?

Data stored in chon.pm4
# ss
Scan centre =    14.1465    7.0730    0.0000   26.5000
Scan range  =     1.0000    0.0000    0.0000    0.0000  ( 11 steps, stepmode)
   20   3256 |                 o     :
   16   3302 |             o          :
    8   3264 |       o               :
   16   3348 |             o            :
   16   3318 +             o           :
   12   3412 |          o                  :
   16   3268 |             o         :
    8   3464 |       o                       :
    6   3290 |     o                  :
   12   3310 +          o               :
    4   3300 |   o                    :
Centre at point  11.000      Angles =    14.6465    7.0730    0.0000   26.5000
I(int)=     0.20  Sigma=13.43  FWHM=0.1421  Imax( 10)=     6.40  Temp=   0.00K
** Unsuitable scanwidth
# th 14.1465
# dr
# sv
Step scan parameters
 Nstep     :   11 ? 21
 Stepwidths:   0.1000   0.0000   0.0000   0.0000 ? 0 0 0.2 0
 Time/step :   0.50 sec ?

Data stored in chon.pm4
# ss
Scan centre =    14.1465    7.0730    0.0000   26.5000
Scan range  =     0.0000    0.0000    4.0000    0.0000  ( 21 steps, stepmode)
```

```
   4   3206 |   o                   :
  10   3434 |        o                    :
  12   3382 |          o                 :
  10   3244 |        o                :
   4   3276 +   o                     :
  14   3306 |            o              :
   8   3268 |      o                  :
  10   3322 |        o                  :
  24   3444 |                    o         :
   4   3298 +   o                      :
  18   3430 |               o              :
  16   3348 |             o             :
  16   3140 |             o       :
  10   3254 |        o                :
  14   3276 +            o             :
  12   3294 |          o               :
  20   3308 |                o         :
  10   3326 |        o                  :
  14   3416 |            o                 :
   6   3240 +     o                   :
   6   3290 |     o                    :
```

Centre at point 11.000 Angles = 14.1465 7.0730 0.0000 26.5000

I(int)= 12.00 Sigma=20.35 FWHM=0.1905 Imax(9)= 15.33 Temp= 0.00K

** Int. too low

ch 0

dr

sv

Step scan parameters

Nstep : 21 ?

Stepwidths: 0.0000 0.0000 0.2000 0.0000 ? 0.2 0 0 0

Time/step : 0.50 sec ?

Data stored in chon.pm4

ss

Scan centre = 14.1465 7.0730 0.0000 26.5000

Scan range = 4.0000 0.0000 0.0000 0.0000 (21 steps, stepmode)

```
   4   6046 |   o                     :
   6   6192 |     o                      :
   4   6140 |   o                       :
   6   6064 |     o                    :
   6   6150 +     o                      :
   6   6116 |     o                     :
  18   6120 |               o           :
```

```
  20   6092 |                 o          :
  16   5846 |             o        :
  18   5996 +              o         :
   6   6114 |     o                      :
   6   6072 |     o                     :
   8   6252 |       o                       :
   2   6118 | o                          :
   8   6038 +       o                   :
   8   6196 |       o                       :
   8   6122 |       o                    :
   6   6062 |     o                     :
   4   6084 |   o                       :
   6   5944 +     o                   :
   2   6054 | o                          :
Centre at point  11.000      Angles =    14.1465    7.0730    0.0000   26.5000
I(int)=    15.40  Sigma=15.03  FWHM=0.8954  Imax(  8)=    15.56  Temp=   0.00K
** Unsuitable scanwidth
# ca 0 4 0
Angles =    18.0158    9.0079    0.0000  -90.0000
# ph 0
# dr
# pp
     18.0158    9.0077   -0.0000    0.0000
# sv
Step scan parameters
 Nstep     :   21 ? 241
 Stepwidths:   0.2000   0.0000   0.0000   0.0000 ? 0 0 0 0.5
 Time/step :   0.50 sec ?

Data stored in chon.pm4
# ss
Scan centre =    18.0158    9.0077    0.0000    0.0000
Scan range  =     0.0000    0.0000    0.0000  120.0000  (241 steps, stepmode)
   6   5970 |     o                         :
   4   5984 |   o                           :
   6   5924 |     o                         :
   6   6016 |     o                         :
   4   5832 +   o                           :
   8   5970 |       o                       :
   4   5854 |   o                           :
   6   5762 |     o                        :
   4   5826 |   o                           :
   6   5708 +     o                        :
   2   5726 | o                            :
```

```
4  5852 |   o                                 :
8  5728 |       o                            :
8  5960 |       o                             :
2  5970 + o                                   :
4  5632 |   o                               :
2  5814 | o                                   :
4  5732 |   o                               :
4  5914 |   o                                :
4  5674 +   o                               :
6  5816 |     o                               :
4  5664 |   o                               :
2  5686 | o                                 :
2  5732 | o                                 :
8  5640 +       o                           :
0  5802 |o                                     :
2  5386 | o                                 :
2  5776 | o                                 :
4  5576 |   o                               :
8  5546 +       o                           :
4  5696 |   o                               :
0  5396 |o                                   :
2  5576 | o                                 :
8  5414 |       o                          :
2  5340 + o                                 :
2  5304 | o                                 :
4  5390 |   o                               :
2  5376 | o                                 :
6  5258 |     o                             :
4  5088 +   o                              :
2  5178 | o                                :
2  4992 | o                                :
0  5222 |o                                   :
2  5398 | o                                 :
4  5056 +   o                              :
2  4912 | o                                :
6  4884 |     o                           :
4  4956 |   o                              :
6  5028 |     o                            :
2  4972 + o                                :
0  4904 |o                                  :
4  4878 |   o                             :
4  4798 |   o                             :
8  4720 |       o                         :
4  4694 +   o                             :
```

```
0   5056 |o                           :
2   4686 | o                        :
2   4722 | o                        :
6   4532 |     o                   :
2   4738 + o                        :
6   4526 |     o                   :
4   4452 |   o                     :
2   4426 | o                       :
4   4434 |   o                     :
2   4256 + o                      :
4   4210 |   o                    :
2   4132 | o                      :
6   4166 |     o                  :
2   4104 | o                      :
6   3954 +     o                 :
4   3920 |   o                   :
2   3980 | o                     :
8   4006 |       o                :
6   4066 |     o                  :
2   3944 + o                     :
0   3656 |o                      :
6   3768 |     o                 :
2   3828 | o                     :
2   3730 | o                     :
4   3760 +   o                   :
6   3632 |     o                :
6   3644 |     o                :
8   3754 |       o               :
2   3688 | o                     :
0   3754 +o                       :
0   3546 |o                      :
6   3452 |     o                :
0   3604 |o                      :
4   3586 |   o                  :
4   3572 +   o                  :
0   3432 |o                      :
2   3482 | o                    :
6   3590 |     o                :
2   3482 | o                    :
6   3580 +     o                :
4   3294 |   o                 :
6   3608 |     o                :
4   3578 |   o                  :
4   3550 |   o                  :
```

```
 4  3430 +   o                   :
 4  3456 |   o                   :
 6  3366 |     o                 :
10   3588 |        o              :
 6  3486 |     o                 :
14   3592 +           o           :
 6  3442 |     o                 :
 2  3388 | o                     :
 4  3318 |   o                  :
 2  3372 | o                    :
 6  3496 +     o                 :
 8  3286 |       o              :
 2  3432 | o                     :
 4  3558 |   o                   :
 6  3412 |     o                 :
 2  3296 + o                    :
 6  3340 |     o                :
 2  3380 | o                    :
 6  3462 |     o                 :
 0  3478 |o                      :
 4  3388 +   o                   :
 2  3236 | o                    :
 4  3250 |   o                  :
10   3350 |        o             :
 4  3376 |   o                  :
 6  3326 +     o                :
 2  3298 | o                    :
 6  3306 |     o                :
 6  3270 |     o                :
 4  3422 |   o                   :
 2  3434 + o                     :
 6  3306 |     o                :
 2  3336 | o                    :
 4  3374 |   o                  :
10   3404 |        o              :
 0  3202 +o                      :
 4  3338 |   o                  :
 6  3336 |     o                :
 2  3224 | o                    :
 2  3198 | o                    :
 6  3268 +     o                :
 8  3366 |       o              :
 2  3324 | o                    :
 2  3380 | o                    :
```

```
 2   3368 | o                    :
 2   3376 + o                    :
 4   3308 |   o                  :
 4   3260 |   o                  :
 4   3352 |   o                  :
 4   3130 |   o                  :
 0   3100 +o                     :
 4   3212 |   o                  :
 4   3178 |   o                  :
 2   3366 | o                    :
 0   3434 |o                     :
12   3298 +          o           :
 2   3362 | o                    :
 6   3280 |     o                :
 0   3224 |o                     :
 0   3292 |o                     :
 4   3128 +   o                  :
 6   3328 |     o                :
 0   3280 |o                     :
 6   3182 |     o                :
 4   3246 |   o                  :
 4   3176 +   o                  :
 4   3182 |   o                  :
 2   3318 | o                    :
 0   3282 |o                     :
 2   3192 | o                    :
 0   3302 +o                     :
 0   3176 |o                     :
 2   3112 | o                    :
 6   3134 |     o                :
 2   3308 | o                    :
 2   3138 + o                    :
12   3294 |          o           :
 4   3422 |   o                  :
 0   3176 |o                     :
 2   3224 | o                    :
 4   3204 +   o                  :
 0   3234 |o                     :
 2   3404 | o                    :
 0   3210 |o                     :
 4   3358 |   o                  :
 4   3280 +   o                  :
 0   3010 |o                     :
 0   3222 |o                     :
```

```
 2   3430 | o                   :
 4   3220 |   o                 :
 2   3276 + o                   :
 4   3250 |   o                 :
 6   3370 |     o               :
 4   3350 |   o                 :
 6   3396 |     o               :
 4   3338 +   o                 :
10   3342 |         o           :
 2   3188 | o                   :
 0   3046 |o                    :
10   3308 |         o           :
 6   3304 +     o               :
 4   3374 |   o                 :
 2   3368 | o                   :
 4   3258 |   o                 :
10   3238 |         o           :
 4   3254 +   o                 :
 2   3266 | o                   :
 4   3324 |   o                 :
 4   3330 |   o                 :
 4   3236 |   o                 :
 8   3324 +       o             :
 4   3426 |   o                 :
 6   3196 |     o               :
 6   3316 |     o               :
 8   3300 |       o             :
 4   3166 +   o                 :
14   3388 |             o       :
 6   3248 |     o               :
 0   3306 |o                    :
 2   3258 | o                   :
 2   3302 + o                   :
 4   3290 |   o                 :
 8   3266 |       o             :
 2   3322 | o                   :
 2   3350 | o                   :
 8   3156 +       o             :
 4   3248 |   o                 :
 2   3294 | o                   :
 4   3398 |   o                 :
 4   3390 |   o                 :
 4   3234 +   o                 :
 6   3358 |     o               :
```

```
   2   3122 | o                    :
   4   3424 |   o                  :
   2   3188 | o                    :
  10   3376 +        o             :
   6   3294 |     o                :
   4   3240 |   o                  :
  12   3312 |          o           :
   8   3410 |       o              :
   0   3234 +o                     :
   2   3260 | o                    :
Centre at point 135.296     Angles =    18.0158    9.0077    0.0000    7.1482
I(int)=   -62.25  Sigma=50.75  FWHM=10.6885  Imax(105)=     9.55  Temp=   0.00K

# ph 120
# ss
Scan centre =    18.0158    9.0077    0.0000  120.0000
Scan range  =     0.0000    0.0000    0.0000  120.0000  (241 steps, stepmode)
   4   3124 |   o                  :
   8   3252 |       o              :
   0   3270 |o                     :
   6   3144 |     o                :
   4   3288 +   o                  :
   4   3116 |   o                  :
   6   3246 |     o                :
  14   3306 |            o         :
   2   3344 | o                    :
   6   3340 +     o                :
   8   3158 |       o              :
   4   3190 |   o                  :
   2   3266 | o                    :
   2   3186 | o                    :
   0   3244 +o                     :
   0   3218 |o                     :
   4   3166 |   o                  :
   2   3110 | o                    :
   6   3246 |     o                :
   4   3330 +   o                  :
  18   3166 |                o     :
   8   3302 |       o              :
   4   3266 |   o                  :
   2   3278 | o                    :
   4   3322 +   o                  :
   2   3266 | o                    :
   4   3286 |   o                  :
```

```
10  3422 |     o            :
 2  3246 | o                :
 6  3400 +   o              :
 4  3308 |  o               :
 0  3242 |o                 :
 6  3188 |   o              :
 4  3270 |  o               :
 6  3222 +   o              :
 2  3172 | o                :
 2  3268 | o                :
 0  3238 |o                 :
 0  3384 |o                 :
 8  3386 +    o             :
 4  3310 |  o               :
 4  3308 |  o               :
 2  3242 | o                :
 0  3420 |o                 :
 0  3344 +o                 :
10  3248 |     o            :
 0  3254 |o                 :
 6  3364 |   o              :
 6  3444 |   o              :
 2  3438 + o                :
10  3274 |     o            :
 2  3306 | o                :
 6  3346 |   o              :
 4  3356 |  o               :
 6  3330 +   o              :
 6  3310 |   o              :
 4  3308 |  o               :
 4  3274 |  o               :
 6  3270 |   o              :
 2  3234 + o                :
 2  3342 | o                :
 2  3182 | o                :
 4  3262 |  o               :
 2  3278 | o                :
 4  3262 +  o               :
 4  3538 |  o               :
 8  3382 |    o             :
 4  3242 |  o               :
 6  3130 |   o              :
 4  3278 +  o               :
 4  3256 |  o               :
```

```
10 3276 |     o             :
 4 3346 |  o                :
 0 3298 |o                  :
 0 3270 +o                  :
 2 3324 | o                 :
 2 3236 | o                 :
 0 3364 |o                  :
 2 3224 | o                 :
10 3230 +     o             :
 2 3274 | o                 :
 6 3248 |   o               :
 0 3298 |o                  :
 2 3278 | o                 :
 2 3238 + o                 :
 2 3304 | o                 :
12 3404 |      o            :
 2 3054 | o                 :
 6 3258 |   o               :
 0 3322 +o                  :
 4 3296 |  o                :
 0 3230 |o                  :
 0 3322 |o                  :
 0 3244 |o                  :
 6 3312 +   o               :
10 3276 |     o             :
 4 3224 |  o                :
 0 3304 |o                  :
 2 3248 | o                 :
 2 3156 + o                 :
 8 3334 |    o              :
 2 3146 | o                 :
 2 3288 | o                 :
 2 3252 | o                 :
 2 3310 + o                 :
 6 2982 |   o               :
 4 3282 |  o                :
 0 3318 |o                  :
 2 3208 | o                 :
12 3176 +      o            :
 4 3254 |  o                :
 2 3218 | o                 :
 2 3016 | o                 :
 2 3220 | o                 :
 8 3250 +    o              :
```

```
 2  3362 | o                :
 0  3224 |o                 :
 4  3344 |  o               :
 4  3300 |  o               :
14  3284 +         o        :
 0  3158 |o                 :
 8  3304 |    o             :
 4  3258 |  o               :
 2  3328 | o                :
 4  3286 +  o               :
 0  3142 |o                 :
 2  5852 | o                        :
 2  6032 | o                        :
 2  6192 | o                         :
 8  5942 +    o                     :
 4  5964 |  o                       :
 4  6068 |  o                       :
 8  5962 |    o                     :
 8  6166 |    o                      :
 6  6234 +   o                       :
10  6072 |      o                    :
 2  6048 | o                        :
 4  6142 |  o                        :
 0  6188 |o                           :
 2  6068 + o                        :
 4  6212 |  o                        :
 2  6126 | o                         :
 6  6272 |   o                       :
 8  6176 |    o                      :
 6  5998 +   o                      :
 2  6316 | o                         :
 2  6102 | o                         :
 2  6176 | o                         :
 4  6172 |  o                        :
 2  6078 + o                         :
 2  6126 | o                         :
 6  6232 |   o                       :
 6  6186 |   o                       :
 6  6208 |   o                       :
 6  6252 +   o                       :
 0  6172 |o                           :
 4  6146 |  o                        :
 6  6108 |   o                       :
 4  6150 |  o                        :
```

```
 2   6106 + o                        :
 4   6136 |   o                      :
 4   6156 |   o                      :
 0   6142 |o                          :
 4   6118 |   o                      :
 2   6208 + o                        :
 4   6000 |   o                     :
12   6002 |          o               :
 4   6250 |   o                      :
 4   6170 |   o                      :
 0   6056 +o                         :
 4   5990 |   o                     :
 2   6014 | o                       :
 0   6208 |o                          :
 6   6162 |     o                    :
 0   6090 +o                          :
 8   6136 |       o                  :
10   6260 |        o                  :
 6   6326 |     o                    :
12   6244 |          o                :
 2   6302 + o                        :
 2   6230 | o                        :
 6   6216 |     o                    :
 4   6158 |   o                      :
 8   6208 |       o                  :
 8   6080 +       o                  :
 6   6108 |     o                    :
 2   6140 | o                        :
 8   5994 |       o                 :
 2   6122 | o                        :
 0   6022 +o                         :
 6   5986 |     o                   :
 8   6028 |       o                 :
 8   5946 |       o                 :
 4   6230 |   o                      :
 4   5936 +   o                     :
 2   5980 | o                       :
 8   6044 |       o                 :
 2   6126 | o                        :
 0   6106 |o                          :
 6   6050 +     o                   :
 6   5834 |     o                   :
 4   6048 |   o                     :
 4   6158 |   o                      :
```

```
   2   5980 | o                      :
   2   6036 + o                      :
   0   6010 |o                       :
   2   6012 | o                      :
   2   6110 | o                      :
   0   6006 |o                       :
   0   6046 +o                       :
  10   6166 |        o               :
   2   5888 | o                      :
   4   5922 |   o                    :
   4   6040 |   o                    :
   2   6230 + o                      :
   8   5948 |       o                :
   0   5998 |o                       :
   4   5888 |   o                    :
   6   5996 |     o                  :
   8   5910 +       o                :
   0   6086 |o                       :
   0   5860 |o                       :
   4   5954 |   o                    :
   4   5892 |   o                    :
   2   6028 + o                      :
   0   5984 |o                       :
   2   5910 | o                      :
   4   6086 |   o                    :
   6   5864 |     o                  :
   2   5912 + o                      :
   4   6010 |   o                    :
   2   5930 | o                      :
   2   6054 | o                      :
  12   5930 |          o             :
   0   5838 +o                       :
   4   5826 |   o                    :
   4   5916 |   o                    :
   6   5906 |     o                  :
   6   5816 |     o                  :
   4   6086 +   o                    :
   4   5764 |   o                    :
Centre at point 241.000      Angles =    18.0158    9.0077    0.0000  180.0000
I(int)=    9.04  Sigma=48.71  FWHM=3.5944  Imax( 21)=    13.55  Temp=   0.00K
** Unsuitable scanwidth
# ca 5 0 0
Angles =    30.3643   15.1822    0.0000    0.0000
# dr
```

```
# ss
Scan centre =    30.3642   15.1820    0.0000    0.0000
Scan range  =     0.0000    0.0000    0.0000  120.0000  (241 steps, stepmode)
    6   4922 |     o                        :
    4   4836 |   o                          :
    2   4872 | o                            :
    2   4676 | o                           :
    4   4734 +   o                         :
    2   4882 | o                            :
    0   4686 |o                             :
    4   4550 |   o                        :
    0   4674 |o                             :
    0   4474 +o                            :
    2   4650 | o                           :
    8   4558 |       o                    :
    2   4244 | o                         :
    4   4368 |   o                       :
    2   4216 + o                         :
    2   4248 | o                         :
    2   4190 | o                         :
    6   4078 |     o                    :
    2   3932 | o                       :
    4   4010 +   o                      :
    2   4012 | o                        :
    2   3846 | o                       :
    4   3864 |   o                     :
   10   3786 |         o               :
    6   3934 +     o                   :
    2   3794 | o                       :
    4   3746 |   o                    :
    4   3786 |   o                    :
    2   3794 | o                       :
    2   3680 + o                      :
    0   3726 |o                        :
    4   3650 |   o                    :
    0   3510 |o                       :
    0   3642 |o                        :
    0   3652 +o                        :
    2   3622 | o                      :
    4   3566 |   o                   :
    6   3492 |     o                 :
    0   3498 |o                       :
    4   3492 +   o                   :
    4   3402 |   o                   :
```

```
 4   3288 |   o                    :
 2   3502 | o                      :
 6   3568 |     o                  :
 2   3360 + o                      :
 4   3546 |   o                    :
 8   3654 |       o                :
 6   3456 |     o                  :
 6   3596 |     o                  :
 0   3408 +o                       :
 0   3422 |o                       :
 2   3362 | o                      :
 4   3444 |   o                    :
 2   3484 | o                      :
 0   3470 +o                       :
 2   3418 | o                      :
 8   3230 |       o                :
 2   3472 | o                      :
 4   3274 |   o                    :
 2   3426 + o                      :
 2   3334 | o                      :
 4   3418 |   o                    :
 4   3480 |   o                    :
 8   3270 |       o                :
 8   3296 +       o                :
 2   3304 | o                      :
 4   3266 |   o                    :
 6   3302 |     o                  :
 0   3292 |o                       :
 2   3410 + o                      :
 2   3214 | o                      :
 8   3310 |       o                :
10   3364 |         o              :
 0   3410 |o                       :
 2   3306 + o                      :
 4   3380 |   o                    :
 0   3322 |o                       :
 6   3348 |     o                  :
 4   3262 |   o                    :
 4   3310 +   o                    :
 2   3284 | o                      :
 2   3568 | o                      :
 0   3222 |o                       :
 2   3416 | o                      :
 2   3288 + o                      :
```

```
10  3516 |        o                :
 8  3244 |      o                 :
 4  3286 |   o                    :
 0  3318 |o                        :
 2  3278 + o                      :
 6  3262 |     o                  :
 4  3288 |   o                    :
 6  3158 |     o                 :
 4  3258 |   o                    :
 0  3152 +o                       :
 6  3446 |     o                   :
 4  3386 |   o                    :
 2  3136 | o                     :
 0  3430 |o                         :
 2  3288 + o                      :
 4  3394 |   o                     :
 4  3286 |   o                    :
 6  3362 |     o                  :
 6  3314 |     o                  :
 2  3256 + o                      :
 6  3466 |     o                   :
 4  3356 |   o                    :
 4  3338 |   o                    :
 4  3326 |   o                    :
 6  3252 +     o                  :
 6  3276 |     o                  :
 0  3580 |o                         :
 2  3360 | o                      :
 8  3426 |      o                  :
 6  3378 +     o                  :
 0  3292 |o                        :
 2  3308 | o                      :
 4  3352 |   o                    :
 2  3234 | o                      :
 6  3416 +     o                   :
 4  3220 |   o                    :
 8  3306 |      o                 :
 0  3364 |o                        :
 4  3420 |   o                     :
 4  3318 +   o                    :
 2  3364 | o                      :
 4  3262 |   o                    :
 6  3384 |     o                  :
 6  3332 |     o                  :
```

```
 2   3288 + o                    :
 2   3250 | o                    :
 2   3288 | o                    :
 2   3294 | o                    :
 2   3262 | o                    :
 6   3446 +    o                 :
 4   3286 |   o                  :
 0   3344 |o                     :
 0   3318 |o                     :
 4   3256 |   o                  :
 4   3304 +   o                  :
 2   3276 | o                    :
 4   3262 |   o                  :
 2   3184 | o                   :
 4   3218 |   o                  :
 2   3436 + o                    :
 4   3226 |   o                  :
 2   3272 | o                    :
 4   3324 |   o                  :
 0   3190 |o                     :
 4   3354 +   o                  :
10   3208 |        o             :
 4   3230 |   o                  :
 2   3438 | o                    :
 4   2948 |   o                :
 4   3148 +   o                 :
 2   3272 | o                    :
 4   3228 |   o                  :
 2   3134 | o                   :
10   3214 |        o             :
 2   3158 + o                   :
 0   3164 |o                     :
 4   3228 |   o                  :
 8   3394 |      o               :
 0   3254 |o                     :
 2   3378 + o                    :
 2   3198 | o                    :
 2   3394 | o                    :
 4   3216 |   o                  :
 4   3274 |   o                  :
 0   3172 +o                     :
 0   3396 |o                      :
 2   3348 | o                    :
 2   3300 | o                    :
```

```
4   3274 |   o                    :
0   3250 +o                       :
4   3228 |   o                    :
2   3334 | o                      :
10  3362 |        o               :
0   3334 |o                       :
0   3456 +o                        :
2   3198 | o                      :
4   3328 |   o                    :
2   3146 | o                     :
4   3408 |   o                     :
4   3278 +   o                    :
0   3428 |o                        :
8   3198 |       o                :
0   3314 |o                       :
0   3332 |o                       :
4   3346 +   o                    :
0   3310 |o                       :
2   3196 | o                      :
0   3214 |o                       :
2   3256 | o                      :
2   3232 + o                      :
4   3184 |   o                   :
6   3340 |     o                  :
2   3238 | o                      :
8   3188 |       o               :
6   3238 +     o                  :
6   3208 |     o                  :
0   3236 |o                       :
0   3340 |o                       :
2   3258 | o                      :
8   3280 +       o                :
2   3432 | o                       :
6   3174 |     o                 :
4   3276 |   o                    :
0   3200 |o                       :
2   3328 + o                      :
8   3258 |       o                :
2   3316 | o                      :
0   3192 |o                       :
8   3274 |       o                :
8   3292 +       o                :
2   3206 | o                      :
2   3326 | o                      :
```

```
    2   3474 | o                    :
    6   3508 |     o                :
    0   3402 +o                      :
    2   3312 | o                  :
    4   3226 |   o                :
    2   3146 | o                :
    2   3184 | o                :
    2   3258 + o                 :
    6   3228 |     o              :
    4   3280 |   o                :
    4   3260 |   o                :
    6   3310 |     o              :
    0   3462 +o                    :
    4   3358 |   o                :
    0   3362 |o                    :
    4   3368 |   o                :
    4   3162 |   o              :
    4   3216 +   o               :
    4   3238 |   o                :
    4   3266 |   o                :
    2   3478 | o                   :
    2   3152 | o                :
    0   3378 +o                    :
    6   3380 |     o              :
Centre at point 121.000      Angles =    30.3642   15.1820    0.0000    0.0000
I(int)=    29.48  Sigma=43.46  FWHM=4.2053  Imax( 24)=     7.09  Temp=   0.00K
** Int. too low
# ph 120
# ss
Scan centre =    30.3642   15.1820    0.0000  120.0000
Scan range  =     0.0000    0.0000    0.0000  120.0000  (241 steps, stepmode)
    4   3264 |   o              :
    0   3164 |o                  :
    4   3272 |   o              :
    6   3486 |     o              :
    0   3254 +o                  :
    4   3070 |   o            :
    0   3328 |o                  :
    4   3366 |   o              :
    2   3356 | o                :
    2   3308 + o                :
    2   3338 | o                :
    4   3290 |   o              :
    0   3184 |o                  :
```

```
4   3198 |   o                  :
0   3250 +o                      :
0   3258 |o                      :
0   3374 |o                      :
6   3236 |     o                 :
2   3276 | o                     :
2   3204 + o                     :
6   3228 |     o                 :
4   3204 |   o                   :
8   3418 |       o               :
0   3304 |o                      :
2   3312 + o                     :
0   3234 |o                      :
2   3266 | o                     :
4   3368 |   o                   :
4   3404 |   o                   :
2   3140 + o                     :
2   3164 | o                     :
2   3292 | o                     :
4   3190 |   o                   :
8   3316 |       o               :
2   3260 + o                     :
2   3304 | o                     :
4   3266 |   o                   :
0   3136 |o                      :
0   3252 |o                      :
6   3448 +     o                 :
2   3188 | o                     :
6   3328 |     o                 :
2   3352 | o                     :
2   3338 | o                     :
2   3308 + o                     :
4   3256 |   o                   :
2   3350 | o                     :
2   3374 | o                     :
6   3286 |     o                 :
8   3124 +       o               :
6   3170 |     o                 :
2   3254 | o                     :
2   3230 | o                     :
6   3194 |     o                 :
0   3292 +o                      :
4   3416 |   o                   :
2   3310 | o                     :
```

```
4  3270 |  o            :
4  3204 |  o            :
8  3274 +    o          :
4  3288 |  o            :
0  3310 |o              :
0  3420 |o              :
2  3170 | o             :
2  3392 + o             :
6  3134 |   o           :
6  3334 |   o           :
4  3302 |  o            :
2  3220 | o             :
0  3278 +o              :
2  3302 | o             :
4  3308 |  o            :
2  3280 | o             :
8  3308 |    o          :
8  3352 +    o          :
2  3306 | o             :
2  3082 | o             :
4  3262 |  o            :
6  3290 |   o           :
2  3200 + o             :
4  3150 |  o            :
0  3246 |o              :
0  3148 |o              :
0  3288 |o              :
6  3294 +   o           :
4  3282 |  o            :
8  3182 |    o          :
4  3312 |  o            :
0  3202 |o              :
2  3276 + o             :
0  3404 |o              :
2  3348 | o             :
6  3278 |   o           :
0  3136 |o              :
0  3254 +o              :
4  3238 |  o            :
0  3304 |o              :
0  3340 |o              :
2  3240 | o             :
4  3112 +  o            :
4  3274 |  o            :
```

```
2   3236 | o                    :
4   3414 |   o                  :
2   3302 | o                    :
2   3310 + o                    :
2   3084 | o                   :
2   3192 | o                    :
2   3280 | o                    :
0   3192 |o                     :
6   3286 +    o                 :
0   3222 |o                     :
8   3326 |      o               :
4   3166 |   o                  :
6   3244 |    o                 :
0   3160 +o                     :
2   3186 | o                    :
0   3262 |o                     :
2   3212 | o                    :
2   3284 | o                    :
6   3284 +    o                 :
2   3102 | o                   :
2   3076 | o                   :
4   3152 |   o                  :
0   3254 |o                     :
4   3306 +   o                  :
4   3248 |   o                  :
2   3266 | o                    :
8   3236 |      o               :
0   3248 |o                     :
8   3018 +      o              :
0   3186 |o                     :
4   3230 |   o                  :
0   3184 |o                     :
0   3222 |o                     :
6   3180 +    o                 :
8   3362 |      o               :
4   3290 |   o                  :
6   3226 |    o                 :
6   3224 |    o                 :
4   3222 +   o                  :
2   3134 | o                    :
4   3350 |   o                  :
2   3308 | o                    :
2   3308 | o                    :
0   3174 +o                     :
```

```
4   3334 |   o                    :
4   3260 |   o                    :
2   3426 | o                      :
2   3374 | o                      :
2   3388 + o                      :
4   3200 |   o                    :
6   3216 |     o                  :
4   3162 |   o                    :
0   3074 |o                       :
6   3254 +     o                  :
4   3394 |   o                    :
6   3278 |     o                  :
6   3204 |     o                  :
2   3332 | o                      :
2   3204 + o                      :
2   3098 | o                     :
8   3302 |       o                :
2   3254 | o                      :
2   3398 | o                      :
2   3260 + o                      :
2   3312 | o                      :
0   3244 |o                        :
4   3310 |   o                    :
2   3294 | o                      :
2   3306 + o                      :
6   3316 |     o                  :
2   3222 | o                      :
0   3260 |o                        :
4   3222 |   o                    :
2   3266 + o                      :
4   3360 |   o                    :
2   3324 | o                      :
2   3196 | o                      :
0   3270 |o                        :
4   3196 +   o                    :
4   3280 |   o                    :
2   6208 | o                              :
4   6176 |   o                            :
6   5942 |     o                         :
2   5996 + o                             :
0   6292 |o                                :
0   6136 |o                               :
0   6202 |o                                :
2   6256 | o                              :
```

```
4   6082 +   o                        :
0   6092 |o                           :
4   6218 |   o                        :
8   6176 |       o                    :
6   6392 |     o                      :
4   6232 +   o                        :
6   6098 |     o                      :
2   6174 | o                          :
0   6164 |o                           :
2   6186 | o                          :
0   6024 +o                           :
6   6252 |     o                      :
6   5930 |     o                      :
2   6148 | o                          :
4   6184 |   o                        :
2   6014 + o                          :
2   6058 | o                          :
2   6158 | o                          :
2   5966 | o                          :
2   5956 | o                          :
0   5994 +o                           :
0   6358 |o                           :
4   6048 |   o                        :
2   6308 | o                          :
0   6090 |o                           :
2   5978 + o                          :
0   6176 |o                           :
2   5994 | o                          :
4   6244 |   o                        :
2   6110 | o                          :
8   6182 +       o                    :
6   6294 |     o                      :
4   6240 |   o                        :
2   6138 | o                          :
4   6154 |   o                        :
6   6066 +     o                      :
8   6166 |       o                    :
0   6184 |o                           :
2   6196 | o                          :
2   6162 | o                          :
2   6108 + o                          :
2   6194 | o                          :
4   6138 |   o                        :
2   6104 | o                          :
```

```
   6  6338 |     o                       :
   4  6158 +   o                         :
   0  6044 |o                            :
   6  6178 |     o                       :
   6  6128 |     o                      :
   0  6096 |o                            :
   2  6000 + o                          :
   8  6348 |       o                     :
Centre at point 121.000      Angles =    30.3642   15.1820    0.0000  120.0000
I(int)=    14.56  Sigma=41.88  FWHM=4.2139  Imax( 23)=     5.30  Temp=   0.00K
** Int. too low
# chi 90
# dr
# ph 0
# dr
# th 40 20
# dr
# pp
     40.0000   20.0000   90.0000    0.0000
# sv
Step scan parameters
 Nstep     :  241 ? 181
 Stepwidths:   0.0000   0.0000   0.0000   0.5000 ? 0.4 0.2 0 0
 Time/step :   0.50 sec ?

Data stored in chon.pm4
# ss
Scan centre =    40.0000   20.0000   90.0000    0.0000
Scan range  =    72.0000   36.0000    0.0000    0.0000  (181 steps, stepmode)
   26   3582 |                      o :
   30   3552 |                       :   o
   32   3466 |                      :    o
   28   3410 |                      :  o
   30   3332 +                      :   o
   22   3488 |                   o    :
   24   3456 |                     o  :
   20   3642 |                 o       :
   22   3588 |                   o     :
   26   3396 +                      :o
   22   3550 |                   o     :
   18   3422 |               o        :
   12   3428 |         o              :
    6  3476 |     o                 :
   14   3316 +           o            :
```

```
12   3348 |          o            :
 6  3330 |     o                  :
 0  3320 |o                       :
 6  3344 |     o                  :
12   3356 +          o            :
 8  3274 |       o                :
 6  3452 |     o                  :
 4  3262 |   o                    :
 6  3410 |     o                  :
 6  3270 +     o                  :
 8  3508 |       o                :
 4  3338 |   o                    :
 2  3362 | o                      :
 6  3214 |     o                  :
 2  3482 + o                      :
 8  3370 |       o                :
 0  3346 |o                       :
 2  3194 | o                      :
 2  3452 | o                      :
 8  3324 +       o                :
 0  3358 |o                       :
 2  3294 | o                      :
 2  3428 | o                      :
 2  3438 | o                      :
 4  3412 +   o                    :
 2  3370 | o                      :
 4  3218 |   o                    :
 4  3300 |   o                    :
 2  3400 | o                      :
 0  3364 +o                       :
 8  3312 |       o                :
 0  3384 |o                       :
 0  3282 |o                       :
 6  3394 |     o                  :
 2  3408 + o                      :
 2  3426 | o                      :
 6  3276 |     o                  :
 4  3270 |   o                    :
 4  3198 |   o                    :
 8  3296 +       o                :
 0  3332 |o                       :
 2  3316 | o                      :
 2  3340 | o                      :
 6  3152 |     o                 :
```

4 3214 + o :
0 3406 |o :
4 3238 | o :
2 3290 | o :
4 3352 | o :
2 3464 + o :
2 3242 | o :
0 3240 |o :
8 3276 | o :
2 3366 | o :
2 3168 + o :
4 3300 | o :
0 3414 |o :
4 3212 | o :
4 3344 | o :
0 3318 +o :
4 3318 | o :
2 3472 | o :
2 3388 | o :
0 3440 |o :
0 3272 +o :
4 3362 | o :
0 3218 |o :
4 3290 | o :
0 3278 |o :
0 3130 +o :
2 3140 | o :
0 3198 |o :
0 3244 |o :
4 3148 | o :
0 3264 +o :
0 3216 |o :
4 3344 | o :
2 3200 | o :
8 3344 | o :
4 3374 + o :
4 3180 | o :
2 3374 | o :
0 3102 |o :
2 3362 | o :
2 3314 + o :
0 3132 |o :
4 3200 | o :
2 3380 | o :

0 6104 |o :
0 6102 +o :
0 6218 |o :
2 5982 | o :
2 6246 | o :
2 5940 | o :
2 5966 + o :
0 6218 |o :
6 6116 | o :
4 6108 | o :
2 6234 | o :
4 6116 + o :
0 6058 |o :
8 6356 | o :
2 5942 | o :
0 6012 |o :
0 6172 +o :
0 6158 |o :
6 6164 | o :
2 6260 | o :
0 6296 |o :
2 6140 + o :
6 6278 | o :
4 6456 | o :
2 6038 | o :
6 6110 | o :
2 6098 + o :
0 6180 |o :
0 6072 |o :
2 6278 | o :
2 6104 | o :
0 6112 +o :
4 6232 | o :
2 6296 | o :
0 6434 |o :
2 6318 | o :
0 6112 +o :
8 6168 | o :
4 6390 | o :
0 6284 |o :
2 6076 | o :
2 6104 + o :
2 6080 | o :
4 6304 | o :

```
   4   6240 |   o                    :
   0   6106 |o                       :
   2   6346 + o                      :
   0   6106 |o                       :
   0   6042 |o                       :
   0   6082 |o                       :
   4   6040 |   o                   :
   4   6174 +   o                   :
   2   6148 | o                     :
   0   5970 |o                       :
   0   6124 |o                       :
   2   5924 | o                     :
   2   6184 + o                     :
   2   5870 | o                     :
   4   5962 |   o                   :
   0   6126 |o                       :
   2   6086 | o                     :
   0   5978 +o                       :
   2   6052 | o                     :
   4   5996 |   o                   :
   2   6020 | o                     :
   2   6036 | o                     :
   4   5910 +   o                   :
   6   6068 |     o                 :
   2   5968 | o                     :
   4   6028 |   o                   :
   2   5848 | o                    :
   0   5944 +o                       :
   0   5930 |o                       :
   0   5952 |o                       :
   4   5982 |   o                   :
   0   5992 |o                       :
   0   6020 +o                       :
   0   6188 |o                       :
Centre at point  78.002      Angles =    34.8010   17.4005   90.0000    0.0000
I(int)=  -132.38  Sigma=57.36  FWHM=13.7505  Imax(  3)=    16.99  Temp=   0.00K
** Unsuitable scanwidth
# ca 1 0 0
Angles =    6.0048    3.0024    0.0000    0.0000
# dr
# sv
Step scan parameters
 Nstep     :  181 ? 241
 Stepwidths:   0.4000   0.2000   0.0000   0.0000 ? 0 0 0 0.5
```

Time/step :   0.50 sec ?

Data stored in chon.pm4
ss
Scan centre =   6.0047   3.0023   0.0000   0.0000
Scan range =   0.0000   0.0000   0.0000 120.0000 (241 steps, stepmode)

```
   22   3398 |           o  :
   16   3644 |        o      :
   24   3606 |            o  :
   14   3528 |       o      :
   22   3638 +           o   :
   20   3604 |          o    :
   34   3440 |             :   o
   28   3348 |             : o
   20   3400 |          o   :
   36   3360 +             :    o
   18   3440 |         o    :
   16   3436 |        o     :
   34   3438 |             :   o
   40   3424 |             :      o
   24   3448 +            o :
   48   3340 |             :          o
   18   3284 |         o    :
   22   3392 |           o  :
   36   3540 |             :    o
   14   3296 +       o      :
   14   3294 |       o      :
   36   3356 |             :    o
   30   3380 |             :  o
   14   3320 |       o      :
   38   3380 +             :     o
   22   3464 |           o  :
   20   3352 |          o   :
   34   3348 |             :   o
   28   3350 |             : o
   28   3442 +             : o
   22   3330 |           o  :
   30   3538 |             :  o
   28   3280 |             : o
   24   3290 |            o :
   38   3292 +             :     o
   32   3352 |             :  o
   22   3424 |           o  :
   20   3302 |          o   :
```

```
10  3284 |   o        :
26  3148 +           : o
30  3324 |            :  o
22  3314 |         o  :
24  3312 |          o :
24  3198 |          o :
22  3372 +         o  :
34  3300 |            :    o
26  3308 |            :o
26  3396 |            :o
30  3384 |            :  o
14  3238 +     o      :
16  3258 |      o     :
26  3350 |            :o
30  3284 |            :  o
24  3192 |          o :
18  3266 +       o    :
18  3288 |       o    :
24  3310 |          o :
24  3212 |          o :
28  3388 |            : o
22  3274 +         o  :
28  3274 |            : o
24  3368 |          o :
30  3318 |            :  o
20  3350 |        o   :
18  3366 +       o    :
22  3382 |         o  :
30  3176 |            :  o
22  3310 |         o  :
18  3346 |       o    :
36  3382 +            :     o
26  3286 |            :o
22  3216 |         o  :
 8  3360 |  o         :
26  3360 |            :o
28  3406 +            : o
28  3368 |            : o
22  3294 |         o  :
26  3384 |            :o
24  3294 |          o :
26  3308 +            :o
22  3392 |         o  :
24  3278 |          o :
```

```
36  3326 |             :    o
22  3474 |           o  :
24  3182 +            o :
22  3334 |           o  :
20  3410 |          o   :
14  3394 |       o      :
22  3238 |           o  :
26  3292 +              :o
24  3394 |            o :
20  3446 |          o   :
26  3518 |              :o
14  3424 |       o      :
16  3132 +        o     :
22  3196 |           o :
18  3344 |         o    :
32  3288 |             :  o
20  4048 |          o    :
18  6128 +         o        :
30  5962 |               o  :
16  6142 |        o         :
16  6244 |        o          :
14  6124 |       o          :
30  6198 +               o   :
30  6084 |               o  :
18  6248 |         o         :
34  6102 |                 o :
32  6372 |                o  :
32  6002 +                o :
26  6102 |             o    :
20  6022 |          o       :
24  6094 |            o     :
22  6166 |           o      :
18  5876 +         o        :
26  6146 |             o    :
22  5970 |           o      :
34  5984 |                 o :
26  6370 |             o     :
24  6204 +            o      :
24  6110 |            o     :
28  6346 |              o    :
32  6420 |                o  :
20  6108 |          o       :
30  6170 +               o  :
28  6004 |              o   :
```

```
22  6096 |          o       :
30  6122 |              o   :
26  6022 |            o     :
28  6128 +             o    :
20  6068 |         o        :
24  6018 |           o      :
16  6442 |       o          :
26  6050 |            o     :
28  6182 +             o    :
30  6154 |              o   :
18  6254 |        o         :
22  6294 |          o       :
26  6150 |            o     :
18  6076 +        o         :
16  6218 |       o          :
12  6312 |     o            :
16  6266 |       o          :
28  6106 |             o    :
20  6144 +         o        :
22  6170 |          o       :
28  6192 |             o    :
26  6096 |            o     :
16  6176 |       o          :
18  6170 +        o         :
34  6278 |                o :
28  6152 |             o    :
28  6280 |             o    :
22  6212 |          o       :
20  5966 +         o        :
20  6112 |         o        :
30  6348 |              o   :
42  6174 |                 :   o
28  6084 |             o    :
26  6198 +            o     :
26  6146 |            o     :
30  6092 |              o   :
20  6168 |         o        :
32  6012 |               o  :
18  6118 +        o         :
32  5954 |               o  :
22  5882 |          o       :
14  6018 |      o           :
40  5996 |                 :  o
28  5984 +             o    :
```

```
26   6012 |            o    :
22   6092 |          o      :
28   6038 |             o   :
30   6086 |              o  :
20   6062 +         o       :
18   6058 |        o        :
18   6230 |        o        :
18   6100 |        o        :
 8   6106 |  o             :
28   6246 +             o   :
24   6186 |           o     :
32   6086 |              o  :
30   6054 |              o  :
34   6014 |               o :
28   6148 +             o   :
12   6070 |     o           :
44   5958 |               :     o
30   5936 |              o  :
22   6030 |          o      :
12   6032 +     o           :
20   5928 |         o       :
20   5964 |         o       :
18   5856 |        o       :
18   5814 |        o       :
14   6022 +      o          :
20   6004 |         o       :
22   5894 |          o      :
14   6140 |      o          :
22   6030 |          o      :
20   6024 +         o       :
28   5928 |             o   :
26   5870 |            o    :
30   5916 |              o  :
20   5916 |         o       :
34   5760 +               :o
18   5928 |        o        :
24   5728 |           o    :
36   5600 |               : o
16   5826 |       o        :
10   5724 +    o           :
44   5742 |               :     o
28   5754 |             o  :
16   5652 |       o        :
24   5796 |           o    :
```

```
   18   5924 +               o               :
   30   5896 |                          o    :
   30   5914 |                          o    :
   18   5854 |               o              :
   26   5960 |                      o        :
   24   5882 +                    o          :
   38   5698 |                            :    o
   22   5702 |                   o          :
   32   5728 |                          o  :
   24   5638 |                     o        :
   32   5766 +                          o  :
   26   5642 |                      o       :
   30   5638 |                          o   :
   38   5496 |                           :     o
   26   5694 |                      o       :
   16   5602 +             o                :
   24   5456 |                     o        :
   22   5586 |                   o          :
   30   5476 |                         o  :
   20   5438 |                 o           :
   20   5522 +                 o           :
   14   5482 |           o                 :
   20   5482 |                 o           :
   28   5416 |                        o    :
   22   5362 |                   o         :
   22   5230 +                   o         :
   26   5192 |                      o     :
Centre at point 123.446      Angles =     6.0047    3.0023    0.0000    1.2231
I(int)=  -118.66  Sigma=122.30  FWHM=61.7429  Imax( 16)=    22.44  Temp=   0.00K

# ph 26.5
# dr
# sv
Step scan parameters
 Nstep     :  241 ? 31
 Stepwidths:   0.0000   0.0000   0.0000   0.5000 ? 0 0 0.2 0
 Time/step :   0.50 sec ?

Data stored in chon.pm4
# ss
Scan centre =     6.0045    3.0023    0.0000   26.5000
Scan range  =     0.0000    0.0000    6.0000    0.0000  ( 31 steps, stepmode)
   20   3500 |                 o            :
   22   3562 |                   o            :
```

```
   32   3446 |                         o :
   20   3368 |                o        :
   20   3358 +                o        :
   22   3462 |                 o        :
   34   3450 |                          : o
   24   3376 |                   o     :
   32   3330 |                      :   o
   30   3416 +                       o :
   30   3318 |                      :  o
   30   3410 |                       o :
   10   3364 |       o                 :
   18   3402 |              o          :
   28   3444 +                     o    :
   28   3234 |                   :   o
   28   3398 |                     o  :
   38   3316 |                      :       o
   16   3416 |            o            :
   38   3282 +                     :        o
   34   3312 |                      :    o
   32   3362 |                       :  o
   22   3464 |                 o        :
   26   3352 |                   o   :
    8   3436 +      o                  :
   26   3414 |                   o    :
   28   3440 |                     o    :
   18   3420 |              o           :
   30   3370 |                       : o
   26   3392 +                   o    :
   22   3404 |                 o       :
Centre at point  16.000      Angles =     6.0045    3.0023    0.0000   26.5000
I(int)=    10.84  Sigma=42.51  FWHM=1.9069  Imax( 18)=    14.05  Temp=   0.00K
** Int. too low
# pp
      6.0045    3.0023   -0.0000  124.0000
# ca 0 0 1
Angles =     3.5282    1.7641   73.7600    0.0000
# ca 0 0 2
Angles =     7.0598    3.5299   73.7600    0.0000
# ca 0 0 3
Angles =    10.5981    5.2990   73.7600    0.0000
# ca 0 0 4
Angles =    14.1465    7.0733   73.7600    0.0000
# ca 1 0 0
Angles =     6.0048    3.0024    0.0000    0.0000
```

```
# ch 5 2
# dr
# co 1 1
   31   3427
# sv
Step scan parameters
 Nstep     :   31 ? 21
 Stepwidths:   0.0000   0.0000   0.2000   0.0000 ? 0.1 0 0 0
 Time/step :   0.50 sec ?

Data stored in chon.pm4
# ss
Scan centre =     6.0047    3.0023    5.0000    2.0000
Scan range  =     2.0000    0.0000    0.0000    0.0000  ( 21 steps, stepmode)
   52   3284 |                      :                  o
   40   3302 |                      :         o
   52   3256 |                     :                   o
   46   3312 |                      :             o
   52   3300 +                      :                  o
   52   3222 |                    :                    o
   46   3338 |                       :            o
   60   3474 |                           :                   o
   36   3434 |                          : o
   44   3370 +                        :          o
   48   3286 |                      :               o
   16   3450 |            o               :
   16   3348 |            o           :
   12   3348 |         o              :
   22   3244 +                 o    :
   28   3346 |                      o  :
   20   3410 |               o           :
   36   3376 |                        :    o
   22   3468 |                 o           :
   22   3470 +                 o           :
   28   3436 |                      o     :
Centre at point  21.000      Angles =     7.0047    3.0023    5.0000    2.0000
I(int)=    -0.60  Sigma=40.23  FWHM=1.0375  Imax(  8)=    20.00  Temp=   0.00K
** Unsuitable scanwidth
# th  6.0047    3.0023    5.0000    2.0000
# sv
Step scan parameters
 Nstep     :   21 ?

 Stepwidths:   0.1000   0.0000   0.0000   0.0000 ? 0 0.1 0 0
```

```
 Time/step :   0.50 sec ?

Data stored in chon.pm4
# ss
Scan centre =     6.0047    3.0023    5.0000    2.0000
Scan range  =     0.0000    2.0000    0.0000    0.0000  ( 21 steps, stepmode)
   28   6296 |                     o        :
   40   6156 |                          :     o
   38   5842 |                    :          o
   56   5972 |                       :                      o
   44   5964 +                       :           o
   44   6202 |                           :       o
   52   6158 |                          :              o
   52   6278 |                            :            o
   48   6052 |                         :             o
   26   6302 +                   o          :
   28   5972 |                     o  :
   44   5982 |                       :           o
   40   6190 |                           :    o
   46   5992 |                        :            o
   40   6214 +                           :    o
   64   6154 |                          :                        o
   54   6154 |                          :                o
   46   6100 |                         :           o
   28   6004 |                     o   :
   14   6080 +          o               :
   22   6074 |                 o        :
Centre at point  11.000      Angles =     6.0047    3.0023    5.0000    2.0000
I(int)=    25.90  Sigma=36.43  FWHM=0.7131  Imax( 16)=    39.56  Temp=   0.00K
** Int. too low
# om 3.5
# sv
Step scan parameters
 Nstep     :   21 ?

 Stepwidths:   0.0000   0.1000   0.0000   0.0000 ? 0 0 0.2 0
 Time/step :   0.50 sec ?

Data stored in chon.pm4
# ss
Scan centre =     6.0047    3.5000    5.0000    2.0000
Scan range  =     0.0000    0.0000    4.0000    0.0000  ( 21 steps, stepmode)
   38   6074 |                      o      :
   34   6100 |                    o        :
```

```
   60   6018 |                          :          o
   54   5922 |                        :        o
   52   5968 +                         :     o
   46   6150 |                           o :
   52   5886 |                        :      o
   58   5974 |                         :        o
   68   5900 |                        :                o
   50   5930 +                         :    o
   62   5804 |                      :              o
   68   6144 |                           :             o
   78   5948 |                         :                     o
   70   5880 |                        :                 o
   64   5810 +                      :               o
   82   5874 |                        :                        o
   62   5562 |                   :                 o
   48   5714 |                     :       o
   48   5770 |                      :     o
   60   5850 +                       :            o
   54   5782 |                      :          o
Centre at point  11.000      Angles =     6.0047    3.5000    5.0000    2.0000
I(int)=    35.80  Sigma=47.71  FWHM=1.2347  Imax( 16)=    30.22  Temp=   0.00K
** Int. too low
# ch 6
# sv
Step scan parameters
 Nstep     :   21 ?

 Stepwidths:   0.0000   0.0000   0.2000   0.0000 ? 0 0 0 0.1
 Time/step :   0.50 sec ?

Data stored in chon.pm4
# ss
Scan centre =     6.0047    3.5000    6.0000    2.0000
Scan range  =     0.0000    0.0000    0.0000    2.0000  ( 21 steps, stepmode)
   64   4124 |                           :     o
   36   4084 |                  o           :
   30   3968 |              o             :
   22   4030 |          o                  :
   32   4122 +              o              :
   40   3720 |                   o   :
   42   3916 |                    o      :
   44   3920 |                      o    :
   20   3912 |         o                 :
   54   3764 +                      :     o
```

```
   96   3742 |                       :                         o
   50   3600 |                     :    o
   36   3728 |                  o    :
   18   3672 |        o              :
   26   3858 +            o             :
   34   3778 |                o       :
   36   3742 |                  o     :
   20   3618 |          o           :
   30   3798 |               o         :
   16   3546 +       o             :
   18   3690 |        o              :
Centre at point  12.966     Angles =    6.0047    3.5000    6.0000    2.1966
I(int)=    8.50  Sigma=38.37  FWHM=0.2703  Imax( 11)=    63.67  Temp=   0.00K
** Unsuitable scanwidth
# co 1 1
   18   3247
# pp
     6.0045    3.5000    6.0000    3.0000
# ph 2.2
# dr
# co 1 1
   41   3291
# ph 2
# dr
# co 1 1
   59   3366
# sv
Step scan parameters
 Nstep    :   21 ?

 Stepwidths:   0.0000   0.0000   0.0000   0.1000 ? 0 0 0 0.04
 Time/step :   0.50 sec ?

Data stored in chon.pm4
# ss
Scan centre =    6.0045    3.5000    6.0000    2.0000
Scan range  =    0.0000    0.0000    0.0000    0.8000  ( 21 steps, stepmode)
   38   3408 |                          :o
   32   3354 |                     o   :
   38   3296 |                      :    o
   48   3280 |                      :           o
   48   3324 +                      :          o
   40   3456 |                          :o
   52   3298 |                      :              o
```

```
   36   3284 |                      :  o
   56   3206 |                    :                   o
   66   3328 +                      :                       o
   60   3246 |                     :                    o
   72   3266 |                     :                            o
   56   3336 |                      :               o
   54   3346 |                       :             o
   30   3272 +                    o  :
   42   3326 |                      :     o
   40   3418 |                         : o
   32   3312 |                     o  :
   22   3220 |              o       :
   20   3336 +              o           :
   42   3526 |                            :o
Centre at point  11.000      Angles =     6.0045    3.5000    6.0000    2.0000
I(int)=    10.08  Sigma=38.21  FWHM=0.2109  Imax( 12)=    40.44  Temp=   0.00K
** Int. too low
# sv
Step scan parameters
 Nstep     :   21 ?

 Stepwidths:   0.0000   0.0000   0.0000   0.0400 ? 0.05 0 0 0
 Time/step :   0.50 sec ?

Data stored in chon.pm4
# ss
Scan centre =     6.0045    3.5000    6.0000    2.0000
Scan range  =     1.0000    0.0000    0.0000    0.0000  ( 21 steps, stepmode)
   90   3292 |                       :               o
  110   3232 |                      :                          o
  100   3382 |                          :                  o
   66   3250 |                      :      o
   70   3428 +                          :   o
   86   3168 |                    :                 o
   78   3280 |                      :           o
   72   3212 |                     :         o
   78   3294 |                       :          o
   60   3132 +                   :       o
   64   3236 |                      :     o
   56   3420 |                       o    :
   50   3176 |                     :o
   30   3386 |            o               :
   30   3234 +            o           :
   22   3312 |         o                :
```

```
  28  3302 |       o        :
  32  3288 |        o       :
  22  3232 |      o        :
  24  3228 +      o        :
  18  3214 |     o         :
Centre at point   6.520      Angles =     5.7805    3.5000    6.0000    2.0000
I(int)=    36.90  Sigma=52.95  FWHM=0.1582  Imax(  2)=    88.67  Temp=   0.00K
** Unsuitable scanwidth
# ss
Scan centre =     5.7805    3.5000    6.0000    2.0000
Scan range  =     1.0000    0.0000    0.0000    0.0000  ( 21 steps, stepmode)
  60  3330 |            :      o
  62  3292 |            :      o
  82  3338 |            :            o
  86  3194 |            :              o
  78  3274 +            :           o
  72  3316 |            :         o
  82  3248 |            :            o
  98  3328 |            :                 o
  66  3256 |            :       o
  90  3124 +            :               o
  72  3416 |            :         o
  84  3356 |            :             o
  60  3204 |            :      o
  92  3210 |            :               o
  78  3298 +            :           o
  54  3188 |            :    o
  50  6062 |               o :
  44  6358 |             o    :
  24  6112 |       o         :
  36  6132 +           o      :
  18  6250 |     o            :
Centre at point  11.000      Angles =     5.7805    3.5000    6.0000    2.0000
I(int)=    20.05  Sigma=46.42  FWHM=0.1586  Imax( 14)=    52.00  Temp=   0.00K
** Int. too low
# th 5.7805    3.5000    6.0000    2.0000
# sv
Step scan parameters
 Nstep     :   21 ?

 Stepwidths:   0.0500   0.0000   0.0000   0.0000 ? 0 0.05 0 0
 Time/step :   0.50 sec ?

Data stored in chon.pm4
```

```
# ss
Scan centre =    5.7805    3.5000    6.0000    2.0000
Scan range  =    0.0000    1.0000    0.0000    0.0000  ( 21 steps, stepmode)
   46   6152 |                     :o
   56   6056 |                   :        o
   34   6050 |                o    :
   64   6130 |                     :          o
   48   6364 +                       o   :
   44   6250 |                     o   :
   66   6162 |                      :          o
   74   6166 |                      :              o
   70   5986 |                  :               o
   62   6256 +                       :      o
   90   6312 |                        :                   o
   84   6072 |                    :                    o
   86   6176 |                      :                   o
   92   6292 |                        :                   o
   66   6054 +                    :           o
   56   6110 |                     :      o
   34   6188 |                o       :
   40   5980 |                  : o
   18   6264 |         o                :
   22   6006 +           o         :
   36   6002 |                 o  :
Centre at point  11.000     Angles =    5.7805    3.5000    6.0000    2.0000
I(int)=    22.30  Sigma=40.43  FWHM=0.2711  Imax( 14)=    60.00  Temp=   0.00K
** Int. too low
# ca 2 0 0
Angles =    12.0262    6.0131    0.0000    0.0000
# ch 6 2
# dr
# co 1 1
    8   5283
# sv
Step scan parameters
 Nstep     :   21 ? 41
 Stepwidths:   0.0000   0.0500   0.0000   0.0000 ?

 Time/step :   0.50 sec ?

Data stored in chon.pm4
# ss
Scan centre =    12.0260    6.0130    6.0000    2.0000
Scan range  =    0.0000    2.0000    0.0000    0.0000  ( 41 steps, stepmode)
```

```
   8   4954 |    o            :
   8   5028 |    o            :
   6   4902 |   o            :
   0   4888 |o                :
   0   4804 +o                :
  14   4826 |       o         :
   8   4882 |    o           :
   6   4774 |   o            :
  12   5012 |      o          :
  12   4732 +      o          :
  10   4750 |     o           :
   6   4784 |   o            :
   8   4616 |    o          :
  10   4708 |     o          :
  10   4528 +     o          :
   6   4758 |   o            :
   8   4546 |    o          :
  10   4506 |     o          :
  10   4434 |     o          :
   4   4154 +  o            :
   2   4174 | o            :
   8   4298 |    o          :
   2   4338 | o             :
  12   4198 |      o        :
  14   4262 +       o       :
   4   4124 |  o           :
  14   4134 |       o      :
  10   4152 |     o         :
   8   4090 |    o         :
   6   4102 +   o          :
  12   4058 |      o       :
   4   3952 |  o          :
   2   3888 | o           :
   2   4086 | o            :
  12   3880 +      o       :
   8   3808 |    o        :
   4   3712 |  o         :
   8   3762 |    o        :
   4   3858 |  o          :
  12   3882 +      o       :
   8   3758 |    o        :
```
Centre at point 21.000   Angles =   12.0260   6.0130   6.0000   2.0000

I(int)=   1.93 Sigma=24.57 FWHM=0.8315 Imax(6)=   7.90 Temp=   0.00K

** Int. too low

```
# ca 0 1 0
Angles =    4.4866    2.2433    0.0000  -90.0000
# ca 0 2 0
Angles =    8.9800    4.4900    0.0000  -90.0000
# ca 2 0 0
Angles =   12.0262    6.0131    0.0000    0.0000
# ca 0 0 2
Angles =    7.0598    3.5299   73.7600    0.0000
# th 10 0 0 0
# dr
# ca 0 0 2
Angles =    7.0598    3.5299   73.7600    0.0000
# ch 33 290
# dr
# pp
      7.0598    3.5297   33.0000  -70.0000
# co 1 1
   141  13312
# sv
Step scan parameters
 Nstep    :   41 ?

 Stepwidths:   0.0000   0.0500   0.0000   0.0000 ? 0 0 0 0.1
 Time/step :   0.50 sec ?

Data stored in chon.pm4
# ss
Scan centre =    7.0598    3.5297   33.0000  -70.0000
Scan range  =    0.0000    0.0000    0.0000    4.0000  ( 41 steps, stepmode)
    50   8860 |         o          :
    66   8900 |            o        :
    46   8624 |        o         :
    34   8842 |      o             :
    60   8780 +          o        :
    56   8848 |          o         :
    42   8602 |       o         :
    44   8480 |        o       :
    54   8816 |         o         :
    62   8830 +          o        :
    52   8832 |         o          :
    60   8848 |          o         :
    60   8444 |          o    :
    62   8530 |          o     :
    66   8718 +           o      :
```

```
   42   8876 |           o                  :
   52   8524 |             o          :
   56   8800 |               o             :
   94   8720 |                         o  :
   90   8756 +                        o    :
  158   8596 |                         :                  o
  192   8548 |                        :                            o
  170   8662 |                          :                    o
  126   8624 |                          :         o
  116   8838 +                              :  o
   96   8818 |                          o   :
   92   8660 |                         o :
  102   8630 |                          :   o
  106   8828 |                             :o
  118   8754 +                            :     o
  104   8626 |                          :    o
  102   8620 |                          :   o
  116   8802 |                             :   o
   78   8778 |                    o        :
   66   8830 +                  o           :
   50   8918 |             o                  :
   48   8582 |            o            :
   68   8856 |                  o            :
   56   8680 |               o           :
   54   8658 +              o            :
   58   8866 |               o              :
Centre at point  25.528      Angles =     7.0598    3.5297   33.0000  -69.5472
I(int)=   101.68  Sigma=71.05  FWHM=0.5046  Imax( 22)=   138.06  Temp=   0.00K
** Unsuitable scanwidth
# ph -69.5472
# sv
Step scan parameters
 Nstep     :   41 ?

 Stepwidths:   0.0000   0.0000   0.0000   0.1000 ? 0 0 0.2 0
 Time/step :   0.50 sec ?

Data stored in chon.pm4
# ss
Scan centre =     7.0598    3.5297   33.0000  -69.5450
Scan range  =     0.0000    0.0000    8.0000    0.0000  ( 41 steps, stepmode)
   62  15350 |                    o        :
   82  15312 |                             o :
   52  15022 |                   o          :
```

```
  72  15088 |                  o   :
  60  14918 +                o     :
  98  15254 |                      :    o
  72  15162 |                  o   :
  70  15088 |                 o    :
  90  15192 |                     :   o
  52  15304 +             o        :
 104  15060 |                      :      o
  88  15208 |                     :  o
 124  15248 |                      :           o
  76  15068 |                   o :
 126  14974 +                     :           o
  98  15176 |                     :    o
  88  15002 |                     :  o
 118  15032 |                     :         o
  82  15048 |                     : o
 142  14950 +                     :                o
  94  14876 |                    :    o
 102  14750 |                     :      o
 120  14852 |                     :          o
  96  15030 |                     :    o
 100  14878 +                     :      o
  98  14674 |                   :      o
 144  14474 |                   :                o
 102  14452 |                   :        o
 124  14644 |                   :           o
  96  14636 +                   :      o
 102  14432 |                   :        o
 102  14698 |                   :        o
 128  14502 |                   :            o
  92  14116 |                 :      o
  74  14680 +                   :o
  80  14356 |                  :  o
 114  14258 |                  :          o
  66  14060 |                 :o
  88  13926 |                :      o
  84  13756 +                :     o
 102  13650 |                :          o
Centre at point  21.000      Angles =     7.0598    3.5297   33.0000  -69.5450
I(int)=   116.80  Sigma=86.22  FWHM=0.7680  Imax( 20)=    62.51  Temp=   0.00K
** Int. too low
# sv
Step scan parameters
 Nstep    :   41 ?
```

```
 Stepwidths:   0.0000   0.0000   0.2000   0.0000 ? 0 0.05 0 0
 Time/step :   0.50 sec ?

Data stored in chon.pm4
# ss
Scan centre =     7.0598    3.5297   33.0000  -69.5450
Scan range  =     0.0000    2.0000    0.0000    0.0000  ( 41 steps, stepmode)
   86  15356 |                      o     :
   60  15282 |                o           :
   56  15188 |               o           :
   60  15102 |                o        :
   76  15054 +                    o    :
   64  15430 |                o             :
   48  15298 |            o               :
   64  15126 |                o         :
   78  15256 |                     o      :
   96  15198 +                         o :
  102  15470 |                          o   :
   90  15122 |                       o  :
  146  15032 |                       :               o
  190  15186 |                          :                        o
  146  15116 +                         :              o
  132  15088 |                        :           o
  180  14832 |                     :                          o
  128  14866 |                      :            o
  104  15202 |                          :  o
  108  15128 +                         :    o
   90  15202 |                       o   :
   76  15128 |                    o     :
   90  15154 |                       o  :
  104  15330 |                            :o
   92  15028 +                       : o
   98  15142 |                        : o
  122  15140 |                         :        o
  112  15186 |                          :    o
  138  15082 |                        :             o
  104  14930 +                       :     o
  124  15210 |                          :       o
  114  14934 |                       :        o
  126  14936 |                       :           o
  106  15352 |                            :o
   94  15072 +                        : o
   80  15062 |                     o    :
```

```
   76  15124 |                    o      :
   70  15208 |                  o        :
   56  15004 |              o          :
   58  14922 +              o         :
   62  14896 |                o      :
Centre at point  21.631      Angles =     7.0598    3.5612   33.0000  -69.5450
I(int)=    62.95  Sigma=78.46  FWHM=0.8296  Imax( 14)=   123.00  Temp=   0.00K

# om 3.3
# sv
Step scan parameters
 Nstep     :   41 ?

 Stepwidths:   0.0000   0.0500   0.0000   0.0000 ? 0.1 0 0 0
 Time/step :   0.50 sec ?

Data stored in chon.pm4
# ss
Scan centre =     7.0598    3.3000   33.0000  -69.5450
Scan range  =     4.0000    0.0000    0.0000    0.0000  ( 41 steps, stepmode)
   94   8822 |                          o :
   84   8820 |                        o    :
   62   8784 |                 o           :
   68   8970 |                   o            :
   70   8790 +                    o        :
   94   8954 |                          o    :
   62   8860 |                 o            :
   86   8550 |                       : o
   86   8790 |                        o    :
   84   8802 +                        o    :
   74   8746 |                     o       :
   84   8838 |                        o     :
   60   8514 |                 o      :
   70   8712 |                    o       :
   72   8772 +                     o       :
   60   8714 |                 o          :
   92   8692 |                         :o
   88   8782 |                         o   :
  104   8868 |                           : o
  120   8834 +                           :       o
  138   8858 |                           :           o
  148   8882 |                            :             o
  160   8668 |                       :                     o
  166   8740 |                         :                     o
```

```
  178   8662 +                       :                         o
  182   8922 |                           :                     o
  140   8814 |                         :           o
  140   8458 |                   :                 o
  112   8632 |                       :       o
   92   8782 +                       o  :
   62   8876 |               o           :
   62   8538 |               o      :
   50   8966 |            o                :
   60   8800 |               o          :
   56   8760 +             o           :
   46   8734 |          o             :
   36   8726 |        o               :
   40   8764 |         o              :
   36   8746 |        o               :
   34   8798 +       o                 :
   42   8760 |          o             :
Centre at point  24.220      Angles =     7.3818    3.3000   33.0000  -69.5450
I(int)=   118.18  Sigma=74.38  FWHM=0.8690  Imax( 26)=   128.83  Temp=   0.00K

# sv
Step scan parameters
 Nstep     :   41 ?

 Stepwidths:   0.1000   0.0000   0.0000   0.0000 ? 0 0.05 0 0
 Time/step :   0.50 sec ?

Data stored in chon.pm4
# ss
Scan centre =     7.3818    3.3000   33.0000  -69.5450
Scan range  =    0.0000    2.0000    0.0000    0.0000  ( 41 steps, stepmode)
   54  14624 |             o              :
   50  14404 |            o               :
   54  14388 |             o              :
   54  14366 |             o              :
   54  14282 +             o              :
   52  14192 |             o             :
   50  14108 |            o              :
   56  13972 |              o            :
   54  14188 |             o             :
   46  13956 +           o               :
   48  14082 |           o               :
   62  13936 |                o          :
   58  13986 |               o           :
```

```
   58  13600 |                o           :
   94  13644 +                         o   :
   70  13432 |                  o         :
  118  13312 |                          :    o
  130  13552 |                          :      o
  158  13336 |                          :             o
  184  13226 +                          :                   o
  188  12690 |                         :                     o
  180  12766 |                         :                   o
  136  12714 |                         :         o
  148  12726 |                         :            o
  110  12366 +                        :   o
  138  12298 |                        :          o
   98  12124 |                       :o
   98  12304 |                       :o
   90  11808 |                      o :
   96  11836 +                      : o
  102  11584 |                       :   o
  108  11232 |                      :    o
  120  10714 |                     :        o
  114  10606 |                     :       o
  110  10548 +                     :      o
  110  10282 |                    :       o
  108  10214 |                    :      o
   84   9822 |                   : o
   70   9832 |                 o    :
   80   9506 +                   :o
   74   9322 |                  o :
Centre at point  23.652      Angles =     7.3818    3.4326   33.0000  -69.5450
I(int)=    49.12  Sigma=80.18  FWHM=0.3376  Imax( 21)=   117.67  Temp=   0.00K

# sv
Step scan parameters
 Nstep     :   41 ?

 Stepwidths:   0.0000   0.0500   0.0000   0.0000 ? 0 0 0 0.1
 Time/step :   0.50 sec ?

Data stored in chon.pm4
# ss
Scan centre =     7.3818    3.4326   33.0000  -69.5450
Scan range  =     0.0000    0.0000    0.0000    4.0000  ( 41 steps, stepmode)
   66   8942 |                 o   :
   60   8696 |               o     :
```

```
  54   8580 |                o        :
  42   8776 |             o            :
  46   8694 +               o          :
  48   8692 |               o          :
  40   8792 |             o            :
  54   9028 |                 o        :
  50   8540 |                o         :
  54   8676 +                 o       :
  62   8762 |                   o      :
  72   8862 |                       o  :
  38   8772 |            o             :
  74   8658 |                       :o
 100   8632 +                        :         o
 160   8670 |                        :                            o
 168   8632 |                        :                              o
 176   8660 |                        :                                 o
 158   8800 |                         :                           o
 144   8864 +                         :                       o
 112   8624 |                        :            o
  82   8730 |                       :   o
  56   8896 |                  o       :
  96   8838 |                       :        o
  98   8664 +                       :        o
 112   8988 |                         :           o
  90   8776 |                       :      o
  92   8608 |                       :      o
  80   8658 |                       :  o
  48   8600 +               o         :
  66  15576 |                      o            :
  48  15556 |                o                  :
  40  15546 |              o                    :
  46  15524 |                o                  :
  44  15398 +               o                   :
  42  15056 |              o                   :
  44  15646 |               o                   :
  66  15284 |                      o            :
  70  15518 |                       o           :
  32  15608 +           o                       :
  66  14964 |                      o           :
```
Centre at point 19.807   Angles =   7.3818   3.4326   33.0000 -69.6643
I(int)=   92.30 Sigma=70.39 FWHM=0.5681 Imax(18)=   123.06 Temp=   0.00K

sv
Step scan parameters

```
 Nstep    :  41 ?

 Stepwidths:   0.0000   0.0000   0.0000   0.1000 ? 0 0 0.3 0
 Time/step :   0.50 sec ?

Data stored in chon.pm4
# ss
Scan centre =     7.3818    3.4326   33.0000  -69.6650
Scan range  =     0.0000    0.0000   12.0000    0.0000  ( 41 steps, stepmode)
   62  14834 |                o            :
   94  15024 |                        o    :
   64  14908 |                o            :
   60  14816 |                o            :
   62  14598 +                o           :
   96  14752 |                         o   :
   72  14394 |                   o        :
   84  14502 |                      o     :
   76  14646 |                   o        :
  118  14706 +                            :    o
   94  14596 |                        o   :
  102  14418 |                           :o
   94  14372 |                        o   :
  118  14496 |                           :     o
  130  14648 +                           :        o
  114  14196 |                           :    o
  136  14212 |                           :          o
  118  14202 |                           :     o
  152  14370 |                           :              o
  182  14006 +                           :                      o
  142  13944 |                           :           o
  160  13804 |                           :                o
  178  13868 |                           :                     o
  158  13608 |                          :                 o
  162  13622 +                          :                  o
  178  13230 |                          :                       o
  132  13132 |                         :           o
  144  12890 |                         :             o
  120  12790 |                         :        o
  144  12590 +                        :               o
  140  12540 |                        :               o
  130  12292 |                        :            o
  124  12290 |                        :          o
  128  11778 |                       :            o
   76  11578 +                    o   :
```

```
  108  11346 |                         :        o
  100  11402 |                         :     o
   86  10886 |                       : o
   84  10640 |                      :  o
   70  10406 +                   o   :
   64  10156 |                 o    :
Centre at point  22.632      Angles =     7.3818    3.4326   33.4896  -69.6650
I(int)=   423.05  Sigma=89.31  FWHM=2.5900  Imax( 20)=   103.19  Temp=   0.00K

# xi 1
Mode (1=HKL, 2=Angles, 3=Film-Coord, 4=HKL+Angles) [1] ? 4
Enter HKL and Angles - Terminate with a blank line
Seq#  12 ? 0 0 2  7.3818    3.4326   33.4896  -69.6650
Seq#  13 ?

  1 entries added to list 1   New n =   1
232 free entries left
# ca 0 0 3
Angles =    10.5981    5.2990   73.7600    0.0000
# ch 33.4896  -69.6650
# dr
# co 1 1
   22  15113
# pp
     10.5980    5.2990   33.4900  -69.6650
# sv
Step scan parameters
 Nstep     :   41 ?

 Stepwidths:   0.0000   0.0000   0.3000   0.0000 ? 0 0.05 0 0
 Time/step :   0.50 sec ?

Data stored in chon.pm4
# ss
Scan centre =    10.5980    5.2990   33.4900  -69.6650
Scan range  =     0.0000    2.0000    0.0000    0.0000  ( 41 steps, stepmode)
   20  14244 |                 o             :
   28  14262 |                       o       :
   18  14046 |               o               :
   30  13974 |                        o     :
   26  13724 +                     o        :
   16  13742 |             o               :
   36  13884 |                              :o
   40  13788 |                              :    o
```

```
   24  13632 |                    o         :
   20  13218 +                o            :
   16  13064 |            o               :
   22  13118 |                 o          :
   34  12944 |                             :o
   32  13006 |                           o  :
   30  12878 +                          o   :
   22  12760 |                 o         :
   26  12716 |                     o     :
   26  12270 |                     o    :
   22  12086 |                 o        :
   26  12084 +                     o    :
   20  11942 |                o          :
   16  11844 |            o             :
   30  11528 |                          :o
   20  11126 |                o        :
   36  11174 +                         :     o
   24  10766 |                   o    :
   26  10466 |                     o  :
   32  10336 |                       :   o
   26  10306 |                     o  :
   34  10222 +                       :     o
   42   9824 |                      :            o
   18   9686 |              o        :
   16   9426 |            o         :
   30   9506 |                    :    o
   24   9186 +                   o  :
   18   9252 |              o       :
   32   8982 |                   :     o
   34   9234 |                    :      o
   16   9004 |            o        :
   34   9134 +                    :      o
   36   8992 |                   :         o
```

Centre at point 21.000   Angles =   10.5980   5.2990   33.4900 -69.6650

I(int)=   1.28 Sigma=48.09 FWHM=0.6123 Imax(8)=   16.31 Temp=   0.00K

** Int. too low

ca 0 0 4

Angles =   14.1465   7.0733   73.7600   0.0000

ch 33.4900 -69.6650

dr

ss

Scan centre =   14.1465   7.0732   33.4900 -69.6650

Scan range =   0.0000   2.0000   0.0000   0.0000 (41 steps, stepmode)

```
   22  14822 |                 o        :
```

```
 18  14908 |        o       :
 14  14848 |      o         :
 18  14868 |        o       :
 10  14862 +    o           :
  8  14762 |   o           :
 18  14826 |        o      :
 14  14536 |      o       :
 16  14826 |       o       :
 34  14782 +              : o
 14  14634 |      o        :
 16  14534 |       o      :
 36  14746 |              :  o
 14  14750 |      o        :
 14  14702 +      o        :
 20  14514 |         o    :
 12  14702 |     o         :
 10  14680 |    o          :
 18  14454 |        o     :
 18  14612 +        o      :
 18  14462 |        o     :
 16  14446 |       o      :
 14  14484 |      o       :
  8  14464 |   o          :
 22  14520 +          o   :
 24  14056 |           o :
 12  14120 |     o       :
 20  14316 |         o    :
 14  14240 |      o       :
 20  13942 +         o   :
 20  13896 |         o  :
 24  14080 |           o :
 22  15220 |          o      :
  8  15010 |   o            :
 12  15144 +     o           :
 14  15108 |      o          :
 14  15246 |      o          :
 20  15260 |         o       :
 14  15004 |      o          :
 12  15104 +     o           :
 16  15416 |       o          :
Centre at point  21.000      Angles =    14.1465    7.0732   33.4900  -69.6650
I(int)=     3.65  Sigma=36.84  FWHM=0.4263  Imax( 13)=    21.00  Temp=   0.00K
** Unsuitable scanwidth
# sv
```

Step scan parameters
 Nstep   : 41 ?

 Stepwidths: 0.0000 0.0500 0.0000 0.0000 ? 0 0.1 0 0
 Time/step : 0.50 sec ?

Data stored in chon.pm4
ss
Scan centre =   14.1465   7.0732   33.4900 -69.6650
Scan range =   0.0000   4.0000   0.0000   0.0000 (41 steps, stepmode)

```
   20  15114 |                  o              :
   24  14864 |                      o          :
    2  15110 | o                               :
   12  15212 |          o                       :
   16  14834 +              o                 :
   26  15302 |                        o         :
   14  15034 |            o                    :
   20  15178 |                  o               :
   22  15060 |                    o            :
   10  15106 +        o                        :
   14  14810 |            o                   :
   10  14648 |        o                       :
   22  15066 |                    o            :
   18  14926 |                o               :
    6  14762 +    o                           :
   22  14732 |                    o           :
   38  14574 |                             :    o
   22  14744 |                    o           :
   16  14642 |              o                :
   14  14562 +            o                  :
   22  14644 |                    o          :
   14  14954 |            o                    :
   26  14676 |                        o      :
   16  14854 |              o                 :
   18  14504 +                o             :
   20  14758 |                  o             :
   14  14546 |            o                  :
   14  14464 |            o                 :
   18  14410 |                o             :
   18  14584 +                o              :
    4  14390 |   o                          :
   26  14272 |                        o    :
   24  14404 |                      o      :
   20  14250 |                  o          :
```

```
   14  14698 +            o                 :
    8  14110 |       o                    :
   20  14012 |                 o        :
    4  13752 |   o                      :
   22  13818 |                   o      :
    8  13816 +       o                  :
   14  13222 |            o          :
Centre at point  21.000      Angles =    14.1465    7.0732   33.4900  -69.6650
I(int)=    9.07  Sigma=36.58  FWHM=0.6722  Imax( 17)=    22.88  Temp=   0.00K
** Int. too low
# sv
Step scan parameters
 Nstep    :   41 ?

 Stepwidths:   0.0000   0.1000   0.0000   0.0000 ? 0.1 0 0 0
 Time/step :   0.50 sec ?

Data stored in chon.pm4
# ss
Scan centre =    14.1465    7.0732   33.4900  -69.6650
Scan range  =     4.0000    0.0000    0.0000    0.0000  ( 41 steps, stepmode)
   34  10328 |                           o  :
   26   9900 |                      o       :
   20  10236 |                 o              :
   16  10024 |             o                 :
   32   9502 +                          :o
   24   9736 |                    o        :
   10   9334 |        o                  :
   20   9536 |                 o          :
   18   9172 |              o          :
   18   9018 +              o          :
   16   9022 |            o            :
   18   8902 |              o         :
    4   8924 |   o                     :
   12   9092 |          o               :
   16   8904 +            o          :
   22   9044 |                   o      :
   22   8730 |                   o     :
   18   9056 |              o          :
   20   8514 |                 o     :
   12   8708 +          o             :
   10   8706 |        o               :
   22   8888 |                   o     :
   12   8892 |          o             :
```

```
   14   8794 |           o           :
   18   8690 +             o         :
   20   8612 |              o        :
   10   8742 |        o              :
   20   8492 |              o       :
   18   8604 |             o         :
   12   8494 +          o           :
   16   8876 |            o          :
   18   8930 |             o          :
   20   8580 |              o        :
   20   8826 |              o         :
   10   8648 +        o              :
   16   8760 |            o          :
    4  8620 |   o                   :
   16   8658 |            o         :
   22   8576 |               o      :
   10   8698 +        o              :
   18   8702 |             o         :
Centre at point  14.533     Angles =    13.4998    7.0732   33.4900  -69.6650
I(int)=   -10.92  Sigma=41.99  FWHM=2.9228  Imax(  1)=     7.88  Temp=   0.00K
```

x?

List 1   mode = 4   n =   1 (0)

Seq#	Stat	H	K	L	2Theta	Omega	Chi	Phi	Int.
12	0	0.00	0.00	2.00	7.382	3.433	33.490	-69.665	0

List 2   mode = 1   n = 11 (0)

Seq#	Stat	H	K	L	2Theta	Omega	Chi	Phi	Int.
1	0	3.00	1.00	1.00	19.907	9.953	9.845	-13.290	0
2	0	4.00	0.00	0.00	24.187	12.094	0.000	0.000	0
3	0	0.00	4.00	0.00	18.016	9.008	0.000	-90.000	0
4	0	0.00	0.00	4.00	14.147	7.073	73.760	0.000	0
5	0	5.00	3.00	1.00	34.479	17.239	5.723	-23.465	0
6	0	5.00	2.00	0.00	31.726	15.863	0.000	-16.645	0
7	0	3.00	4.00	1.00	26.584	13.292	7.385	-43.370	0
8	0	2.00	7.00	3.00	36.868	18.434	16.285	-64.520	0
9	0	9.00	5.00	3.00	65.594	32.797	9.422	-21.485	0
10	0	9.00	6.00	7.00	76.188	38.094	19.593	-23.835	0
11	0	9.00	7.00	8.00	81.127	40.564	21.323	-26.890	0

232 free entries left

```
# th 7.382    3.433   33.490  -69.665       0
# th 7.382    3.433   33.490  -69.665
# dr
# sv
Step scan parameters
 Nstep     :   41 ? 181
 Stepwidths:   0.1000   0.0000   0.0000   0.0000 ?

 Time/step :   0.50 sec ?

Data stored in chon.pm4
# th 50 24.85
# dr
# pp
     50.0000   24.8500   33.4900  -69.6650
# sv
Step scan parameters
 Nstep     :  181 ?

 Stepwidths:   0.1000   0.0000   0.0000   0.0000 ? 0.4 0.2 0 0
 Time/step :   0.50 sec ?

Data stored in chon.pm4
# ss
Scan centre =    50.0000   24.8500   33.4900  -69.6650
Scan range  =    72.0000   36.0000    0.0000    0.0000  (181 steps, stepmode)
   18  15314 |              o              :
    4  15082 |   o                         :
   10  15430 |        o                    :
   14  14974 |           o                 :
   14  15302 +           o                 :
    6  15376 |     o                       :
   14  15314 |           o                 :
   14  15166 |           o                 :
    8  15324 |       o                     :
    4  15056 +   o                         :
   10  14892 |        o                    :
   16  15234 |             o               :
    6  15010 |     o                       :
   10  15296 |        o                    :
   16  15194 +             o               :
   18  14858 |              o              :
   10  15264 |        o                    :
   20  15080 |                o            :
```

```
 8 14858 |    o             :
18 15028 +         o         :
 8 14994 |    o             :
 8 14908 |    o             :
10 15160 |     o             :
12 14732 |      o            :
 8 14758 +    o             :
10 14738 |     o             :
20 14566 |          o        :
34 14628 |                 o :
40 14518 |                  : o
24 14462 +            o      :
 8 14516 |    o             :
12 14354 |      o            :
 8 14342 |    o             :
 2 14288 | o                :
 6 14316 +   o              :
 6 14142 |   o              :
 8 13676 |    o             :
14 14070 |       o          :
12 13574 |      o           :
 6 13398 +   o             :
14 13314 |       o          :
14 12840 |       o         :
 8 12798 |    o            :
 8 12846 |    o            :
 2 12478 + o               :
 6 12328 |   o             :
16 11936 |        o        :
 6 12098 |   o            :
 6 11800 |   o            :
12 11490 +      o          :
20 11014 |          o     :
 4 10440 |  o            :
 8 10196 |    o          :
16  9912 |        o      :
10  9872 +     o         :
 8  9550 |    o         :
 6  9220 |   o         :
10  9456 |     o         :
 8  9382 |    o         :
16  9018 +        o     :
16  9108 |        o     :
 8  8892 |    o        :
```

```
 8  8956 |      o             :
14  8902 |           o        :
 6  8852 +    o               :
 6  8822 |    o               :
 2  8962 | o                  :
 4  8758 |  o                 :
 8  8736 |      o             :
 6  8732 +    o               :
 8  8748 |      o             :
 6  8672 |    o               :
 2  8714 | o                  :
 8  8940 |      o             :
 6  8512 +    o               :
18  8676 |              o     :
14  8916 |           o        :
30  8578 |                    :  o
12  8704 |         o          :
 4  8738 +  o                 :
14  8604 |           o        :
 8  8788 |      o             :
18  8706 |              o     :
 2  8750 | o                  :
10  8522 +       o            :
14  8784 |           o        :
 8  8504 |      o             :
 6  8828 |    o               :
 4  8568 |  o                 :
 4  8720 +  o                 :
 8  8646 |      o             :
 6  8526 |    o               :
10  8708 |       o            :
 6  8668 |    o               :
10  8336 +       o            :
16  8484 |            o       :
12  8504 |         o          :
16  8594 |            o       :
 6  8592 |    o               :
 2  8634 + o                  :
14  8520 |           o        :
 6  8698 |    o               :
10  8508 |       o            :
 8  8678 |      o             :
14  8498 +           o        :
 4  8724 |  o                 :
```

```
10  8498 |     o       :
 2  8682 | o           :
 6  8526 |   o         :
 6  8426 +   o         :
 8  8574 |    o        :
16  8548 |        o    :
 8  8750 |    o        :
10  8678 |     o       :
10  8486 +     o       :
18  8550 |         o   :
 8  8454 |    o        :
 6  8838 |   o         :
10  8576 |     o       :
10  8902 +     o       :
12  8604 |      o      :
 4  8622 |  o          :
 8  8812 |    o        :
16  8722 |        o    :
14  8666 +       o     :
10  8762 |     o       :
 8  8800 |    o        :
 4  8754 |  o          :
 4  8838 |  o          :
 4  8576 +  o          :
18  8516 |         o   :
10  8562 |     o       :
 6  8574 |   o         :
 2  8608 | o           :
10  8838 +     o       :
 4  8780 |  o          :
10  8698 |     o       :
 6  8700 |   o         :
 8  8630 |    o        :
 8 15276 +    o            :
 8 15202 |    o            :
 4 15408 |  o              :
 6 15560 |   o             :
10 15178 |     o           :
20 15620 +          o      :
22 15228 |           o     :
10 15330 |     o           :
 6 15516 |   o             :
 6 14970 |   o             :
 2 15176 + o               :
```

```
   4  15234 |   o                        :
   2  15366 | o                          :
   6  15366 |     o                      :
   6  15438 |     o                      :
   6  15160 +     o                      :
   6  15276 |     o                      :
   4  15296 |   o                        :
   4  15252 |   o                        :
   6  15022 |     o                     :
   6  15124 +     o                      :
  14  15168 |           o                 :
   6  15128 |     o                      :
   2  15116 | o                          :
  10  15184 |         o                   :
  12  14868 +         o                  :
   8  15152 |       o                    :
  10  14872 |         o                  :
  10  14906 |         o                  :
   6  15100 |     o                      :
   8  15030 +       o                   :
  16  15052 |             o              :
   8  14914 |       o                   :
  12  15178 |           o                 :
  16  14888 |             o              :
   6  14974 +     o                     :
  10  14972 |         o                  :
   4  14960 |   o                       :
   8  14552 |       o                   :
  14  14666 |           o                :
   6  14854 +     o                     :
   4  14558 |   o                       :
Centre at point  61.461      Angles =    38.1842   18.9421   33.4900  -69.6650
I(int)=   -42.56  Sigma=69.10  FWHM=10.1638  Imax( 29)=    26.79  Temp=   0.00K

# th 25.6 12.8
# dr
# pp
     25.6000   12.8000   33.4900  -69.6650
# sv
Step scan parameters
 Nstep     :  181 ? 41
 Stepwidths:   0.4000   0.2000   0.0000   0.0000 ? 0 0.1 0 0
 Time/step :   0.50 sec ?
```

Data stored in chon.pm4
ss
Scan centre =   25.6000   12.8000   33.4900 -69.6650
Scan range =   0.0000   4.0000   0.0000   0.0000 (41 steps, stepmode)

```
   18   8550 |               o      :
   16   8822 |             o         :
   16   8746 |             o         :
   18   8602 |               o      :
   26   8520 +                      : o
   12   8440 |         o            :
   10   8466 |       o              :
   26   8876 |                      :o
   12   8990 |         o             :
   14   8660 +           o           :
   14   8562 |           o          :
   26   8514 |                      : o
   14  13390 |           o               :
   18  15166 |              o             :
   10  15176 +       o                    :
   26  15094 |                     o       :
   28  15358 |                       o     :
   20  15158 |                o           :
   10  15152 |       o                    :
   28  15438 +                       o     :
   22  15308 |                  o         :
   22  15244 |                  o         :
   18  15260 |              o             :
   10  15180 |       o                    :
   20  15180 +                o           :
   16  15080 |            o               :
   24  15368 |                    o       :
   18  15124 |              o             :
   20  15226 |                o           :
   14  15122 +          o                 :
   14  15008 |          o                 :
   16  14752 |            o              :
   22  15272 |                  o         :
   20  14832 |                o          :
   22  15170 +                  o         :
   20  15122 |                o           :
   22  15438 |                  o         :
   18  15074 |              o             :
   18  15246 |              o             :
   22  15136 +                  o         :
```

16 14862 | o :
Centre at point 2.333 Angles = 25.6000 10.9333 33.4900 -69.6650
I(int)= -0.25 Sigma=40.80 FWHM=0.2910 Imax(17)= 9.69 Temp= 0.00K
** Unsuitable scanwidth
# th 25.6 12.65
# sv
Step scan parameters
Nstep : 41 ?

Stepwidths: 0.0000 0.1000 0.0000 0.0000 ? 0.1 0 0 0
Time/step : 0.50 sec ?

Data stored in chon.pm4
ss
Scan centre = 25.6000 12.6500 33.4900 -69.6650
Scan range = 4.0000 0.0000 0.0000 0.0000 (41 steps, stepmode)
10 8616 | o :
6 8770 | o :
20 8636 | o :
6 8740 | o :
14 8432 + o :
16 8620 | o :
20 8482 | o :
22 8670 | o :
20 14960 | o :
22 15202 + o :
26 15256 | o :
24 15108 | o :
22 15176 | o :
20 14950 | o :
28 15322 + o :
32 15256 | o :
26 14914 | o :
32 15214 | o :
22 15018 | o :
12 15054 + o :
20 15290 | o :
20 15336 | o :
8 15126 | o :
22 15254 | o :
14 15122 + o :
14 15198 | o :
18 14978 | o :
10 15130 | o :

```
  8  15030 |      o                    :
 12  15096 +         o                 :
  6  15046 |    o                      :
 14  15120 |           o               :
  8  15096 |      o                    :
 16  14866 |             o             :
  2  15042 + o                         :
 12  14982 |         o                 :
  6  14906 |    o                      :
  8  14864 |      o                    :
  6  15090 |    o                      :
  6  14934 +    o                      :
  8  15162 |      o                    :
Centre at point  21.000      Angles =    25.6000   12.6500   33.4900  -69.6650
I(int)=    23.48  Sigma=30.53  FWHM=1.0012  Imax( 18)=    21.80  Temp=   0.00K
** Int. too low
# sv
Step scan parameters
 Nstep     :   41 ? 31
 Stepwidths:   0.1000   0.0000   0.0000   0.0000 ? 0.1 0.05 0 0
 Time/step :   0.50 sec ?

Data stored in chon.pm4
# ss
Scan centre =    25.6000   12.6500   33.4900  -69.6650
Scan range  =     3.0000    1.5000    0.0000    0.0000  ( 31 steps, stepmode)
  8  15192 |      o                    :
 18  15192 |              o            :
 20  15122 |                o          :
  8  14958 |      o                  :
 20  14972 +                o        :
 18  15186 |              o            :
 26  14942 |                    o    :
 28  14858 |                      o  :
 22  14906 |                  o      :
 18  14800 +              o         :
 22  14838 |                  o      :
 26  14972 |                    o    :
 32  15224 |                        o  :
 18  14758 |              o         :
 18  14658 +              o       :
 16  14934 |             o           :
  8  14770 |      o                 :
 12  14958 |         o               :
```

```
   22  14966 |                  o         :
   16  14784 +             o            :
    0  14864 |o                         :
    8  14488 |       o               :
   10  14830 |         o                :
   14  14616 |            o            :
   10  14864 +         o                 :
    4  14742 |   o                     :
   10  14538 |         o              :
    6  14406 |     o               :
    8  14570 |       o               :
    6  14624 +     o                  :
   16  14448 |             o         :
Centre at point  16.000      Angles =    25.6000   12.6500   33.4900  -69.6650
I(int)=     4.80  Sigma=30.16  FWHM=0.3994  Imax( 13)=    19.35  Temp=   0.00K
** Int. too low
# th 45.2 22.5
# ss
Scan centre =    45.2000   22.5000   33.4900  -69.6650
Scan range  =     3.0000    1.5000    0.0000    0.0000  ( 31 steps, stepmode)
   10   8706 |        o           :
   12   8666 |          o         :
   18   8790 |               o      :
    8   9010 |       o              :
   10   8766 +        o           :
   12   8662 |          o         :
   14   8610 |            o         :
   18   8800 |               o      :
   18   8980 |               o      :
    8   8850 +       o              :
   14   8886 |            o          :
   18   8598 |               o     :
   14   8646 |            o         :
   16   8740 |             o        :
   10  15374 +         o                     :
   18  15348 |               o              :
   16  15100 |             o                :
   16  14920 |             o               :
   12  15230 |          o                   :
   16  15352 +             o                :
   18  15438 |               o              :
   22  15122 |                  o           :
   14  15144 |            o                 :
   14  15304 |            o                 :
```

```
   20  15212 +                o             :
    8  15086 |      o                       :
    4  15158 |   o                          :
   12  15022 |          o                   :
   14  15064 |            o                 :
    4  15210 +   o                          :
    6  14950 |     o                        :
Centre at point  16.000     Angles =    45.2000   22.5000   33.4900  -69.6650
I(int)=    5.51  Sigma=27.31  FWHM=0.0556  Imax( 22)=    13.03  Temp=   0.00K
** Int. too low
# x
# x?

List 1    mode = 4    n =   1  (  0)

Seq# Stat    H      K      L    2Theta    Omega     Chi      Phi     Int.
 12   0    0.00   0.00   2.00    7.382    3.433   33.490  -69.665        0

List 2    mode = 1    n =  11  (  0)

Seq# Stat    H      K      L    2Theta    Omega     Chi      Phi     Int.
  1   0    3.00   1.00   1.00   19.907    9.953    9.845  -13.290        0
  2   0    4.00   0.00   0.00   24.187   12.094    0.000    0.000        0
  3   0    0.00   4.00   0.00   18.016    9.008    0.000  -90.000        0
  4   0    0.00   0.00   4.00   14.147    7.073   73.760    0.000        0
  5   0    5.00   3.00   1.00   34.479   17.239    5.723  -23.465        0
  6   0    5.00   2.00   0.00   31.726   15.863    0.000  -16.645        0
  7   0    3.00   4.00   1.00   26.584   13.292    7.385  -43.370        0
  8   0    2.00   7.00   3.00   36.868   18.434   16.285  -64.520        0
  9   0    9.00   5.00   3.00   65.594   32.797    9.422  -21.485        0
 10   0    9.00   6.00   7.00   76.188   38.094   19.593  -23.835        0
 11   0    9.00   7.00   8.00   81.127   40.564   21.323  -26.890        0

232 free entries left
# th 7.382    3.433   33.490  -69.665
# dr
# co 1 1
   159  13778
# cp
Centring parameters
 Measuring time  :  1.00 sec ?

 Omega halfwidth :  0.150 ?
```

```
 Delta(2theta)   :  0.030 ?

 Delta(chi)     :  0.050 ?

 Tolerance      :  0.010 ?

# cp
Centring parameters
 Measuring time  :  1.00 sec ?

 Omega halfwidth :  0.150 ? 0.5
 Delta(2theta)   :  0.030 ? 0.3
 Delta(chi)     :  0.050 ? 0.5
 Tolerance      :  0.010 ?

Data stored in chon.pm4
# cr
** No maximum found
# x?

List 1    mode = 4    n =   1  (  0)

Seq# Stat    H      K      L    2Theta    Omega     Chi      Phi     Int.
 12   0    0.00   0.00   2.00    7.382    3.433   33.490  -69.665       0

List 2    mode = 1    n =  11  (  0)

Seq# Stat    H      K      L    2Theta    Omega     Chi      Phi     Int.
  1   0    3.00   1.00   1.00   19.907    9.953    9.845  -13.290       0
  2   0    4.00   0.00   0.00   24.187   12.094    0.000    0.000       0
  3   0    0.00   4.00   0.00   18.016    9.008    0.000  -90.000       0
  4   0    0.00   0.00   4.00   14.147    7.073   73.760    0.000       0
  5   0    5.00   3.00   1.00   34.479   17.239    5.723  -23.465       0
  6   0    5.00   2.00   0.00   31.726   15.863    0.000  -16.645       0
  7   0    3.00   4.00   1.00   26.584   13.292    7.385  -43.370       0
  8   0    2.00   7.00   3.00   36.868   18.434   16.285  -64.520       0
  9   0    9.00   5.00   3.00   65.594   32.797    9.422  -21.485       0
 10   0    9.00   6.00   7.00   76.188   38.094   19.593  -23.835       0
 11   0    9.00   7.00   8.00   81.127   40.564   21.323  -26.890       0

232 free entries left
# ca 1 1 1
Angles =     8.9775    4.4887   22.1875  -32.6950
# ca 0 0 1
```

```
Angles =    3.5282   1.7641  73.7600   0.0000
# ca 0 0 2
Angles =    7.0598   3.5299  73.7600   0.0000
# ca 0 2 0
Angles =    8.9800   4.4900   0.0000 -90.0000
# ca 2 0 0
Angles =   12.0262   6.0131   0.0000   0.0000
# ca 1 0 1
Angles =    7.7720   3.8860  25.8575   0.0000
# ca 1 0 0
Angles =    6.0048   3.0024   0.0000   0.0000
# ca 2 0 1
Angles =   13.4558   6.7279  14.6125   0.0000
# ca 1 0 2
Angles =   10.4782   5.2391  40.3425   0.0000
# ca 0 2 1
Angles =    9.6509   4.8255  20.5700 -83.7250
# ix 1
** IX needs at least 4 reflections
# pi 1
Replace indices in list(s) (Y or N) ? y

Seq#    H     K     L
 12   0.15  1.29  1.20 *
# ca 0 1.5 1.5
Angles =    8.5676   4.2838  36.4075 -77.5950
# ca 0 1.3 1.3
Angles =    7.4235   3.7118  36.4075 -77.5950
# x?

List 1   mode = 4   n =   1  (  0)

Seq# Stat   H     K     L  2Theta   Omega    Chi     Phi    Int.
 12  8   0.15  1.29  1.20   7.382   3.433  33.490 -69.665       0

List 2   mode = 1   n =  11  (  0)

Seq# Stat   H     K     L  2Theta   Omega    Chi     Phi    Int.
  1  0   3.00  1.00  1.00  19.907   9.953   9.845 -13.290       0
  2  0   4.00  0.00  0.00  24.187  12.094   0.000   0.000       0
  3  0   0.00  4.00  0.00  18.016   9.008   0.000 -90.000       0
  4  0   0.00  0.00  4.00  14.147   7.073  73.760   0.000       0
  5  0   5.00  3.00  1.00  34.479  17.239   5.723 -23.465       0
  6  0   5.00  2.00  0.00  31.726  15.863   0.000 -16.645       0
```

```
 7  0   3.00  4.00  1.00  26.584  13.292   7.385  -43.370      0
 8  0   2.00  7.00  3.00  36.868  18.434  16.285  -64.520      0
 9  0   9.00  5.00  3.00  65.594  32.797   9.422  -21.485      0
10  0   9.00  6.00  7.00  76.188  38.094  19.593  -23.835      0
11  0   9.00  7.00  8.00  81.127  40.564  21.323  -26.890      0

232 free entries left
# ca 3 3 3
Angles =   27.1585  13.5792  22.1875  -32.6950
# ca 4 0 0
Angles =   24.1874  12.0937   0.0000   0.0000
# ca 2 -4 -4
Angles =   24.0588  12.0294  -34.5600  65.8150
# ca 1 0 4
Angles =   16.8687   8.4344  53.7100   0.0000
# ca 4 0 4
Angles =   31.4579  15.7289  25.8575   0.0000
# ca 4 0 0
Angles =   24.1874  12.0937   0.0000   0.0000
# dr
# co 1 1
   28   8736
# ca 4 0 0
Angles =   24.1874  12.0937   0.0000   0.0000
# ch 61 328.5
# dr
# co 5 1
   76  15018    74  14953    86  14973    80  14811    76  14860
Average Intensity =     78.400 cps, standard deviation =    4.271 cps
Average Monitor   =  14923.000 cps, standard deviation =   76.076 cps
# sv
Step scan parameters
 Nstep    :  31 ?

 Stepwidths:  0.1000  0.0500  0.0000  0.0000 ? 0 0 0 0.1
 Time/step :  0.50 sec ?

Data stored in chon.pm4
# ss
Scan centre =   24.1872  12.0935  61.0000  -31.5000
Scan range  =   0.0000   0.0000   0.0000   3.0000  ( 31 steps, stepmode)
   18  13526 |       o                 :
   34  13182 |               o         :
   30  13420 |            o            :
```

```
   32  12966 |                 o            :
   30  12934 +                o             :
   44  13004 |                       o      :
   52  12504 |                            :o
   54  12292 |                            : o
   54  12360 |                            : o
   46  12146 +                        o   :
   66  11916 |                           :        o
   76  11732 |                           :             o
   96  11456 |                          :                        o
   68  11062 |                         :            o
   88  11060 +                         :                     o
   60  10602 |                        :        o
   78  10398 |                        :                o
   62  10100 |                       :          o
   80   9934 |                      :                  o
   56   9798 +                      :      o
   48   9510 |                     :   o
   36   9512 |                  o    :
   44   9196 |                    :  o
   32   9328 |                o      :
   42   8924 +                    :o
   42   9288 |                    o :
   20   8916 |         o            :
   30   8958 |              o       :
   20   8986 |         o            :
   20   8820 +         o            :
   18   8632 |       o             :
Centre at point  15.382      Angles =    24.1872   12.0935   61.0000  -31.5618
I(int)=    69.48  Sigma=45.05  FWHM=0.5212  Imax( 13)=    69.97  Temp=   0.00K

# sv
Step scan parameters
 Nstep    :   31 ?

 Stepwidths:   0.0000   0.0000   0.0000   0.1000 ? 0 0 0.2 0
 Time/step :   0.50 sec ?

Data stored in chon.pm4
# ss
Scan centre =    24.1872   12.0935   61.0000  -31.5600
Scan range  =     0.0000    0.0000    6.0000    0.0000  ( 31 steps, stepmode)
   30   8660 |            o           :
   44   8756 |                  o       :
```

```
  46   8486 |                   :  o
  48   8608 |                     o :
  54   8650 +                      : o
  54   8746 |                       o :
  64   8660 |                      :      o
  58   8748 |                       : o
  78   8746 |                       :           o
  82   8842 +                         :          o
  66   8854 |                         :   o
  86   8670 |                      :                 o
 104   8532 |                    :                          o
  76   8750 |                       :          o
  74   8496 +                   :              o
  66   8602 |                     :        o
  82   8480 |                   :                  o
  84   8572 |                    :                 o
  74   8628 |                     :           o
  78   8562 +                   :              o
  76   8702 |                      :          o
  62   8648 |                      :     o
  64   8646 |                      :      o
  66   8590 |                    :        o
  48   8726 +                   o    :
  44   8796 |                  o        :
  32   8656 |             o          :
  28   8596 |           o           :
  32   8696 |             o           :
  48   8704 +                   o   :
  30   8658 |            o           :
Centre at point  15.906      Angles =    24.1872   12.0935   60.9813  -31.5600
I(int)=   132.56  Sigma=56.62  FWHM=2.0343  Imax( 13)=    63.60  Temp=   0.00K

# sv
Step scan parameters
 Nstep    :   31 ?

 Stepwidths:   0.0000   0.0000   0.2000   0.0000 ? 0.1 0 0 0
 Time/step :   0.50 sec ?

Data stored in chon.pm4
# ss
Scan centre =    24.1872   12.0935   60.9825  -31.5600
Scan range  =     3.0000    0.0000    0.0000    0.0000  ( 31 steps, stepmode)
  12   8606 |      o                    :
```

```
   12   8586 |      o                    :
   16   8548 |       o                  :
   24   8758 |           o                  :
   26   8800 +           o                  :
   36   8454 |                o      :
   12   8698 |      o                     :
   32   8484 |              o         :
   22   8686 |          o                  :
   34   8700 +               o            :
   42   8518 |                   o    :
   32   8730 |              o             :
   36   8576 |                o        :
   68   8636 |                         :        o
   70   8572 +                         :         o
   96   8420 |                     :                          o
   62   8440 |                      :         o
   72   8782 |                            :       o
   84   8468 |                      :                   o
   68   8600 +                         :        o
   66   8822 |                             :   o
   82   8656 |                          :             o
  100   8736 |                          :                      o
   60   8490 |                       :       o
  104   8616 +                         :                         o
   68   8672 |                          :       o
   68   8374 |                    :             o
  100   8644 |                          :                      o
   76   8766 |                            :         o
   76   8514 +                       :              o
   66   8804 |                             :   o
```
Centre at point 19.357   Angles =   24.5229   12.0935   60.9825 -31.5600

I(int)=   24.64 Sigma=60.16 FWHM=0.0909 Imax(16)=   48.40 Temp=   0.00K

** Unsuitable scanwidth

sv

Step scan parameters

Nstep   :   31 ?

Stepwidths: 0.1000   0.0000   0.0000   0.0000 ? 0.2 0 0 0

Time/step :   0.50 sec ?

Data stored in chon.pm4

th 25

ss

Scan centre =   25.0000   12.0935   60.9825 -31.5600

```
Scan range  =     6.0000    0.0000    0.0000    0.0000  ( 31 steps, stepmode)
   16  14920 |       o                    :
   22  15004 |          o                 :
   10  15216 |   o                        :
   18  15096 |        o                   :
   16  14988 +       o                    :
   26  14732 |           o                :
   18  14940 |        o                   :
   22  14990 |          o                 :
   24  14702 |          o                :
   52  14638 +                      o    :
   84  14786 |                          :            o
   82  14524 |                        :            o
   84  14704 |                         :           o
   72  14588 |                         :      o
   86  14572 +                         :            o
   84  15008 |                           :          o
  110  14504 |                        :                         o
   64  14662 |                         :   o
   58  14332 |                       :  o
   32  14732 +              o             :
   22  14726 |          o                :
   28  14474 |            o             :
   18  14134 |        o                :
   22  14162 |          o              :
   12  13930 +     o                  :
   12  13916 |     o                  :
   16  14202 |       o                :
   18  14132 |        o               :
   16  13826 |       o              :
   28  13482 +            o         :
   26  13494 |           o          :
Centre at point  14.282      Angles =    24.6564   12.0935   60.9825  -31.5600
I(int)=   124.28  Sigma=40.55  FWHM=1.5272  Imax( 17)=    91.23  Temp=   0.00K

# sv
Step scan parameters
 Nstep     :   31 ?

 Stepwidths:   0.2000   0.0000   0.0000   0.0000 ? 0 0.1 0 0
 Time/step :   0.50 sec ?

Data stored in chon.pm4
# ss
```

```
Scan centre =    24.6564   12.0935   60.9825  -31.5600
Scan range  =     0.0000    3.0000    0.0000    0.0000  ( 31 steps, stepmode)
   40   8822 |                         o         :
   34   8518 |                     o        :
   40   8840 |                         o         :
   40   8720 |                         o       :
   34   8730 +                     o           :
   42   8808 |                          o       :
   42   8576 |                          o    :
   36   8690 |                      o         :
   50   8754 |                               :o
   38   8678 +                        o       :
   36   8724 |                      o          :
   42   8538 |                          o   :
   58   8480 |                          :           o
   52   8680 |                             :    o
   80   8904 +                                 :                  o
   92   8798 |                               :                            o
   86   8632 |                             :                          o
   88   8418 |                         :                               o
   50   8784 |                               :o
   60   8668 +                             :         o
   48   8318 |                       :       o
   34   8600 |                     o         :
   24   8530 |               o              :
   52   8730 |                              :   o
   26   8680 +                o               :
   44   8584 |                            o  :
   26   8598 |                o              :
   40   8916 |                         o          :
   24   8756 |               o                  :
   30   8706 +                   o             :
   24   8908 |               o                    :
Centre at point  16.000      Angles =    24.6564   12.0935   60.9825  -31.5600
I(int)=    38.28  Sigma=50.66  FWHM=0.4294  Imax( 16)=    58.80  Temp=   0.00K
** Int. too low
# sv
Step scan parameters
 Nstep     :   31 ?

 Stepwidths:   0.0000   0.1000   0.0000   0.0000 ? 0 0 0 0.1
 Time/step :   0.50 sec ?

Data stored in chon.pm4
```

```
# ss
Scan centre =    24.6564   12.0935   60.9825  -31.5600
Scan range  =     0.0000    0.0000    0.0000    3.0000  ( 31 steps, stepmode)
   28  15430 |           o                  :
   32  15074 |             o             :
   44  15252 |                   o          :
   38  15336 |                o             :
   58  15022 +                         o  :
   52  14916 |                      o    :
   56  15084 |                        o   :
   54  14884 |                       o  :
   66  15218 |                             : o
   64  14986 +                         :   o
   90  15376 |                              :          o
   92  14960 |                          :               o
   88  14996 |                          :             o
   88  15330 |                            :          o
  118  15060 +                          :                          o
   98  15312 |                            :              o
   70  14840 |                         :      o
   88  14966 |                          :             o
   86  15016 |                           :            o
   92  15098 +                          :             o
   58  14904 |                         :o
   78  14890 |                         :          o
   42  14824 |                   o       :
   56  14828 |                        o :
   38  14590 +                o       :
   46  14784 |                   o     :
   50  14882 |                     o    :
   38  14756 |                o        :
   46  14624 |                   o   :
   36  14610 +               o       :
   32  14522 |             o        :
Centre at point  15.278      Angles =    24.6564   12.0935   60.9825  -31.6322
I(int)=    67.58  Sigma=56.13  FWHM=0.6096  Imax( 15)=    77.82  Temp=   0.00K

# ah
H,K,L for vector 1 ? 0 0 2
H,K,L for vector 2 ? 4 0 0
Vector 1: X Y Z =   0.037905 -0.000000  0.130132  Length =  0.13554
Vector 2: X Y Z =   0.461224  0.000000  0.000000  Length =  0.46122
Angle between vectors =   73.76
# xi 1
```

```
** List 1 already exists
# xa 1
Enter HKL and Angles - Terminate with a blank line
Seq#  13 ?

# x?

List 1    mode = 4    n =   1  (  0)

Seq# Stat    H      K      L    2Theta    Omega     Chi      Phi     Int.
 12   8    0.15   1.29   1.20    7.382    3.433   33.490  -69.665       0

List 2    mode = 1    n =  11  (  0)

Seq# Stat    H      K      L    2Theta    Omega     Chi      Phi     Int.
  1   0    3.00   1.00   1.00   19.907    9.953    9.845  -13.290       0
  2   0    4.00   0.00   0.00   24.187   12.094    0.000    0.000       0
  3   0    0.00   4.00   0.00   18.016    9.008    0.000  -90.000       0
  4   0    0.00   0.00   4.00   14.147    7.073   73.760    0.000       0
  5   0    5.00   3.00   1.00   34.479   17.239    5.723  -23.465       0
  6   0    5.00   2.00   0.00   31.726   15.863    0.000  -16.645       0
  7   0    3.00   4.00   1.00   26.584   13.292    7.385  -43.370       0
  8   0    2.00   7.00   3.00   36.868   18.434   16.285  -64.520       0
  9   0    9.00   5.00   3.00   65.594   32.797    9.422  -21.485       0
 10   0    9.00   6.00   7.00   76.188   38.094   19.593  -23.835       0
 11   0    9.00   7.00   8.00   81.127   40.564   21.323  -26.890       0

232 free entries left
# av
2Theta, Omega, Chi, Phi for vector 1 ? 7.382    3.433   33.490  -69.665
2Theta, Omega, Chi, Phi for vector 2 ? 24.6564   12.0935   60.9825  -31.6322
Vector 1: X Y Z =   0.040473  0.111045  0.078198  Length =  0.14172
Vector 2: X Y Z =   0.193116  0.121217  0.411025  Length =  0.47003
Angle between vectors =   36.69
# ah
H,K,L for vector 1 ? 2 0 0
H,K,L for vector 2 ?

# ah
H,K,L for vector 1 ? 0 0 2
H,K,L for vector 2 ? -4 0 0
Vector 1: X Y Z =   0.037905 -0.000000  0.130132  Length =  0.13554
Vector 2: X Y Z =  -0.461224  0.000000  0.000000  Length =  0.46122
Angle between vectors =  106.24
```

```
# ah
H,K,L for vector 1 ? 0 0 -2
H,K,L for vector 2 ? 4 0 0
Vector 1: X Y Z =  -0.037905  0.000000 -0.130132  Length =  0.13554
Vector 2: X Y Z =   0.461224  0.000000  0.000000  Length =  0.46122
Angle between vectors =  106.24
# ah
H,K,L for vector 1 ? 0 0 -2
H,K,L for vector 2 ? -4 0 0
Vector 1: X Y Z =  -0.037905  0.000000 -0.130132  Length =  0.13554
Vector 2: X Y Z =  -0.461224  0.000000  0.000000  Length =  0.46122
Angle between vectors =   73.76
# xa 1
Enter HKL and Angles - Terminate with a blank line
Seq#  13 ? 4 0 0 24.6564   12.0935   60.9825  -31.6322
Seq#  14 ?

  1 entries added to list 1   New n =   2
231 free entries left
# ca 4 0 0
Angles =    24.1874   12.0937    0.0000    0.0000
# ch 24 119
# dr
# co 1 1
   56  11164
# sv
Step scan parameters
 Nstep     :   31 ?

 Stepwidths:   0.0000   0.0000   0.0000   0.1000 ?

 Time/step :   0.50 sec ?

# ss
Scan centre =    24.1872   12.0935   24.0000  119.0000
Scan range  =     0.0000    0.0000    0.0000    3.0000  ( 31 steps, stepmode)
   54   9280 |                          :          o
   62   9470 |                            :              o
   50   9324 |                           :      o
   66   9016 |                        :                    o
   48   8882 +                       :         o
   52   8682 |                      :             o
   42   8752 |                      :     o
   50   8664 |                     :            o
```

```
   50   8966 |                        :          o
   48   8638 +                      :           o
   56   8926 |                        :              o
   50   8812 |                       :          o
   66   8750 |                      :                      o
   62   8772 |                      :                   o
   54   8660 +                      :               o
   64   8732 |                      :                    o
   52   8588 |                      :             o
   52   8722 |                      :             o
   54   8448 |                    :                 o
   44   8802 +                       :      o
   52   8706 |                      :             o
   48   8732 |                      :         o
   62   8454 |                    :                      o
   70   8692 |                      :                          o
   50   8544 +                     :            o
   42   8752 |                      :     o
   64   8686 |                      :                    o
   70   8684 |                      :                          o
   38   8686 |                      :  o
   54   8608 +                      :               o
   54   8946 |                        :             o
Centre at point  14.750      Angles =    24.1872   12.0935   24.0000  118.8750
I(int)=    -5.60  Sigma=64.72  FWHM=0.3271  Imax( 24)=    14.00  Temp=   0.00K

# sv
Step scan parameters
 Nstep     :   31 ?

 Stepwidths:   0.0000   0.0000   0.0000   0.1000 ? 0 0 0.2 0
 Time/step :   0.50 sec ?

Data stored in chon.pm4
# ss
Scan centre =    24.1872   12.0935   24.0000  118.8750
Scan range  =     0.0000    0.0000    6.0000    0.0000  ( 31 steps, stepmode)
   52   8466 |                     :          o
   54   8594 |                      :         o
   50   8504 |                      :        o
   50   8784 |                          :    o
   64   8670 +                        :              o
   42   8752 |                        o :
   38   8720 |                     o    :
```

```
  56  8620 |                           :           o
  66  8572 |                          :                  o
  62  8968 +                                 :         o
  58  8464 |                        :               o
  84  8422 |                       :                                 o
  40  8630 |                           :o
  70  8504 |                         :                      o
  54  8556 +                          :           o
  42  8768 |                            o   :
  56  8656 |                            :          o
  58  8570 |                          :             o
  40  8514 |                         :   o
  48  8522 +                         :       o
  56  8580 |                          :            o
  52  8616 |                           :        o
  44  8686 |                            : o
  40  8482 |                        :    o
  36  8640 +                         o   :
  34  8532 |                       o   :
  54  8462 |                        :             o
  40  8648 |                           :o
  56  8524 |                         :             o
  52  8502 +                         :          o
  40  8792 |                           o    :
```

Centre at point 1.000 Angles = 24.1872 12.0935 21.0000 118.8750

I(int)= 0.16 Sigma=61.97 FWHM=2.2729 Imax(12)= 31.94 Temp= 0.00K

** Unsuitable scanwidth

ch 22

sv

Step scan parameters

Nstep : 31 ?

Stepwidths: 0.0000 0.0000 0.2000 0.0000 ?

Time/step : 0.50 sec ?

ca 1 3 1

Angles = 15.5845 7.7922 12.5925 -62.5550

ca 3 3 1

Angles = 23.6900 11.8450 8.2800 -35.3150

ca 3 3 2

Angles = 25.2257 12.6129 15.7075 -33.9600

ca 1 5 1

Angles = 23.9067 11.9533 8.2050 -72.6900

ca 1 4 2
Angles =   20.8854   10.4427   19.0350 -66.0350
#

ca 0 4 4
Angles =   22.9794   11.4897   36.4075 -77.5950
ca 0 5 2
Angles =   23.6788   11.8394   16.7450 -84.9700
ca 0 5 3
Angles =   24.9964   12.4982   24.1875 -82.4800
ah
H,K,L for vector 1 ? 0 0 2
H,K,L for vector 2 ? 0 5 3
Vector 1: X Y Z =   0.037905 -0.000000 0.130132 Length = 0.13554
Vector 2: X Y Z =   0.056858 0.430849 0.195198 Length = 0.47641
Angle between vectors =   64.74
ah
H,K,L for vector 1 ? 0 0 2
H,K,L for vector 2 ? 0 5 2
Vector 1: X Y Z =   0.037905 -0.000000 0.130132 Length = 0.13554
Vector 2: X Y Z =   0.037905 0.430849 0.130132 Length = 0.45167
Angle between vectors =   72.54
x?

List 1   mode = 4   n =   2 (0)

Seq#	Stat	H	K	L	2Theta	Omega	Chi	Phi	Int.
12	8	0.15	1.29	1.20	7.382	3.433	33.490	-69.665	0
13	0	4.00	0.00	0.00	24.656	12.094	60.983	-31.630	0

List 2   mode = 1   n = 11 (0)

Seq#	Stat	H	K	L	2Theta	Omega	Chi	Phi	Int.
1	0	3.00	1.00	1.00	19.907	9.953	9.845	-13.290	0
2	0	4.00	0.00	0.00	24.187	12.094	0.000	0.000	0
3	0	0.00	4.00	0.00	18.016	9.008	0.000	-90.000	0
4	0	0.00	0.00	4.00	14.147	7.073	73.760	0.000	0
5	0	5.00	3.00	1.00	34.479	17.239	5.723	-23.465	0
6	0	5.00	2.00	0.00	31.726	15.863	0.000	-16.645	0
7	0	3.00	4.00	1.00	26.584	13.292	7.385	-43.370	0
8	0	2.00	7.00	3.00	36.868	18.434	16.285	-64.520	0
9	0	9.00	5.00	3.00	65.594	32.797	9.422	-21.485	0
10	0	9.00	6.00	7.00	76.188	38.094	19.593	-23.835	0
11	0	9.00	7.00	8.00	81.127	40.564	21.323	-26.890	0

231 free entries left
av
2Theta, Omega, Chi, Phi for vector 1 ? 7.382   3.433   33.490 -69.665
2Theta, Omega, Chi, Phi for vector 2 ? 24.656   12.094   60.983 -31.630
Vector 1: X Y Z =   0.040473 0.111045 0.078198 Length = 0.14172
Vector 2: X Y Z =   0.193117 0.121200 0.411020 Length = 0.47002
Angle between vectors =   36.69
ah
H,K,L for vector 1 ? 0 0 2
H,K,L for vector 2 ? 1 5 1
Vector 1: X Y Z =   0.037905 -0.000000 0.130132 Length = 0.13554
Vector 2: X Y Z =   0.134259 0.430849 0.065066 Length = 0.45595
Angle between vectors =   77.33
ca 0 2 4
Angles =   16.7807   8.3904   54.1175 -66.2550
ah
H,K,L for vector 1 ? 0 0 2
H,K,L for vector 2 ? 0 4 4
Vector 1: X Y Z =   0.037905 -0.000000 0.130132 Length = 0.13554
Vector 2: X Y Z =   0.075811 0.344679 0.260264 Length = 0.43851
Angle between vectors =   51.82
ah
H,K,L for vector 1 ? 0 0 2
H,K,L for vector 2 ? 0 1 5
Vector 1: X Y Z =   0.037905 -0.000000 0.130132 Length = 0.13554
Vector 2: X Y Z =   0.094764 0.086170 0.325330 Length = 0.34964
Angle between vectors =   14.27
ah
H,K,L for vector 1 ? 0 0 2
H,K,L for vector 2 ? 0 3 5
Vector 1: X Y Z =   0.037905 -0.000000 0.130132 Length = 0.13554
Vector 2: X Y Z =   0.094764 0.258509 0.325330 Length = 0.42620
Angle between vectors =   37.34
ca 0 3 5
Angles =   22.3261   11.1630   49.7575 -69.8700
ca 1 3 5
Angles =   24.4211   12.2106   44.3250 -50.9000
ah
H,K,L for vector 1 ? 0 0 2
H,K,L for vector 2 ? 1 3 5
Vector 1: X Y Z =   0.037905 -0.000000 0.130132 Length = 0.13554
Vector 2: X Y Z =   0.210070 0.258509 0.325330 Length = 0.46561
Angle between vectors =   37.15

```
# ro
Enter H,K,L Omega,Chi,Phi for 2 reflections:
 1 ? 0 0 2 3.433   33.490  -69.665
 2 ? 1 3 5 12.094   60.983  -31.630

Orienting    -0.06997179  0.05478732  0.01953338
Matrix        0.00237786 -0.05060714  0.05303640
(A* B* C*)    0.09161758  0.04315661  0.03739494
Data stored in chon.pm4
# ca 1 0 0
Angles =     6.0048    3.0024   52.6125 -178.0550
# dr
# co 1 1
   101   8752
# ca 4 0 0
Angles =    24.1874   12.0937   52.6125 -178.0550
# dr
# co 1 1
    34   8604
# sv
Step scan parameters
 Nstep    :   31 ?

 Stepwidths:   0.0000   0.0000   0.2000   0.0000 ? 0 0.1 0 0
 Time/step :   0.50 sec ?

Data stored in chon.pm4
# ss
Scan centre =    24.1872   12.0935   52.6125 -178.0550
Scan range  =     0.0000    3.0000    0.0000    0.0000  ( 31 steps, stepmode)
    18   8688 |               o              :
    20   8552 |                 o           :
    38   8638 |                            :   o
    20   8632 |                 o            :
    22   8452 +                   o        :
    22   8164 |                   o    :
    44   8300 |                       :             o
    38   8600 |                            :   o
    34   8630 |                           o :
    32   8370 +                         : o
    20   8574 |                 o           :
    16   8470 |             o              :
    26   8348 |                      o   :
    22   8568 |                   o         :
```

```
   16   8434 +              o               :
   40   8558 |                             :      o
   34   8616 |                              o :
   34   8522 |                             : o
   32   8392 |                           : o
   16   8606 +             o                 :
   42   8606 |                              :       o
   34   8574 |                              :o
   26   8486 |                      o      :
   32   8500 |                            o :
   14   8234 +            o            :
   30   8380 |                           :o
   24   8414 |                    o      :
   18   8426 |               o            :
   24   8632 |                    o          :
   20   8118 +                  o     :
   32   8558 |                            o  :
Centre at point  16.000      Angles =    24.1872   12.0935   52.6125 -178.0550
I(int)=    10.84  Sigma=42.33  FWHM=0.2358  Imax(  7)=    20.40  Temp=   0.00K
** Int. too low
# ca 0 4 0
Angles =    18.0158    9.0079   30.0550   42.7300
# dr
# co 5 1
   25   8651    31   8600    21   8594    27   8625    22   8646
Average Intensity =      25.200 cps, standard deviation =     3.600 cps
Average Monitor   =    8623.200 cps, standard deviation =    23.181 cps
# ca 0 0 6
Angles =    21.2882   10.6441   33.4900  -69.7800
# ca 1 4 5
Angles =    27.2420   13.6210   60.5000  -14.7800
# ca 5 5 1
Angles =    39.1671   19.5836   74.5650  106.6850
# dr
# co 5 1
   17  14966    19  14988    12  15088    18  14987    21  14944
Average Intensity =      17.400 cps, standard deviation =     3.007 cps
Average Monitor   =   14994.600 cps, standard deviation =    49.403 cps
# ca 0 4 4
Angles =    22.9794   11.4897   47.2875   -1.8700
# dr
# co  1 1
   24  14116
# sv
```

```
Step scan parameters
 Nstep    :  31 ?

 Stepwidths:  0.0000  0.1000  0.0000  0.0000 ?

 Time/step :  0.50 sec ?

# ss
Scan centre =   22.9793   11.4895   47.2875   -1.8700
Scan range  =    0.0000    3.0000    0.0000    0.0000  ( 31 steps, stepmode)
   12  12330 |      o           :
   12  12524 |      o           :
   22  12404 |           o      :
   16  12034 |        o        :
   20  11962 +          o      :
   16  11838 |        o        :
   14  11532 |       o         :
   32  11150 |               o :
   10  10802 |     o          :
   20  10726 +          o     :
   16  10198 |        o      :
   22  10144 |           o   :
   26   9692 |            o :
    8   9716 |   o          :
   18   9548 +         o    :
   12   9220 |      o      :
   20   9008 |          o  :
   12   9064 |      o      :
   16   9024 |        o    :
   18   8838 +        o    :
   28   8946 |            :  o
   12   8638 |      o      :
   16   8966 |        o    :
   12   8732 |      o      :
   26   8750 +            :o
   20   8656 |          o  :
   18   8708 |        o    :
   22   8602 |           o :
   20   8896 |          o  :
   18   8758 +        o    :
   22   8790 |           o :
Centre at point  25.681     Angles =    22.9793   12.4576   47.2875   -1.8700
I(int)=   -0.82  Sigma=36.92  FWHM=0.4849  Imax( 8)=    14.91  Temp=   0.00K
** Unsuitable scanwidth
```

```
# ca 4 0 4
Angles =    31.4579   15.7289   59.8525 -132.3100
# dr
# co 1 1
   13   8688
# ca 2 0 0
Angles =    12.0262    6.0131   52.6125 -178.0550
# ca 1 0 0
Angles =     6.0048    3.0024   52.6125 -178.0550
# ca 0 1 0
Angles =     4.4866    2.2433   30.0550   42.7300
# ca 0 2 0
Angles =     8.9800    4.4900   30.0550   42.7300
# ca 1 1 0
Angles =     7.4982    3.7491   69.4350  107.4750
# x?

List 1    mode = 4    n =   2  (  0)

Seq# Stat    H      K      L    2Theta    Omega     Chi      Phi     Int.
 12   8    0.15   1.29   1.20    7.382    3.433   33.490  -69.665       0
 13   0    4.00   0.00   0.00   24.656   12.094   60.983  -31.630       0

List 2    mode = 1    n =  11  (  0)

Seq# Stat    H      K      L    2Theta    Omega     Chi      Phi     Int.
  1   0    3.00   1.00   1.00   19.907    9.953    9.845  -13.290       0
  2   0    4.00   0.00   0.00   24.187   12.094    0.000    0.000       0
  3   0    0.00   4.00   0.00   18.016    9.008    0.000  -90.000       0
  4   0    0.00   0.00   4.00   14.147    7.073   73.760    0.000       0
  5   0    5.00   3.00   1.00   34.479   17.239    5.723  -23.465       0
  6   0    5.00   2.00   0.00   31.726   15.863    0.000  -16.645       0
  7   0    3.00   4.00   1.00   26.584   13.292    7.385  -43.370       0
  8   0    2.00   7.00   3.00   36.868   18.434   16.285  -64.520       0
  9   0    9.00   5.00   3.00   65.594   32.797    9.422  -21.485       0
 10   0    9.00   6.00   7.00   76.188   38.094   19.593  -23.835       0
 11   0    9.00   7.00   8.00   81.127   40.564   21.323  -26.890       0

231 free entries left
# ah
H,K,L for vector 1 ? 1 1 0
H,K,L for vector 2 ? 4 0 0
Vector 1: X Y Z =  -0.015184 -0.048229  0.134774  Length =  0.14395
Vector 2: X Y Z =  -0.279887  0.009511  0.366470  Length =  0.46122
```

```
Angle between vectors =   36.77
# ro
Enter H,K,L Omega,Chi,Phi for 2 reflections:
 1 ? 1 1 0 3.433   33.490  -69.665
 2 ? 4 0 0 12.094   60.983  -31.630

Orienting     0.04760065 -0.00662789 -0.05122722
Matrix       0.02961753  0.08322406  0.00714444
(A* B* C*)    0.10075945 -0.02133199  0.04378954
Data stored in chon.pm4
# ca 2 0 0
Angles =    12.0262    6.0131   60.9075  -31.8900
# dr
# co 1 1
   29   8692
# sv
Step scan parameters
 Nstep     :   31 ?

 Stepwidths:   0.0000   0.1000   0.0000   0.0000 ? 0 0 0 0.1
 Time/step :   0.50 sec ?

Data stored in chon.pm4
# ss
Scan centre =    12.0260    6.0130   60.9075  -31.8900
Scan range  =     0.0000    0.0000    0.0000    3.0000  ( 31 steps, stepmode)
   42   8502 |                        :          o
   36   8734 |                           : o
   26   8516 |                    o    :
   40   8236 |                   :            o
   28   8478 +                      o  :
   22   8386 |                 o      :
   40   8674 |                         :      o
   40   8544 |                        :       o
   34   8470 |                        :   o
   24   8306 +                   o   :
   24   8330 |                   o   :
   30   8774 |                        o     :
   48   8376 |                      :                 o
   44   8504 |                        :            o
   26   8684 +                    o       :
   22   8318 |                 o     :
   42   8710 |                           :       o
   40   8628 |                         :       o
```

```
   50  8514 |                  :           o
   38  8654 +                    :   o
   28  8436 |                   o :
   40  8438 |                   :      o
   40  8568 |                    :     o
   36  8508 |                   :   o
   32  8374 +                  :  o
   24  8782 |                o       :
   32  8748 |                    o   :
   26  8520 |                 o   :
   22  8586 |               o      :
   42  8352 +                  :         o
   40  8462 |                   :      o
Centre at point  16.000     Angles =    12.0260    6.0130   60.9075  -31.8900
I(int)=    2.26  Sigma=50.10  FWHM=0.4465  Imax( 19)=    16.83  Temp=   0.00K
** Int. too low
# ca 0 2 0
Angles =    8.9800    4.4900  -14.3325  -94.5550
# dr
# ss
Scan centre =    8.9800    4.4900  -14.3325  -94.5550
Scan range  =    0.0000    0.0000    0.0000    3.0000  ( 31 steps, stepmode)
   52  8706 |                      :     o
   72  8540 |                    :                 o
   60  8338 |                   :              o
   66  8712 |                      :             o
   56  8672 +                     :        o
   50  8740 |                      :    o
   52  8542 |                    :       o
   48  8550 |                    :     o
   58  8530 |                    :          o
   64  8510 +                    :             o
   46  8640 |                     :  o
   64  8496 |                   :              o
   64  8578 |                     :            o
   56  8510 |                    :         o
   58  8716 +                      :        o
   56  8530 |                    :         o
   30  8530 |                 o     :
   68  8526 |                    :               o
   48  8578 |                     :    o
   44  8580 +                     :  o
   44  8124 |                 :        o
   56  8708 |                      :        o
```

```
   44   8360 |                        :       o
   42   8368 |                        :     o
   58   8264 +                        :               o
   50   8644 |                            :       o
   44   8346 |                        :       o
   64   8462 |                          :                   o
   58   8416 |                         :               o
   70   8480 +                          :                        o
   54   8424 |                         :            o
Centre at point  17.034      Angles =     8.9800    4.4900  -14.3325  -94.4516
I(int)=   -15.16  Sigma=66.63  FWHM=1.6359  Imax( 30)=    12.12  Temp=   0.00K

# ca 1 0 0
Angles =    6.0048    3.0024   60.9075  -31.8900
# ca 0 1 0
Angles =    4.4866    2.2433  -14.3325  -94.5550
# ca 0 0 1
Angles =    3.5282    1.7641   40.2525 -172.0600
# ca 0 0 2
Angles =    7.0598    3.5299   40.2525 -172.0600
# ca 0 2 0
Angles =    8.9800    4.4900  -14.3325  -94.5550
# ca 2 0 0
Angles =   12.0262    6.0131   60.9075  -31.8900
# ca 1 1 0
Angles =    7.4982    3.7491   33.4900  -70.0450
# ca 1 0 1
Angles =    7.7720    3.8860   75.6650  -95.6350
# ca 0 1 1
Angles =    5.7088    2.8544   11.8200 -122.6300
# ca 1 1 1
Angles =    8.9775    4.4887   45.6575  -94.8850
# ca  0 2 0
Angles =    8.9800    4.4900  -14.3325  -94.5550
# ch 40 140
# dr
# sv
Step scan parameters
 Nstep     :   31 ?

 Stepwidths:   0.0000   0.0000   0.0000   0.1000 ?

 Time/step :   0.50 sec ?
```

```
# ss
Scan centre =     8.9800    4.4900   40.0000  140.0000
Scan range  =     0.0000    0.0000    0.0000    3.0000  ( 31 steps, stepmode)
   76   8458 |            o         :
   62   8744 |          o                :
   52   8448 |        o             :
   74   8464 |            o          :
   74   8516 +            o           :
   54   8838 |         o                   :
   56   8702 |         o                 :
   96   8768 |                o           :
  114   8662 |                   o      :
  100   8636 +                 o         :
  132   8806 |                     o      :
  196   8608 |                      :          o
  234   8858 |                          :           o
  272   8764 |                        :                   o
  274   8872 +                          :                 o
  216   8638 |                      :            o
  304   8648 |                      :                          o
  232   8832 |                          :           o
  160   8498 |                    :     o
  102   8456 +                 o     :
   78   8838 |            o                :
   74   8546 |            o          :
   80   8458 |             o        :
   80   8898 |             o                :
   98   8542 +               o       :
   76   8586 |            o           :
   54   8742 |         o                 :
   80   8662 |             o            :
   86   8882 |              o              :
   92   8964 +              o              :
   76   8548 |            o          :
Centre at point  15.280      Angles =     8.9800    4.4900   40.0000  139.9280
I(int)=   150.34  Sigma=75.80  FWHM=0.6744  Imax( 17)=   231.02  Temp=   0.00K

# sv
Step scan parameters
 Nstep     :   31 ?

 Stepwidths:   0.0000   0.0000   0.0000   0.1000 ? 0 0 0.3 0
 Time/step :   0.50 sec ?
```

```
Data stored in chon.pm4
# ss
Scan centre =     8.9800    4.4900   40.0000  139.9300
Scan range  =     0.0000    0.0000    9.0000    0.0000  ( 31 steps, stepmode)
   126  15456 |                           o        :
   116  15270 |                         o        :
   140  15540 |                              o     :
   154  15486 |                                 o  :
   194  15094 +                             :             o
   174  15366 |                                :     o
   190  15366 |                                :         o
   222  15382 |                                :                o
   248  15364 |                                :                     o
   186  15416 +                                 :       o
   230  15286 |                               :                  o
   244  15178 |                              :                      o
   270  15250 |                               :                            o
   250  15040 |                             :                         o
   272  15170 +                              :                            o
   264  15188 |                              :                           o
   254  15106 |                             :                         o
   280  15144 |                              :                              o
   216  15092 |                             :                 o
   196  15048 +                             :             o
   204  15074 |                             :               o
   214  15076 |                             :                 o
   174  15152 |                              :       o
   126  14990 |                           o   :
   158  14966 +                            :      o
   174  15062 |                             :        o
   150  14972 |                            :    o
   110  15332 |                        o          :
   130  14972 |                            o  :
    84  14650 +                  o      :
    76  15116 |               o              :
Centre at point  15.594      Angles =     8.9800    4.4900   39.8782  139.9300
I(int)=   557.40  Sigma=103.55  FWHM=2.7550  Imax( 18)=   154.77  Temp=   0.00K

# sv
Step scan parameters
 Nstep     :   31 ?

 Stepwidths:   0.0000   0.0000   0.3000   0.0000 ? 0 0.05 0 0
 Time/step :   0.50 sec ?
```

```
Data stored in chon.pm4
# ss
Scan centre =     8.9800    4.4900   39.8775  139.9300
Scan range  =     0.0000    1.5000    0.0000    0.0000  ( 31 steps, stepmode)
   38  10550 |                         :  o
   38  10166 |                       :    o
   30   9890 |                     o  :
   28   9644 |                   o   :
   40   9880 +                      :      o
   32   9308 |                     :  o
   22   9456 |                o       :
   32   9448 |                      : o
   30   9202 |                     : o
   32   9168 +                     :  o
   48   9098 |                    :                 o
   28   9092 |                    :o
   42   8954 |                   :             o
   32   8922 |                   :    o
   32   8790 +                   :    o
   42   8722 |                  :             o
   44   8534 |                  :               o
   22   8788 |                o     :
   40   8824 |                   :           o
   26   8690 +                  :o
   50   8628 |                  :                   o
   18   8650 |             o       :
   46   8774 |                   :               o
   32   8716 |                  :    o
   34   8502 +                  :       o
   36   8764 |                   :       o
   40   8580 |                  :           o
   34   8616 |                  :      o
   44   8608 |                  :              o
   28   8584 +                  :  o
   56   8618 |                  :                        o
Centre at point  15.801      Angles =     8.9800     4.4801    39.8775   139.9300
I(int)=    -3.48  Sigma=53.04  FWHM=0.5181  Imax( 31)=    15.17  Temp=   0.00K

# sv
Step scan parameters
 Nstep     :   31 ?

 Stepwidths:   0.0000   0.0500   0.0000   0.0000 ? 0.1 0 0 0
```

Time/step :   0.50 sec ?

Data stored in chon.pm4

```
# ss
Scan centre =    8.9800    4.4801   39.8775  139.9300
Scan range  =    3.0000    0.0000    0.0000    0.0000  ( 31 steps, stepmode)
   44   8604 |    o                      :
   40   8734 |    o                         :
   50   8410 |     o                   :
   40   8740 |    o                         :
   44   8696 +    o                        :
   36   8754 |   o                          :
   44   8540 |    o                     :
   46   8718 |     o                       :
   36   8842 |   o                             :
   38   8618 +    o                       :
   32   8584 |   o                        :
   38   8636 |    o                       :
   50   8920 |     o                           :
   46   8558 |     o                    :
   30   8504 +  o                        :
   42   8658 |    o                       :
   36   8646 |   o                        :
   48   8656 |     o                      :
   58   8838 |      o                        :
  140   8580 +               o        :
  292   8848 |                             :      o
  356   8778 |                            :               o
  372   8698 |                          :                   o
  328   8754 |                          :            o
  414   8914 +                             :                    o
  398   8540 |                        :                       o
  408   8390 |                     :                            o
  412   8622 |                         :                         o
  364   8406 |                     :                        o
  236   8786 +                            : o
  128   8422 |               o       :
Centre at point  25.641      Angles =     9.9441    4.4801   39.8775  139.9300
I(int)=   329.44  Sigma=117.93  FWHM=0.9482  Imax( 25)=   370.40  Temp=   0.00K
** Unbalanced background
# ss
Scan centre =    9.9441    4.4801   39.8775  139.9300
Scan range  =    3.0000    0.0000    0.0000    0.0000  ( 31 steps, stepmode)
   36   8616 |   o                        :
```

```
   46   8670 |     o                       :
   40   8660 |     o                       :
   32   8454 |   o                     :
   48   8772 +      o                       :
   30   8664 |   o                        :
   36   8376 |    o                    :
   48   8838 |      o                       :
   78   8676 |          o                   :
  108   8716 +             o                :
  286   8546 |                           :         o
  344   8718 |                             :            o
  370   8662 |                            :                 o
  416   8682 |                            :                       o
  398   8534 +                           :                      o
  366   8716 |                             :               o
  404   8512 |                          :                       o
  430   8592 |                           :                          o
  346   8726 |                             :            o
  286   8632 +                            :       o
  158   8632 |                    o         :
   74   8754 |         o                    :
   46   8612 |     o                      :
   40   8630 |     o                      :
   42   8554 +     o                     :
   28   8754 |   o                         :
   30   8286 |   o                  :
   22   8750 |  o                          :
   28   8444 |   o                     :
   26   8496 +   o                      :
   26   8762 |   o                         :
Centre at point  15.742      Angles =     9.9183    4.4801   39.8775  139.9300
I(int)=   363.26  Sigma=56.88  FWHM=0.9725  Imax( 18)=   397.68  Temp=   0.00K

# sv
Step scan parameters
 Nstep     :   31 ?

 Stepwidths:   0.1000   0.0000   0.0000   0.0000 ? 0 0.05 0 0
 Time/step :   0.50 sec ?

Data stored in chon.pm4
# ss
Scan centre =     9.9183    4.4801   39.8775  139.9300
Scan range  =     0.0000    1.5000    0.0000    0.0000  ( 31 steps, stepmode)
```

```
   46   8620 |   o                   :
   62   8802 |     o                 :
   42   8878 |   o                   :
   48   8446 |    o                 :
   60   8566 +     o                 :
   66   8774 |     o                 :
   74   8580 |      o                :
   58   8468 |     o                :
  130   8488 |           o           :
  156   8826 +             o          :
  126   8698 |          o            :
  148   8700 |           o          :
  246   8606 |                    o  :
  278   8664 |                     : o
  284   8370 +                    :   o
  336   8744 |                     :      o
  322   8492 |                    :     o
  420   8432 |                    :             o
  418   8524 |                    :             o
  418   8446 +                    :             o
  466   8608 |                     :                 o
  446   8372 |                    :                o
  472   8496 |                    :                  o
  486   8630 |                     :                  o
  564   8590 +                     :                         o
  578   8684 |                     :                         o
  576   8808 |                     :                         o
  614   8576 |                     :                             o
  534   8548 |                     :                       o
  432  15296 +                              :      o
  354  15398 |                              :o
Centre at point  22.445      Angles =     9.9183    4.8023   39.8775  139.9300
I(int)=   383.02  Sigma=146.73  FWHM=0.2667  Imax( 28)=   562.40  Temp=   0.00K
** Unsuitable scanwidth
# ss
Scan centre =     9.9183    4.8023   39.8775  139.9300
Scan range  =     0.0000    1.5000    0.0000    0.0000  ( 31 steps, stepmode)
  148  15072 |            o            :
  136  15072 |           o             :
  122  15208 |         o                 :
  120  15002 |         o              :
  176  15036 +               o          :
  178  15218 |               o            :
  194  15080 |                 o        :
```

```
  256  15062 |                    o   :
  306  14892 |                     :    o
  314  15480 +                          o   :
  382  15062 |                       :        o
  338  14976 |                      :      o
  428  15278 |                         :          o
  432  14896 |                     :              o
  462  15342 +                         :            o
  438  14816 |                  :                 o
  446  15050 |                      :              o
  572  15080 |                      :                        o
  582  15256 |                         :                      o
  570  15006 +                      :                        o
  586  14862 |                    :                           o
  578  14990 |                      :                         o
  500  14982 |                      :                  o
  470  15120 |                        :               o
  304  15084 +                       :  o
  262  15120 |                      o   :
  216  15088 |                  o      :
  196  14806 |                o     :
  222  14836 |                  o   :
  190  14982 +                o        :
  242  14918 |                    o  :
Centre at point  17.439      Angles =     9.9183    4.8743   39.8775  139.9300
I(int)=   244.26  Sigma=117.26  FWHM=0.6065  Imax( 19)=   396.80  Temp=   0.00K

# sv
Step scan parameters
 Nstep     :   31 ?

 Stepwidths:   0.0000   0.0500   0.0000   0.0000 ? 0 0 0 0.1
 Time/step :   0.50 sec ?

Data stored in chon.pm4
# ss
Scan centre =     9.9183    4.8743   39.8775  139.9300
Scan range  =     0.0000    0.0000    0.0000    3.0000  ( 31 steps, stepmode)
   62  14338 |     o                         :
   82  14354 |       o                       :
   66  14216 |     o                         :
  112  14330 |         o                      :
   90  13788 +       o                       :
  152  14054 |           o                    :
```

```
  168  13784 |             o              :
  240  13548 |                  o         :
  324  13854 |                       o   :
  328  13326 +                       o  :
  466  13722 |                         :         o
  384  13118 |                        :   o
  472  13310 |                        :          o
  450  13002 |                        :        o
  500  13150 +                        :            o
  582  12816 |                        :                  o
  636  12398 |                       :                      o
  506  12358 |                       :             o
  360  12346 |                       :   o
  270  12086 +                  o     :
  226  11982 |                o        :
  190  11654 |              o          :
  268  11454 |                  o    :
  290  11522 |                   o  :
  256  11012 +                 o    :
  256  10574 |                 o   :
  268  10492 |                  o  :
  242   9944 |                o   :
  292   9948 |                  :  o
  274   9786 +                  :o
  324   9548 |                  :     o
Centre at point  17.875      Angles =     9.9183    4.8743   39.8775  140.1175
I(int)=   658.16  Sigma=122.14  FWHM=0.8783  Imax( 17)=   553.60  Temp=   0.00K
** Unbalanced background
# sv
Step scan parameters
 Nstep    :   31 ?

 Stepwidths:   0.0000   0.0000   0.0000   0.1000 ? 0 0 0.4 0
 Time/step :   0.50 sec ?

Data stored in chon.pm4
# ss
Scan centre =     9.9183    4.8743   39.8775  140.1200
Scan range  =     0.0000    0.0000   12.0000    0.0000  ( 31 steps, stepmode)
   50  14612 |     o                        :
   44  14864 |    o                         :
   60  14240 |     o                       :
   74  14620 |      o                       :
  128  14466 +            o                 :
```

```
   184  14342 |                    o              :
   208  14258 |                       o           :
   244  14368 |                           o       :
   286  14196 |                                o :
   354  14258 +                                   :    o
   408  14040 |                                   :          o
   466  13844 |                                  :                  o
   486  13998 |                                  :                    o
   480  13532 |                                  :                    o
   494  13544 +                                  :                     o
   518  13334 |                                 :                         o
   468  13376 |                                 :                   o
   512  13164 |                                 :                        o
   528  12870 |                                :                           o
   424  13000 +                                :               o
   388  12734 |                                :           o
   334  12336 |                               :      o
   240  12404 |                          o    :
   232  12042 |                         o    :
   160  12186 +                 o             :
   134  11524 |              o              :
    76  11386 |       o                    :
    56  11186 |    o                       :
    44  10904 |   o                       :
    32  10730 +  o                        :
    14   9986 | o                       :
Centre at point  15.886      Angles =     9.9183    4.8743   39.8319  140.1200
I(int)=  2533.68  Sigma=97.79  FWHM=5.3060  Imax( 19)=   473.29  Temp=   0.00K

# sv
Step scan parameters
 Nstep    :   31 ?

 Stepwidths:   0.0000   0.0000   0.4000   0.0000 ? 0 0 0 0.2
 Time/step :   0.50 sec ?

Data stored in chon.pm4
# ss
Scan centre =     9.9183    4.8743   39.8325  140.1200
Scan range  =     0.0000    0.0000    0.0000    6.0000  ( 31 steps, stepmode)
** Error while executing ./fcdscan.py 0.500000 31 0.000000 0.000000 0.000000 0.200000>NULL
# th 20 0 0 0
# dr
# th 9.9183    4.8743   39.8325  140.1200
```

```
# sv
Step scan parameters
 Nstep     :   31 ?

 Stepwidths:   0.0000   0.0000   0.0000   0.2000 ?

 Time/step :   0.50 sec ?

# ss
Scan centre =     9.9183    4.8743   39.8325  140.1200
Scan range  =     0.0000    0.0000    0.0000    6.0000  ( 31 steps, stepmode)
    28   9532 |  o                          :
    34   9604 |   o                         :
    48   9298 |    o                       :
    36   9334 |   o                        :
    26   9012 +  o                      :
    44   9242 |    o                      :
    60   8874 |    o                    :
    56   8886 |    o                    :
    72   8794 |      o                 :
   106   8660 +          o             :
   152   8828 |              o          :
   238   8550 |                     :o
   410   8868 |                        :            o
   428   8728 |                       :               o
   420   8760 +                       :              o
   560   8790 |                       :                          o
   436   8610 |                      :                 o
   212   8818 |                   o     :
   210   8668 |                   o    :
   280   8848 +                        : o
   248   8622 |                      :o
   278   8808 |                       :  o
   354   8558 |                     :          o
   330   8426 |                    :         o
   418   8870 +                        :             o
   292   8724 |                       :   o
   158   8814 |              o          :
    96   8662 |        o              :
    84   8724 |       o                :
    56   8592 +     o                 :
    42   8904 |    o                    :
Centre at point  18.000      Angles =     9.9183    4.8743   39.8325  140.5200
I(int)=   865.44  Sigma=79.43  FWHM=2.6639  Imax( 16)=   499.20  Temp=   0.00K
```

```
# xa 1
Enter HKL and Angles - Terminate with a blank line
Seq#  14 ? 1 1 1 9.9183    4.8743   39.8325  140.5200
Seq#  15 ?

  1 entries added to list 1   New n =   3
230 free entries left
# ca 0 2 0
Angles =     8.9800    4.4900  -14.3325  -94.5550
# ch 48.5 267
# dr
# sv
Step scan parameters
 Nstep     :   31 ?

 Stepwidths:   0.0000   0.0000   0.0000   0.2000 ? 0.1 0 0 0
 Time/step :   0.50 sec ?

Data stored in chon.pm4
# s
# ss
Scan centre =     8.9800    4.4900   48.5000  -93.0000
Scan range  =     3.0000    0.0000    0.0000    0.0000  ( 31 steps, stepmode)
   42   8714 |            o             :
   40   8744 |           o               :
   50   8582 |               o        :
   52   8692 |                o          :
   54   8764 +                o           :
   52   8770 |                o           :
   80   8800 |                         o    :
   82   8614 |                         :o
   84   8660 |                          :o
  104   8484 +                     :          o
  138   8788 |                           :              o
  112   8504 |                     :            o
  168   8542 |                      :                            o
  130   8654 |                        :             o
  146   8460 +                    :                        o
  156   8774 |                           :                     o
  132   8646 |                        :              o
   90   8576 |                      :    o
  114   8688 |                         :         o
   90   8690 +                        : o
```

```
   50   8596 |               o         :
   54   8588 |                o        :
   38   8666 |           o              :
   28   8500 |        o              :
   30   8886 +         o                     :
   34   8580 |          o              :
   34   8598 |          o              :
   26   8562 |       o                 :
   34   8580 |          o              :
   24   8518 +       o                :
   38   8608 |           o             :
Centre at point  14.223      Angles =     8.8023    4.4900   48.5000  -93.0000
I(int)=   108.46  Sigma=56.32  FWHM=0.7649  Imax( 13)=   126.71  Temp=   0.00K

# sv
Step scan parameters
 Nstep     :   31 ?

 Stepwidths:   0.1000   0.0000   0.0000   0.0000 ? 0 0.08 0 0
 Time/step :   0.50 sec ?

Data stored in chon.pm4
# ss
Scan centre =     8.8023    4.4900   48.5000  -93.0000
Scan range  =     0.0000    2.4000    0.0000    0.0000  ( 31 steps, stepmode)
   50   8596 |           o              :
   48   8522 |          o             :
   44   8664 |          o                 :
   40   8478 |         o              :
   50   8762 +           o                  :
   36   8452 |        o              :
   42   8432 |          o             :
   38   8532 |         o               :
   34   8464 |       o                :
   44   8594 +          o               :
   62   8656 |              o            :
   98   8700 |                      o      :
  108   8420 |                    :    o
  162   8546 |                       :              o
  180   8582 +                       :                  o
  216   8682 |                         :                        o
  172   8706 |                          :             o
  124   8748 |                           :o
  144   8526 |                       :           o
```

```
  146   8440 +                      :             o
  120   8668 |                           : o
  106   8514 |                       : o
  138   8738 |                            :    o
  106   8606 |                        o :
   92   8568 +                    o   :
  114   8526 |                       :    o
   80   8612 |                  o       :
  110   8556 |                        :  o
   64   8804 |              o             :
   44   8562 +         o               :
   54   8484 |           o           :
Centre at point  18.392      Angles =     8.8023    4.6813   48.5000  -93.0000
I(int)=    84.45  Sigma=67.45  FWHM=0.5353  Imax( 16)=   157.60  Temp=   0.00K

# sv
Step scan parameters
 Nstep    :   31 ?

 Stepwidths:   0.0000   0.0800   0.0000   0.0000 ? 0 0 0.4 0
 Time/step :   0.50 sec ?

Data stored in chon.pm4
# ss
Scan centre =     8.8023    4.6813   48.5000  -93.0000
Scan range  =     0.0000    0.0000   12.0000    0.0000  ( 31 steps, stepmode)
   50   8676 |                o    :
   50   8728 |                o     :
   60   8620 |                   :o
   56   8596 |                 o  :
   84   8522 +                   :         o
   72   8522 |                   :     o
  104   8590 |                    :                o
   94   8686 |                   :            o
   92   8522 |                   :           o
   72   8736 +                    :    o
  108  10356 |                       :              o
  106  15204 |                             :        o
  114  15434 |                             :           o
  128  15102 |                             :                o
  134  15418 +                             :                  o
  144  15362 |                             :                      o
  130  15286 |                             :                o
  146  15266 |                             :                       o
```

```
  110  15438 |                            :         o
  114  15236 +                            :           o
  132  15422 |                            :                 o
  120  15286 |                            :            o
  124  15486 |                            :              o
   92  15328 |                          :   o
   78  15434 +                        o   :
   80  15166 |                         o  :
   56  15258 |                  o         :
   44  15534 |              o             :
   62  15216 |                    o        :
   48  15376 +                o            :
   50  15406 |                 o           :
Centre at point  16.736      Angles =     8.8023    4.6813   48.7945  -93.0000
I(int)=   447.20  Sigma=71.41  FWHM=5.1704  Imax( 18)=    90.62  Temp=   0.00K

# sv
Step scan parameters
 Nstep     :   31 ?

 Stepwidths:   0.0000   0.0000   0.4000   0.0000 ? 0 0 0 0.2
 Time/step :   0.50 sec ?

Data stored in chon.pm4
# ss
Scan centre =     8.8023    4.6813   48.7950   -93.0000
Scan range  =     0.0000    0.0000    0.0000    6.0000  ( 31 steps, stepmode)
   36  14936 |          o               :
   56  14976 |               o           :
   36  15260 |          o                :
   46  14996 |            o              :
   36  15140 +          o                :
   36  14914 |          o               :
   32  14880 |        o                 :
   42  15120 |            o              :
   38  14742 |          o              :
   50  14824 +             o            :
   64  14874 |                 o         :
  104  14652 |                         :  o
  172  14522 |                         :                        o
  136  14638 |                         :            o
  138  14242 +                         :             o
  138  14786 |                         :            o
  118  14800 |                         :       o
```

```
  122  14478 |                        :         o
  132  14610 |                         :          o
   90  14640 +                       o  :
   96  14506 |                        : o
   64  14188 |                 o        :
   40  14234 |           o               :
   36  14194 |          o               :
   54  13926 +              o          :
   28  13732 |        o                :
   36  13560 |          o             :
   34  13450 |         o              :
   28  13298 |        o               :
   58  13130 +               o      :
   34  12882 |         o            :
Centre at point  16.265      Angles =     8.8023    4.6813   48.7950  -92.9471
I(int)=   178.00  Sigma=57.94  FWHM=1.5206  Imax( 13)=   131.54  Temp=   0.00K

# sv
Step scan parameters
 Nstep     :   31 ?

 Stepwidths:   0.0000   0.0000   0.0000   0.2000 ? 0 0 0 0.15
 Time/step :   0.50 sec ?

Data stored in chon.pm4
# ss
Scan centre =     8.8023    4.6813   48.7950  -92.9450
Scan range  =     0.0000    0.0000    0.0000    4.5000  ( 31 steps, stepmode)
   42   8616 |          o             :
   34   8924 |        o                    :
   46   8746 |          o             :
   50   8910 |           o               :
   40   8886 +         o                   :
   34   8770 |        o                 :
   40   8756 |         o              :
   64   8850 |               o           :
   80   8724 |                  o      :
  122   9010 +                          :  o
  154   8870 |                       :             o
  198   8854 |                       :                        o
  174   8926 |                        :                 o
  152   8788 |                       :            o
  104   8938 +                        o  :
  142   8726 |                       :           o
```

```
  180   8506 |                    :                         o
  138   8804 |                         :          o
   98   8718 |                      : o
  110   8974 +                          o  :
  108   8686 |                       :    o
   64   8834 |               o           :
   68   8618 |                 o      :
   52   8728 |            o            :
   50   8672 +            o            :
   44   8796 |          o              :
   44   8636 |          o            :
   32   8838 |        o                 :
   50   8898 |            o               :
   28   8614 +      o                :
   38   8876 |          o                 :
Centre at point  15.260      Angles =     8.8023    4.6813   48.7950  -93.0560
I(int)=   199.14  Sigma=58.56  FWHM=1.2809  Imax( 12)=   156.98  Temp=   0.00K

# xa 1
Enter HKL and Angles - Terminate with a blank line
Seq#  15 ? 0 2 0  8.8023    4.6813   48.7950  -93.0560
Seq#  16 ?

  1 entries added to list 1   New n =   4
229 free entries left
# x? 1

List 1    mode = 4    n =   4  (  0)

Seq# Stat    H      K      L    2Theta    Omega     Chi      Phi     Int.
 12   8    0.15   1.29   1.20    7.382    3.433   33.490  -69.665       0
 13   0    4.00   0.00   0.00   24.656   12.094   60.983  -31.630       0
 14   0    1.00   1.00   1.00    9.918    4.874   39.833  140.520       0
 15   0    0.00   2.00   0.00    8.802    4.681   48.795  -93.055       0

# av
2Theta, Omega, Chi, Phi for vector 1 ?  9.918    4.874   39.833  140.520
2Theta, Omega, Chi, Phi for vector 2 ?  8.802    4.681   48.795  -93.055
Vector 1: X Y Z =  -0.112612 -0.093130  0.121896  Length =  0.19030
Vector 2: X Y Z =  -0.005106  0.111168  0.127094  Length =  0.16893
Angle between vectors =   79.76
# ca 1 2 0
Angles =    10.8096    5.4048   16.2700  -80.0650
# ca 2 1 0
```

Angles = 12.8416 6.4208 47.0475 -58.1300
# ca 0 1 2
Angles = 8.3678 4.1839 24.3600 -138.2050
# ca 0 0 3
Angles = 10.5981 5.2990 40.2525 -172.0600
# ca 1 0 2
Angles = 10.4782 5.2391 69.5425 -141.3250
# ca 0 1 3
Angles = 11.5136 5.7568 29.8875 -146.8600
# av
2Theta, Omega, Chi, Phi for vector 1 ? 7.382 3.433 33.490 -69.665
2Theta, Omega, Chi, Phi for vector 2 ? 8.802 4.681 48.795 -93.055
Vector 1: X Y Z = 0.040473 0.111045 0.078198 Length = 0.14172
Vector 2: X Y Z = -0.005106 0.111168 0.127094 Length = 0.16893
Angle between vectors = 22.76
ah
H,K,L for vector 1 ? 1 1 0
H,K,L for vector 2 ? 0 2 0
Vector 1: X Y Z = 0.040973 0.112842 0.079427 Length = 0.14395
Vector 2: X Y Z = -0.013256 0.166448 -0.042664 Length = 0.17234
Angle between vectors = 53.23
# ca 3 0 0
Angles = 18.0811 9.0405 60.9075 -31.8900
# ca 0 3 0
Angles = 13.4874 6.7437 -14.3325 -94.5550
# ca 0 0 3
Angles = 10.5981 5.2990 40.2525 -172.0600
# av
2Theta, Omega, Chi, Phi for vector 1 ? 7.382 3.433 33.490 -69.665
2Theta, Omega, Chi, Phi for vector 2 ? 9.918 4.874 39.833 140.520
Vector 1: X Y Z = 0.040473 0.111045 0.078198 Length = 0.14172
Vector 2: X Y Z = -0.112612 -0.093130 0.121896 Length = 0.19030
Angle between vectors = 101.48
# ca 1 -1 0
Angles = 7.4982 3.7491 58.0125 44.6700
# ah
H,K,L for vector 1 ? 1 -1 0
H,K,L for vector 2 ? 0 2 2
Vector 1: X Y Z = 0.054229 -0.053607 0.122091 Length = 0.14395
Vector 2: X Y Z = -0.115710 0.180737 0.044915 Length = 0.21925
Angle between vectors = 109.39
# ca 0 2 2
Angles = 11.4318 5.7159 11.8200 -122.6300
# ca 1 1 2

Angles =   11.4033   5.7016   49.7825 -115.8100
ca 0 2 1
Angles =   9.6509   4.8255   0.3475 -110.3800
ah
H,K,L for vector 1 ? 0 2 1
H,K,L for vector 2 ? 0 0 2
Vector 1: X Y Z = -0.064483 0.173593 0.001126 Length = 0.18519
Vector 2: X Y Z = -0.102454 0.014289 0.087579 Length = 0.13554
Angle between vectors =   68.53
ah
H,K,L for vector 1 ? 0 2 1
H,K,L for vector 2 ? 1 1 0
Vector 1: X Y Z = -0.064483 0.173593 0.001126 Length = 0.18519
Vector 2: X Y Z =   0.040973 0.112842 0.079427 Length = 0.14395
Angle between vectors =   50.28
x?

List 1   mode = 4   n =   4 (0)

Seq#	Stat	H	K	L	2Theta	Omega	Chi	Phi	Int.
12	8	0.15	1.29	1.20	7.382	3.433	33.490	-69.665	0
13	0	4.00	0.00	0.00	24.656	12.094	60.983	-31.630	0
14	0	1.00	1.00	1.00	9.918	4.874	39.833	140.520	0
15	0	0.00	2.00	0.00	8.802	4.681	48.795	-93.055	0

List 2   mode = 1   n = 11 (0)

Seq#	Stat	H	K	L	2Theta	Omega	Chi	Phi	Int.
1	0	3.00	1.00	1.00	19.907	9.953	9.845	-13.290	0
2	0	4.00	0.00	0.00	24.187	12.094	0.000	0.000	0
3	0	0.00	4.00	0.00	18.016	9.008	0.000	-90.000	0
4	0	0.00	0.00	4.00	14.147	7.073	73.760	0.000	0
5	0	5.00	3.00	1.00	34.479	17.239	5.723	-23.465	0
6	0	5.00	2.00	0.00	31.726	15.863	0.000	-16.645	0
7	0	3.00	4.00	1.00	26.584	13.292	7.385	-43.370	0
8	0	2.00	7.00	3.00	36.868	18.434	16.285	-64.520	0
9	0	9.00	5.00	3.00	65.594	32.797	9.422	-21.485	0
10	0	9.00	6.00	7.00	76.188	38.094	19.593	-23.835	0
11	0	9.00	7.00	8.00	81.127	40.564	21.323	-26.890	0

229 free entries left
av
2Theta, Omega, Chi, Phi for vector 1 ? 7.382   3.433   33.490 -69.665
2Theta, Omega, Chi, Phi for vector 2 ? 9.918   4.874   39.833 140.520

Vector 1: X Y Z =   0.040473 0.111045 0.078198 Length = 0.14172
Vector 2: X Y Z = -0.112612 -0.093130 0.121896 Length = 0.19030
Angle between vectors = 101.48
av
2Theta, Omega, Chi, Phi for vector 1 ? 8.802   4.681   48.795 -93.055
2Theta, Omega, Chi, Phi for vector 2 ? 9.918   4.874   39.833 140.520
Vector 1: X Y Z = -0.005106 0.111168 0.127094 Length = 0.16893
Vector 2: X Y Z = -0.112612 -0.093130 0.121896 Length = 0.19030
Angle between vectors =   79.76
ca 1 1 0
Angles =   7.4982   3.7491   33.4900 -70.0450
ca 0 0 2
Angles =   7.0598   3.5299   40.2525 -172.0600
ca 0 2 1
Angles =   9.6509   4.8255   0.3475 -110.3800
ca 1 2 0
Angles =   10.8096   5.4048   16.2700 -80.0650
ca 1 0 2
Angles =   10.4782   5.2391   69.5425 -141.3250
ca 0 1 2
Angles =   8.3678   4.1839   24.3600 -138.2050
ca 1 1 1
Angles =   8.9775   4.4887   45.6575 -94.8850
ca 0 2 0
Angles =   8.9800   4.4900 -14.3325 -94.5550
ah
H,K,L for vector 1 ? 1 1 1
H,K,L for vector 2 ? 0 2 1
Vector 1: X Y Z = -0.010254 0.119986 0.123217 Length = 0.17229
Vector 2: X Y Z = -0.064483 0.173593 0.001126 Length = 0.18519
Angle between vectors =   47.32
ah
H,K,L for vector 1 ? -1 1 1
H,K,L for vector 2 ? 0 2 1
Vector 1: X Y Z = -0.105456 0.060751 -0.078302 Length = 0.14472
Vector 2: X Y Z = -0.064483 0.173593 0.001126 Length = 0.18519
Angle between vectors =   49.91
ah
H,K,L for vector 1 ? -1 -1 1
H,K,L for vector 2 ? 0 2 1
Vector 1: X Y Z = -0.092200 -0.105697 -0.035638 Length = 0.14472
Vector 2: X Y Z = -0.064483 0.173593 0.001126 Length = 0.18519
Angle between vectors = 117.67
ah

H,K,L for vector 1 ? 1 -1 1
H,K,L for vector 2 ? 0 2 1
Vector 1: X Y Z =   0.003001 -0.046462 0.165881 Length = 0.17229
Vector 2: X Y Z = -0.064483 0.173593 0.001126 Length = 0.18519
Angle between vectors = 104.66
ah
H,K,L for vector 1 ? 1 1 -1
H,K,L for vector 2 ? 0 2 1
Vector 1: X Y Z =   0.092200 0.105697 0.035638 Length = 0.14472
Vector 2: X Y Z = -0.064483 0.173593 0.001126 Length = 0.18519
Angle between vectors =   62.33
ah
H,K,L for vector 1 ? 1 -1 -1
H,K,L for vector 2 ? 0 2 1
Vector 1: X Y Z =   0.105456 -0.060751 0.078302 Length = 0.14472
Vector 2: X Y Z = -0.064483 0.173593 0.001126 Length = 0.18519
Angle between vectors = 130.09
ah
H,K,L for vector 1 ? -1 1 -1
H,K,L for vector 2 ? 0 2 1
Vector 1: X Y Z = -0.003001 0.046462 -0.165881 Length = 0.17229
Vector 2: X Y Z = -0.064483 0.173593 0.001126 Length = 0.18519
Angle between vectors =   75.34
ah
H,K,L for vector 1 ? -1 -1 -1
H,K,L for vector 2 ? 0 2 1
Vector 1: X Y Z =   0.010254 -0.119986 -0.123217 Length = 0.17229
Vector 2: X Y Z = -0.064483 0.173593 0.001126 Length = 0.18519
Angle between vectors = 132.68
ah
H,K,L for vector 1 ? 1 1 1
H,K,L for vector 2 ? 0 -2 1
Vector 1: X Y Z = -0.010254 0.119986 0.123217 Length = 0.17229
Vector 2: X Y Z = -0.037971 -0.159304 0.086454 Length = 0.18519
Angle between vectors = 104.66
ah
H,K,L for vector 1 ? -1 1 1
H,K,L for vector 2 ? 0 -2 1
Vector 1: X Y Z = -0.105456 0.060751 -0.078302 Length = 0.14472
Vector 2: X Y Z = -0.037971 -0.159304 0.086454 Length = 0.18519
Angle between vectors = 117.67
ah
H,K,L for vector 1 ? 1 -1 1
H,K,L for vector 2 ? 0 -2 1

Vector 1: X Y Z = 0.003001 -0.046462 0.165881 Length = 0.17229
Vector 2: X Y Z = -0.037971 -0.159304 0.086454 Length = 0.18519
Angle between vectors = 47.32
ah
H,K,L for vector 1 ? 1 1 -1
H,K,L for vector 2 ? 0 -2 1
Vector 1: X Y Z = 0.092200 0.105697 0.035638 Length = 0.14472
Vector 2: X Y Z = -0.037971 -0.159304 0.086454 Length = 0.18519
Angle between vectors = 130.09
ah
H,K,L for vector 1 ? -1 -1 1
H,K,L for vector 2 ? 0 -2 1
Vector 1: X Y Z = -0.092200 -0.105697 -0.035638 Length = 0.14472
Vector 2: X Y Z = -0.037971 -0.159304 0.086454 Length = 0.18519
Angle between vectors = 49.91
ah
H,K,L for vector 1 ? -1 1 -1
H,K,L for vector 2 ? 0 -2 1
Vector 1: X Y Z = -0.003001 0.046462 -0.165881 Length = 0.17229
Vector 2: X Y Z = -0.037971 -0.159304 0.086454 Length = 0.18519
Angle between vectors = 132.68
ah
H,K,L for vector 1 ? 1 -1 -1
H,K,L for vector 2 ? 0 -2 1
Vector 1: X Y Z = 0.105456 -0.060751 0.078302 Length = 0.14472
Vector 2: X Y Z = -0.037971 -0.159304 0.086454 Length = 0.18519
Angle between vectors = 62.33
ah
H,K,L for vector 1 ? -1 -1 -1
H,K,L for vector 2 ? 0 -2 1
Vector 1: X Y Z = 0.010254 -0.119986 -0.123217 Length = 0.17229
Vector 2: X Y Z = -0.037971 -0.159304 0.086454 Length = 0.18519
Angle between vectors = 75.34
ah
H,K,L for vector 1 ? 1 1 1
H,K,L for vector 2 ? 0 2 -1
Vector 1: X Y Z = -0.010254 0.119986 0.123217 Length = 0.17229
Vector 2: X Y Z = 0.037971 0.159304 -0.086454 Length = 0.18519
Angle between vectors = 75.34
ah
H,K,L for vector 1 ? 0 2 0
H,K,L for vector 2 ? 0 2 1
Vector 1: X Y Z = -0.013256 0.166448 -0.042664 Length = 0.17234
Vector 2: X Y Z = -0.064483 0.173593 0.001126 Length = 0.18519

```
Angle between vectors =   21.47
# ca 1 1 1
Angles =     8.9775    4.4887   45.6575  -94.8850
# ch 50 97
# dr
# sv
Step scan parameters
 Nstep    :  31 ?

 Stepwidths:  0.0000   0.0000   0.0000   0.1500 ? 0.2 0 0 0
 Time/step :  0.50 sec ?

Data stored in chon.pm4
# ss
Scan centre =    8.9775    4.4885   50.0000   97.0000
Scan range  =    6.0000    0.0000    0.0000    0.0000  ( 31 steps, stepmode)
   56  11392 |                               o  :
   66  11040 |                               :     o
   60  10880 |                               : o
   62  10472 |                             :    o
   48  10096 +                          o    :
   46  10138 |                         o    :
   48   9810 |                         o  :
   62   9642 |                         :       o
   42   9522 |                      o    :
   38   9312 +                   o       :
   50   9114 |                        : o
   46   9026 |                       :o
   90   8954 |                       :                         o
  102   9094 |                         :                               o
   98   8990 +                        :                             o
   96   9084 |                       :                            o
   68   8680 |                      :              o
   38   8566 |                   o     :
   32   8822 |                 o        :
   32   8826 +                 o        :
   28   8826 |              o           :
   28   8700 |              o          :
   30   8424 |                o       :
   32   8886 |                 o        :
   32   8876 +                 o        :
   22   8684 |           o             :
   10   8728 |    o                    :
   30   8898 |                o         :
```

```
   28   8622 |        o      :
   32   8742 +          o    :
   14   8562 |    o          :
Centre at point  16.000      Angles =     8.9775    4.4885   50.0000   97.0000
I(int)=    41.48  Sigma=55.93  FWHM=0.8661  Imax( 14)=    58.66  Temp=   0.00K
** Int. too low
# sv
Step scan parameters
 Nstep    :   31 ?

 Stepwidths:   0.2000   0.0000   0.0000   0.0000 ? 0.1 0 0 0
 Time/step :   0.50 sec ?

Data stored in chon.pm4
# s
# ss
Scan centre =     8.9775    4.4885   50.0000   97.0000
Scan range  =     3.0000    0.0000    0.0000    0.0000  ( 31 steps, stepmode)
   60   8692 |                :  o
   58   8804 |                 :o
   62   8728 |                :   o
   36   8680 |          o      :
   48   8386 +             : o
   42   8358 |            o  :
   42   8892 |            o      :
   40   8686 |           o     :
   66   8326 |             :       o
   66   8530 +               :    o
  106   8682 |                :               o
   86   8568 |               :          o
   96   8636 |               :             o
   70   8312 |             :        o
   86   8624 +               :          o
   94   8584 |               :            o
   84   8520 |               :          o
   60   8806 |                 : o
   62   8674 |                :   o
   46   8544 +             o   :
   32   8518 |         o      :
   36   8570 |          o      :
   28   8430 |        o      :
   34   8802 |         o        :
   34   8912 +          o         :
   30   8598 |         o        :
```

```
   26   8804 |            o               :
   38   8622 |                  o       :
   32   8610 |              o          :
   32   8682 +              o           :
   20   8626 |        o                :
Centre at point  16.000     Angles =    8.9775    4.4885   50.0000   97.0000
I(int)=    37.48  Sigma=56.26  FWHM=0.3399  Imax( 11)=    60.34  Temp=   0.00K
** Int. too low
# x? 1

List 1    mode = 4    n =   4  (  0)

Seq# Stat    H      K      L    2Theta    Omega     Chi      Phi     Int.
 12   8    0.15   1.29   1.20    7.382    3.433   33.490  -69.665       0
 13   0    4.00   0.00   0.00   24.656   12.094   60.983  -31.630       0
 14   0    1.00   1.00   1.00    9.918    4.874   39.833  140.520       0
 15   0    0.00   2.00   0.00    8.802    4.681   48.795  -93.055       0

# av
2Theta, Omega, Chi, Phi for vector 1 ? 7.382    3.433   33.490  -69.665
2Theta, Omega, Chi, Phi for vector 2 ? 8.802    4.681   48.795  -93.055
Vector 1: X Y Z =   0.040473  0.111045  0.078198  Length =  0.14172
Vector 2: X Y Z =  -0.005106  0.111168  0.127094  Length =  0.16893
Angle between vectors =   22.76
# ah
H,K,L for vector 1 ? 0 0 2
H,K,L for vector 2 ? 0 2 1
Vector 1: X Y Z =  -0.102454  0.014289  0.087579  Length =  0.13554
Vector 2: X Y Z =  -0.064483  0.173593  0.001126  Length =  0.18519
Angle between vectors =   68.53
# ah
H,K,L for vector 1 ? 0 0 2
H,K,L for vector 2 ? 0 -2 1
Vector 1: X Y Z =  -0.102454  0.014289  0.087579  Length =  0.13554
Vector 2: X Y Z =  -0.037971 -0.159304  0.086454  Length =  0.18519
Angle between vectors =   68.53
# ah
H,K,L for vector 1 ? 1 1 0
H,K,L for vector 2 ? 0 2 1
Vector 1: X Y Z =   0.040973  0.112842  0.079427  Length =  0.14395
Vector 2: X Y Z =  -0.064483  0.173593  0.001126  Length =  0.18519
Angle between vectors =   50.28
# ah
H,K,L for vector 1 ? 1 -1 0
```

```
H,K,L for vector 2 ? 0 2 1
Vector 1: X Y Z =   0.054229 -0.053607  0.122091  Length =  0.14395
Vector 2: X Y Z =  -0.064483  0.173593  0.001126  Length =  0.18519
Angle between vectors =  118.37
# ah
H,K,L for vector 1 ? -1 1 0
H,K,L for vector 2 ? 0 2 1
Vector 1: X Y Z =  -0.054229  0.053607 -0.122091  Length =  0.14395
Vector 2: X Y Z =  -0.064483  0.173593  0.001126  Length =  0.18519
Angle between vectors =   61.63
# ah
H,K,L for vector 1 ? 1 1 0
H,K,L for vector 2 ? 0 -2 1
Vector 1: X Y Z =   0.040973  0.112842  0.079427  Length =  0.14395
Vector 2: X Y Z =  -0.037971 -0.159304  0.086454  Length =  0.18519
Angle between vectors =  118.37
# ca 0 2 1
Angles =    9.6509    4.8255    0.3475 -110.3800
# ah
H,K,L for vector 1 ? 1 1 0
H,K,L for vector 2 ? 1 1 1
Vector 1: X Y Z =   0.040973  0.112842  0.079427  Length =  0.14395
Vector 2: X Y Z =  -0.010254  0.119986  0.123217  Length =  0.17229
Angle between vectors =   22.54
# x?

List 1    mode = 4    n =   4  (  0)

Seq# Stat    H      K      L    2Theta    Omega     Chi      Phi     Int.
 12   8    0.15   1.29   1.20    7.382     3.433   33.490  -69.665       0
 13   0    4.00   0.00   0.00   24.656    12.094   60.983  -31.630       0
 14   0    1.00   1.00   1.00    9.918     4.874   39.833  140.520       0
 15   0    0.00   2.00   0.00    8.802     4.681   48.795  -93.055       0

List 2    mode = 1    n =  11  (  0)

Seq# Stat    H      K      L    2Theta    Omega     Chi      Phi     Int.
  1   0    3.00   1.00   1.00   19.907     9.953    9.845  -13.290       0
  2   0    4.00   0.00   0.00   24.187    12.094    0.000    0.000       0
  3   0    0.00   4.00   0.00   18.016     9.008    0.000  -90.000       0
  4   0    0.00   0.00   4.00   14.147     7.073   73.760    0.000       0
  5   0    5.00   3.00   1.00   34.479    17.239    5.723  -23.465       0
  6   0    5.00   2.00   0.00   31.726    15.863    0.000  -16.645       0
  7   0    3.00   4.00   1.00   26.584    13.292    7.385  -43.370       0
```

```
 8   0    2.00   7.00   3.00   36.868   18.434   16.285  -64.520       0
 9   0    9.00   5.00   3.00   65.594   32.797    9.422  -21.485       0
10   0    9.00   6.00   7.00   76.188   38.094   19.593  -23.835       0
11   0    9.00   7.00   8.00   81.127   40.564   21.323  -26.890       0

229 free entries left
# ro
Enter H,K,L Omega,Chi,Phi for 2 reflections:
 1 ? 1 1 0  3.433   33.490  -69.665
 2 ? 1 1 1 4.681   48.795  -93.055

Orienting     0.05707884 -0.01610609 -0.04582913
Matrix        0.02976224  0.08307935  0.00087123
(A* B* C*)    0.09566453 -0.01623706  0.04991720
Data stored in chon.pm4
# ca 2 0 0
Angles =    12.0262    6.0131   56.0625  -27.5400
# dr
# co 1 1
   17  15321
# ca 4 0 0
Angles =    24.1874   12.0937   56.0625  -27.5400
# dr
# co 5 1
   10  14975     11  14831     9  14798     10  14789     9  14745
Average Intensity =       9.800 cps, standard deviation =     0.748 cps
Average Monitor   =   14827.600 cps, standard deviation =    78.648 cps
# ca 1 0 4
Angles =    16.8687    8.4344   66.1525 -165.2450
# dr
# co 5 1
   11   8780     8   8778     11   8689     12   8681     7   8650
Average Intensity =       9.800 cps, standard deviation =     1.939 cps
Average Monitor   =    8715.600 cps, standard deviation =    53.384 cps
# sv
Step scan parameters
 Nstep     :   31 ?

 Stepwidths:   0.1000   0.0000   0.0000   0.0000 ?

 Time/step :   0.50 sec ?

# ss
Scan centre =    16.8685    8.4343   66.1525 -165.2450
```

```
Scan range =     3.0000    0.0000    0.0000    0.0000  ( 31 steps, stepmode)
   16   8574 |            o        :
   16   8646 |            o         :
   16   8764 |            o         :
   18   8456 |              o      :
   16   8630 +            o        :
    6   8652 |    o                :
    2   8516 | o                  :
   14   8670 |          o           :
    4   8506 |  o                 :
   18   8602 +              o      :
    6   8580 |    o               :
   12  15144 |        o                   :
    6  14948 |    o                      :
   14  15186 |          o                 :
   14  14824 +          o                :
   12  15132 |        o                  :
   10  15468 |      o                    :
    8  15032 |     o                     :
   12  15002 |        o                  :
   22  14934 +                o         :
    6  15008 |    o                      :
   22  15268 |                o          :
    2  15140 | o                         :
    4  15148 |  o                        :
   10  15410 +      o                    :
   12  15138 |        o                  :
   10  15106 |      o                    :
   12  15184 |        o                  :
    8  15040 |     o                     :
   12  14866 +        o                 :
   10  14966 |      o                    :
Centre at point  11.592      Angles =    16.4277    8.4343   66.1525 -165.2450
I(int)=    -6.54  Sigma=31.49  FWHM=0.8696  Imax( 22)=     9.98  Temp=   0.00K
** Unsuitable scanwidth
# x? 1

List 1    mode = 4    n =   4  (  0)
```

Seq#	Stat	H	K	L	2Theta	Omega	Chi	Phi	Int.
12	8	0.15	1.29	1.20	7.382	3.433	33.490	-69.665	0
13	0	4.00	0.00	0.00	24.656	12.094	60.983	-31.630	0
14	0	1.00	1.00	1.00	9.918	4.874	39.833	140.520	0
15	0	0.00	2.00	0.00	8.802	4.681	48.795	-93.055	0

```
# ca 0 2 1
Angles =    9.6509    4.8255    5.4050 -115.0450
# ah
H,K,L for vector 1 ? 1 -1 0
H,K,L for vector 2 ? 1 1 1
Vector 1: X Y Z =   0.073185 -0.053317  0.111902  Length =  0.14395
Vector 2: X Y Z =  -0.004856  0.113713  0.129345  Length =  0.17229
Angle between vectors =   71.05
# ah
H,K,L for vector 1 ? 1 -1 0
H,K,L for vector 2 ? 1 -1 1
Vector 1: X Y Z =   0.073185 -0.053317  0.111902  Length =  0.14395
Vector 2: X Y Z =   0.027356 -0.052446  0.161819  Length =  0.17229
Angle between vectors =   22.54
# ah
H,K,L for vector 1 ?

# ro
Enter H,K,L Omega,Chi,Phi for 2 reflections:
 1 ?

# x? 1

List 1    mode = 4    n =   4  (  0)

Seq# Stat    H      K      L    2Theta    Omega     Chi      Phi     Int.
 12   8    0.15   1.29   1.20    7.382    3.433   33.490  -69.665       0
 13   0    4.00   0.00   0.00   24.656   12.094   60.983  -31.630       0
 14   0    1.00   1.00   1.00    9.918    4.874   39.833  140.520       0
 15   0    0.00   2.00   0.00    8.802    4.681   48.795  -93.055       0

# ro
Enter H,K,L Omega,Chi,Phi for 2 reflections:
 1 ? 1 -1 0 7.382    3.433   33.490  -69.665
 2 ?

# ro
Enter H,K,L Omega,Chi,Phi for 2 reflections:
 1 ? 1 -1 0 3.433   33.490  -69.665
 2 ? 1 -1 1 4.681   48.795  -93.055

Orienting    -0.02250430 -0.06347706 -0.04582913
Matrix        0.11108766 -0.00175393  0.00087123
```

(A* B* C*)   0.02117960 -0.05824786 0.04991720
Data stored in chon.pm4
ca 0 2 1
Angles =   9.6509   4.8255 -21.0700 179.1250
ah
H,K,L for vector 1 ? -1 1
H,K,L for vector 2 ? -1 1 1
Vector 1: X Y Z = -0.040973 -0.112842 -0.079427 Length = 0.14395
Vector 2: X Y Z = -0.086802 -0.111970 -0.029510 Length = 0.14472
Angle between vectors =   27.15
ah
H,K,L for vector 1 ? -1 1 0
H,K,L for vector 2 ? -1 1 1
Vector 1: X Y Z = -0.040973 -0.112842 -0.079427 Length = 0.14395
Vector 2: X Y Z = -0.086802 -0.111970 -0.029510 Length = 0.14472
Angle between vectors =   27.15
ah
H,K,L for vector 1 ? 1 1 0
H,K,L for vector 2 ? 1 1 -1
Vector 1: X Y Z = -0.085981 0.109334 -0.037068 Length = 0.14395
Vector 2: X Y Z = -0.040152 0.108462 -0.086985 Length = 0.14472
Angle between vectors =   27.15
exit

STOP - DIF4 13-May-17 22:54

27-May-17 02:28 Protocol ON
pp
    9.9180   4.9590   53.0000 209.0000
X?

List 1   mode = 4   n =   4 (0)

Seq#	Stat	H	K	L	2Theta	Omega	Chi	Phi	Int.
12	8	0.15	1.29	1.20	7.382	3.433	33.490	-69.665	0
13	0	4.00	0.00	0.00	24.656	12.094	60.982	-31.630	0
14	0	1.00	1.00	1.00	9.918	4.874	39.833	140.520	0
15	0	0.00	2.00	0.00	8.802	4.681	48.795	-93.055	0

List 2   mode = 1   n = 11 (0)

Seq#	Stat	H	K	L	2Theta	Omega	Chi	Phi	Int.
1	0	3.00	1.00	1.00	19.907	9.953	9.845	-13.290	0
2	0	4.00	0.00	0.00	24.187	12.094	0.000	0.000	0

3	0	0.00	4.00	0.00	18.016	9.008	0.000	-90.000	0
4	0	0.00	0.00	4.00	14.146	7.073	73.760	0.000	0
5	0	5.00	3.00	1.00	34.479	17.239	5.722	-23.465	0
6	0	5.00	2.00	0.00	31.726	15.863	0.000	-16.645	0
7	0	3.00	4.00	1.00	26.583	13.292	7.385	-43.370	0
8	0	2.00	7.00	3.00	36.868	18.434	16.285	-64.520	0
9	0	9.00	5.00	3.00	65.594	32.797	9.422	-21.485	0
10	0	9.00	6.00	7.00	76.188	38.094	19.593	-23.835	0
11	0	9.00	7.00	8.00	81.127	40.564	21.323	-26.890	0

229 free entries left
# ca 0 0 1
Angles = 3.5282 1.7641 47.4400 -178.9100
# ca 1 0 0
Angles = 6.0048 3.0024 10.5850 -101.4500
# ca 0 1 0
Angles = 4.4866 2.2433 -42.5300 178.4150
# ca 0 0 2
Angles = 7.0598 3.5299 47.4400 -178.9100
# ca 0 2 0
Angles = 8.9800 4.4900 -42.5300 178.4150
# ca 2 0 0
Angles = 12.0262 6.0131 10.5850 -101.4500
# ca 1 1 1
Angles = 8.9775 4.4887 4.2775 -140.1000
# ca 0 1 2
Angles = 8.3678 4.1839 15.0075 179.9950
# ca 0 1 1
Angles = 5.7088 2.8544 -4.3575 179.5350
# ca 1 1 0
Angles = 7.4982 3.7491 -14.9225 -128.1800
# ca 1 0 1
Angles = 7.7720 3.8860 28.4600 -121.4000
# ex

STOP - DIF4 27-May-17 10:31

10-Aug-17 12:11 Protocol ON
pp
-0.0000 0.0000 -0.0000 0.0000
# ca 1 1 1
Angles = 8.9775 4.4887 4.2775 -140.1000
# dr
# ca 5 3 1

Angles = 34.4788 17.2394 -1.6625 -122.3300
# dr
# ca 5 0 0
Angles = 30.3643 15.1822 10.5850 -101.4500
# dr
#

Table S16. PL strength of compound **4** in MeOH from 223 K to 323 K

Wave Length (nm)	-50 °C	-40 °C	-30°C	-20°C	-10°C	0°C	10°C	20°C	30°C	40°C
380	397130	393370	368340	344950	319830	281870	353000	421790	399930	366190
381	421960	414460	390720	362190	338380	299170	379420	456980	432380	398960
382	454540	441530	420700	386090	357250	317310	413380	499490	471630	432900
383	487410	474740	443960	411870	382940	338980	446610	545190	513390	473420
384	519770	508410	473680	440830	407640	361450	481320	589230	554240	507000
385	557330	546640	510630	469200	432840	387070	518060	640160	602990	548890
386	595240	583500	543580	500040	463360	411260	554060	687270	640390	586390
387	635130	615650	576330	525350	482320	439100	587200	726980	684870	623520
388	665030	651300	604550	552810	511900	458650	626770	769700	722400	655060
389	701070	685110	636590	577050	533220	480420	653130	808920	759720	687080
390	731880	713260	663510	606760	556630	504310	688390	847540	796170	729310
391	761190	739850	688770	629630	579910	519120	713480	890250	835940	760560
392	791100	773080	709360	653500	601950	538490	742430	920030	864840	790140
393	820650	790290	736000	672590	621190	559380	767720	955710	900080	817230
394	851120	817170	764800	698620	638350	580670	804510	995180	934720	856770
395	867460	840920	783070	714350	660120	597600	826980	1039680	976210	882250
396	896870	864430	813880	735110	680650	619630	861970	1066390	1004330	922610
397	921230	896420	835240	761270	695570	638370	898050	1111760	1045980	960990
398	950110	924460	857910	783950	720010	659050	919320	1151680	1088390	993440
399	974950	947340	883190	805690	744450	680180	954260	1200750	1129320	1035630
400	1002690	977470	906300	828320	768180	701400	984050	1232160	1160510	1066670
401	1026480	999870	934960	863420	795010	725950	1020990	1278470	1212660	1109000
402	1058320	1029100	960520	878760	820100	750310	1057160	1333870	1260380	1148260
403	1090790	1062140	988450	913630	844150	773670	1092540	1382930	1296590	1196730
404	1119950	1089840	1017160	946850	871690	795620	1143440	1429410	1346030	1230520
405	1148000	1120430	1054520	964100	893950	820680	1167420	1473230	1382490	1273230
406	1176980	1147640	1077260	985940	923280	843880	1205650	1515350	1425670	1311250
407	1203940	1176400	1106800	1016820	943550	867110	1235250	1561970	1469770	1343080
408	1234710	1208080	1129490	1037700	968870	891990	1274990	1606000	1520240	1391000
409	1258740	1227620	1155060	1064580	984350	912400	1301390	1637270	1550440	1426610
410	1281310	1245500	1175760	1086990	1008280	925160	1330680	1675730	1581200	1450710
411	1296810	1270500	1194570	1102410	1026700	939710	1356520	1704450	1603200	1481450
412	1322650	1289070	1207570	1121000	1042920	962100	1383280	1728400	1635790	1495690
413	1334730	1302320	1227140	1128390	1047080	970440	1396620	1752570	1657670	1522050
414	1339690	1316440	1232420	1140420	1057730	985910	1419220	1778330	1679810	1547890
415	1353450	1328260	1242130	1147090	1065190	987560	1424430	1799670	1702900	1566710
416	1358620	1328620	1251150	1161310	1081810	1005460	1438500	1812460	1713370	1573830
417	1367100	1329290	1248380	1162420	1084550	1002180	1458290	1831630	1728950	1590140
418	1363840	1337830	1261150	1171460	1090670	1009940	1470000	1847340	1752370	1607920
419	1368550	1339130	1263070	1178450	1095120	1011840	1470740	1847020	1757510	1621420
420	1371040	1340090	1266150	1174690	1098050	1017420	1479280	1858270	1764140	1625170
421	1374110	1341680	1269640	1175740	1095250	1022420	1486450	1873280	1770090	1623620
422	1369630	1339140	1264320	1181210	1096800	1020150	1488750	1881990	1781740	1638250
423	1371870	1340300	1271140	1180460	1098700	1020670	1490550	1878690	1775580	1640620
424	1369740	1342780	1273250	1171540	1104640	1023310	1493320	1881880	1786670	1648400
425	1368570	1335600	1265390	1183060	1100600	1020740	1494290	1886950	1788120	1654740
426	1362800	1330850	1267650	1173930	1102960	1027030	1504610	1887070	1791050	1652560
427	1360490	1329960	1268180	1180460	1095750	1031450	1499300	1891830	1798420	1653080
428	1359390	1338430	1267580	1176940	1103060	1031120	1500180	1890590	1791880	1657590
429	1360280	1325760	1264340	1178940	1099400	1029740	1494010	1897770	1800240	1666910
430	1356020	1329460	1261810	1175820	1099070	1033310	1511290	1895030	1794340	1655630
431	1349760	1321430	1261010	1170320	1100000	1022890	1499990	1886640	1795140	1658190
432	1345320	1326830	1251150	1167030	1102280	1021880	1491000	1891170	1794630	1660040
433	1345110	1310980	1248320	1167290	1089910	1019900	1500390	1886040	1794090	1654610
434	1335710	1313920	1245030	1162930	1085920	1024130	1491660	1875670	1789440	1645050
435	1332170	1305230	1240510	1150700	1082360	1014590	1483350	1878150	1778100	1643510
436	1323890	1298870	1231710	1150680	1077720	1013350	1478850	1869940	1763250	1642420
437	1309880	1292690	1228940	1148470	1074350	1001630	1477450	1851420	1751970	1627950
438	1313440	1280520	1219810	1130630	1066840	998550	1458920	1843970	1748490	1616530
439	1296400	1275240	1212880	1128230	1060220	991950	1452980	1825130	1740600	1604110
440	1282790	1260700	1200350	1122420	1056640	981700	1447550	1814720	1722850	1593610
441	1277400	1247240	1192420	1106950	1040230	976570	1431230	1800790	1709810	1581860

442	1261580	1243070	1179160	1104860	1033420	970770	1418770	1787990	1697120	1573560
443	1254810	1227120	1174330	1094610	1022690	960580	1406940	1773980	1685330	1560860
444	1242170	1215260	1159360	1081060	1016440	948210	1394330	1764780	1662280	1542590
445	1224490	1205500	1146320	1068390	1003640	941620	1382240	1748060	1656610	1527750
446	1209360	1191290	1135480	1053440	992180	932670	1363950	1725010	1637500	1505740
447	1200180	1174820	1125530	1051090	987590	918120	1357990	1704730	1620020	1490020
448	1189670	1164470	1109170	1044460	974090	911510	1341120	1687560	1604980	1478990
449	1174500	1151160	1102970	1032290	967860	900230	1325160	1668110	1581640	1458790
450	1159810	1137570	1090590	1014660	949690	899920	1310350	1654240	1565620	1444260
451	1157340	1125300	1079450	1003470	945950	878600	1290210	1634490	1558400	1425940
452	1139190	1113510	1055020	1000130	926230	871450	1278170	1615850	1527680	1415730
453	1124990	1106080	1047710	982970	922230	864980	1258200	1586910	1518410	1392140
454	1111820	1086370	1036150	972320	904900	852750	1246920	1574460	1496200	1389310
455	1098710	1075320	1024520	958670	895680	838510	1235070	1555110	1474490	1361180
456	1090420	1066270	1012070	941880	888260	826510	1225770	1526270	1459990	1336840
457	1063740	1051470	987310	933820	877790	820030	1204570	1509350	1445030	1329180
458	1065770	1036990	985500	924150	862850	810030	1190730	1492980	1422360	1307640
459	1040530	1028590	972930	907480	853710	795690	1170070	1476440	1400690	1295420
460	1030910	1011300	967170	903890	846740	787100	1160850	1452830	1389760	1276340
461	1015260	1001470	951170	886070	832410	780210	1147360	1442460	1362720	1256210
462	1008090	987990	940970	880010	824580	767930	1120160	1419570	1348460	1240190
463	991000	972710	921500	867280	812950	759850	1110980	1389830	1318340	1228150
464	982700	965170	909680	850820	798770	752530	1095430	1375460	1312890	1210570
465	967970	950980	905390	842570	788200	741790	1083550	1361600	1291880	1186420
466	959710	931930	886080	829440	776000	721310	1070030	1345040	1269750	1171660
467	940620	922340	875410	817420	764360	716050	1046150	1320860	1247910	1155270
468	927320	908150	861430	808370	753660	703810	1028970	1294300	1227420	1132280
469	915300	894870	850910	793230	738080	691430	1013680	1275590	1206000	1110160
470	897790	878320	836970	776070	729290	680560	993210	1249920	1181920	1086650
471	879670	862110	813960	763790	712820	666560	972710	1220730	1162090	1067080
472	862420	840570	805810	750250	699410	652150	958050	1199760	1137470	1047180
473	844700	828650	787580	737610	689160	640870	942810	1175980	1123650	1023800
474	832950	814460	774430	723730	674000	626170	918950	1152890	1101700	1005690
475	816570	798320	763900	706580	662350	614060	899900	1136470	1074810	987410
476	799280	782470	743670	693670	646690	602170	889200	1116070	1053800	969300
477	790830	772790	731030	684770	638560	596280	872490	1101170	1030110	949450
478	776190	758170	719660	669490	626310	583240	856930	1073890	1010070	933910
479	763690	748240	705380	661180	617120	575070	837310	1053410	995460	917530
480	755790	734610	693390	642930	603420	562340	823720	1034910	981150	900890
481	740220	717500	681370	631680	596480	549030	808420	1012620	957330	887480
482	721880	707150	669280	623540	586700	544980	799800	988990	942090	862100
483	710210	697560	657600	610630	573130	531690	781300	979100	931510	848620
484	701990	685200	651050	601490	563680	525300	766210	955210	900980	829650
485	692260	675290	637620	590870	545290	512670	754620	942180	889100	819760
486	677480	661580	626070	578640	542440	502310	741370	922310	871830	797830
487	671790	653760	618420	570350	530610	496030	724020	904040	858410	789490
488	658060	642280	605710	562950	528110	488280	713080	889680	843070	771950
489	648190	625990	591540	552640	509560	478890	699050	873480	823010	756190
490	634760	616480	584480	545490	500500	467630	681800	856700	811030	742840
491	622630	610040	575360	534560	496590	463350	674490	841960	795990	725760
492	615470	599790	566880	525230	488740	452140	660700	825610	781380	716660
493	601430	588210	555160	517120	477050	445560	645230	815770	761960	702190
494	595800	580640	547670	504620	474120	438290	635280	792280	754000	685580
495	581270	569720	541960	499550	465540	427430	625830	785460	738020	672770
496	572890	560030	522470	486840	452890	422480	612270	764280	722430	663310
497	564800	545910	519200	478720	445580	414800	604040	754330	710970	645620
498	557570	543470	509770	470220	437560	405910	592620	742420	694670	638800
499	543840	531540	497480	461440	434530	396840	581440	724360	684450	633700
500	534230	519980	491750	452160	422710	389560	569640	713640	670430	617130
501	525700	516960	483200	448550	415560	384200	557930	697660	653640	601520
502	518160	500830	473500	434350	405790	380050	546270	681990	649900	590480
503	507340	496320	469020	430840	398910	366520	540900	675400	631390	585140
504	499310	483800	459340	423640	391920	361860	532630	659670	622240	572960
505	490860	477190	454180	414290	385160	359940	518460	648310	612810	563670
506	479630	467220	441450	404220	379380	346690	507140	635720	598930	547770
507	473670	461440	431780	397670	372300	342110	501290	624170	587510	536820
508	463890	450220	428160	392360	361460	336210	491110	610330	572370	531460
509	455280	443650	419450	380630	359480	331900	480460	601490	564010	514500

510	450610	440690	411860	376880	350880	325750	474960	586530	550640	503350
511	435540	427860	400810	371380	341840	317080	460020	577490	541070	498010
512	427540	422870	396160	368310	336730	314350	455450	562020	531280	486110
513	424440	414340	389510	357720	331040	305980	445200	555000	519150	471250
514	415870	402970	376600	349630	326530	299670	434770	537030	511120	468560
515	408600	397250	372020	343620	319830	293590	426700	525960	496180	454940
516	404250	385290	367130	335340	311540	290200	419610	516190	484740	445350
517	393520	384910	357400	331200	305910	283470	409840	507060	480500	437110
518	381980	375530	349700	323890	302800	275120	400070	492840	463880	425440
519	376470	366320	344100	317560	293720	272950	392610	484020	458220	417650
520	368010	359330	341060	309230	286710	262640	387760	477520	449480	410130
521	361850	354940	330700	300300	280090	259510	377300	465200	439000	399080
522	351910	343070	325560	296370	277390	252750	370480	455020	426950	391760
523	344630	336150	318970	292680	270230	247580	359150	445820	419780	385360
524	342480	331210	311670	285310	263550	239350	351630	437050	410870	374650
525	330760	321540	305080	278670	258130	237570	342240	426710	400400	369420
526	325910	318050	299720	272160	253190	233360	337780	415530	392580	357140
527	317280	311500	289220	265150	246840	228630	329900	407940	386020	351110
528	312020	303620	286150	261290	243170	224190	321540	395930	375320	337170
529	307660	297990	279380	257470	235670	216550	313520	387120	369650	332320
530	298650	291350	273650	250310	231010	213430	305830	377390	357150	323600
531	293610	283740	266140	246740	225310	209040	300630	370100	351420	318560
532	284950	281180	262860	239050	221380	204390	295950	360220	339100	309600
533	278430	274470	255720	235930	216520	198570	288040	353030	333670	304630
534	274180	267170	248600	230960	214090	195710	281060	343620	326930	296400
535	270240	262240	245020	227230	207980	190290	274840	334460	317710	290110
536	263030	255150	241410	218590	201940	189280	266400	330060	312780	285180
537	253940	250030	235400	212590	198550	181710	262990	323840	305290	275400
538	250910	246370	230800	211170	194520	178730	256030	313330	298850	272660
539	246990	240290	225450	204990	188400	174530	251320	307040	288870	262830
540	239900	229970	217850	199790	184980	171050	245090	300720	284330	257600
541	237920	228610	212820	198650	180360	162750	236910	293110	276620	252440
542	228900	222750	209420	190680	176110	161060	232000	285610	267900	244180
543	226770	215580	206760	186990	172580	157910	226710	276900	261230	241800
544	221990	214290	199410	184960	172680	156240	220430	269300	257880	238360
545	212000	210530	197380	181780	166820	150160	218300	266930	248280	227190
546	210270	203150	190600	178780	160190	148600	213220	260070	246220	222010
547	207250	199900	187750	172980	156850	144640	206730	255920	238090	216770
548	199820	195400	181850	168900	153710	143870	204420	246370	233570	211800
549	197460	192240	179020	163610	152870	137870	197310	241060	226710	208130
550	192610	186690	175110	159720	149520	135760	192250	235240	222540	204800
551	186160	181280	169610	156860	145750	129990	186030	228480	215900	195690
552	184270	177650	167700	151720	143320	128860	181030	224960	212330	195390
553	179570	173150	163530	152110	138020	125560	182010	220140	207380	187460
554	172530	170060	159290	146760	135660	122740	175110	214470	201850	183610
555	170680	167360	155610	142240	132410	121280	169530	208900	195120	180870
556	167010	162140	151450	141250	128240	117310	167260	205620	192090	173510
557	164450	162030	150840	136850	124680	113990	161930	200320	187280	169910
558	163620	155010	146610	134700	125190	112040	159380	195270	182690	170680
559	158000	153090	143750	132790	121840	109090	154850	190690	176840	165090
560	151890	148890	140320	128580	119100	109020	150600	185350	175440	161010
561	149970	147170	137110	125930	115170	104230	145860	183100	169930	152810
562	146020	144070	136010	123220	114220	101770	146210	175270	169380	152890
563	143880	139820	131140	121270	109260	100720	140010	169790	160970	147960
564	141680	136270	130150	117430	109780	98060	139400	168390	157980	144050
565	136630	132920	125170	114800	107270	94640	134780	165320	155780	140330
566	137800	130440	121300	111450	104090	94910	134240	159440	150250	139870
567	129930	128570	120790	110070	102340	93400	130480	156240	145930	135870
568	128110	127060	115910	107500	100170	88600	125920	153370	142930	130060
569	124780	123680	115730	106770	96770	88560	122850	148010	139130	129780
570	121070	120430	115370	101850	94900	84380	118530	145230	137470	125590
571	120440	116680	110920	102640	92950	85110	116490	141080	134400	123270
572	119070	114700	108460	100090	91110	83570	116060	138800	130300	119590
573	116870	112840	103550	95830	88540	80690	110990	134780	127710	118190
574	113030	111450	102270	95290	88500	79400	110700	132370	124750	112670
575	111270	107880	99500	90540	86180	77110	105040	130970	122050	109250
576	107760	104230	98800	90600	84380	73960	102930	125140	118990	108440
577	105530	103270	96080	88880	81350	71900	103170	120980	114130	105830

578	103610	99270	93360	86330	78160	70500	98060	121460	112640	102450
579	101440	100450	92010	83380	78690	70020	96020	117020	108860	102230
580	97640	94350	90080	82540	74880	68340	92540	114600	106940	95950
581	97480	92540	87430	79020	72670	66630	90340	111270	106200	94540
582	93900	91050	85990	79190	72600	65120	91400	107810	102940	94180
583	92450	88620	83700	75800	70150	62620	85920	107620	97940	90410
584	90570	86360	80910	74040	69340	61160	84850	104540	96230	88830
585	85880	85510	80430	72000	67280	61050	83450	99820	93110	86910
586	84720	83490	78800	72000	65510	58100	81680	96960	92080	83310
587	84480	81820	77350	69590	64650	57560	80230	94160	90430	82110
588	82890	78580	76360	67850	63530	55830	77910	93210	88770	78550
589	80970	79650	74220	67600	62550	55140	75280	91990	86150	77200
590	77620	76710	71440	65630	60680	54680	74090	89080	83770	77430
591	77320	74480	69970	62760	57940	53630	72280	86050	81980	74140
592	75030	73520	69550	63880	58080	50890	71120	84740	79810	73640
593	74310	72360	67170	62720	57700	51190	69150	82940	77690	71310
594	72120	72400	66270	58770	55700	49390	66950	82300	77970	70120
595	71800	69500	66500	58010	54330	47870	66220	78200	74640	68410
596	70660	67430	63320	57770	52500	47590	64800	75340	71130	67080
597	68470	66260	61990	55910	52930	46240	63140	74790	72490	66060
598	67470	65470	59920	55730	52740	45530	60920	73580	70440	62700
599	65000	63910	60070	55940	49950	44830	59480	70990	67230	63340
600	63650	63450	58080	54020	48250	43490	59140	68650	65510	59590
601	62360	60950	56500	51710	47670	43350	55540	66980	63610	57650
602	59670	60120	55490	51960	47650	42300	56500	65430	62790	57520
603	60470	57880	54430	49470	46170	40660	54650	64670	60290	54940
604	56870	57420	54030	48490	44500	39520	52380	63290	59550	54750
605	57800	55180	52540	47690	43580	38890	52180	62520	58130	52330
606	53000	54420	51540	46640	42970	37680	51010	60720	56590	52150
607	55030	54470	50620	45890	41360	37030	50090	57760	55290	49530
608	53790	52660	49310	44870	41650	35610	48460	55920	54090	49150
609	52050	50630	47080	44120	40570	35880	46220	54710	53100	48670
610	51330	50260	47630	43030	38940	34020	45330	51870	50560	46410
611	51190	48720	46400	42320	39700	34470	44450	53650	49340	44530
612	49300	47310	44860	42190	39070	32520	43780	52780	48470	44340
613	48090	48120	44330	41370	37100	32570	43120	52030	48030	43300
614	47390	45410	43340	39230	37600	30990	41720	50240	46830	42460
615	48100	45390	41590	39440	36080	31410	40950	48010	45730	41560
616	45280	44920	41840	37960	35270	30970	40450	46780	44350	40360
617	45410	44460	40670	38990	33800	30390	39560	46120	44070	39980
618	43980	42080	40680	37540	34950	30100	37820	43640	41720	38040
619	43790	42000	38880	36320	33580	29070	38240	43800	41380	38010
620	42130	42190	39420	35460	32290	27140	36740	43470	40670	35900
621	42440	41020	37640	35240	32950	27910	35780	41370	39960	35120
622	41180	39120	37980	34300	31920	26870	34490	40910	38500	34760
623	41810	39800	35850	35190	30690	26440	34040	39060	36560	34340
624	39530	38800	36380	33220	30550	25530	34510	38250	36970	33130
625	37740	37240	35870	32620	30230	25530	32570	37190	35870	32570
626	38240	36540	34330	31960	29630	24560	32030	36910	34950	30720
627	36980	36670	33120	31140	28660	24960	31610	35500	32950	31080
628	37090	36050	33990	31100	28250	24320	30260	35160	33970	30910
629	36030	36470	33120	30480	28030	24200	30340	34070	31640	29390
630	34070	35270	32390	29890	27240	23350	28820	34030	31070	29110
631	35220	34030	31200	29510	26710	23280	29290	32860	30600	28930
632	33800	32600	31960	29530	26640	22610	28060	32880	30220	28150
633	33210	32250	30440	28170	27130	22150	27840	31720	29570	26480
634	33990	32520	29680	28450	26020	21710	26500	29990	28930	25900
635	32470	31180	29790	26500	25240	21050	26440	29370	27290	25210
636	32920	31190	29080	27190	25060	21000	25340	28830	27150	24660
637	32730	32040	29180	27720	24720	20730	24550	29010	26720	24620
638	30700	30020	28810	26190	23350	20720	25600	27270	25010	23820
639	29920	30330	28100	26190	23230	18470	24010	28090	25730	22990
640	30160	29200	27440	25010	23750	20200	23820	25510	24970	23210
641	30060	30120	27290	25200	23470	19640	22720	25780	25060	22100
642	29590	28990	26280	24560	23330	19420	23840	25790	25100	22330
643	29900	28160	26450	25160	22980	19170	22050	25870	23260	21670
644	28560	28550	27260	24730	22540	18460	21980	24090	23570	21870
645	27860	27810	26880	24550	22650	19050	22160	24540	22800	21210

646	28420	27850	26830	23640	22310	18750	21710	23730	22860	19900
647	28610	27500	26150	24250	22380	18860	20800	24460	21870	19400
648	27130	27030	26470	23590	21140	18470	20290	22530	21520	20420
649	28180	27050	25050	23370	21950	17320	20870	22540	21700	20210
650	27550	27280	25440	23160	21510	17800	20290	22390	21770	18880
651	26670	26460	25080	22380	20450	17220	20580	22310	21140	18560
652	27320	25500	23900	23300	21250	17400	19860	22250	20640	18040
653	26820	25770	24960	22560	21180	17650	19610	20890	20420	17530
654	27280	25260	25030	21800	20280	16700	19050	20930	19740	18060
655	26930	25520	24620	22580	20310	17100	18290	21190	19410	18160
656	26060	26210	23990	22560	20540	17450	18520	19870	20080	17620
657	25420	25260	23640	21240	21010	16930	18600	19890	18530	17430
658	25430	25010	23870	21940	19280	16120	18230	20080	18300	16990
659	26210	25000	23170	21640	19760	15950	17940	18940	18670	16680
660	26000	25360	21630	21830	19530	16060	17790	19310	17940	16220
661	24820	24860	22960	21890	19660	16210	17130	18910	17580	16450
662	25760	25020	23510	21520	20510	15560	17610	17060	17620	15450
663	25170	25270	22570	21270	19890	15840	17220	18230	17870	15400
664	25350	24940	22880	21740	19550	16040	18150	18580	17050	15360
665	24740	25000	24000	21030	19100	15920	16840	17920	17870	14820
666	25080	23850	23010	21540	20240	15650	16690	17850	16420	16390
667	25330	23840	21420	20400	19280	15900	17440	17030	16010	15220
668	24390	25330	23180	21010	20220	16290	16490	17590	16620	14440
669	24610	24260	22570	20930	20500	15720	16650	17390	16310	14590
670	24800	25060	22730	21010	19460	15480	15910	16890	16420	14870
671	24860	24700	23750	20680	19570	15110	16280	17110	15730	14740
672	24450	24550	22950	19940	20090	15480	15920	17200	16640	13990
673	25710	24730	22700	21820	19590	15370	16520	17160	15960	14900
674	24900	23440	22700	21320	18780	15180	16310	15890	15890	14050
675	24310	24400	22750	20910	19560	15120	16380	16560	15680	14560
676	25310	23690	23130	21740	19400	15290	16030	16630	15140	13820
677	24460	24220	23690	21990	19700	15620	15900	17100	15270	13780
678	25720	24270	22820	21550	19340	15560	15390	17090	14930	14210
679	24680	25100	22830	21260	19260	15400	15810	16250	15240	13270
680	23740	24840	23080	21130	20040	15670	16340	16130	14800	14010
681	25130	24960	23510	20300	20100	15800	16190	15800	15130	13760
682	25900	25390	23710	21860	21060	16400	15650	16300	15540	13910
683	25990	25290	24060	22380	21330	16280	16230	16540	16000	13370
684	27050	26440	23640	23290	21270	17080	16580	16960	15110	13860
685	26860	26370	25080	23030	20680	16680	16650	16410	15180	14870
686	26690	27330	25820	23330	21500	16870	16760	16690	15040	14560
687	28340	27470	26960	23740	22180	17350	17430	17070	15290	13830
688	29210	28480	26810	24110	22010	18380	18620	16550	15290	14120
689	30240	28370	28050	25680	23970	18800	17880	17490	15630	13860
690	29530	29560	27950	25760	24460	18380	17640	17080	16250	14840
691	29810	30200	28790	26190	23940	18830	18420	17540	15930	15180
692	31680	29770	29560	28050	25300	19140	18920	18530	15830	15410
693	31740	31340	30240	27720	25880	19810	19090	17770	16700	15360
694	32220	30920	30550	28120	25150	19570	19510	18810	17570	14400
695	33160	32930	32190	28150	26250	20620	20170	18950	17450	15760
696	34120	33900	31460	30550	27480	21750	19650	18520	17780	15670
697	35730	35310	33020	31170	27500	21880	20930	18720	19260	16330
698	36720	35640	34260	31780	28190	21770	20820	20200	19010	17260
699	38400	36300	34770	32690	29700	23080	21340	20570	19190	17620
700	40940	38790	36050	35150	31520	23160	23260	20640	20180	18320

Table S17. PL strength of compound **4** in mixture of $CHCl_3$ and MeOH (20%) from 223 K to 323 K

Wave length (nm)	-50 °C	-40 °C	-30°C	-20°C	-10°C	0°C	10°C	20°C	30°C	40°C
380	326590	306610	277570	264260	236350	220470	192630	196150	193020	193950
381	322550	302250	278060	259250	231460	215680	191700	199180	194550	192800
382	323290	302860	278060	261890	233060	218770	192330	198900	195500	194110
383	326090	306420	278570	261120	238090	223360	199040	204660	202580	201230
384	334960	310440	288310	266400	241690	225250	201280	209420	206850	206160
385	348340	320390	295440	276630	252060	234230	208650	215170	212670	216270
386	358070	330470	300410	283850	258140	243120	215540	220010	220720	225370
387	370500	342820	305480	289150	268500	250140	222260	229630	230350	230020
388	384470	349050	317530	301690	270400	255230	228040	235960	235560	236890
389	394030	359870	325040	306470	280090	259380	235810	241390	240010	246210
390	412980	374790	335990	314590	286680	270640	243110	250300	252700	251480
391	420080	384590	347740	326630	296370	278610	244700	254840	256430	258200
392	435770	396990	356230	333640	302420	287990	254000	263530	263860	264860
393	448790	406710	364400	344030	311000	291540	263210	270320	271580	281700
394	459460	420520	372850	348950	321340	303660	268860	280910	279180	288780
395	473310	428500	383050	357720	328370	308020	277450	286260	289300	296150
396	485100	440110	388080	370730	336960	315460	282980	293690	298630	304480
397	498440	454310	398220	376650	347530	326600	294890	305600	310860	315360
398	513400	464210	412720	388340	354340	334930	301640	318280	323780	334010
399	523810	476960	419110	394360	365880	351670	318390	330470	330330	342490
400	536530	490130	435420	410310	378160	362490	325390	338510	346090	357060
401	557300	502800	452170	424590	391550	374610	341900	356720	362390	374710
402	576920	521750	467050	441710	404240	391060	354970	374230	379220	391300
403	594330	534230	486340	457210	421860	403780	368840	387890	398370	410320
404	622050	563450	501870	475360	443390	420720	388830	407890	415170	433830
405	644330	581530	520950	498530	458820	441760	403890	426360	434270	452090
406	666730	608060	547840	516270	475760	460170	424970	444310	453740	473960
407	699280	630530	569730	536490	498440	478620	443570	462620	468730	490160
408	724540	658410	586000	560090	519450	501250	459730	480580	490690	505460
409	749240	683580	616160	580230	539780	516520	476530	492700	505330	524780
410	774490	706270	629460	596910	552200	534300	488980	511040	520390	534750
411	799110	724000	651640	612180	568440	545440	497600	517250	527980	546370
412	821280	746220	664130	631880	582000	554570	509350	530010	539840	555110
413	837270	762660	686000	642990	593000	569330	522550	545810	549430	566510
414	859880	771850	690220	651800	601760	576820	529660	551460	558810	573340
415	871440	781210	697490	663250	609000	584990	532880	554600	563850	581750
416	877920	796360	707440	665730	618230	588340	537310	556270	563940	586460
417	880140	798170	713920	671300	623880	587420	541680	566110	573230	585380
418	890410	800110	713140	679260	631080	590980	541390	570400	576510	594400
419	900170	809300	720030	679890	624800	596660	549910	568520	574340	599290
420	894880	812570	722080	683440	630900	601300	546810	575940	582310	602310
421	900500	804530	725620	683580	629870	598540	549280	573020	577170	603410
422	900400	815010	727130	687380	634090	598600	553190	578450	582970	605460
423	906950	811370	728990	688450	628430	604650	552530	582210	588480	614850
424	901100	818610	724520	686260	637610	603590	556470	584960	586660	615440
425	906460	818250	729460	686730	635770	607860	560190	586500	597710	616810

426	900080	825700	736240	695750	642860	611700	561500	594080	599110	623840
427	913600	823910	734370	697810	650250	614390	567720	592960	595980	624610
428	917270	829400	739310	696400	647440	619850	566140	602240	608800	628660
429	922420	830210	744610	704510	653640	627430	575990	603550	612500	630250
430	929440	841070	750960	709700	653960	634030	580660	611270	621780	642740
431	936120	847240	757210	712650	658380	631560	581130	608910	619700	645530
432	933670	852280	757360	723200	670420	637010	588850	618640	621820	647540
433	946310	856440	766250	725410	672170	646810	592000	619560	624320	654910
434	957410	860920	771490	733970	674960	645740	595620	623910	629110	650170
435	954070	863970	770970	736510	680560	648730	592760	627310	632290	659260
436	957780	870230	780780	737780	682620	651900	599110	623510	632170	655280
437	970330	876480	782370	743380	684010	648570	597830	625100	637290	659140
438	971300	881840	783900	737390	681850	657450	597710	629920	636040	657370
439	967960	876080	782550	739110	685300	653160	600070	625490	635750	651820
440	970500	874490	785650	735510	684920	653630	594910	622460	631280	649290
441	967230	873000	780780	736710	678120	646820	593950	620730	626090	643830
442	966480	871670	774910	731720	683830	644260	588320	617920	616470	638040
443	961290	867960	775580	729540	673130	641610	585330	610840	616640	632990
444	952800	854440	767750	729800	671120	640820	579880	603410	607280	629160
445	952950	859170	763430	720690	661410	631840	572450	602330	597550	625120
446	943700	850520	761010	715750	657090	628040	575180	597810	600640	615310
447	938850	844590	750210	712330	649990	619130	568090	589150	595970	613740
448	933150	841610	739980	698720	647720	619370	558470	581120	588560	604120
449	922780	829550	738610	693010	639060	612220	554850	580820	582300	601880
450	908340	822430	735780	688730	634710	604150	545210	577360	578980	594820
451	905960	819320	731280	682290	633850	600590	540970	567710	569820	586970
452	897130	812140	718880	679300	624180	594560	541250	563330	564650	583460
453	891940	800840	716520	668710	616580	588770	534050	552540	561550	574540
454	878510	797180	708710	665630	614700	584370	527430	553460	555140	573170
455	871160	794520	699460	663790	605440	579130	524280	550480	551130	567990
456	865410	774950	694130	658050	600990	569650	518870	545410	543750	563880
457	859090	774750	692740	650540	596290	564530	513550	538920	547790	557180
458	852870	769670	683990	653030	595270	563490	512780	531910	530730	551400
459	849850	763550	681190	638370	588800	559570	509570	526080	533650	546430
460	845240	762280	674830	638480	586530	556300	510350	524600	528580	547430
461	838800	751990	672430	636410	584630	553380	500670	525300	524240	536240
462	831960	752370	674530	631840	580710	547840	497740	517070	521760	535280
463	823610	751910	667210	625140	580120	546780	493620	510620	515030	528430
464	825830	745280	659990	625360	569920	544070	492860	509270	512610	524420
465	818600	733690	661080	614140	567430	542450	487000	503350	507630	522210
466	813570	735290	653950	608620	563170	529730	484390	498600	501290	513830
467	802600	733380	645750	608500	559380	527850	478820	492970	497180	506460
468	799980	726130	636230	603240	553850	526340	471490	484600	492190	495960
469	791530	711470	633730	592910	547230	514400	463500	480230	482480	491530
470	778080	706660	625530	584210	542530	508270	459640	473220	475460	486080
471	769850	698570	613660	576020	529540	502670	452350	467390	466460	474900
472	758830	683950	606370	569040	520420	491770	440590	460170	458850	471110
473	755060	679040	603230	562690	515370	487090	435000	450090	448050	461330
474	738850	665350	590160	553650	508840	479340	424710	441410	441460	450300

475	727680	657280	585660	550980	493620	467460	421570	435340	436110	443300
476	720760	650570	570870	541110	490120	464170	413000	419800	430700	439310
477	710140	642920	573340	530110	482820	456140	409230	421930	418370	426620
478	701740	633070	559520	522820	474180	451800	401230	412620	411500	421700
479	696150	625240	558790	515470	469450	445600	396740	406840	403770	411930
480	685080	617880	548160	509840	463400	434470	386630	400590	396020	407490
481	676950	608340	536980	498840	457090	427000	380840	392120	390800	401030
482	664350	599940	528510	493470	448460	425330	374790	387560	387200	393250
483	654120	593860	526270	489120	442690	419180	371760	381550	380200	387410
484	651700	587670	521500	482260	435540	412120	369120	376230	373840	381540
485	641950	578530	507100	474600	429960	406610	358020	367400	368130	371580
486	634830	571280	504670	467470	422850	400300	355680	362480	363210	367340
487	630510	563620	496490	458340	421960	394830	350170	357620	356780	361290
488	615940	558210	494680	455190	416270	389870	347650	355670	350580	359290
489	616090	545820	478390	449600	407130	382690	339610	347640	345130	353430
490	608050	542900	477100	446690	400980	376740	335500	343160	339210	346070
491	597480	542600	478140	443100	399940	373580	329190	341000	337350	342650
492	593110	534940	466790	437610	394660	370670	324140	330910	328890	336230
493	586550	527480	462810	431630	389950	367070	322610	326620	324390	335130
494	579300	526520	458470	426110	383080	359640	319760	326560	318610	328050
495	573790	512910	453640	422630	377330	357150	310400	318190	318940	322120
496	572070	511340	449190	418320	375560	351700	308750	312540	312660	315750
497	563490	500020	441360	407720	367280	345650	304730	311070	306720	315960
498	557110	497880	441460	405140	367600	340790	298470	307000	298970	308740
499	552460	496330	432920	400580	359270	337640	295980	300980	296030	302470
500	544330	487330	425810	395390	353390	331990	291680	294050	292920	299570
501	537950	478400	424370	390320	350480	330670	286450	295380	287140	288720
502	533440	477920	412610	385370	348760	324530	283420	284480	286470	289200
503	527850	471190	414810	375120	341020	314590	274700	283780	276190	282850
504	522060	465320	407250	378860	338790	312660	274720	278600	276140	279270
505	514680	454550	400790	372560	331630	309360	270210	276430	268880	273160
506	510850	451030	397050	371600	328420	305160	264050	271510	266330	267220
507	503150	446130	394340	360650	323460	302600	263400	266430	259840	263050
508	493480	441060	386420	359790	319830	296360	253750	262840	258890	259160
509	488700	435510	380720	351930	313030	291480	256490	254460	253610	255340
510	480730	433720	377530	347610	309040	288780	250440	251850	248960	251170
511	478320	425500	369410	343500	308150	283180	246730	247230	245180	248950
512	473350	424360	365730	337830	298990	278570	240050	244060	238270	244560
513	460830	414760	358110	333110	295150	272140	234990	239940	235550	238000
514	456650	410990	353900	327780	288740	267720	232120	234860	230390	233780
515	449370	399680	353350	320240	284450	265370	230220	229660	226810	229450
516	447430	393590	343920	316270	281470	259810	227260	225190	224260	226150
517	435950	389310	343430	314170	276170	254700	221370	225700	218380	219210
518	430090	385780	337510	306270	270020	250750	218850	216280	215450	218400
519	423500	378650	327490	304180	270420	244600	214130	213950	213870	213030
520	415580	373000	327310	299460	260450	246640	208410	210600	204910	210960
521	411570	366510	317630	293850	258150	238220	204760	208240	203360	204880
522	405100	360170	313690	289240	254450	236260	201990	202760	197170	201610
523	399080	353590	307110	280930	248040	230440	198780	200010	194370	196180

524	396360	347300	301020	277280	242350	224480	194610	194610	191330	196590
525	388090	342530	297400	272390	238200	222710	191130	192500	188910	187400
526	381680	335790	295300	269230	236220	220910	186110	186450	186100	187930
527	373430	332930	288910	262550	234660	213440	183040	186400	182200	181220
528	367500	324890	281670	258990	226770	212560	181630	182630	176920	179400
529	359760	322630	277010	248380	224620	206360	175070	176450	175080	172950
530	354280	313990	270970	252160	218870	204440	175440	174780	168830	169450
531	348120	304670	269010	243900	215700	199190	171220	170150	163170	168470
532	343340	305760	263290	239140	207880	193650	168320	169920	161110	165270
533	338550	298730	258890	235460	206030	192740	163200	162940	159430	160160
534	330960	293440	254520	230720	203580	186520	160440	161700	157110	158800
535	321860	289260	249540	226670	200010	186720	156930	157040	153630	156040
536	319380	280700	244100	223330	197170	180330	154900	153290	150570	152610
537	312290	275300	241070	219650	190480	177680	152270	154920	147300	147010
538	309590	274120	232070	215710	190060	172820	149650	147380	147660	146300
539	302170	269430	230950	210780	187110	168030	144800	145190	141510	141800
540	294550	262530	224510	208500	181310	168390	142830	142350	139270	136150
541	293330	256580	222770	202770	178670	163400	139210	138230	134470	137700
542	286570	252620	218240	201070	175850	162510	136420	139770	134020	133070
543	280210	248480	215640	196060	171410	156760	134280	135580	130620	132500
544	272760	242670	209260	190020	171280	156340	129750	131550	127670	127970
545	268940	241700	205510	187500	163710	152500	127920	129900	125050	124530
546	267280	236110	200110	183740	164160	146220	127070	129060	121600	121540
547	262540	229690	197500	180810	159410	145170	125610	126820	119660	120930
548	255500	224710	195430	177370	156400	141490	123070	122710	118050	119120
549	248210	222840	189430	176300	152180	140660	117770	118580	115990	117140
550	246470	215350	185860	171230	147200	136180	117640	116300	113770	110290
551	239790	214250	184430	169840	147310	134200	115950	112930	111450	111090
552	235500	209290	179160	166000	142810	131370	111080	112790	107760	106700
553	232560	205360	177610	160890	141700	130030	109610	110250	106430	106590
554	228480	201330	170600	159080	135680	126580	107210	107060	106010	104070
555	226340	195660	168920	155260	132910	123190	106010	105750	99000	101560
556	219010	191710	166920	152420	132930	121590	102450	103990	99030	98250
557	213400	187480	161720	149180	129340	117900	101390	103280	98350	95760
558	209520	186660	161110	145970	125940	116940	98650	97430	95930	94670
559	204400	181620	157610	144040	125700	113260	97120	96150	94170	94200
560	205140	176970	154020	139310	123660	111070	95570	96550	92390	91600
561	199480	178050	151560	134220	118710	109650	93710	91910	91050	89280
562	196050	171020	148620	135000	116920	106920	91720	90890	87580	89670
563	191350	166760	146060	131560	115810	104880	89040	90460	87190	87170
564	188350	162920	142890	129010	114160	102300	89230	86430	84460	84110
565	184260	163950	139120	127420	110880	100870	85150	88660	83610	83140
566	178380	161890	138550	125120	107480	101420	85260	85440	80510	82120
567	179050	153350	134230	123170	106240	96620	81360	84380	78530	79280
568	174920	152520	132850	120410	104400	97530	81600	80440	78410	78140
569	171390	150810	130520	118610	103070	92560	79420	80230	76390	76840
570	169180	149600	127340	116140	101130	92440	77260	76880	74000	75420
571	163010	143670	124770	113910	98100	88690	76330	76800	74430	72480
572	161530	140040	124260	112570	95450	88620	74610	76390	71780	70770

573	156200	136760	118240	109400	96430	84990	73450	73410	70690	69710
574	154450	136740	117960	109670	93180	84660	72920	72720	68980	69430
575	152240	134370	114680	104300	91410	82300	68740	71240	68600	68530
576	147130	130090	113280	100850	90310	80860	68300	69140	66720	66790
577	143700	130870	109650	99560	86530	81150	69000	68190	66080	64540
578	143820	124450	105990	99840	84190	76200	66940	65720	63840	63130
579	138060	121370	103770	96890	82880	75560	64390	64850	62710	60850
580	135700	120470	102850	93860	82870	72950	64190	63580	62710	59290
581	132480	115640	99250	92330	80420	72350	62800	62720	60590	60740
582	129700	114370	99370	88710	77120	70320	60170	60750	58850	58480
583	128270	113660	96920	88290	77550	70080	62210	59010	58130	56520
584	126160	110280	95360	86440	75030	67910	59340	58850	57520	55250
585	121590	109200	93310	85480	74790	68980	56500	56980	55350	54710
586	120810	104890	92080	85030	71980	65850	55440	55630	55260	54870
587	118780	103590	88630	81340	71480	66000	55380	54380	53640	53080
588	116130	102600	87880	80870	68460	63370	53400	53310	52010	50220
589	114080	100550	86060	78320	69360	63980	53440	53030	51350	51420
590	112430	98820	84620	76690	67670	61280	52950	52890	51160	50760
591	109680	95850	83850	75830	65700	58870	51220	51290	49210	49090
592	108690	96620	81550	74070	64560	59330	49820	49900	49220	48430
593	107520	93460	79670	75210	63980	59130	49450	50560	49170	47000
594	103420	93620	79590	73440	61140	57280	50320	50160	46780	47630
595	102080	89490	76050	70720	63210	56470	47180	47460	46770	45960
596	102640	89170	76590	68700	60510	55190	48640	45620	46880	46170
597	101130	88280	74630	68410	59260	54040	45580	46120	44560	44890
598	96040	85000	74730	66900	59390	54670	46030	45460	43580	43170
599	97300	84160	72750	67090	57470	52890	44450	43110	42830	42360
600	94180	82960	71780	64790	55710	50740	43880	43210	42200	42310
601	92530	81370	69660	64840	55010	49810	43970	43590	41340	40790
602	90170	78040	68990	63150	54590	50260	41600	43850	41340	39800
603	88590	76830	67090	61310	53190	48390	42110	41560	41430	39300
604	85480	77310	65970	60750	50940	48670	40860	39370	38760	38210
605	86550	75600	65300	59410	51250	46910	39430	40460	37100	37960
606	85120	75350	63990	58600	52030	46180	39180	39930	36790	38310
607	83180	72560	62500	57180	49670	44210	38440	38620	36750	36990
608	81620	72860	60740	56030	50520	44960	37180	38880	36160	35870
609	80310	71070	59710	56020	48630	44120	37030	36020	36490	35000
610	79160	70470	59760	54660	47500	43530	37130	37550	35560	34710
611	79460	68540	58090	53740	45840	43070	35940	35680	35320	33140
612	77510	67930	58570	52010	46380	41710	35400	37030	34780	33580
613	75720	66470	56730	52030	45790	40950	34550	34390	34400	32470
614	73700	65720	57250	52250	43750	41150	35430	34550	33200	32840
615	73220	64590	55090	51270	44070	39470	33730	33400	32340	31630
616	72660	64940	54770	49810	43160	38410	33930	34200	32480	31830
617	72320	63100	54040	49960	43070	38470	33180	32840	31920	31570
618	69850	63020	53390	48100	42540	38370	32930	31770	31760	31250
619	69180	61020	52300	47510	40340	37980	31270	31810	30970	30050
620	67450	60120	51930	48270	41770	37260	31870	30510	30000	29570
621	68730	61500	51530	46540	40500	37480	31980	31510	29570	29220

622	66700	57090	50450	44770	39370	36530	30360	30020	29380	29270
623	66450	58080	49420	45900	39130	34780	29620	30310	28580	28790
624	65020	57740	49580	44260	39140	34620	29570	29970	29380	27800
625	64180	55630	48390	43670	38640	35320	30500	29220	27940	28990
626	62940	55270	47180	43200	37220	34370	30550	29350	27960	27620
627	62490	56510	46830	42770	37560	33750	28700	29140	27100	27960
628	61330	53740	47300	42860	36680	33090	29130	28720	26240	26710
629	61750	55070	46300	42230	35970	33040	27270	27880	27320	26460
630	60350	51610	45590	41810	34650	32950	27250	26670	25930	25980
631	59490	51720	44800	39540	34930	32310	27890	27370	26190	25340
632	57500	52140	44130	40170	35390	32720	26720	26320	25120	24510
633	57810	51220	43600	40190	35690	31080	26470	27440	24350	24840
634	56900	50130	43400	38820	33480	30780	27470	26610	24630	25070
635	56210	50490	42000	38560	34340	31010	25750	25700	23700	23530
636	57150	48340	41470	38410	33140	29340	25960	26350	24510	23940
637	56100	48860	42470	36540	31690	31100	24680	25470	24410	24190
638	54130	47750	42100	36170	32870	29190	25050	25500	23520	22900
639	54500	46570	39930	37140	32270	28680	24870	24610	23240	23140
640	53440	46850	40360	36920	33020	28700	25090	24640	23320	22770
641	54350	46500	40170	35750	31490	29850	25520	24710	22990	22190
642	53680	47240	40360	35110	30850	28790	24320	24230	22920	22300
643	51250	46340	38210	36650	31450	29400	24370	23700	22840	22480
644	51290	45620	39040	36090	31290	29690	23890	23480	22410	21850
645	51560	45150	39750	35730	29780	27350	23930	23450	22350	21930
646	50670	44980	39530	36390	30750	26890	23310	23500	22550	21940
647	50670	44220	36690	35640	31100	27980	22540	23160	22470	21760
648	51020	44030	38220	34740	30520	28120	23400	23200	21310	21760
649	51470	44310	38150	35640	30180	28660	23130	23700	21840	20490
650	50290	43340	38740	33090	29780	27360	23000	21910	21890	20690
651	49610	43970	38100	34090	29530	27220	22900	23030	21470	20600
652	49210	43030	38390	35160	30620	26710	22640	23640	21830	20730
653	50160	41510	37450	34170	28520	27930	22840	21960	22380	20620
654	50020	42940	36620	33520	29340	25860	22020	22830	21010	20240
655	48740	42510	36380	33900	29170	26380	22180	22350	20340	20720
656	47800	42040	36860	33300	28600	26970	21700	22430	20590	20260
657	48700	42790	36440	34350	29150	25870	22120	22560	21260	20380
658	46670	42290	35350	33270	28920	25380	21900	22260	20660	19710
659	46840	42640	37250	33410	28440	26020	21570	21590	20740	20230
660	48090	41940	35800	32960	27520	26570	21850	22380	20640	19830
661	47320	41380	36010	33090	29040	26140	21600	23160	20410	19860
662	48300	40600	35920	32930	28440	26420	21020	22020	20660	19070
663	46540	41170	36370	32640	29220	25810	21590	21240	19950	20230
664	46680	42380	36510	33010	28400	26750	21160	21750	20090	19570
665	47290	39890	36020	32760	26810	25590	21360	21900	19870	19750
666	45340	41590	36580	31790	28750	25190	21490	21100	19310	20420
667	46490	42080	35160	32140	28590	25930	21580	21150	20300	19950
668	46070	38870	35890	32120	29200	26250	22090	22020	20570	19350
669	45750	39600	35160	32200	28590	25500	21700	21910	20780	18320
670	45490	39640	34980	32240	29050	25560	21490	21350	20690	18910

671	46150	39420	35520	32950	27550	25780	20300	20540	19700	19470
672	43830	40370	34110	32130	28130	24540	21500	21290	19950	18930
673	45060	41350	34200	32940	28140	26120	20700	21470	19670	18890
674	44820	40380	33970	31570	27840	24250	21060	21200	20560	19670
675	43550	39520	34840	32410	27700	25840	20780	20720	20410	19220
676	43870	38760	33480	31870	28110	24560	21030	20900	19780	19640
677	44280	39160	34660	31530	28090	25530	22040	21350	19820	19200
678	45150	38240	34370	31750	27300	24670	21300	21630	19990	19290
679	42890	38710	34590	32020	27480	25050	21350	20960	20600	19030
680	42930	40230	34610	32060	27540	25080	21590	21900	19560	19970
681	45050	37580	35170	32820	28210	24940	21940	21650	19840	19020
682	44350	40850	34590	32260	28910	26520	22450	21110	20740	20000
683	44770	39740	34960	33490	28690	26690	22140	22850	20470	19650
684	44300	39860	35690	33470	29510	26220	22520	22380	22270	19700
685	44100	42770	36280	33460	29060	25990	22430	22610	21220	20490
686	46660	41300	35290	33830	29660	27540	22930	23050	21620	21610
687	47750	41010	37200	34920	30490	27370	23610	23610	21950	21090
688	46830	40550	38610	35170	31060	27690	23500	24430	22580	21290
689	47940	43920	38060	35680	30640	28770	24070	24060	22420	21890
690	46850	43030	38920	36840	31490	29480	24790	25390	23610	22340
691	46830	44940	40500	36610	32250	29140	25230	25280	24160	23390
692	48990	45700	40010	37970	32960	30760	25990	25080	24260	22470
693	49330	43900	41180	38630	34210	29990	25400	26760	25210	22930
694	51370	46460	43090	39340	35280	31290	27220	26590	25620	24320
695	51670	46360	42500	39530	34930	31680	26480	26860	25600	24490
696	51420	46780	44550	40530	37280	32300	26890	28070	26020	25100
697	52720	48120	43990	40940	36030	33990	28740	29340	26990	24930
698	53660	49030	44920	42500	38120	33760	28630	28860	27770	25100
699	54800	50390	46450	42860	39080	35400	29940	30080	27740	26540
700	56160	51460	46100	43000	39400	35720	30840	32370	28820	27680

Table S18. Crystal data and structure refinement for **38**.

Identification code	MeoMe	
Empirical formula	C50 H56 N8 O2	
Formula weight	801.02	
Temperature	273(2) K	
Wavelength	0.71073 Å	
Crystal system	Triclinic	
Space group	P-1	
Unit cell dimensions	a = 7.355(3) Å	α= 84.533(11)°.
	b = 15.211(6) Å	β= 90°.
	c = 18.611(8) Å	γ = 90°.
Volume	2072.7(15) Å^3	
Z	2	
Density (calculated)	1.283 Mg/m^3	
Absorption coefficient	0.080 mm^{-1}	
F(000)	856	
Crystal size	0.090 x 0.070 x 0.030 mm^3	
Theta range for data collection	2.199 to 26.388°.	
Index ranges	-9<=h<=9, -18<=k<=18, -23<=l<=23	
Reflections collected	49480	
Independent reflections	8412 [R(int) = 0.4049]	
Completeness to theta = 25.242°	99.2 %	
Refinement method	Full-matrix least-squares on F^2	
Data / restraints / parameters	8412 / 0 / 576	
Goodness-of-fit on F^2	1.024	
Final R indices [I>2sigma(I)]	R1 = 0.1302, wR2 = 0.3137	
R indices (all data)	R1 = 0.3360, wR2 = 0.4270	
Extinction coefficient	0.008(2)	
Largest diff. peak and hole	0.543 and -0.250 e.Å^{-3}	

Table S19. Atomic coordinates (x 10^4) and equivalent isotropic displacement parameters ($Å^2$x 10^3) for **38** ,U(eq) is defined as one third of the trace of the orthogonalized U^{ij} tensor.

	x	y	z	U(eq)
C(1)	6967(12)	-1465(5)	3760(5)	65(2)
C(2)	8357(12)	-1815(6)	4186(5)	78(3)
C(3)	8230(13)	-2683(7)	4464(5)	76(3)
C(4)	6734(14)	-3198(6)	4323(5)	69(3)
C(5)	5341(11)	-2851(5)	3894(5)	60(2)
C(6)	5413(11)	-1974(5)	3597(4)	54(2)
C(7)	4235(11)	-1405(5)	3143(4)	54(2)
C(8)	5099(11)	-605(5)	3035(5)	64(2)
C(9)	2427(11)	-1601(5)	2853(5)	66(2)
C(10)	1513(11)	-727(6)	2601(5)	73(3)
C(11)	4297(12)	201(6)	2624(5)	77(3)
C(12)	5600(12)	706(6)	2115(6)	81(3)
C(13)	4814(11)	1500(5)	1703(5)	63(2)
C(14)	5640(11)	2315(5)	1566(5)	62(2)
C(15)	4434(11)	2851(5)	1110(5)	60(2)
C(16)	4489(14)	3716(6)	780(5)	74(3)
C(17)	3064(16)	4050(7)	355(6)	91(3)
C(18)	1573(15)	3533(7)	256(5)	87(3)
C(19)	1433(12)	2658(6)	553(5)	73(3)
C(20)	2876(12)	2346(5)	972(5)	64(2)
C(21)	7469(11)	2489(5)	1865(5)	64(2)
C(22)	8370(11)	1615(5)	2111(5)	68(3)
C(23)	7748(15)	-4463(7)	5048(6)	108(4)
C(24)	3582(13)	900(5)	3152(5)	74(3)
C(25)	6298(12)	7(5)	1579(5)	71(3)
C(26)	1988(12)	6461(5)	6247(5)	67(2)
C(27)	3374(12)	6825(6)	5820(5)	85(3)
C(28)	3229(12)	7681(6)	5544(5)	74(3)
C(29)	1733(13)	8185(5)	5686(5)	62(2)
C(30)	317(12)	7864(5)	6106(4)	63(2)
C(31)	455(11)	6986(5)	6407(4)	53(2)
C(32)	-751(11)	6415(5)	6866(4)	57(2)

C(33)	117(11)	5618(5)	6981(5)	65(2)
C(34)	-2554(11)	6601(5)	7159(5)	67(3)
C(35)	-3463(11)	5717(5)	7411(5)	72(3)
C(36)	-678(14)	4813(6)	7414(6)	97(4)
C(37)	612(14)	4284(6)	7877(6)	86(3)
C(38)	-216(11)	3476(5)	8284(5)	65(2)
C(39)	614(11)	2684(5)	8423(5)	56(2)
C(40)	-571(12)	2144(5)	8884(5)	62(2)
C(41)	-511(13)	1283(5)	9212(5)	75(3)
C(42)	-1931(16)	947(7)	9641(6)	90(3)
C(43)	-3433(15)	1469(7)	9752(5)	84(3)
C(44)	-3559(12)	2328(6)	9445(5)	73(3)
C(45)	-2121(12)	2643(6)	9011(5)	64(2)
C(46)	2444(11)	2511(5)	8133(5)	72(3)
C(47)	3344(11)	3379(5)	7890(5)	70(3)
C(48)	2715(14)	9470(7)	4959(6)	106(4)
C(49)	-1403(12)	4098(5)	6848(5)	72(3)
C(50)	1301(12)	5003(6)	8420(5)	72(3)
N(1)	6715(10)	-625(5)	3410(4)	77(2)
N(2)	2718(9)	-97(5)	2179(4)	69(2)
N(3)	7189(9)	995(5)	2541(4)	64(2)
N(4)	3116(10)	1501(5)	1333(4)	73(2)
N(5)	1728(11)	5642(5)	6609(5)	87(3)
N(6)	-2282(9)	5100(5)	7834(5)	70(2)
N(7)	2180(10)	3987(5)	7451(4)	67(2)
N(8)	-1897(10)	3484(5)	8654(4)	75(2)
O(1)	6505(10)	-4076(4)	4572(4)	102(2)
O(2)	1494(10)	9070(4)	5434(4)	100(2)
